# 43rd International Symposium on Intensive Care & Emergency Medicine

**DOI:** 10.1186/s13054-024-04822-5

**Published:** 2024-03-19

## P001 Effect of prolonged emergency department length of stay on process of care measures of critically ill patients

### J Saoraya, ACKB Amaral, F Angriman

#### Sunnybrook Health Sciences Centre, Department of Critical Care Medicine, Toronto, Canada

*Critical Care* 2024, **28(Suppl 1):** P001

**Introduction:** Prolonged boarding of critically ill patients in the emergency department (ED) may be associated with worse clinical outcomes. However, whether this association would exist in settings that have implemented a critical care consultation model in the ED remains unknown. We thus sought to explore the association between prolonged ED length of stay (LOS) and the deployment of evidence-based processes of care measures for critically ill patients in hospitals implementing a critical care consultation model in the ED.

**Methods:** We conducted a retrospective cohort study including eight academic intensive care units (ICUs) in Toronto, Ontario, Canada (i.e., Toronto Intensive Care Observational Registry: iCORE) from June 2014 to February 2023. We included adult patients who were directly admitted to the ICU from the ED. Patients with ED LOS for more than 24 h were excluded. The cohort was divided into an acceptable ED LOS group (LOS < 6 h) and a long ED LOS group (LOS 6–24 h). We explored the association between long ED LOS and processes of care measures on day 2 after admission to the ICU using logistic regression. Multivariable models were adjusted for age, sex, comorbidities, severity, study site, and diagnosis.

**Results:** We included 7072 patients, of whom 1462 (20.7%) patients had long ED LOS. Both groups had comparable severity at baseline. In the long ED LOS group, more patients had cardiovascular, chronic respiratory, and chronic kidney disease as comorbidities and fewer patients were admitted due to trauma. There was no difference in the deployment of processes of care measures on day 2 after admission to the ICU between the two groups, as shown in the Table.

**Conclusions:** In this multicenter study of ICUs in Toronto where a critical care consultation model has been implemented in the ED, long ED LOS was not associated with a decrease in the deployment of evidence-based processes of care measures for critically ill adult patients.

**Table (abstract P001):** Process of care measures on day 2

Process outcomes	Long ED LOS; N = 1462	Acceptable ED LOS; N = 5610	Adjusted odds ratio (95% CI)
Low tidal volume ventilation	174/281 (61.9%)	643/979 (65.7%)	0.84 (0.62–1.13)
Spontaneous breathing trial among those eligible	245/575 (42.6%)	950/2493 (38.1%)	1.09 (0.90–1.33)
Extubation among those eligible	116/179 (64.8%)	480/685 (70.1%)	0.76 (0.52–1.11)
DVT prophylaxis among those eligible	751/968 (77.6%)	2353/3225 (73%)	1.15 (0.96–1.37)
Continuous sedation	723/1186 (61%)	3002/4609 (65.1%)	0.92 (0.80–1.06)
Physical restraints	503/1184 (42.5%)	1979/4590 (43.1%)	1.08 (0.93–1.25)

## P002 Development of intensive medicine in the emergency department: 1 year of experience in a tertiary referral hospital

### AR Leite Cruz, C Pires, F Faria, L Bento

#### Centro Hospitalar Universitário Lisboa Central, Intensive Care Department, Lisboa, Portugal

*Critical Care* 2024, **28(Suppl 1):** P002

**Introduction:** The assimilation of Intensive Care Specialist (ICS) into the Emergency Department (ED) is a recent development in the southern part of the country supported by the recommendation of the Order of Physicians (OM) for the presence of intensivists in the emergency room, under the organizational jurisdiction of level II/level III areas. This article aims to present and analyse the results of the first year of ICS support to the ED in a central hospital in this region.

**Methods:** The analysis of records was conducted using the ED application, covering the period from June 1, 2021, to July 31, 2022. Records made in the ED were considered, as well as data related to all patients transferred to intensive care units (ICU).

**Results:** During this period, the ED admitted a total of 155,786 patients, with 69,290 triaged (Manchester’s triage) as yellow priority, 10,750 as orange priority, and 721 as red priority. Out of 873 patients evaluated by the intensivist in the ED, 804 were admitted in the ICU, while 47 died in the ED, 28 of which did not require admission to ICU. ICS support was primarily provided to medical patients (52%), followed by trauma patients (18.9%) and surgical patients (13.7%). ICS conducted observations within the first 2 h of stay in the ED in 39.4% of cases and after 12 h in 15% of cases. Written records on-site were made in 56% of patients evaluated by ICS in the ED, making it difficult to define the moment of intervention due to posthumous records in critical patients.

**Conclusions:** The shortage of requests for collaboration in trauma patients can be attributed to the traditional management of such patients by surgical specialties and anesthesia. These initial results provide valuable insights to plan the restructuring of teams and training of intensivists in approaching these critical patients in the context of the ED.

## P003 Leveraging routinely collected data and large language models to inform patients about their emergency department stay: a feasibility study

### D von Wedel^1^, M Thiele^2^, D Shay^3^, R Leuner^1^, SF Bigdon^4^, F Balzer^1^, N Rutsch^4^

#### ^1^Charité Universitätsmedizin Berlin, Institute of Medical Informatics, Berlin, Germany, ^2^Charité Universitätsmedizin Berlin, Berlin, Germany, ^3^Harvard T.H. Chan School of Public Health, Department of Epidemiology, Boston, USA, ^4^Inselspital, University Hospital Bern, Department for Orthopedic Surgery and Traumatology, Bern, Switzerland

*Critical Care* 2024, **28(Suppl 1):** P003

**Introduction:** Large language models (LLMs) demonstrate impressive abilities in processing and generating text, with first studies highlighting their utility in creating discharge letters and answering medical questions [1, 2]. We assessed whether LLMs could be used to provide patients with individualized and easily understandable discharge summaries, including diagnoses and interventions of their emergency department (ED)-stay and recommendations on next steps, based on routinely collected data.

**Methods:** Anonymized data for 30 ambulatory ED-patients of a large level-1 trauma center in Switzerland was obtained. This study complies with the Swiss Human Research Act. Medical history, diagnoses, and interventions of the ED-stay, alongside physicians’ recommendations on next steps after discharge were extracted from patient charts using GPT-4 through the Application Programming Interface (Figure). Then, the LLM was prompted to combine information into an easily understandable summary. Two clinicians independently assessed completeness in the LLM’s reporting of a priori-defined items, which included aspects a patient must be informed about by a physician before being discharged. They further checked for the introduction of medical errors in LLM-generated summaries.

**Results:** The median (interquartile range) proportion of a priori*-*defined items reported in LLM-generated summaries was 75.0% (57.3–100). 8/30 (26.7%) summaries included all items. No medical errors were introduced by the LLM.

**Conclusions:** After prompt refinement and validation in external cohorts, LLMs may be an effective tool to create individualized ED-patient discharge summaries. Furthermore, LLM’s ability to easily translate texts to other languages might help communicate information to patients with limited proficiency in the local language. Inclusion of interactive elements, allowing for patient-chatbot interaction, could reduce follow-up questions, readmissions, and workload.

**References**

1. Ayers JW et al. JAMA Intern Med. 2023;183:589–596
2. Patel SB et al. Lancet Digit Health. 2023;5:e107–e108

**Figure (abstract P003):**
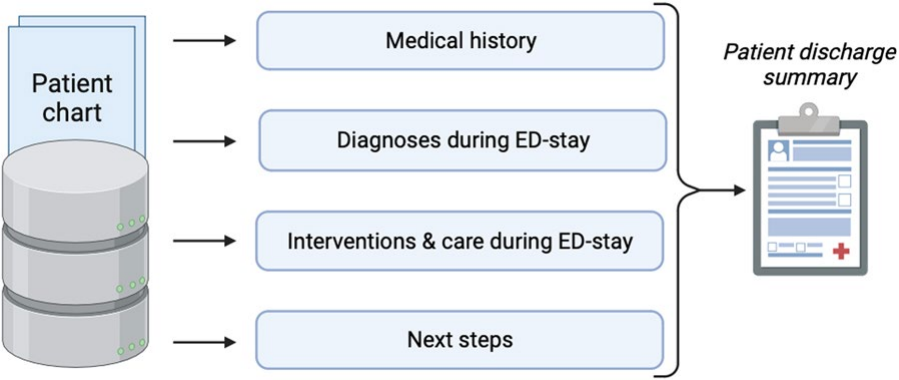
Creation of patient discharge summaries using a large language model. On November 17th, 2023, the large language model (GPT-4-1106-preview) was requested to extract relevant data from different data sources of the hospital’s electronic healthcare records, including patient’s medical history, diagnoses, interventions, alongside the next steps, recommended by the treating physician. Then, based on the extracted data, the large language model was prompted to summarize this information and create an easily understandable text, to inform patients about their stay in the emergency department. Standardized prompts were used and passed onto GPT-4 through the Application Programming Interface.

## P004 The ABC team at Gødstrup Hospital

### MB Jensen-Holm, RP Nielsen, N Dey

#### Gødstrup Hospital, Operation and Intensive Care Unit, Herning, Denmark

*Critical Care* 2024, **28(Suppl 1):** P004

**Introduction:** The ABC team functions as a rapid response team with the purpose of securing a fast ABCDE guided treatment of patients with an acute and/or potentially life-threatening condition [1–2]. The aim of this study was to investigate the use of the ABC team after the move from Regional Hospital West Jutland to the newly built Gødstrup Hospital. In addition, the chart review investigated if there was a relevant indication for activating the ABC team, and the number of subsequent admissions to the intensive care unit (ICU), operating theater, and other hospitals.

**Methods:** A retrospective chart review was made from May 1st, 2022 until October 31st, 2022. The data regarding the ABC codes was collected through a search in the patient medical records.

**Results:** During the study period, 138 codes for the ABC team occurred at Gødstrup Hospital compared to 68 codes for the ABC team at Regional Hospital West Jutland from December 2019 to May 2020 [3]. When activating the ABC code 4% of the patients had airway problems, 22% had breathing problems, 13% had circulatory problems, 17% had disability problems, 1% had exposure problems, 35% had more than one of the mentioned problems, 6% were stable at arrival of the ABC team, and 2% were false code (Figure). After ABCDE rescue 28% of the patients were transferred to the ICU, operating theater or another hospital compared to 35% at Regional Hospital West Jutland [3].

**Conclusions:** There was a notable increase in the number of ABC codes. The proportion of patients transferred to the ICU, operating theater, and other hospitals combined with the high proportion of patients with relevant ABCDE-problems demonstrate the need for the ABC team. The implemented ABC code is recommended to continue based on the mentioned results.

**References**

1. Semeraro F et al. Resuscitation. 2021;161:80–97
2. Maharaj R et al. Crit Care. 2015;19:254
3. Birkebaek S et al. ICMx. 2021;9 (Suppl 1):50

**Figure (abstract P004):**
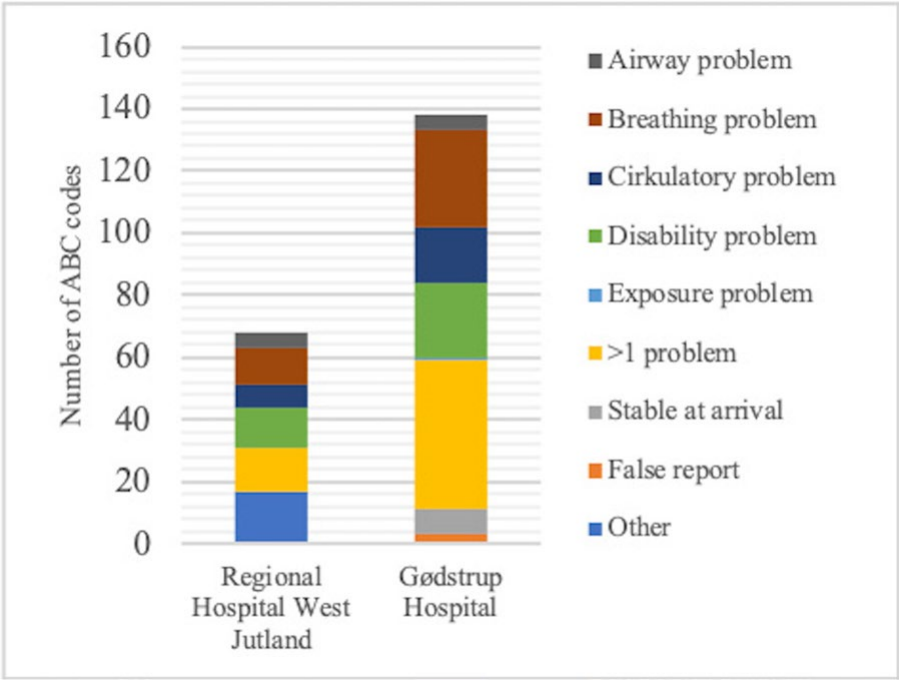
The number of ABC codes and indications for activating the ABC team at Gødstrup Hospital from May 2022 until October 2022 and at Regional Hospital West Jutland from December 2019 to May 2020.

## P005 Doctors’ and nurses’ perceptions on barriers and facilitators to implementing emergency medicine clinical pharmacy services in Singapore general hospital: a mixed-methods study

### QYG Mun^1^, KRR Kwan^1^, CP Lim^2^, JL Soong^2^

#### ^1^National University of Singapore, Pharmacy, Singapore, Singapore, ^2^Singapore General Hospital, Pharmacy, Singapore, Singapore

*Critical Care* 2024, **28(Suppl 1):** P005

**Introduction:** Emergency medicine (EM) clinical pharmacy services are increasingly common globally and have been shown to improve patient safety and care in the high-risk environment of the emergency department (ED). However, such services are not widely available in Singapore. This study assessed the current needs of ED doctors and nurses in Singapore General Hospital (SGH) and evaluated their perceptions towards EM clinical pharmacy services. Barriers and facilitators to the implementation of these services were further explored.

**Methods:** An anonymous quantitative survey was sent to all SGH ED doctors and nurses to assess their perceptions towards EM clinical pharmacy services. Qualitative data on barriers and facilitators were explored through open-ended questions in the survey and semi-structured interviews, and then analysed via thematic analysis using the Consolidated Framework of Implementation Research.

**Results:** A total of 205 survey responses were collected, and 7 interviews were conducted. Majority of respondents were receptive to having clinical pharmacists in the ED (81.7%) and perceived that clinical pharmacists improve medication safety and patient outcomes (77.5%). Top-ranked pharmacy services identified were drug information consultation, facilitating timely treatment of emergency conditions, and providing staff and patient education. Barriers and facilitators to implementation of the services are shown in the Figure. Pertinent barriers were cost, culture, compatibility, and manpower, while key facilitators included relative advantage of the services, tension for change within SGH ED, tailoring strategies, and engaging doctors and nurses during the implementation process.

**Conclusions:** ED doctors and nurses favourably perceived EM clinical pharmacy services. Strategies to implement the services should be tailored to address barriers and leverage facilitators identified for successful implementation within the local SGH ED context.

**Figure (abstract P005):**
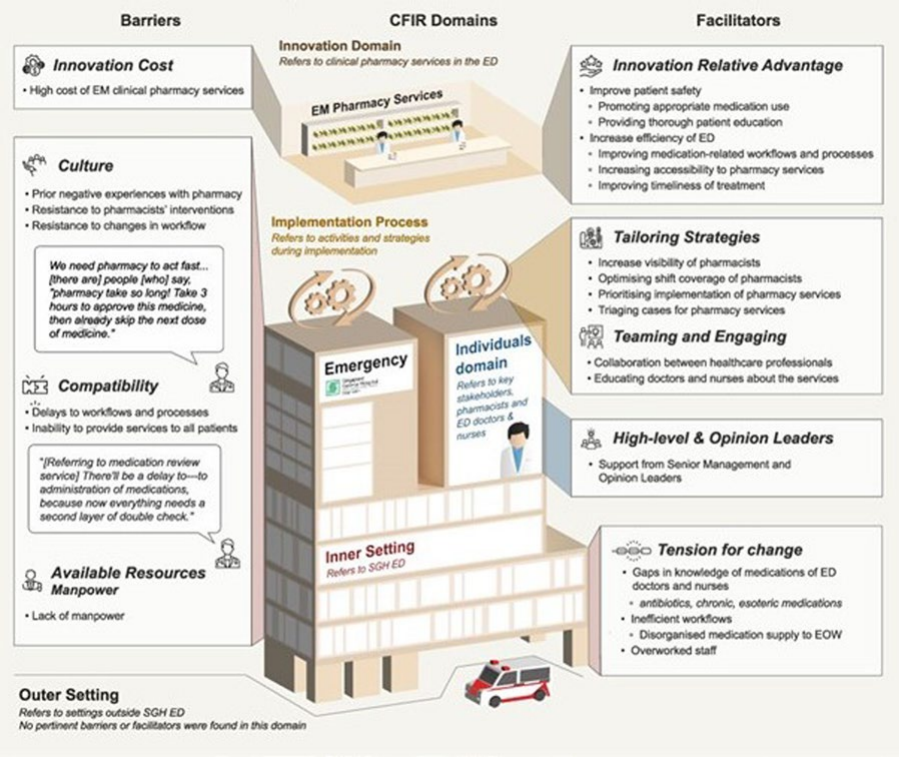
Barriers and facilitators to implementation of emergency medicine clinical pharmacy services.

## P006 Evaluating the MeMed BV® score in adult patients presenting with respiratory infections: a prospective multicenter observational trial (CRONUS study)

### I Nikolakakis^1^, E Tasouli^2^, S Gerakari^1^, S Sympardi^2^, P Thanasoulias^3^, E Polyzogopoulou^4^, J Parissis^4^, E Giamarellos-Bourboulis^5^, K Leventogiannis^5^

#### ^1^Tzaneion General Hospital, Emergency Department, Piraeus, Greece, ^2^Thriasio General Hospital, 1st Department of Internal Medicine, Eleusis, Greece, ^3^Hellenic Institute for the Study of Sepsis, Athens, Greece, ^4^National and Kapodistrian University of Athens, Emergency Department, Athens, Greece, ^5^National and Kapodistrian University of Athens, 4th Department of Internal Medicine, Medical School, Athens, Greece

*Critical Care* 2024, **28(Suppl 1):** P006

**Introduction:** MeMed BV® is a diagnostic assay which integrates the information from three blood biomarkers, namely TRAIL, IP-10, CRP, into a score (BV score) ranging between 0 and 100 that aids towards the differential diagnosis of viral versus bacterial infections in patients with acute infections. Preliminary results of the CRONUS study are presented analyzing the impact of the BV score on the decision to prescribe antibiotics.

**Methods:** CRONUS is an ongoing prospective study powered for 230 patients and running in three Emergency Departments (ED) in Greece. Enrolled patients are adults admitted at the ED with symptoms of respiratory tract infections up to seven days. On admission, 100 μL of serum is analyzed using the using MeMed BV test. Attending physicians are asked to provide their feedback on the need to prescribe antibiotics without being aware of the result of the BV score using a Likert scale questionnaire. Next, the physicians received the BV score and they were asked to repeat the questionnaire. The impact of the BV score on decision making is presented.

**Results:** Preliminary analysis included 200 patients. Overall, the BV score was lower in patients for whom no antibiotics were prescribed (n = 95; median = 8 [2–31]) compared to patients receiving antibiotics (n = 90; median = 96 [2–31]). In patients where the decision was changed in favor of giving antibiotics, the BV score was higher (n = 12; median = 72 [49–97]) compared to patients not given antibiotics (n = 95; median = 8 [2–31]) (Figure). In patients where the decision was changed in favor of not giving antibiotics, the BV score was lower (n = 3; median = 4 [2–31]) compared to patients given antibiotics (n = 90; median = 96 [2–31]). In the latter subgroups, unnecessary antibiotic prescribing was reduced by 3.23%.

**Conclusions:** These interim results suggest that the MeMed BV test effectively differentiates viral from bacterial infections in patients with acute respiratory symptoms, aiding antibiotic stewardship.

**Figure (abstract P0006):**
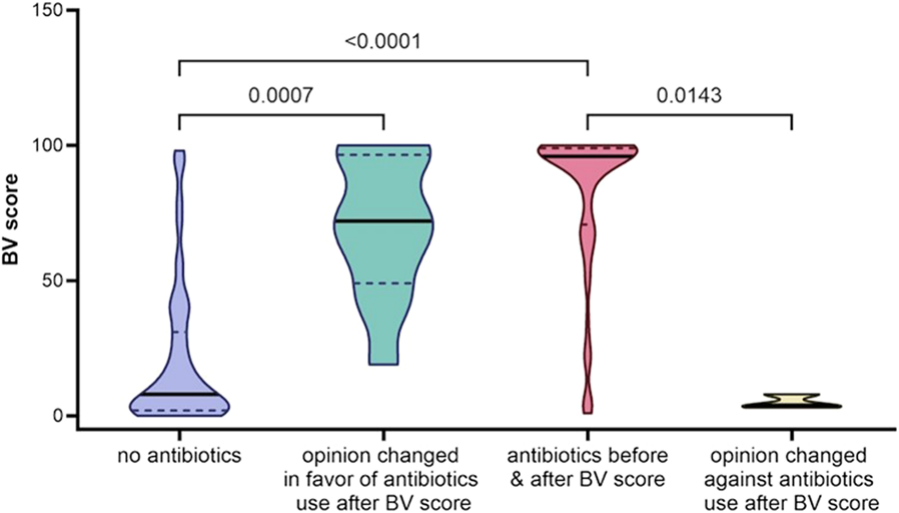
BV score impact on decision of antibiotic administration.

## P007 Retrospective evaluation of an automated early warning score in a Dutch hospital

### THGF Bakkes^1^, AJR de Bie^2^, JA van der Stam^3^, U Kaymak^4^, M Mischi^4^, RA Bouwman^5^, S Turco^4^

#### ^1^Eindhoven University of Technology, Electrical Engineering, Eindhoven, Netherlands, ^2^Catharina Hospital, Intensive Care, Eindhoven, Netherlands, ^3^Eindhoven University of Technology, Biomedical Engineering, Eindhoven, Netherlands, ^4^Eindhoven University of Technology, Eindhoven, Netherlands, ^5^Catharina Hospital, Eindhoven, Netherlands

*Critical Care* 2024, **28(Suppl 1):** P007

**Introduction:** Timely identification of patients at risk is crucial to allow for prompt intervention and improve patient outcomes while reducing irrelevant alarms. Previous research has shown that the advanced alert monitor (AAM), an automated early warning score (EWS) based on electronic medical records (EMR) data, improved patient outcomes in an American multicenter trial [1, 2]. The objective of this study is to determine if the AAM can achieve the same performance outside its original population in a Dutch hospital.

**Methods:** Data was extracted from the EMR for all patients admitted to the Catharina Hospital (Eindhoven, The Netherlands) from December 2018 till January 2023. The data was preprocessed similar to the original study [1]. The data was divided into hospital admissions episodes with and without adverse events. Events were defined based on IC admissions, mortality, and acute surgeries. The AAM was evaluated and compared to the national early warning score (NEWS) as a baseline. Additionally, the AAM model was retrained and evaluated via fivefold cross-validation.

**Results:** The resulting dataset consisted of data from 88,561 valid episodes, with 1930 episodes ending with an adverse event. The AAM outperformed the NEWS with an episode-based area under the receiver operating characteristics curve (AUROC) of 78.5% versus 72.6%. The original performance obtained in [1] was 82%. Retraining the AAM resulted in an average AUROC over the 5-folds of 80.5% (Figure). Retraining the model also increased the sensitivity of the alarms from 31.8 to 49.9%.

**Conclusions:** The AAM showed improved performance over conventional EWS when evaluated on an independent dataset outside of its original patient population. Retraining the model resulted in improved performance underlining the importance of local optimization over a one-size-fits-all solution.

**References**

1. Kipnis P et al. J Biomed Inform 2016;64:10–19
2. Martines VA et al. Jt Comm J Qual Patient Saf 2022;48:370–375

**Figure (abstract P007):**
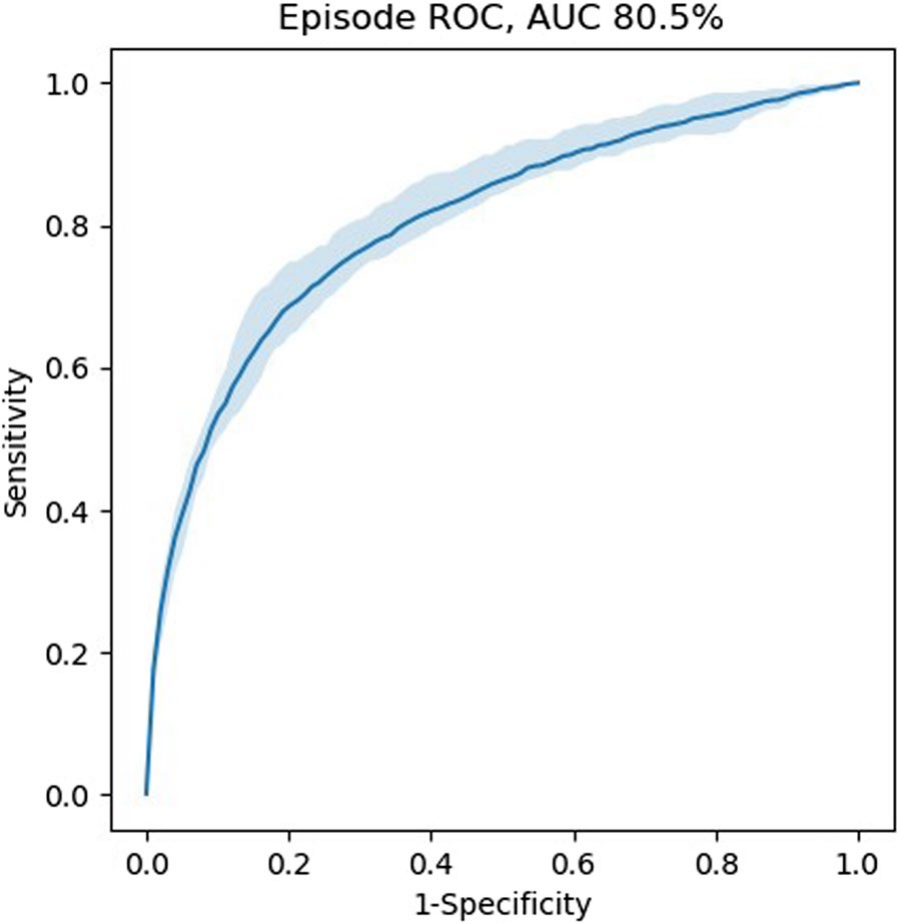
Receiver operating characteristics curve of retrained AAM model, with confidence interval of 5-folds.

## P008 The fiscal impact of a rapid sepsis diagnostic in the emergency department (ED)

### C D´Antonio^1^, HR O´Neal, Jr.^1^, AK Hollis^2^, K Richard^2^, L Booty^2^, T Jagneaux^1^, M Laperouse^3^, L Teague^2^, C Pierce^2^, CB Thomas^4^

#### ^1^LSUHSC - Baton Rouge, Pulmonary and Critical Care, Baton Rouge, USA, ^2^Our Lady of the Lake Regional Medical Center, Quality & Safety, Baton Rouge, USA, ^3^Our Lady of the Lake Regional Medical Center, Emergency Medicine, Baton Rouge, USA, ^4^Franciscan Ministries of Our Lady Health System, Quality & Safety; Pulmonary & Critical Care, LSUHSC, Baton Rouge, USA

*Critical Care* 2024, **28(Suppl 1):** P008

**Introduction:** Sepsis is the most costly condition in US hospitals [1]. The majority of cases present to EDs [2], where early recognition is difficult [3]. A rapid diagnostic may improve the efficiency of sepsis care. We recently implemented a novel sepsis diagnostic (IntelliSep, Cytovale, SanFrancisco, USA) into the sepsis process of a large US academic medical center. Using a pre-post analysis, we studied the impact of this implementation on the efficiency of sepsis care.

**Methods:** In the pre-implementation (control) cohort, IntelliSep was performed but not released to clinicians in Jun-2023. For the post-implementation (intervention) cohort, IntelliSep was performed and released as standard care from Aug through Sept-2023. For both cohorts, the IntelliSep ordering process was a nurse-driven protocol (Figure). Financial and utilization data was obtained via billing records according to hospital chargemaster. Descriptive statistics were performed on clinical and economic outcomes for the index evaluation. Fiscal comparison was further separated between observation and inpatient status.

**Results:** The study included 196 control and 413 intervention patients. Compared to controls, the total cost of care was reduced by $1429 per patient in the intervention cohort. A cost reduction of $1930 per patient with an associated 1.28 reduction in average length of stay (LOS) was observed for inpatients. In patients with an ICU encounter, a cost reduction of $3624 per patient with an associated average LOS reduction of 2.42 days was demonstrated. In patients admitted to observation status, a cost reduction of $243 was observed.

**Conclusions:** IntelliSep, when integrated into an early sepsis diagnosis protocol in a large US academic medical center, results in reduction in overall cost of care and LOS compared to a similar process without the test.

**References**

1. Liang L et al. HCUP Statistical Brief #261, Jul-2020
2. Page DB et al. Crit Care Med 2015;43:1945–51
3. Newman-Toker et al. AHRQ Comp Eff Rev (US) 2022; report no. 22(23)-EHC043.

**Figure (abstract P008):**
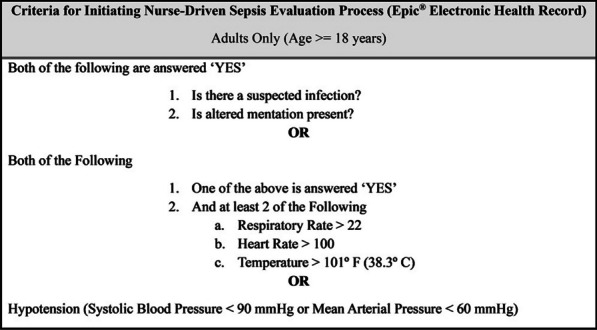
Criteria and logic for a nurse-driven sepsis evaluation process, implemented at triage. If criteria are met, the triage nurse can order initial laboratory evaluation for sepsis, including comprehensive metabolic profile, complete blood count, lactic acid, and IntelliSep.

## P009 Sepsis burden and organ failure among patients in the emergency department: a Danish population-based cohort study

### C Schade Skov^1^, M Brabrand^1^, C Backer Mogensen^2^, H Skøjt-Arkil^2^, F Schønning Rosenvinge^3^, I Somuncu Johansen^4^, A Touborg Lassen^1^

#### ^1^Odense University Hospital, Department of Emergency Medicine, Odense, Denmark, ^2^Hospital Sønderjylland, Department of Emergency Medicine, Aabenraa, Denmark, ^3^Odense University Hospital, Department of Clinical Microbiology, Odense, Denmark, ^4^Odense University Hospital, Department of Infectious Diseases, Odense, Denmark

*Critical Care* 2024, **28(Suppl 1):** P009

**Introduction:** In this study the aim was to estimate the proportion and incidence of sepsis among adult patients with a contact an emergency department in the Region of Southern Denmark (adult population N = 968,761), based on clinical findings and laboratory measures. Furthermore, type and number of organ failures were estimated.

**Methods:** A register-based cohort study including all acute patients with a contact to an emergency department (ED) from 1 January 2016 to 19 March 2018. Sepsis patients were identified based on fulfilling following criteria: (1) blood culture performed within 48 h of arrival, (2) antibiotic(s) administered within 48 h of arrival, and (3) 1 organ failure(s) based on a SOFA-score modified for the ED. An increase of 2 point for an organ at the modified SOFA-score was considered equivalent to one organ failure. We calculated 7-, 30-, and 90-days mortality rates. Type and number of organ failure(s) were calculated in relation to ICU-admission and 30-days mortality.

**Results:** We identified 443,953 patients with an acute contact to an ED in the RSD and among those 13,081 had one or more episodes of sepsis (3.0%, 95% CI 2.9–3.0), while 10,597 had incident sepsis; 53.7% were male, and median age was 75 (IQR: 65–84). The incidence rate of sepsis in the RSD was 494/100,000 person years (95% CI 484–504). Overall 7-, 30-, and 90-days mortality was 8.2%, 17.1%, and 23.3%, respectively. Some 1235 patients were admitted to the intensive care unit (ICU) with 47 h median ICU-length of stay (IQR: 21–111). The overall 30-days mortality for patients with an ICU-admission was 30.0%, and 15.4% for patients without ICU-admission. A description of organ failures is provided in the Figure.

**Conclusions:** Based on clinical findings and laboratory measures, the incidence rate of patients with sepsis admitted through the ED was 494/100,000 person-years. Most sepsis patients had single organ failure, but 2 or more organ failures were present in 14%.

**Figure (abstract P009):**
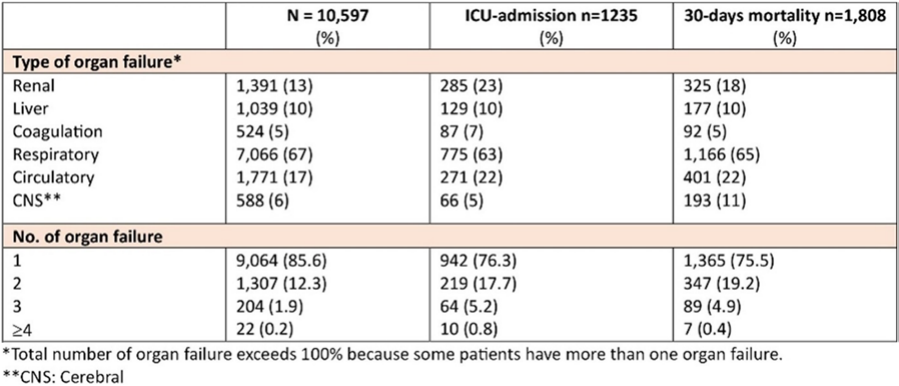
Description of organ failures.

## P010 Differences between cardiac arrest and heart attack: public awareness and willingness to provide CPR

### YCM Chia^1^, WX Lim^2^

#### ^1^Tan Tock Seng Hospital, Emergency Department, Singapore, Singapore, ^2^Singapore University of Social Sciences, School of Science and Technology, Singapore, Singapore

*Critical Care* 2024, **28(Suppl 1):** P010

**Introduction:** This study aims to assess public’s knowledge, awareness and attitudes towards cardiac arrest and heart attack, and the willingness and barriers in providing CPR.

**Methods:** First-round survey was carried out on symptoms, signs and treatment between cardiac arrest and heart attack, followed by willingness and barriers in providing bystanders CPR. Poster (Figure) was drawn to illustrate differences between both conditions, followed by second-round survey to access level of understanding.

**Results:** There were 101 participants in first survey. 69% had post diploma education. 42% were not aware of differences between cardiac arrest and heart attack. Participants that said were aware of differences, only 63% knew cardiac arrest had no pulse and breathing. 51% mistakenly expressed shortness of breath as a symptom. For heart attack, 51% mistook speech difficulty and 12% mistook no pulse as symptoms. 18% would perform CPR and 14% would retrieve AED for someone with chest pain. 38% stated lack of confidence as barrier to performing CPR, and 37% stated fear of prosecution for unsuccessful CPR. 50% expressed making CPR an educational requirement for students, and 16% stated more campaigns to raise awareness could increase bystander CPR. There were 62 participants in the second survey on poster feedback. 42% did not know there was difference between cardiac arrest and heart attack. 70% understood the poster content, while 10% stated poster should be simplified more. While 97% stated the poster could help raise awareness between both conditions and importance of CPR, only 79% understood the differences after reading the poster.

**Conclusions:** Significant proportion of public confuses cardiac arrest and heart attack, despite from high education background. Those who said knew the differences, majority still misunderstood the signs and symptoms. Lack of confidence and fear of prosecution were top barriers to performing CPR. Well-designed poster using simple layman language is useful to educate and explain the differences.

**Figure (abstract P010):**
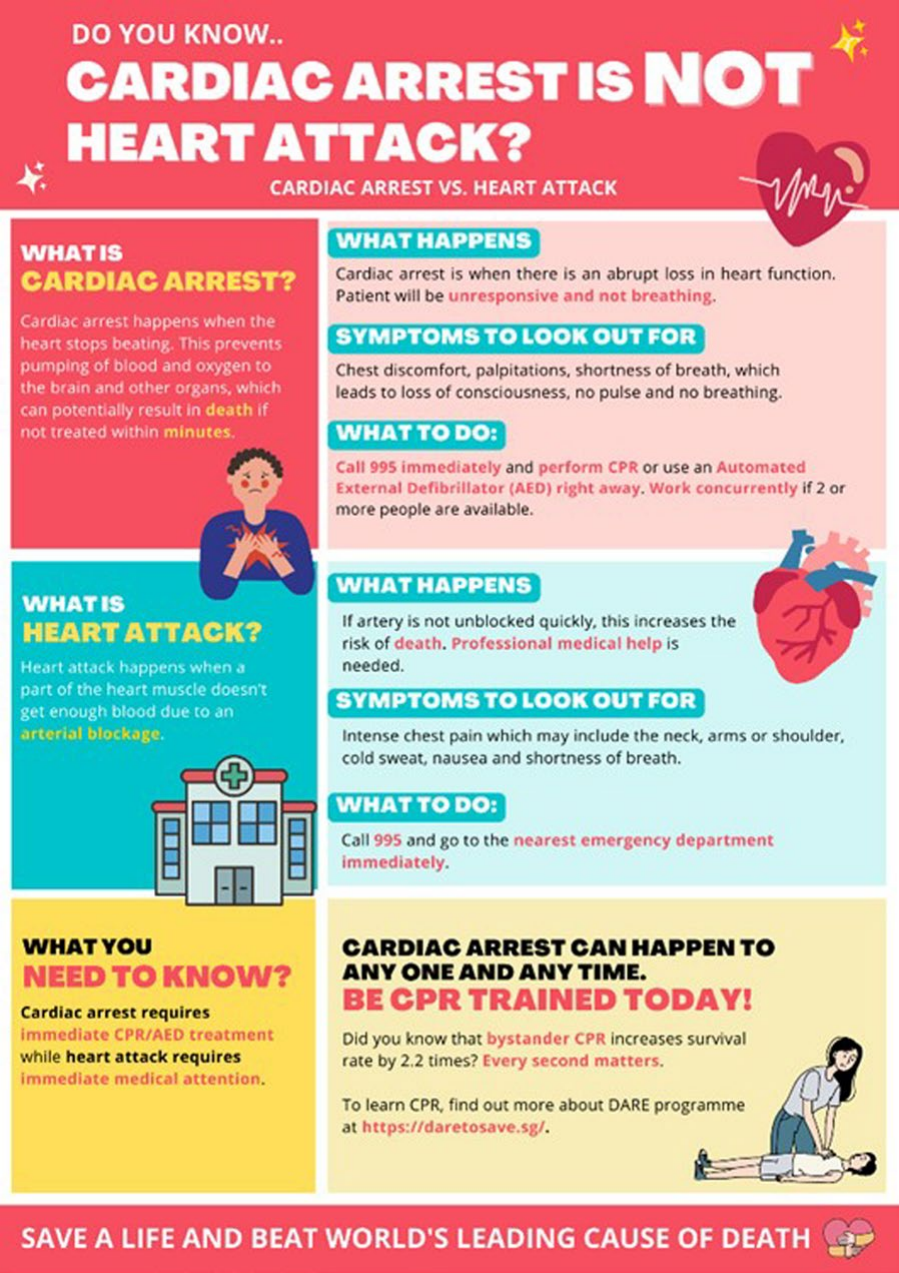
Poster to illustrate differences between cardiac arrest and heart attack.

## P011 Interaction effects between parental history of heart disease and own heart disease on incidence of out-of-hospital cardiac arrest: a case–control study

### H Ryu, J Lee, C Park

#### Chonnam National University, Emergency Department, Gwangju, South Korea

*Critical Care* 2024, **28(Suppl 1):** P011

**Introduction:** There were no studies on the interaction effect between parental history of heart disease and own heart disease for out-of-hospital cardiac arrest (OHCA) incidence. The purpose of our case–control study is to determine whether history of parental heart disease was associated with OHCA incidence, and the effect of parental history is affected by the own heart disease history.

**Methods:** We conducted a multicenter prospective case–control study in 17 University hospitals in Korea from September 2017 to December 2020. Cases were EMS-treated OHCA patients age 19–79 with presumed cardiac etiology. Community-based controls were recruited at a 1:1 ratio after matching age, sex, and urbanization level of residence. Multivariable conditional logistic regression analysis were conducted to estimate the risk of parental heart disease including coronary artery disease (CAD), non-CAD, and sudden cardiac death (SCD) on OHCA. Interaction analysis between parental heart disease and own heart disease were also performed.

**Results:** A total 948 OHCA cases and 948 community-based controls were analyzed. Compared to the no parental SCD group, the risks of OHCA increased in the parental SCD group [aOR (95% CI): 2.67 (1.65–4.30)]. Parental history of SCD had interaction effects with own non-coronary heart disease for OHCA incidence: 2.60 (1.45–4.66) for no heart disease and 2.08 (0.43–10.07) for heart disease (*p* for interaction < 0.05).

**Conclusions:** In our study, parental history of SCD increased the incidence of OHCA and the effect of parental history for SCD was maintained in patients without own heart disease.

## P012 Intra-arrest transport and good neurological recovery among out-of-hospital cardiac arrest with refractory ventricular fibrillation: a nationwide observational study

### J Lee, H Ryu, J Lee, C Park

#### Chonnam National University, Emergency Department, Gwangju, South Korea

*Critical Care* 2024, **28(Suppl 1):** P012

**Introduction:** This study analyzes the impact of on-scene resuscitation and intra-arrest transport on survival outcomes for patients with recurrent ventricular fibrillation (rVF) following out-of-hospital cardiac arrest (OHCA). While many essential treatments for rVF can only be administered at the hospital level, especially in settings with limited Advanced Life Support (ALS) resources, the deterioration in CPR quality during transport complicates the decision of whether early transport to a hospital improves patient outcomes.

**Methods:** Using a nationwide OHCA registry, adult medical OHCA patients with rVF who underwent CPR between 2015 and 2021 were included. The primary outcome was survival outcome at discharge. Time-dependent propensity score matching was used to assess the association between intra-arrest transport and survival outcomes. Risk ratio (RRs) and 95% confidence intervals (CIs) were estimated, and stratified analysis by the timing and ECMO-capable center visit were also performed.

**Results:** Of the 9108 patients, they were divided into on-scene resuscitation and Intra-arrest transport groups of 1365 each by 1:1 time-dependent propensity score matching. In the matched cohort, On-scene resuscitation was more effective in improving neurological outcome (18.5% in On-scene resuscitation, 13.7% in Intra-arrest transport; RR [95% CI] 0.82 [0.72–0.94]) (Table). The stratified analyses according to the timing of matching shows better outcomes with on-scene resuscitation across different time intervals, except for the initial 0–2 min. Positive outcomes associated with on-scene resuscitation were maintained regardless of the ECMO capabilities of the center (25.7% in On-scene resuscitation, 20.0% in Intra-arrest transport; RR [95% CI] 0.83 [0.68–1.01]).

**Conclusions:** When rVF is initially identified by EMS, providing on-scene resuscitation for up to 8 min results in better patient outcomes compared to Intra-arrest transport.

**Table (abstract P012):** Survival outcomes by intra-arrest transport in the matched cohort

	Outcome	Risk ratio (95% CI)	
	n/N (%)	Unadjusted	Adjusted
*Good neurological recovery*			
On-scene resuscitation	252/1365 (18.5)	1	1
Intra-arrest transport	187/1365 (13.7)	0.74 (0.63–0.87)	0.82 (0.72–0.94)
*Survival to discharge*			
On-scene resuscitation	331/1365 (24.2)	1	1
Intra-arrest transport	257/1365 (18.8)	0.78 (0.69–0.87)	0.85 (0.77–0.94)

## P013 Cooling the storm: targeted temperature management (TTM) and improved survival outcomes following non-traumatic out-of-hospital cardiac arrests (OHCAs)

### AHJ Lim, MYC Chia

#### Tan Tock Seng Hospital, Emergency Medicine, Singapore, Singapore

*Critical Care* 2024, **28(Suppl 1):** P013

**Introduction:** OHCAs is a pressing global health concern with unfavourable outcomes. TTM decreases metabolic demands and is pivotal in management of post-cardiac arrest syndrome. This study investigates effect of TTM at the Emergency Department(ED) on survival and neurological outcomes of OHCAs.

**Methods:** This is a single centre retrospective cohort study with data from a local cardiac arrest registry across the period of 2010–2020. Out of the 1312 patients, 157 underwent TTM at 34 degrees Celsius, followed by a cooling protocol in the intensive care unit. Patients were followed up for 30 days, during which demographic variations and outcomes were analyzed between TTM and normothermia groups.

**Results:** Age and cardiac history were comparable between both groups (Table). There was a significant difference in odds of 30 day mortality between TTM and normothermia groups (OR 0.28, [95% CI 0.17–0.46], *p* < 0.001). Patients with a shockable rhythm in the TTM group had a lower 30-day mortality of 70.7%, compared to those with a non-shockable rhythm (X^2^ = 9.08, *p* = 0.003). At 30 days, 50.5% who survived had a Cerebral Performance Category (CPC)^1^ Score of 1–2 (Glasgow–Pittsburgh CPC 1–2 defined as favourable neurologic outcome); a greater proportion of favourable outcomes in the TTM group. Odds of survival with unfavourable CPC scores did not differ significantly between both groups (OR 0.61 [95% CI 0.24–1.51], *p* = 0.28). Among patients with shockable rhythm and TTM, survival rate was 100% when pre-hospital bystander CPR and AED were used, whereas it was 17.5% with only bystander CPR (Fisher exact (F) = 0.042, *p* < 0.05). 89.1% of bystander CPR was performed by laymen and survival rates did not differ between group with CPR performed by healthcare personnel (F = 0.010, *p* > 0.05).

**Conclusions:** TTM in the ED improves survival outcomes in OHCAs, especially within groups with shockable rhythms. Bystander CPR and AED play a crucial role in the chain of survival, crowdsourcing community first responders by a mobile app is valuable.

**Table (abstract P013):** Demographics and survival outcomes in targeted temperature management and normothermia groups

	Targeted Temperature Management (N = 157)	Normothermia (N = 1155)	*p* value
Age, mean (SD)	61.0 (15.51)	68.0 (15.19)	0.000
Gender–Male	119 (75.8%)	746 (64.6%)	0.005
History of cardiac disease	56 (35.7%)	413 (35.8%)	0.983
Shockable rhythm on first arrest	58 (36.9%)	196 (17.0%)	< 0.001
Survived to hospital admission	150 (95.5%)	596 (51.6%)	< 0.001
Mortality at 30 days after admission	123 (78.3%)	532 (89.3%)	0.015
CPC Score 1–2 at 30 days	16 (59.2%)	30 (46.9%)	0.280

## P014 Determinants of vascular leakage after cardiac arrest

### A Rutault^1^, E Guérin^2^, I Marangon^1^, C Desnos^1^, PL Tharaux^3^, A Combes^4^, S Germain^1^, N Bréchot^2^

#### ^1^INSERM U1050-Collège de France, Paris, France, ^2^European Georges Pompidou Hospital, APHP, Medical ICU, Paris, France, ^3^INSERM U970, Paris Cardiovascular Research Center, Paris, France, ^4^La Pitié-Salpêtrière Hospital, APHP, Medical ICU, Paris, France

*Critical Care* 2024, **28(Suppl 1):** P014

**Introduction:** Vascular leakage is a major feature of post-cardiac arrest syndrome. Here we performed RNAseq analysis of circulating monocytes and identified 38 genes differentially expressed between patients with and without massive vascular leakage after cardiac arrest. Among them, a gene coding for a protein P (ongoing patent protection) had a 56-time higher expression in patients with massive leakage. In accordance, plasma levels of P-protein strongly associated with the level of fluid requirements in a validation cohort of 52 post-cardiac arrest patients. The objective of the study was to characterize P-protein effects on post-cardiac arrest vascular leakage.

**Methods:** Effects of P-protein was analyzed in a model of resuscitated cardiac arrest in mice, with a no-flow and a low-flow time of 8 min each.

**Results:** In vivo, we confirmed an important vascular leakage in all organs after the return of spontaneous circulation (ROSC) in WT mice and an induction of circulating P-protein levels at one-hour post-ROSC. Intra-venous injection of recombinant mouse (rm)P-protein at the time of resuscitation significantly reduced vascular leakage in all the organs, quantified by extravasation of fluorescent dextran dyes. Consistent with less myocardial edema, left ventricular ejection fraction was also significantly improved in rmP-protein-injected mice. Finally, survival significantly increased in mice injected with the protein versus controls (Figure).

**Conclusions:** These results demonstrate that P-protein, identified from human samples, is a key regulator of post-cardiac arrest vascular leakage, and a promising new treatment during this condition. Further experiments are ongoing to characterize its mechanisms of action, as well as its optimal dosing regimen in pre-clinical settings.

**Figure (abstract P014):**
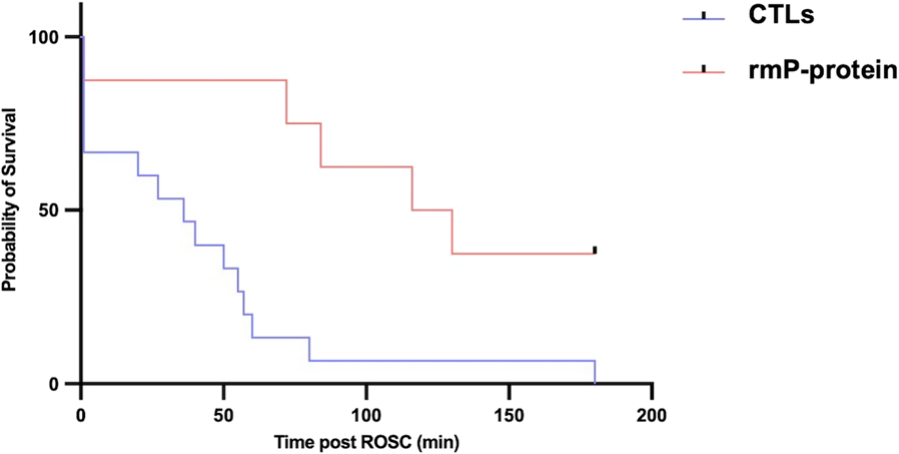
Survival after cardiac arrest of wild-type mice, injected (rmP-protein, n = 8) or not (CTLs, n = 15) with recombinant mouse P-protein at the time of resuscitation.

## P015 The detrimental effects of intestinal injury mediated by inflammation are limited in cardiac arrest patients: a prospective cohort study

### B Farbu^1^, S Lydersen^2^, RM Mohus^1^, T Ueland^3^, P Klepstad^1^, H Langeland^1^

#### ^1^St. Olav’s University Hospital, Department of Anaesthesiology and Intensive Care Medicine, Trondheim, Norway, ^2^Norwegian University of Science and Technology (NTNU), Regional Centre for Child and Youth Mental Health and Child Welfare, Department of Mental Health, Faculty of Medicine and Health Sciences, Trondheim, Norway, ^3^University of Oslo, Institute of Clinical Medicine, Oslo, Norway

*Critical Care* 2024, **28(Suppl 1):** P015

**Introduction:** The mechanisms leading from whole-body ischaemia to multiple organ dysfunction and death are unclear. In this post-hoc study we explored the potential effects of intestinal injury mediated through the inflammatory response on organ dysfunction and mortality after out-of-hospital cardiac arrest (OHCA).

**Methods:** We measured plasma intestinal fatty acid binding protein (IFABP), a biomarker for intestinal injury, and inflammatory biomarkers in 50 patients at admission to intensive care unit after OHCA. We stratified patients on median IFABP and compared biomarker levels between the “low” and “high” IFABP group. By causal mediation analyses we assessed effects of IFABP through the most important inflammatory biomarkers on day two circulatory variables, Sequential Organ Failure Assessment (SOFA)-score, and 30-day mortality. ClinicalTrials.gov: NCT02648061.

**Results:** Inflammatory biomarkers were increased in the high IFABP group. In the mediation analyses, the natural indirect effect of IFABP through interleukin (IL)-6 was 0.9 (95% CI 0.2–1.5) SOFA points, 75th percentile of IFABP, compared to 25th percentile, and the natural direct effect of IFABP was 2.3 (95% CI 0.7–3.9) SOFA points. Effects on circulatory variables were uncertain. Patients on 75th percentile of IFABP, compared to 25th percentile, had a 53% (95% CI 33–74; *p* < 0.001) higher chance of dying, where 13 (95% CI 3–23; *p* = 0.01) percentage points were mediated through a natural indirect effect of IL-6, and 41 (95% CI 19–62; *p* < 0.001) percentage points were due to a natural direct effect (Figure). Effects through terminal C5b-9 complement complex were not statistically significant.

**Conclusions:** Effects of IFABP mediated through inflammation on organ dysfunction and mortality were limited. Small, but significant, effects through IL-6 were noted. This suggests that intestinal injury is not a “driver” of critical illness through an inflammatory response after OHCA.

**Figure (abstract P015):**
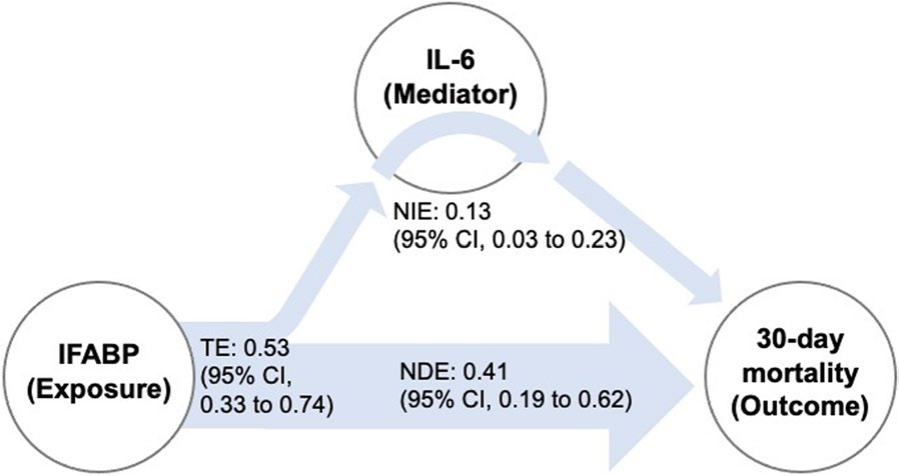
Effects of IFABP and IL-6 on mortality in mediation analysis. The reported effects are risk differences (95% CI) between the 75th percentile of IFABP, compared to 25th percentile. TE is the total effect of IFABP on 30-day mortality, which is the sum of NDE, the natural direct effect of IFABP, and NIE, the natural indirect effect through IL-6. IFABP: Intestinal fatty acid binding protein; IL: interleukin; CI: confidence interval.

## P016 Health related quality of life after refractory cardiac arrest treated with conventional or extracorporeal CPR

### AF van de Koolwijk^1^, MM Suverein^1^, TSR Delnoij^2^, B Winkens^3^, JG Maessen^4^, R Lorusso^4^, MCG van de Poll^1^, INCEPTION investigators^5^

#### ^1^Maastricht University Medical Centre, Department of Intensive Care Medicine, Maastricht, Netherlands, ^2^Maastricht University Medical Centre, Department of Intensive Care Medicine & Department of Cardiology, Maastricht, Netherlands, ^3^Maastricht University, Department of Methodology & Statistics and Care and Public Health Research Institute, Maastricht, Netherlands, ^4^Maastricht University Medical Centre, Department of Cardiothoracic Surgery, Maastricht, Netherlands, ^5^Maastricht University Medical Centre, Maastricht, Netherlands

*Critical Care* 2024, **28(Suppl 1):** P016

**Introduction:** Extracorporeal cardiopulmonary resuscitation (ECPR) is emerging as an intervention to improve outcome after out-of-hospital cardiac arrest (OHCA). The importance of health-related quality of life (HRQoL) in OHCA survivors is increasingly recognized. We aimed to determine HRQoL and functional outcome during 1-year after refractory OHCA, treated with ECPR or conventional CPR (CCPR) and to assess factors associated with functional outcome in these patients.

**Methods:** This is a post-hoc analysis of a multicenter randomized clinical trial, that studied the effectiveness of ECPR in patients with refractory OHCA. During 1 year after OHCA, data on HRQoL was collected using the 5Q-5D-5L questionnaire. Based on the health utility index at the last follow-up, we categorized functional outcome as good or poor. Poor functional outcome was defined as a health utility index > 1 standard deviation below the age adjusted norm. Data on HRQoL were analyzed using mixed linear models. Univariable analyses were performed to assess factors potentially associated with functional outcome.

**Results:** We enrolled 134 patients, 55 received ECPR and 79 CCPR. Hospital survival was 20% (27 patients), 5 (9%) patients survived after ECPR and 22 (28%) after CCPR. HRQoL data were available for 25 patients. One year after OHCA, estimated mean health utility index was 0.84 ± 0.12 versus 0.71 ± 0.05 in ECPR versus CCPR survivors (*p* = 0.31). Overall estimated mean health utility index was 0.73 ± 0.05. All ECPR survivors versus 60% of the CCPR survivors had a good functional outcome (*p* = 0.14). We identified no specific factors that were significantly associated with functional outcome (Table).

**Conclusions:** One year after refractory OHCA, 68% of the survivors have a good functional outcome. We found no statistically significant difference in HRQoL and functional outcome in survivors of refractory OHCA treated with ECPR or CCPR and no factors that were associated with functional outcome.

**Table (abstract P016):** Results

Parameter	Overall (n = 25)	Good (n = 17)	Poor (n = 8)	*p* value
Age—years	57 [43–63]	57 [44–63]	55 [38–64]	0.79
Duration of the arrest—min	46 [23–57]	35 [23–65]	49 [30–54]	0.68
pH at arrival at ED*	7.02 [6.87–7.14]	7.02 [6.87–7.17]	7.04 [6.88–7.14]	1.00
Lactate at arrival at ED**	11.3 [7.0–14.5]	11.3 [7.5–15.2]	11.3 [6.1–14.7]	0.89
Treatment (ECPR/CCPR)	5(20)/20(80)	5(100)/12(60)	0(0)/8 (40)	0.23
Health utility index	0.73 ± 0.05	0.87 ± 0.05	0.44 ± 0.06	< 0.01
Cerebral performance category*** (1/2/3)	20(87)/2(9)/1(4)	14(70)/1(50)/0(0)	6(30)/1(50)/1(100)	0.62

Data are presented as median [IQR] compared using Mann Whitney U test. Health Utility Index presented as estimated mean with standard error. Categorical data are presented as no. (%), compared using Fisher’s exact test. ED = emergency department, CCPR= conventional cardiopulmonary resuscitation, ECPR: extracorporeal cardiopulmonary resuscitation *Data on 24 patients, in kPa, **Data of 20 patients, ***Data of 23 patients

##  P017 White blood cell count as an inflammatory marker after cardiac arrest: an observational database study

### AMJ Seppä^1^, MB Skrifvars^2^, PT Pekkarinen^1^

#### ^1^University of Helsinki and Helsinki University Hospital, Division of Intensive Care, Department of Anaesthesiology and Intensive Care, Helsinki, Finland, ^2^University of Helsinki and Helsinki University Hospital, Department of Emergency Care and Services, Helsinki, Finland

*Critical Care* 2024, **28(Suppl 1):** P017

**Introduction:** Ischaemia–reperfusion injury often triggers an inflammatory response that complicates the post-resuscitation care of cardiac arrest (CA) patients. Elevated levels of numerous inflammatory biomarkers are seen in circulation after CA [1]. We assessed the association between white blood cell count (WBC) and 1-year mortality in a large population of CA patients.

**Methods:** We retrospectively collected CA patients treated in the Finnish university hospital ICUs from a nationwide multicentre registry. We created a LOESS curve to assess the impact of early WBC on predicted 1-year mortality. Multivariable logistic regression models were used to assess independent association between early WBC and 1-year mortality. In patients with available data on ROSC delay we also conducted a nested cohort analysis in which linear regression was performed to test the association between ROSC delay and early WBC.

**Results:** 8047 CA patients were included. Mortality was lowest in the second quintile of WBC (Figure). The LOESS curved was U-shaped with lowest predicted mortality at 8 E9/l WBC. In patients with high WBC (n = 6561), higher WBC was independently associated with increased 1-year mortality (OR: 1.03, 95% CI 1.02–1.04, *p* < 0.001). In patients with low WBC (n = 1486), lower WBC was independently associated with increased mortality (OR: 0.90, 95% CI 0.84–0.95, *p* < 0.001). In a nested cohort analysis longer ROSC delay was associated with higher WBC in patients with a shockable rhythm (β = 0.10, R^2^ = 0.04, *p* < 0.001).

**Conclusions:** Early WBC was independently associated with 1-year mortality in CA patients. This association was U-shaped with both high and abnormally low values of WBC being associated with increased risk of adverse long-term outcomes. We hypothesize that low WBC reflects impaired immune response as high WBC reflects more severe inflammation.

**Reference**

1. Seppä AMJ et al. Acta Anaesthesiol Scand. 2023;67:1273–1287

**Figure (abstract P017):**
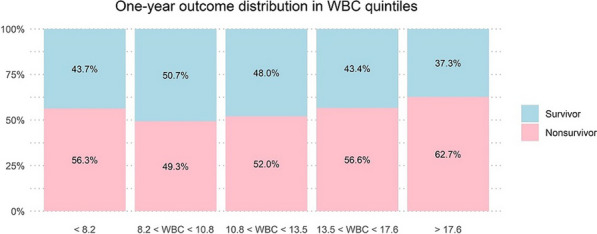
One-year mortality rates in WBC quintiles. The unit for WBC values is E9/l. Differences in mortality between quintiles were significant (*p *< 0.001)

## P018 The association of gender with cardiopulmonary resuscitation performance, knowledge, and attitudes amongst laypersons in Singapore

### S See^1^, AE White^2^, PT Naing Win^2^, NA Jalil^2^, SF Chong^2^, ME Ong^3^

#### ^1^Lee Kong Chian School of Medicine, Nanyang Technological University, Singapore, Singapore, ^2^Unit for Prehospital Emergency Care, Singapore, Singapore, ^3^Department of Emergency Medicine, Singapore General Hospital, Singapore, Singapore

*Critical Care* 2024, **28(Suppl 1):** P018

**Introduction:** Clinical research has found that males tend to achieve better CPR performance, particularly in compression depth. However, limited research has been conducted in the Asian context and few papers delve in-depth into the effect of gender on additional factors, such as CPR knowledge and attitudes. The objective of this paper was to uncover the association of gender with CPR performance as well as the knowledge and attitudes of laypersons towards CPR following CPR training in Singapore.

**Methods:** We evaluated the CPR performance of 983 participants by analysing the metrics of compression depth, rate and flow fraction as measured by the CPRCard™. The criteria for adequate CPR were defined according to the Singapore Resuscitation and First Aid Council guidelines in 2021. We assessed participants’ knowledge and attitudes via pre-training and post-training surveys. We also gathered feedback on the CPRCard™ via a survey employing a Likert scale.

**Results:** Male participants attained a statistically significantly higher proportion of compressions meeting the requirements of adequate rate (81.5% vs. 74.2%; *p* < 0.001) and depth (81.0% vs. 76.7%; *p* = 0.001). Prior to training, there was a statistically significant difference in the total knowledge quiz scores between genders, favouring males (*p* < 0.001); however, this gender gap became statistically insignificant after training (*p* = 0.454). The training had a positive impact on both male and female attitudes towards CPR, with a more pronounced effect observed in females. Overall, feedback on the CPRcard™ was favourable, with increased confidence and perceived ease of use, particularly in the male population.

**Conclusions:** The male gender was associated with better CPR compression depth, rate, and flow fraction. CPR training played a crucial role in bridging the knowledge gap between genders and fostered increased confidence among participants. Emerging feedback devices such as the CPRCard™ maintain a promising outlook.

## P019 Hyperoxemia and mortality in acute brain injury: a systematic review and preliminary metaanalysis

### N Romero-Garcia, B Monleon, A Ruiz, M Pascual, A Ruiz Pacheco, F Perdomo, JF Marti, A Gutierrez, JA Carbonell, R Badenes

#### Hospital Clinico Universitario De Valencia, Anaesthesiology and Critical Care, Valencia, Spain

*Critical Care* 2024, **28(Suppl 1):** P019

**Introduction:** Oxygen is the most frequently prescribed drug in critically ill patients. It is a well-known fact that hypoxemia correlates to higher mortality in the ICU; however, hyperoxemia could be related to similarly negative outcomes [1]. Patients suffering from acute brain injury, such as traumatic brain injury (TBI), post cardiac arrest (PCA), subarachnoid hemorrhage (SAH) or stroke, may be particularly vulnerable to the detrimental effects of oxygen [2]. The primary objective of our study was to determine if arterial hyperoxia correlates with higher mortality in patients with acute brain injury.

**Methods:** We performed a systematic review in a number of databases for studies on the relationship between hyperoxia and mortality in adult intensive care unit (ICU). The definition of hyperoxia used the arterial PO_2_ stated by each independent study. Studies including paediatric patients, patients with neurological or respiratory disease, or hyperbaric oxygenation were excluded. We evaluated risk of bias through the ROBINS-I tool. Adjusted odds ratio (OR) of patients exposed to hyperoxia versus normoxia were extracted and fixed-effects models were used for quantitative synthesis of data.

**Results:** 6802 records eligible for abstract screening and 236 for full-text screening. The final qualitative synthesis included 35 retrospective studies. The quantitative synthesis included 17 studies, with a total of 78.065 patients with TBI (k = 4), stroke (k = 2) and PCA (k = 11). A significant association was found between hyperoxia and increased mortality in patients with acute brain injury (OR 1.07, 95% CI 1.01–1.15) (Figure).

**Conclusions:** Hyperoxia may be associated with increased mortality in patients with acute brain injury. However, these results should be confirmed through subset analysis of studies with low risk of bias and carefully interpreted due to the significant heterogeneity of included studies.

**References**

1. Kilgannon JH et al. JAMA. 2010;303:2165–2171.
2. Brenner M et al. Arch Surg. 2012;147:1042–1046.

**Figure (abstract P019):**
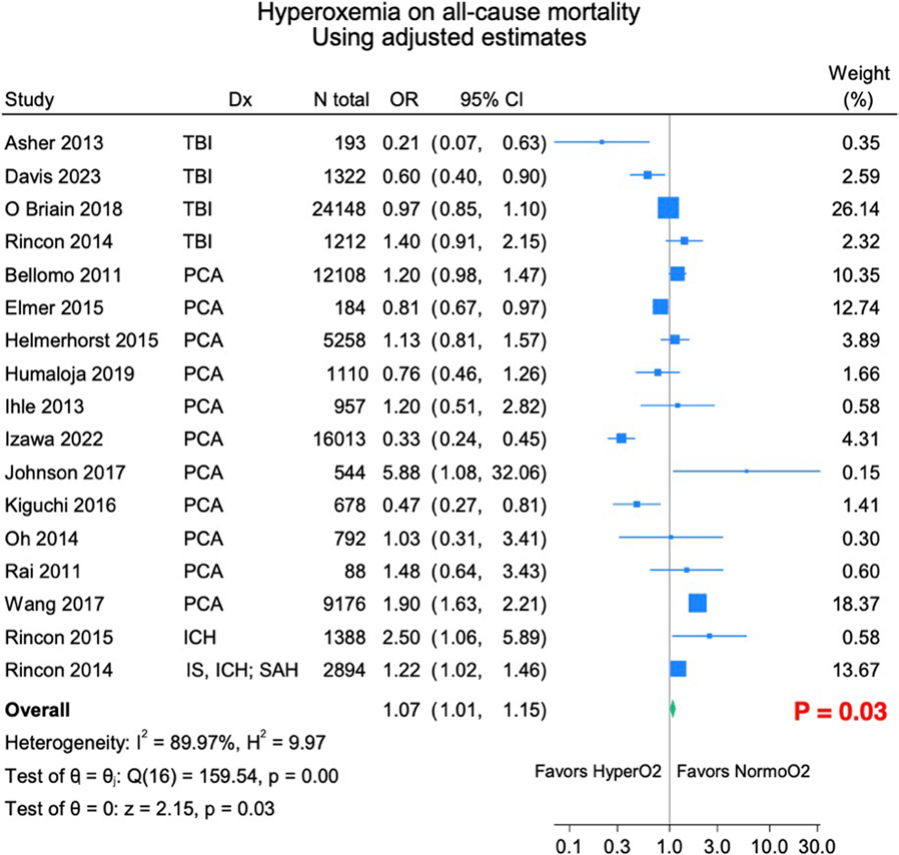
Forest plot showing individual odds ratios for studies on patients with acute brain injury of different causes.

## P020 Impact of early neurocognitive disorders following surgery on clinical outcomes: a comprehensive meta-analysis

### V Likhvantsev^1^, G Landoni^2^, L Berikashvili^1^, N Ermokhina^1^, M Yadgarov^1^, Y Kotani^3^, K Kadantseva^1^, D Makarevich^4^, A Grechko^5^

#### ^1^V. Negovsky Reanimatology Research Institute, Department of Clinical Trials, Moscow, Russian Federation, ^2^IRCCS San Raffaele Scientific Institute, Department of Anesthesia and Intensive Care, Milan, Italy, ^3^Kameda Medical Center, Kamogawa, Japan, ^4^V. Demikhov Municipal Hospital No68, Moscow, Russian Federation, ^5^Federal Scientific and Clinical Center of Reanimatology and Rehabilitation, Moscow, Russian Federation

*Critical Care* 2024, **28(Suppl 1):** P020

**Introduction:** Early postoperative neurocognitive disorders (PND), manifesting as emergence delirium and emergence agitation, remain among the least explored aspects of perioperative neurocognitive disorders. This meta-analysis primarily aims to assess the influence of early PND (ePND) on outcomes that hold clinical significance.

**Methods:** We conducted a systematic review of studies published over the past 20 years using Medline, PubMed, Google Scholar, and Cochrane Library. The protocol of this study was registered on PROSPERO (CRD 42022382008). The inclusion criteria focused on adults with emergence agitation and/or emergence delirium, and outcomes included mortality, postoperative delirium, and length of post-anesthesia care unit and hospital stay. The studies' internal validity, risk of bias, and certainty of evidence were assessed.

**Results:** This meta-analysis synthesized data from 22 studies, encompassing a total patient cohort of 15,935. We observed that ePND were present in 16% of these patients. Notably, the mortality rate for patients with ePND stood at 2.4%, which was double the rate of 1.2% in patients who experienced normal cognitive emergence post-surgery (odds ratio of 2.7, *p* = 0.01). Furthermore, postoperative delirium was significantly more prevalent in the ePND group, affecting 29%, in stark contrast to just 4.6% in those with normal emergence—a 15-fold increase as indicated by an odds ratio of 15.7 (relative risk 9.4, *p* < 0.001) (Figure). ePND was associated with longer post-anesthesia and hospital stays (*p* = 0.004 and *p* < 0.001, respectively).

**Conclusions:** The meta-analysis indicates that ePND is associated with a doubled risk of all-cause mortality and a ninefold increase in postoperative delirium, highlighting the need for further research and improved clinical approaches to ePND management.

**Figure (abstract P020):**
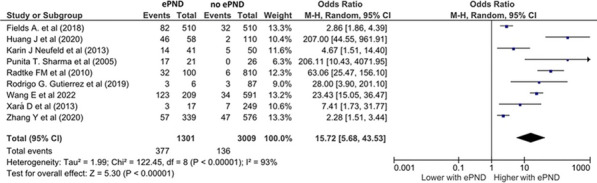
Forest plot for postoperative delirium representing the odds ratio for the effect of normal emergence versus early postoperative neurocognitive disorders (ePND) on postoperative delirium incidence for the included studies.

## P021 Impact of low cardiac output on the development of cerebral hypoxia after acute brain injury

### CA Santacruz^1^, K Puerto^2^, J Alvarado^2^, E Gouvea Bogossian^3^, M Polato^3^, C Robba^4^, D De Backer^5^, FS Taccone^3^

#### ^1^Fundación Santa Fe de Bogotá, Bogota, Colombia, ^2^Fundación Santa Fe de Bogotá, ICU, Bogota, Colombia, ^3^Erasme Hospital, ICU, Brussels, Belgium, ^4^Department of Surgical Science and Integrated Diagnostic, University of Genoa, ICU, Genova, Italy, ^5^CHIREC Hospitals, ICU, Brussels, Belgium

*Critical Care* 2024, **28(Suppl 1):** P021

**Introduction:** Despite the critical role of adequate cerebral oxygenation in managing acute brain injury (ABI), the influence of cardiac index (CI) on regional oxygen metabolism remains poorly understood. This study aimed to assess the impact of CI at intensive care unit (ICU) admission on regional and global cerebral oxygen metabolism in ABI patients.

**Methods:** We prospectively analyzed consecutive ABI patients requiring invasive monitoring of regional partial brain tissue oxygen tension (PbtO_2_), intracranial pressure (ICP), cerebral perfusion pressure (CPP), and global cerebral oxygen metabolism in two academic ICUs. The first available PbtO_2_ measurement was categorized based on CI (< 2.4 vs. ≥ 2.4 L/min), ICP (< 20 vs. ≥ 20 mmHg), and CPP (< 65, 65–80, and > 80 mmHg). Regional hypoxia was defined as PbtO_2_ < 20 mmHg, and global anaerobic metabolism was assessed by SvjO_2_ < 60%, oxygen glucose index (OGI < 6), lactate glucose index (LGI < 0), lactate oxygen index (LOI < 0), or cerebral lactate > 2 mmol/L.

**Results:** We included 51 patients (median age 56.0 years, IQR [44.0–65.0]), most of them (n = 31, 61%) suffered spontaneous subarachnoid hemorrhage. Patients with low CI (1.8 L/min [1.7–2.1]) exhibited significantly lower PbtO2 compared to those with higher CI (12 mmHg [9–20] vs. 24 mmHg [19–32]; *p* = 0.004). Similar associations were observed with elevated ICP (12 mmHg [6–19] vs. 24 mmHg [18–32]; *p* = 0.006) and low CPP (10 mmHg [8–16] vs. 24 mmHg [12–27] vs. 24 mmHg [18–34]; *p* = 0.016). Notably, CI displayed a significant positive correlation with PbtO_2_ (r = 0.3; 95% CI 0.01–0.52; *p* = 0.03). Interestingly, only low CPP levels were associated with elevated cerebral lactate levels (2.65 mmol/L [1.58, 3.88] vs. 2.10 mmol/L [1.65–5.95] vs. 1.20 mmol/L [0.98–1.53]; *p* = 0.02).

**Conclusions:** Low CI at ICU admission significantly impacts regional cerebral oxygenation in ABI patients, independent of ICP and CPP levels. However, it does not appear to directly translate to global anaerobic metabolism as assessed by various indices.

## P022 The role of viral load for COVID-19-induced encephalitis

### L Dogan^1^, D Kaya^2^, N Yurtturan Uyar^3^, A Dincer^4^, S Kocagoz^5^, B Gucyetmez^1^, İO Akinci^6^

#### ^1^Acibadem University, Department of Anesthesiology and Reanimation, İstanbul, Turkey, ^2^Acibadem University, Department of Neurology, İstanbul, Turkey, ^3^Acibadem University, Department of Medical Microbiology, İstanbul, Turkey, ^4^Acibadem University, Department of Radiology, İstanbul, Turkey, ^5^Acibadem University, Department of Infectious Diseases and Clinical Microbiology, İstanbul, Turkey, ^6^Acibadem Altunizade Hospital, Intensive Care Unit, İstanbul, Turkey

*Critical Care* 2024, **28(Suppl 1):** P022

**Introduction:** Numerous studies have linked encephalitis (ECP) to COVID-19, presenting a broad spectrum of clinical manifestations and underlying diverse pathophysiological mechanisms [1]. The viral load of SARS-CoV-2, which can be identified using the Ct value [2], can also be a risk factor for ECP. In the study, we investigated the probable risk factors for encephalitis in patients with COVID-19. We hypothesized that SARS-CoV-2 viral load at ICU admission may predict COVID-19-induced ECP.

**Methods:** This was a single-center retrospective study between May 2020 and August 2022. Patients older than 18 years with COVID-19 whose length of ICU stays (LOS-ICU) was over one week were included. Patients were divided into ECP + and ECP − groups. In the patients’ weaning period, ECP was diagnosed by altered mental status or presence of seizure and positive CSF and MRI findings. Patients' characteristics, laboratories, and the Ct values at the ICU admission and outcomes were recorded.

**Results:** ECP was diagnosed in 38 (39.6%) of 96 patients. Seizure was observed in 10 (27%) patients with ECP. In the ECP + group, age, d-dimer levels, MV duration, and LOS-ICU were significantly higher, whereas Ct levels were significantly lower than in the ECP-group (*p* = 0.006, *p* < 0.001, *p* = 0.006, *p* = 0.009 and *p* < 0.001 respectively). For ECP + , the AUC values of age (> 70), Ct (≤ 26.2) and D-dimer (> 2.3) were 0.67 (0.56–0.79), 0.71 (0.60–0.82) and 0.78 (0.68–0.88) (*p* = 0.004, *p* = 0.001 and *p* < 0.001). In the Cox regression analysis, the risk of ECP + was 1.9-fold (1.1–4.4) and 2.9-fold (1.3–6.6) increased by only Ct ≤ 26.2 and D-dimer > 2.3 at the ICU admission (*p* = 0.032, *p* = 0.010).

**Conclusions:** The viral load at ICU admission is an important risk factor for COVID-19-induced ECP. This risk is increased by an especially high viral load combined with high D-dimer. Moreover, the existence of ECP results in increased MV duration and LOS-ICU.

**References**

1. Pilotto A et al. J Infect Dis. 2021;223:28–37
2. Yu X et al. Crit Care. 2020;24:170

##  P023 Perioperative morbimortality in elective resection of supratentorial tumors, evaluated using the Clavien–Dindo classification in a low-income country: Bogotá, Colombia

### MC Niño^1^, L Rincon^1^, D Cohen^1^, W Amaya-Zuñiga^1^, D Benitez^1^, A Obando^1^, A Camargo^2^, L Carvajal^2^, MI Daza^2^, JF Parada-Márquez^1^

#### ^1^Hospital Universitario Fundacion Santa Fe de Bogota, Anesthesiology, Bogotá, Colombia, ^2^ Universidad de los Andes, Faculty of Medicine Bogota, Colombia

*Critical Care* 2024, **28(Suppl 1):** P023

**Introduction:** Supratentorial tumor resection surgery (STTR) results in significant perioperative morbidity and mortality [1]. We describe the perioperative mortality and morbidity of patients undergoing elective STTR in a low-income country using the Clavien-Dindo (CD) classification as a validated scale of postoperative complications (PC) and an indicator of quality of care.

**Methods:** This descriptive retrospective cohort study was conducted between 2019 and 2023. We sought to describe the sociodemographic variables, preoperative characteristics, and postoperative morbimortality of patients who underwent STTR at Fundación Santa Fe de Bogotá in Bogotá, Colombia.

**Results:** A total of 201 patients were included in the study, including 83 men and 118 women, with an average age of 53 years. Among women, 41.5% had meningiomas, 21% had gliomas, and 19.5% had pituitary adenomas. In males, 19% of the tumors were meningiomas, 38% were gliomas, and 16.8% were pituitary adenomas. Preoperative anemia was present in 35.3% of the patients, but only 2.5% required blood transfusions. Of these, 23.8% presented with PC. According to the CD scale, 11.94% presented with type 1 complications, 7.96% with type 2 complications, 1% with type 3 complications, and 1% with type 4 complications. Renal failure occurred in 0.5% of patients and readmission to the ICU in 2.9%, 4.4%, and 4.2% of patients, respectively. The median hospital stay was 4 days (IQR 3–5) and 30-day mortality was 0.5%.

**Conclusions:** We observed that teamwork and the use of care protocols allowed good quality of care with a low rate of complications [1] (mostly CD type 1 and 2) and mortality in this group of patients from a low-income country, despite the fact that a high percentage of patients presented preoperative risk factors such as PA.

**Reference**

1. Senders JT et al. Neurosurgery. 2018;83:1249–59

## P024 Preoperative anemia and its impact on supratentorial tumor resection complications in a high-altitude resident population

### MC Niño^1^, L Rincon^1^, JA Mejia^2^, W Amaya-Zuñiga^1^, D Benitez^1^, A Obando^1^, MI Daza^3^, JF Parada-Márquez^1^, S Ruiz^3^, S Corredor^3^

#### ^1^Hospital Universitario Fundación Santa Fe de Bogotá, Anesthesiology, Bogotá, Colombia, ^2^Hospital Universitario Fundación Santa Fe de Bogotá, Neurosurgery, Bogotá, Colombia, ^3^Universidad de Los Andes, Faculty of Medicine, Bogotá, Colombia

*Critical Care* 2024, **28(Suppl 1):** P024

**Introduction:** Preoperative anemia (PA) is a known risk factor for postoperative complications (PC). The aim of this study was to describe the impact of PA on PCs in patients undergoing Supratentorial Tumor Resection (STTR) in a hospital located 2600 m above sea level (mSNM).

**Methods:** A historical cohort of elective patients who underwent STTR at the Fourth-level University Hospital Bogotá from 2019 to 2022 was analyzed. PA-related PC was analyzed to determine the length of hospital stay (LOS). Descriptive statistics of the variables were performed, followed by an analysis of the medians and difference in the sum of ranges of hospital stay. In addition, Spearman or Pearson’s correlations were performed, as appropriate. The definition of anemia was adjusted for height in mSNM (hemoglobin males < 14.3 and females < 13.3 g/dL).

**Results:** A total of 201 patients were included in the analysis: 41.29% men and 58.71% women. Mean age 53.38 years (sd:13.96). The prevalence of anemia was 35.32%. The mean hemoglobin level was 14.3 Â g/dL (sd 1.55); in the anemia group it was 12.7 (sd 1.02) versus 15.13 (sd 1.11) (*p* = 0.00001). More complications were found in patients with PA, which were statistically significant for operative site infections (*p* = 0.043). PA was associated with a higher LOS because of the difference between the sum of the ranks (*p* = 0.003). An inverse correlation was found between hemoglobin levels and hospital stay (⍴:− 0.2; *p* = 0.004). The Table shows the behavior of the LOS and its variables. It can be seen that LOS was associated with PA and a higher rate of reoperation and readmission to the intensive care unit.

**Conclusions:** PA was frequent in our STTR population and associated with a higher LOS and incidence of operative site infection.

**Table (abstract P024):** Relationship between anemia, quartiles of median hospital stay, and postoperative complications in patients undergoing supratentorial tumor resection at 2600 m above sea level

n = 201	Length of stay (days) M	(p25–p75)	IQR	*p* value
Age (years) < 60 versus ≥ 60	(4 vs. 4)	(3–5 vs. 3–5)	(2 vs. 2)	0.8 u
Renal failure No versus Yes	(4 vs. 3)	(3–5 vs. 3–3)	(2 vs. 0)	0.5 u
Preoperative anemia* No versus Yes	(4 vs. 4)	(3–5 vs. 3–5)	(2 vs. 2)	0.03 u
Transfusion requirement No versus Yes	(4 vs. 11)	(3–5 vs. 5–45)	(2 vs. 40)	0.007 u
Surgical site infection No versu Yes	(4 vs. 45)	(3–5 vs. 3–92)	(2–88)	0.1 u
ICU readmission No versus Yes	(4 vs 26.5)	(3–5 vs. 4–92)	(2 vs. 88)	0.03 u
Reoperation No versus Yes	(4 vs 11)	(3–5 vs. 6–45)	(2 vs. 39)	0.000 u

^u^Mann Whitney U test + Kruskal Wallis test *There were significant differences between the two groups; however, this was due to the length of stay of the patients in the extreme preoperative anemia category.

## P025 A rare complication of a trans-thoracic core biopsy

### D Correia^1^, J Gonçalves^2^, P La Feria^3^, G Nobre de Jesus^1^, C Candeias^1^, JM Ribeiro^1^

#### ^1^Centro Hospitalar Universitário Lisboa Norte, Intensive Care Medicine Department, Lisboa, Portugal, ^2^Centro Hospitalar do Oeste, Serviço de Medicina Intensiva, Caldas da Rainha, Portugal, ^3^Hospital Prof. Doutor Fernando Fonseca, Serviço de Medicina Intensiva, Amadora, Portugal

*Critical Care* 2024, **28(Suppl 1):** P025

**Introduction:** Percutaneous CT-guided biopsies are recommended for suspicious lung lesions. They are considered safe, minimally invasive and highly accurate for neoplasm diagnosis. Its most frequently reported complications are pneumothorax (20–30% incidence) and alveolar hemorrhage (4–30%). Systemic air embolism (SAE) is a potentially life-threatening but extremely rare complication. With a radiological incidence of 3.8%, only 0.49% are clinically apparent and only 0.16% have associated mortality.

**Methods:** A review of the literature was conducted, with a posterior analysis and a description of the clinical case.

**Results:** A 66-year-old patient with a smoking history referred anorexia and weight loss. A thoracic CT-scan identified a right superior upper lobe spiculated pulmonary lesion, 20 mm of diameter, that doubled the size in 6 months, with a high standardized uptake value of 3.5 on the PET scan. A pulmonary core biopsy was performed under local anesthesia. Immediately after, sudden coma emerged, and cardiac arrest ensued with pulseless electrical activity. Advanced life support was initiated with return of spontaneous circulation after 8 min, but he remained in coma (GCS 3). Thoracic and brain CT-scan performed immediately showed right pneumothorax; air in the left lateral ventricle and in the high brain convexity bi-hemispheric subarachnoid space (Figure). A right chest drain was placed, and the patient referred to hyperbaric oxygen therapy. Reevaluation of brain CT-scan after a 7-h session showed total absorption of the air emboli. Post-resuscitation care was instituted at the ICU and protocolized neuroprognostication showed signs of poor anticipated recovery. The patient remained in coma and died from irreversible brain lesion.

**Conclusions:** Although the needle core biopsy is described as safe, there is a real risk of life threatening complications, as the case described. Efforts should be made to protocolize the care in these scenarios, including in particular hyperbaric oxygen therapy and ICU admission.

**Acknowledgement:** Written consent to publish was received from the patient’s family.

**Figure (abstract P025):**
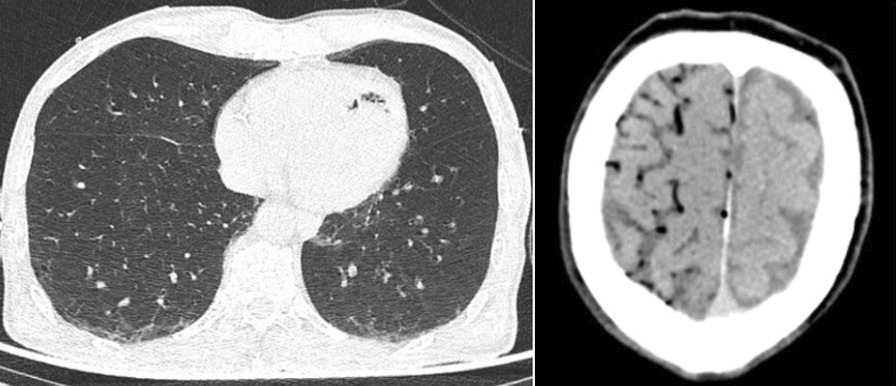
On the left side a thoracic CT-scan showing air emboli in the left ventricle; on the right side a brain CT-scan showing SAE in the bi-hemispheric subarachnoid space of the high brain convexity.

## P026 Continuous EEG as a neurophysiological tool in a critical care setting

### M Papaioannou, M Tzimou, F Davora, I Mouskeftara, E Eleftheriadou, V Tsiapara, C Charachristos, G Vasileiadou, A Lavrentieva

#### General Hospital of Thessaloniki "G. Papanikolaou", A ICU, Thessaloniki, Greece

*Critical Care* 2024, **28(Suppl 1):** P026

**Introduction:** The aim of the study was to evaluate the clinical status of comatose ICU patients using continuous electroencephalography (cEEG) and its association with disease prognosis.

**Methods:** This was an observational, prospective study including adult patients in a single Intensive Care Unit (ICU). Patients’ demographic characteristics, severity of illness, comorbidities, reason for undertaking EEG, pattern of EEG, management of therapeutic strategies were recorded and were related to the prognosis.

**Results:** Data of 55 patients were analyzed (median age 61 years (range 19–86), median APACHE II score 22 (range 5–38) and median SOFA score 9 (range 4–16) at admission. Median duration of mechanical ventilation was 35 days and median ICU length of stay (LOS) was 42 days. The majority of cEEG (61.1%) was performed due to medical reasons (ichaemic stroke, septic shock, status epilepticus); 25.9% of cEEG was conducted in neurosurgical patients (both post traumatic acute brain injury and malignancy) whereas 13% was carried out in post-anoxic comatose patients. Patients who started anticonvulsant therapy after EEG examination had higher mortality rate than patients who had already received anticonvulsant medications (x^2^ = 8.077, *p* = 0.004). In more than half of the patients encephalopathic EEG pattern was observed in comparison with a lower percentage of patients who had lateralized periodic discharges (LPDs); one patient had burst suppression and one patient had electrocerebral inactivity (ECI) or silence (ECS). Patients with epileptic disorders had higher mortality rate (*p* = 0.018) in comparison with the other categories of patients. Taking their medical comorbidities into consideration, patients diagnosed with diabetes mellitus were more likely to have higher mortality (x^2^ = 5.115, *p* = 0.045).

**Conclusions:** Continuous EEG improves management of patients, could modify therapeutic strategies and appears to be a useful prognostic tool in critical care patients.

## P027 Factors correlated with delay or non-delay presentation for acute stroke in emergency department

### DI Popa^1^, C Williams^1^, F Buleu^2^, D Șutoi^1^, C Trebuian^1^, A Iancu^3^, OA Mederle^1^

#### ^1^Victor Babes University of Medicine and Pharmacy, Surgery, Timisoara, Romania, ^2^Victor Babes University of Medicine and Pharmacy, Cardiology, Timisoara, Romania, ^3^Victor Babes University of Medicine and Pharmacy, Radiology, Timisoara, Romania

*Critical Care* 2024, **28(Suppl 1):** P027

**Introduction:** The incidence of acute stroke in the ED is increasing by up to 7% annually, well beyond that explained by the increase in average population size. Furthermore as statistical reports showed that the country with the highest rates of new strokes and deaths due to stroke is Romania. According to studies, the time elapsed from onset to reperfusion treatment is very important, and according to the current protocol, only those with onset up to 4.5 h can perform thrombolytic therapy and/or up to 6 h, mechanical reperfusion therapies such as thrombectomy.

**Methods:** The study was carried out in the Emergency Municipal Clinical Hospital, the second-largest hospital with about 30,000 annual ED patient visits. All patients included in this study presenting to the ED with signs and symptoms of stroke between January 1, 2019, and December 31, 2022, identified as stroke patients in time of thrombolysis were included.

**Results:** Of the 202 interviewed patients (Table), 104 (51.48%) presented late (> 4.5 h) after onset of stroke and 98 (48.51%) presented early. Statistically significant correlations were observed between the higher frequency of right hemiparesis and patients who arrived after 4.5 h (29.59% vs. 40.38%, *p* = 0.010) and between loss of consciousness and aphasia in patients who arrived early (≤ 4.5 h) (13.26% vs. 1.92%, *p* = 0.004, respectively (61.22% vs. 21.15%, *p* < 0.001). Statistically significant risk factors associated with early arrival (≤ 4.5 h) were smoking and previous stroke/TIA.

**Conclusions:** Approximately 51.48% presented after 4.5 h of stroke onset. Of the factors that contribute to the delay in admission to the ED after the onset of stroke symptoms, most can be overcome. To improve community awareness of the signs and symptoms of stroke, health promotion campaigns are needed, and timely transfer to hospitals with thrombolysis facilities and effective use of the ambulance service are strategies that help early presentation to reperfusion treatment after stroke onset.

**Table (abstract P027):** Characteristics of patients

Risk factors	Full sample, n = 202	Under 4.5 h, n = 98	Over 4.5 h, n = 104	*p* value
Obesity	12 (5.94%)	8 (8.16%)	4 (3.84%)	0.120
Smoking	37 (18.31%)	25 (25.51%)	12 (11.53%)	0.005*
Alcohol	31 (15.34%)	16 (16.32%)	17 (16.34%)	0.986
Dyslipidemia	22 (10.89%)	8 (8.16%)	14 (13.46%)	0.7
Arterial hypertension	164 (81.18%)	73 (74.48%)	91 (87.5%)	0.670
Diabetes mellitus	53 (26.23%)	27 (27.55%)	26 (25.0%)	1.00
Atrial fibrillation	40 (19.80%)	15 (15.30%)	25 (24.03%)	0.752
Anticoagulant treatment	34 (16.63%)	16 (16.32%)	18 (17.30%)	0.950

## P028 Analysis of inflammatory biomarkers in predicting delayed cerebral ischemia in non-traumatic subarachnoid hemorrhage (SAH)

### S Serra Soler^1^, M Sánchez Satorra^2^, MT Misis del Campo^2^, M Alcalde-Herraiz^3^, M Català^4^, J Ferrés Llach^2^, I Selva Armadans^1^, M Bauçà Socías^2^, T Pons Lopez^2^, D Mota Montané^2^

#### ^1^Hospital Germans Trias i Pujol, Medicina Intensiva, Badalona, Spain, ^2^Hospital Germans Trias i Pujol, Intensive Care Unit, Badalona, Spain, ^3^Universitat Politècnica de Catalunya, Barcelona, Spain, ^4^Oxford University, Oxford, UK

*Critical Care* 2024, **28(Suppl 1):** P028

**Introduction:** Subarachnoid hemorrhage (SAH) represents a neurological emergency. Following an aneurysm rupture, an inflammatory cascade is initiated, contributing to the development of delayed cerebral ischemia (DCI), exacerbating the functional prognosis. The study aims to identify inflammatory biomarkers in plasma significantly associated with the risk of DCI.

**Methods:** A prospective observational study conducted in a ICU from January 2019 to September 2022. Inclusion criteria comprised patients over 18 years with a SAH diagnosis, excluding those with uncertain bleeding chronology or an unfavorable vital prognosis at admission. Blood and cerebrospinal fluid biomarkers involved in neuroinflammation, thrombosis, and nitric oxide regulation were analyzed on days 0, 3, and 7 of admission. Inflammatory parameters included leukocytes (and their ratios), fibrinogen, ferritin, troponin, D-dimer, procalcitonin, interleukin-6 (IL-6), and C-reactive protein (CRP) (Table). Univariate analysis of normally distributed variables used the ANOVA test, while categorical variables employed the χ^2^ test. A *p* value less than 0.05 was considered statistically significant. Data are expressed as means and standard deviation.

**Results:** 162 patients were included with a mean age of 57 years (25–88 years), 96 women (57%). APACHE II scored 20 points (4–39 points), *p* = 0.01. DCI occurred in 47 patients (29%). Hunt–Hess severity: I 16% (12% developed DCI), II 34% (22%), III 23% (43%), IV 16% (23%), V 11% (56%). Hunt-Hess severity significantly associated with DCI presence (*p* = 0.01). 75% had Fisher IV SAH, with 32% developing DCI but no significant association (*p* = 0.26). Biomarker results are summarized in the Table.

**Conclusions:** Nearly one-third of SAH patients exhibit delayed cerebral ischemia, associated with clinical severity (Hunt-Hess and Apache II) at admission, rather than Fisher scale severity. Leukocyte counts, neutrophils, CRP, IL-6, and ferritin values at admission are significantly linked to DCI development in SAH patients.

**Table (abstract P028):** Results

	DAY 0	DAY 3	DAY 7
Leukocytes (× 10^9^/L)	No DCI: 13.5 (4.9) Yes DCI: 14.7 (4.4) *p* = 0.04	No DCI: 11.6 (3.7) Yes DCI: 13.2 (5) *p* = 0.01	No DCI: 11 (3.3) Yes DCI: 11.1 (4.2) *p* = 0.99
Neutrophils (%)	No DCI: 82 (9.4) Yes DCI: 85.4 (6.3) *p* = 0.01	No DCI: 76.7 (9.6) Yes DCI: 78.3 (8.6) *p* = 0.24	No DCI: 73.1 (10.1) Yes DCI: 73.9 (8.4) *p* = 0.71
Fibrinogen (mg/dL)	No DCI: 434.8 (99.1) Yes DCI: 448.1 (102.9) * p* = 0.0	No DCI: 571.8 (177.9) Yes DCI: 643.5 (188) * p* = 0.03	No DCI: 617.1 (196.5) Yes DCI: 746.6 (247.5) * p* = 0.0
CRP (mg/L)	No DCI: 11.9 (22.9) Yes DCI: 16.4 (21.1) * p* = 0.04	No DCI: 47.3 (59.5) Yes DCI: 70.1 (70.1) * p* = 0.22	No DCI: 36.6 (55.9) Yes DCI: 68.1 (92.3) * p* = 0.01
Interleukin-6 (pg/mL)	No DCI: 25.4 (38.9) Yes DCI: 49.9 (125.4) * p* = 0.03	No DCI: 43.2 (93.9) Yes DCI: 37.8 (46.8) * p* = 0.83	No DCI: 35.1 (126.1) Yes DCI: 19.2 (18.2) * p* = 0.5
Ferritin (ng/mL)	No DCI: 117 (75.5) Yes DCI: 268.9 (183.9) * p* = 0.01	No DCI: 176.3 (108) Yes DCI: 317.5 (173.6) * p* = 0.01	No DCI: 240.3 (155.8) Yes DCI: 442.3 (205.1) * p* = 0

Data expressed as median (standard).

## P029 Assessment of platelet inhibition secondary to antiplatelet therapy using thromboelastography-platelet mapping (TEG-PM) in South-East Asian (SEA) patients with spontaneous intracerebral haemorrhage (sICH): a pilot feasibility observational study

### M Chew^1^, M Ye^2^, K Cao^1^, YJD Quek^3^, YT Chew^1^, YL Wong^1^

#### ^1^Tan Tock Seng Hospital, Department of Anaesthesiology, Intensive Care and Pain Medicine, Singapore, Singapore, ^2^Khoo Teck Puat Hospital, Department of Anaesthesia, Singapore, Singapore, ^3^Tan Tock Seng Hospital, Department of Emergency Medicine, Singapore, Singapore

*Critical Care* 2024, **28(Suppl 1):** P029

**Introduction:** In patients with antiplatelet-associated sICH, rapid identification of patients with platelet dysfunction is essential in guiding blood product administration and correction of coagulopathy, particularly in the setting of neurosurgical intervention. TEG-PM is designed to assess platelet inhibition due to antiplatelet therapy [1]. In SEA patients with sICH, the role of this test is still unclear. The objective of this study was to evaluate the usefulness of TEG-PM in SEA patients with sICH on aspirin and/or clopidogrel.

**Methods:** This is a prospective observational cohort study of patients admitted to a SEA intensive care unit from August 2022 to June 2023 with sICH. Patients were divided into two groups: those taking aspirin, and/or clopidogrel, and a control group. Patients on anticoagulants were excluded. TEG-PM was performed within 6 h after symptom onset. Computed tomography head (CTH) was obtained on admission and at 24 ± 12 h.

**Results:** Thirty-four patients with sICH were included. 14 were on aspirin or clopidogrel, and 20 were controls. Patients on antiplatelet had higher mean arachidonic acid (AA) and adenosine diphosphate (ADP) inhibition than controls. Mean (SD) AA inhibition was 88.5%(13.4) in the antiplatelet group, and 54.4%(38.2) in the control group (*p* < 0.001). Mean maximum amplitude was higher in controls than the antiplatelet group (36.7 vs. 24.1, *p* = 0.025). Platelet dysfunction was observed in 7 controls; the dysfunction was not accounted for by antiplatelet therapy. Haemorrhagic expansion on CTH was not associated with increase or decrease in AA inhibition (*p* = 0.29).

**Conclusions:** This is the first prospective study in SEA patients. TEG-PM is able to detect platelet inhibition in SEA patients with sICH on antiplatelet. However, the overlap in platelet inhibition values between the antiplatelet and control group may confound its clinical usefulness and defining suitable transfusion thresholds. This warrants further investigation.

**References**

1. Xu FWX et al. Euro J Med Res. 2022;27:191

## P030 Delayed ischemic neurological deficit detection and management after aneurysmal subarachnoid hemorrhage: a 7-year retrospective monocentric study

### L Patrini^1^, F Graziano^2^, P Gilardi^3^, L Meletti^3^, F Elli^3^, M Baggiani^3^, A Guglielmi^1^, P Remida^4^, CG Giussani^5^, G Citerio^6^

#### ^1^IRCCS Policlinico San Matteo di Pavia, Department of Clinical-Surgical Diagnostic and Paediatric Sciences, Unit of Anaesthesia and Intensive Care, Pavia, Italy, ^2^Fondazione IRCCS San Gerardo dei Tintori, Neuroscience Department, NeuroIntensive Care Unit, Monza, Italy, ^3^Fondazione IRCCS San Gerardo dei Tintori, Neuroscience Department, Neurosurgery, Monza, Italy, ^4^Fondazione IRCCS San Gerardo dei Tintori, Neuroscience Department, Neuroradiology, Monza, Italy, ^5^Fondazione IRCCS San Gerardo dei Tintori, Neuroscience Department, Neurosurgery, Fondazione IRCCS San Gerardo dei Tintori, Monza, Italy, School of Medicine and Surgery, University of Milano-Bicocca, Milan, Italy, ^6^Fondazione IRCCS San Gerardo dei Tintori, Neuroscience Department, NeuroIntensive Care Unit, Monza, Italy, School of Medicine and Surgery, University of Milano - Bicocca, Milano, Italy

*Critical Care* 2024, **28(Suppl 1):** P030

**Introduction:** Delayed ischaemic neurological deficit (DIND) represents the initial onset of a new focal or global neurological deficit (defined as a two-point decrease in neurological function). Detection and management of DIND after aneurysmal subarachnoid haemorrhage (aSAH) are very heterogeneous. Our study aims to provide a description of DIND episodes and applied therapeutic strategies, comparing them with functional outcomes.

**Methods:** A 7-year monocentric observational retrospective study of a total 270 consecutive aSAH treated patients to describe the deficit and compare only medical therapy and rescue therapy.

**Results:** Through electronic medical records, it was found that 27.8% experienced DIND, typically around the 6.5th day, documented at discharge as clinical/angiographic evidence of vasospasm, requiring specific treatment (Table). All patients with DIND received hemodynamic therapy; in 18 of them the deficit resolved (average saline bolus = 1.26 L/24 h; maximum mean dose of norepinephrine = 0.18 mcg/kg/min; ∆mean arterial pressure = 24 mmHg). No episodes of rebleeding after the increased pressure. 57 patients required rescue therapy, with all of them receiving intra-arterial nimodipine; 57.9% underwent angioplasty. 87.7% needed external ventricular drainage. Episodes of high-ICP occurred in 63.2% of this group. The mean ICP value that triggered therapy was 24.5 mmHg; treatment involved hypertonic (47.4%), mannitol (36.8%) and decompressive craniectomy (10.7%). There was no significant difference between the two groups in terms of in-hospital mortality (23.5% vs. 17.9%). However, comparing the overall population with DIND patients, a significant difference was found in the functional outcome at discharge: respectively, went home 139 (52.1%) versus 23 (31.5%).

**Conclusions:** The difficulty with DIND remains early recognition and the high functional impact among people in their working years. In this study, vasopressors have been shown to be effective treatment strategies without evidence of increased complications/poor-outcome.

**Table (abstract P030):** Comparison of baseline characteristics between DIND patients receiving medical therapy only and those requiring rescue therapy

	Only medical therapy N = 18	Rescue therapy N = 57
Female; Age > 70, yes (%)	15 (83.3); 4 (22.2)	39(68.4); 8 (14)
Hypertension	7 (38.9)	22 (38.6)
WFNS grading scale > 3	6 (33.3)	21 (36.8)
mFisher > 2	14 (77.8)	42 (73.7)
Spoke access	4 (22.2)	29 (50.9)
Anterior circulation aneurysm	17 (94.4)	54 (94.7)
Pre procedural rebleeding	3 (16.7)	11 (19.3)

## P032 Role of UCH-L1/GFAP and t-Tau/GFAP ratios in predicting neuronal and glial injury following systemic insults in traumatic brain injury

### E Ioppolo^1^, F Graziano^2^, M Baggiani^2^, L Patrini^3^, A Guglielmi^3^, G Citerio^2^

#### ^1^Università degli Studi di Milano-Bicocca, Dipartimento di Medicina e Chirurgia, Monza, Italy, ^2^Fondazione IRCCS San Gerardo dei Tintori, Neuroscience Department, NeuroIntensive Care Unit, Monza, Italy, ^3^University of Pavia, Department of Clinical-Surgical Diagnostic and Paediatric Sciences, Unit of Anaesthesia and Intensive Care, Pavia, Italy

*Critical Care* 2024, **28(Suppl 1):** P032

**Introduction:** The aim of this study is to assess if early systemic insults (SIs) have a different impact on neuronal and glial cells, and consequently on three brain injury biomarkers (GFAP, t-Tau and UCH-L1) and their ratios (UCH-L1/GFAP and t-Tau/GFAP). SIs, specifically hypoxemia and hypotension, aggravate brain damage following traumatic brain injury (TBI) and are associated with injury severity, treatment intensity in the intensive care unit (ICU), and patient outcomes.

**Methods:** We extracted data from ICU patients recruited for the CENTER-TBI study and divided them into four groups according to the occurrence of SIs in the prehospital setting: hypoxemia (SpO_2_ < 90%), hypotension (SB* p* < 90 mmHg), both, no SIs. We then compared neuronal (expressed by the release of UCH-L1 and t-Tau) and glial (expressed by the release of GFAP) injury and the biomarker ratios among the groups.

**Results:** A total of 1695 patients were included in the analysis. The impact of SIs on neuronal and glial injury showed significant differences among the four groups (Table). UCH-L1 and t-Tau levels were higher in the presence of hypotension and both SIs compared to the no-SIs group, whereas they showed only a slight increase in the case of hypoxemia alone. Conversely, GFAP values did not differ significantly among the four groups. Additionally, the UCHL-1/GFAP and t-Tau/GFAP ratios displayed higher values in hypotension and both SIs groups and minimum values in patients without SIs or with hypoxemia only.

**Conclusions:** Early SIs led to the release of brain injury biomarkers, with a more significant increase in biomarkers of cell body and axonal injury than of glial injury. Moreover, there was a clear hierarchy among the four groups, with patients affected by hypotension and both SIs showing the highest values. This was further confirmed by the rise in the two ratios. These findings could have potential implications for the management of hypotension and hypoxemia in the prehospital setting.

**Table (abstract P032):** Results

	No SIs	Hypoxemia	Hypotension	Both
N (%)	1280 (75.5)	158 (9.3)	142 (8.4)	115 (6.8)
UCH-L1***	245.90 (108.8, 531.7)	344.50 (182.5, 717.9)	362.71 (177.8, 849.4)	449.03 (240.1, 887.9)
t-Tau***	6.29 (3.0, 13.4)	7.90 (4.1, 18.6)	9.43 (4.6, 20.7)	10.43 (5.5, 19.3)
GFAP°	14.66 (4.8, 36.7)	17.63 (7.5, 41.2)	15.92 (5.7, 42.7)	17.18 (6.0, 50.8)
Ratios				
UCH-L1/GFAP***	16.86 (11.9, 26.7)	17.44 (11.95, 33.3)	20.38 (14.4, 30.4)	23.19 (15.2, 53.1)
t-Tau/GFAP*	0.47 (0.3, 0.8)	0.43 (0.3, 0.96)	0.56 (0.3, 1.1)	0.62 (0.4, 1.4)

Data are median (IQR); first value; Abbreviations: GFAP = glial fibrillary acidic protein (mcg/L), UCH-L1 = ubiquitin C-terminal hydrolase L1 (pg/mL), t-Tau = total Tau (pg/mL). Kruskal–Wallis test for the comparison of SIs groups. *P* value signif. codes: *** < 0.001, **0.001; *0.01; °0.1

## P033 The cerebral adaptive index (CAI): a novel index to monitor cerebral autoregulation in real-time

### A Albanese^1^, P Benni^1^, Z Jian^1^, F Hatib^1^, H Liu^2^, D Veelo^3^, A Vlaar^3^, R Immink^3^, REC van den Dool^3^, CW Hogue^4^

#### ^1^Edwards Lifesciences, Irvine, USA, ^2^UC Davis Health, Sacramento, USA, ^3^Amsterdam UMC, Amsterdam, Netherlands, ^4^Northwestern University Feinberg School of Medicine, Chicago, USA

*Critical Care* 2024, **28(Suppl 1):** P033

**Introduction:** Cerebral autoregulation (CA) maintains cerebral blood flow (CBF) within a lower limit (LLA) and an upper limit (ULA) of perfusion pressure range. There is wide intra-patient LLA and ULA variability. Perioperative exposure to mean arterial pressure (MAP) outside of these limits is presumably linked to postoperative complications. We have developed a novel index, the Cerebral Adaptive Index (CAI), which quantifies the effectiveness of CA in real-time on a 0–100 scale. CAI is derived from MAP and regional cerebral oxygen saturation (StO_2_) measurements based on modified coherence analysis. We sought to validate the capability of CAI to characterize the status of CA against CA labels obtained from simultaneous measurements of MAP and transcranial doppler (TCD) CBF velocity (CBFV).

**Methods:** We conducted a prospective observational study in 50 surgical patients to simultaneously collect MAP, cerebral StO_2_, and CBFV data. Individual plots of CBFV versus MAP were constructed and the LLA and ULA determined. CAI values were generated by postprocessing MAP and cerebral StO_2_ data through the CAI algorithm. Receiver Operating Characteristic (ROC) analysis was then conducted to assess the capability of CAI to discriminate the two classes: 1. The dysfunctional autoregulation class, where MAP is beyond the individual LLA/ULA limits; 2. The functional autoregulation class, where MAP is within the LLA/ULA limits.

**Results:** Enrolled patients (12 females, 38 males; mean ± SD age, 63 ± 14 years), underwent cardiac (n = 27) or non-cardiac (n = 23) surgeries. The ROC analysis (Figure) shows area under the curve (AUC), sensitivity, and specificity of 0.94, 0.85, and 0.954, respectively. These results demonstrate the capability of CAI to discriminate the state of functional autoregulation from the state of dysfunctional autoregulation.

**Conclusions:** Real-time monitoring of cerebral autoregulation based on MAP and regional cerebral StO_2_ is feasible. The proposed CAI index may allow for personalized CA-oriented MAP targets.

**Figure (abstract P033):**
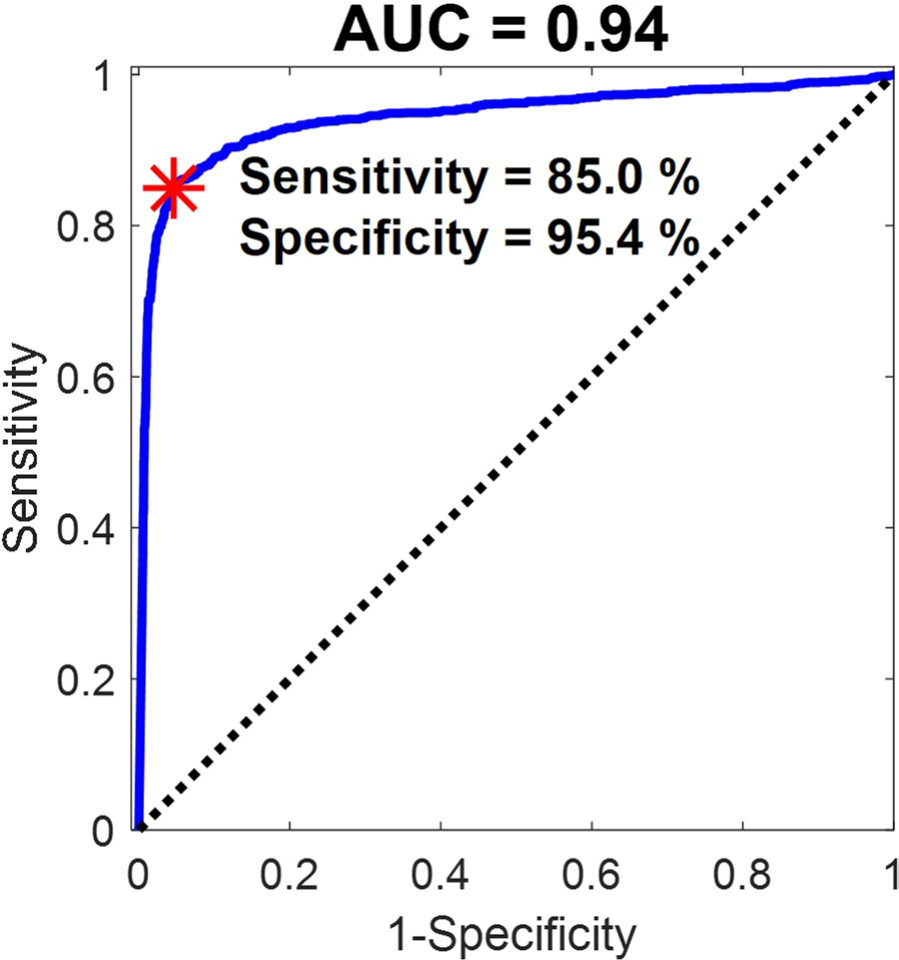
ROC curve for CAI. The reported values of sensitivity and specificity are obtained with a CAI threshold value of 45, chosen in order to minimize false positive (FP) rate while preserving true positive (TP) rate. TP are dysfunctional autoregulation MAP data points with CAI values above threshold; FP are functional autoregulation MAP data points with CAI values above threshold.

## P034 IL-1β and TNF-α association with functional outcome in adults with traumatic brain injury admitted to ICU: a prospective cohort

### E Cáceres, JC Olivella, AE Viñan Garces, LF Reyes

#### Facultad de Medicina, Universidad de La Sabana, Bogota, Colombia

*Critical Care* 2024, **28(Suppl 1):** P034

**Introduction:** A prospective cohort was conducted to establish the association between inflammatory cytokines and outcomes in patients with traumatic brain injury (TBI) admitted to the intensive care unit (ICU). The inflammatory response after TBI might play a major role in healing and recovery. However, a dysregulated inflammatory response could be deleterious. A better understanding of these dynamics could improve TBI outcomes.

**Methods:** From December 2019 to August 2023, we conducted a prospective cohort in a single Colombian center. Patients older than 17 y, admitted to the ICU for moderate and severe TBI, and an mRS < 3 were enrolled. Data was collected in RedCap, including demographics, the severity of the injury, lab tests, and hospital variables. Blood samples were drawn at 72 h post-injury. IL-1β, IL-6, and TNF-α levels were measured. Outcomes at 6 months were evaluated using the Glasgow Outcome Scale-Extended (GOSE). The unfavorable outcome was defined as GOSE < 4. Univariate and multivariate analysis assessed the association between inflammatory cytokines and GOSE. RStudio was used for the analysis.

**Results:** During the study, 96 patients were included. The median (IQR) age was 35.5 (24.2–53.7), and the most frequent cause was road traffic accidents (58%). 83/96 patients (86%) had severe TBI (AIS head ≥ 3). At 6-month follow-up, 41/96 (42%) had GOSE < 4. Univariate analysis revealed significant associations between unfavorable outcomes and age, head AIS, APACHE II, TNF-α, IL-1β, and IL-6 at 72 h. Multivariate logistic regression, adjusted by age, head trauma severity, and systemic compromise, yielded three models for each cytokine. Following adjustment, IL-1β and TNF-α showed significant association (Table).

**Conclusions:** In this cohort, higher levels of IL-1B and TNF-α measured at 72 h post-injury were positively correlated to fatality and disability. Further research is needed to understand the role of inflammation after TBI and its use as a potential therapeutic target.

**Table (abstract P034):** Univariate and multivariate analysis of clinical variables and cytokines with a 6-month unfavorable outcome in patients with TBI admitted to ICU

Variables	Univariate analysis (OR [95% CI]; *p* value)	IL-1β Multivariate analysis (OR [95% CI]; *p* value	TNF-α Multivariate analysis (OR [95% CI]; *p* value)
Age	1.04 [1.01–1.07]; 0.01	1.06 [1.02–1.11]; < 0.01	1.04 [1.01–1.09]; 0.03
Admission GCS	0.77 [0.68–0.88]; < 0.01		
AIS head	3.10 [1.67–5.76]; < 0.01	2.63 [1.08–6.36]; 0.03	2.77 [1.07–7.16]; 0.03
APACHE II	1.21 [1.10–1.33]; < 0.01	1.21 [1.05–1.38]; 0.01	1.21 [1.05–1.40]; < 0.01
Interleukin-1β at 72 h of admission	1.35 [0.93–1.95]; 0.11	1.48 [1.01–2.18]; 0.04	
Interleukin 6 at 72 h of admission	1.01 [1.00–1.02]; 0.04		
Tumor necrosis factor-α at 72 h of admission	1.14 [1.05–1.23]; < 0.01		1.13 [1.03–1.25]; 0.01

## P035 Traumatic brain injury: Could Motor Score–Pupils have a higher prognostic value than Glasgow Coma Scale?

### W Bahria, C Tlaies, F Sebeai, I Boussaid, M Miledi, I Sahnoun, M Boussen, NE Nouira

#### Mongi Slim Academic Hospital, Emergency Department, Tunis, Tunisia

*Critical Care* 2024, **28(Suppl 1):** P035

**Introduction:** The Glasgow Coma Scale (GCS) is a classic clinical tool used to classify traumatic brain injury (TBI) severity at the emergency department (ED). The motor component of the GCS appears to be the most powerful predictor of outcome. Recently, some studies suggested a new model by adding the pupil reactivity score to the motor component of GCS creating the Motor Score-Pupils (MS-P). We aimed to evaluate if MS-P could have a higher impact on the mortality rate than the Glasgow Coma Scale in TBI admitted to the ED.

**Methods:** This observational prospective and analytical cohort study was conducted over 3 years in an universal hospital including patients admitted to the ED for traumatic brain injury. MS-P was calculated at admission by subtracting 0, 1, 2 points of the better motor response evaluated using GCS when pupils were bilaterally responsive, only one responsive and bilaterally irresponsive respectively with a variation of − 1 to 6 points. The primary end-point was the mortality at the ED. ROC curves were used to identify variables predicting mortality.

**Results:** One hundred and fifty six patients were included in our study. The mean age was 39 years [15–88] with a sex ratio of 4.8. The mean GCS was 12.95 [3–15]. The mean motor response was 5.37 [1–6]. The mean MS-P was 5.02 [-1–6]. For the pupils reactivity: 140 (89%) patients had bilateral response, 4 (2.6%) had only one response and 12 (7.7%) had bilateral irresponsive. The mortality at the ED was shown in 12 patients (7.7%). The GCS and MS-P area under the curve for the mortality at the ED were respectively (*p* = 0.000; AUC = 0.883; 95% CI [0.810–0.957]) and (*p* = 0.000; AUC = 0.943; 95% CI [0.902–0.983]).

**Conclusions:** GCS and MS-P calculated on admission to the ED could both identify patients with traumatic brain injury at high risk of death. However, MS-P demonstrated useful discrimination and higher AUC value than the GCS for predicting mortality. This funding deserves more research in multicentre studies to be useful.

## P036 Treatment following intracranial pressure monitoring improves outcome but extends the length of stay: a retrospective analysis of a single center

### R Antolini^1^, G Perini^2^, F Violini^2^, A Raponi^3^, C Pacini^2^, F Santoni^2^, E Vitali^2^, A Salvucci Salice^4^, A Donati^5^, A Carsetti^5^

#### ^1^UNIVPM, Ancona, Italy, ^2^UNIVPM, Department of Biomedical Sciences and Public Health, Università Politecnica delle Marche, Ancona, Italy, ^3^UNIVPM, Emergency Medicine Residency Program, Università Politecnica delle Marche, Ancona, Italy, ^4^UNIVPM, Medicina E Chirurgia, Ancona, Italy, ^5^UNIVPM, Department of Biomedical Sciences and Public Health, Università Politecnica delle Marche, Ancona, Italy/Clinica di Anestesia e Rianimazione Generale, Respiratoria e del Trauma Maggiore, AOU delle Marche, Ancona, Italy

*Critical Care* 2024, **28(Suppl 1):** P036

**Introduction:** Traumatic brain injury (TBI) is the leading cause of mortality and morbidity in children and young adults in both developed and developing nations worldwide. The severity of the head trauma TBI is assessed using the GCS score and it is classified as: severe ≤ 8, moderate 9–13, mild ≥ 14. Treatments include: antiepileptic prophylaxis, management of intracranial hypertension in order to maintain cerebral perfusion pressure and ensuring adequate oxygen delivery. The use of intracranial pressure (ICP) monitoring has been postulated to be beneficial in patients with severe TBI, although studies investigating this hypothesis have reported conflicting results. The objective of this study is to compare the mortality of the patients treated following ICP monitoring.

**Methods:** We retrospectively analyzed all the patients admitted to our ICU between January 2018 and June 2023. We collected the following variables: gender, age, pre-intubation GCS, whether they underwent neurosurgical decompressive craniotomy, ICP monitoring, AEDs and EEG results, length of stay (LoS) and outcome. For statistical analysis Mann–Whitney-U test was used for comparing medians and Fisher’s test for group comparisons.

**Results:** We included 453 patients, 338 (74%) were male. Median age was 58 [37; 75] years and a median pre-intubation GCS of 8 [6; 13]. 165 (36%) patients have isolated TBI, 133 (29%) had ICP monitoring. The median days of LoS of the patients with ICP monitoring was higher than in the ones without ICP monitoring (14 [8; 22] vs. 8 [4; 13], *p* < 0.05) but there is a reduction in mortality with odds ratio of 2.11 (95% CI: 0.97, 5.12) [Table]. The same results are obtained by considering the population with GCS ≤ 8.

**Conclusions:** Our study highlights the effectiveness of ICP monitoring in TBI, demonstrating an improvement in clinical outcome despite an extended LoS and possible mortality reduction. These findings emphasize the importance of considering ICP monitoring as pivotal intervention especially in severe TBI management.

**Table (abstract P036):** Patient outcome: comparison between ICP monitoring and no ICP monitoring

	ICP Monitoring	No ICP Monitoring	*p* value
Discharged from ICU	123	264	< 0.05
Dead	9	41	

## P037 Hemoglobin levels, transfusions and outcomes after traumatic brain injury. Data from CENTER-TBI

### A Guglielmi^1^, F Graziano^2^, M Baggiani^3^, L Patrini^3^, A Di Cristofano^4^, E Ioppolo^5^, FS Taccone^5^, G Citerio^3^

#### ^1^University of Pavia-IRCCS Policlinico San Matteo, Pavia, Department of Clinical-Surgical Diagnostic and Paediatric Sciences, Unit of Anaesthesia and Intensive Care, Pavia, Italy, ^2^IRCCS Fondazione San Gerardo dei Tintori, NeuroIntensive Care Unit, Department Neuroscience, Monza, Italy, ^3^University of Pavia-IRCCS Policlinico San Matteo, Pavia, Anaesthesia and Intensive Care Department Fondazione Pavia, Italy, ^4^IRCCS Fondazione San Gerardo dei Tintori, School of Medicine and Surgery, University of Milano – Bicocca, Milan, Italy, ^5^Department of Intensive Care, Erasme Hospital, Université Libre de Bruxelles, Brussels, Belgium

*Critical Care* 2024, **28(Suppl 1):** P037

**Introduction:** Uncertainties remain in TBI patients about hemoglobin (Hb) thresholds and red blood cell (RBC) transfusion strategies. Our aim was to describe hemoglobin values and RBC transfusion in TBI admitted to ICU.

**Methods:** Secondary analysis of CENTER-TBI, a multicenter, prospective, observational study on a large cohort of TBI patients. We included all the patients admitted to ICU, with Hb data available at admission and during first week.

**Results:** 1385 TBI patients admitted to ICU had a hemoglobin value at baseline (mean ISS 32 (sd = 15). At admission the mean value of hemoglobin was of 12.9 (± 1.9) g/dL decreasing at the end of the first week in ICU, 9.8 (± 1.5) g/dL. Patients with Hb values lower than di 7.5 or 9.5 g/dL were 0.4% (n = 5) and 5.6% (n = 77) while at the end of the first week were 3.7% (n = 24) and 42.6% (n = 276). The median fluids balance over the first week in ICU was 1.08L (QI–QIII = − 0.09 to 3.70). 23% (318) underwent RBC transfusion, with 156 (49%) needing more than 1 unit of RBC. Median Hb values before transfusion were 8.40 g/dL (QI–Q3 = 7.7–9.1) with high variability amongst centers and countries with a range from 5.9 g/dL to 14.3 g/dL and 7.3 g/dL to 14.3 g/dL in centers and countries, respectively. No univocal transfusion policy was identified, with a prevalence of liberal strategy within countries (Figure). No differences in Hb values before transfusion were found between patients with poor and good neurological outcomes (8.44 g/dL, sd = 1.10, and 8.64 g/dL, sd = 1.46, *p* = 0.166). The same results were obtained when considering mortality at 6 months as an outcome.

**Conclusions:** A reduction of Hb values during first week of ICU admission were observed in our cohort. Hb less than 7.5 g/dL is uncommon in TBI patients and only a low percentage of patient need an RBC transfusion during ICU stay. Transfusion policy is heterogenous and seem not strictly following current guidelines indications. Although Hb values differs among patients, this seems to have not an impact on long term outcome.

**Figure (abstract P037):**
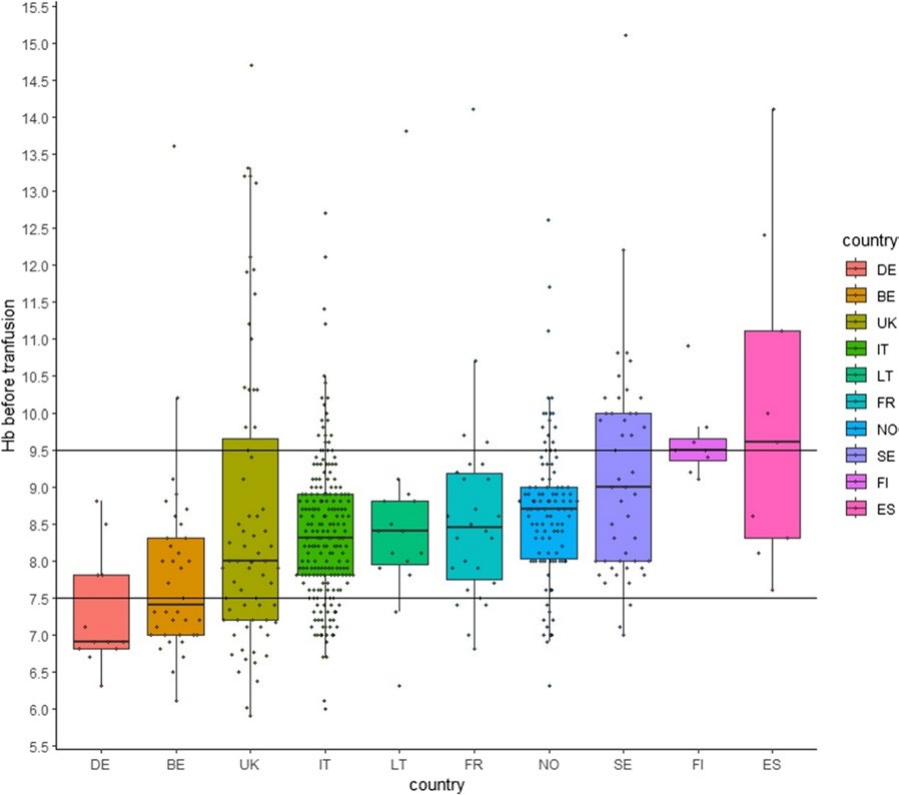
Box and whisker plot: values of Hb before transfusion in each country, considering centers with more than 15 observations. Median values are indicated.

## P038 Early extracranial surgery in early traumatic brain injury (TBI) and systemic insults

### M Baggiani^1^, F Graziano^1^, L Patrini^2^, E Ioppolo^3^, A Guglielmi^4^, E Picetti^5^, G Citerio^2^

#### ^1^Fondazione IRCCS San Gerardo dei Tintori, Neuroscience Department, NeuroIntensive Care Unit, Monza, Italy, ^2^University of Pavia, Department of Clinical-Surgical Diagnostic and Paediatric Sciences, Pavia, Italy, ^3^University of Milano - Bicocca, School of Medicine and Surgery, Milan, Italy, ^4^University of Pavia, Department of Clinical-Surgical Diagnostic and Paediatric Sciences, Unit of Anaesthesia and Intensive Care, Pavia, Italy, ^5^Parma University Hospital, Department of Anesthesia and Intensive Care, Parma, Italy

*Critical Care* 2024, **28(Suppl 1):** P038

**Introduction:** Our study aims to provide a comprehensive overview of early extracranial surgery in patients with traumatic brain injuries (TBIs) and its correlation with systemic insults (SIs), specifically hypotension and hypoxia.

**Methods:** We analyzed TBIs admitted to 66 intensive care units participating in the CENTER-TBI study. We focused on acute care needs and interventions during the ICU stay, with particular attention to extracranial surgery (ES) performed within the first 48 h. Utilizing a logistic regression model, we assessed potential predictors, including SIs.

**Results:** Out of 1695 patients, 258 (15.2%) underwent ES within the initial 48 h. These patients tended to be younger and had a higher total Injury Severity Scale than those without ES (Table). In a multivariable analysis:

- Hypotension alone and both SIs were identified as factors associated with an increased risk of undergoing extracranial surgery within 48 h (OR 2.6, 95% CI 1.6–4.1, *p* < 0.001; and OR 2.1, 95% CI 1.2–3.5, *p* = 0.005; respectively).

- Higher Abbreviated Injury Scale (AIS) scores (> 3) in specific extracranial body regions, such as the abdomen and pelvis (OR 2.4, 95% CI 1.7–3.5), extremities (OR = 5.9, 95% CI 4.3–8.3), and spine (OR 1.4, 95% CI 0.9–2.1), were also associated with an elevated risk.

- Evaluating GOSE score in patients undergoing ES, we have found both more moderate and disability (34.5% and 33.3% vs. 24.8% and 20.4%), but mortality at 6 months was lower (13.2% vs. 24.1%).

**Conclusions:** The need for surgical interventions increases with the severity of trauma and with Systemic Insults (SIs). This has an impact on clinical outcomes: ES do not increase mortality at 6 months but these patients are more susceptible to an increased disability, both moderate and severe. Identifying these populations at hospital admission could facilitate the healing process and identify their clinical needs.

**Table (abstract P038):** Patient characteristics

	Overall (1695)	Extracranial surgery (258)	No extracranial surgery (1437)	*p*
Age (mean (SD))	50.41 (19.34)	44.15 (17.98)	51.54 (19.37)	< 0.001
Road traffic accident	741 (45.2)	177 (69.7)	564 (40.7)	< 0.001
Total ISS (mean (SD))	33.61 (16.18)	45.05 (15.37)	31.56 (15.46)	< 0.001
Classification (%)—both	115 ( 6.8)	37 (14.3)	78 ( 5.4)	< 0.001
Hypotension	142 ( 8.4)	50 (19.4)	92 ( 6.4)	
Hypoxia	158 ( 9.3)	27 (10.5)	131 ( 9.1)	
Mortality % (6 months)	380 (22.4)	34 (13.2)	346 (24.1)	< 0.001

## P039 Study of a large cohort of traumatic spinal cord injury patients attended in intensive care unit during the last 4 years

### I Selva Armadans^1^, AF Jiménez Alfonso^2^, J Baena Caparrós^2^, M Baguena Martínez^2^

#### ^1^Hospital Universitari Germans Trias i Pujol, Intensive Care, Badalona, Spain, ^2^Hospital Universitari Vall d´Hebron, Intensive Care Unit, Barcelona, Spain

*Critical Care* 2024, **28(Suppl 1):** P039

**Introduction:** Traumatic spinal cord injury (TSCI) has a significant impact on mortality and morbidity, with a substantial burden on healthcare systems. This condition stands as a leading cause of disability, especially among young people. Our aim was to report the clinical characteristics of patients admitted with TSCI to the Neurotrauma and Rehabilitation Intensive Care Unit at Vall d’Hebron University Hospital, Barcelona (ICU-HVH).

**Methods:** A descriptive, cross-sectional, retrospective study was conducted on TSCI patients in the ICU-HVH, utilizing epidemiological, clinical, surgical, and treatment parameters. The clinical evolution of patients was assessed using the Glasgow Outcome Scale (GOS) at 3 and 6 months.

**Results:** 195 patients (84% males) were treated. Mean age was 51 ± 19 years [14–87], average length of stay was 9 ± 12 days [0–85]. Most common causes were: same level falls (30%), motor vehicle accidents (21%), sports accidents (16%) and, different level falls (13%). Most patients presented cervical spinal cord injuries (69%), of which 63% were incomplete; 30% had thoracic spinal cord injuries, of which 62% were complete (*p* < 0.005). 45% of cervical TSCI received corticoids. Fracture site stabilization was performed in 90% of patients, annual increase within the first 24 h, increased from 6% in 2019 to 57% in 2022. After 72 h a progressive decrease in surgeries was observed (54–15%; *p* < 0.001). At 6 months, the percentage of patients with good recovery increased to 27% (*p* < 0.001). Patients operated on within the first 24 h exhibited a 37% good recovery on the GOS at 3 months (*p* < 0.001). At 6 months, 13% patients walked without assistance, 3% with crutches or a walker, and 57% used a wheelchair.

**Conclusions:** There is an increasing trend in both the number and early timing of interventions. Early intervention in TSCI patients reflects a better prognosis at 3 months, resulting in lower rehabilitation therapy costs.

## P040 Examining the predictive effectiveness of ATLS, rTS in polytrauma patients and defining new parameters to be added to trauma scores

### S Hosaf^1^, O Ayvaz^1^, T Kayım^1^, E Karakoc^2^, B Yelken^2^

#### ^1^Eskisehir Osmangazı University, Anaesthesia and Reanimation, Eskisehir, Turkey, ^2^Eskisehir Osmangazı University, Intensive Care Unit, Eskisehir, Turkey

*Critical Care* 2024, **28(Suppl 1):** P040

**Introduction:** Many scoring systems have been evaluated in predicting mortality and prognosis of trauma patients, but their superiority and reliability over each other are still not sufficient. In this study, it was aimed to determine the parameters that could effect mortality and prognosis in order to determine the parameters that should be used for a new scoring system.

**Methods:** Polytrauma patients hospitalized in the ICU of a university hospital between June 2020 and June 2023 were retrospectively evaluated. Patients' files, archive records and hospital automation recording systems were examined in detail. Age, gender, chronic disease, trauma etiology, APACHE-2 and weighted revised trauma score, Glasgow coma scores (GCS) at admission to the ICU, ATLS 10 score, length of stay in the ICU, length of hospital stay, mortality rates were evaluated. Lactate levels for the first 5 days were evaluated. Mann Whitney U-Test was used for comparisons between groups, and chi-square or Fisher’s exact test was used for comparisons of categorical variables. Spearman’s correlation analysis was used to examine the relationship between continuous variables.

**Results:** A total of 4053 patients were treated in the ICU between the specified dates and a total of 96 patients with a diagnosis of polytrauma were included in the study. The median age of the polytrauma patients was 43 and 68.8% (n:66) of them were male. There was statistically significant difference between survivors and nonsurvivors in terms of APACHE-2, revised trauma score, GCS at admission to ICU, ATLS 10 score, lactate levels at 2, 3, 4 and 5 days. Presence of thoracic trauma and ATLS 10 score were correlated with length of stay in ICU.

**Conclusions: **As we reported that nonsurvivors’ mean GCS points were significantly lower than survivors’, we concluded that a score that designed to predict mortality of polytrauma patients should be mostly dominated by GCS. The predictability of the first 5 days lactate levels should be examined to use as a parameter of a mortality score.

## P041 Impact of E-FAST and massive transfusion in polytrauma approach

### AR Simões, M Sequeira, L Simões, L Linhares, E Trigo, P Martins

#### Centro Hospitalar e Universitário de Coimbra, Serviço Medicina Intensiva, Coimbra, Portugal

*Critical Care* 2024, **28(Suppl 1):** P041

**Introduction:** Trauma patients frequently suffer hemorrhagic shock and its rapid control impacts the patient’s outcome. Massive transfusion (MT) usually involves administering 10 or more units of whole blood or packed red blood cells (PRBCs) within 24 h. Extended Focused Assessment with Sonography for Trauma (E-FAST) is a point-of-care ultrasound easily performed in the emergency room (ER) to detect internal bleeding, enabling early treatment. To assess the outcome and efficacy of the Trauma Fast Track protocol (TFT) in trauma patients admitted to the intensive care unit (ICU), focusing on those with a positive E-FAST at the ER.

**Methods:** Retrospective study of severe trauma patients from TFT admitted in the ICU with a positive E-FAST at the ER in the trauma center of the center region of Portugal, from November 2020 to August 2023.

**Results:** 159 patients were admitted in TFT protocol. 31% (n = 50) were submitted to E-FAST and of those 28% had a positive E-FAST (n = 14) with a mean TRISS score of 52.15%. Road accidents accounted for 83% of major causes of lesion. 12 patients were included in this study. E-FAST revealed 10 cases of hemoperitoneum, 3 of pneumothorax and 1 of pleural effusion. 60% initiated the MT protocol with a median ratio of 4.25:0.42:1.75 PRBCs, plasma and platelets within 24 h. 83% were submitted to emergent surgery (80% exploratory laparotomy). The median time to emergent surgery was 1.97 h. Overall, in-hospital mortality was 42% (40% due to coagulation dysfunction).

**Conclusions:** E-FAST was performed in 31% of patients because TFT protocol only considers its implementation in unstable patients. An effective implementation of MT protocol demands a standardized approach, ongoing education and a continuous review of procedures. The analysis of the total volume of blood products revealed a disparity in relation to the recommended ratio of 1:1:1. This gap suggests the need for a review of procedures to ensure an approach aligned with the best practices and prevent coagulopathy.

## P043 D-dimer levels at the time of admission to hospital as predictor of outcome in trauma patients. A prospective observational study

### A Hazarika^1^, J Ahluwalia^2^, K Jain^1^, N Bhatia^1^, D Kumar^3^

#### ^1^Postgraduate Institute of Medical Education & Research, Department of Anaestheisa & Intensive care, Chandigarh, India, ^2^Postgraduate Institute of Medical Education & Research, Hematology, Chandigarh, India, ^3^Postgraduate Institute of Medical Education & Research, Orthopaedics, Chandigarh, India

*Critical Care* 2024, **28(Suppl 1):** P043

**Introduction:** Trauma causes a state of hypercoagulability, and its presence is common early in the injury course. D-dimer (DD) easily measured and considered a good screening tool of coagulation activation. Its increase amount in plasma is a reflection of the extent of hyper coagulopathy. Hence, in trauma, measuring DD levels may be helpful in providing useful prognostic information. So, our study tried to find whether DD levels at the time of admission can be a predictor to the outcome of patients.

**Methods:** This prospective observational study involved 205 adult patients of age group 18–60 years coming to trauma emergency within 24 h of injury and blood samples collected within this period. The primary outcome was to assess whether DD levels at the time of admission predicts outcome. Association of DD levels with injury severity score (ISS), with blunt or penetrating trauma, time from injury to admission (TIA), and to hospital stay were secondary outcomes. Value of DD > 250 ng/mL were considered elevated. For ease of statistical analysis, patients were divided into two groups—ISS < 16 and ISS ≥ 16. We also divided the patients on basis of TIA; within 6 h and more than 6 h of injury.

**Results:** The DD level were significantly high in patients who died then those who were discharged. [1316.28 (384.5, 3331.18) vs. 498.03 (140, 693), *p* = 0.041] (Figure). Blunt trauma victims had significant high levels than penetrating [1280 (565, 3377) vs. 162 (82, 526), *p* = 0.001]. ISS < 16, TIA < 6 h and hospital stay had lesser DD levels but of no significant difference. Plotting a ROC of D-dimer values, a cut-off value of 1793.35 was calculated (sensitivity-0.72; specificity-0.4) and on its basis hospital stay was compared. There was no statistically significant difference.

**Conclusions:** Our study found that high DD levels at admission among non-survivors. Similarly, blunt trauma had increased levels than penetrating trauma victims. Hence, DD values at admission can be a useful screening tool to predict outcome.

**Figure (abstract P043):**
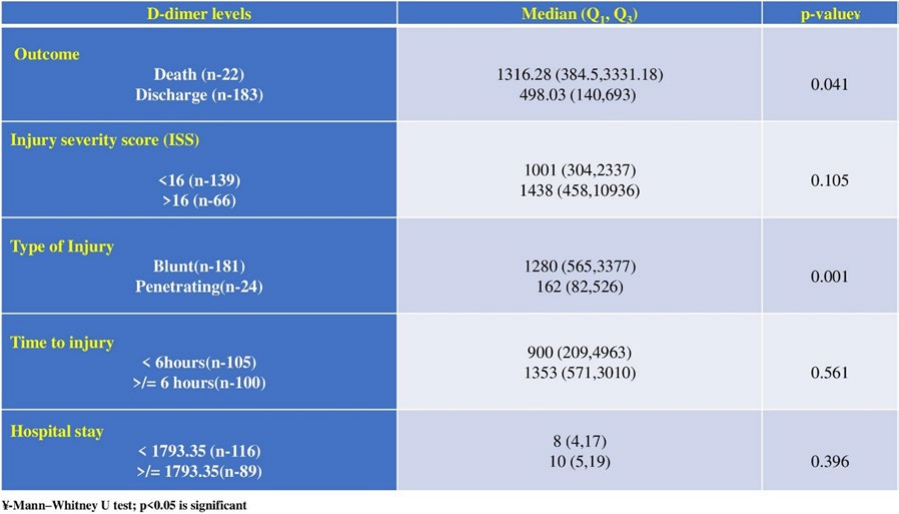
D-dimer levels among death and discharge patients, in relations to ISS, type of injury, time to injury and hospital stay.

## P044 FORMA-10 sub-analysis: a real-world, retrospective, observational study of fibrinogen concentrate in patients with trauma-related bleeding and fibrinogen deficiency

### F Stéphan^1^, L Gutermann^2^, S Bourget^3^, S Djabarouti^4^, J Berdugo^5^, Y Fardini^6^, P Clerson^6^, G Hébert^2^, C Belmokhtar^7^

#### ^1^Service de Réanimation Adultes, Hôpital Marie Lannelongue, Groupe Hospitalier Paris Saint Joseph, Le Plessis Robinson, France, ^2^Service Pharmacie et Stérilisation, Hôpital Marie Lannelongue, Groupe Hospitalier Paris Saint Joseph, Le Plessis Robinson, France, ^3^Service Pharmacie, Centre Hospitalier de Valence, Valence, France, ^4^Service Pharmacie, Groupe Hospitalier Sud, CHU de Bordeaux, Pessac, France, ^5^Service Pharmacie, Hôpital Saint-Joseph, Marseille, France, ^6^Soladis Clinical Studies, Roubaix, France, ^7^Octapharma France SAS, Boulogne-Billancourt, France

*Critical Care* 2024, **28(Suppl 1):** P044

**Introduction:** Low fibrinogen levels are characteristic of acquired fibrinogen deficiency, observed in trauma due to haemodilution and loss of clotting factors associated with major haemorrhage. This can be treated with fibrinogen sources, including human fibrinogen concentrate. The aim of this analysis was to extract the data from patients in the FORMA-10 study treated for non-surgical, trauma-related bleeding.

**Methods:** FORMA-10 [1] was a retrospective, observational, non-interventional study in patients with fibrinogen deficiency receiving fibrinogen concentrate (Fibryga®) from December 2017 to February 2020 in 6 study centres in France. The study evaluated real-world use of fibrinogen concentrate for the on-demand treatment of bleeding and in surgical prophylaxis. The primary endpoint was the indication and dose for fibrinogen concentrate use, with secondary endpoints including a measure of treatment success. For this sub-analysis, the FORMA-10 datasets were searched to extract available data regarding patients with trauma-related bleeding.

**Results:** FORMA-10 included 8 patients (11.3%) with non-surgical, trauma-related bleeding, comprising 7 males and 1 female, all aged ≥ 18 years. These patients had a mean age of 43.6 years and a mean weight of 73.5 kg. Mean baseline plasma fibrinogen levels (n = 7 reported) were 1.34 ± 0.54 g/L. The mean initial dose of Fibryga® administered for trauma-related bleeding was 2 ± 0.93 g or 28.65 ± 16.74 mg/kg, and mean total dose was 2.88 ± 1.25 g or 40.83 ± 20.01 mg/kg with a mean of 1.63 doses per patient. Treatment success (defined as complete cessation of bleeding or < 20% decrease in haemoglobin) for on-demand treatment for non-surgical bleeding episodes in these patients (n = 7) was 100%. No adverse drug reactions were documented.

**Conclusions:** This contemporary, real-life data set indicates that fibrinogen concentrate is successfully used in the management of non-surgical trauma-related bleeding in a European healthcare setting.

**Reference**

1. Stéphan F et al. J Clin Pharmacol. 2023;63:1186–96

## P045 An investigational four-factor prothrombin complex concentrate for vitamin K antagonist reversal: hemostatic efficacy and safety demonstrated with and without concurrent vitamin K therapy

### JN Goldstein^1^, G Simonian^2^, T Milling^3^, D Hinterberger^4^, M Gareis^4^, R Sarode^5^

#### ^1^Department of Emergency Medicine, Massachusetts General Hospital, Harvard Medical School, Boston, USA, ^2^Department of Surgery, Hackensack University Medical Center, Hackensack, USA, ^3^Seton Dell Medical School Stroke Institute, Dell Medical School, University of Texas at Austin, Austin, USA, ^4^Octapharma Pharmazeutika Produktionsges.m.b.H, Vienna, Austria, ^5^Division of Transfusion Medicine and Hemostasis, University of Texas Southwestern Medical Center, Dallas, USA

*Critical Care* 2024, **28(Suppl 1):** P045

**Introduction:** This LEX-209 subanalysis assessed efficacy and safety of four-factor prothrombin complex concentrate (4F-PCC), irrespective of concomitant vitamin K therapy. LEX-209 was a Phase 3 non-inferiority randomized control trial of an investigational 4F-PCC (Octaplex®, Octapharma), which demonstrated hemostatic non-inferiority to the FDA-approved control 4F-PCC (Kcentra®, CSL Behring) in rapid vitamin K antagonist (VKA) reversal before urgent surgery (*p* < 0.001).

**Methods:** In LEX-209, patients aged ≥ 18 years on VKA therapy with an international normalized ratio (INR) ≥ 2.0 and anticipating significant bleeding risk (≥ 50 mL) were enrolled across 24 United States and European hospitals. Randomized patients (1:1) received a single intravenous 4F-PCC infusion dosed by body weight and baseline INR. Concomitant vitamin K was administered unless VKA anticoagulation was required 24 h post-surgery. The primary endpoint, hemostatic non-inferiority, was evaluated by a blinded Independent Endpoint Adjudication Board using a surgeon-assessed objective scale. Additional endpoints included INR correction and safety.

**Results:** Baseline characteristics were comparable between investigational (n = 105) and control (n = 103) groups. Median 4F-PCC dose was 25 IU/kg (both groups). Most patients received concomitant vitamin K (75.2% investigational; 68.9% control). Investigational 4F-PCC demonstrated non-inferiority to control 4F-PCC, irrespective of vitamin K administration (Table). The proportion of patients achieving INR ≤ 1.5 at 30 min post-infusion was higher for investigational 4F-PCC compared to control, with similar safety profiles.

**Conclusions:** Investigational 4F-PCC was hemostatically non-inferior to control 4F-PCC for rapid VKA reversal in patients requiring urgent surgery with significant bleeding risk, regardless of concomitant vitamin K therapy. These findings support consideration of investigational 4F-PCC as a therapeutic option for surgical patients requiring rapid VKA reversal.

**Table (abstract P045):** Hemostatic efficacy and safety of 4F-PCC for urgent surgery requiring rapid VKA reversal with and without concomitant vitamin K therapy in the LEX-209 sub-analysis

4F-PCC	Investigational (n = 79)	Control (n = 71)	Investigational (n = 26)	Control (n = 32)
Concomitant vitamin K	Yes	Yes	No	No
Effective hemostasis, n (%)	73 (92.4%)	66 (93.0%)	26 (100%)	31 (96.9%)
Proportional efficacy difference (95% CI) *p* value	− 0.006 (− 0.103: 0.092); 0.002	–	0.031 (− 0.118: 0.181); 0.009	–
30 min post-infusion INR ≤ 1.5, n (%):	63 (79.7%)	54 (76.1%)	19 (73.1%)	20 (62.5%)
Proportional INR difference (95% CI)	0.037 (− 0.096: 0.170)	–	0.106 (− 0.139: 0.351)	–
Any TEAE	67 (84.8%)	59 (83.1%)	19 (73.1%)	21 (65.6%)
Any serious TEAE	11 (13.9%)	6 (8.5%)	2 (7.7%)	–

4F-PCC, four-factor prothrombin complex concentrate; CI confidence interval; INR, international normalized ratio; TEAE, treatment emergent adverse event; VKA vitamin K antagonist.

## P046 Impact of increasing concentration of direct oral anticoagulants and their impact on ROTEM variables: an experimental study

### L Sunnersjö, L Lindquist, J Undén, A Hillarp, U Schött, T Kander

#### Lund University, Medical Faculty, Lund, Sweden

*Critical Care* 2024, **28(Suppl 1):** P046

**Introduction:** Direct oral anticoagulants (DOAC) have become increasingly common and replaced warfarin in many instances. Despite DOACs being used in clinical practice for over 10 years there is still no readily available and accredited laboratory test to assess the anticoagulative effect of the different concentrations of different DOACs. Rotational thromboelastometry (ROTEM) is a widespread point-of-care instrument that has the potential to be used for this assessment. The primary aim was to investigate how the ROTEM variable clotting time (CT) in the EXTEM assay was affected by increasing concentrations of rivaroxaban. Secondary aims were to evaluate the impact of different concentrations of rivaroxaban, dabigatran and apixaban on the ROTEM variables CT, clot formation time (CFT) and α-angle.

**Methods:** Blood from twelve healthy volunteers for each DOAC was spiked to anticipated concentrations between 0 and 1000 µg/L. These different concentrations were analyzed in four different ROTEM assays (INTEM, EXTEM, FIBTEM and HEPTEM). CT, CFT and α-angle were measured in each assay. Chromogenic anti-IIa and anti-Xa assays were used to determine the actual concentration of the modified samples.

**Results:** The concentrations of all three DOACs were proportional to the CT of the four ROTEM assays (Figure). Rivaroxaban presented a significant increase in CT-EXTEM for the 200–1000 µg/L concentration compared to baseline. CFT and α-angle were affected mostly in supratherapeutic concentrations and primarily in the INTEM assay for all the tested DOACs.

**Conclusions:** We demonstrated that CT-EXTEM was affected by the increasing concentrations of rivaroxaban with a linear dose-dependent prolongation. CT in the other ROTEM assays was also affected in a similar manner while the impact on increasing DOAC concentrations on CFT and α-angle were not significant. In clinical practice, CT can be used as a surrogate marker for DOAC concentration.

**Figure (abstract P046):**
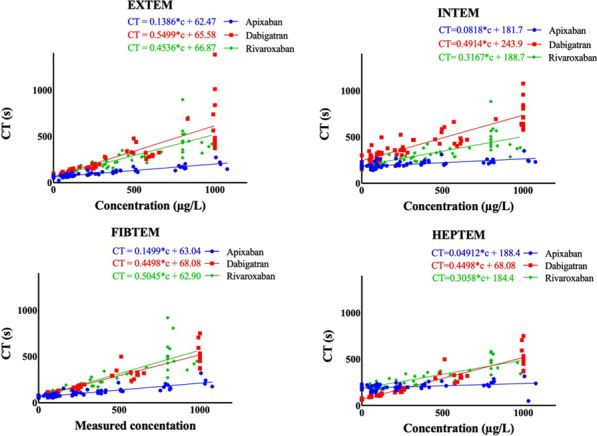
Linear regression of clotting time (CT) value depending on actual concentration of DOAC in EXTEM-, FIBTEM-, INTEM- and HEPTEM-assays. The estimated equations for the different slopes are given. The slopes between all DOACs in all diagrams were different from each other (*p* < 0.0001) except for the slopes of rivaroxaban and dabigatran in the EXTEM- and FIBTEM-assays.

## P047 Impact of red blood cell transfusion on physiologic parameters suggestive of anemia intolerance in critically ill children

### A Willems

#### HUDERF, Pediatric Intensive Care Unit, Brussels, Belgium

*Critical Care* 2024, **28(Suppl 1):** P047

**Introduction:** Some intensivists use physiological parameters in addition to the hemoglobin (Hb) concentration to give a red blood cell (RBC) transfusion. The reliability of such indicators remains to be determined. We used the dataset of P-OpTTICCA (NCT03871244) to study the impact of RBC transfusions on physiological parameters suggestive of anemia intolerance and the need for a RBC transfusion.

**Methods:** P-OpTTICCA was a pilot randomized controlled trial conducted in 4 PICUs in Canada, France, and the United Kingdom. Patients were allocated to an Hb threshold of 70 g/L (intervention arm) or to usual care (controls). Hb and five physiological parameters that can suggest anemia intolerance (PaO_2_, FiO_2_, PaO_2_/FiO_2_ ratio, ScvO_2_ and blood lactate) were collected prospectively.

**Results:** The data of the two RCT arms were merged, given that all demographic baseline data and outcomes were similar in the two arms. We included 120 patients (median age: 75.0 ± 72.2 months) of whom 68 (56.7%) were male and 46 (38.3%) under mechanical ventilation. In the cohort, 39 (32.5%) were transfused for a total of 89 RBC transfusions, and 81 (67.6%) were not. Both PaO_2_/FiO_2_ ratio and ScvO_2_ were higher before the 1st transfusion in the transfused group than during the entire PICU stay in the non-transfused patients (*p* = 0.003 and *p* = 0.0041). Blood lactate was similar. The change of physiological parameters measured before and after the 1st RBC transfusion in those 39 transfused participants, showed an increased Hb concentration (∆: + 22.0 ± 15.3 g/L, *p* < 0.001) and a decreased blood lactate[1.8 ± 2.5–1.5 ± 1.8 mmol/L, (*p* = 0.07)] even though it was > 2 mmol/L in only 11 out of the 37 transfused patients (29.7%).

**Conclusions:** In anemic critically ill children, a RBC transfusion increased the Hb concentration, decreased the blood lactate level, but did not change significantly pre- and post-transfusion values of four other potential physiological parameters suggestive of anemia intolerance.

## P048 Prevalence and significance of disseminated intravascular coagulation in COVID-19

### S Gando^1^, T Akiyama^2^

#### ^1^Hokkaido University Faculaty of Medicine, Anesthesiology and Critical Care Medicine, Sapporo, Japan, ^2^National Center for Global Health and Medicine, AMR Clinical Reference Center, Tokyo, Japan

*Critical Care* 2024, **28(Suppl 1):** P048

**Introduction:** Coagulopathy is a well-known complication of coronavirus disease 2019 (COVID-19). However, there are limited data on the prevalence and significance of disseminated intravascular coagulation (DIC) in COVID-19. We aimed to investigate the prevalence of DIC in COVID-19 and test the hypothesis that DIC is independently associated with a poor prognosis in patients with COVID-19.

**Methods:** A multicenter retrospective cohort study was conducted using large-scale COVID-19 registry data from hospitalized adult patients diagnosed with COVID-19. The patients were classified into DIC and non-DIC groups based on the diagnosis on admission (day 1) and the diagnosis on any of days 1, 4, 8, and 15.

**Results:** A total of 23,054 patients were included and were divided into 264 DIC patients and 22,790 non-DIC patients on day 1. A total of 1654 patients diagnosed with DIC on any of the days 1 to 15 were divided into 181 DIC patients and 1473 non-DIC patients. The prevalence of DIC on day 1 was 1.1%, which increased to 10.9% by day 15. The diagnosis of DIC on day 1 had moderate predictive performance for the development of multiple organ dysfunction syndrome (MODS) on day 4 and in-hospital death, and was independently associated with MODS and in-hospital death. The diagnosis of DIC on days 1 to 15, especially those diagnosed with DIC on days 8 and 15, was associated with a lower survival probability in comparison to patients without DIC and it also showed a significant association with in-hospital death in COVID-19 patients.

**Conclusions:** While it is not common, DIC, particularly when occurring later in the course, significantly contributes to the development of a poor prognosis in individuals with COVID-19.

## P049 Effects of therapeutic anticoagulation and high dose dexamethasone on mortality in patients with COVID-19 pneumonia admitted to the intensive care unit (ICU)

### K Cistera^1^, J Swartz^1^, J Alhashemi^2^

#### ^1^Lakeshore General Hospital, Respiratory Therapy, Pointe Claire, Canada, ^2^Lakeshore General Hospital, Anesthesiology, Pointe Claire, Canada

*Critical Care* 2024, **28(Suppl 1):** P049

**Introduction:** Therapeutic anticoagulation in critically ill patients with COVID-19 pneumonia remains controversial. This study evaluated the effects of therapeutic anticoagulation combined with high dose dexamethasone on mortality and length of stay among patients with COVID-19 pneumonia admitted to ICU.

**Methods:** A retrospective, observational, single-centre study in a community hospital ICU was performed from April 1, 2020 to June 30, 2021. Adults admitted to the ICU for COVID-19 pneumonia confirmed by polymerase chain reaction were included. Patients were excluded if they were pregnant or admitted to the ICU for reasons other than COVID-19 pneumonia. Participants received high-dose intravenous (IV) dexamethasone and therapeutic anticoagulation with IV heparin or low molecular weight heparin for a total of 28 days, or until ICU discharge, whichever came first. Outcomes included ICU mortality and length of stay (LOS). Chi-square and Kruskal–Wallis tests were used for mortality and LOS analyses. A stepwise binary logistic regression analysis used ICU mortality as the dependent variable while the independent variables were anticoagulation, bleeding, renal insufficiency, coronary artery disease, and heart failure. P to enter and remove were 0.15.

**Results:** Among the 146 patients studied, 100 received IV heparin to maintain aPTT at 70–100 s (group H), 22 received dalteparin 200 units/kg subcutaneously (SQ) daily (group L), and 24 received heparin 5000 units SQ twice daily or dalteparin 5000 units SQ daily (group P). Mortality was 40%, 23%, 58%, for groups H, L, and P, respectively (*p* = 0.045). Age (*p* = 0.021), bleeding (*p* < 0.001), and renal insufficiency (*p* = 0.063) were independent predictors of ICU mortality. The median (Q1, Q3) ICU length of stay was 10 (6, 17), 7 (4, 12), and 8 (4, 26) days for groups H, L, and P, respectively (*p* = 0.095).

**Conclusions:** Therapeutic dalteparin combined with high dose dexamethasone was associated with a mortality benefit in patients with COVID-19 pneumonia who were admitted to ICU.

## P050 Characterization of critically ill oncological patients before the pandemic: a nationwide analysis

### RL López^1^, PV Vargas^1^, JM Montes^1^, YB Bernal^2^, XA Aguilera^2^, ID Delgado^2^

#### ^1^Clínica Alemana de Santiago, Departamento Paciente Crítico, Santiago, Chile, ^2^Facultad de Medicina Clínica Alemana - Universidad del Desarrollo, Instiututo de Ciencias e Innovación en Medicina, Santiago, Chile

*Critical Care* 2024, **28(Suppl 1):** P050

**Introduction:** In Chile, neoplastic diseases have become the leading cause of death, surpassing cardiovascular diseases. In recent years, better clinical outcomes have been described in critically ill oncological patients, leading to their consideration for support in critical care units [1, 2]. However, in Chile, the proportion of critical patients with a history of cancer is unknown. This study aimed to describe the population of critically ill oncological patients in Chile and determine the proportion of critical patients who had cancer.

**Methods:** This study analyzed the 2019 Diagnosis Related Group (DRG) data, covering hospitalizations from November 1, 2018, to December 31, 2019. Patients were categorized by their presence in critical care units and oncological status per ICD-10. Comparisons between groups were conducted using Chi-squared and Student's T-tests via SPSS software. Key focus areas included hospital stay length and mortality rates. Ethical approval was granted based on data anonymity, negating the need for informed consent.

**Results:** In our study, we identified 727,979 patients, among whom 63,509 (8.7%) required care in critical care units. Of these critically ill patients, 13,238 (20.8%) were diagnosed with oncological conditions, predominantly solid neoplasms, accounting for 80% of these cases. Interestingly, a higher percentage of hospitalized oncological patients (15.9%) required critical care compared to their non-oncological counterparts (7.8%; *p* < 0.001). The detailed comparative analysis between oncological and non-oncological patient groups is comprehensively presented in the Table.

**Conclusions:** Nationally, one-fifth of critically ill patients were oncological, with significant variability among regions. Hospitalized oncological patients proportionally use more critical care units than non-oncological ones. Hospital stay and hospital mortality are higher in oncological patients.

**References**

1. Siegel RL et al. CA Cancer J Clin 2022;71:7–33.
2. Allemani C et al. Lancet. 2018;391:1023–1075.

**Table (abstract P050):** Comparative analysis of oncological versus non-oncological patients

Variable	Critically ill oncological patients (N = 13,238)	Critically ill non-oncological patients (N = 50,271)	Significance
Male, N (%)	7,061 (53.3)	27,896 (55.5)	< 0.001
Age, years, mean (SD)	60 (18)	59 (18)	< 0.001
Acute respiratory failure, N (%)	1,384 (10.5)	5,166 (10.3)	0.548
Sepsis, N (%)	1,893 (14.3)	2,896 (5.8)	< 0.001
Hospital length of stay, days, mean (SD)	14 (12)	11 (10)	< 0.001
ICU mortality, N (%)	882 (6.7)	2,231 (4.4)	< 0.001
Hospital mortality, N (%)	193 (14.6)	5,630 (11.2)	< 0.001

## P051 Intraoperative use of hydroxyethyl starch in patients undergoing major surgery is not associated with higher mortality: a propensity score matched analysis

### NS Schreiber^1^, AP Pichler^2^, MK Kolland^3^, DF Freidorfer^2^, ME Eichinger^2^, PZ Zoidl^2^, SF Fida^1^, LH Heuschneider^2^, ME Eichlseder^2^

#### ^1^Medical University of Graz, Department of Anesthesiology and Intensive Care Medicine 2, Graz, Austria, ^2^Medical University of Graz, Department of Anesthesiology and Intensive Care 1, Graz, Austria, ^3^Medical University of Graz, Division of Nephrology, Department of Internal Medicine, Graz, Austria

*Critical Care* 2024, **28(Suppl 1):** P051

**Introduction:** After the publication of three ICU-trials [1–3], which indicated that the utilization of hydroxyethyl starch (HES) adversely affects mortality in critically ill patients, the European Medicines Agency advised suspending the marketing authorization for HES. Despite this data has been questioned concerning generalizability regarding intraoperative use [4], and the fact that most trials focusing on goal-directed therapy in perioperative medicine have employed colloid boluses [5], there is only a small number of studies that have compared the use of HES and crystalloids in major surgery.

**Methods:** We analyzed the publicly available INSPIRE dataset [6] using propensity score matching to compare intraoperative use of HES versus HES-free volume therapy regarding hospital mortality in patients undergoing major surgery (> 2 h duration, postoperative ICU admission). The propensity score for intraoperative receipt of HES was calculated using multivariable logistic regression with covariates that affect both the receipt of HES and mortality, a 1:1 nearest-neighbor matching algorithm without replacement and a caliper width of 0.1.

**Results:** The matched cohort included 4458 patients. Hospital mortality in patients who intraoperatively received HES was 5.9% (132/2229), versus 5.1% (114/2229) in patients who only received crystalloids. The odds ratio for hospital mortality in patients treated with HES was 1.17 (95% CI 0.90–1.51, *p* = 0.24).

**Conclusions:** The intraoperative use of HES was not associated with significantly higher hospital mortality in a large propensity score matched dataset of patients undergoing major surgery.

**References**

1. Brunkhorst FM et al. N Engl J Med. 2008;358:125–139.
2. Perner A et al. N Engl J Med. 2012;367:124–134.
3. Myburgh JA et al. N Engl J Med. 2012;367:1901–1911.
4. Weiss R et al. Anesth Analg. 2018;127:1440–1444.
5. Zarbock A et al. Anesth Analg. 2022;134:683–685.
6. Lee H-C et al. INSPIRE, PhysioNet.

## P052 Correlation of corrected carotid artery flow time with left ventricular outflow tract velocity time integral after mini fluid challenge for assessment of fluid responsiveness

### K Putka^1^, S Kazūne^2^, I Būce-Šatoba^1^

#### ^1^Riga East University Hospital, Intensive Care Unit, Riga, Latvia, ^2^The Hospital of Traumatology and Orthopaedics, Anaesthesiology and Intensive Care, Riga, Latvia

*Critical Care* 2024, **28(Suppl 1):** P052

**Introduction:** Corrected carotid artery flow time (ccFT) assessed by ultrasound may be an attractive method for detecting fluid responsiveness in critically ill patients. However, it is not clear whether mini fluid challenge can induce changes in carotid flow measurements. In our study, we attempted to compare whether ccFT changes after mini fluid challenge and how it correlates with left ventricular outflow tract velocity time integral (LVOT VTI).

**Methods:** The study involved 20 adult patients. Only patients who met all inclusion criteria were included. Patients with acute pancreatitis and any type of gastrointestinal bleeding were selected. Carotid artery flow time was measured by ultrasonography. ccFT was calculated using Wodey’s formula. All measurements were taken before and after the mini fluid challenge with 100 mL crystalloid fluid. LVOT VTI was measured automatically. The fluid responsiveness was defined as an increase of 10% in LVOT VTI. All examinations were performed by a single physician in the intensive care unit.

**Results:** Among 20 patients, 12 (60%) were fluid responders. The average change in carotid corrected flow time after mini fluid challenge for fluid responders was 14 ms (± 12 ms). ccFT increase in 7 ms was defined as fluid responsiveness with sensitivity 80% and specificity 67%. The positive predicted value was 80.2% and the negative predicted value was 66.8%. The positive likelihood ratio was 2.40 (95% CI 0.46–13) and the negative likelihood ratio was 0.30 (95% CI 0.04–2.06). There was no significant difference between groups (pancreatitis vs gastrointestinal bleeding).

**Conclusions:** ccFT may be useful test to predict fluid responsiveness among critically ill patients using mini fluid challenge test.

## P053 Fluid resuscitation and acute kidney injury in the electric burn patient. Reconsidering initial resuscitation goals

### SJ Bernal Salazar^1^, L Nasiff Cabrales^1^, N Navarrete^2^

#### ^1^Universidad del Rosario, Burn ICU rotation, Bogota, Colombia, ^2^Hospital Simon Bolivar, Burn ICU Hospital Simón Bolívar, Bogota, Colombia

*Critical Care* 2024, **28(Suppl 1):** P053

**Introduction:** In burned patients the risk of acute kidney injury and mortality attributed to nonoptimal fluid resuscitation is well known [1, 2], nonetheless electrical burns patients are excluded from studies due to poor correlation between TBSA and injury severity. Volume management remain controversial this study aims to describe fluid resuscitation and describe an association between sub-optimal or excessive fluid administration and the incidence of early AKI (eAKI).

**Methods:** Retrospective cohort study. Registry data for adults admitted to Burn ICU after electrical injury, between 2007 and 2013 was analyzed. The primary outcome was eAKI. Incidence was modeled using multivariable logistic regression, including age, sex, TBSA, time to treat, and creatine phosphokinase maximum (CPKmax). CPK, fluid requirements, and urine output (UO) were recorded on days 0–6 of admission. The protocol targets were UO > 1.5 mL/kg/h in rhabdomyolysis or > 2 mL/kg/h if myoglobinuria were present.

**Results:** 456 patients were included, median TBSA was 5% (2–12%), 27 patients (5,9%) developed eAKI. There were no significant differences in age, sex, CPK, or CPKmax value. Volume infused in 0–2 days was lower in the eAKI group, with no significant differences regarding hourly resuscitation volume. The UO0 and UO1 (mL/hr) were significantly lower in the eAKI group (37.9 vs. 139.1 mL/hr, *p* < 0.001 and 162.0 vs. 187.6 mL/hr, *p* = 0.005 respectively). The UO0 was the only variable associated with the development of eAKI (OR 0.984, 95% CI 0.976–0.992).

**Conclusions:** Neither the time to treat nor the fluid volume of resuscitation are associated with the development of eAKI in electrical injuries. Crystalloid infusion guided by urinary output may result in a lower incidence of eAKI.

**References**

1. Dépret F et al. Burns. 2018;44:1887–94
2. Wu G et al. J Burn Care Res. 2017;38:271–282

## P054 Standardising central venous port selection to minimise extravasation risk: a complete audit cycle from a UK hospital

### RC Gill, N Manning, E Thomas, M Saba, J Patel, E McKemey, R Chauhan

#### University Hospitals Birmingham NHS Foundation Trust, Intensive Care Medicine, Birmingham, UK

*Critical Care* 2024, **28(Suppl 1):** P054

**Introduction:** Annually, 120,000 central venous access devices (CVADs) are inserted in the UK [1]. The incidence of immediate complications is estimated at 6.8% [1, 2]. Long term risks include infection, air embolism and extravasation [3]. Guidelines to reduce the latter are not yet widely established and incidence may be as high as 39% in adults [4]. This project aimed to improve the care of CVADs on Critical Care, thereby reducing the morbidity and mortality associated with extravasation.

**Methods:** Data were collected prospectively over 3 days in 2023 at an adult 100-bed intensive care unit. An audit in 2019 demonstrated sub-optimal port selection, and guidelines were introduced recommending that CVP should be transduced from the proximal and high-risk drugs given via the distal ports. To check adherence, every CVAD was considered a separate event. Data were collected regarding site, method of securing, medications administered and transducer location. Events were excluded if CVADs were inserted elsewhere or if data were incomplete. Any vasoactive drugs or those with high osmotic potential were considered high-risk.

**Results:** 127 CVADs were observed. 65% (n = 82) central venous catheters (CVCs) and 35% (n = 45) temporary dialysis catheters. Regarding CVCs, most (68%) were in the right internal jugular vein (IJV). 7% were transduced through the proximal port (6% in 2019) with 56% through the distal port (77% in 2019) and 13% not transduced. 28% (n = 23) had high-risk drugs administered through the proximal port (41% in 2019). 27% (n = 96) of high-risk drugs were infused proximally. 87% (n = 82) were sutured to the skin (21% not secured in 2019).

**Conclusions:** Despite some improvement with fewer CVADs transduced distally and fewer high-risk drugs proximally, port-choice remains suboptimal.

**References**

1. Wong AV et al. J Intensive Care. 2017;19:19–25
2. Lockwood J et al. Br J Hosp Med. 2019;80:114–9
3. Jarding EK et al. J Perianesth Nurs. 2021;36:328–33
4. Jones AM et al. The National Extravasation Information Service. 2020.

## P055 Evaluation of QT interval management in ICU: a two-cycle audit at Cambridge University Hospital, UK

### A Alsinbili, MG Kaya, P Bradley

#### Cambridge University Hospitals NHS Foundation Trust, Intensive Care Unit, Cambridge, UK

*Critical Care* 2024, **28(Suppl 1):** P055

**Introduction:** QT interval prolongation in ICU patients is common and poses significant risks, including potentially fatal arrhythmias. Clinical guidelines exist to address this specific risk however there are few guidelines for ICU [1]. This audit was conducted to assess and improve adherence to best medical practice for the detection and management of QTc prolongation in intensive care patients.

**Methods:** The audits were carried out at the adult general ICU in Cambridge University Hospitals during two separate weeks in April and July 2023. Employing Plan-Do-Study-Act cycles, the project measured the frequency and severity of QTc prolongation. Following the first cycle we increased staff awareness of QTc management guidelines [2]. Tip-sheet posters were created for staff along with an online account for checking medications. In the second cycle, data were again collected for prolonged QTc and also what management changes were made. Data analysis was conducted with statistical methods along with a narrative summary.

**Results:** In a two-cycle study of 98 patients, 18 of 40 in Cycle One and 40 of 58 in Cycle Two exhibited prolonged QTc. Key observations include a high prevalence of electrolyte disturbances, notably hypomagnesaemia (57%). and use of QTc prolonging medications (88%). Cycle Two showed increased female representation (40% up from 22%), improved QTc recognition (78%, *p* = 0.013), and better management with 95% follow-up ECGs (*p* = 0.001) and 68% (*p* = 0.023) medication reviews (100% in severe cases). Notably, patients with liver disease frequently showed non-severe QTc prolongation, unlike those with heart disease (50% severe prolongation).

**Conclusions:** This audit revealed a statistically significant improvement in the management and documentation of QT interval prolongation in ICU patients. The implementation of simple tools to raise awareness of QTc management can help clinicians and improve patient safety.

**References**

1. Priori SG et al. Europace. 2013;15:1389–406
2. Taylor MJ et al. BMJ Qual Saf. 2014;23:290–8

## P056 Locoregional anesthesia in thoracic trauma: ICU outcomes and implications

### A Barbosa Ribeiro^1^, G Sousa^2^, E Segura^2^, C Ferreira Santos^1^

#### ^1^Centro Hospitalar Tondela-Viseu, Intensive Care Medicine Department, Viseu, Portugal, ^2^Centro Hospitalar Tondela-Viseu, Anesthesiology Department, Viseu, Portugal

*Critical Care* 2024, **28(Suppl 1):** P056

**Introduction:** Pain management in thoracic trauma patients has, historically, relied heavily on systemic analgesic approaches, mostly opioids, associated with numerous adverse effects. Locoregional anesthesia/analgesia (LRAA), present a promising alternative by specifically targeting pain pathways at the injury site.

**Methods:** This study investigates the impact of LRAA on pain management and clinical outcomes in thoracic trauma patients within an ICU setting. It aims to assess the effectiveness of LRAA in reducing pain and its potential to influence ICU-related outcomes. We retrospectively analyzed 43 LRAA procedures performed on 33 patients. Fourteen procedures were excluded as they were unrelated to thoracic trauma.

**Results:** The median age of the patients was 65 years, with a notable male predominance (84%). LRAA techniques included thoracic epidural catheters, erector spinae blocks, and serratus plane blocks. Our study found that 50% of patients who received LRAA before invasive mechanical ventilation (IMV) avoided intubation (*p* < 0.05; odds ratio = 5.3). No severe complications were associated with the catheters, despite a median utilization time of 7 days. Patients who underwent LRAA before IMV had a significantly shorter ICU stay (median 9 vs. 13 days, *p* = 0.05). The study also noted a trend towards longer ventilation duration in patients who received LRAA before but still required IMV. In terms of mortality, there was one death in the ICU, but no 30-day post-discharge mortality. Regarding pain chronification, only 12.5% of patients experienced this issue post-discharge.

**Conclusions:** The study demonstrates the potential of LRAA in improving clinical outcomes for thoracic trauma patients in the ICU, particularly in reducing the need for IMV and shortening ICU stays. The findings suggest that early application of LRAA can be beneficial, although more research is needed to understand its full impact, especially on patients who still require IMV after LRAA.

**Acknowledgement: **The first and second authors contributed equally to this work.

## P057 Description of pharmacologic pain control using the analgesia nociception index monitor during the performance of noninvasive procedures in adult patients undergoing invasive mechanical ventilation in the intensive care unit

### C Catalán, A Cañas, J Tello, A Ulloa, C Díaz, J Contreras

#### Clinica Alemana, Intensive Care Unit, Santiago, Chile

*Critical Care* 2024, **28(Suppl 1):** P057

**Introduction:** This study examines ANI variations during noninvasive ICU procedures in mechanically ventilated patients. Primary objective: To analyze ANI levels before, during, and after procedures. Secondary objectives: to categorize analgesic responses and classify regimens for pain management in the absence of a gold standard for ICU pain assessment.

**Methods:** Prospective study in 2 ICUs, Jan–Sep 2023, aimed to detect 10-point ANI difference between different times. Data from 76 patients collected. Ethical approval granted without informed consent.

**Results:** Significant differences in ANIi were observed before, during and after the procedures. At rest, while CPOT indicated the presence of 1/85 measurements with moderate pain, ANIi identified 16 situations with pain (< 50), adequate analgesia in 25 and 44 (> 70) with analgesic overdose (Figure). During the procedures, ANIi values decreased significantly and returned to baseline values by time 2. The relationship between ANIi values and CPOT had significant differences, such as the use of analgesics, sedatives that showed significant correlations, such as the RASS sedation scale. Fentanyl did not show significant differences, but its association with other analgesics would allow a reduction in its dose.

**Conclusions:** ANI detects nociceptive stimuli during non-invasive procedures and reveals variability in analgesic dosing [1]. ANI makes visible situations of clinical importance, such as analgesic overdose, allowing for adjustment of analgesic doses based on patient response and avoidance of associated complications. On the other hand, by identifying a greater number of cases of inadequate analgesia, we can reduce the perpetuation of stressful situations that could have repercussions in delirium, post-ICU stress, and facilitate rehabilitation or weaning, among others.

**Reference**

1. Boselli E et al. J Clin Monit Comput 2016;30:977–984

**Figure (Abstract P057):**
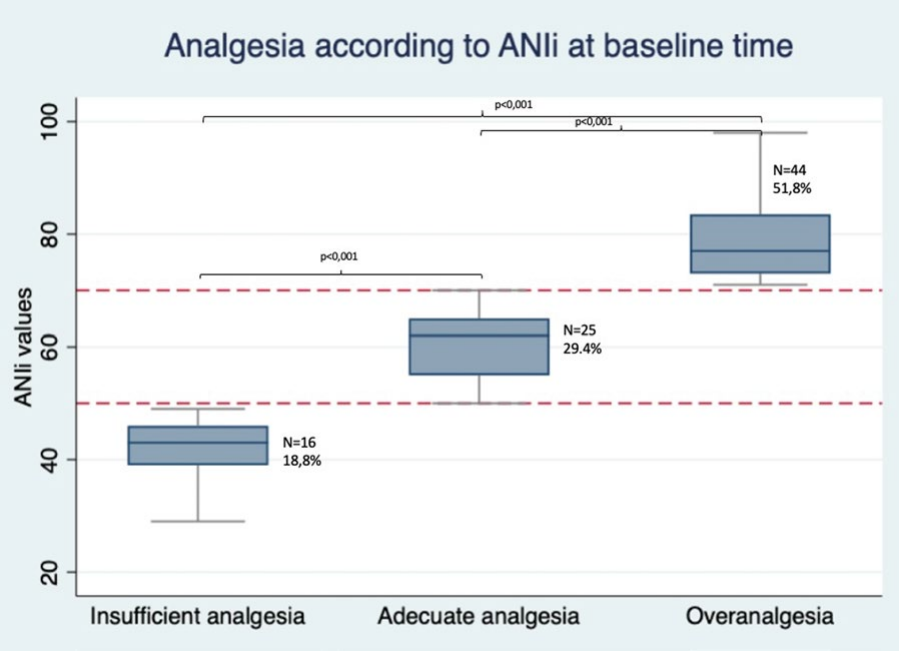
Classification according to ANIi values at baseline time as inadequate analgesia (< 50), adequate analgesia (50–70) or overanalgesia (> 70).

## P058 Effective pain management in chest trauma: should we go regional?

### D Martins Fernandes^1^, J Cabral^2^, JM Pereira^3^, S Fonseca^2^, N Gatta^3^, AR Teles^2^, JA Paiva^3^

#### ^1^Diana Fernandes, Intensive Care Department, Areosa, Portugal, ^2^Centro Hospitalar Universitário São João, Anesthesiology Department, Paranhos, Portugal, ^3^Centro Hospitalar Universitário São João, Intensive Care Department, Paranhos, Portugal

*Critical Care* 2024, **28(Suppl 1):** P058

**Introduction:** Chest trauma (CT) patients admitted to intensive care (ICU) experience significant pain, with psychological and physiological deleterious effects, risk of chronification and prolonged hospital stay. Opioids remain the first line and although regional analgesia (RA) is expanding, its role in the ICU is yet to be proven.

**Methods:** We aimed to describe and evaluate the role of RA for the critically ill CT patient. A retrospective cohort study of all CT patients admitted to a Portuguese tertiary’s hospital ICU was conducted. Patients’ demographics, analgesia modalities and opioid consumption measured in oral morphine milligram equivalents were assessed. RA was compared to standard analgesia (SA) and we considered T0 as the average time of RA initiation. Pain and opioid consumption were evaluated 24 h before T0 and five days later.

**Results:** 119 patients were included, mainly male (83%), a mean age of 50 years. Mean severity scores: SAPS 31(± 16), TRISS 36 (± 27). Mean ICU and hospital stay were 11 (± 11) and 29 (± 30) days respectively. Only 7% presented isolated CT and traumatic brain injury was the most frequent associated trauma (61%). 44% required invasive mechanical ventilation for a mean duration of 14 days, 16% developed respiratory nosocomial infection and 2.5% died in hospital. RA was used in 48.7% of CT patients. Thoracic epidural (TE) was the most common form of RA used (71%) followed by continuous erector spinae block (29%). RA reached a statistically significant reduction in opioid consumption (− 186 ± 1200 vs. − 103 ± 533, *p* = 0.028) compared to SA. Reduction reached statistical difference only for the TE subgroup. No significant differences were found between RA and SA and between both strategies of RA. Two minor TE associated complications were observed.

**Conclusions:** RA, mainly TE, is feasible, safe and offers a meaningful reduction in opioid consumption in CT patients despite no significant impact on other outcomes. Choice of analgesia strategy demands thoughtful patient-specific risk consideration.

## P059 Ultrasound-guided cervical plexus nerve block versus cervical plexus superficial nerve block, single injection, for postoperative haemodynamic and pain control in carotid endarterectomy operation

### DR Lončar Stojiljković^1^, P Stojilković^2^

#### ^1^Institute for Cardiovascular Surgery Dedinje, Belgrade, Anesthesia and ICU, Belgarde, Serbia, ^2^University of Banja Luka, Department of Pharmacology, Toxicology and Clinical Pharmacology, Banja Luka, Bosnia and Herzegovina

*Critical Care* 2024, **28(Suppl 1):** P059

**Introduction:** The carotid endarterectomy (CEA) as a treatment for extracranial carotid artery disease has been well established. Postoperative pain can increase the level of disturbed postoperative cerebral and cardiovascular autoregulation. Well-established perioperative pain control can improve cardiovascular stability and reduce the use of antihypertensive drugs. We studied how the efficacy of the cervical plexus superficial nerve block lateral, single injection vs ultrasound-guided cervical plexus nerve block application, as anaesthetic technique, influenced cardiovascular stability and pain control in the first 4 postoperative hours.

**Methods:** Fifty patients in the ASA III classification undergoing CEA under general anaesthesia) were randomised in two groups. Group I received ultrasound-guided superficial cervical plexus nerve block, while Group II received cervical plexus superficial nerve block, single injection without ultrasound. Postoperative evaluation was performed for 4 h: cardiovascular—mean blood pressure (MAP), heart rate (HR) and pulse pressure (PP) and level of pain (numerical pain scale, NPS) and postoperative complications such as myocardial infarction, or postoperative neurologic deficits. On admission to the intensive care unit, the patient received acetaminophen 1 g q6h and an additional painkiller on demand.

**Results:** There were no statistical significances among groups regarding MAP. HR was significantly lower in the non-US group. PP was increased to the third postoperative hour, while in the US group it was not significantly increased until the second hour and then it decreased. In the US-guided group a better pain control could be seen. The variation of pain in the non-US group was significantly different over time and in comparison with the US group.

**Conclusions:** In this study, there were no mortalities or neurological deficits. There was no difference in blood pressure changes. US-guided analgesia provided better pain control.

## P060 A comparison of droperidol 5 mg and 10 mg for acute agitation in the emergency department

### K Glass

#### Hennepin Healthcare, Pharmacy, Minneapolis, USA

*Critical Care* 2024, **28(Suppl 1):** P060

**Introduction:** Droperidol is commonly used in the emergency department (ED) to treat acute agitation. Previous studies have evaluated the safety and efficacy of droperidol compared with other medications for acute agitation. Intramuscular (IM) droperidol has been shown to be as effective as midazolam, ziprasidone, and lorazepam in reducing duration of violent behavior without significant differences in incidence of QTc prolongation [1–3]. The efficacy of IM droperidol at various doses (5 mg and 10 mg) was further investigated in this study.

**Methods:** This single-center, retrospective study examined adult patients in the ED who received either IM droperidol 5 mg or 10 mg as the first agent for treatment of acute agitation between 01 Jan. 2007 and 30 Jun. 2023. The primary objective was the need for rescue sedation within 1 h of initial droperidol dose. Secondary outcomes included the need for rescue sedation during the entire ED encounter, ED length of stay, and adverse events due to droperidol (hypoxemia, prolonged QTc, or cardiovascular events).

**Results:** In the preliminary analysis, 100 patients received IM droperidol 5 mg and 100 patients received IM droperidol 10 mg. In the droperidol 5 mg group, 26.6% of patients required rescue sedation within 1 h of receiving droperidol, compared with 35% of patients in the droperidol 10 mg group. The average ED length of stay was 7.9 h for droperidol 5 mg and 9 h for droperidol 10 mg.

**Conclusions:** Droperidol 10 mg for treatment of acute agitation may not result in less rescue sedation needed or shorter ED lengths of stay compared to droperidol 5 mg. However, this may be in part due to baseline levels of agitation resulting in provider preference when selecting droperidol doses.

**References**

1. Cole JB et al. West J Emerg Med. 2020;21:728–736
2. Isbister GK et al. Ann Emerg Med. 2010;56:392–401.e1.
3. Martel ML et al. Acad Emerg Med. 2021;28:421–434

## P061 Sevoflurane therapy using anaesthetic conserving device in life-threatening refractory bronchospasm in adults

### H Chandramohan^1^, S Dhanvijay^1^, A Kansal^1^, E Dela Pena^2^, E Sunico^2^, T Conanan^2^, P Dela Cruz^2^, M Vidanes^2^, F Ahmed Khan^1^

#### ^1^Ng Teng Fong General Hospital, Intensive Care Medicine, Singapore, Singapore, ^2^Ng Teng Fong General Hospital, Respiratory Therapist, Singapore, Singapore

*Critical Care* 2024, **28(Suppl 1):** P061

**Introduction:** Life-threatening asthma and chronic obstructive airway disease (COPD), which are refractory to standard medical therapy, has high mortality. Sevoflurane usage in ICU is difficult due to the need to operate anaesthetic machine. This is a single centre retrospective case series describing the usage and effectiveness of sevoflurane via anaesthetic conserving device (ACD) to treat refractory bronchospasm in adult medical intensive care unit (MICU).

**Methods:** Seven patients ranging from 34 to 62 years old was admitted to MICU for life-threatening status asthmaticus. A patient aged 67 years old was admitted to MICU post-cardiac arrest for hypercapnic respiratory failure due to COPD. They all required invasive mechanical ventilation with high dose inhaled beta-2 agonist, anti-muscarinic agent, paralytic agent, corticosteroid, and ketamine infusion. Sevoflurane was initiated using ACD in these patients as a rescue therapy in view of worsening intrinsic positive end expiratory pressure (iPEEP) and respiratory acidosis.

**Results:** The mean (range) PCO_2_ (mmHg) level before starting sevoflurane (via ACD), 6 h after, and at the end of therapy were 76.4 (57.4–93.8), 74.4 (50.4–95.3), and 56.6 (40.4–86). The mean (range) pH was 7.2 (7.1–7.3), 7.2 (7–7.4) and 7.4 (7–7.5). The mean (range) iPEEP (cmH_2_0) was 10.5 (5.6–16.2), 5.7 (2.9–9.3) and 7.3 (2.8–12.4). The mean (range) duration (hours) of sevoflurane was 63.5 (20–93). A patient had rapidly worsening ventilatory difficulties despite using sevoflurane hence, the patient was transferred to another centre for extra-corporeal membrane oxygenation (ECMO). No severe adverse effects apart from hypotension, which was manageable with noradrenaline infusion. All eight patients survived and were discharged from the hospital.

**Conclusions:** Sevoflurane via ACD can be safely used in ICU and can improve PCO_2_, pH, iPEEP and clinical condition of mechanically ventilated adults, who failed standard treatment for refractory bronchospasm.

## P062 Evaluation of the rate of continuation of antipsychotics started for delirium in ICU trauma patients

### J Spadgenske

#### Hennepine Healthcare, Pharmacy, Minneapolis, USA

*Critical Care* 2024, **28(Suppl 1):** P062

**Introduction:** A variety of medications are started in the ICU with the sole purpose of treating acute delirium (dl), which occurs in 21.7% of trauma patients [1]. Previous retrospective studies involving mixed ICU populations have found continuation rates ranging from 21 to 61% at hospital discharge for antipsychotics (AP) started in the ICU for dl [2–3]. Admission to the surgical ICU was cited as a risk factor for continuation of AP though no further descriptions of AP continuation have been described [3]. The continuation rate of AP was evaluated in a level 1 trauma academic medical center.

**Methods:** This single-center, retrospective study examined adult surgical ICU patients admitted for trauma from 01 May 2020 to 31 May 2023. Patients were excluded from the study if they had a history of select psychiatric disorders or were in the ICU for less than 48 h. Prior AP use was noted, and escalation was described as new AP use. The rates of dl and agitation were recorded to assess the indication for AP initiation. Of secondary interest, the rate of clonidine use for dexmedetomidine withdrawal was described.

**Results:** Preliminary results show of the 226 patients included, 59% were started on AP. Of these 133 patients, 71% were continued on AP at ICU discharge and 55% were continued at hospital discharge. Clonidine was initiated in 28 patients and continued in 10 patients at hospital discharge. Of patients on AP, 46% were discharged to acute rehabilitation, 35% to home, and 18% to long term care. At baseline, 6.6% of the population were on AP on admission.

**Conclusions:** Use of AP was common in the trauma ICU population. The rate of continuation at ICU and hospital discharge was similar to previous studies and warrants further studies to investigate the appropriateness of AP at each transition of care.

**References**

1. Marquetand J et al. Eur J Trauma Emerg Surg. 2022; 48:1017–1024
2. Dixit D et al. Am J Health Syst Pharm. 2021;78:1385–1394
3. Marshall J et al. J Crit Care. 2016;33:119–124

## P063 Population pharmacodynamics to study hemodynamic and sedative effects of clonidine in mechanically ventilated adult patients

### H van den Oever^1^, M Cloesmeijer^2^, M Zeeman^3^, M Arbouw^4^

#### ^1^Deventer Hospital, Intensive Care, Deventer, Netherlands, ^2^Amsterdam UMC location AMC, University of Amsterdam, Department of Hospital Pharmacy – Clinical Pharmacology, Amsterdam, Netherlands, ^3^Deventer Hospital, Clinical Geriatrics, Deventer, Netherlands, ^4^Deventer Hospital, Hospital Pharmacy, Deventer, Netherlands

*Critical Care* 2024, **28(Suppl 1):** P063

**Introduction:** The objective of this study was to describe the effects of clonidine on heart rate (HR), mean arterial blood pressure (MAP) and sedation.

**Methods:** In four cohorts of eight adult mechanically ventilated patients, we added clonidine to standard sedation in doses of 0, 600, 1200, and 1800 µg/24 h. HR, MAP, and sedation score (RASS) were measured 8-hourly; plasma was sampled to determine clonidine concentrations. Individual clonidine concentrations were predicted using a pharmacokinetic model [1], to serve as input for the pharmacodynamic models. NONMEM was used to derive linear effect models for HR and MAP and logistic models for sedation. The models were evaluated and validated by goodness-of-fit plots, bootstrapping and visual predictive checks.

**Results:** For the models of HR and MAP, 783 measurements were combined with predicted clonidine concentrations. HR was described by a linear effect model with a baseline of 90.6 bpm (relative standard error (RSE) 2%), clonidine concentrations. and a slope of − 3.30 bpm per µg/L clonidine (RSE 10%). MAP was described by a linear effect model with a baseline of 76.9 mmHg (RSE 2%), and a slope of − 0.887 mmHg per µg/L (RSE 42%). After including inter-individual variability on the baselines, the objective function values for HR and MAP decreased by 300 and 151 points. Panels A and B on the Figure display the measured HR and MAP against the 275 measured clonidine concentrations. For the sedation model, 773 measurements were available. The probability of deep sedation (RASS ≤ − 4) was described by a logit model with a baseline logit of 2.23 (RSE 25%), a slope for clonidine of − 0.383 (RSE 17%), a midazolam effect of − 2.48 (RSE 7%) and a propofol effect of − 1.54 (RSE 13%; Figure panel C).

**Conclusions:** In sedated, mechanically ventilated patients, the addition of clonidine was associated with dose-dependent reductions in HR and MAP. Clonidine alone and in combination with other sedatives was associated with a dose-dependent increase in the probability of deep sedation.

**Reference**

1. Cloesmeijer ME et al. Br J Clin Pharmacol 2020;86:1620–1631

**Figure (abstract P063):**
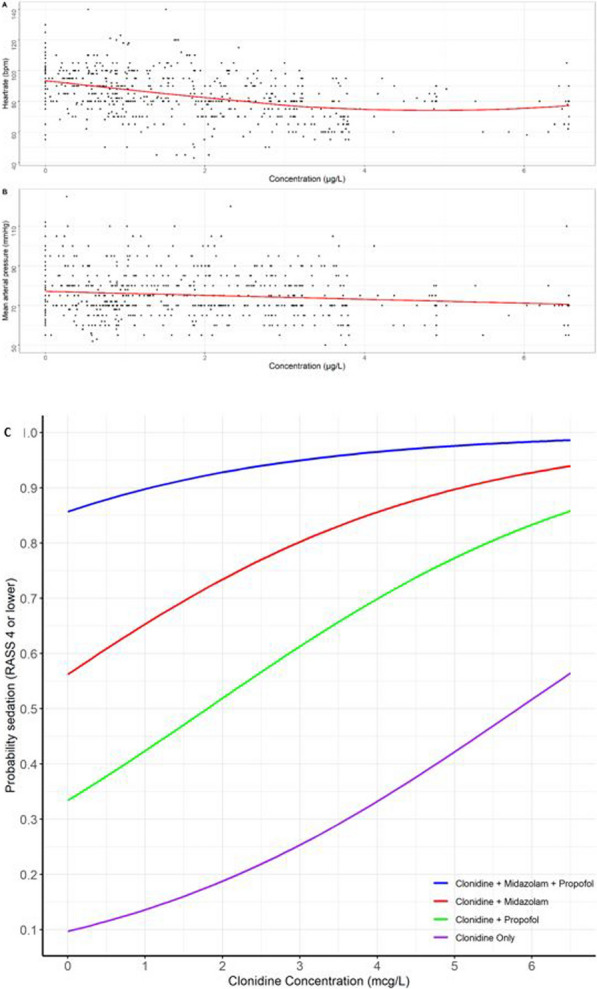
Directly measured clonidine concentrations (n = 275) versus **A** heart rate, and **B** mean arterial pressure. The red lines represent the local regression smooth lines (not linear regression curves).** C** Probability curve for induced deep sedation with clonidine only, and clonidine with midazolam, propofol, or a combination.

## P064 Cardioprotective effect of dexmedetomidine in patients with ischemic heart disease in non-cardiac surgery

### ME Kavlak^1^, Z Sungur^2^, M Orhan Sungur^2^, M Savran Karadeniz^2^, AK Bilge^3^, NM Senturk^4^, E Camcı^2^, M Tugrul^5^

#### ^1^Üsküdar University School of Medicine, Intensive Care Unit, Istanbul, Turkey, ^2^Istanbul University School of Medicine, Department of Anesthesiology and Reanimation, Istanbul, Turkey, ^3^Istanbul University School of Medicine, Department of Cardiology, Istanbul, Turkey, ^4^Acıbadem University School of Medicine, Department of Anesthesiology and Reanimation, Istanbul, Turkey, ^5^Vadi Liv Hospital, Department of Anesthesiology and Reanimation, Istanbul, Turkey

*Critical Care* 2024, **28(Suppl 1):** P064

**Introduction:** It is known that dexmedetomidine (Dx) reduces postoperative troponin-I (Tn-I) levels and diminishes ischemia–reperfusion injury in cardiopulmonary bypass [1]. We aimed to investigate the effect of Dx on myocardial ischemia in patients with ischemic heart disease (IHD) in non-cardiac surgery.

**Methods:** Patients with IHD who will be operated for peripheral vascular surgery were included in the study. They were allocated into 2 groups: the Dx group (GDx) and the midazolam (Md) group (GMd). In both groups, standard lumbar epidural anesthesia was performed. For sedation, Dx (0.6 mcg/kg/h) was used in GDx, whereas Md (0.025–0.05 mg/kg/h) was used in GMd during the operation. Patients’ characteristics, intraoperative hemodynamic parameters, Tn-I levels (preop, postop 8th, 24th, and 48th h), and ECG changes were recorded.

**Results:** Patients’ characteristics and preoperative Tn-I levels in GDx (n = 23) and GMd (n = 24) were similar. During the operation, the Ramsey Scale in GDx (2 ± 0.3) was significantly lower than in GMd (3 ± 0.3) (*p* < 0.001). Also, heart rate and systolic and diastolic blood pressure levels in GDx were significantly lower than in GMd (for all *p* < 0.001). In the first 24 h of the postoperative period, the numbers of ischemic episodes in both groups were similar (GDx:4 and GMd:8, *p* = 0.052). However, the duration of ischemic episodes in GDx (2.4 ± 0.9 min) was significantly shorter than in GMd (5.4 ± 1.2 min) (*p* < 0.001). Tn-I levels at the postoperative 8th, 24th, and 48th hours in GDx were significantly lower than in GMd (*p* values were between 0.05 and 0.01). Myocardial infarction was not determined in either group at any time.

**Conclusions:** Dx is a reliable sedative in this dosage in patients with IHD undergoing non-cardiac surgery. Although it can cause hypotension during surgery, it may have a cardioprotective effect by reducing afterload and heart rate when compared to Md.

**Reference**

1. Zhang GR et al. Eur Rev Med Pharmcol Sci. 2021;25:7409–17

## P065 Post-infarction ventricular septal defect: surgical repair and percutaneous closure with ASD occluder: single-center experience

### DV Pevzner^1^, EA Avetisyan^1^, EV Merkulov^2^, IA Merkulova^1^, AK Alieva^1^, IN Zharovin^1^, PF Kushnir^1^, I Ziuriaev^1^, LO Dulaev^1^, AG Osiev^3^

#### ^1^Federal State Budgetary Institution National Medical Research Centre of Cardiology Named After Academician E.I.Chazov of the Ministry of Health of the Russian Federation, Intensive Care Unit of the Emergency Cardiology Department, Moscow, Russian Federation, ^2^Federal State Budgetary Institution National Medical Research Centre of Cardiology Named After Academician E.I.Chazov of the Ministry of Health of the Russian Federation, Department of Endovascular Diagnostics And Treatment, Moscow, Russian Federation, ^3^Clinical and Diagnostic Center MEDSI, Endovascular Department, Moscow, Russian Federation

*Critical Care* 2024, **28(Suppl 1):** P065

**Introduction:** Early mortality in post-infarction ventricular septal defect (PIVSD) remains high and reaches 95% if untreated. Surgical repair (SR) is a standard of care, but in up to 50%patients open-heart surgery is associated with high risk, highlighting the need in an alternative promising method such as percutaneous closure (PC).The evidence on timing, operational technique and intensive care approach is still lacking.

**Methods:** This prospective, open-label, non-randomized study included patients admitted to a tertiary care center with the diagnosis of PIVSD and undergoing invasive procedures. Enrolled patients underwent either SR with patch, PC with atrial septal defect occlusion device or both methods in series, depending on decisions made by a dedicated heart team. In-hospital outcomes were assessed.

**Results:** From April 2019 to July 2023 43 patients were admitted with PIVSD, 9.3% (n = 4/43) did not underwent any procedure and were excluded from the analysis. Among 39 patients included in the analysis: 28.2% (n = 11/39) underwent SR, 66.7% (n = 26/39) underwent PC, 5.1 (n = 2/39) underwent combined stepwise approach (SR + PC). The median age was 67.7 ± 13 years. Cardiogenic shock (C–E by SCAI) at the procedure day was in 9.1% in the SR arm, 80.8% in the PC arm and 0% in the SR + PC arm. The median time from infarction to procedure was 34 days (interquartile range (IQR) 33–54) and 9 days (IQR 4.5–21.5) in the SR and PC arms, respectively (*p* < 0.001). IABP was implanted in 43.6% and VA ECMO in 20.5%. Overall in-hospital mortality was 43.6% (27.3% in the SR arm, 53.8% in the PC arm, 0% in the SR + PC arm). In patients received occluding device implantation success rate was 96% (n = 27/28). Fatal complications of occluder implantation occurred in 7.1% (n = 2/28), both were due to cardiac tamponade.

**Conclusions:** PC with atrial septal defect occlusion devices can be an effective and safe alternative to SR for patients with early deterioration. Early percutaneous closure can be considered in high-risk patients to improve outcomes.

## P066 The predictive value of the simplified CAMI score in fibrinolysis failure in patients admitted to the emergency room for myocardial infarction with elevated ST segment (STEMI)

### Y Walha^1^, W Bahria^1^, K Jmai^1^, H Ouerghi^1^, J Sebai^1^, M Bouraoui^1^, F Azaiez^2^, NE Nouira^1^

#### ^1^Mongi Slim Academic Hospital, Emergency Department, Tunis, Tunisia, ^2^Mongi Slim Academic Hospital, Cardiology Department, Tunis, Tunisia

*Critical Care* 2024, **28(Suppl 1):** P066

**Introduction:** STEMI is a medical emergency that requires immediate reperfusion. The CAMI score (China Acute Myocardial Infarction) has proven its predictive value for mortality as well as major cardiovascular events, in Chinese patients and subsequently in other populations presenting with STEMI but has never been evaluated in the prediction of fibrinolysis failure. The aim of our study was to determine the predictive value of CAMI score in fibrinolysis failure.

**Methods:** This was a prospective, observational and prognostic study conducted from January 2022 until May 2023, including patients who received fibrinolytic treatment with alteplase or tenecteplase for STEMI in the emergency room. The CAMI score, calculated for all patients, was simplified by eliminating the biological parameters (white blood cells and creatinine) to adapt it to the urgency of decision between thrombolysis and primary angioplasty. The score varies from 0 to 181.

**Results:** During the study period, 54 patients received fibrinolysis among 122 patients admitted to the emergency room with STEMI. The mean age was 60 ± 13 years. The sex ratio was 2.6; among these patients, 16.7% had known coronary artery disease of which 77.7% had previously undergone primary angioplasty, lysis failure was observed in 46.3% of patients, four patients presented with cardiogenic shock and three patients died in the emergency room. The predictive fibrinolysis failure value of the CAMI score was statistically tested and the results were significant (*p* = 0.004), as well as by analyzing the ROC curve (*p* = 0.004; air under the curve = 0.73; CI [0.59–0.86]). The cut-off value was 47 sensitivity and specificity were 96% and 71%, respectively.

**Conclusions:** The simplified CAMI score can predict fibrinolysis failure, it remains to be tested on a larger sample.

## P067 Evaluation of the HEAR score to exclude the hypothesis of acute coronary syndrome in patients consulting the emergency department for chest pain

### Y Walha^1^, I Boussaid^1^, W Demni^1^, R Haggi^1^, M Rebhi^1^, F Azaiez^2^, E Lagha^2^, M Boussen^1^, NE Nouira^1^

#### ^1^Mongi Slim Academic Hospital, Emergency Department, Tunis, Tunisia, ^2^Mongi Slim Academic Hospital, Cardiology Department, Tunis, Tunisia

*Critical Care* 2024, **28(Suppl 1):** P067

**Introduction:** Chest pain is one of the most common presentations to the emergency department (ED), the main diagnosis representing a short-term life-threatening emergency is acute coronary syndrome. Several studies have concluded that the HEAR score would allow, if it is ≤ 2, to exclude the hypothesis of acute coronary syndrome (ACS) with a low risk of false negatives. The main objective of our study was to evaluate the performance of the HEAR score in patients consulting for acute chest pain in the ED.

**Methods:** This was a prospective, descriptive study. Done over a period of seven months (from June 2022 until January 2023), carried out at the emergency room service. We included patients consulting for non-traumatic chest pain and whose clinical, biological and electrocardiographic data were complete. The HEAR score was calculated. ACS was retained on the diagnostic criteria based on the European society of cardiology recommendations and after consultation with the cardiologist.

**Results:** We included 416 patients. The mean age was 56 ± 14 years with a sex ratio of 1.88. Among these patients 27.5% had known coronary artery disease. Only 15.3% benefited from pre-hospital transport. The diagnosis of ACS was retained in 58.6% of patients. The HEAR score was ≤ 2 for 9.7% of patients, 96.8% of them had a troponin level lower than 3 times the normal value. The negative predictive value for ACS in patients with a low risk HEAR score was 94.5. By analyzing the ROC curve (*p* < 0.01; air under the curve = 0.83; CI [0.79–0.87]). The cut-off value was 1.5 sensitivity and specificity were 99.1% and 78.3%, respectively.

**Conclusions:** The HEAR Score is an interesting tool for daily practice which could allow a withdrawal from emergency departments by returning patients at low risk of ACS to home earlier.

## P068 Anesthesia management for high-risk patients with critical aortic stenosis. TAVI life salvage procedure

### A Kuanyshbek^1^, S Tulegenov^1^, T Kapyshev^1^, T Lesbekov^2^, D Seidanov^1^, D Zhuparkhan^1^

#### ^1^National Research Cardiac Surgery Center, CICU, Astana, Kazakhstan, ^2^National Research Cardiac Surgery Center, Cardiac Surgery, Astana, Kazakhstan

*Critical Care* 2024, **28(Suppl 1):** P068

**Introduction:** Aortic stenosis is one of the most common heart valve pathologies and its prevalence increases with age. It used to be associated with catastrophically high perioperative mortality, with the risk being significantly increased by the presence of coexisting heart failure and cardiac arrhythmias, whereas newer procedures such as transaortic valve replacement have reduced mortality rates.

**Methods:** Prospective observational study. Patients over 18 years of age with critical aortic stenosis who underwent a TAVI procedure. All patients signed a consent form for data collection. Standard monitoring: invasive BP, ECG. Pulse oximetry, measurement of CO_2_, ABG.

**Results:** In period January 2023–November 2023, 193 TAVI procedures were performed. 11 hybrid type of surgery (multy valves). Anaesthesia management in combination with local anesthesia (mean dosage)—sedation IV propofol infusion 4.2 ± 1.5 mg/kg/hour. Analgesia fentanyl 2.65 ± 0.8 mcg/kg/hour. 98.7% of patients had spontaneous breathing. 1.3% patients were intubated. Inotropic and vasopressor support was required in 16.7% of patients. LOS in the intensive care unit 5.79 h. Main complications of the procedure. Arrhythmia 37. 5% atrial fibrillation, ventricular extrasystole 12%, ventricular fibrillation − 2.3%. Bleeding from the femoral artery (puncture site) 7.3%. TIA—9.35%. The most severe cases were on ECMO support—3 cases.

**Conclusions:** Anaesthetic management may mistakenly appear simple. However, it requires utmost attention and comprehensive intraoperative monitoring. In case of worsening of the condition, emergency airway management and decision making requires expert knowledge and skills. TAVI is a life-saving procedure that has a positive impact on the outcomes of patients with advanced heart failure. IV anaesthesia makes it possible to maintain spontaneous breathing and shorten the stay in the intensive care unit.

**Acknowledgement:** Science Committee of the Ministry of Science and Higher Education of the Republic of Kazakhstan Grant # AP19677596.

## P069 Implementation of ERAS program in cardiac surgery: preliminary results of an ERAS certified centre

### D Carel^1^, M Verdugo-Marchese^2^, MZ Gunga^2^, A Nowacka^2^, V Melly^2^, C Botteau^1^, M Kirsch^2^, V Rancati^3^, Z Ltaief^1^

#### ^1^CHUV, ICU, Lausanne, Switzerland, ^2^CHUV, Cardiac Surgery, Lausanne, Switzerland, ^3^CHUV, Anaesthesia, Lausanne, Switzerland

*Critical Care* 2024, **28(Suppl 1):** P069

**Introduction:** In the dynamic landscape of cardiac surgery (CS), the application of Enhanced Recovery After Surgery for Critical Care (ERAS) is pivotal. This transdisciplinary, multimodal initiative aims to reduce complications, postoperative stay, and costs, prioritizing patient well-being. Building upon the success in cardiac surgery, we extended ERAS-CS to our intensive care units (ICUs) at Lausanne University Hospital, Switzerland.

**Methods:** Our approach faced specific challenges in the critical care setting. Early extubation (in the first 6 h) and early mobilization, defined as sitting in a chair for the first post-extubation meal, also implementing multimodal analgesia (locoregional anesthesia) with early daily opioid cessation assessment. We have also lowered our thoracic chest tube removal thresholds. Here, we present the preliminary results on ICU outcomes of the above measures in the first 50 ERAS patients received as part of the quality control for the certification process.

**Results:** 86% versus 88% of patients were extubated in the two groups (NS). Early mobilization on Day 0 and Day 1 reached 83% in ERAS versus 60% before (*p* = 0.05). Opioid use at day three was noted in 30% of ERAS patients versus 50% before (*p* = 0.04) with the same value of mean, median and maximum pain level assessed by visual analogue scale during the first three days. Constipation and respiratory complications were reduced respectively to 22 and 13% in ERAS patients versus 52 and 36% before (*p* < 0.05). Also, delirium was lower in ERAS group 4 versus 12% (NS).

**Conclusions:** Our ERAS strategy combining early extubation, mobilization and chest tube removal with multimodal analgesia yields promising results. This will have to be confronted with the increasing volume of ERAS patients and the risk of reduced compliance, which will be our future challenge.

## P070 Association of hemodynamic variables in the first 4 h after intensive care unit admission and the development of cardiac surgery-associated acute kidney injury within 72 h after cardiac surgery. A single center cohort study

### F Dogrul, W Vandenberghe, E Hoste

#### Ghent University Hospital, Ghent University, Department of Critical Care Medicine, Ghent, Belgium

*Critical Care* 2024, **28(Suppl 1):** P070

**Introduction:** Cardiac surgery-associated acute kidney injury (CSA-AKI) is influenced by both modifiable and non-modifiable factors. In this study we evaluated the association between the modifiable factors mean arterial pressure (MAP), central venous pressure (CVP) and mean perfusion pressure calculated as MAP-CVP (MPP) measured in the first 4 h (h) after intensive care unit (ICU) admission with the development of CSA-AKI within 72 h after cardiac surgery.

**Methods:** In this single center cohort study we collected data in adult patients who underwent cardiac surgery during a 5-year period (2012–2017). Exclusion criteria were cardiac transplantation, and use of ECMO or LVAD. We recorded baseline characteristics and MAP, CVP and MPP values during the first 4 h after ICU admission. The primary endpoint was CSA-AKI within 72 h according the Kidney Disease Improving Global Outcomes (KDIGO) definition based on both serum creatinine (SCr) and urine output (UO) criteria. We used Mann–Whitney and Chi2 test to compare groups, Area Under the Receiver Operating Characteristics (AUROC) and a logistic regression analysis to evaluate the association of the hemodynamic variables with CSA-AKI.

**Results:** A total of 3415 patients were included. CSA-AKI occurred in 2200 patients (64.4%). CSA-AKI patients were older, had more severe Chronic Kidney Disease (CKD) and had a higher Euroscore II. More CSA-AKI patients had emergency surgery, and combined CABG and valve surgery (all *p* < 0.001). Patients with CSA-AKI had lower MAP (71 vs. 72 mmHg, *p* < 0.001), higher CVP (10 vs. 9 mmHg, *p* < 0.001) and lower MPP (60 vs. 62 mmHg, *p* < 0.001) compared with patients without AKI. This association remained after adjustment for other risk factors (Table). ROC analysis showed that there was a weak association of lower MAP, higher CVP and lower MPP with CSA-AKI (Table).

**Conclusions:** Small changes in MAP, CVP and MPP during the first 4 h in the ICU were associated with CSA-AKI, even after adjustment for covariates. This association was weak for all 3 variables.

**Table (abstract P070):** Association of hemodynamic variables during the first 4 h of ICU admission and CSA-AKI within 72 h of ICU admission

	Area under the curve (95% CI)	*p* value
**A: AUROC analysis**		
MAP	0.443 (0.424–0.463)	< 0.001
CVP	0.600 (0.580–0.619)	< 0.001
MPP	0.405 (0.386–0.425)	< 0.001
B: Logistic regression analysis *	OR (95% CI)	*p* value
MAP (model 1)	0.990 (0.981–0.998)	0.016
CVP (model 1)	1.109 (1.083–1.136)	< 0.001
MPP (model 2)	0.988 (0.980–0.996)	0.003

*adjusted for age, chronic kidney disease (CKD) stage, admission reason (elective/urgent), type of surgery, cardiopulmonary bypass (CPB), Euroscore II (%). Goodness of fit for model 1 p=0.979 and for model 2 p=0.643. Abbreviations: MAP: mean arterial pressure, CVP: central venous pressure, MPP: mean perfusion pressure, CI: confidence interval

## P071 The modified ATRIA risk score predicts mortality for atrial fibrillation

### W Bahria^1^, F Hamdoun^2^, H Ouerghi^2^, D Hamdi^2^, F Azaiez^1^, F Ben Jaballah^2^, M Boussen^2^, NE Nouira^2^

#### ^1^Mongi Slim Academic Hospital, Cardiology Department, Tunis, Tunisia, ^2^Mongi Slim Academic Hospital, Emergency Department, Tunis, Tunisia

*Critical Care* 2024, **28(Suppl 1):** P071

**Introduction:** The modified ATRIA risk stroke score (M-ATRIA-RS) was used to predict thrombotic events and mortality for patients hospitalized for COVID-19. We aimed to investigate whether M-ATRIA-RS had a higher discrimination than CHA2DS2-VASc and than the ATRIA-RS for predicting mortality in patients with atrial fibrillation (FA).

**Methods:** This observational, prospective and analytical cohort study was conducted over 2 years, in an universal hospital, including patients admitted to the ED for atrial fibrillation. The ATRIA-RS, the m-ATRIA-RS (defined as a change in sex points of ATRIA RS from female to male) and the CHA_2_DS_2_VASc score were calculated at the admission for all patients. The primary end-point was the in-hospital mortality. ROC curves were used to identify variables predicting mortality.

**Results:** One hundred and fifty one patients were included in our study. The mean age was 67 years old [35–95] with a sex ratio of 0.62. The mean heart rate was 134 ± 24 pm. The mean CHA_2_DS_2_VASc score was 2.76 [0–7]. The mean ATRIA-RS and M-ATRIA-RS were respectively 4.52 [0–12] and 4.3 [0–11]. Forty patients (9.4%) died at the ED. The CHA_2_DS_2_VASc score, the ATRIA-RS and the m ATRIA-RS area under the curve for predicting in-hospital mortality were respectively (*p* = 0.002; AUC = 0.758; 95% CI [0.614–0.903]), (*p* = 0.001; AUC = 0.773; 95% CI [0.612–0.934]) and (*p* = 0.000; AUC = 0.817; 95% CI [0.682–0.952]).

**Conclusions:** M-ATRIA-RS is useful to predict in-hospital mortality among patients hospitalized with AF after the period of COVID-19. It is superior to ATRIA-RS and to CHA_2_DS_2_-VASc-RS in predicting mortality for patients with AF.

## P072 Preload responsiveness cannot be detected by micro-fluid challenge assessed by bioreactance

### N De Vita, R Shi, C Bruscagnin, D Rosalba, F Gavelli, A Pavot, C Lai, JL Teboul, X Monnet

#### Hôpital de Bicêtre, Hôpitaux Universitaires Paris-Saclay, INSERM UMR_S999, Université Paris-Saclay, AP-HP, Service de Médecine Intensive-Réanimation, Le Kremlin-Bicêtre, France

*Critical Care* 2024, **28(Suppl 1):** P072

**Introduction:** The micro-fluid challenge has been proposed to assess fluid responsiveness because of the very small volume of fluid administered. Different monitoring tools have been used to assess the efficacy of this test. Bioreactance has been proposed as a non-invasive method to measure cardiac output and to assess fluid responsiveness. We thus aim to test the ability of bioreactance to measure the changes in cardiac output induced by the rapid infusion of 50 mL of fluid.

**Methods:** In patients with acute circulatory failure, we rapidly infused 50 mL of saline over 30 s (micro-fluid challenge). We measured cardiac index by calibrated pulse contour analysis (PCCI) (PiCCO_2_, Getinge) and BioCI by bioreactance (Starling,Baxter). Preload responsiveness was defined by an increase in PCCI during a passive leg raising (PLR) test ≥ 10%.

**Results:** We included 46 patients, with mean age of 59 ± 15 years. The main reason of ICU admission was septic shock 28 (61%). There were 15 (33%) preload responders versus 31 (67%) preload non-responders. PCCI increased during PLR by 16 ± 4% in preload responders and by 2 ± 6% in non-responders (*p* < 0.001). Micro-fluid challenge-induced changes in PCCI were significantly different among preload responders and non-responders (2 ± 9% vs. 0 ± 2%, respectively; *p* = 0.04). The area under the receiver operating characteristic curve (AUROC) of micro-fluid challenge assessed by PCCI to detect preload responsiveness was 0.607 (0.433–0.781). Changes in BioCI during PLR and the micro-fluid challenge were not different between responders and non-responders (PLR: 8 ± 16% vs.11 ± 17%, respectively, *p* = 0.9; micro-fluid challenge: 2 ± 7% vs. 3 ± 10%, respectively, *p* = 0.78). The AUROC for the micro-fluid challenge assessed by bioreactance to detect preload responsiveness was 0.481 (0.308–0.655).

**Conclusions:** In patients with acute circulatory failure, preliminary results suggests that micro-fluid challenge is not a reliable method for predicting preload responsiveness, neither when assessed by pulse contour analysis, nor by bioreactance. The study is ongoing.

## P073 Intrarenal vein flow pattern trajectory correlates with alterations in fluid responsiveness in critically ill patients

### K Trigkidis^1^, S Kokkoris^1^, A Bogas^1^, I Siempos^1^, C Routsi^2^

#### ^1^Medical School, National and Kapodistrian University of Athens, Evangelismos Hospital, Athens, Greece, ^2^Medical School, National and Kapodistrian University of Athens, Evangelismos Hospital, ICU, Athens, Greece

*Critical Care* 2024, **28(Suppl 1):** P073

**Introduction:** Intra-renal vein flow (IRVF) pattern has been characterized as an ultrasonography marker of venous congestion [1]. We aimed to evaluate the relationship between the early trajectory of IRVF pattern with the fluid responsiveness, assessed by the passive leg raising (PLR) test.

**Methods:** This is a prospective, observational study including patients consecutively admitted to a multidisciplinary ICU from October 2022 through July 2023. Patients < 18 years old and those with chronic kidney disease, abdominal surgery and contraindications for PLR test, i.e., brain and spinal injury, were excluded. Ultrasonography assessment of IRVF patterns was obtained within 24 h (Day 1) and at 48-72 h following ICU admission (Day 3). At the time point of IRVF assessment, evaluation of fluid responsiveness was performed by a PLR test while measuring left ventricular outflow tract velocity–time integral (LVOT-VTI) via transthoracic echocardiography. An LVOT-VTI increase ≥ 10% was considered fluid responsiveness [2]. Fluid balance was recorded.

**Results:** A total of 56 mechanically ventilated patients [median age 62.5 (53–72) years, SOFA score 9 (8–12), 60% males, admission diagnosis: 53 (94.6%) medical and 3 (5.4%) surgical], were included. Fluid responsiveness on Days 1 and 3 were not associated with cumulative fluid balance or IRVF pattern. The difference in fluid responsiveness between the 2 days was inversely associated with the difference in fluid balance (*p* = 0.013), and the difference in the IRVF pattern (*p* = 0.008). Multiple regression revealed that the difference in fluid responsiveness was independently associated with the difference in IRVF pattern (RR = − 1.64, 95% CI − 3.07, − 0.21, *p* = 0.024).

**Conclusions:** In ICU patients, fluid responsiveness during the first 3 days following admission was not associated with cumulative fluid balance nor with IRVF pattern. However, temporal changes in IRVF pattern were related to changes in fluid responsiveness.

**References**

1. Wiersema R et al. J Crit Care. 2020;59:57
2. Monnet X et al. Crit Care Med. 2006;34:1402

## P074 Impact of an early deresuscitation strategy on mechanical ventilation duration in septic shock patients: a secondary analysis of two multicentre controlled trials

### W Juguet^1^, M Boubaya^2^, JP Quenot^3^, D Dreyfuss^4^, S Gaudry^1^

#### ^1^Hôpital Avicenne AP-HP, Intensive Care, Bobigny, France, ^2^Hôpital Avicenne AP-HP, Unité de Recherche Clinique, Bobigny, France, ^3^Hôpital François Mitterrand, Intensive Care, Dijon, France, ^4^Hôpital Tenon AP-HP, French National Institute of Health and Medical Research (INSERM), UMR S1155, CoRaKiD, Paris, France

*Critical Care* 2024, **28(Suppl 1):** P074

**Introduction:** The benefit of deresuscitation in critically patients with septic shock is widely recognized. The optimal time for its initiation remains unclear. We aimed to assess whether an early deresuscitation strategy defined by its initiation before vasopressor weaning may decrease mechanical ventilation (MV) duration.

**Methods:** This is a secondary analysis of two randomized trials (AKIKI, AKIKI2), focusing on renal replacement therapy initiation strategies. We included septic shock patients with severe acute kidney injury who received invasive MV and vasopressor support. The first day of deresuscitation was identified as the day when loop diuretics were first administered or when daily ultrafiltration volume exceeded 200 mL for patients receiving RRT. Patients were categorized into three groups based on their deresuscitation management: early deresuscitation (initiation before vasopressor weaning), delayed deresuscitation (initiation the day of vasopressor weaning or later) and no deresuscitation. The primary outcome was the time to successful extubation. To take into account the variation in deresuscitation decision overtime, we used a marginal structural model with inverse probability of treatment weighting (IPTW).

**Results:** Among 757 patients, 380 patients (50.2%) underwent an early deresuscitation strategy, 201 patients (26.6%) a delayed one and 176 (23.2%) did not undergo any deresuscitation. After checking the distribution of weights and covariate balance in groups, the Cox marginal structural model showed that the delayed deresuscitation was associated with reduced time to extubation. The hazard ratio for extubation was 1.86 [1.33–2.61](*p* < 0.0003) for the delayed group compared to the early one. The risk of occurrence of a composite outcome including ‘death’ or ‘RRT dependency’ or ‘absence of renal recovery’ at day 60 did not differ between groups.

**Conclusions:** In patients with septic shock and severe AKI, a deresuscitation initiated before vasopressor weaning was associated with increase duration of MV.

## P075 Perioperative fluid management in critically ill surgical intra-abdominal septic shock and outcome: SIASS-F-III STUDY (effect of preoperative and intraoperative vasopressor)

### S Kongsayreepong^1^, T Jaroensri^1^, N Deeprasert^2^, S Visuthisakchai^2^

#### ^1^Department of Anesthesiology, Siriraj Hospital, Mahidol University, Anesthesiology and Critical Care, Bangkok, Thailand, ^2^Department of Anesthesiology, Siriraj Hospital, Mahidol University, Bangkok, Thailand

*Critical Care* 2024, **28(Suppl 1):** P075

**Introduction:** In septic shock, fluid overload caused more complications while restricted fluid did not improve outcome. Early NE helped early restore MAP and decrease fluid intake. But still question about tissue perfusion and outcome. This study aimed to explore the effect of perioperative fluid management in pts with intra-abdominal septic shock admitting to SICU and outcome.

**Methods:** This prospective observational study was done in 255 pts, age > 18 years underwent surgery for intra-abdominal infection and had septic shock on SICU admission between June 2018 and Sep 2023. Pts underwent minimal invasive procedure, transplant, traumatic, cardiothoracic and obstetric surgery were excluded. Data record included patient’s demographics; ASA status; comorbidities; type and detail of anesthesia/surgery; vasopressor/inotropic support; steroid administration, detail of fluid, blood/blood component intake/output; serum alb, Cr, Hb, lactate; since day 0 (on ICU admission postop) until day 7. APACHE-II and SOFA score; and outcome as 28 day mortality and early postop complications.

**Results:** The incidence of 28 day mortality was 16.6%; early AKI, early RRT, ARDS, stroke, PMI, liver injury, bowel ischemia and DIC were 59.5%, 10.2%,40.0%, 5%, 7.3%, 31.7%, 11.1% and 31.2%; 35% had profound intraop hypotension, Higher intraop fluid balanced/kg was significantly associated with the discontinuation of vasopressor < 72 h, less incidence of postoperative AKI and RRT. From multivariate analysis. high ASA status (> III), poor control DM, not receive steroid, lower fluid bolus (< 39 mL/kg before starting NE), NE started within 1 h of fluid bolus, higher dose of intraop NE, lower intraop and day-0 (day of surgery and ICU admission); higher day 1 and day 3 fluid balanced/kg and lower day 2 serum alb were significant associated with 28 days mortality.

**Conclusions:** Restrictive fluid may not be the regimen for initial fluid resuscitation, intraop and day 0 postop in pts with intra-abdominal surgical septic shock.

## P076 Mortality in septic shock patients with persistent tachycardia treated with short-acting betablockers: a meta-analysis of randomized controlled trials

### MG Alexandru^1^, R Borgstedt^1^, T Whitehouse^2^, S Rehberg^1^, SS Scholz^1^

#### ^1^University Hospital Bielefeld, Campus Bielefeld-Bethel, University of Bielefeld, Department of Anaesthesiology, Intensive Care, Emergency Medicine, Transfusion Medicine and Pain Therapy, Bielefeld, Germany, ^2^College of Medical and Dental Sciences University of Birmingham, Institute of Inflammation and Ageing, Birmingham, UK

*Critical Care* 2024, **28(Suppl 1):** P076

**Introduction:** Treatment with short-acting betablockers in septic patients remains controversial as recent studies [1–3] indicated diverging results. We aimed to evaluate effects of treatment with short-acting betablockers on mortality in adult septic shock patients with persistent tachycardia.

**Methods:** Data search included PubMed, Web of Science, and the Cochrane Library. A meta-analysis of all eligible studies was performed in accordance with the PRISMA statement. Only randomized controlled studies providing valid classifications of septic shock and intravenous treatment of short-acting betablockers were included. All available data was analyzed comprehensively, with short-term mortality serving as the primary outcome, which contained the 28-day mortality [1–2] and the hospital mortality [3]. Sensitivity analyses were performed regarding potential substance related effects as well as biological heterogeneity (age).

**Results:** A total of 7 studies including 810 patients fulfilled the predefined criteria and were analyzed. The analysis of the short-term mortality suggested a significant reduction in patients who received short-acting betablockers. (Risk difference, − 0.11 [95% CI − 0.23 to 0.00]; *p* = 0.05; *p* for Cochran Q = 0.0005; I^2^ = 75%; Figure). Notably, one study [3] only reported the hospital mortality. Subgroup analysis on substance related effects indicated no significant subgroup difference (*p* = 0.16). Finally, analysis on potential biological heterogeneity (age) did not reveal a significant effect either (*p* = 0.20). Besides considerable statistical heterogeneity, no visual sign of publication bias was observed.

**Conclusions:** The administration of short-acting betablockers might reduce the short-term mortality in septic shock patients with persistent tachycardia. Future studies may be required to define subgroups who could benefit from this treatment.

**References**

1. Morelli A et al. JAMA. 2013;310:1683–1691
2. Whitehouse T et al. JAMA. 2023;330:1641–1652
3. Cocchi MN et al. Shock. 2022;57:508–517

**Figure (abstract P076):**
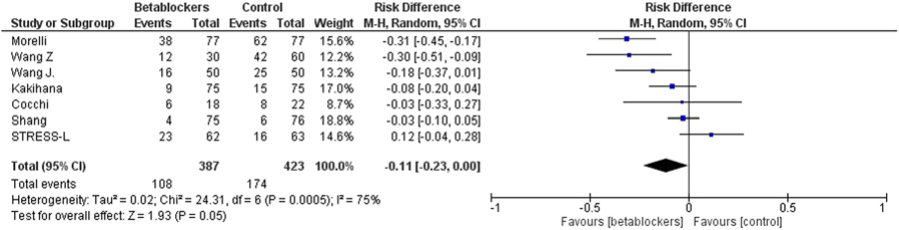
Short-term mortality, risk difference, betablockers treatment versus Control. M–H: Mantel–Haenszel, CI: confidence interval.

## P077 Comparison of the efficacy and safety of esmolol and landiolol in critically ill patients: a retrospective cohort study

### X Si, R Shi, H Yuan, W Song, J Guo, J Wu, X Guan

#### The First Affiliated Hospital of Sun Yat-sen University, Critical Care Medicine, Guangzhou, China

*Critical Care* 2024, **28(Suppl 1):** P077

**Introduction:** Esmolol, as a selective short-acting β-blocker, was shown to be beneficial for the prognosis of septic shock patients. However, recent studies did not observe the same effect of landiolol, which is a β1-superselective ultra short-acting intravenous agent. Our study aims to compare landiolol and esmolol regarding their performance in treating tachycardia and impact on the prognosis of critically ill patients.

**Methods:** This is a single center, retrospective cohort study. Patients with tachycardia who were treated with esmolol or landiolol without any other anti-arrhythmia drugs were included. Data were collected from the First Affiliated Hospital, Sun Yat-sen University from 06/2016 to 12/2022. Propensity score matching (PSM) was used to balance the baseline differences. The primary outcome is 28-day mortality. Secondary outcomes include heart rate reduction, hemodynamic stability, the ICU length of stay (ICULOS), and the hospital length of stay (HLOS).

**Results:** In total, 558 patients (372 in Esmolol vs. 186 in Landiolol) were included after PSM. The median age was 63 (IQR: 52–74) years old with 31% males. The main reason for ICU admission was follow-up after major surgery (62.4%), with the median SOFA score of 8 (IQR: 6–9). Among them, 45.2% had a sepsis/septic shock. The 28-day mortality in Landiolol group was significantly lower than that of the Esmolol group (12% vs. 23%, *p* < 0.001; KM curves: HR = 0.62 (0.39, 0.99), *p* = 0.041, Figure). Within the first 72 h of β-blocker administration, landiolol lowered heart rate by 5 (2, 8) beats per minute more than esmolol (*p* = 0.001) with no difference in blood pressure and dose of norepinephrine between two groups. There was no difference in the HLOS between the two groups (26.0 vs. 26.0 days, *p* = 0.651), while the Landiolol group had a significantly shorter ICULOS (4.8 vs. 7.0 days, *p* = 0.005).

**Conclusions: **In critically ill patients with tachycardia, compared to esmolol, landiolol can control the heart rate more effectively and may lead to a better prognosis.

**Figure (abstract P077):**
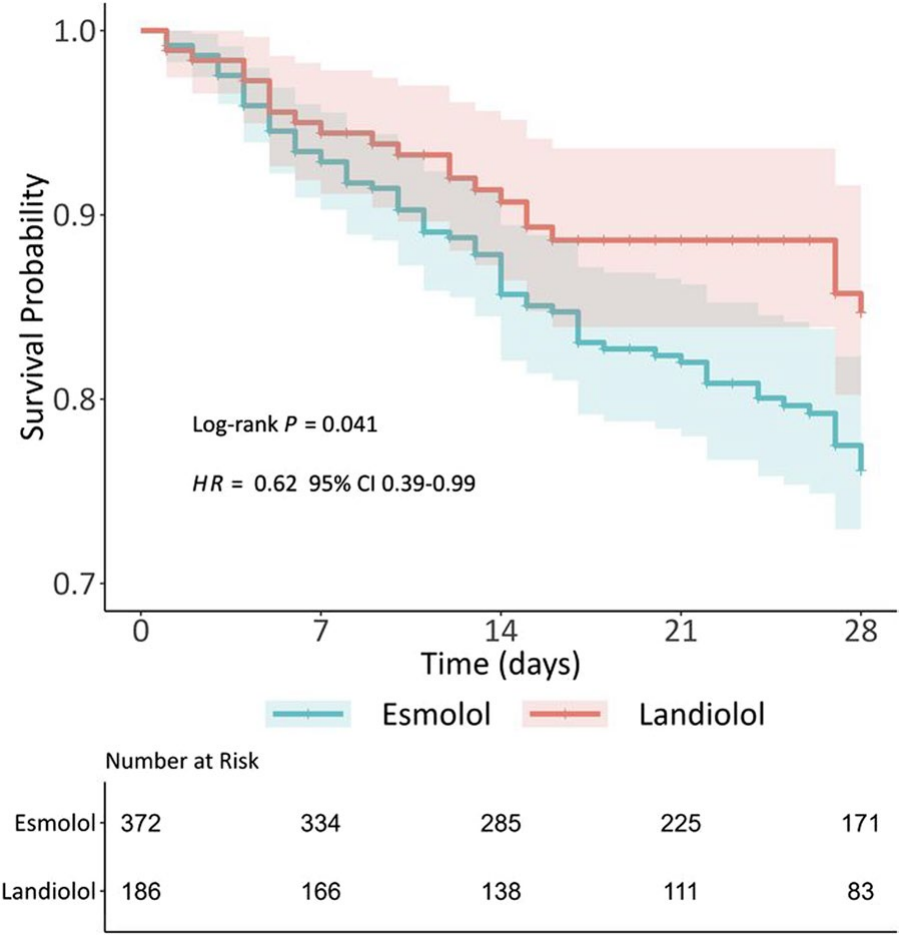
Kaplan–Meier survival curves of the two groups of patients.

## P078 Continuous monitoring of mitral annular plane systolic excursion using transesophageal echocardiography and deep learning predicts postoperative cardiac biomarkers

### J Yu^1^, E Skogvoll^1^, TD Tannvik^2^, AA Taskén^3^, B Grenne^1^, I Kirkeby-Garstad^4^, S Aakhus^1^

#### ^1^Norwegian University of Science and Technology/St Olav´s Hospital, Department of Circulation and Medical Imaging/Department of Anesthesia and Intensive Care, Trondheim, Norway, ^2^St Olavs Hospital, Department of Anesthesia and Intensive Care, Trondheim, Norway, ^3^Norwegian University of Science and Technology, Department of Computer Science, Trondheim, Norway, ^4^Norwegian University of Science and Technology, Department of Circulation and Medical Imaging, Trondheim, Norway

*Critical Care* 2024, **28(Suppl 1):** P078

**Introduction:** Perioperative myocardial distress often leads to left ventricular (LV) dysfunction and postoperative release of cardiac biomarkers. Since echocardiographic LV dysfunction presents before change in cardiac biomarkers, we have developed a tool for continuous monitoring of mitral annular plane systolic excursion (MAPSE). This method uses deep learning to automatically estimate MAPSE on transesophageal echocardiographic images obtained in a hands-free manner (autoMAPSE). Our aim was to test the ability of continuous autoMAPSE in predicting the subsequent release of the two cardiac biomarkers N-terminal pro B-type natriuretic peptide (proBNP) and high-sensitivity troponin T (TnT).

**Methods:** We monitored 50 patients for two hours after cardiac surgery. Every 5 min, we recorded mean arterial pressure, heart rate and autoMAPSE. Every 40 min, we obtained arterial and central venous blood gasses. We also performed one echocardiographic exam at the beginning or the end of the protocol. ProBNP and TnT were repetedly measured postoperatively from ICU arrival to the first postoperative evening and summarized by their respective area under the curve. The hemodynamic parameters and autoMAPSE were summarized by their respective time-weighted averages (TWA). Finally, we divided the patients into two groups by the median of autoMAPSE and compared proBNP and TnT.

**Results:** Continuous autoMAPSE was feasible in 48 patients (96%). Of all the parameters, the TWA of autoMAPSE was the only one that correlated significantly with both proBNP and TnT (Table). In the two groups defined by median autoMAPSE, both proBNP and TnT were significantly different by t-test (*p* < 0.01 and *p* < 0.05, respectively).

**Conclusions:** The TWA of autoMAPSE in the two first postoperative hours predicted the next day release of both proBNP and TnT. Compared to current practice, continuous monitoring of LV function seems to improve the sensitivity for detecting subsequent release of proBNP and TnT.

**Table (abstract P078):** Correlation coefficients with cardiac biomarkers

	ProBNP (AUC)	TnT (AUC)
AutoMAPSE (TWA)	− 0.44**	− 0.32*
Mean arterial pressure (TWA)	− 0.24	− 0.16
Heart rate (TWA)	0.16	0.00
ScvO_2_ (TWA)	− 0.13	− 0.39**
AutoMAPSE (single measurement)	− 0.34*	− 0.11
Ejection fraction	− 0.52**	− 0.16
Cardiac output	− 0.10	− 0.15

AUC, area under the curve; autoMAPSE, automatic estimated mitral annular plane systolic excursion; proBNP, N-terminal pro B-type natriuretic peptide; ScvO_2_, central venous oxygenation; TnT, high-sensitivity troponin T, TWA, time-weighted average. *indicates *p* < 0.05 **indicates *p* < 0.01.

## P079 Continuous mitral annular plane systolic excursion monitored using transesophageal echocardiography and deep learning reflects ventriculo-arterial coupling

### J Yu^1^, E Skogvoll ^1^, TD Tannvik^2^, AA Taskén^3^, B Grenne^1^, I Kirkeby-Garstad^4^, S Aakhus^1^

#### ^1^Norwegian University of Science and Technology/St Olav’s Hospital, Department of Circulation and Medical Imaging/Department of Anesthesia and Intensive Care, Trondheim, Norway, ^2^St Olavs Hospital, Department of Anesthesia and Intensive Care, Trondheim, Norway, ^3^Norwegian University of Science and Technology, Department of Computer Science, Trondheim, Norway, ^4^Norwegian University of Science and Technology, Department of Circulation and Medical Imaging, Trondheim, Norway

*Critical Care* 2024, **28(Suppl 1):** P079

**Introduction:** Both left ventricular (LV) function and ventriculo-arterial (VA) coupling are crucial during hemodynamic optimization. We have developed a method for continuous monitoring of LV function by automatically obtaining mitral annular plane systolic excursion (MAPSE) using deep learning in transesophageal echocardiographic images (autoMAPSE). As MAPSE reflects load-dependent contraction, we hypothesize that the clinical behavior of autoMAPSE reflects VA coupling.

**Methods:** We explored the relationship between autoMAPSE and VA coupling in 50 ICU patients after cardiac surgery. We approached VA coupling in two ways. The first was by exploring the relationship between MAPSE and mean arterial pressure (MAP) within each patient. For this, we recorded autoMAPSE and MAP every 5 min for two hours. We then assessed the within-patient MAPSE/MAP-relationship using a mixed linear model with random slopes and intercepts. We quantified this relationship with the intra-class correlation. The second approach was by estimating VA coupling as the ratio between effective arterial elastance and end-systolic elastance (Ea/Ees-ratio). Using transesophageal echocardiography, we estimated end-systolic elastance noninvasively and effective arterial elastance as (0.9 × systolic arterial pressure)/stroke volume.

**Results:** We found a strong within-patient MAPSE/MAP-relationship with highly significant random slopes (intra-class correlation = 0.81, *p* < 0.001). This MAPSE/MAP-relationship varied between patients, indicated by the different slopes for each patient (Figure). There was a significant correlation between autoMAPSE and Ea/Ees-ratio (*r* = − 0.5, *p* < 0.01), Ees (r = 0.51, *p* < 0.01) but not with Ea (r = − 0.15, *p* > 0.05).

**Conclusions:** Each patient has a unique MAPSE/MAP-relationship and autoMAPSE correlates with VA coupling. This suggests that VA coupling may be continuously monitored using autoMAPSE.

**Figure (abstract P079):**
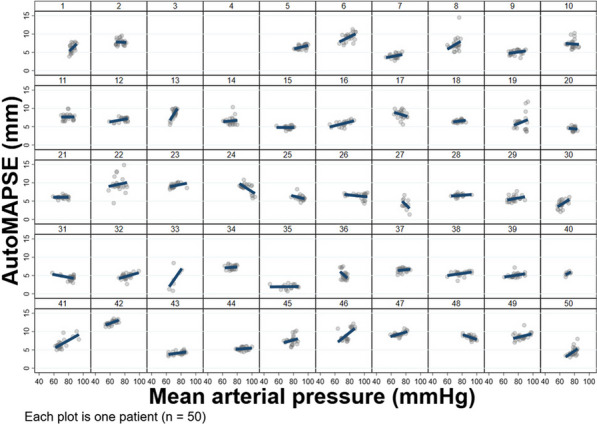
Automatically estimated mitral annular plane systolic excursion (autoMAPSE) plotted against mean arterial pressure for each patient. Navy line represents the fitted linear relationship. Patient number 3 and 4 did not have any feasible autoMAPSE measurements.

## P080 Evaluation of bias and trending ability of non-calibrated multi-beat analysis continuous cardiac output monitoring in critically patients

### L Bitker^1^, I Noirot^2^, L Chauvelot^2^, M Mezidi^2^, F Dhelft^2^, M Gaillet^2^, H Yonis^2^, G Deniel^2^, JC Richard^2^

#### ^1^Hôpital de la Croix Rousse, Hospices Civils de Lyon, Médecine Intensive - Réanimation, Lyon, France, ^2^Hôpital de la Croix Rousse, Hospices Civils de Lyon, Lyon, France

*Critical Care* 2024, **28(Suppl 1):** P080

**Introduction:** Prediction of fluid responsiveness using non-calibrated continuous cardiac output remain poorly evaluated. We aimed to evaluate bias and trending ability of non-calibrated multi-beat analysis continuous cardiac index (CCI_MBA_), against calibrated pulse-contour analysis continuous cardiac index (CCI_PCA_) in ICU patients undergoing a passive leg raise (PLR) and/or a fluid challenge (FC).

**Methods:** In this single-center observational prospective study, we enrolled hemodynamically stable adult patients treated with norepinephrine, monitored with transpulmonary thermodilution-calibrated CCI_PCA_ (reference), and in which a PLR and/or a FC (500 mL in < 15 min) was clinically indicated. After connection of the CCI_MBA_ device (evaluated method) to the bedside monitor, paired CCI_MBA_ and CCI_PCA_ were recorded prior to (baseline replicate) and during the PLR and/or FC (maximum replicate, i.e. highest value). Fluid responsiveness was identified if the relative change in CCI_PCA_ between baseline and maximum replicates (∆%25CCI_PCA_) was > 10% during PLR and > 15% during FC. Bias of paired data was evaluated using all replicates, and the trending ability using 4-quadrant and radial plots. ∆%25CCI_MBA_ optimal threshold during PLR to predict fluid responsiveness (∆%25CCI_PCA_ > 15% during FC) was estimated using the receiver-operating curve (AUROC).

**Results:** 29 patients (median 68 [IQR: 57–74] yo, SOFA 12 [8–14]) received 28 PLR and 16 FC. CCI_MBA_ and CCI_PCA_ were significantly correlated (R^2^ = 0.73). The bias between methods increased with higher cardiac index values (Figure panel A). ∆%25CCI_MBA_ adequately tracked changes in ∆%25CCI_PCA_ (Figure panel B) with an angular bias of 2 ± 23° (*p* = 0.52 compared to 0°, Figure panel C). ∆%25CCI_MBA_ during a PLR had an AUROC of 0.82 (*p* < 0.05), and an optimal diagnostic threshold > 15% to predict fluid responsiveness (sensitivity: 0.99, specificity: 0.76).

**Conclusions:** Non-calibrated CCI_MBA_ showed a non-constant bias but an adequate ability to track changes in CCI_PCA_ and good performance to predict fluid responsiveness.

**Figure (abstract P080):**
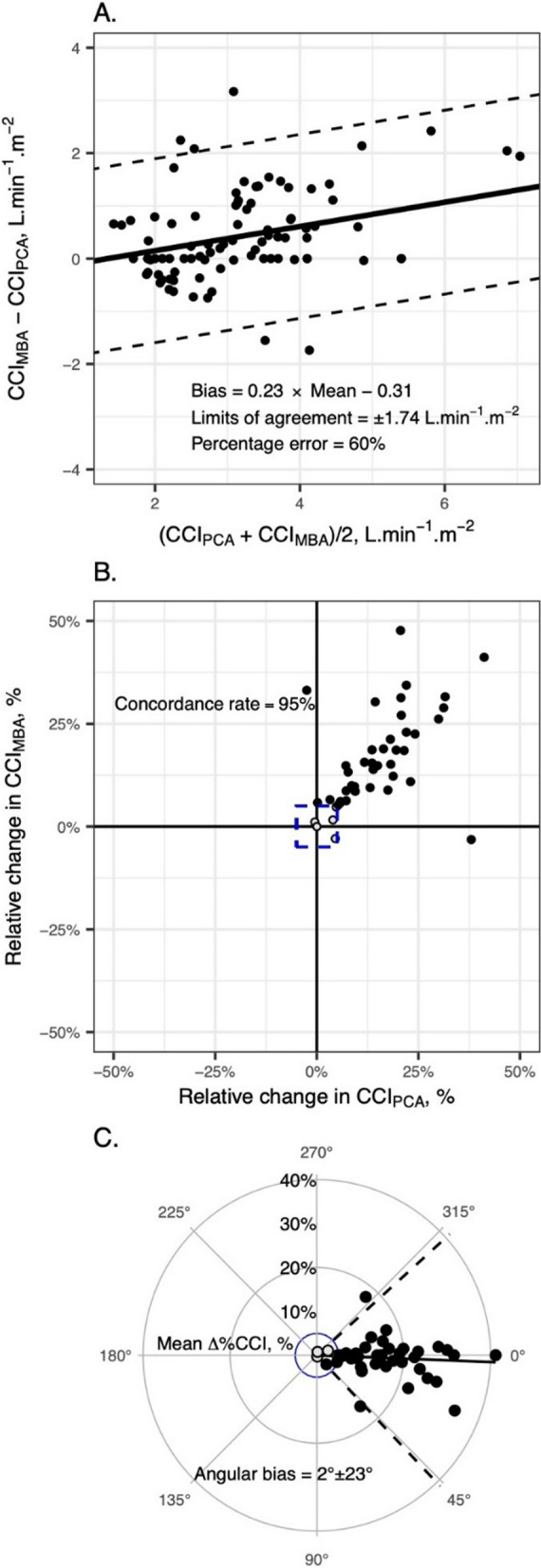
Bland and Altman plot of CCI_MBA_ against CCI_PCA_ (panel A), quadrant plot of ∆%CCI_MBA_ against ∆%CCI_PCA_ (panel B), and corresponding radial plot (panel C). Broad solid lines are bias (A) and angular bias (C), and dashed lines are their limits of agreement. Vertical and horizontal solid lines delimit quadrant limits (B). The blue square/circle identifies excluded ∆%CCI values with a relative change < 5% (B and C). The equation describing the non-constant bias is given in panel A.

## P081 Hemodynamic management and monitoring practices in intensive care units in Portugal: a national survey

### FA Gonzalez^1^, JP Silva^2^, M Mourisco^3^, JJ Martins^4^

#### ^1^Hospital Garcia de Orta, Intensive Care Medicine Department, Almada, Portugal, ^2^Hospital Vila Franca de Xira, Intensive Care Medicine Department, Lisboa, Portugal, ^3^Centro Hospitalar de Entre o Douro e Vouga, Intensive Care Medicine Department, Santa Maria da Feira, Portugal, ^4^Hospital de Braga, Intensive Care Medicine Department, Braga, Portugal

*Critical Care* 2024, **28(Suppl 1):** P081

**Introduction:** The Surviving Sepsis Campaign and the ESICM consensus on circulatory shock and hemodynamic monitoring are some of the few guidelines available for patients developing hemodynamic instability. We designed an electronic survey to better understand current practice and alignment with these guidelines, in intensive care units (ICUs) in Portugal.

**Methods:** A short questionnaire was shared on social networks or via email (e-blast) by the Portuguese Society of Critical Care from May 11th to September 11th 2023 to answer 24 questions about hemodynamic monitoring and management in the ICU.

**Results:** Globally, 174 valid questionnaires were available for analysis. 54% were intensive care specialists, and the rest were in training. For the patient in septic shock, the volume of fluid challenge differed between 500 mL (42%), 250 mL (30.5%), and 1000 mL (19%), and the most used type of fluid used was balanced solution (85.5% vs 14.4% saline). The main criteria for starting vasopressors were a MAP ≤ 65 mmHg after 30 mL/kg of fluids and peripheral perfusion compromise (77.6%), with norepinephrine being universally the first choice. For the second vasopressor, 66.1% chose vasopressin. The most frequently used tools to assess fluid responsiveness were the evaluation of IVC (52.3%), cardiac output by echocardiography (45.4%), and PPV measurement (44.8%). The hemodynamic monitoring devices most used by the responders were echocardiography (97.1%), transpulmonary thermodilution system (82.2%), and pulmonary artery catheter (45.4%).

**Conclusions:** Our survey suggests that most patients in septic shock in Portuguese ICUs receive fluids, norepinephrine, and vasopressin, according to current recommendations. Echocardiography was one of the main tools to assess volume status, fluid responsiveness, and hemodynamic monitoring. Still, almost a third of responders considered that more than half of the team was not skilled enough to perform echocardiography, highlighting the need for training and standardization in this technique.

## P082 Improving the governance of focused echocardiography on a district general hospital critical care unit

### MI Smith, M Sheikh, P Parulekar

#### William Harvey Hospital, Critical Care, Ashford, UK

*Critical Care* 2024, **28(Suppl 1):** P082

**Introduction:** In this work we aimed to improve the governance of focused echocardiography (echo) in a district general hospital critical care. Focused echo is increasingly used in critical care to aid management and diagnosis of shock syndromes, fluid tolerance and cardiac output assessment. Effective governance is essential to ensure its appropriate use and interpretation. Key to the governance of echo is the ability to access and review images of scans. UK guidelines state that focused echo images should be stored in a location other than on the machine [1]. Use of portable ultrasound machines to undertake focused echo can impede this.

**Methods:** We undertook a baseline audit in May 2023. Data was collected for 1 month and all focused echos carried out on the critical care unit were included. Scans performed purely for accreditation purposes were excluded. Interventions were to present the findings of this audit at a departmental meeting, arrange for all echo and ultrasound machines to upload scans to our institution’s online echo database and introduce a monthly echo governance meeting. The audit was then repeated in October 2023.

**Results:** 31 focused echos were performed in each 1-month cycle. The majority of scans (76%) were performed using portable ultrasound machines and almost half of all echos were performed out of hours. Our interventions increased the ability to review echo images online from 39 to 71% (Figure). The greatest area of improvement was seen in out of hours echos where this improved from 25 to 67% of scans. Scans with a documented report improved slightly from 65 to 71%.

**Conclusions:** Connecting all echo and ultrasound machines to our institutions echo database and the introduction of a focused echo governance meeting greatly increased the proportion of focused echos with images available to review online. This not only improved compliance with UK guidelines but also greatly increased opportunities for echo governance.

**Reference**

1. Faculty of Intensive Care Medicine. Guidelines For The Provision of Intensive Care Services v2.1 (2022)

**Figure (abstract P082):**
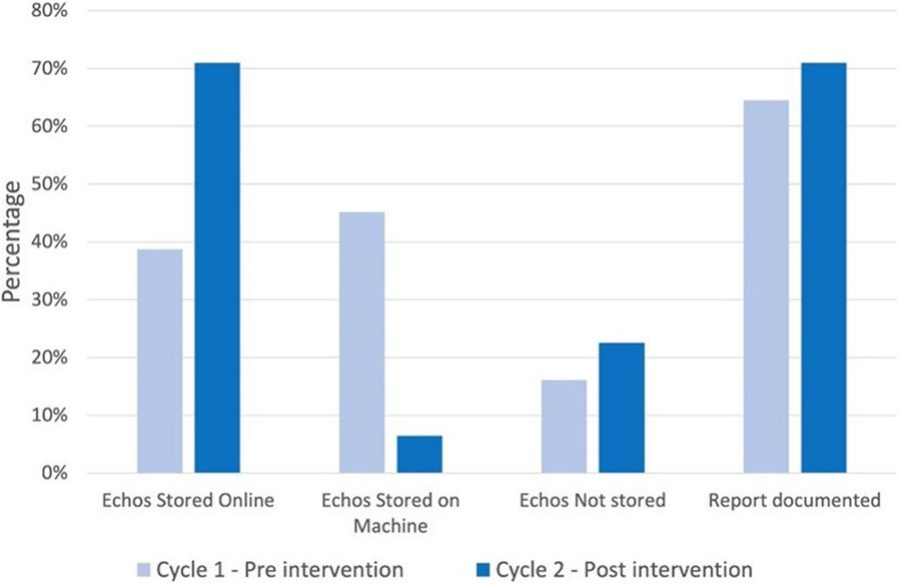
Bar chart showing the main governance findings before and after intervention with the introduction of a governance meeting and improved connectivity of echo machines.

## P083 Let us talk about ultrasound club: weekly peer-led teaching sessions on ultrasound in an intensive care unit

### E Mclean, C Passalacqua, DE Samuel

#### London Northwest Healthcare NHS Trust, Intensive Care Unit, London, UK

*Critical Care* 2024, **28(Suppl 1):** P083

**Introduction:** Using point-of-care ultrasound (POCUS) in the intensive care unit (ICU) is becoming increasingly important and useful for diagnostics and monitoring our patients’ response to treatment. However, training in POCUS within ICU is variable by unit and often ad hoc leading to a lack of confidence among trainee doctors and Advanced critical care practitioners (ACCPs) to use this modality at the bedside. We, therefore, sought to create a teaching programme to increase confidence in using POCUS and extend knowledge.

**Methods:** We created a weekly peer-led teaching programme on POCUS. We use a hybrid format with a small group tutorial available to join online or in person, followed by practical scanning. This was designed to increase both theoretical knowledge and hands-on practice for skills in the acquisition and understanding of images. We surveyed the staff attending to monitor changes in self-reported level of knowledge and confidence. We also sought feedback on the quality of the sessions and had an adaptive program so we could change the session format or content accordingly.

**Results:** So far, each session has been attended by 4–11 trainee doctors and ACCPs. We have found consistent improvement in self-reported knowledge of POCUS, confidence in obtaining POCUS images, and interpretation of images. Furthermore, the mode has increased substantially for each category showing a growing skew towards confidence within the group. There has also been complimentary feedback about the sessions and repeated attendance for both online and in-person teaching.

**Conclusions:** Overall, we have found that providing a dedicated weekly peer-led teaching session about ultrasound for trainee doctors and ACCPs in ICU has increased overall knowledge and confidence in using POCUS. This has benefits for both the individual clinicians' further professional development but also patients having increased access to enthusiastic clinicians with improved training in POCUS.

## P084 Setting up an echocardiography teaching and governance meeting within critical care in a district general hospital

### M Sheikh, M Smith, P Parulekar

#### William Harvey Hospital, Critical Care, Ashford, UK

*Critical Care* 2024, **28(Suppl 1):** P084

**Introduction:** We looked at the feasibility of introducing an echo governance meeting in a district general hospital. Focussed echocardiography is an important component in the diagnosis and management of the critical care patient. It must be performed by appropriately trained individuals and be subject to data governance and audit/quality improvement [1].

**Methods:** In May 2023 a monthly meeting was established for echo teaching and governance. The meeting was broadcast in person and virtually. Each session had a FICE/FUSIC accredited junior to act as chair and at least one BSE level 2 accredited critical care clinician. Sessions were advertised to multiple departments trust-wide. Sessions included the review of focussed echos and more recently, a five-question review related to data governance. At the beginning and end of each session a questionnaire was distributed.

**Results:** From 7 meetings, 47 responses were generated from in-person attendees. Just over half of attendees had a critical care background and the remainder were from anaesthetics, general medicine, respiratory medicine and emergency medicine. Attendees included consultants, trainees and wider members of the MDT. 42 post-meeting questionnaires returned extremely positive data: 100% of attendees reported the meeting interesting and found they learnt something; 98% found the meeting educational and useful; 66% said the meeting altered their practice; and 74% reported the meeting encouraged them to consider echo governance such as storing images and writing reports (Figure).

**Conclusions:** This shows it is not only possible, but beneficial, to introduce echo governance in a district general hospital. Future work includes the introduction of a 24/7 echo helpline for immediate review of scans, and increasing the frequency of these meetings to weekly, embedding this as a key part of practice.

**Reference**

1. Faculty of Intensive Care Medicine. Guidelines For The Provision of Intensive Care Services v2.1 (2022)

**Figure (abstract P084):**
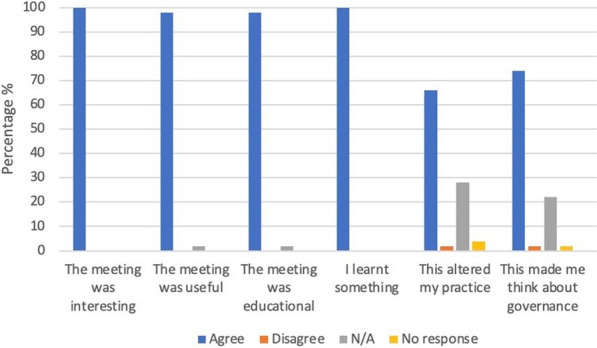
A bar chart to show the feedback obtained from echo governance meetings.

## P085 Clinically framed analysis of the hypotension prediction index: comparison of methodological approaches

### S Davies^1^, Z Jian^2^, D Sessler^3^, N Fleming^4^, M Mythen^2^, D Veelo^5^, T Scheeren^2^, M Sander^6^, M Cannesson^7^, F Hatib^2^

#### ^1^York and Scarborough Teaching Hospitals NHS foundation Trust, York, UK, ^2^Edwards Lifesciences, Irvine, USA, ^3^Cleveland Clinic, Cleveland, Ohio, USA, ^4^UC Davis School of Medicine, Sacramento, CA, USA, ^5^Amsterdam University Medical Center, Amsterdam, Netherlands, ^6^University Hospital Giessen, Giessen, Germany, ^7^UCLA School of Medicine, Los Angeles, CA, USA

*Critical Care* 2024, **28(Suppl 1):** P085

**Introduction:** Hypotension is associated with organ injury including acute kidney injury and myocardial injury in surgical and critically ill patients. The Acumen Hypotension Prediction Index (HPI) software is a machine learning algorithm that detects early hemodynamic instability that may lead to hypotension. The original validation of HPI used a case control analysis and excluded data from a zone of uncertainty (grey zone) [1]. It is important that clinically used algorithms reflect the use of data in a real-world setting. We therefore conducted a clinically framed analysis of the ability of HPI.

**Methods:** We conducted a retrospective analysis of data from 9 previously reported studies. The data were analysed with 2 different methods and receiver operating characteristic curves constructed: a case–control analysis with a grey zone approach [1], and a cohort analysis that used all data points [2–6]. In the analyses hypotension was defined as mean arterial pressure < 65 mmHg for ≥ 1 min.

**Results:** 2022 patients (913 female) were included, 339 (17%) were from intensive care units and 1683 (83%) were surgical. 1683 patients (83%) had at least 1 hypotensive event and in total there are 24,654 hypotensive events. The case–control analysis shows the area-under-ROC-curve for HPI to predict hypotension 5, 10, and 15 min in advance was 0.957 (95% CI 0.947–0.964), 0.933 (0.924–0.942), and 0.929 (0.918–0.938), respectively (Figure). The cohort analysis shows corresponding AUROC was 0.923 (95% CI 0.912–0.933), 0.923 (0.911–0.933), and 0.926 (0.914–0.937), respectively (Figure).

**Conclusions:** HPI was shown to be able to predict hypotension with high performance, using a clinically relevant analysis methodology and the originally reported case–control methodology.

**References**

1. Hatib F et al. Anesthesiology. 2018;129:663–674
2. Bedoya AD et al. JAMIA Open. 2020;3:252–260
3. Hyland SL et al. Nat Med. 2020;26:364–373
4. Tomasev N et al. Nature. 2019;572:116–119
5. Wiens J et al. J Mach Learn Res. 2016;17:2797–819
6. Wijnberge M et al. Eur J Anaesthesiol2021;38:609–15

**Figure (abstract P085):**
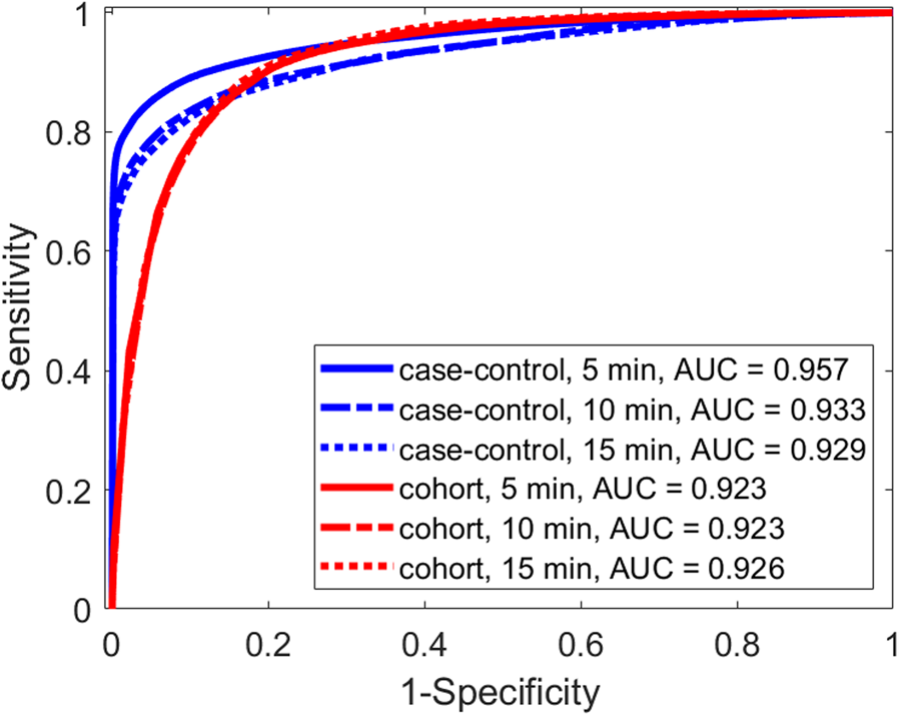
Performance of HPI in predicting hypotension, analyzed with a case–control approach, and a cohort approach using all data points. Both analyses show HPI predicts hypotension with high accuracy.

## P086 Assessing the statistical impact of mathematical coupling of continuous map data to predict hypotension

### F Hatib^1^, Z Jian^1^, S Davies^2^, M Mythen^1^

#### ^1^Edwards Lifesciences, Critical Care, Irvine, USA, ^2^York Teaching Hospital National Health Service Foundation Trust, York, UK

*Critical Care* 2024, **28(Suppl 1):** P086

**Introduction:** The issue of mathematical coupling of data is well known in medicine. It has been suggested that MAP can be used to predict hypotension [1]. Hypotension is a condition where flow to local organs is disrupted leading to hypoperfusion, but it is often defined with simple arbitrary thresholds of MAP. The purpose of this study is to assess the impact of mathematical coupling on ROC analysis when MAP is used to predict hypotension.

**Methods:** A total of 2022 patients yielding 4,152,124 datapoints were used to construct ROCs for MAP, SV and HR to predict a decrease of themselves for random thresholds 10 min into the future. The patient data are from 9 published studies. Additionally, simulation was performed, where time-series of randomly increasing and decreasing numbers in a range of 0–5 with no particular meaning were generated. 1000 signals each of a duration of 10 h were simulated and ROC for a randomly selected threshold was constructed.

**Results:** The Figure shows ROC curves for SV, HR and MAP predicting a decrease of themselves. All variables have a high AUC in predicting their future values regardless of the threshold chosen. The simulation shows equally high AUC.

**Conclusions:** MAP does not predict hypotension as a condition of local organ hypoperfusion. The high AUC is caused by mathematical coupling when MAP is used to predict a threshold in its range. When using the current MAP to predict MA* p* < 65 mmHg, there is a natural relationship that creates a statistical dependency. The ROC analysis simply illustrates the likelihood that a MAP value of 65 mmHg will be preceded by other MAP values (e.g., 67 or 70 mmHg), which is intuitive since to reach a hypotensive event from a higher MAP, the blood pressure must pass through adjacent values. As the Figure shows, all hemodynamic variables and the simulation data ‘predict’ themselves regardless of the chosen threshold due to this statistical coupling in a time series analysis.

**Reference**

1. Mulder MP et al. Anesthesiology. 2023;38:657–658

**Figure (abstract P086):**
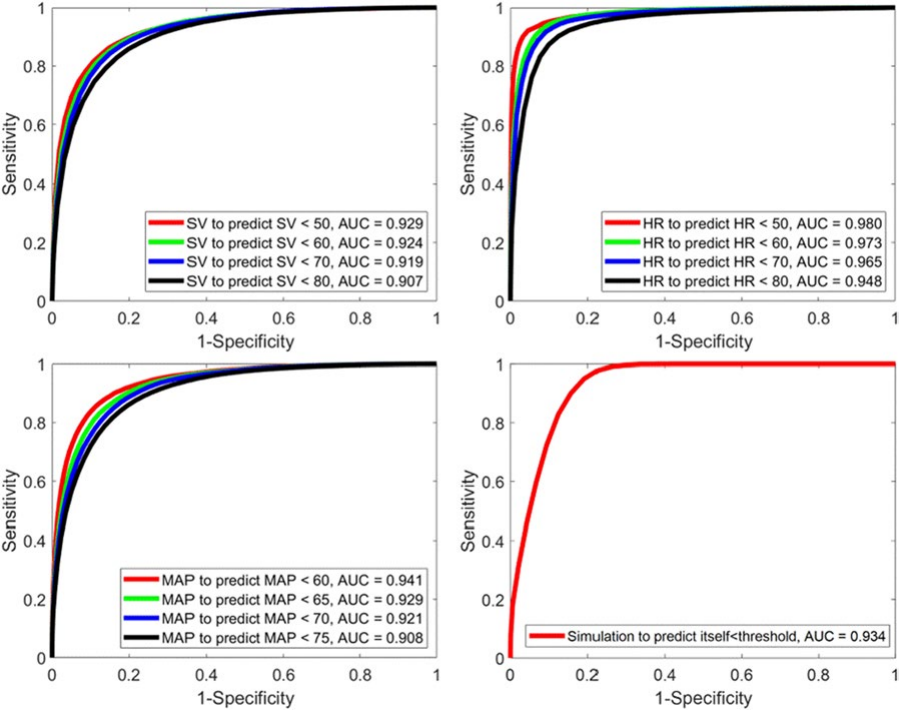
ROC curves for SV, HR, MAP and Simulation of random time-series predicting a decrease below various thresholds 10 min into the future.

## P087 Derivation and validation of endotypes for hypotension using unsupervised deep learning

### Z Jian^1^, X Liu^1^, J Settels^1^, K Kouz^2^, S Davies^3^, T Scheeren^1^, F Hatib^1^, B Saugel^2^

#### ^1^Edwards Lifesciences, Irvine, USA, ^2^University Medical Center Hamburg-Eppendorf, Hamburg, Germany, ^3^York Teaching Hospital National Health Service Foundation Trust, York, UK

*Critical Care* 2024, **28(Suppl 1):** P087

**Introduction:** Hypotension is associated with organ injury including acute kidney injury and myocardial injury in surgical and critically ill patients. Understanding the varied underlying physiological mechanisms that lead to hypotension may help choose specific therapeutic interventions to causally treat hypotension. We aimed to identify endotypes for hypotension using big data and unsupervised deep learning methods.

**Methods:** We developed an unsupervised deep learning algorithm to identify endotypes of hypotension. The algorithm is a deep learning autoencoder model combined with unsupervised learning. The autoencoder model captures the most important information of the input data to reduce data dimensionality, and the unsupervised learning classifies the information into a number of types in the autoencoder latent space. Stroke volume index, heart rate, systemic vascular resistance index, and stroke volume variation were the inputs to the algorithm. Hypotension was defined as mean arterial pressure < 65 mmHg for at least 1 min. The algorithm was developed using 871 surgical patients who had 6848 hypotensive events. We validated the algorithm using an additional 926 surgical patients who had 4842 hypotensive events and 1000 critically ill patients who had 31,615 hypotensive events.

**Results:** Unsupervised deep learning revealed four distinct hypotension endotypes. Based on their physiological and clinical characteristics, these endotypes can be labeled as hypovolemia, vasodilation, bradycardia, and myocardial depression (Figure). The independent validation data confirmed these four hypotension endotypes.

**Conclusions:** With unsupervised deep learning we found four endotypes of hypotension, namely hypovolemia, vasodilation, bradycardia, and myocardial depression. Considering these hypotension endotypes may help clinicians identify the underlying cause of hypotension and choose specific therapeutic interventions to causally treat hypotension.

**Figure (abstract P087):**
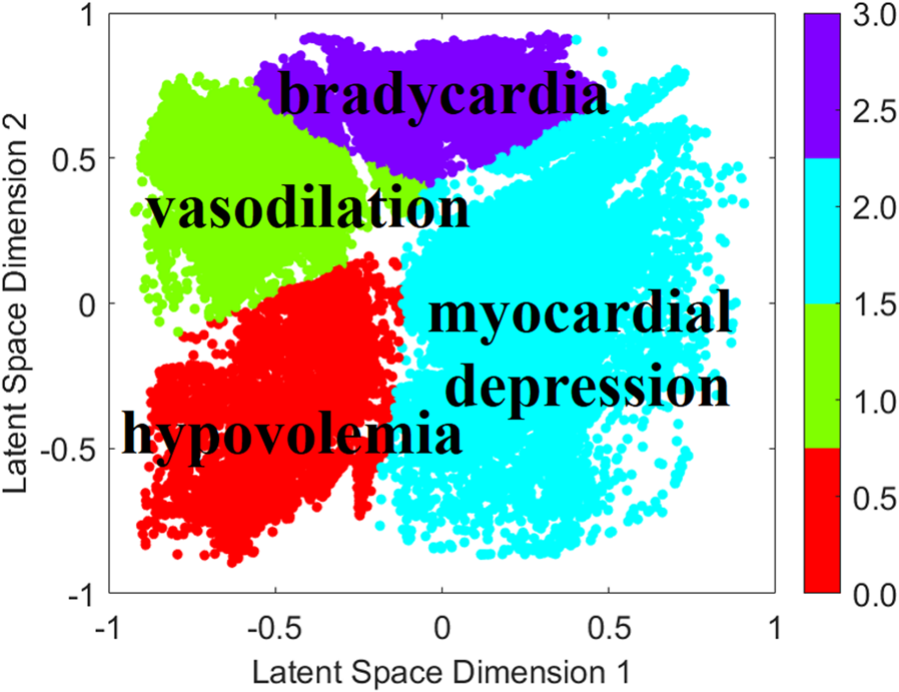
Four endotypes for hypotension in the autoencoder latent space: hypovolemia (red), vasodilation (green), bradycardia (purple), and myocardial depression (cyan).

## P088 Elevated dipeptidyl peptidase 3 predicts morbidity and mortality in cardiogenic shock: pre-specified secondary analyses from the ACCOST-HH trial

### M Karakas^1^, A Picod^2^, H Nordin^2^, T Zeller^3^, C Oddos^2^, K Santos^4^, D Jarczak^5^, A Mebazaa^5^, F Azibani^2^, S Kluge^5^

#### ^1^University Medical Center Hamburg-Eppendorf, Department of Intensive Care Medicine, Hamburg, Germany, ^2^INSERM UMR-S 942 MASCOT, Paris, France, ^3^University Heart & Vascular Center Hamburg-Eppendorf, Hamburg, Germany, ^4^4TEEN4 Pharmaceuticals, Hennigsdorf, Germany, ^5^University Medical Center Hamburg-Eppendorf, Hamburg, Germany

*Critical Care* 2024, **28(Suppl 1):** P088

**Introduction:** Here we evaluated the prognostic value of dipeptidyl peptidase 3 (DPP3), a zinc-dependent metalloprotease that inactivates angiotensin II and related peptides, and thereby initiates and perpetuates cardiogenic shock according to recent molecular studies.

**Methods:** The investigator-initiated, multi-center, placebo-controlled, phase 2 study ACCOST-HH was the very first clinical trial to evaluate a monoclonal antibody, adrecizumab, in cardiogenic shock [1]. As pre-specified in the protocol, circulating DPP3 levels were measured at baseline and after 72 h within the ACCOST-HH study, and were associated with 30-day mortality and organ support measures.

**Results:** A DPP3 concentration of 40 ng/mL, derived from the 97.5th percentile in the general population, was pre-defined as cut-off. Within the study, median DPP3 concentration at baseline was 43.2 ng/mL [21.2–74]. Elevated DPP3 was associated with a higher 30-day mortality (hazard ratio 1.7 [95% CI 1.0–2.9], as well as with all organ support measures tested: (i) days without cardiovascular organ support including VA-ECMO and Impella (21 vs. 3 days, *p* = 0.0005), (ii) need for renal replacement therapy (22% vs. 56%, *p* < 0.0001), and (iii) rate of mechanical ventilation (74% vs. 90%, *p* = 0.04). Longitudinal analyses with determination of DPP3 at baseline and 72 h revealed that low and initially elevated but decreasing DPP3 concentrations were associated with a markedly reduced 30-day mortality, when compared to constantly high DPP3 concentrations (30% vs. 74%) (Figure).

**Conclusions:** Circulating DPP3 is a strong predictor of morbidity and mortality in cardiogenic shock. The results need to be validated in further cardiogenic shock cohorts, but otherwise hold strong promise for the upcoming clinical trials on the evaluation of the specific DPP3-antibody procizumab as new breakthrough therapy in cardiogenic shock.

**Reference**

1. Karakas M et al. Lancet Respir Med 10:247–54, 2022.

**Figure (abstract P088):**
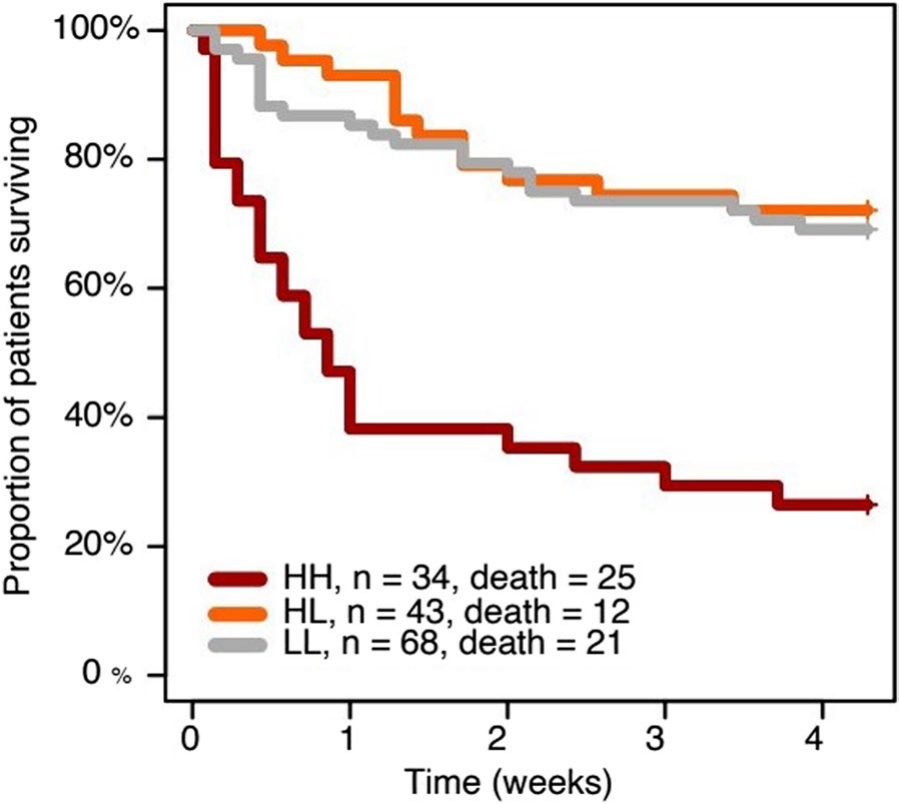
Kaplan–Meier analysis for the association of constantly high (HH = high-high), decreasing (HL = high-low), and constantly low (LL = low-low) DPP3 levels with mortality. DPP3 was determined at randomization and after 72 h.

## P089 Clinical observational study into mitochondrial oxygenation predicting ominous lactate in trajectory around abdominal surgery (COSMOPOLITAS)

### LWJM Streng, CJ de Wijs, S Riemens, FA Harms, D Sneiders, BPL Wijnhoven, RJ Stolker, JNM IJzermans, EG Mik

#### Erasmus MC, Anesthesiology, Rotterdam, Netherlands

*Critical Care* 2024, **28(Suppl 1):** P089

**Introduction:** COSMOPOLITAS studied the relationship between mitoPO_2_ and serum lactate in surgical patients. Mitochondrial oxygen tension in the skin (mitoPO_2_) can be measured by the COMET system (Photonics Healthcare B.V., Utrecht, The Netherlands). To enable its use, COMET relies on Alacare plasters (photonamic GmbH, Pinneberg, Germany) to induce protoporphyrin IX (PpIX) in the skin. The COMET technology uses the optical properties of PpIX to measure oxygen in the mitochondria. Serum lactate is a marker for hypoxia, and abnormally increased serum lactate is associated with adverse patient outcome. Thus, the existence of an association between mitoPO_2_ and lactate would demonstrate usability of mitoPO_2 _as clinical parameter.

**Methods:** In an observational clinical study in 40 patients undergoing major abdominal surgery, mitoPO_2_ was monitored peri-operatively, i.e. during surgery and stay on the ICU/PACU. Serum lactate was measured at least every 6 h. The relationship between mitoPO_2_ and lactate values was investigated using mixed model logistic regression.

**Results:** The predicting value of the area under the curve of mitoPO_2 _during 60 min [mitoPO_2_]_60_, versus increased lactate (> 2 mmol/L) in 474 pairs of lactate and mitoPO_2_ was analyzed. An odds-ratio of (OR) of 0.97 (*p* < 0.001) per unit [mitoPO_2_]_60_ (in mmHg) was found. An analysis of the intraoperative subset of lactate and mitoPO_2_ pairs (n = 220) resulted in and odds-ratio for [mitoPO_2_]_60_ of 0.93 (*p* < 0.002). A scatterplot and frequency distribution of the intraoperative subset are presented in the Figure.

**Conclusions:** A prolonged period of low mitoPO_2_ is associated with an increased risk of pathological serum lactate, and thus increased perioperative risk. Therefore, it can be concluded that mitoPO_2_ provides useful information about patient status, and could potentially be used as an early warning sign of patient deterioration or endpoint of resuscitation.

**Figure (abstract P089):**
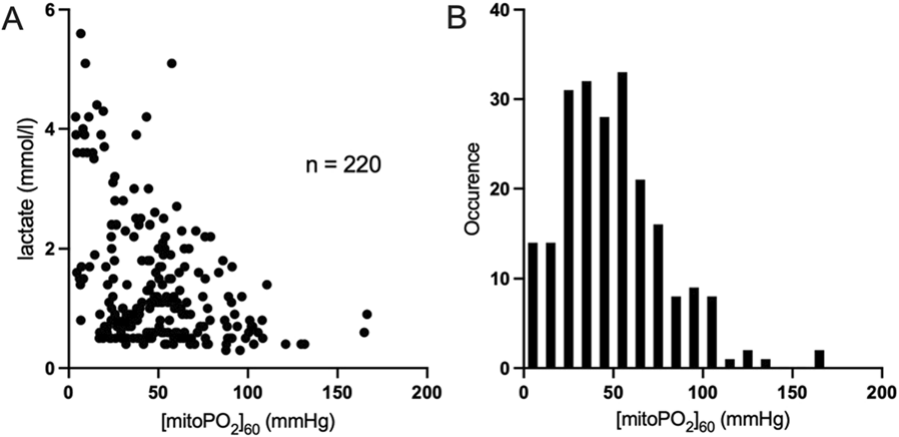
Intraoperative data. A and B: Scatterplot and frequency distribution of intraoperative data subset.

## P090 Mitochondrial oxygenation monitoring and acute kidney injury risk in cardiac surgery: a prospective cohort study

### CJ de Wijs^1^, LWJM Streng^1^, RJ Stolker^1^, M ter Horst^1^, EJ Hoorn^2^, EAF Mahtab^3^, EG Mik^1^, FA Harms^1^

#### ^1^Erasmus MC, Anesthesiology, Rotterdam, Netherlands, ^2^Erasmus MC, Internal Medicine, Rotterdam, Netherlands, ^3^Erasmus MC, Cardiothoracic Surgery, Rotterdam, Netherlands

*Critical Care* 2024, **28(Suppl 1):** P090

**Introduction:** Cardiac surgery-associated acute kidney injury (CSA-AKI) is a common complication of cardiac surgery and is associated with increased morbidity and mortality. Recent guidelines emphasize the need for new monitoring methods to facilitate targeted CSA-AKI prevention and treatment strategies. In-vivo mitochondrial oxygen tension (mitoPO_2_), which is semi-continuous and can be measured in real time, could potentially fulfill this role during cardiac surgery.

**Methods:** This prospective observational study examines the association between mitoPO_2_ < 20 mmHg and the risk of CSA-AKI. The study included 75 patients with an increased pre-operative CSA-AKI risk. All patients underwent coronary artery bypass grafting with cardiopulmonary bypass. MitoPO_2_ was measured intraoperatively and CSA-AKI was defined by the Kidney Disease: Improving Global Outcomes criteria. Post-hoc analyses were conducted for four additional mitoPO_2_ thresholds < 25, < 30, < 35, < 40 mmHg.

**Results:** Patients who developed CSA-AKI had significantly more intraoperative time with mitoPO_2_ below 20 mmHg, but this threshold was not associated with CSA-AKI risk (*p* = 0.06). Conversely, mitoPO_2_ below the other thresholds was associated with CSA-AKI risk, with the < 25 mmHg showing the highest odds ratio (OR). Every minute spent < 25 mmHg increased the risk of CSA-AKI by 0.7% (*p* = 0.02), visualized in the Figure, while every percentage increase in intraoperative time below this threshold raised the risk by 2.1% (*p* = 0.02). The percentage of time that mitoPO_2_ was < 25 mmHg remained an independent risk predictor when analyzed in a multivariate regression model (OR 1.02, 95% CI 1.00–1.04, *p* = 0.04).

**Conclusions:** This study highlights the association between mitoPO_2_ and the onset of CSA-AKI. Extended durations below the mitoPO_2_ threshold of 25 mmHg strongly correlate with an elevated CSA-AKI risk. Using mitoPO_2_ as a monitoring tool shows promise in potentially predicting and possibly preventing CSA-AKI when used as a treatment trigger in cardiac surgery patients.

**Figure (abstract P090):**
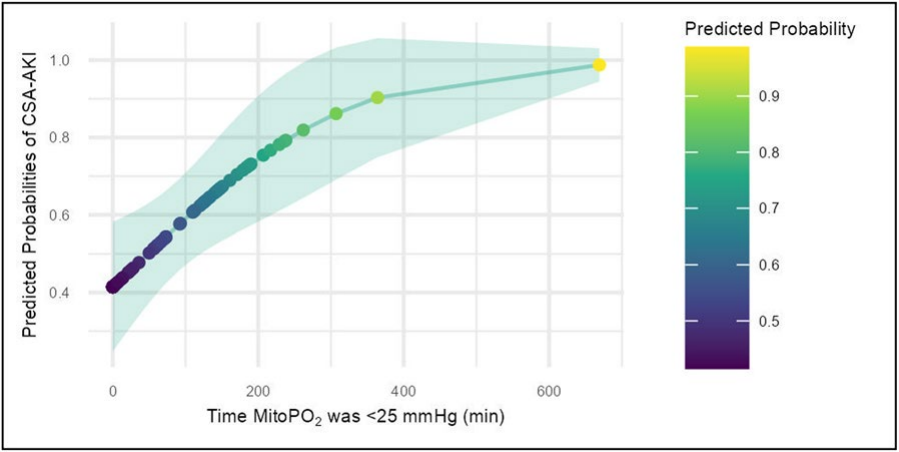
Predictive probabilities: cardiac surgery associated acute kidney injury versus time under mitoPO_2_ threshold (< 25 mmHg).

## P091 Averaged versus persistent reduction in urine output to define oliguria in critically ill patients

### C Monard^1^, NA Bianchi^1^, T Kelevina^1^, M Altarelli^1^, A Chaouch^2^, A Schneider^1^

#### ^1^Centre Hospitalier Universitaire Vaudois, Service de Médecine Intensive Adulte, Lausanne, Switzerland, ^2^University of Lausanne, Department of Epidemiology and Health Systems, Center for Primary Care and Public Health (Unisanté), Lausanne, Switzerland

*Critical Care* 2024, **28(Suppl 1):** P091

**Introduction:** We aimed to compare the incidence of oliguria and its association with outcomes according to whether it is determined using an average (mean hourly urinary output (UO) below threshold for the specified period) or a persistent method (all UO below threshold for the specified period).

**Methods:** We included all adults patients admitted to a tertiary ICU between 2010 and 2020 except for those on chronic dialysis or who declined consent. Hourly UO, clinical and socio-demographic data were extracted from electronic medical records, and 90-day mortality from the national death registry. We determined oliguria incidence according to each assessment method and for different thresholds. Performance of each method to predict 90-day mortality and acute kidney disease (AKD, defined by a 20% decrease in eGFR compared with baseline) at hospital discharge was calculated. The association between oliguria and 90-day mortality was explored with logistic regression models and quantified using marginal relative risks (MRR), stratified by admission type and controlling for age, SAPS II, Charlson index and admission year. Missing covariates were multiply imputed.

**Results:** Among the 15,253 patients analyzed, the average method identified oliguria more often than the persistent method (73 [95%CI 72–74] vs. 55 [95% CI 54–55] %) (Figure). It displayed a higher sensitivity for 90-day mortality prediction (85 [95% CI 84–86] vs. 70 [95% CI 69–72] %)and AKD at hospital discharge (87 [95% CI 85–88] vs. 72 [95% CI 70–74] %). However, its specificity was lower for both outcomes (30 [95% CI 29–31] % vs. 49 [95% CI 48–50] % and 30 [95%CI 29–31]% vs. 49 [95% CI 48–50] %). After adjusting for corrected SAPS II, Charlson comorbidities index, age and admission year, oliguria was, with both methods, associated with an attributable mortality of 4%.

**Conclusions:** The assessment method of oliguria has major diagnostic and prognostic implications, and the definition of oliguria should be standardized in this regard.

**Figure (abstract P091):**
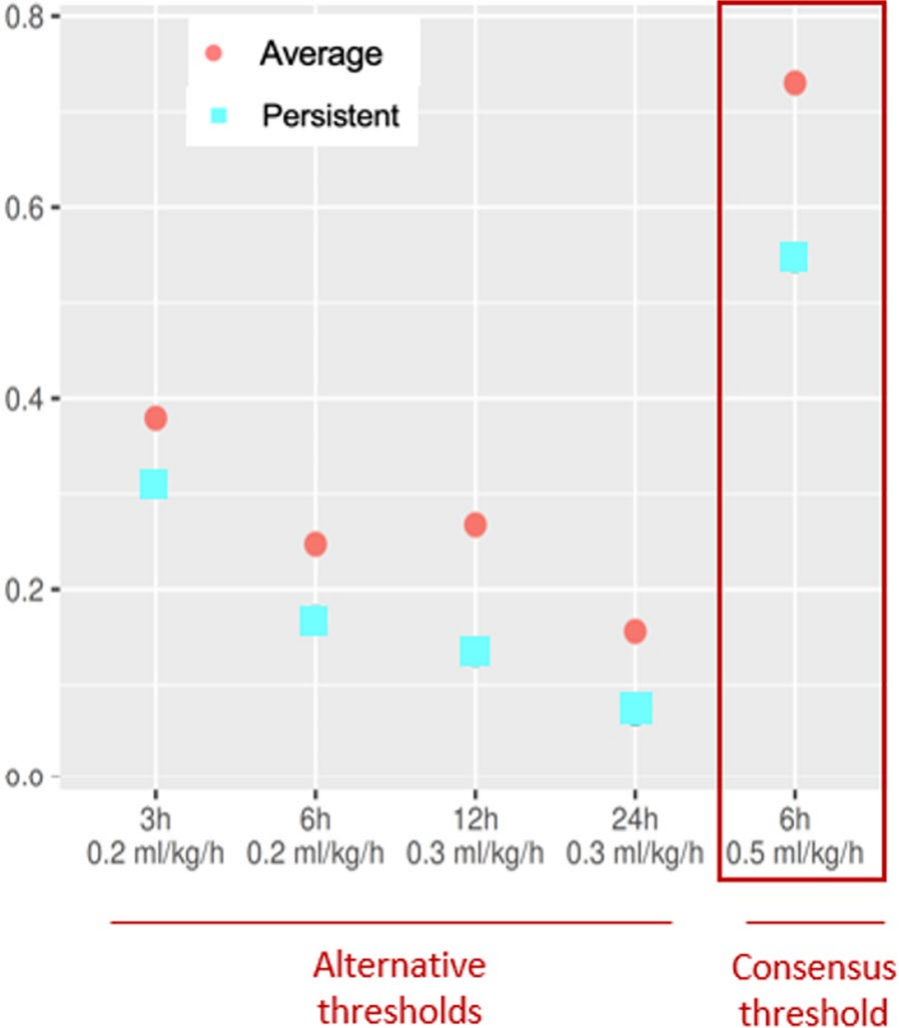
Incidence of oliguria considering an averaged (red) or a persistent (green) reduction in urine output under different thresholds (95% confidence interval are comprised within the size of the dot).

## P092 Acute kidney injury in critically ill patients with COVID-19: a single-center cohort analysis

### C Branco^1^, C Costa^1^, L Carvalho^2^, D Cabral^2^, A Gaspar^2^, C Candeias^2^, S Fernandes^3^, J Santos Silva^2^

#### ^1^Centro Hospitalar Universitário Lisboa Norte, Division of Nephrology and Renal Transplantation, Department of Medicine, Lisboa, Portugal, ^2^Centro Hospitalar Universitário Lisboa Norte, Intensive Care Department, Lisboa, Portugal, ^3^Faculdade de Medicina da Universidade de Lisboa, Clínica Universitária Medicina Intensiva, Lisboa, Portugal

*Critical Care* 2024, **28(Suppl 1):** P092

**Introduction:** Acute kidney injury (AKI) has been reported as a frequent complication of critical COVID-19, and renal involvement has been associated with poor outcomes [1]. We aimed to evaluate the occurrence of AKI, the use of renal replacement therapy (RRT), patient and kidney outcomes, and the risk factors associated with AKI in critical COVID-19.

**Methods:** Single-center, retrospective cohort analysis of patients with critical COVID-19 in a university hospital in Portugal. AKI was defined according to KDIGO serum creatinine (sCr) and urinary output (UO) criteria. Multivariable logistic regression analysis was used to explore the risk factors for developing AKI and to assess the association between AKI and ICU mortality.

**Results:** Of 476 evaluated patients, 60% (n = 286) had AKI, of which 93.7% fulfilled UO criteria. AKI could be established by sCr in 59.1% of patients, while 40.9% had exclusively UO criteria. Age above 60 years (OR 2.9), obesity (OR 3.1), a higher SAPS II score (OR 1.03), use of mechanical ventilation at day 1 of ICU stay (OR 5.2) and previous chronic renal disease (OR 5.6) were associated with an increased risk for AKI. Overall mortality in this cohort was 24.4% (32.9% in patients with AKI and 11.6% in patients without AKI). KDIGO stages 2 (OR 3.7) and 3 (OR 10.6) were associated with ICU mortality (*p* < 0.001). Of note, patients diagnosed only by UO criteria did not have an increased ICU mortality. RRT was used in 16% (n = 47) of patients. Among survivors, only 2 patients persistently needed RRT at ICU discharge. Global kidney function recovery was 95.3% (n = 183).

**Conclusions:** In a single-center cohort analysis of 476 critically-ill COVID-19 patients, 60% had AKI. This was driven by the occurrence rate of AKI defined by UO criteria. KDIGO stages 2 and 3 were associated with ICU mortality. Most of the patients with AKI had recovered by the time of ICU discharge. Future studies should explore long term kidney outcomes.

**Reference**

1. Gabarre P et al. Intensive Care Med. 2020;46:1339–1348.

## P093 Height-adjusted phase angle for 24 h-creatinine clearance estimation in immunocompromised ICU patients

### P Vargas^1^, N Dreyse^1^, R Lopez^1^, M Cano-Cappellaci^3^, J Guerrero^3^, M San Martin^4^, M Barriga^4^, S Aracena^4^, M Roa^1^, J Montes^1^

#### ^1^Clínica Alemana de Santiago Unidad de Paciente Crítico, Unidad de Paciente Critico, Santiago, Chile, ^2^Universidad de Chile, Physical Therapy Department Universidad de Chile, Santiago, Chile, ^3^Clínica Alemana de Santiago Unidad de Paciente Crítico, Instituto de Ciencias Biomédicas, Facultad de Medicina, Universidad de Chile, Santiago, Chile, ^4^Clinica Alemana, Servicio de Urgencias Clínica Alemana, Santiago, Chile

*Critical Care* 2024, **28(Suppl 1):** P093

**Introduction:** We propose the height-adjusted phase angle (HAPA), derived from bioimpedance analysis (BIA), to be considered as a weight-independent surrogate for 24-h urinary creatinine excretion (UCE-24 h). This approach allows for a more precise assessment of 24-h creatinine clearance (CrCl-24 h) compared to the Cockroft-Gault (CG) formula, particularly in circumstances requiring drug dosage adjustments in renal excreted drugs in immunocompromised ICU patients.

**Methods:** This prospective observational cohort study (Conducted in a single center), included immunocompromised ICU adults undergoing BIA (mBCA 525, Seca, Germany) and UCE/CrCl-24 h measurements over a 3-year period. Clinical and anthropometric variables, along with serum creatinine (SCr), were registered. Data were presented using median and p25–p75.

**Results:** A total of 105 measurements were collected, with 74.3% representing male subjects with a median age of 62 years (41–69), CrCl-24 h at 60 mL/min (42–89), and a PA of 3.4° (3.0–4.2). Non-oncological immunosuppressed, hematooncological, and patients with solid tumors accounted for 51.4%, 34.3%, and 14.3%, respectively. The initial 75 measurements were allocated to the training cohort, showing a significant linear correlation between HAPA and UCE-24 h, as well as HAPA/SCr and CrCl-24 h (r = 0.67 and r = 0.81 respectively). Through stepwise linear regression, a predictive formula for CrCl-24 h was develop using HAPA and SCr. The model underwent validation with the remaining 30 measurements (validation cohort), resulting in an R^2^ of 0.77. The sensitivity to predicting CrCl-24 h below 60 mL/min was higher compared to the CG formula, with 83% and 67% sensitivity, respectively.

**Conclusions:** HAPA, as a weight-independent surrogate for UCE-24 h, affords a more precise CrCl-24 h estimation compared to CG, particularly exhibiting higher sensitivity within renal dosage adjustment ranges for immunocompromised ICU patients.

**Figure (abstract P093):**
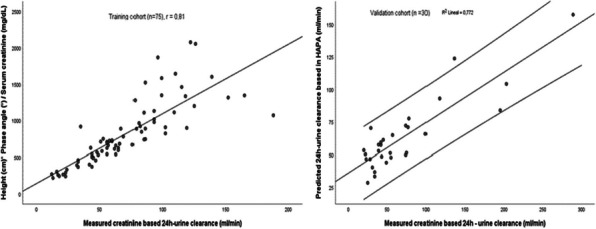
Left panel: correlation plot between height-adjusted phase angle/serum creatinine (cm*/mg/dL) and measured 24 h creatinine clearance (mL/min) in training cohort. Right panel: correlation plot between measured 24 h-creatinine clearance (mL/min) and predicted 24 h-creatinine clearance based in height adjusted phase angle (mL/min) in validation cohort. Parallels lines represent 95% confidence interval.

## P094 Serum creatinine ratio as predictor of patient-centered long-term outcome after cardiac surgery

### P Pickkers^1^, MC Kraan^2^, K Pardali^2^, A Shaw^3^, A Shenoi^4^, J Gerss^5^, A Zarbock^6^

#### ^1^Radboudumc, Intensive Care, Nijmegen, Netherlands, ^2^AM-Pharma, AM-Pharma, Utrecht, Netherlands, ^3^Cleveland Clinic, Intensive Care and Resuscitation, Cleveland, USA, ^4^Boston Strategic Partners, Statistics, Boston, USA, ^5^Institute of Biostatistics and Clinical Research, University of Münster, Münster, Germany, ^6^University Hospital Münster, Dept. of Anesthesiology, Intensive Care and Pain Medicine, Münster, Germany

*Critical Care* 2024, **28(Suppl 1):** P094

**Introduction:** Acute kidney injury (AKI) in patients undergoing cardiac surgery relate to the development of major adverse kidney events up to day 90 (MAKE90). We tested whether the serum creatinine ratio (sCrRatio), defined as the ratio between the highest serum creatinine in the first five post-operative days and the pre-operative measurement, can predict major adverse kidney events up to day 90 (MAKE90).

**Methods:** A development cohort and an external validation cohort with patients undergoing cardiac surgery were investigated.

**Results:** In the development cohort (n = 6576), 54.2% of patients developed AKI by post-operative Day 5 using the kidney disease global outcomes (KDIGO) criteria, and in the validation cohort (n = 554) this was 28.4%. The cut-off value (where sensitivity was equal to specificity) for sCrRatio was 1.25 in the development cohort. The incidence of MAKE90 in the development cohort was 16.7% and in the validation cohort 24.4%.In the development cohort, the positive and negative predictive value (PPV and NPV (95% confidence interval)) for patients with AKI to develop MAKE90 were 25% (23–26) and 92% (91–93), for sCrR higher than cut-off they were 33% (31–35) and 90% (90–91). In the validation cohort they were 42% (34–51) and 83% (79–88) and 47% (38–54) and 81% (2.37–6.03), respectively (Table).

**Conclusions:** Calculation of the sCrRatio is simple and identifies patients at risk for meeting the MAKE criteria 90 days after cardiac surgery.

**Table (abstract P094):** Performance characteristics of all measurements

	KDIGO-AKI (development)	sCrRatio (development)	KDIGO-AKI (validation)	sCrRatio (validation)
Sensitivity	78% (95% CI 75–80%)	70% (95% CI 67–73%)	53% (95% CI 43–63%)	42% (95% CI 34–51%)
Specificity	52 (95% CI 50–53%)	67% (95% CI 66–68%)	77% (95% CI 72–81%)	84% (95% CI 79–88%)
PLR	1.61 (95% CI 1.55–1.68)	2.11 (95% CI 2.00–2.23)	2.26 (95% CI 1.72–2.96)	2.61 (95% CI 1.87–3.63)
NLR	0.43 (95% CI 0.39–0.48)	0.45 (95% CI 0.41–0.49)	0.61 (95% CI 0.50–0.76)	0.69 (95% CI 0.59–0.81)
PPV	25% (95% CI 23–26%)	33% (95% CI 31–35%)	42% (95% CI 34–51%)	47% (95% CI 38–55%)
NPV	92% (95% CI 91–93%)	90% (95% CI 90–91%)	83% (95% CI 79–88%)	81% (95% CI 79–84%)
Accuracy	56% (95% CI 55–57%)	67% (95% CI 66–75%)	71% (95% CI 66–75%)	73% (95% CI 69–78%)

## P095 Biomarkers to predict the need for renal replacement therapy in severe acute kidney injury (bioMARkers in acute Kidney injury settings to predict interventions and outcomes: the MARKISIO study)

### K Chaïbi^1^, A Picod^2^, S Tubiana^3^, V Jullien^4^, S Placier^5^, J Quenot^6^, F Azibani^2^, A Mebazaa^2^, D Dreyfuss^5^, S Gaudry^5^

#### ^1^Avicenne Hospital, Intensive Care Unit, Bobigny, France, ^2^Hôpital Lariboisière, INSERM UMR-S 942 MASCOT – Paris – Cité University, Paris, France, ^3^APHP Hôpital Bichat, Centre de Ressources Biologiques, Paris, France, ^4^APHP Hôpital Jean Verdier, Service de Pharmacologie, Bondy, France, ^5^Hôpital Tenon, Common and Rare Kidney Diseases, Sorbonne Université, INSERM, UMR-S 1155, Paris, France, ^6^François Mitterrand University Hospital, Réanimation Polyvalente, Dijon, France

*Critical Care* 2024, **28(Suppl 1):** P095

**Introduction:** Predicting the need for renal replacement therapy (RRT) in severe acute kidney injury (AKI) remains challenging. The usefulness of biomarkers has been explored, but previous studies lacked clarity on RRT indications and were conducted before the release of key randomized controlled trials (RCTs) that refined RRT initiation criteria. The aim of the MARKISIO study was to assess the performance of a panel of biomarkers in predicting the criteria for RRT initiation in severe AKI patients.

**Methods:** This ancillary study of the AKIKI2 trial included critically ill patients with severe AKI. Blood and urine samples were collected within 12 h after the occurrence of KDIGO 3 AKI. The primary endpoint was the occurrence of RRT initiation criteria within 72 h after the onset of severe AKI. These criteria were rigorously defined based on recent RCTs on RRT timing. The study analyzed a panel of routine biomarkers (pH, serum potassium, and serum creatinine) and novel biomarkers (urinary C–C motif chemokine ligand 14 [CCL14], urinary Kidney Injury Molecule 1 [KIM1], nicotinamide and its metabolites, circulating dipeptidyl peptidase [cDPP3] concentration and plasma proenkephalin A 119–159).

**Results:** Among the 256 patients, 101 (39%) met at least one criterion for RRT initiation or died within 72 h after the inclusion. None of the individual biomarkers or their combinations demonstrated satisfactory predictive performance for the primary endpoint. The best biomarker combination was ‘pH’, ‘serum creatinine concentration’ and ‘urinary CCL-14’ with an area under the receiver operating characteristic curve (AUC) (95% CI) of 0.72 (0.65–0.78). In an exploratory analysis, urinary CCL14 showed promise in patients with toxic-induced kidney injury (AUC 0.74 [0.57–0.90]).

**Conclusions:** No biomarker or combination of biomarkers demonstrated conclusive predictive accuracy for the need for RRT in severe AKI patients.

## P096 User experience (UX) study to evaluate clinical decision support system prototype supporting continuous kidney replacement therapy in a simulated ICU environment

### LM Kunz^1^, M Metzger^2^, C Schäfer^3^, R Pohlmeier^4^, J Petrovic^5^, MT Nosch^6^

#### ^1^Fresenius Medical Care Deutschland GmbH, Critical Care & Ventures, Bad Homburg, Germany, ^2^Marienhospital Bottrop gGmbH, Information Technology Department, Bottrop, Germany, ^3^Marienhospital Bottrop gGmbH, Nursing for Intensive Care Medicine, Bottrop, Germany, ^4^Fresenius Medical Care Deutschland GmbH, Global Medical Office, Bad Homburg, Germany, ^5^Fresenius Medical Care Deutschland GmbH, Market Access & Health Economics, Bad Homburg, Germany, ^6^Marienhospital Bottrop gGmbH, Anesthesiology and Intensive Care Medicine, Bottrop, Germany

*Critical Care* 2024, **28(Suppl 1):** P096

**Introduction:** The increasing amount of data routinely collected on ICUs poses a challenge for clinicians [1] which is aggravated with data-heavy therapies like continuous kidney replacement therapy (CKRT). We developed the CKRT Supporting Software Prototype (CKRT-SSP), a clinical decision support system for use before, during and after CKRT. The aim of this user experience (UX) study was to prospectively evaluate CKRT–SSP on usability, user experience, and workload in a simulated ICU setting.

**Methods:** We simulated CKRT treatments in a fully equipped ICU box and evaluated CKRT-SSP with validated questionnaires: System Usability Scale (SUS) [2] and User Experience Questionnaire (UEQ) [3]. Further, a modified NASA-TLX (task load index) [4] compared workload before and after using CKRT-SSP. A total of 12 clinicians and nurses participated in this study.

**Results:** The SUS reached a median value of 87.5 for the CKRT-SSP, reflecting excellent usability. In the UEQ, CKRT-SSP scored clearly positive in the dimension attractiveness and the three task related dimensions perspicuity, efficiency, and dependability (95% CI fully > 0.8). For the two non-task related dimensions stimulation and novelty there was a positive trend (mean > 0.8, while lower limit of 95% CI < 0.8). The modified NASA-TLX showed a significant workload reduction in physical demand, effort, and frustration (Figure).

**Conclusions:** CKRT-SSP is a promising tool for improving workload on ICUs and specifically application of CKRT. We obtained valuable insights for further user centric development.

**References**

1. Manor-Shulman O et al. J Crit Care. 2008;23:245–250
2. Brooke J, in Usability Evaluation in Industry 1996, pp:189–194
3. Laugwitz B et al. Lecture Notes in Computer Science 2008;5298:63–76
4. Bustamante EA et al. Proceedings of the Human Factors and Ergonomics Society Annual Meeting 2008;52:1522–1526

**Figure (abstract P096):**
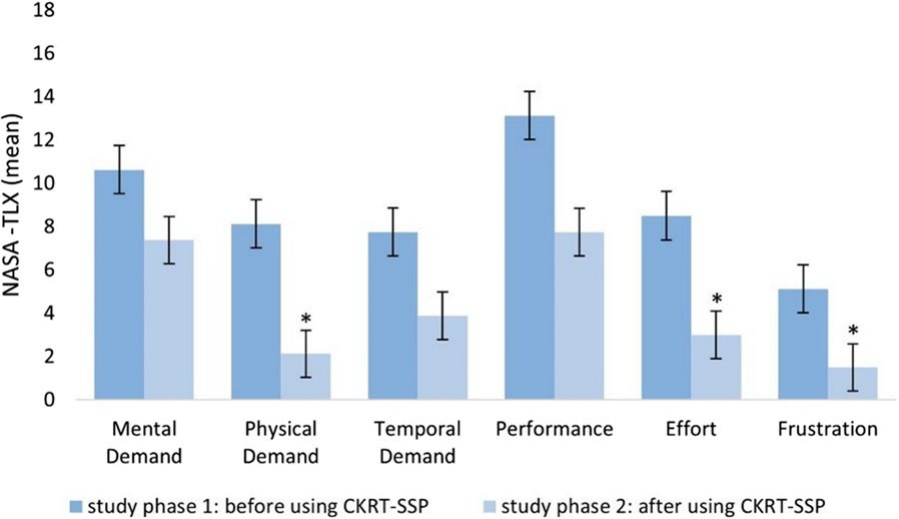
Results of the modified NASA-TLX before and after using CKRT-SSP (mean and SD), *indicates statistically significant reduction by Mann–Whitney-U test.

## P097 Troponin clearance via continuous renal replacement therapies in the ICU

### S Boyd^1^, S Mason^1^, S Griffin^1^, C Keane^1^, E Deasy^2^, M Donnelly^1^, J Chevarria^3^, DM D´Arcy^4^, YP Kelly^5^

#### ^1^Tallaght University Hospital, Department of Critical Care, Dublin 24, Ireland, ^2^Tallaght University Hospital, Department of Pharmacy, Dublin 24, Ireland, ^3^Tallaght University Hospital, Department of Nephrology, Dublin 24, Ireland, ^4^Trinity College Dublin, School of Pharmacy and Pharmaceutical Sciences, Dublin 2, Ireland, ^5^Tallaght University Hospital, Intensive Care, Dublin 24, Ireland

*Critical Care* 2024, **28(Suppl 1):** P097

**Introduction:** The aim of this prospective cohort observational study was to compare the removal of cardiac troponin T via continuous venovenous haemofiltration (CVVH), continuous venovenous haemodialysis (CVVHD) and continuous venovenous haemodiafiltration (CVVHDF) as a function of circulating blood troponin levels in critically ill patients.

**Methods:** This was a single-centre, prospective observational study conducted at Tallaght University Hospital (Dublin, Ireland). Adult patients admitted to ICU and commenced on continuous renal replacement therapy (CRRT) were included. All included patients required a Troponin T level of greater than 50 ng/L. All patients were required to have commenced CRRT at least four hours before the first sample was taken. The aim was to take three serum samples per patient every 24 h, i.e. over a total of 72 h, with simultaneous sampling of the waste effluent of the RRT for troponin. Our primary outcome measure was estimated troponin clearance according to CRRT modality.

**Results:** We found no significant difference in estimated troponin clearance according to CRRT modality; with an overall median troponin clearance of 2.6 ng/kg/hour. The percentage of troponin clearance was statistically significantly higher for CVVH compared to CVVHD and CVVHDF (17 vs. 14 vs. 12% respectively; *p* = 0.008), though this was not felt to be clinically significant.

**Conclusions:** In this a single-centre, prospective observational study, we measured simultaneous blood and effluent troponin levels in patients on either CVVHDF, CVVHD or CVVH to compare estimated troponin clearance between the three CRRT modalities. We found no significant difference in estimated troponin clearance according to CRRT modality. Our results show that clearance of troponin on CRRT is generally small across all modalities and that therefore ongoing treatment with CRRT should not significantly impact our interpretation and tracking of troponin T results in patients with concern for acute coronary syndrome.

## P098 Hypothermia associated with high-volume continuous renal replacement therapy: dependence on the method of warming blood and dialysis fluids

### I Yovenko, D Gavrichenko

#### Medical Home Odrex, Department of Anesthesiology and Intensive Care, Odessa, Ukraine

*Critical Care* 2024, **28(Suppl 1):** P098

**Introduction:** Up to 50% of critically ill patients require renal replacement therapy (RRT) due to acute kidney injury and multiple organ failure. Extended high-volume continuous renal replacement therapy (HV-CRRT) can significantly improve treatment outcomes for patients with septic and nonseptic multiorgan failure by effectively eliminating medium and large toxic molecules [1, 2]. It promotes better hemodynamic tolerability of renal replacement therapy, modulation of the inflammatory and immune response, and has a positive effect on outcomes and mortality.

**Methods:** During 2021–2023, we provided 97 sessions of HV-CRRT in patients with multiple organ failure in our intensive care unit. We used HV-CRRT with a flow rate of 35–50 mL/kg/h. In 13 patients, we used the Prismaflex device, where extracorporeal blood warming occurs by heating the return line (blood returning to the patient). In 21 patients, we used the multiFiltrate device, which uses built-in heating elements to warm dialysate and substitute solutions. We monitored hemodynamics, water-electrolyte and acid–base balance, and axillary body temperature of the patients. To assess the effectiveness of therapy, we assessed the duration of patients’ stay in the ICU, their functional indicators of the cardiovascular and respiratory systems, and laboratory dynamics of markers of liver, kidney, and hemostasis function.

**Results:** The effectiveness of CRRT was identical in both groups of patients. However, in the group where blood warming was used by heating the return line, the procedure was accompanied by the development of stable mild hypothermia in the range of 34–36 °C, which remained stable despite the use of additional methods of external warming of patients.

**Conclusions:** Hypothermia in critically ill patients receiving high-volume continuous renal replacement therapy is more common with the use of external rewarming systems.

References

1. Pedreros-Rosales C et al. Toxins*.* 2023;15:531.
2. Rimmelé T et al. Anesthesiology. 2012;116:1377–87.

## P099 The use of the advanced organ support (ADVOS) hemodialysis system in cardiosurgical patients with end-stage shock helps to correct acidosis and to reduce vasopressor needs

### V Walter^1^, E Hinrichs^1^, J Dreyer^1^, T Alloush^1^, A Perez Ruiz de Garibay^2^, G Warnecke^1^, W Sommer^1^, A Thiem^1^, B Panholzer^3^

#### ^1^Universitätsklinikum Schleswig-Holstein Campus Kiel, Kiel, Germany, ^2^ADVITOS GmbH, München, Germany, ^3^Universitätsklinikum Schleswig-Holstein Campus Kiel, Cardiac Surgery, Kiel, Germany

*Critical Care* 2024, **28(Suppl 1):** P099

**Introduction:** The ADVOS hemodialysis system corrects acidosis in patients with multiple organ failure (MOF) [1] and it is intriguing if its application in cardiosurgical patients with end-stage shock may aid to restore the homeostasis and reduce the need of vasopressors. Thus, we aim to evaluate the effect of ADVOS in these patients since established therapies like RRT or extracorporeal life support (ECLS) improve prognosis, but disease progression with metabolic disruption, refractory acidosis and subsequent deterioration of vasoplegia remains challenging.

**Methods:** Patients treated with ADVOS for MOF and refractory shock that received at least 2 consecutive sessions from 11/2021 to 09/2023 were included in this retrospective analysis. The primary objective was the reduction of vasoactive inotropic score (VIS) after 48 h. The course of hemodynamic, hepatic, renal, ventilation and blood gas parameters as well as mortality rates at 28 days and ICU discharge were documented.

**Results:** 22 patients (77% male, median age 59,) with a median SOFA Score of 15 received 110 ADVOS sessions (median blood flow and duration of 150 mL/min and 23 h, respectively). At 48 h, a significant increase of blood pH (7.33–7.44, *p* = 0.001), bicarbonate (22.6–26.8 mmol/L, *p* < 0.001) and base excess (− 3.2 to 2.4 mmol/L, *p* > 0.001) was observed. A significant reduction of median noradrenaline doses (0.470–0.180 µg/mL/kg, *p* = 0.009) and VIS (59–21, *p* = 0.007) was achieved too (Figure). No device-related adverse events were observed. 28-day and ICU mortality was 55% and 59%, respectively. Considering an expected mortality > 90% [2], this resulted in a SOFA-Score standardized mortality ratio of 0.68 (95% CI 0.31–1.06), an absolute risk reduction of 29% and a number needed to treat of 3.5.

**Conclusions:** In cardiosurgical patients with end-stage shock, ADVOS was safe and feasible, and significant hemodynamic improvement could be observed.

**References**

1. Fuhrmann V et al. Medicine (Baltimore). 2021;100:e24653
2. Ferreira FL et al. JAMA. 2001;286:1754–8.

**Figure (abstract P099):**
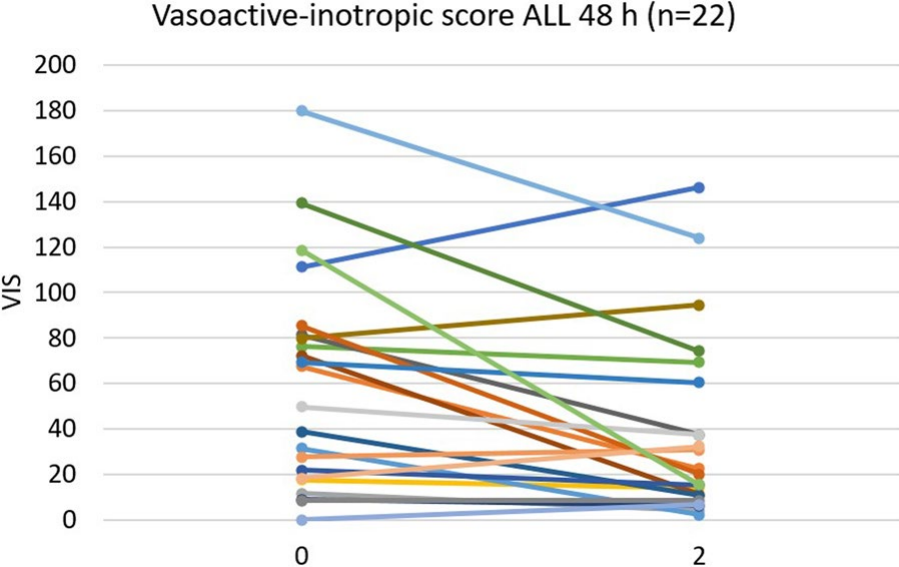
Vasoactive-inotropic score course in the first 48 h.

## P100 Regional citrate anticoagulation (RCA) for CVVHD in metformin associated lactic acidosis (MALA) patients. Comparison with population affected by severe metabolic acidosis due to isolated renal failure (RF)

### A Casazza^1^, E Bellazzi^2^, D Ciprandi^2^, A Poretti^2^, R Preda^2^, D Spagna^2^, MP Storti^2^, R Vanzino^2^

#### ^1^Ospedale Civile di Vigevano ASST Pavia, Anaesthesia and Intensive Care Vigevano, Vigevano, Italy, ^2^Ospedale Civile di Vigevano ASST Pavia, Vigevano, Italy

*Critical Care* 2024, **28(Suppl 1):** P100

**Introduction:** CRRT with RCA is progressively wider used in ICU patients. RCA is however controversial and potentially dangerous in shock, severe or worsening lactic acidosis and metformin intoxication due to possible citrate metabolism impairment [1].

**Methods:** We observed pH and BE increase rate and Ca_tot_/Ca^++^ ratio during the first 48 CRRT hours in 13 pts admitted to our ICU over 1 year for MALA (7) and severe acidosis due to isolate RF (6) and treated with RCA-CVVHD using hypertonic 4% citrate solution. We analyzed data by Wilcoxon test.

**Results:** CRRT mean duration was 43 in MALA versu 40 h in RF group. Mean blood flow was 127 in MALA and 125 mL/min in RF pts. Dialysate flow was respectively 2285 and 2416 mL/h. Mean pH and BE at admission were in MALA 6.99 and − 24 and in RF 7.18 and − 15 (*p* = 0.001). During first 6 h, pH increased by 0.13 in MALA and 0.18 in RF but faster in MALA than in RF pts in the next 6 h (*p* > 0.05). BE increased by 3.9 in MALA and 6.3 in RF in the first 6 h and by 7.4 and 4.5 in the next 6 h (*p* > 0.05) (Figure). Ca_tot_/Ca^++^ ratio was significantly higher in MALA group (*p* = 0.003), mostly during the first 12 RRT hours. 6 MALA pts needed Ca infusion rate increase. Nobody needed citrate infusion variation or develop citrate accumulation with Ca_tot_/Ca^++^  > 2.5 or acidosis worsening. All pts were discharged alive from ICU, 5 pts in MALA and 2 in RF group show renal function recovery.

**Conclusions:** In our series concentrated citrate solution RCA seemed to be safe and effective in MALA treatment. Acidosis improvement was delayed only in the very early treatment stage without reaching statistical significance. At the same time, in MALA pts, Ca_tot_/Ca^++^ was higher probably due to a certain degree of citrate metabolism impairment but stayed always below 2.5 [2]. Careful monitoring and strict adherence to citrate and Ca infusion protocols are effective to obtain treatment safety.

**References**

1. Pistolesi V et al. J Anesth Analg Crit Care. 2023;3:7–20
2. Brunoni B et al. Blood Purif 2023;52:802–811

**Figure (abstract P100):**
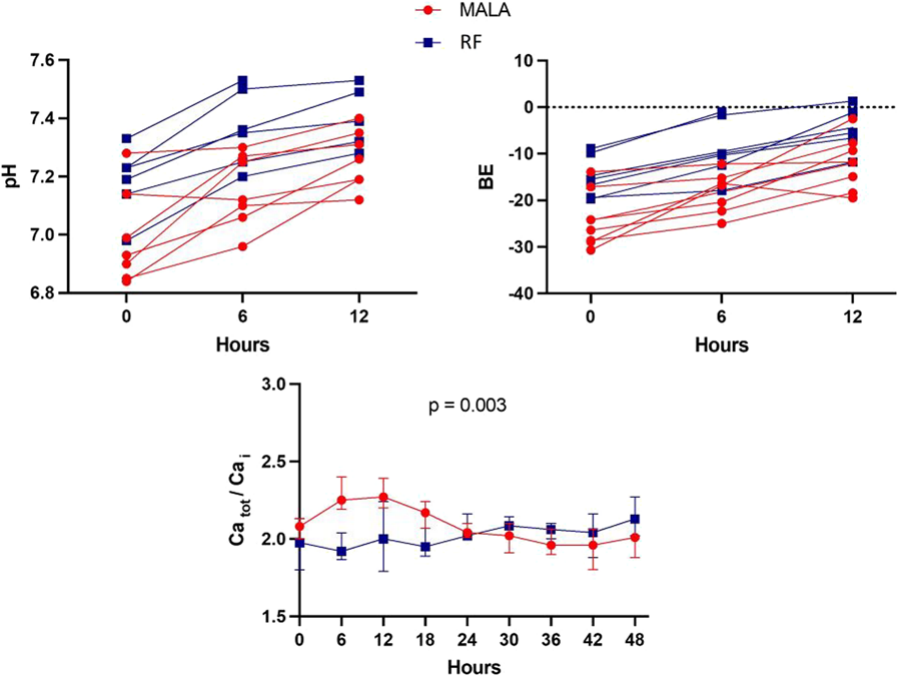
Results.

## P101 Predictors of failure in withdrawing continuous renal replacement therapy in the ICU

### JM Gil Perdomo^1^, I Gich Saladich^2^, A Núñez Reiz^1^, M Sánchez García^1^, E Calvo Manuel^3^, F Ortuño Andériz^1^

#### ^1^Hospital Clínico San Carlos, Intensive Care Unit, Madrid, Spain, ^2^Hospital de la Santa Creu i Sant Pau, Clinic Epidemiology and Public Health, Barcelona, Spain, ^3^Hospital Clínico San Carlos, Internal Medicine, Madrid, Spain

*Critical Care* 2024, **28(Suppl 1):** P101

**Introduction:** The criteria for initiating continuous renal replacement therapy (CRRT) in patients with AKI are well established, but not for its withdrawal. The main objective of our study was to identify clinical and analytical variables on the day of CRRT withdrawal and two days after it, that could predict the failure of discontinuing the therapy and serve as a tool to clinical decision making.

**Methods:** Prospective observational study in 55 patients with AKI of any cause in a 38-bed polyvalent ICU. The failure of the withdrawal of CRRT was defined as the need to restart therapy within 7 days after withdrawal. We collected clinical, analytical and ultrasound variables of interest on the day of removal (day 0) and 2 days after removal (day + 2) the CRRT. Through a logistic regression model and the use of ROC curves, the model with the best performance was determined.

**Results:** CRRT withdrawal was successful in 30/55 patients. Patients in whom CRRT withdrawal failed had a lower diuresis on day 0, a worse 2-h (2-h) urine creatinine clearance, a greater plasma creatinine ratio (day + 2/day 0) and a longer duration of CRRT. The area under the curve (AUC) to predict failure in CRRT withdrawal of diuresis with no use of diuretics was 0.815 (95% CI 0.593–1.000), optimal cut-off of 935 mL/day, of diuresis with the use of diuretics was 0.712 (95% CI 0.550–874), optimal cut-off of 1815 mL/day, of 2-h creatinine clearance was 0.81 (95% CI 0.67–0.95), optimal cut-off of 16.7 mL/min, of creatinine ratio was 0.86 (95% CI 0.75–0.97), optimal cut-off of 1.65 (95% CI 1.27–1.59) and of duration of CRRT was 0.68 (95% CI 0.53–0.82) (Table). The combination of the four variables obtained the best performance with an AUC of 0.92 (0.83–1.00).

**Conclusions:** In this prospective study we found that the combined use of clinical and analytical variables on day 0 and day + 2 of withdrawal of renal replacement therapy is a useful tool to predict failure of discontinuation of CRRT and could help in clinical decision making.

**Table (abstract P101):** Multivariate analysis of the relevant clinical and analytical variables, obtained on day 0 and + 2 of discontinuation of CRRT

	OR (95% CI)	Model	Area under de curve	*p* value
Day 0 diuresis (dL)	0.93 (0.89–0.98)	A	0.73 (0.60–0.86)	0.003*
2-h creatinine clearance (mL/min)	0.95 (0.90–0.99)	B	0.81 (0.67–0.95)	0.001*
Plasma creatinine ratio (day + 2/day 0)	37.89 (3.67–391.05)	C	0.86 (0.75–0.97)	0.001*
Duration of CRRT (days)	1.29 (1.03–1.64)	D	0.68 (0.53–0.82)	0.027*
		A + B + C	0.88 (0.77–1.00)	0.001*
		A + B + C + D	0.92 (0.83–1.00)	0.001*

Creatinine ratio (day +2/day 0): the division between the plasma creatinine on day +2 after withdrawal by the plasma creatinine obtained on the day of withdrawal; *statistically significant

## P102 Predictive factors of success after weaning attempt of RRT (deciding on patients orientations after renal replacement therapy stopping: the DOORS study)

### Y Akrour^1^, S Gaudry^1^, G Louis^2^, L Martin Lefevre^3^, D Titeca-Beauport^4^, B La Combe^5^, J Quenot^6^, D Dreyfuss^1^, K Chaïbi^7^

#### ^1^Hôpital Tenon, Common and Rare Kidney Diseases, Sorbonne Université, INSERM, UMR-S 1155, Paris, France, ^2^CHR Metz-Thionville Hôpital de Mercy, Réanimation Polyvalente, Metz-Thionville, France, ^3^CHR Départementale La Roche Sur Yon, Réanimation Polyvalente, La Roche Sur Yon, France, ^4^CHU d’Amiens Picardie, Réanimation Polyvalente, Amiens, France, ^5^CH de Bretagne Sud, Réanimation Polyvalente, Lorient, France, ^6^François Mitterrand University Hospital, Réanimation Polyvalente, Dijon, France, ^7^Avicenne Hospital, Intensive Care Unit, Bobigny, France

*Critical Care* 2024, **28(Suppl 1):** P102

**Introduction:** Renal replacement therapy (RRT) is a life-saving intervention for critically ill patients with severe acute kidney injury (AKI). While evidence have emerged for the initiation of RRT, the discontinuation of RRT remains an area lacking in robust evidence. We aimed to identify the variables that are predictive of successful discontinuation of RRT in patients with severe AKI managed under a conservative approach, and to formulate a clinically applicable scoring system to guide decision-making in this context called the UNDERSCORE.

**Methods:** In this *post-hoc* analysis of two large randomized controlled trials (AKIKI and AKIKI2), we included adult ICU patients with stage 3 AKI who underwent RRT (as a standard delayed initiation strategy) and had an attempt to discontinue RRT. The primary outcome was successful discontinuation of RRT, defined as no need for a new RRT session within seven days after the initial discontinuation. A logistic regression model was developed to predict the primary outcome, leading to the formation of the UNDERSCORE tool (Figure).

**Results:** Among 554 patients managed with a conservative RRT approach, weaning attempts were made in 180 patients; of these, successful discontinuation of RRT was achieved in 101 (56%) patients. Factors such as urine output, mechanical ventilation and vasopressive agent the day after the weaning attempt, duration of RRT, and septic shock at admission were identified as significant predictors [SG1]. The UNDERSCORE tool, which incorporates these predictors, showed high predictive accuracy with an area under the receiver operating characteristic curve (AUROC) of 0.85.

**Conclusions:** The DOORS study identified evidence-based predictors for successful discontinuation of RRT initiated under a conservative approach. It also introduced the UNDERSCORE as a valuable tool for clinicians. This score can assist in the decision-making process for discontinuing RRT in ICU patients with AKI, potentially reducing complications and healthcare costs.

**Figure (abstract P102):**
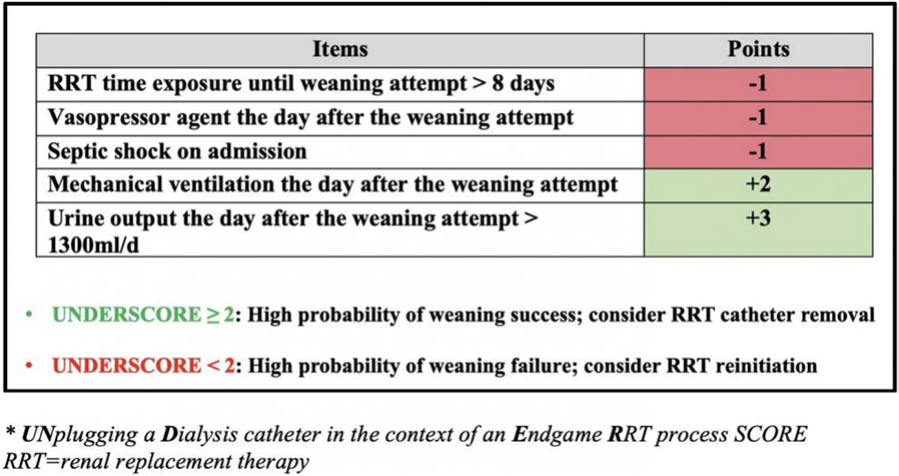
The UNDERSCORE* for predicting successful weaning of RRT in ICU.

## P103 How to predict early hypophosphatemia in the ICU?

### C Lauwers, L Langouche, J Gunst, A Pesonen, MP Casaer

#### KU Leuven, Laboratory of Intensive Care Medicine, Leuven, Belgium

*Critical Care* 2024, **28(Suppl 1):** P103

**Introduction:** A caloric intake of > 50% of target may be harmful in critically ill patients developing absolute (AHP) and/or relative hypophosphatemia (RHP). Preadmission risk factors remain unclear, however, rendering it impossible to anticipate such hypophosphatemia. Therefore, we aimed to describe the incidence of AHP and RHP in the absence of early parenteral nutrition (PN) and identify potential risk factors.

**Methods:** Patients aged over 2 years admitted to the intensive care unit (ICU) for > 2d in the University Hospitals Leuven between September 2022 and May 2023 with available phosphate levels were retrospectively investigated (n = 412). Hypophosphatemia was defined as an absolute value < 0.65 mmol/L or a relative decrease of > 0.16 mmol/l on the second day in ICU. Baseline characteristics, and the caloric intake on the third day in ICU were recorded. Potential risk factors for developing AHP and RHP were assessed by multivariate logistic regression, separately in the adult and pediatric cohorts.

**Results:** AHP occurred in 17 patients (4.7%) and RHP in 122 patients (33.9%) in the adult cohort (n = 360). In the pediatric cohort (n = 52), AHP developed in only 2 patients (3.9%) and RHP in 22 patients (42.3%). Age, sex, body weight and body-mass index (BMI) were similarly distributed among patients with and without AHP and RHP in both the adult and pediatric cohorts. The subsequent caloric intake on day 3 was > 50% of target in 58 patients (14.1%) in the total cohort, in 3 patients with AHP (15.8%) and 19 patients with RHP (13.2%). Age, sex, body weight and BMI did not independently associate with the occurrence of AHP or RHP.

**Conclusions:** RHP occurred often after ICU admission, whereas AHP was rather uncommon. The potentially detrimental combination of AHP/RHP and a nutritional intake > 50% of target was rare in the absence of early PN. Preadmission characteristics could not predict the occurrence of AHP or RHP. Future studies should further investigate the significance of AHP and RHP in relation to outcome.

## P104 Prealbumin dynamic assessment for nutrition support effectiveness in critically ill patients

### E Pardo^1^, M Jabaudon^2^, T Godet^2^, B Pereira^3^, D Morand^4^, E Futier^2^, E Le Cam^5^, MP Bonnet^6^, JM Constantin^7^

#### ^1^Hôpital Saint-Antoine, Département Anesthésie-Réanimation, Paris, France, ^2^CHU de Clermont-Ferrand, Department of Perioperative Medicine, Clermont-Ferrand, France, ^3^CHU de Clermont-Ferrand, Biostatistics and Data Management Unit, Clermont-Ferrand, France, ^4^CHU de Clermont-Ferrand, Direction de la Recherche Clinique, Clermont-Ferrand, France, ^5^Hôpital Saint-Antoine, Département d’Anesthésie-Réanimation, Paris, France, ^6^Hôpital Armand Trousseau, Département Anesthésie-Réanimation, Paris, France, ^7^Hôpital Pitié-Salpêtrière, Département d’Anesthésie-Réanimation, Paris, France

*Critical Care* 2024, **28(Suppl 1):** P104.

**Introduction:** Prealbumin (PAB) has been suggested to be a sensitive predictor of clinical outcomes and a quality marker of nutrition support. Given its sensibility to inflammation, its use in critically ill patients is not supported by current guidelines. We assessed the performance of PAB initial value and dynamic changes for predicting short-term outcome and nutrition support effectiveness.

**Methods:** The study included patients admitted to the ICU (2009–2016), with at least one initial PAB value available. Data, prospectively recorded, were extracted from the electronic ICU charts. We estimated the performance of PAB to predict ICU mortality using univariable then multivariable logistic regressions. Association of PAB dynamic changes and nutrition support was assessed using multivariable linear mixed-effects model and multivariable linear regression. Subgroup analysis was performed to identify patients in whom PAB dynamic assessment may be particularly relevant.

**Results:** We included 3136 patients with a total of 4942 available PAB levels. Both initial PAB (aOR 0.04 CI 95% 0.01–0.23) and PAB first week change (aOR 0.02, CI 95% 0.00–0.19) were negatively and significantly associated with ICU mortality. During the whole ICU stay, PAB dynamic changes were associated with both cumulative energy (estimate: 33.2, SE: 0.001, *p* < 0.01) and protein delivery (1.39, SE 0.001, *p* < 0.01). During the first week of stay, PAB changes were independently associated with mean energy (6.03e−04, SE 2.32e−04, *p* < 0.01) and protein intakes (1.97e−02, SE 5.91e−03, *p* < 0.01). The association of PAB with energy intake was strongest among older, malnourished patients, with increased inflammation and disease severity. Finally, PAB changes was associated with a positive mean nitrogen balance at D7, only in patients with SOFA < 4 (*p* = 0.047).

**Conclusions:** PAB initial and first week change is an accurate predictor of ICU mortality. PAB dynamic assessment may be a reliable tool to estimate ICU nutrition support effectiveness, especially in at-risk patients.

## P105 Hypokalemia during treatment of diabetic ketoacidosis. A retrospective analysis

### AM Miller^1^, SD Darawshi^2^, MQ Qassum^3^, IH Hochberg^2^

#### ^1^Rambam Medical Center, Medical ICU, Haifa, Israel, ^2^Rambam Medical Center, Endocrinology, Haifa, Israel, ^3^Rambam Medical Center, Cardiology, Haifa, Israel

*Critical Care* 2024, **28(Suppl 1):** P105

**Introduction:** Patients with diabetic ketoacidosis (DKA) are often potassium depleted and prone to hypokalemia during treatment. The reasons to hypokalemia are mainly kaliuresis, inadequate oral intake, gastrointestinal losses and insulin supplementation during treatment. There is a concern that hypokalemia during treatment leads to hazardous events including arrhythmias and cardiac death, however the actual effect of hypokalemia during treatment has not been characterized. We assessed the outcomes of DKA hospitalizations according to whether hypokalemia presence.

**Methods:** We retrieved all hospitalizations of adult patients with moderate or severe DKA (defined by a combination of clinical diagnosis, pH under 7.3, glucose > 250 mg/dL and IV insulin treatment) in a tertiary medical center between 2/2012 and 1/2020 and compared demographic, anthropometric and hospitalization variables, blood tests, 30 day and total mortality between patients who did or did not develop hypokalemia.

**Results:** There were 456 DKA hospitalizations. 304 were complicated by hypokalemia (potassium < 3.5 meq/L within the 3 days before or after the time of the lowest pH). Compared to patients who did not develop hypokalemia, patients who did were younger (46.3 ± 19.3 vs. 52.3 ± 21.5, *p* = 0.003), had a lower pH (7.08 ± 0.14 vs. 7.15 ± 0.11 *p* < 0.001) and lower bicarbonate (8.99 ± 4.50 vs. 12.3 ± 4.11, *p* < 0.001) (Table). More patients who developed hypokalemia were admitted to ICU (36.8% vs. 13.8%, *p* < 0.01) and had higher maximal creatinine (2.18 ± 1.6 vs. 1.82 ± 1.36, *p* < 0.05). Hypokalemia during treatment was associated with a longer hospital stay. The 30 day mortality was similar between groups (6.3% in the hypokalemia and 7.9% in normokalemia, *p* = 0.55) as was long term mortality (26.6% and 33.6% respectively, *p* = 0.13).

**Conclusions:** Hypokalemia occurs commonly during treatment of moderate to severe DKA and is associated with lower pH and bicarbonate, acute renal failure and higher rate of ICU admissions. However, it is not associated with a higher short or long-term mortality.

**Table (abstract P105):** Characteristics of patients according to potassium levels

Characteristic	Min K ^+^ < 3.5; N = 304	Min K ^+^ ≥ 3.5; N = 152	*p* value
Male	129 (42.4%)	81 (53.3%)	*p* < 0.036
Female	175 (57.6%)	71 (46.7%)	
Minimal PH	7.08 ± 0.14	7.15 ± 0.11	*p* < 0.001
Maximal creatinine (mg/dL)	2.18 ± 1.6	1.82 ± 1.36	*p* = 0.011
ICU admission	112 ± 36.8	21 ± 13.8	*p* < 0.001
30 day mortality	12 (7.9%)	19 (6.3%)	*p* = 0.55
Mortality > 30 days	51 (33.6%)	81 (26.6%)	*p* = 0.13

## P106 The association between duration of hyperglycemia above different glucose levels and 90-day mortality in critically ill patients: a retrospective cohort study

### E Robinson^1^, L Statlender^2^, A Grossman^3^, H Duskin^4^, T Shochat^5^, M Hellerman Itzhaki^2^, G Fishman^2^, P Singer^2^, I Kagan^2^, I Bendavid^2^

#### ^1^Rabin Medical Center - Beilinson Hospital, General Intensive Care Unit, Petah Tikva, Israel, ^2^Rabin Medical Center - Beilinson Hospital, Department of General Intensive Care, Petah Tikva, Israel, ^3^Rabin Medical Center - Beilinson Hospital, Department of Medicine B, Petah Tikva, Israel, ^4^Rabin Medical Center - Beilinson Hospital, Institute of Endocrinology, Petah Tikva, Israel, ^5^Rabin Medical Center - Beilinson Hospital, Statistical Consulting Unit, Petah Tikva, Israel

*Critical Care* 2024, **28(Suppl 1):** P106

**Introduction:** Practice regarding glucose control has changed over time due to accumulating evidence [1, 2], however, the specific thresholds chosen for initiating insulin therapy are somewhat arbitrary and a different approach might be needed for patients with and without diabetes mellitus (DM) [3]. We attempted to shed light on these issues by examining the association between the time spent above different glucose levels and 90-day mortality.

**Methods:** A retrospective cohort from an adult ICU. Patients hospitalized for 48 h and up to 8 weeks were included. Patients admitted for DKA/HHS, OB/GYN, liver transplant, and suspected brain death were excluded. Separate univariate and then multivariate analyses were performed for each glucose level from 100 to 250 mg/dL in 10 mg/dL increments to test for the association between the time spent above that level and 90-day mortality. Due to interaction, the analysis was stratified by DM status.

**Results:** 1397 patients (21.67% with DM) with 72,030 glucose measurements were included in the final analysis. Patients with DM had higher mean glucose (163 ± 28.1 vs. 138 ± 26.5; *p* < 0.01), higher glucose variability (30 ± 10.6 vs. 22.3 ± 9.7; *p* < 0.01), and higher 90-day mortality (43.9% vs. 33.2%; *p* < 0.01). No association with 90-day mortality was found for time spent above all glucose levels in patients with DM, however, for patients without DM, time spent above all levels from 140 mg/dL and higher was associated with 90-day mortality (Figure). This was adjusted for known predictors of mortality.

**Conclusions:** The duration of hyperglycemia above 140 mg/dL was associated with 90-day mortality in patients without DM, even when adjusted for APACHE2. Prospective studies are needed to assess different glucose thresholds and explore novel approaches to glucose control including individualized protocols for patients with and without DM.

**References**

1. Van Den Berghe G et al. N Engl J Med. 2001;345:1359–67
2. Finfer S et al. N Engl J Med. 2009;360;1283–97
3. Wu Z et al. J Intensive Med. 2202;2:131–45

**Figure (abstract P106):**
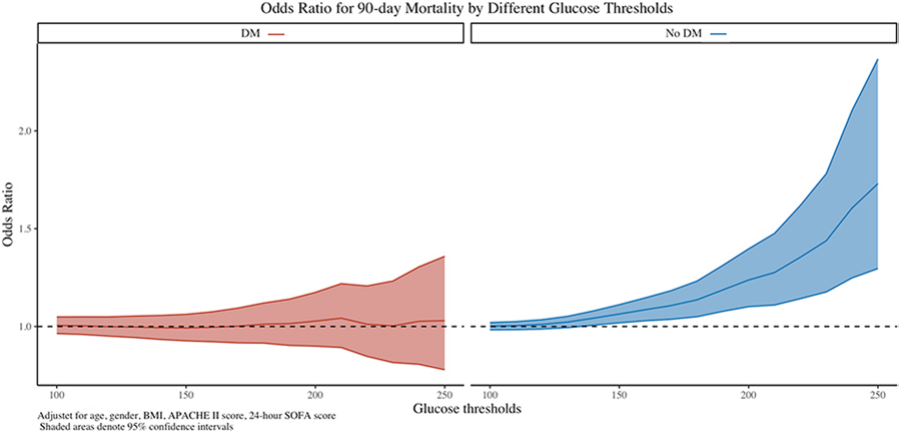
Odds ratios from multivariate analysis of time spent above glucose levels ranging from 100 to 250 mg/dL in 10 mg/dL increments. Left panel (red graph) shows regression results for patients with DM, right panel (blue graph) shows results for patients without DM.

## P107 Assessment of insulin requirements and glycemic control in adult patients on extracorporeal membrane oxygenation

### J Camacho

#### Hennepin County Medical Center, Pharmacy, Minneapolis, USA

*Critical Care* 2024, **28(Suppl 1):** P107

**Introduction:** Hyperglycemia is a known complication associated with poor patient outcomes in critically ill patients [1, 2]. Current evidence on glycemic control and insulin (ins) requirements for patients on extracorporeal membrane oxygenation (ECMO) is limited to smaller retrospective studies. Prior studies reported glycemic control had no effect on mortality for pediatric or adult patients on ECMO despite finding higher rates of hyperglycemia [3, 4]. Hyperglycemia has been associated with ECMO, but no further studies have evaluated ins requirements for patients on ECMO. Ins requirements and glycemic control were assessed in patients on ECMO at a level 1 trauma and academic medical center.

**Methods:** This single-center retrospective analysis was conducted in adult patients who received ECMO for ≥ 48 h between 1 June 2015 and 1 June 2023. The primary objective was the mean daily ins requirements while receiving ECMO support compared to mean daily ins requirements while not receiving ECMO support. Hypoglycemia was defined as blood glucose ≤ 70 mg/dL and hyperglycemia was defined as a blood glucose ≥ 180 mg/dL.

**Results:** Preliminary analysis included 107 patients. Most patients required veno-venous ECMO (62%) for a mean duration of 10.2 days. At baseline, 17% of patients had diabetes. The mean daily ins requirements were higher in patients while receiving ECMO (26.7 units/day) compared to when off ECMO (10.9 units/day). Hypoglycemia occurred in 8 patients while receiving ECMO and 14 patients off ECMO. Patients on ECMO had an increased rate of hyperglycemia compared to when off ECMO (46.5% vs. 19.6%).

**Conclusions:** Patients on ECMO had a higher rate of hyperglycemia, while receiving more ins compared to when off ECMO.

**References**

1. Finfer S et al. N Engl J Med. 2009;360:1283–97
2. Doenst T et al. J Thorac Cardiovasc Surg. 2005;130:1144
3. Lou S et al. Pediatr Crit Care Med. 2015;16:p 270–275
4. Lou S et al. J Extra Corpor Technol. 2010;42:281–5

## P108 Early macronutrient restriction is more commonly used in Belgian than in other European ICUs

### K Dams^1^, D Glorieux^2^, E Gilbert^3^, N Serck^4^, X Wittebole^5^, P Druwé^6^, M Simon^7^, E De Waele^8^, JC Preiser^9^

#### ^1^University Hospital Antwerp, Critical Care, Edegem, Belgium, ^2^Grand Hôpital de Charleroi, Intensive Care Unit, Charleroi, Belgium, ^3^Centre Hospitalier de Wallonie Picarde, Intensive Care Unit, Tournai, Belgium, ^4^Clinique Saint-Pierre, Intensive Care Unit, Ottignies, Belgium, ^5^Clinique Universitaire Saint-Luc, Department of Critical Care Medicine, Brussels, Belgium, ^6^Ghent University Hospital, Department of Intensive Care Medicine, Ghent, Belgium, ^7^Vivalia – Clinique Saint-Joseph, Intensive Care Unit, Arlon, Belgium, ^8^University Hospital Brussels, Department of Intensive Care Medicine, Brussels, Belgium, ^9^Hôpital Erasme, Intensive Care Unit, Brussels, Belgium

*Critical Care* 2024, **28(Suppl 1):** P108

**Introduction:** The EuroPN survey [1] was a prospective cohort study that assessed nutrition practice in European ICUs and its association with clinical outcomes. The aim of this post-hoc subgroup analysis was to compare the medical nutrition therapy (MNT) practices in Belgian ICUs to the overall EuroPN population and the 2019 ESPEN ICU guideline [2].

**Methods:** Clinical data of 149 patients from 9 Belgian ICUs were compared to 1,172 patients from 77 ICUs in 11 European countries. Macronutrient intake from enteral nutrition (EN), parenteral (PN) nutrition, and non-nutritional sources during 15 days after ICU admission were assessed and compared to the ESPEN targets of 20–25 kcal/kg/day and up to 1.3 g protein equivalents/kg/day, as well as to the results of the overall cohort.

**Results:** Both median ICU (12 [7;22] vs. 10 [7;16] d) and hospital (25 [15;37] vs. 23 [15;36] d) length of stay were longer in Belgian patients vs. the overall population and 31% versus 15% of patients originated from the emergency room. Median time to start MNT was longer compared to overall (EN: day 2.5 [2.0;4.0] vs. 2.0 [2.0;4.0]; PN: day 5.0 [3.0;7.0] vs. 2.0 [2.0;4.0]) and patients received EN more often. Provision of calories and proteins increased progressively over the first 5 days after ICU admission and, on average, were in the range of daily 10–20 kcal/kg and < 0.8 g protein/kg. Belgian patients met on average 71% of the ESPEN caloric and 53% of the protein targets over the study period, whereas overall this was 83% and 65%, respectively.

**Conclusions:** This Belgian subgroup analysis of the EuroPN study showed that average calorie and protein intakes during the first 15 ICU days were below the ESPEN targets (Figure). In line with the results of the overall cohort, it was common practice in Belgian ICUs to progressively increase MNT during the first days to moderate energy targets while a moderate protein intake of > 0.8 to < 1.2 g/kg/d as in the overall population was not achieved.

**References**

1. Matejovic M et al. Crit Care 2022;26,143
2. Singer P et al. Clin Nutr. 2019;38:48

**Figure (abstract P108):**
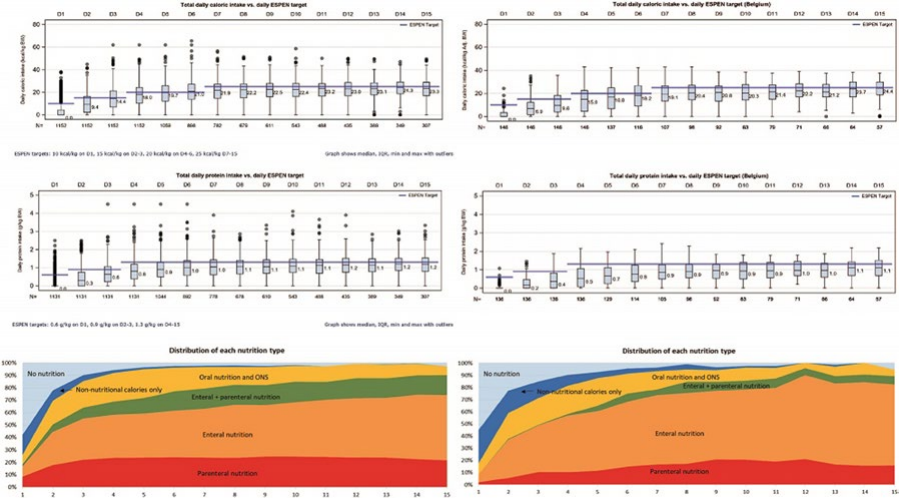
Daily caloric and protein intake and distribution of nutrition types (left: overall and right: Belgium). Intake is presented as median, IQR with outliers versus pre-defined targets (blue horizontal bars) based on the 2019 ESPEN Guideline on Clinical Nutrition in Critical Care, and with proportion of nutrition resources used on a respective day.

## P109 Gastrointestinal complications in critical care patients: data from a single tertiary hospital ICU in Greece

### M Papaioannou, D Daiou, E Eleftheriadou, S Bagdasarian, M Alevizaki, C Christou, A Lavrentieva

#### General Hospital of Thessaloniki "G. Papanikolaou", A ICU, Thessaloniki, Greece

*Critical Care* 2024, **28(Suppl 1):** P109

**Introduction:** The goal of this study was to evaluate the prevalence of gastrointestinal complications estimated with Gastrointestinal Dysfunction Score (GIDS) and its association with disease severity and prognosis in critical care patients.

**Methods:** This was an observational, prospective study including adult patients in a single Intensive Care Unit (ICU). Patients’ demographic characteristics, severity of illness, comorbidities, opioid prescription, administration of antibiotics, vasopressor requirements and gastrointestinal symptoms were recorded.

**Results:** Data of 64 patients were analyzed (median age 58 years (range 19–84), median APACHE II score 20 (range 5–39) and median SOFA score 11 (range 5–15) at admission. Median duration of mechanical ventilation was 38 days and median ICU length of stay (LOS) was 45 days. Patients were categorized in four groups regarding GIDS. Duration of mechanical ventilation and ICU LOS were significantly longer in patients with GIDS 3 and GIDS 4 (*p* < 0.05). The duration of opioid treatment, the total number of antibiotic drugs delivered, the duration of drug prescription and the duration of vasopressor use were associated with greater severity in GIDS. Diabetes mellitus was associated with more severe GIDS (2, 3 and 4). Patients who exhibited enteral feeding intolerance were more likely to have severe gastrointestinal dysfunction (x^2^ = 9.374, *p* < 0.025). Sepsis was related to higher GIDS (x^2^ = 9.663, *p* < 0.022). Patients with upper digestive bleeding had a higher mortality rate (x^2^ = 11.852, *p* < 0.01). Patients who underwent surgery (x^2^ = 37.966, *p* < 0.00) or upper gastrointestinal *endoscopy* (x^2^ = 15.129, *p* < 0.02) had higher GIDS. Higher GIDS grades were linked with higher mortality (Fisher’s exact test = 12.862, *p* = 0.002).

**Conclusions:** GIDS seems to be a useful severity score for ICU patients who exhibit gastrointestinal complications. More severe forms of gastrointestinal dysfunction estimated with GIDS are associated with higher morbidity and mortality rates among critical care patients.

## P110 Untargeted metabolomics analysis of acute-phase energy metabolism in critically ill patients

### A Yamamoto^1^, T Oshima^2^, T Oami^1^, S Ishida^1^, A Eguchi^3^, K Sakurai^3^, TA Nakada^1^

#### ^1^Chiba University, Department of Emergency and Critical Care Medicine, Graduate School of Medicine, Chiba, Japan, ^2^Chiba University, Institute for Advanced Academic Research, Chiba, Japan, ^3^Chiba University, Center for Preventive Medical Sciences, Chiba, Japan

*Critical Care* 2024, **28(Suppl 1):** P110

**Introduction:** Critically ill patients metabolize endogenous energy supply during the acute phase of their illness, gradually replaced by exogenous energy sources such as nutrition. International nutrition guidelines recommend the gradual progression of nutrition to adapt to the acute phase metabolic evolution, but the timing and mechanism of the metabolic change remain unclear. This study aimed to elucidate the acute-phase metabolic change in critically ill patients by untargeted metabolomics to guide optimal nutrition therapy.

**Methods:** A single-center prospective case series study was conducted on critically ill adults expected to require mechanical ventilation for ≥ 7 days in our ICU; data collection was started within 48 h of ICU admission. Daily serum samples from days 1 to 7 were analyzed by untargeted metabolomics using mass spectrometry. MetaboAnalyst 5.0® was employed for multivariate analysis. Principal component analysis (PCA) and partial least squares discriminant analysis (PLS-DA) were performed to evaluate time-series changes in the metabolomic profile. We also compared the sepsis and non-sepsis patient groups to evaluate the difference according to pathophysiology.

**Results:** Ten patients were included during the study period from July 2021 to September 2022; 8 were male, with a median age of 74.5 years. A total of 123 metabolites were annotated by untargeted metabolomics. PCA failed to demonstrate common time-series changes in metabolite patterns across the patients. PLS-DA revealed time-series changes in galactonic acid, ornithine, and L-arginine levels. Sepsis and non-sepsis groups were classified with significant contributions from creatine phosphate, uric acid, and creatinine.

**Conclusions:** Untargeted metabolomics revealed unique metabolite patterns contributing to time-series and pathophysiological metabolic changes in acute-phase critically ill patients. Further analysis to determine the optimal timing to start nutrition is warranted.

## P111 Impact of nutritional therapy during intensive care unit admission on post intensive care syndrome and post-COVID-19 condition in patients with COVID-19: a multicenter prospective study

### S Suganuma, K Kawabata, N Yokoyama, M Idei, S Kashiwagi, M Yokose, S Takaki, K Nakamura

#### Yokohama City University Hospital, Intensive Care, Yokohama-city, Japan

*Critical Care* 2024, **28(Suppl 1):** P111

**Introduction:** Nutritional therapy is an important component of intensive care. We examined the impact of acute nutritional therapy on long-term outcomes of Post Intensive Care Syndrome (PICS) and post COVID-19 condition in severely ill COVID-19.

**Methods:** This work was supported by MHLW Research on Emerging and Re-emerging Infectious Diseases and Immunization (Program Grant Number JPMH21HA2011). Total energy per body weight (kcal/kg/week) and protein intake (g/kg/week) were calculated during the first week after admission to the intensive care unit (ICU). The primary outcome was a decrease in quality of life (QOL) defined by EuroQol-5dimensions-5level < 0.8 at 1 year after the date of COVID-19 diagnosis. We made secondary outcomes physical impairment, mental impairment, Malnutrition Universal Screening Tool (MUST) and post COVID-19 condition at 1 year after the date of COVID-19 diagnosis. Logistic regression analysis examined the association between energy and protein intake and PICS, MUST and post COVID-19 condition.

**Results:** The 220 patients were included in the study. The median total energy and protein intake per body weight during the first 7 days after admission to the ICU was 65.12 kcal/kg/week and 3.30 g/kg/week. Logistic regression analysis adjusted by age, gender, body mass index, Sequential Organ Failure Assessment score, duration of mechanical ventilation, noninvasive ventilatory therapy, and extracorporeal circulation showed that total energy and protein intake during the first 7 days after ICU admission reduced QOL decline (energy: odds ratio 0.98 [0.97–0.99], *p* = 0.008; protein: odds ratio 0.75 [0.59–0.95], *p* = 0.018). The incidence of malaise and muscle weakness as the post COVID-19 condition and the incidence of middle risk or higher in MUST was lower with higher energy and protein administration.

**Conclusions:** Acute nutritional therapy after admission to the ICU was associated with better QOL after 1 year after COVID-19 infection and may prevent some of the post COVID-19 conditions.

## P112 The difference of resting energy expenditure by using predictive formulas and indirect calorimetry in Asia burn patients

### HC Hu^1^, LT Chou^2^, HY Cho^2^, SW Lin^1^, KC Kao^1^

#### ^1^Chang Gung Memorial Hospital, Department of Thoracic Medicine, Taoyuan, Taiwan, Republic of China, ^2^Chang Gung Memorial Hospital, Department of Respiratory Therapy, Taoyuan, Taiwan, Republic of China

*Critical Care* 2024, **28(Suppl 1):** P112

**Introduction:** The nutrition support of severe burn patients are crucial. Overfeeding or underfeeding will cause different complication[1]. There is several predictive formulas are developed for patients nutritional supplement[2–4]. The gold standard for assessing energy expenditure (EE) is indirect calorimetry (IC). The aim of this research is to investigate the difference of resting EE (REE) in Asian patients measured by IC and several current used formulas.

**Methods:** This is a prospective study. Adults admitted to burn ICU due to burn injury with greater than 25% of body surface area and respiratory failure were enrolled. All enrolled patients received measurements on day-1, 3, 5 after admitting to ICU. Calorimeter module on the ventilator was used for IC measurement. Basal metabolic rate (BMR) was calculated by the Harris–Benedict equation. Predictive equations were used for comparison [5–9].

**Results:** There were statistically significant differences between measured EE and predicted REE and between measured EE and real caloric intake. Bland–Altman analysis showed a mean bias of 550.5 kcal/day. The 95% CI for the lower and upper limits of agreement ranged from − 1731.7 to 709.7 kcal/d and from − 391.3 to 630.8 kcal/d, respectively.

**Conclusions:** Our results suggest that measured REE by IC was significantly higher than calculated REE in Asian burn patients need mechanical ventilation. Indirect calorimetry is advised to modify the patient’s nutrition strategy.

**References**

1. Clark A et al. Burns Trauma. 2017;5:11
2. Ireton-Jones CS et al. Nutr Clin Pract. 2002;17:29–31
3. Ireton-Jones CS et al. J Burn Care Rehabil. 1992;13:330–333
4. Mendonca Machado N et al. Nutr Hosp. 2011;26:692–700
5. Harris JA et al. Proc Natl Acad Sci USA.1918; 4:370–3
6. World Health Organ Tech Rep Ser. 1985;724:1–206
7. Mifflin MD et al. Am J Clin Nutr. 1990;51:241–247
8. Owen OE et al. Am J Clin Nutr. 1986;44:1–19
9. Owen OE et al. Am J Clin Nutr. 1987;46:875–885

## P113 The effect of hypocaloric-high protein feeding critically ill adult patients with obesity: a systematic review and meta-analysis

### A Alsuwaylihi^1^, P Skorepa^2^, Y Alhindi^3^, A Avery^4^, J Musson^4^

#### ^1^University of Nottingham, Nottingham Digestive Diseases Centre, Division of Translational Medical Sciences, School of Medicine, University of Nottingham, Queen’s Medical Centre, Nottingham, UK, Nottingham, UK, ^2^Faculty of Military Health Sciences, University of Defence, Trebesska 1575, Department of Military Internal Medicine and Military Hygiene, Hradec Kralove, 500 02, Czech Republic, ^3^University of Nottingham, Clinical, Metabolic and Molecular Physiology, MRC-Versus Arthritis Centre for Musculoskeletal Ageing Research, Nottingham, UK, ^4^University of Nottingham, School of Biosciences, Division of Food, Nutrition & Dietetics, Nottingham, UK

*Critical Care* 2024, **28(Suppl 1):** P113

**Introduction:** Use of a hypocaloric high protein feeding approach for critically ill obese patients has been recommended by a number of organisations and institutes with the goal being to avoid overfeeding and its complications that may worsen patients’ overall status. The objective of this review is to assess the efficacy of hypocaloric-high protein feeding protocol on mortality and ventilator-associated pneumonia (VAP) incidences among critically ill obese patients.

**Methods:** The Preferred Reporting Items for Systematic Reviews and Meta-Analyses statement (PRISMA) methodology was used to search PubMed, EMBASE and MIDLINE databases. All papers published up to up to July 2021 with mortality and VAP incidence as primary outcomes were retrieved. The secondary outcomes were average daily caloric intake and length of intensive care unit (ICU) stay.

**Results:** No significant reduction in the mortality incidence when the hypocaloric-high protein feeding approach was used (RR 0.74, CI 0.35–1.56, *p* = 0.43), similarly, no significant reduction in VAP incidence were observed when the hypocaloric-high protein feeding approach was used (RR 0.84, CI 0.50–1.41, *p* = 0.52). Additionally, no significant reduction in the mean ICU days when the hypocaloric-high protein feeding approach was used (MD − 0.82 days, CI − 5.90 to 4.27, *p* = 0.75), but there was a significant reduction in mean daily caloric intake in the hypocaloric-high protein feeding approach groups (MD − 4.80 kcal/kg/day, CI − 6.99 to − 2.62, *p* < 0.0001).

**Conclusions:** This review did not indicate superiority of hypocaloric high protein feeding approach to eucaloric or standard feeding approach in regards of mortality, VAP incidence and length of ICU stay. There was a significant reduction in daily caloric between hypocaloric high protein feeding compared with eucaloric or standard feeding approach, with no benefits to the outcomes.

## P114 An automated electronic medical tool to recall nutritional intake and present feeding adequacy in the critically ill: it all starts with an excel file

### L Buyle^1^, L de Hart^2^, Z Rosseel^3^, BG Jimenez Garcia^4^, L Leemans^5^, E De Waele^4^

#### ^1^UZ Brussel, Department Clinical Nutrition, Jette, Belgium, ^2^UZ Brussel, Universitair Ziekenhuis Brussel (UZ Brussel), Department Clinical Nutrition, Jette, Belgium, ^3^UZ Brussel, Universitair Ziekenhuis Brussel (UZ Brussel), Department of Pharmacy, Jette, Belgium, ^4^UZ Brussel, Vrije Universiteit Brussel (VUB), Universitair Ziekenhuis Brussel (UZ Brussel), Department Clinical Nutrition, Research Cluster Development - Ageing and Pathology, Jette, Belgium, ^5^UZ Brussel, Vrije Universiteit Brussel (VUB), Rehabilitation Research (RERE), Jette, Belgium

*Critical Care* 2024, **28(Suppl 1):** P114

**Introduction:** Feeding adequacy in the intensive care unit (ICU) remains an issue. Both under- and overfeeding are present and are harmful to ICU patients [1]. Daily calculations can help optimize clinical practice by giving a quick and clear overview of intentional and non-intentional nutritional intake. A structured, systematic approach is often lacking. In this study, the development and implementation of an electronic feeding adequacy tool was investigated.

**Methods:** Intensivists and medical nutrition experts designed the content of the tool, facilitated the implementation plan and introduced a quality control system. The information technologists (IT) integrated the tool in the hospitals’ medical system. This tool allowed ICU healthcare practitioners to chart feeding adequacy daily.

**Results:** Automated 24 h-recall of energy and protein content, electrolytes, vitamins and trace elements of artificial nutrition (i.e. parenteral and enteral nutrition) and non-intentional calories (e.g., propofol, glucose) was launched. In 2021, the tool was used 5278 times for 8832 possible treatment days (60%). In 2022, the tool was used 4440 times and in 2023, the tool was already used for 4244 times.

**Conclusions:** Designing, implementation and use by cooperation between medical doctors, dieticians and IT of an automated electronic medical tool proved to be feasible in clinical practice.

**Reference**

1. Zusman O et al. Crit Care 2016;20:367

**Figure (abstract P114):**
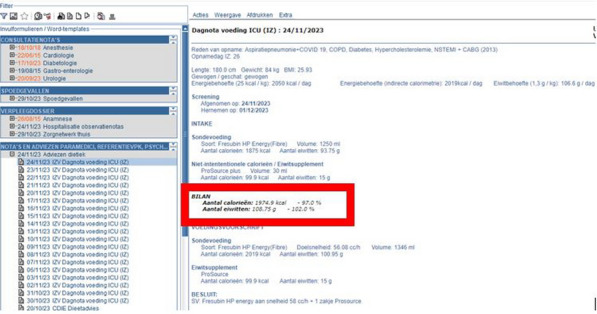
Example of the automated electronic medical where nutritional adequacy was calculated and nutritional prescription is noted by the ICU dietician.

## P115 Nutritional adequacy in intensive care patients using daily checklist instrument: a retrospective analysis

### RI Prado^1^, MT Tanita^1^, AS Larangeira^1^, LS Hirata^2^, LTQ Cardoso^3^, CMC Grion^3^

#### ^1^University Hospital – Londrina State University, Critical Care, Londrina, Brazil, ^2^University Hospital – Londrina State University, Nutrition Department, Londrina, Brazil, ^3^University Hospital – Londrina State University, Department of Internal medicine, Londrina State University, Londrina, Brazil

*Critical Care* 2024, **28(Suppl 1):** P115

**Introduction:** Malnutrition leads to worsening clinical outcomes in critically ill patients and underfeed is a prevalent problem in intensive care units (ICU). The aim of this study was to analyze data collected from a checklist instrument to quantify nutritional adequacy of patients in an intensive care bed in a public service and the reasons for interruptions in nutrition support.

**Methods:** Retrospective analysis performed on a database collected prospectively, during the daily multidisciplinary round, in eight ICUs at a University Hospital, which included surgical and nonsurgical patients, from March 2021 to April 2023. In total 46,063 records were analyzed regarding the caloric adequacy of nutritional support and the reasons for nonadherence to 80% of the defined daily caloric target.

**Results:** The adequacy to the target of 80% of the daily caloric value remained stable, with an average of 67.3%. The reason for nonadherence to the daily nutritional support target was a significant increase in fasting due to preoperative and diagnostic procedures. A reduction in the delay to start nutritional support was detected from the year 2022 and remained stable at the beginning of 2023. Other reasons for nonadequacy remained stable during the evaluated period. The average annual rates of nonadequacy to nutritional support in comparison between the years 2021 and 2022 and 2021 and 2023 were statistically significant (*p* < 0.001 in both analyses). While the comparison between 2022 and 2023 was marginally significant (*p* = 0.04).

**Conclusions:** It was verified an increase in preoperative fasting and for diagnostic procedures from the year 2022, which coincides with the return of elective surgeries, suspended due to the COVID-19 pandemic. From this same period, was observed a reduction in delay in starting nutritional support. Our hypothesis is greater awareness and adherence to the calculation of the daily caloric target and prescription of more agile nutritional support by the entire multidisciplinary team.

## P116 Optimal choice of parenteral nutrition in ICU patients treated with continuous venovenous hemofiltration

### J Jonckheer^1^, W Pieteraerens^2^, L de Hart^3^, D Goethals^4^

#### ^1^Universitair Ziekenhuis Brussel, Intensive Care, Jette, Belgium, ^2^Universitair Ziekenhuis Brussel, Department of Nursing and Midwifery Research Group (NUMID), Jette, Belgium, ^3^Universitair Ziekenhuis Brussel, Department of Clinical Nutrition and Dietetics, Jette, Belgium, ^4^Universitair Ziekenhuis Brussel, Department of Internal Medicine, Jette, Belgium

*Critical Care* 2024, **28(Suppl 1):** P116

**Introduction:** The energy and protein target can be expressed as E/P(energy to protein ratio) and can be used to guide the choice of medical nutrition. ESPEN guidelines still advocate energy prescription based on indirect calorimetry (IC) adapted for the non-nutritional calories [1]. Continuous venovenous hemofiltration (CVVH), which is a type of continuous renal replacement therapy will alter energy and protein targets due to exchange of nutrients [2,3]. We aimed to evaluate how the E/P needs changes due to CVVH and how this could impact the choice of available parenteral medical nutrition.

**Methods:** A retrospective analysis was performed on adult ICU patients admitted between 01/01 and 31/12/2022 treated with CVVH who had at least one IC measurement. We calculated the energy target based on REE (resting energy expenditure) measured with IC from which the bioenergetic balance of the CVVH was deducted. The E/P of each subject was calculated.

**Results:** A total of 45 IC measurements were included. When citrate was used, E/P ratio was 10.28 ± 2.44 kcal/g compared to 15.95 ± 3.15 kcal/g when citrate was not used which was statistically significant different (*p* < 0.01). During citrate predilution Olimel N12 concurred in 20.7% of cases as compared to 10.3% with SMOFKabiven Extra Amino and 0% with Olimel N9 and SMOFKabiven.

**Conclusions:** Patients treated with CVVH have a specific E/P ratio which can be further stratified based on citrate use. Olimel N12 is the most appropriate parenteral nutrition in CVVH patients treated with citrate. Further pharmaceutical developments are necessary to develop ready to use parenteral nutrition to individualize the nutritional therapy in CVVH patients.

**References**

1. Singer P et al. Clin Nutr. 2023;42:1671–89
2. Fiaccadori E et al. Clin Nutr. 2021;40:1644–68
3. Jonckheer J et al. Nutrients 2022;14:2112

## P117 A rare and deadly disease with a paradigm shift

### C Oliveira Paulo^1^, C Gonçalves^2^, F Almeida^3^, J Lopes^4^, V Pereira^1^, R Gomes^1^, A Fernandes^1^

#### ^1^Hospital Garcia de Orta, Intensive Care Unit, Almada, Portugal, ^2^Hospital Prof. Doutor Fernando Fonseca, Intensive Care Unit, Lisboa, Portugal, ^3^Unidade Local de Saúde do Baixo Alentejo, Intensive Care Unit, Beja, Portugal, ^4^Unidade Local de Saúde do Litoral Alentejano, Intensive Care Unit, Santiago do Cacém, Portugal

*Critical Care* 2024, **28(Suppl 1):** P117

**Introduction:** Esophageal perforation is a rare and potentially life-threatening emergency, associated with a high morbidity and mortality. Given multiple aetiologies and presentations, the diagnosis is commonly delayed, impacting negatively the outcome. There has been a paradigm shift in management from an aggressive surgical strategy to minimal invasive endoscopic therapies, including stenting. We present 7 case reports as a reminder to assist physicians in a prompt diagnosing and managing of this deadly disease.

**Methods:** We reviewed 7 esophageal perforation clinical cases that were admitted in our intensive care unit (ICU) during 2023. We reviewed the patient’s clinical files, blood tests, radiology results and surgical protocols.

**Results:** The cases occurred amongst 7 male adults: 2 iatrogenic (age 19), 2 cases after food impaction (age 31–65, 1 Mallory-Weiss Syndrome) and the remaining with Boerhaave Syndrome (BS) (age 45–77). Clinical presentation included pneumomediastinum (85.7%), pneumothorax (28.6%), mediastinitis (28.6%), pneumoperitoneum (14.3%) and cervical abscess (14.3%). Time to diagnosis was on average 2.41 days (6 h–8 days), and admission in the ICU with an average APACHE 17% and SAPS II 24%. Mechanical ventilation was needed in 57% and vasopressor support in 43%. Endoscopic stenting was the treatment strategy in 3 patients (1 needing subsequent surgery), 1 needed emergency surgery and the remaining 3 resolved spontaneously. Surgical control of mediastinitis was paramount in 2 cases. Five patients initiated parenteral nutrition, with an average of 7 days of therapy, enteral nutrition initiated on average 5.3 days after diagnosis. There was no associated mortality, although 3 patients remain hospitalized with clinical stability.

**Conclusions:** These reports emphasize the importance of early diagnosis, namely in BS. A multidisciplinary approach with prompt use of esophageal stenting and thoracostomy when presenting with mediastinitis, suggests the improvement of prognosis in a long feared disease.

## P118 The accuracy of new non-invasive intra-abdominal pressure measurement by physical examination and ultrasonography to diagnose intra-abdominal hypertension

### C Achavanuntakul, P Sirilaksanamanon, T Thawitsri, P Bootjeamjai

#### King Chulalongkorn Memorial Hospital, Department of Anesthesiology, Bangkok, Thailand

*Critical Care* 2024, **28(Suppl 1):** P118

**Introduction:** Intra-abdominal hypertension (IAH) is a serious condition that can lead to life-threatening complications if left untreated. Early diagnosis and treatment are the mainstay to decrease morbidity and mortality. The gold standard for non-invasive intra-abdominal pressure monitoring is intravesical pressure measurement, which has several limitations. For this reason, this study is conducted to develop a new non-invasive method to diagnose IAH, especially for unreliable or unable to measure intravesical pressure.

**Methods:** This cross-sectional diagnostic study was conducted in the surgical intensive care unit. The gold standard intravesical pressure measurement was measured, and intraabdominal pressure (IAP) was recorded. Simultaneously, a new non-invasive method by physical examination combined with ultrasound was assessed in all patients. The ratio of maximal anteroposterior to transverse abdominal diameter (AP-T) was also obtained. The relationship between IAP and the ratio of maximal AP-T was evaluated by Pearson’s correlation coefficient. The ability of a new non-invasive method to detect high IAP was assessed by receiver operating characteristic curve (ROC) analysis with the area under the ROC curve (AUROC) and by computing sensitivity and specificity.

**Results:** Of the 67 patients, 18 patients were diagnosed with IAH defined by IAP ≥ 12 mmH_2_O, and 49 patients had normal abdominal pressure. The relationship between the ratio of maximal AP-T was correlated with IAP (R^2^ 0.704, *p* < 0.001). The ratio of maximal AP-T diameter exhibited good discrimination for abdominal hypertension with AUROC 0.84, 95% CI 0.73–0.94. A ratio of maximal AP-T diameter ≥ 0.56 could detect high IAP with a sensitivity of 83.33%, a specificity of 83.67%, a positive predictive value of 65.2%, and a negative predictive value of 93.2%.

**Conclusions:** In this study, a new non-invasive method may be considered an alternative to intravesical pressure measurement for detecting IAH.

## P119 Demographic variables as predictors of clinical outcomes in patients undergoing liver transplantation

### G Madrid^1^, AF Zuluaga^1^, A Montoya^2^, A Obando^1^, A Delgadillo^1^, A Pinilla^1^, F Raffan^1^, JF Parada-Márquez^1^

#### ^1^Hospital Universitario Fundación Santa Fe de Bogotá, Anesthesiology, Bogotá, Colombia, ^2^Universidad de Los Andes, Faculty of Medicine Universidad de Los Andes, Bogotá, Colombia

*Critical Care* 2024, **28(Suppl 1):** P119

**Introduction:** We conducted a study to determine sociodemographic factors associated with postoperative ICU requirement in liver transplant patients. Liver transplantation (LT) is the treatment of choice for multiple liver diseases. It is estimated that up to 40% of transplant patients require intensive care unit (ICU) [1]. Studies have suggested that demographic factors are associated with poor clinical outcomes.

**Methods:** A historical cohort of patients who underwent LT at Fundación Santa Fe University Hospital in Bogotá from the year 2021–2023 was analyzed. Sociodemographic variables and their association with the postoperative ICU requirement were analyzed. Descriptive statistics and bivariate analyses were performed. To explore the associations between qualitative variables, the chi2 or Fisher test was used, and for the quantitative variables, T-Student or Man Whitney, depending on normality. A two-tailed *p* < 0.05 was established as the level of statistical significance.

**Results:** 78 patients were included in the analysis, of which 41 (52.56%) were men and 53.9% were over 60 years of age. 52 (66.67%) required ICU admission and 26 (33.33%) required a general ward. Demographic variables such as age, sex, BMI, and number of comorbidities were analyzed. A statistically significant association was found between the number of comorbidities (OR 3.13; 95% CI 1.16–8.46 *p* = 0.024) (Table). Of the total population, 62.3% had five or more comorbidities and 37.7% had four or fewer comorbidities. ICU requirement was more frequent in patients with five or more comorbidities than in those who had four or fewer comorbidities (71.15% vs. 28.85%). No statistically significant associations were observed for the other variables studied.

**Conclusions:** The results of our research suggest that the need for intensive care is related to the number of comorbidities but not to other demographic variables.

**Reference**

1. Taner CB et al. Liver Transpl. 2012;18:361–9.

**Table (abstract P119):** Demographic variables associated with ICU requirement

Variable (n = 78)	ICU (n = 52) (66.67%)	Hospital ward (n = 26) (33.33%)	OR (95% CI)	*p* value
Sex (female)	26 (50)	11 (42.30)	1.36 (0.52–3.52)	0.522
Age (≥ 60 years)	29 (55.77)	13(50)	1.26 (0.49–3.23)	0.630
Weight (Kg)*	68.98 (15.22)	65.51 (11.59)	1.01 (0.98–1.05)	0.307
BMI				
Overweight	21 (40.38)	10 (38.46)	2.1 (0.11–37.12)	0.613
Obese	11 (21.15)	2 (7.69)	5.5 (0.23–128.96)	0.290
Comorbidities ≥ 5	37(71.15)	11 (42.30)	3.13 (1.16–8.46)	0.024

*mean (SD).

## P120 Metabolic factors associated with ICU requirement in patients with liver transplant

### G Madrid^1^, JF Parada-Márquez^1^, AF Zuluaga^1^, E Arango^1^, O Amaya^1^, J Barrios^1^, A Montoya^2^, M Gonzalez^1^, J Cortes^1^

#### ^1^Hospital Universitario Fundación Santa Fe de Bogotá, Anesthesiology, Bogotá, Colombia, ^2^Universidad de Los Andes, Faculty of Medicine Universidad de Los Andes, Bogotá, Colombia

*Critical Care* 2024, **28(Suppl 1):** P120

**Introduction:** We conducted a study to determine the intraoperative metabolic factors associated with postoperative ICU requirement in liver transplant patients. Liver transplantation (LT) is a treatment option for multiple liver diseases. Different biomarkers have been associated with greater ICU requirements [1] and worse postoperative outcomes; however, the impact of metabolic factors on liver transplantation has not been clearly established.

**Methods:** Arterial blood gases were analyzed prior to discharge from the surgery rooms of 78 patients who underwent LT at Fundación Santa Fe University Hospital in Bogotá from 2021 to 2023. The main outcome was the postoperative ICU requirement. Metabolic variables (pH, bicarbonate, base deficit, electrolytes, and CO_2_) were analyzed and adjusted for confounding variables using a multivariate analysis. Descriptive statistics and bivariate analysis were performed. To explore the associations between qualitative variables, the chi2 test was used, and for the quantitative variables, Student’s t-test or Mann Whitney U test was used, depending on normality.

**Results:** A total of 78 patients were analyzed. 52.56% (41) were men, and 53.9% were over 60 years old. 52 (66.67%) required ICU admission (Table). A pH of less than 7.35 was associated with a greater ICU requirement (OR 10.9; 95% CI 2.28–52.14 *p* = 0.003), after adjusting for confounding factors (sex, BMI, age, and number of comorbidities). Of the patients with acidosis, 92.31% required ICU. Additionally, a greater base deficit was found in patients with ICU requirements − 3.9 (IQR − 6.9 to − 1.7) versus − 1.1 (IQR − 3.9 to 0.1) (*p* = 0.0310) and lower bicarbonate levels 20.7 (SD 3.0) versus 22.28 (SD 2.9) *p* = 0.0059.

**Conclusions:** The analysis of metabolic variables in patients who underwent LT suggests that arterial blood gas analysis is a useful tool to determine a patient's in-hospital fate, as factors such as pH, base deficit, and bicarbonate are associated with a greater ICU requirement.

**Reference**

1. Moore HB et al. Am J Surg 2023;226:829–834

**Table (abstract P120):** Univariate analysis of biomarkers used

Variable	ICU (n = 52)	Hospital ward (n = 26)	*p* value
Perioperative pH	7.36 (7.33–7.4)	7.42 (7.38–7.44)	0.0005
Perioperative base deficit	− 3.9 (− 6.9, − 1.7)	− 1.1 (− 3.9, 0.1)	0.03
Perioperative sodium*	138.45 (3.7)	136.85 (3.2)	0.07
Perioperative potassium	4.1 (3.6–4.7)	3.85 (3.4–4.3)	0.18
Perioperative bicarbonate*	20 (3)	22 (2.9)	0.005
Perioperative CO_2_	36 (34–38)	35.5 (34–38)	0.78
Preoperative albumin	3.2 (2.83,3.7)	3.3 (2.8–3.6)	0.883

Values expressed as medians (IQR). *mean (SD).

## P121 Perioperative fluids and blood products: determinants of intensive care unit admission in liver transplant patients

### G Madrid^1^, JF Parada-Márquez^1^, AF Zuluaga^1^, MC Escobar^1^, AC Gomez^1^, J Navarro^1^, A Montoya^2^, J Cortes^1^, C Ceballos^1^

#### ^1^Hospital Universitario Fundación Santa Fe de Bogotá, Anesthesiology, Bogotá, Colombia, ^2^Universidad de Los Andes, Faculty of Medicine Universidad de Los Andes, Bogotá, Colombia

*Critical Care* 2024, **28(Suppl 1):** P121

**Introduction:** We conducted a study to determine the association between intravenous (IV) fluids and transfusion with postoperative ICU requirement in LT patients. liver transplantation (LT) is the treatment of choice for various liver diseases. Intraoperative bleeding and the requirement for transfusion have been correlated with a higher risk of morbidity and mortality [1] as well as unfavorable clinical outcomes in transplant patients. We conducted a study to determine the association between intravenous (IV) fluids and transfusion with postoperative ICU requirement in LT patients.

**Methods:** A historical cohort of patients who underwent LT was analyzed at Fundación Santa Fe University Hospital in Bogotá between the period 2021–2023. The requirement for transfusion and IV fluids, and the need for postoperative ICU were analyzed. Descriptive statistics were performed and the chi2 or Fisher’s test was used to explore associations.

**Results:** 78 patients were included in this analysis. 41 (52.56%) were men. The majority (53.9%) were over 60 years old. Of the total patients, 52 (66.67%) required an ICU. 100% of the population was transfused. In 50% (26) of the patients who required ICU, all blood products were transfused; 9 (17.31%) red blood cells, plasma and platelets; 8 (15.38%) red blood cells and plasma; 6 (11, 54%) plasma and platelets; 1 (1.92%) red blood cells plasma and cryoprecipitates; 1 (1.92%) received only plasma and 1 (1.92%) plasma, cryoprecipitates and platelets. A statistically significant association was found between the transfusion of blood products and ICU requirement (*p* = 0.002). Interestingly, patients who did not require ICU were administered a greater amount of crystalloids (M:2300 IQR:1600–3100 vs. M:2000 IQR: 1165–3500; *p* = 0.5109).

**Conclusions:** Intraoperative IV fluid administration did not have a significant impact on postoperative ICU requirement. In contrast, transfusion of blood products is associated with greater postoperative ICU requirement.

**Reference**

1. Justo I et al. Transplant Proc. 2021;53:2298–304.

## P122 Transfusion of blood derivatives and their impact on the length of stay in patients undergoing hepatectomy

### G Madrid^1^, AF Zuluaga^1^, JF Parada^1^, A Montoya^2^, E Arango^1^, L Ferrer^1^, C Guerra^1^, D Diaz^1^

#### ^1^Hospital Universitario Fundación Santa Fe de Bogotá, Anesthesiology, Bogotá, Colombia, ^2^Universidad de los Andes, Universidad de los Andes, Bogotá, Colombia

*Critical Care* 2024, **28(Suppl 1):** P122

**Introduction:** The use of perioperative blood products (PBP) has been shown to have an impact on the morbidity of patients undergoing surgical procedures. In some cases, patients undergoing hepatectomy (HEP) are exposed to blood derivative transfusion (BDT) [1]; however, there is a gap in the literature in relation to the effect of BDT use on length of stay in the intensive care unit (LSICU) or general ward (LSGW).

**Methods:** An historical cohort type analytical observational study was conducted between January 2021 and August 2023. Patients older than 18 years who underwent HEP with a complete medical record were included. Our primary outcome was LSICU and secondary outcome was LSGW, and Sociodemographic variables (SDV) were analyzed. Descriptive statistics were calculated using mean differences, medians, and percentages, Additionally, analytical statistics were used with different statistical tests such as Chi2, Student’s t-test, Spearman and Mann Whitney depending on the distribution of the variable analyzed. A *p* < 0.05 was established as the level of statistical significance.

**Results:** A total of 62 patients were analyzed, of whom 35.48% went to intensive care unit (ICU) and 64.54% to general ward (GW). Two groups were analyzed in relation to the use of BDT: exposed and not exposed. Patients exposed to BDT who required ICU (31.82%) presented a median LSICU in hours (h) of 50 h (IQR 29–216) compared to 27 h (IQR 14–38) of non-exposed (*p* = 0.012). Patients exposed to BDT who required GW (20%) presented a median LSGW in days (D) of 5 D (IQR 1.5–5.5) compared to 3 D (IQR 1–4.5) of non-exposed (*p* = 0.25). Different SDV and their impacts on LSICU and LSGW were analyzed in both groups, as shown in the Table.

**Conclusions:** BDT in perioperative HEP affects the LSICU but not LSGW. SDV were not related to LSICU or LSGW, except for BMI, which linked obesity with more LSICU. We acknowledge the limitations of our study, owing to the small sample size.

**Reference**

1. Lyu X et al. Oncotarget. 2017;8:41740–48

**Table (abstract P122):** Sociodemographic variables of the study population

	GW (N:40) Transfusion	GW (N:40) Transfusion	ICU (N: 22) Transfusion	ICU (N: 22) Transfusion
N: 62	YES (N:8) N:(LS(IQR))*	NO(N:32) N:(LS(IQR))*	YES (N:7)N:(LS(IQR))*	NO N:(LS(IQR))*
Sex: (M vs. F)	N:3(2(0–9)) versus N:5(5(5–5)) *0.56	N: 17(3(0–5)) versus N: 15(3(1–4)) *0.56	N: 1 (264) versus N:6(48(29–96)) *0.7	N:7(27(1–50)) versus N:8(26(19–33.5)) *0.7
Age: (60 years)	N:1(1) versus N:7(5(3–6))*0.78	N:12(3(1–5)) versus N:20(2.5(1–4)) *0.78	N:1(50) versus N:6(71(29–216)) versus	N:7(27(0–38)) versus N:8(26(23–39))
Weight (Kg)	68.5(SD 13.43) *0.37	67.71(SD 9.63)*0.37	69 (SD 17.31)*0.27	66.6 (SD15.31)*0.27
BMI: (normal weight vs. overweight vs. obese)	N:5(5 (5–6)) versus N:2(2.5(0–5)) versus N:1(3)*0.98	N:18(2(0–5)) versus N:13(3(2–4)) versus N:1(3)*0.98	N:2(39.5(29–50))/N:2(36(26–46)/N:3(216(96–264)0.04	N:8(24(7–33))/N:4(27(IQR13-27))/N:3(50(22–58)0.046
Comorbidities: (less than 3 vs. 3 or more)	N:5(5(3–5)) vs. N:3(5 (0–9))*0.9	N:14 (3(2–4)) versus N:18(2.5(1–5)) *0.9	N:6(71(29–216)) versus N:1(50)*0.22	N:6(27.5(27–50)) versus N:9(22 (1–29))*0.22
Pathology: (metastasis vs. hepacarci vs. other:)	N:3(6(5–9)) versus N:3(3(0–5)) versus N:2(2.5(0–5))*0.78	N:12(2 (0–4)) versus N:9(3(1–4)) versus N:11(3(2–5)) *0	N:3(96(50–264)) versus N:0(1) versus N:4(37.5(27.5–131))	N:10(26(0–28)) versus N:1(50) versus N:4(30(18–55))*0.61

*p value: correlated with length of hospital stay and intensive care unit stay, respectively. IQR: interquartile ranges, SD: standard deviation, LS: median length of stay in GW (days) or ICU (hours), N: number of patients.

## P123 What is the FiO_2_ delivered between a non-rebreathing mask (NRM), a combined NRM and nasal cannula, and a high flow nasal cannula? A bench study

### L Rosier^1^, J Jacques^2^, M Verde^2^, G Sauvage^3^, F Duprez^4^

#### ^1^Emergency Care and URICE Epicura Hospital Hornu Belgium, Emergency Care and URICE, Hornu, Belgium, ^2^Epicura, Emergency, Hornu, Belgium, ^3^Haute École Condorcet, Haute École Condorcet, Tournai, Belgium, ^4^Epicura, ICU, Hornu, Belgium

*Critical Care* 2024, **28(Suppl 1):** P123

**Introduction:** During the COVID-19 pandemic, many patients developed acute hypoxic respiratory failure. In this case, a high-flow nasal cannula (HFNC) device was preferred to achieve satisfactory oxygenation. For some clinicians, in order to achieve sufficient oxygenation when HFNC is not available, supplemental oxygen therapy through a nasal cannula (NC) (max 6 L/min) could be added to a non-rebreathing mask (NRM) at 15 L/min [1]. However, the FiO_2_ values delivered by this improvised system (IS) are not known. The aim of this study was to compare, on a bench, the FiO_2_ value delivered by these devices.

**Methods:** In a bench study, we analysed the FiO_2_ delivered by (a) NRM alone (15 L/min) (b) a combination of NRM (15 L/min) and a nasal cannula (6 L/min) c) HFNC (FiO_2_ 100% and flow 60 L/min). To simulate ventilation, we used an adult lung model (Michigan Instrument Inc., Grand Rapids, MI), driven by a mechanical ventilator (Servoi™ Maquet) in volume control mode, peep of 0 cmH_2_O. The resistive and elastic characteristics of the test lung were set at 5 cmH_2_O/L/s and 0.06 L/cm H_2_O, respectively. Data acquisition was carried out using Labscribe 3™ software (Iworx®, United States). The parameters were modified as follows: Minute Ventilation (MV) for 10, 15 and 20 L/min and Ti/Ttot of 0.33 and 0.25. The experiment was repeated three times. A one-way repeated measures of variance or Friedman test was performed to analyse the differences between results.

**Results:** See the Figure.

**Conclusions:** MV has a negative impact on FiO_2_. NRM at 15 L/min O_2_ does not achieve 100% FiO_2_. The combination of NRM (15 L/min O_2_) and NC (6 L/min O_2_) allows an increase in FiO_2_, but the impact in absolute values is limited (+ 8.7%). HFNC offers the highest FiO_2_ value but it does not reach 100% and is influenced by MV. Some results are consistent with previous studies [2, 3].

**References**

1. Kumar A et al. Trends in Anaesthesia & Critical Care 2021;38:24–25
2. Duprez F et al. J Clin Monit Comput. 2022;36:1441–1448
3. Duprez F et al. Respir Care 2022;67:322–330

**Figure (abstract P123):**
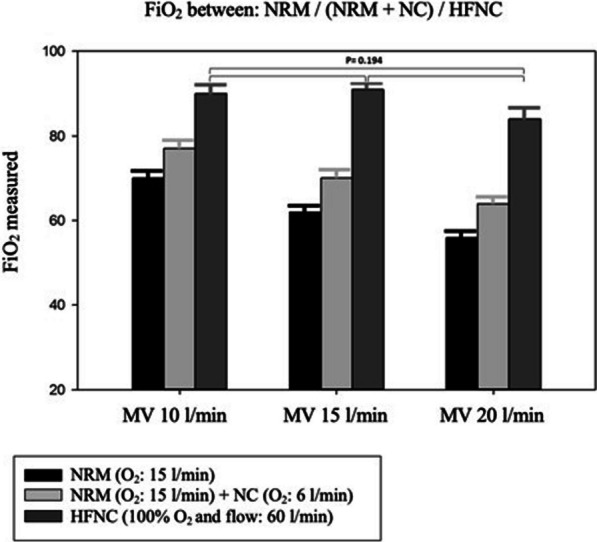
Non-rebreathing mask (15 L/min O_2_)—Non-rebreathing mask (15 L/min O_2_) associated with a nasal cannula (6 L/min O_2_)—High flow nasal cannula (100% FiO_2_ and 60 l/min flow gas). The MV was analysed at 10, 15 and 20 L/min.

## P124 Rescue helmet noninvasive ventilation after high-flow oxygen failure in COVID-19 patients with do-not-intubate order

### M Cesarano, J Vargas, D Settanni, F Del Tedesco, ES Tanzarella, G Pintaudi, SL Cutuli, G De Pascale, M Antonelli, DL Grieco

#### Fondazione Policlinico Universitario A. Gemelli IRCCS, Department of Emergency, Intensive Care Medicine and Anesthesia, Rome, Italy

*Critical Care* 2024, **28(Suppl 1):** P124

**Introduction:** Our study aims at evaluating the outcomes of elderly COVID-19 patients with do-not-intubate (DNI) order who received helmet noninvasive ventilation (HNIV) after meeting objective criteria indicating failure of high-flow oxygen therapy.

**Methods:** In this single-center prospective observational study (2020–2022), we enrolled consecutive patients (1) aged > 80 years, (2) who had COVID-19 respiratory failure, (3) had DNI directives, and (4) received HNIV after meeting objective criteria indicating high-flow failure. These included two of the following: unbearable dyspnea, respiratory rate > 35 breaths per minute, PaO_2_/FiO_2_ < 100 mmHg or SpO_2_ < 90% with FiO_2_ ≥ 70%. Helmet was applied continuously for at least 24 h with PEE* p* = 12 cmH_2_O and pressure support = 12 cmH_2_O. The primary outcome was in-hospital mortality.

**Results:** 77 patients were included (55 males (65%), median [IQR] age 82 [81–84] years, PaO_2_/FiO_2_ during high-flow oxygen 89 [69–113], respiratory rate 30 breaths per minute [25–35]). The overall duration of treatment was 48 h [29–79]. Survival rate to hospital discharge was 38% [95% CI 27–49%]. With HNIV, PaO_2_/FiO_2 _after 24 h improved irrespectively of the subsequent outcome (90 vs. 144 mmHg in patients who survived, 89 vs. 140 mmHg in patients who died, *p* = 0.19) [Figure]. At the multivariate analysis, independent predictors of survival to hospital discharge were lower SAPSII (OR 0.66 [95% CI 0.47, − 0.93]) and lower respiratory rate before treatment start (OR 0.69 [95% CI 0.48, − 0.97]).

**Conclusions:** In elderly patients with COVID-19 hypoxemic respiratory failure refractory to high-flow oxygen and DNI order, HNIV applied continuously for at least 24–48 h and with standardized settings was associated to 38%-possibility of being discharged alive from the hospital. Treatment success was more likely in patients with lower SAPS II and lower respiratory rate before treatment start, while it was independent from the oxygenation benefit produced by HNIV.

**Figure (abstract P124):**
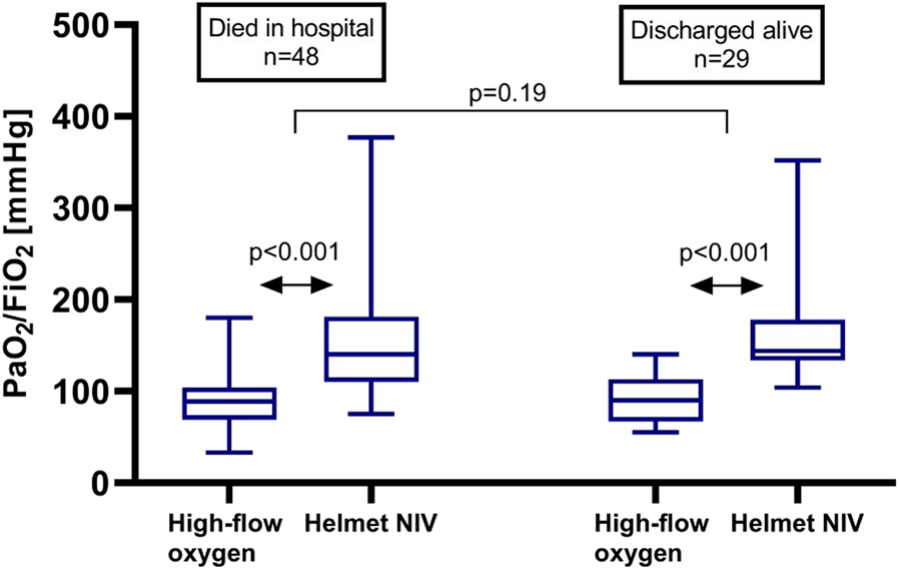
PaO_2_/FiO_2_ before treatment and after 24 h of HNIV in both groups.

## P125 Predictors of non-invasive ventilation failure for community acquired pneumonia: a retrospective cohort study

### A Watson^1^, D Fritche^1^, T Roe^1^, C Thomas^1^, K Saeed^2^, A Dushianthan^1^

#### ^1^University Hospital Southampton, General Intensive Care Unit, Southampton, UK, ^2^University Hospital Southampton, Department of Microbiology, Southampton, UK

*Critical Care* 2024, **28(Suppl 1):** P125

**Introduction:** The use of non-invasive ventilation (NIV) for community acquired pneumonia (CAP) is not routinely recommended. NIV failure is associated with increased mortality, highlighting the need for careful patient selection. We aimed to identify predictors of NIV failure for severe CAP in our ICU.

**Methods:** In this single-center retrospective cohort study, we included consecutive adults with CAP who received NIV as their initial respiratory support on our ICU. The study data was collected for the period between 1st February 2016 and 30th April 2017. We categorized patients as either NIV success (defined as discharged alive from ICU) or NIV failure (defined as requirement for mechanical ventilation or death). Sequential Organ Failure Assessment (SOFA) score, Heart Rate, Acidosis, Consciousness, Oxygenation, and Respiratory Rate (HACOR) score and ratio of oxygen saturations (ROX) index at various timepoints are reported.

**Results:** We included 106 patients (median age 63 years, 56% male). Median PaO_2_/FiO_2_ ratio and SOFA score on ICU admission were 155 mmHg and 5 respectively. Overall, our NIV success rate was 59% and in patients with NIV success, 28-day mortality was lower than for patients who failed NIV (13 vs 35%, *p* = 0.0085). In univariate analysis, NIV failure was associated with SOFA score (OR 1.33), HACOR score (OR 1.14) and presence of septic shock (OR 3.99). SOFA score has an AUC of 0.75 for NIV failure on ICU admission, whilst HACOR has an AUC of 0.76 after 2 h of NIV. A threshold of HACOR ≤ 5 after 2 h of NIV predicts success with sensitivity and specificity of 53% and 85%, whereas SOFA ≤ 4 has a sensitivity and specificity of 61% and 72%. There were no differences in pH, PaO_2_/FiO_2_ ratio, PaCO_2_ or ROX index between NIV success or failure at any timepoint.

**Conclusions:** Our results suggest that SOFA ≤ 4 and HACOR ≤ 5 are reasonable thresholds to identify patients with severe CAP likely to benefit from NIV.

## P126 Impact of ultrasound settings on B-lines: an exploratory study in mechanically ventilated patients

### J Leote^1^, A Gonçalves^2^, J Fonseca^2^, D Guerreiro^2^, H Dias^2^, I Ribeiro^2^, R Meireles^2^, R Varudo^1^, J Bacariza^1^, F Gonzalez^1^

#### ^1^Hospital Garcia de Orta, EPE, Almada, Critical Care, Almada, Portugal, ^2^Escola Superior de Tecnologia da Saúde de Lisboa, Instituto Politécnico de Lisboa, Lisboa, Portugal

*Critical Care* 2024, **28(Suppl 1):** P126

**Introduction:** The number of B-lines in lung ultrasound (LUS) impacts patients clinical management. This study aimed to demonstrate the US settings influence on the number of B-lines in patients under invasive mechanical ventilation (IMV).

**Methods:** Patients were prospectively recruited for LUS recordings including three breathing cycles with a motionless curvilinear probe on the thoracic region with more B-lines. Three clinicians were randomly enquired for the number of B-lines in baseline LUS and, blindly, after altering US settings for a total of 20 test recordings. The number of B-lines (mean ± standard deviation (SD)) across clinicians was compared between recordings.

**Results:** Twenty-nine patients (mean age 58 ± 18 years) admitted to critical care (mean SOFA score of 6.9 ± 3.3; mean delta SOFA of 2.4 ± 1.6) were under IMV due to neurological (n = 19) and respiratory illness (n = 10). They were evaluated at day 4 (± 2.6 days) of passive ventilation (plateau pressure 16 ± 3 mmHg; PEEP 6 ± 2 mmHg). On LUS day, patients had a fluid balance of 835 ± 1326 mL, an ultrasound-driven cardiac index of 3.1 ± 0.7 mL/min/m^2^ and seven were under norepinephrine (0.5 ± 0.7 mcg/kg/min). Baseline recordings showed a mean number of 1.6 ± 1.2 B-lines from a total of 87 clinicians classifications. Clinicians classifications were grouped in grades (grade 0, one to two B-lines: 59; grade I, three to six B-lines: 25; grade II, above seven B-lines: 3). The classifications agreement level was strong (Kendall’s coefficient of 0.77, *p* < 0.002). The probe frequency of 4 MHz (vs. 6/8 MHz), a gain of 90% (vs. 80%), and dynamic range of 84 dB (vs. 60 dB) increased the B-lines number by 0.4 ± 0.03 (Friedman pairwise comparison test, *p* < 0.03). US post-processing tools such as frame averaging, image enhancement or artifact, and speckle reduction decreased the B-lines number by 0.9 ± 0.24 (*p* < 0.007).

**Conclusions:** In this study, the US settings mildly influenced the number of B-lines but had a minor impact on clinical practice grades (± one B-line).

## P127 Total signal intensity of ultrasound laboratory vertical artifacts: a semi-quantitative tool

### J Leote^1^, R Loução^2^, M Aguiar^3^, M Tavares^3^, P Ferreira^3^, T Muxagata^3^, D Guerreiro^3^, H Dias^3^, J Bacariza^4^, F Gonzalez^4^

#### ^1^Hospital Garcia de Orta, EPE, Almada, Critical Care, Almada, Portugal, ^2^ University Hospital Cologne, Centre for General Neurosurgery, Cologne, Germany, ^3^Escola Superior de Tecnologia da Saúde de Lisboa, Instituto Politécnico de Lisboa, Lisboa, Portugal, ^4^Hospital Garcia de Orta, EPE, Almada, Portugal

*Critical Care* 2024, **28(Suppl 1):** P127

**Introduction:** Quantitative proposals to improve lung ultrasound (LUS) vertical artifacts (VA) interpretation using total signal intensity (I_TOT_), are not widely available for clinical practice [1–3]. In this study, we aimed: (i) to develop a mathematical algorithm to extract I_TOT_ as a post-hoc LUS analysis, and (ii) to confirm I_TOT_ utility by conducting laboratory VA research using an in vitro model with different acoustic channels.

**Methods:** The I_TOT_ was extracted from static and conventional LUS imaging recorded from in vitro models after varying the amount of water content or the pores size of the phantom, in comparison with a control condition.

**Results:** The developed algorithm was able to calculate the I_TOT_ from all phantoms. Mean I_TOT_ showed statistically significantly different values across phantom categories.

**Conclusions:** We demonstrate that I_TOT_ may be able to differentiate the in vitro acoustic channels formed by increased water content from those with small size pores. However, the utility of this semi-quantitative tool in clinical practice or other LUS imaging data sets remains unclear.

**References**

1. Leote J et al. Ultrasound Med Biol. 2023;49:1901–1908
2. Mento F et al. Ultrasound Med Biol. 2022;48:2398–2416
3. Mento F et al. Ultrasonics. 2023;135:107143

## P128 Sidestream-dark field videomicroscopy for the in vivo evaluation of the pulmonary alveoli and microcirculation in mechanically ventilated pigs

### E Casarotta^1^, C Di Bella^2^, S Zuccari^3^, M Galosi^2^, F Serino^2^, A Angorino^2^, AM Tambella^2^, A Donati^1^, E Damiani^1^

#### ^1^Università Politecnica delle Marche, Department of Biomedical Sciences and Public Health, Ancona, Italy, ^2^Università di Camerino, School of Biosciences and Veterinary Medicine, Matelica, Italy, ^3^Azienda Ospedaliero-Universitaria delle Marche, Emergency Department, Ancona, Italy

*Critical Care* 2024, **28(Suppl 1):** P128

**Introduction:** Under mechanical ventilation, the pulmonary microcirculation can be affected by the expansion or collapse of alveoli, resulting in a change in pulmonary vascular resistance. Sidestream-dark field (SDF) videomicroscopy has been used in animal models to assess pulmonary microcirculation in vivo. This study aimed to evaluate whether different mechanical ventilation settings could affect the alveolar size and pulmonary vessels in a porcine model.

**Methods:** This experimental study was conducted in four healthy pigs on mechanical ventilation under general anesthesia. The ventilation was initially set at a tidal volume (VT) of 8 mL/kg, PEEP 5 cmH_2_O, and FiO_2_ 50%. Access to the thoracic cavity was obtained through surgical thoracotomy. The subpleural pulmonary microcirculation was assessed using SDF videomicroscopy at different ventilator settings: VT 8 mL/kg, PEEP 5 cmH_2_O, FiO_2_ 50%; VT 12 mL/kg, PEEP 5 cmH_2_O, FiO_2_ 50%; VT 8 mL/kg, PEEP 12 cmH_2_O, FiO_2_ 50%; VT 8 mL/kg, PEEP 5 cmH_2_O, FiO_2_ 100%. We calculated the diameter of the alveoli and extra-alveolar microvessels.

**Results:** Comparing VT 8 mL/kg and VT 12 mL/kg, we observed a significant increase in alveolar diameter (89 [70.6–114.7] μm vs. 94.6 [78.3–115] μm, *p* = 0.04) and a significant decrease in vessels diameter (10.4 [8.6–12.7] μm vs. 9.2 [7.6–11.2] μm, *p* < 0.01). We did not observe a significant difference in alveolar and vessels diameters after changing the PEEP from 5 to 12 cmH_2_O. Increasing the FiO_2_ from 50 to 100%, the alveolar diameter significantly raised (86.7 [69.6–112.6] μm vs. 94 [72.7–122.5] μm, *p* = 0.03) as well as the vessels diameter (10.4 [8.5–12.5] μm vs. 12.2 [10.3–14.7] μm, *p* < 0.01). Subpleural pulmonary microcirculation is shown in the Figure.

**Conclusions:** Mechanical ventilation affects alveolar and pulmonary vessel size. SDF microscopy represents a valid tool to assess the subpleural pulmonary microcirculation in vivo in porcine models.

**Figure (abstract P128):**
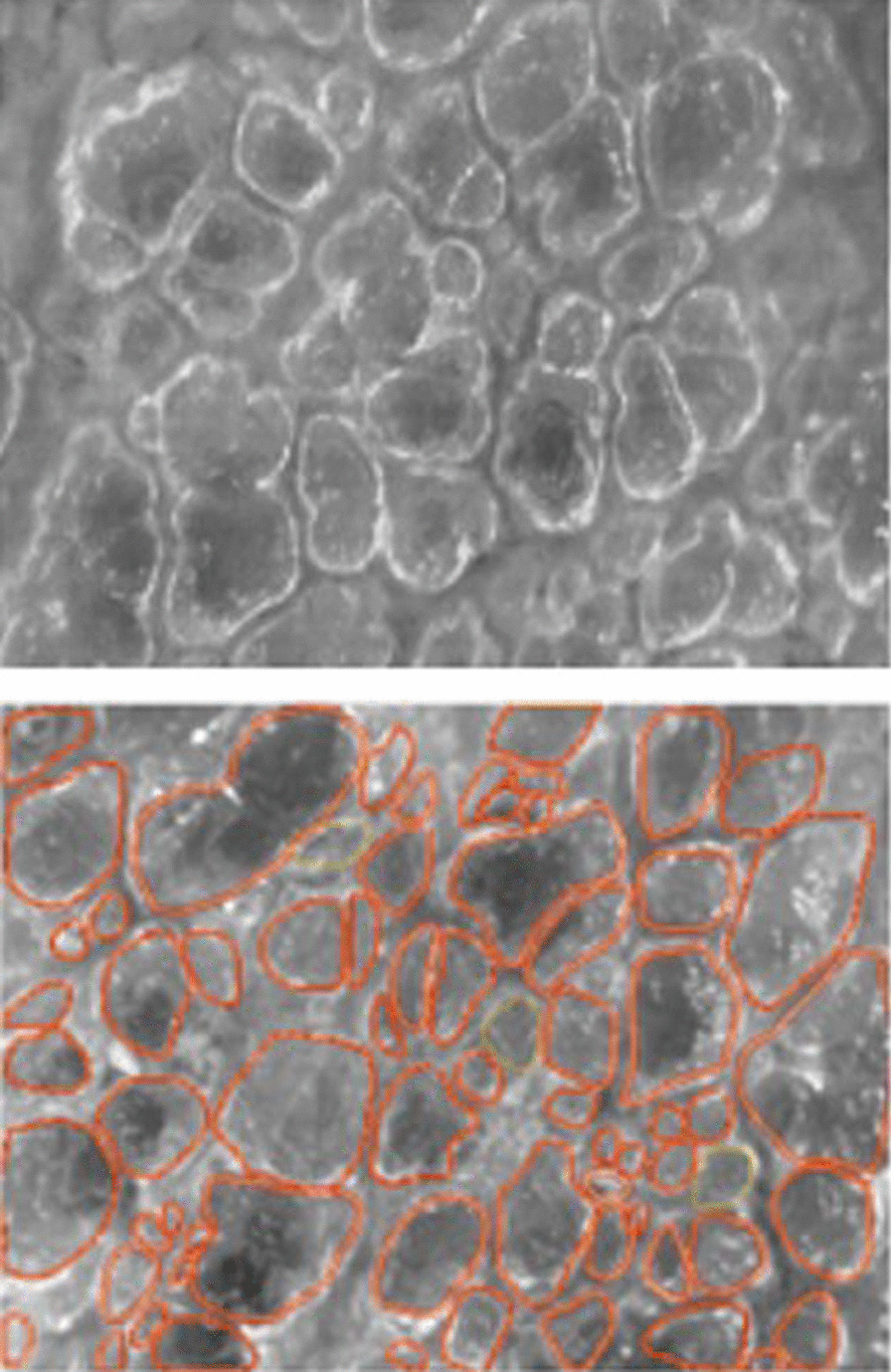
Subpleural pulmonary microcirculation.

## P129 Augmenting simulated pressure support ventilation data using adversarial learning for asynchrony detection

### THGF Bakkes^1^, L Hao^2^, A van Diepen^3^, N Chennakeshava^3^, RA Bouwman^4^, AJR De Bie Dekker^4^, P Woerlee^3^, F Mojoli^5^, M Mischi^3^, S Turco^3^

#### ^1^Eindhoven University of Technology, Electrical Engineering, Eindhoven, Netherlands, ^2^Systems Engineering Research Institute, Beijing, China, ^3^Eindhoven University of Technology, Eindhoven, Netherlands, ^4^Catharina Hospital, Eindhoven, Netherlands, ^5^Policlinico San Matteo, Pavia, Italy

*Critical Care* 2024, **28(Suppl 1):** P129

**Introduction:** In this study, we introduce a novel approach for generating synthetic ventilation waveforms with labels, based on generative adversarial networks (GANs). These waveforms serve as training data for machine-learning algorithms to improve detection and classification of patient-ventilator asynchrony (PVA), an adverse event during mechanical ventilation [1, 2].

**Methods:** GANs refine simulations of patient-ventilator interactions incorporating ventilator-specific and patient-specific factors while employing adversarial learning and self-regularization to preserve the labeling of the simulations. We evaluated the use of the generated data for training PVA detection and classification methods, comparing it to the original simulated data [3].

**Results:** The use of generated data translated into enhanced PVA detection and classification. Notably, the detection method trained on generated data showed improved accuracy in locating the patients' respiration which resulted in an increased classification performance when validated with an independent clinical dataset from a different hospital (Table). Although the detection method trained on generated data excelled across various PVAs, detecting “ineffective effort” presented challenges due to inherent noise in real clinical data.

**Conclusions:** This study underscores the potential of GANs in improving PVA detection and classification, contributing to improved quality of mechanical ventilation. Moreover, the proposed approach could be translated to other areas where labeled data are scarce.

**References**

1. Bakkes T et al. Comput Methods Programs Biomed 2023;230:107333
2. Sassoon CS et al. Curr Opin Crit Care 2001;7:28–33
3. van Diepen A et al. J Clin Monit Comput 2022;36:1739–1752

**Table (abstract P129):** Detection result for simulated and generated

Metric	Simulated data	Generated data
Recall	94.8%	91.4%
Precision	98.5%	99.3%
RMSE In	0.099 s	0.085 s
RMSE Ex	0.097 s	0.073 s

RMSE (Root mean squared error).

## P130 Examining social determinants of care in ventilated patients in critical care

### G Angelotti^1^, J Gallifant^2^, J Byers^3^, P Morandini^1^, A Carrel^4^, N Dundas^5^, LL Weishaupt^6^, LM Hampton^5^, T Wang^7^, LA Celi^8^

#### ^1^IRCCS Humanitas Research Hospital, Artificial Intelligence Center, Milan, Italy, ^2^Massachusetts Institute of Technology, Laboratory for Computational Physiology, Cambridge, MA, USA, ^3^Beth Israel Deaconess Medical Center, Respiratory Therapy, Boston, MA, USA, ^4^Imperial College London, London, UK, ^5^Massachusetts Institute of Technology, Electrical Engineering and Computer Science, Cambridge, MA, USA, ^6^Harvard & MIT, Health Sciences and Technology, Cambridge, MA, USA, ^7^Ronald Reagan UCLA Medical Center, Los Angeles, USA, ^8^Beth Israel Deaconess Medical Center, Division of Pulmonary, Critical Care, and Sleep Medicine, Boston, MA, USA

*Critical Care* 2024, **28(Suppl 1):** P130

**Introduction:** This study investigates non clinical factors affecting adherence to clinical turning protocols in mechanically ventilated patients; factors we call social determinants of care (SDoC). Social determinants of health are known contributors to health outcomes, however the impact of SDoC on clinical care has yet to be investigated. Utilizing the MIMIC-IV database, we analyzed a cohort of 8919 patients to identify disparities in care related to social factors.

**Methods:** The study included all patients who underwent invasive mechanical ventilation (IMV), excluding cases with missing weight data or weights outside the 10–250 kg range. Frequency of turning documentations per day were evaluated and compared using Kolmogorov–Smirnov tests and predictive models such as ridge regression, to assess adherence to turning protocols during IMV. These methods were cross-validated and included varying degrees of artificially injected noise for robustness.

**Results:** The patient cohort mean age was 63.5 years, with 58% males, white ethnicity (61.2%), and 88.8% reported English as their first language. There was a significant difference between the observed and simulated turning frequencies (*p* < 0.05), indicating poor adherence to local protocols, as confirmed by Fisher's method over the two-sided Kolmogorov–Smirnov test. Furthermore, regression on the quantile space, reflecting the relationship between patient weight and turning frequency, yielded a low R^2^ score (ranging from 0.0185 to − 0.0166), suggesting high variance across quantiles. A positive correlation between weight and turning frequency was significant across all noise thresholds and Fisher’s aggregation (*p* < 0.05) (Figure).

**Conclusions:** Our findings indicate significant disparities in the adherence to IMV care protocols in ICUs, influenced by patients weight. The variance in care frequencies, irrespective of clinical features, highlights the need for tracking such features to encourage more equitable ICU care practices.

**Figure (abstract P130):**
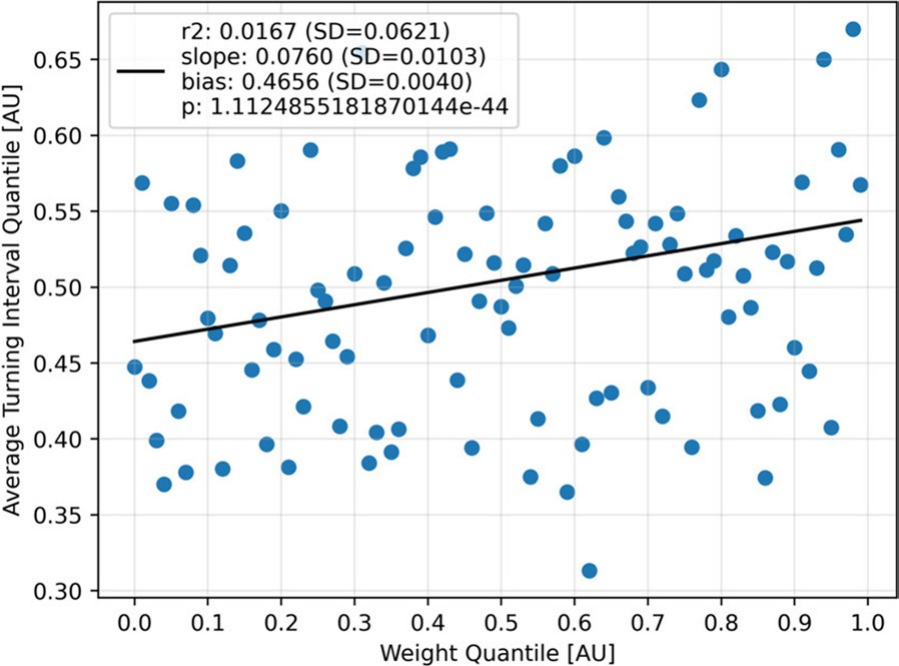
Correlation between the quantiles of weight and the quantiles of the observed average turning interval.

## P131 Mechanical ventilation strategy during cardiopulmonary bypass. Impact on outcomes and pulmonary complications in the intensive care unit

### I Vakhrushev, T Li, A Kuanyshbek, S Tulegenov, P Bukirova

#### National research cardiac surgery center, CICU, Astana, Kazakhstan

*Critical Care* 2024, **28(Suppl 1):** P131

**Introduction:** The immediate post-operative phase following cardiac surgery is a delicate and difficult phase in which serious complications can occur. One of the most dangerous complications is respiratory insufficiency, which is aggravated by the restriction of chest movement, haemodilution and non-physiological blood circulation during cardiopulmonary bypass (CPB). The impact of mechanical ventilation settings during CPB on the postoperative period is still debated.

**Methods:** Prospective, randomised, controlled study at one centre. Adult patients undergoing on-pump cardiac surgery (coronary artery bypass grafting (CABG)) by sternotomy for coronary artery disease were included. Patients were randomised into two groups—one group that received mechanical ventilation and one group that did not receive ventilation during CPB. The main endpoint was PaO_2_/FiO_2_ as a marker for the quality of ventilation and perfusion measured in the ICU in the immediate postoperative period. Secondary endpoints were driving pressure, minute ventilation and postoperative pulmonary complications such as atelectasis and acute respiratory distress syndrome. Atelectasis was diagnosed using the USI method. The patients in both groups were comparable with regard to the primary parameters.

**Results:** Fifty-two consecutive patients were included, 25 and 27 in each group. No significant difference was found in the PaO_2_/FiO_2_ ratio in the groups (*p* = 0.06). A significant difference was found in driving pressure 10.13 ± 2.76 versus 8.75 ± 3.1 cmH_2_O with *p* = 0.03. More complications such as acute respiratory distress syndrome and USI signs of atelectasis (5 vs. 16) were observed in the non- ventilated group. Results are shown in the Table.

**Conclusions:** Prolonged absence of mechanical ventilation during CPB may lead to an increase in cases of atelectasis. Maintaining mechanical ventilation during CPB may be beneficial for patients undergoing cardiac surgery. Strategy of mechanical ventilation during CPB need in further research.

**Table (abstract P131):** Results

	Ventilated group (n = 25) [mean ±]	Non ventilated group (n = 27) [mean ±]	*p* value (95% CI)
PaO_2_/FiO_2_	315.33 ± 67.31	283.86 ± 76.26	0.06
Driving pressure	10.13 ± 2.76	8.75 ± 3.1	0.03
MV	6.48 ± 3.15	8.05 ± 2.93	0.06
PCO_2_	43.17 ± 17.82	39.26 ± 21.64	0.27
ARDS	0	1 (3.7%)	
Atelectasis	5 (20%)	16 (59.2%)	

## P132 Open-lung ventilation versus no ventilation during cardiopulmonary bypass in an innovative animal model of heart transplantation

### SM Colombo^1^, V Karnik^1^, L Rickards^2^, L See Hoe^1^, K Wildi^1^, M Passmore^1^, J Suen^1^, D McGriffin^3^, J Fraser^1^, G Li Bassi^1^

#### ^1^Critical Care Research Group, Brisbane, Australia, ^2^Sunshine Coast Hospital, Sunshine Coast, Australia, ^3^Alfred Hospital, Melbourne, Australia

*Critical Care* 2024, **28(Suppl 1):** P132

**Introduction:** Open-lung ventilation is a potential strategy to mitigate acute respiratory failure following cardiac surgical procedures with cardiopulmonary bypass (CPB), such as heart transplantation (HTx). We conducted a randomized study in an innovative ovine model of HTx to investigate whether open-lung ventilation during CPB reduces postoperative lung damage and complications.

**Methods:** Eighteen sheep received HTx either from brain dead (n = 9) or sham (no neurological injury) donors (n = 9). During the period of CPB, ventilatory interventions were randomly assigned: the OPENVENT group received low tidal volume (V_T_) of 3 mL/kg and positive end expiratory pressure (PEEP) of 8 cm of H_2_0, while no ventilation was provided in the NOVENT group, as per standard of care. The recipient sheep were monitored for 6 h post-surgery. The primary outcome was histological lung damage score at the end of study. Secondary outcomes included pulmonary shunt, driving pressure, hemodynamics, and inflammatory cell lung infiltration.

**Results:** The OPENVENT group showed significantly lower histological lung damage versus the NOVENT group (Figure: 1.05 vs. 1.51, *p* < 0.001). Pulmonary shunt (19.2% vs. 32.1%, *p* = 0.0012) and driving pressure was reduced in the OPENVENT group (9.6 vs. 12.8 cm of H_2_O, *p* = 0.04). Finally, lungs in the OPENVENT group presented lower neutrophil (5.25% vs. 7.97%, *p* < 0.001) and macrophage infiltrations (11.1% vs. 19.6%, *p* < 0.001). No significant differences were observed in hemodynamic parameters between groups.

**Conclusions:** In an ovine model of HTx, open-lung ventilation during CPB significantly reduced lung histological injury and inflammatory cell infiltration. The study highlights the value of an open-lung approach during CPB and emphasizes the need of translating these findings to decrease risks of acute respiratory failure in HTx patients.

**Figure (abstract P132):**
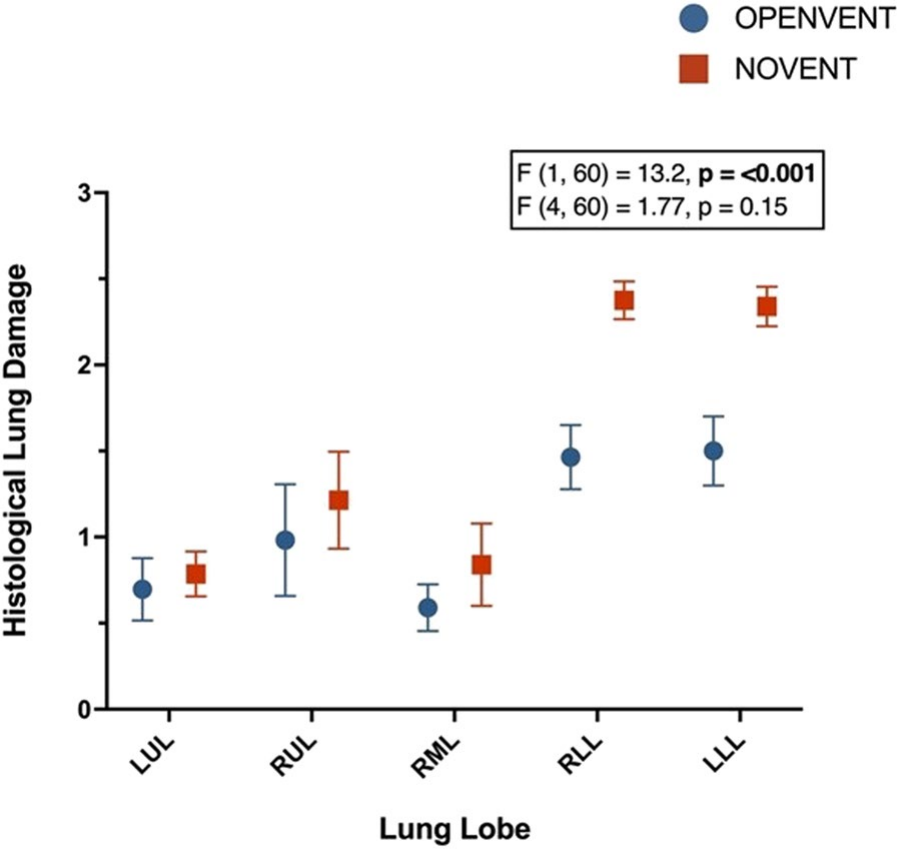
Histological damage scoring in postoperative lung samples from recipient sheep treated with either no ventilation (NOVENT ■) or open-lung ventilation (OPENVENT ●) during cardiopulmonary bypass. Data was segmented into lobes, and shown as mean ± SEM with n = 9 for both groups. 2-way ANOVA and Sidak’s test were performed with a significance threshold of 0.05. A significant difference between NOVENT and OPENVENT sheep for any given lobe is designated by ✱. Top F and *p* values represent effect of ventilation strategy, and bottom F and *p* values represent differences between ventilation strategies over time (interaction term). LUL left upper lobe, RUL right upper lobe, RML right middle lobe, RLL right lower lobe and LLL left lower lobe.

## P133 Spontaneous-breathing trials before weaning from mechanical ventilation in French ICUs: the multicenter WAVE cohort study

### L Barrot^1^, G Besch^2^, F Audibert^1^, O Abou-Arab^3^, N Mongardon^4^, PG Guinot^5^, A Tran Dinh^6^, C Roger^7^, W WAVE study group^8^

#### ^1^Jean Minjoz Hospital, Anesthesia and Intensive Care, Besancon, France, ^2^Jean Minjoz Hospital, Besancon, France, ^3^CHU Amiens, Anesthesia and Intensive Care, Amiens, France, ^4^ CHU Créteil, Anesthesia and Intensive Care, Paris, France, ^5^CHU Dijon, Anesthesia and Intensive Care, Dijon, France, ^6^Institut Mutualiste Montsouris, Anesthesia and Intensive Care, Paris, France, ^7^CHU Nîmes, Anesthesia and Intensive Care, Nîmes, France, ^8^16 ICUs, Anesthesia and Intensive Care, 14 cities, France

*Critical Care* 2024, **28(Suppl 1):** P133

**Introduction:** Weaning from mechanical ventilation (MV) is a common challenge that concerns many patients admitted in intensive care units (ICUs). Current guidelines recommend to systematically perform a spontaneous-breathing trial (SBT) to confirm decision of extubation [1]. Previous studies reported heterogeneous practices in SBT among ICUs. In particular, arterial blood gas (ABG) analysis appeared to be widely prescribed at the end of SBT in some ICUs despite no clear recommendation. The aim of the WAVE study was to describe daily practices in weaning from MV in French ICUs.

**Methods:** The observational WAVE study (NCT05801692) was conducted in 16 French ICUs between March and June 2023. All adult patients receiving invasive MV for > 48 h and undergoing a SBT were eligible. Participating ICUs were asked to include all consecutive eligible patients during a period of at least 4 weeks. The primary outcome was the modality of SBT. Secondary outcomes were the SBT duration, the SBT success, the timing of extubation and the realisation of ABGs.

**Results:** 256 patients were included (age: 62 ± 16 years; female: 82 (32%); SAPS II: 51 ± 18; SOFA: 7 ± 3; median duration of MV: 6 days (3.5–11), and 196 (76%) were extubated after a successful SBT. SBT used a T-piece and a pressure-support ventilation (PSV) in respectively 119 (46%) and 137 (53%) patients (*p* = 0.02). Secondary outcomes are presented in the Table.

**Conclusions:** PSV SBT was more often performed in participating ICUs and was more often successful than T-piece SBT. However, T-piece SBT was still used in 46% of patients [2, 3]. ABG analysis was widely prescribed regardless of the modality of SBT. Whether ABG analysis should be part of routine practices before weaning from MV deserves further studies.

**References**

1. Boles JM et al. Eur Respir J. 2007;29:1033–1356
2. Subirà C et al. JAMA. 2019;321:2175–2182
3. Thille AW. N Engl J Med. 2022;387:1843–54

**Table (abstract P133):** Results of WAVE study

	T-Piece	PSV	Statistic T-Piece versus PSV	Total n (%)
SBT type, n (%)*	119 (46.5)	137 (53.5)	*p* = 0.02	256
SBT duration, mean (min) $	37 ± 18	64 ± 55	*p* < 0.001	254
SBT success, n (%)∏	91 (76.5)	118 (86.1)	*p* = 0.001	219
Extubation during the day following SBT success	60 (65.9%)	100 (84.7%)	*p* < 0.001	160 (76.6%)
Extubation during 24–48 h following SBT success	16 (17.6%)	4 (3.4%)	*p* < 0.001	20 (9.5%)
Extubation more than 48 h following SBT success	4 (4.4%)	12 (10.2%)		16 (7.7%)
ABG during SBT, n (%)	88 (73.9)	95 (63.9)	*p* = 0.07	183 (71.2%)

SBT=spontaneous breathing trial, ABG=arterial blood gas *1 missing value, $ 3 missing data Π 3 missing data £ 13 missing data

## P134 A new method for estimating spontaneous breathing effort using changes in central venous pressure: a pig model study

### M Kyogoku^1^, T Miyasho^2^, Y Endo^3^, Y Inata^4^, M Takeuchi^4^

#### ^1^Osaka Women’s and Children’s Hospital, Intensive Care, Osaka, Japan, ^2^Rakuno Gakuen University, Hokkaido, Japan, ^3^Feinstein Institutes for Medical Research, NY, USA, ^4^Osaka Women’s and Children’s Hospital, Osaka, Japan

*Critical Care* 2024, **28(Suppl 1):** P134

**Introduction:** The standard method for evaluating spontaneous respiratory effort is by esophageal pressure measurement. However, this method requires a special device that is not readily available. In contrast, central venous catheters are commonly used in critically ill patients, and central venous pressure is known to vary depending on changes in intrathoracic pressure (ΔPpl). We previously reported a simple correction method for estimating ΔPpl using changes in central venous pressure (ΔCVP) in postoperative pediatric patients under spontaneous breathing assistance. This study aimed to clarify this method can evaluate ΔPpl, spontaneous respiratory effort from CVP waveform, without an esophageal pressure catheter, under various respiratory mechanics in adult-sized pig respiratory failure models.

**Methods:** Ten pigs (42.1 ± 1.8 kg) with lung injuries undergoing saline lung lavage procedure with spontaneous breathing assistance were selected. Each pig was subjected to 2 different chest wall compliance and 2 different airway resistance. The ratio of ΔCVP to airway pressure change during the occlusion test was measured and assumed to be constant even during normal ventilation. The magnitude of spontaneous respiratory effort during assisted ventilation was also estimated. The Bland–Altman analysis compared the estimated pleural pressure value (eΔPpl) calculated from ΔCVP with the esophageal pressure measurement value (ΔPes).

**Results:** The mean and standard deviation of ΔPes and eΔPpl were − 9.7 ± 6.6 and − 9.5 ± 6.5 cmH_2_O, respectively. Bland–Altman analysis showed that the bias was − 0.3, and the precision was 2.6 cmH_2_O (Figure).

**Conclusions:** Our method can assess spontaneous respiratory effort with reasonable accuracy in adult-sized pigs with respiratory failure without requiring an esophageal pressure catheter across various respiratory mechanics.

**Figure (abstract P134):**
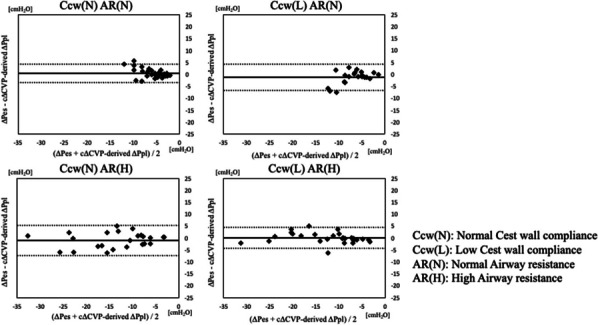
The Bland–Altman analysis for the agreement between ΔPes and cΔCVP-derived ΔPpl, classified by each condition.

## P135 Comparison of occlusion pressures and P_0.1_ in neuro and non-neuro patients considered for a spontaneous breathing trial: a single center prospective pilot study

### S Slagmulder^1^, W Baas^1^, LMA Heunks^2^, M van der Jagt^1^, AH Jonkman^1^

#### ^1^Erasmus MC, Intensive Care, Rotterdam, Netherlands, ^2^Radboud UMC, Intensive Care, Nijmegen, Netherlands

*Critical Care* 2024, **28(Suppl 1):** P135

**Introduction:** Monitoring inspiratory effort and lung stress during assisted ventilation has become an important aspect of lung- and diaphragm protective ventilation. Two noninvasive parameters are important for estimating lung stress and inspiratory effort: decrease in airway pressure during the first 100 ms of inspiration (P_0.1_) and the full-breath occlusion pressure (Pocc). Their value in ventilated patients with acute brain injury, is unclear, considering that brain injury can lead to high respiratory drive. We hypothesize that P_0.1_ and Pocc would be higher in patients with acute brain injury, compared to non-neurological ventilated patients appearing ready for extubation.

**Methods:** This prospective pilot study included mechanically ventilated patients admitted to the ICU for acute brain injury (neuro group) or primary respiratory failure (non-neuro group), estimated to be ready for a spontaneous breathing trial (SBT) and possible extubation within 24 h. Pocc and P_0.1_ values (average of 2 measurements within 5-min interval) were collected. Exclusion criteria: tracheostomy, pH < 7.35, metabolic encephalopathy and agitated delirium.

**Results:** Twenty-one patients (mean age 51 ± 17 years, mean days ventilated = 7) were enrolled (n = 8 neuro, n = 13 non-neuro). Neuro-patients showed no increase in Pocc (6.6 ± 2.8 vs. 9.2 ± 2.6 cmH_2_O) or P_0.1_ (1.2 ± 0.8 vs. 1.2 ± 0.9 cmH_2_O) compared to non-neuro patients (Figure, panels A-B). One extubation failed, in the non-neuro group. P_0.1_ values and the set support level were correlated in the neuro group (R = − 0.67, *p* = 0.012) (Figure panel D), no such correlation was observed for Pocc (Figure panel C).

**Conclusions:** P_0.1_ and Pocc values in patients considered ready for SBT did not differ significantly between patients with and without acute brain injury. Additional analysis with a larger sample size and including the effect of ventilator support on drive is necessary to determine the clinical value of monitoring drive and effort in neurological ICU patients.

**Figure (abstract P135):**
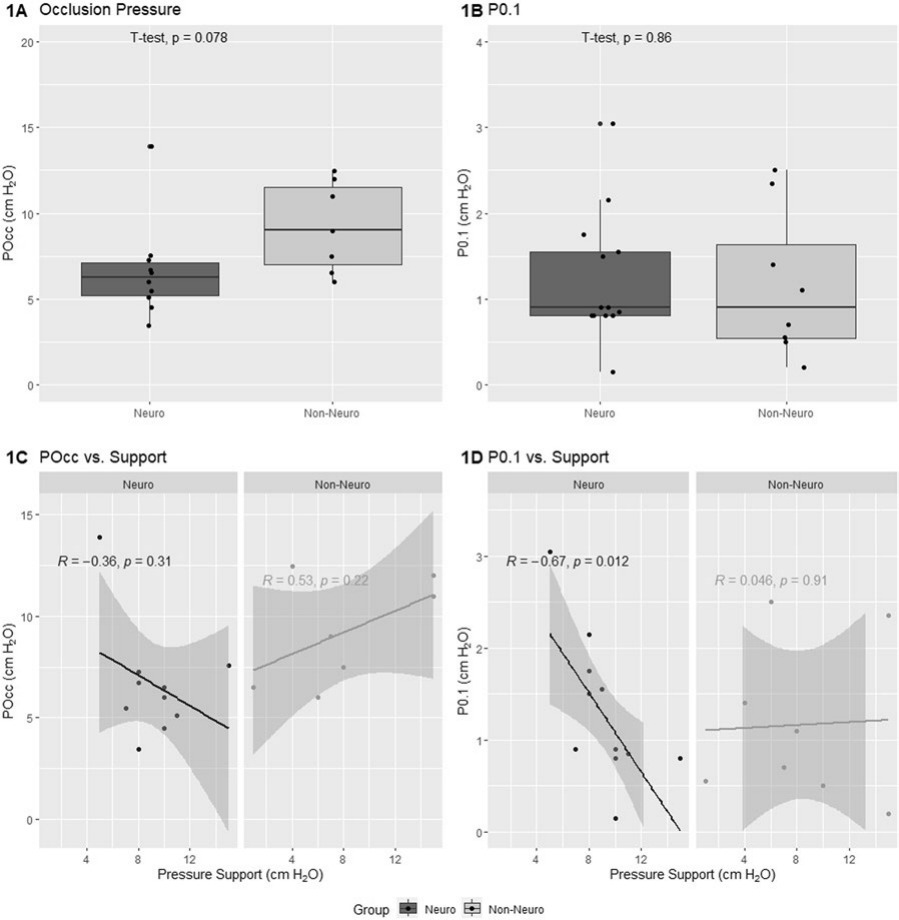
Occlusion pressures and P_0.1_ values for each group in the upper section. The lower section depicts the relationship between Pocc and P_0.1_ concerning the levels of support for each group.

## P136 Protocolized versus non-protocolized mechanical ventilation weaning in patients intubated for toxic coma: a randomised study

### A Bedhiafi^1^, A Ben Jazia^2^, H Ben Ghezala^2^, N Brahmi^2^

#### ^1^Mahmoud Yaacoub Center of Emergency Medicine and Intensive Care, Intensive Care Department, Tunis, Tunisia, ^2^Mahmoud Yaacoub Center of Emergency Medicine and Intensive Care, Tunis, Tunisia

*Critical Care* 2024, **28(Suppl 1):** P136

**Introduction:** Weaning from mechanical ventilation (MV) is a major challenge in intensive care unit (ICU). Protocolized weaning strategies are strongly recommended to mitigate associated morbidity and mortality. Patients requiring mechanical ventilation for acute intoxication constitute a distinctive population characterized by a low prevalence of comorbidities and often brief ventilation duration. This study aimed to evaluate the value of protocolizing the weaning in patients intubated for acute toxic coma.

**Methods:** This is a randomized prospective study conducted over four months in a toxicological ICU. Patients intubated for acute intoxication, meeting extubation criteria within 72 h of ventilation, were randomized into two groups: one underwent a protocolized weaning with a spontaneous breathing trial (SBT) through a 30-min zero end-expiratory pressure (ZEEP) trial and a cuff-leak test, while the second group underwent non-protocolized extubation based solely on basic clinical parameters (neurological status, blood pressure, pulse and oxygen saturation) with no SBT.

**Results:** In total, 52 patients were included, mean aged 32 (± 13) years, with a sex-ratio ratio of 0.79, SAPS-II 24 (± 3) and APACHE-II 9 (± 2). Main comorbidities were psychological disorder 59.6%, hypertension 7.7% and diabetic 5.7%. Neurological distress was the primary indication for intubation (100%), with associated respiratory distress in 3 cases (5.7%). The most frequently identified toxic agent was alpha-chloralose (40.4%), followed by carbamazepine (13.5%), benzodiazepine and tricyclic antidepressant (9.6%). Main sedation agent was propofol (92%). On analysis, the two groups were comparable in age, sex, medical history and MV duration (*p* > 0.05). The primary outcome was extubation success, and no statistically significant difference was observed between the two groups (96.1% versus 92.6%, *p* = 0.55) (Table).

**Conclusions:** This study prompt reconsideration regarding necessity of a protocolized weaning in patients ventilated for a brief toxic coma.

**Table (abstract P136):** Results

Variable	Protocolized weaning n = 26	Non-protocolized weaning n = 26	*p*
Extubation success, n (%)	25 (96.1)	24 (92.3)	0.552
IMV duration (hours)	40.9 ± 19.2	37 ± 13.4	0.403
LOS (days)	3.1 ± 2.2	3.1 ± 1.7	0.955
O_2_ after extubation, n (%)	3 (11.5)	5 (19.2)	0.213
Aspiration pneumonia	12 (46.1)	14 (53.8)	0.809
Mortality at 28 days, n (%)	0	0	> 0.99

IMV: invasive mechanical ventilation, LOS: length of stay.

## P137 Towards a digital patient twin for ICU ventilation

### C Hennigs^1^, J Sauer^1^, A Bigalke^2^, T Hardel^3^, P Rostalski^4^

#### ^1^Universität zu Lübeck, Institut of Electrical Engineering in Medicine, Lübeck, Germany, ^2^Universität zu Lübeck, Institute of Medical Informatics and Drägerwerk AG & Co. KGaA, Lübeck, Germany, ^3^Center for Anesthesiology and Intensive Care Medicine, Clinic for Intensive Care Medicine, University Medical Center Hamburg-Eppendorf, Hamburg, Germany, ^4^Universität zu Lübeck, Institut of Electrical Engineering in Medicine and Fraunhofer Research Institution for Individualized and Cell-Based Medical Engineering, Lübeck, Germany

*Critical Care* 2024, **28(Suppl 1):** P137

**Introduction:** Growing digitalization and increasing availability of sensors in the intensive care unit lead to a plethora of challenges in clinical practice. Despite the vast amount of measured and digitized data, tracking the actual patient condition and deciding on an adequate ventilation therapy remains a highly non-trivial task. This research aims at interpreting the flood of sensor data and available prior knowledge to foster accessibility and usability in intensive care ventilation.

**Methods:** A concept and a first demonstrator for a monitoring tool for mechanically ventilated patients in the ICU has been developed. The resulting patient-specific digital twin illustrates the processing of incoming sensor data, applying model-based sensor fusion, and estimating critical patient parameters in a single holistic model. The measured and estimated signals and parameters are concisely displayed in a fully integrated graphical user interface and e.g. allow the prediction of changes in the patient's condition as ventilation parameters are adjusted. A 12-h patient view allows the assessment of therapy progress and helps to decide on an adequate ventilation therapy. A monitoring tab provides live information about the current patient status.

**Results:** An initial test of the monitoring tool was performed on a combined dataset from two observational clinical trials at the University Medical Center Hamburg-Eppendorf and the Charité Berlin. Long-term ventilation, EIT, and BGA measurements are used from the first trial. The second trial provides ventilation and sEMG signals. The Figure shows screenshots of the monitoring tool.

**Conclusions:** The proposed demonstrator of this monitoring tool provides a first concept on how to collect, fuse, and visualize various measurements of mechanically ventilated patients in the ICU. The current version of this digital patient twin was only tested on a single combined patient dataset and needs to be validated in further clinical trials.

**Figure (abstract P137):**
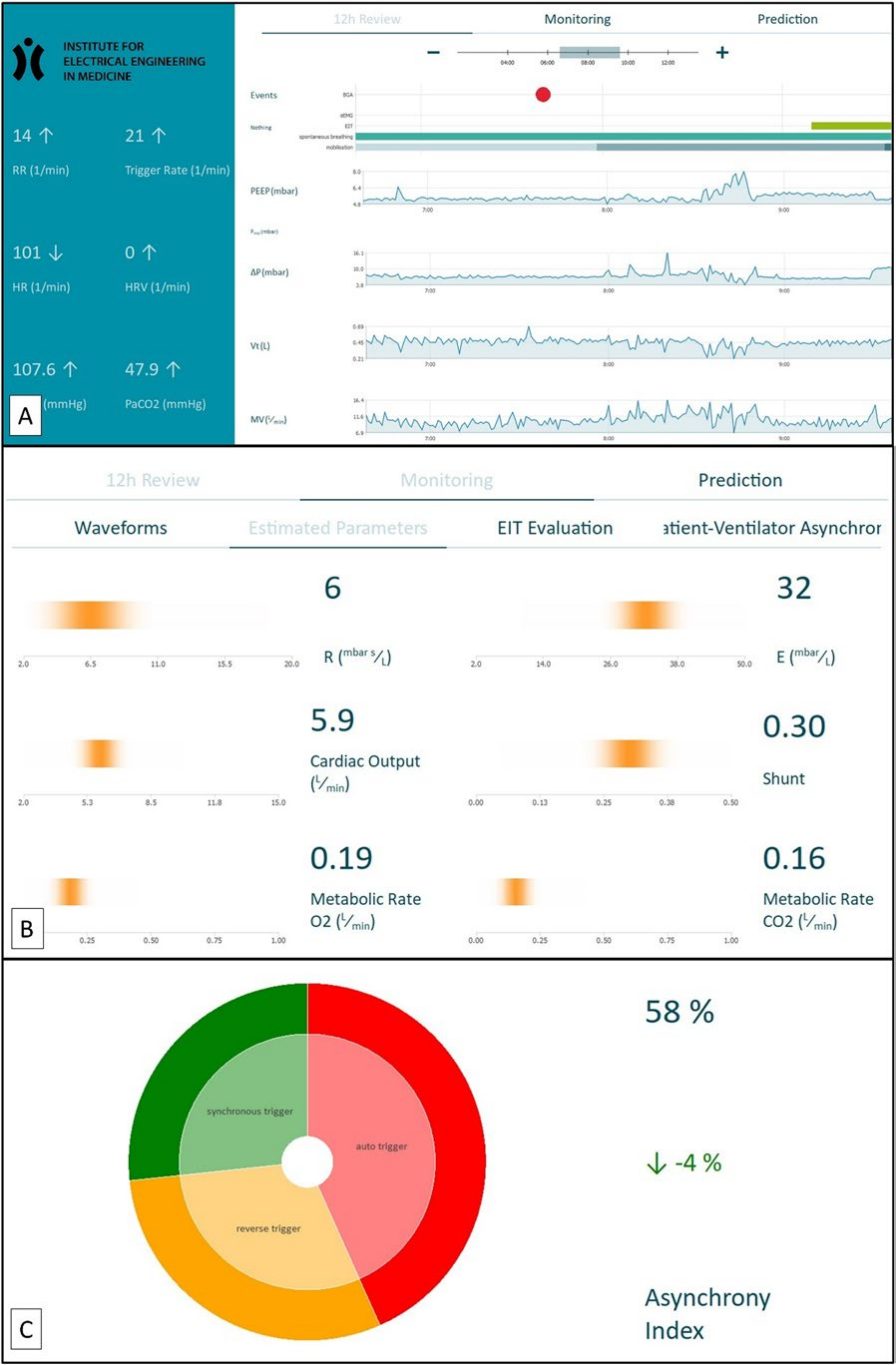
**A** 12 h patient view, **B** estimated clinical parameter view, **C** asynchrony index.

## P138 Are there abilities to improve the safety and protectiveness of mechanical ventilation in obese patients by using “intellectual” modes?

### R Komnov^1^, A Eremenko^1^, E Koshek^1^, P Titov^1^, S Gerasimenko^1^, A Urbanov^1^, P Polyakova^1^, M Fominukh^2^, A Alferova^1^, D Fomina^1^

#### ^1^Petrovsky National Research Centre of Surgery, Post-Cardiac Surgery Intensive Care Unit, Moscow, Russian Federation, ^2^Petrovsky National Research Centre of Surgery, Moscow, Russian Federation

*Critical Care* 2024, **28(Suppl 1):** P138

**Introduction:** The number of surgical patients with obesity is increasing. There are no data about using fully closed-loop “intellectual” modes in these patients. We assume that automatic management modes of mandatory and assisted ventilation allow to increase patient’s safety and decrease medical staff workload.

**Methods:** In this randomized prospective trial 80 adult cardiosurgical patients with BMI > 30 kg/m^2^ were included. 40 of them in the postoperative period were ventilated with INTELLiVENT-ASV® mode, 40—with conventional ventilation modes provided by 8 physicians. Hamilton G5 ventilators were used. Care in both groups was standardized, except management of mechanical ventilation. The primary endpoint of this study was to compare safety of respiratory support by considering driving pressure (∆P), tidal volume (V_t_), mechanical power (MP), PEEP and FiO_2_ level.

**Results:** Patients in the INTELLiVENT-ASV® group were ventilated with more protective parameters compared to conventional mode through significant reduction in the ∆P, V_t_, MP, FiO_2 _and PEEP levels. It is important, that there was no significant difference in PaO_2_/FiO_2_ ratio during all phases of respiratory support and after extubation of trachea (Table). SpO_2_ (98 (97 − 99) vs. 100 (98 − 100), *p* < 0.0001) and PaO_2_ (89 ± 9 vs. 107 ± 18 mmHg, *p* < 0.0001) were lower in the INTELLiVENT-ASV group, but in physiological ranges, in conventional modes group mild hyperoxia was met. Also, this research has showed that using of the automatic ventilation management mode is associated with a lower level of pressure support (6 (5–7) vs. 8 (6–10) mmH_2_O, *p* < 0.0001) during period of spontaneous ventilation.

**Conclusions:** The comparison of automatic management of mechanical ventilation with traditional physician-directed respiratory support after cardiac surgery in the patients with obesity showed that intellectual modes are more safe and protective in this population of patients.

**Table (abstract P138):** Main data

Variable	INTELLiVENT-ASV® group (n = 40)	Control group (n = 40)	*p*
BMI (body mass index), kg/m^2^	33.5 (32–35)	33.5 ± 2	0.5006
∆P (driving pressure), cmH_2_O	7 (6 − 8)	9 (8 − 10)	< 0.0001
Tidal volume (Vt), mL/kg/PBW	6.4 ± 0.7 CI mean 95% (6.2 − 6.6)	8.6 ± 1.3 CI mean 95% (8.1 − 9.0)	< 0.0001
PEEP, cmH_2_O	7 (6 − 8)	9 (7 − 10)	0.0001
FiO_2_	28 (24 − 30)	35 (30 − 39)	< 0.0001
Mechanical power, J/min	9 ± 3	12 (10 − 15)	< 0.0001
Ratio PaO_2_/FiO_2_	332 ± 51 CI mean 95% (316 − 349)	316 ± 58 CI mean 95% (297 − 334)	0.1746

## P139 The effect of positive end-expiratory pressure on pulmonary vascular resistance depends on alveolar recruitability in acute respiratory distress syndrome

### S Cappio Borlino, J Hagry, C Lai, E Rocca, G Fouqué, M Fasan, R Shi, T Pham, JL Teboul, X Monnet

#### ^1^Université Paris-Saclay, Hôpital de Bicêtre, Service de Médecine Intensive-Réanimation, Le Kremlin-Bicêtre, France

*Critical Care* 2024, **28(Suppl 1):** P139

**Introduction:** A U-shape relationship should exist between lung volume and pulmonary vascular resistance (PVR), with minimal PVR at functional residual capacity. Thus, positive end-expiratory pressure (PEEP) in patients with acute respiratory distress syndrome (ARDS) should increase PVR if overdistension predominates and not in case of alveolar recruitment. However, this has never been proven in patients. We aimed to study the effect of PEEP on PVR according to lung recruitability, evaluated by the recruitment-to-inflation ratio.

**Methods:** In ARDS patients, we measured hemodynamic (with pulmonary artery catheter), echocardiographic and ventilatory variables (including esophageal pressure), at high PEEP, and after lowering PEEP by 10 cmH_2_O.

**Results:** Among 54 cases measured in 20 patients, 23 cases had low recruitment potential (LRP) (recruitment-to-inflation ratio < 0.5) and 31 high recruitment potential (HRP). Lowering PEEP from 14 ± 2 to 4 ± 2 cmH_2_O reduced PVR in LRP cases (from 233 ± 98 to 177 ± 79 dynes·s/cm^5^, *p* < 0.0001), while PVR was unchanged in HRP cases (from 223 ± 68 to 220 ± 65 dynes·s/cm^5^, *p* = 0.66) (Figure). Right-to-left end-diastolic ventricular areas ratio simultaneously decreased in LRP cases (from 0.67 ± 0.14 to 0.56 ± 0.12, *p* < 0.0001), while remaining stable in HRP cases (from 0.71 ± 0.17 to 0.73 ± 0.19, *p* = 0.32). At PEEP lowering, cardiac output significantly increased only in preload responsive cases (from 3.0 ± 0.8 to 3.6 ± 1.1 L/min/m^2^, *p* < 0.0001) while it remained almost stable in preload non-responsive cases (from 3.4 ± 1.2 to 3.6 ± 1.4 L/min/m^2^, *p* = 0.06).

**Conclusions:** Changing PEEP affects PVR when inducing overdistension, while exerting no effect on PVR in case of alveolar recruitment. Tailoring PEEP on the estimated alveolar recruitability should mitigate its hemodynamic effects.

**Figure (abstract P139):**
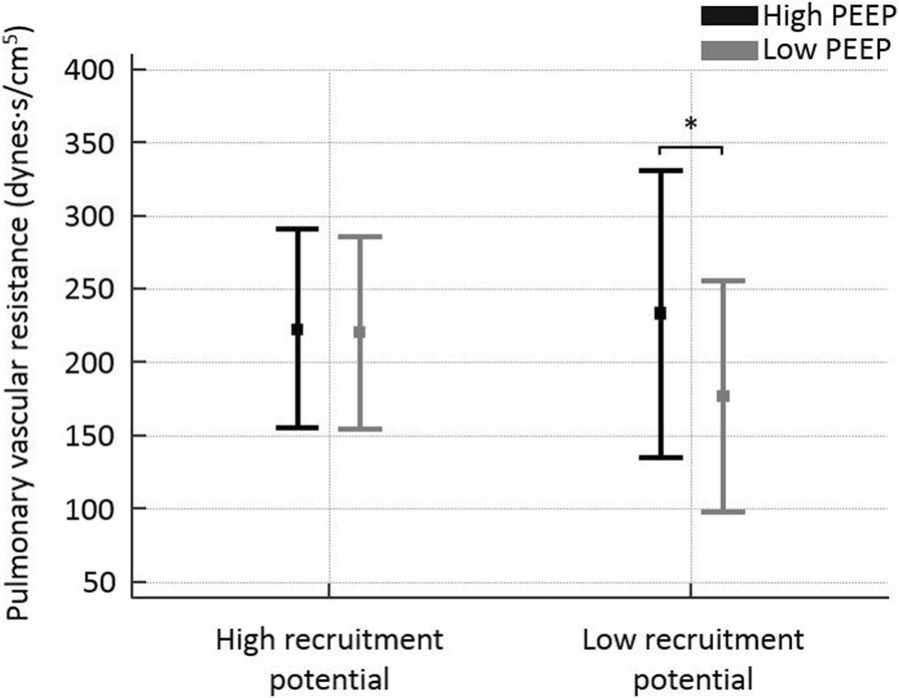
Change in pulmonary vascular resistance induced by lowering positive end-expiratory pressure (PEEP), according to lung recruitability potential. *p less than 0.05.

## P140 A method for personalized reconstruction of electrical impedance tomography static images

### I Minev

#### Medical University of Plovdiv, Department of Anaesthesiology, Emergency and Intensive Care Medicine, Plovdiv, Bulgaria

*Critical Care* 2024, **28(Suppl 1):** P140

**Introduction:** Electrical impedance tomography (EIT) is a non-invasive bedside tool for personalized monitoring of lung ventilation [1]. Although for years in clinical practice, the capabilities of the technology for monitoring the structural changes in the lung tissue are not fully revealed. That is why we aimed to develop a method for personalized reconstruction of EIT static images.

**Methods:** Based on patient’s thoracic CT scan analysis, the slice, presenting the largest intersection with the region of interest, is selected. The level of the slice defines the level of interest at which the electrodes are positioned according to patient’s anatomical Reference points. The margins of the patient’s thorax are determined by applying a fem mesh onto the CT image. Subsequently the raw data from the EIT is processed within the individualized contour of the thorax, reflecting the body structure of the patient [2].

**Results:** The method provides reliable EIT images with significant conformity to the CT images taken at the corresponding level (Figure). This personalized approach surmounts the limitations for placing the EIT electrodes [3] at different than initially recommended positions and reveals indications for potential clinical application of EIT in conditions characterized by heterogeneously disseminated or solitary lesions.

**Conclusions:** The personalized approach in reconstruction of EIT images reveals potential for optimization of monitoring of mechanical ventilation and the monitoring of lung injury dynamics at the bedside.

**References**

1. Rauseo M et al. J Anesth Analg Crit Care 2022;2:28.
2. Grychtol B et al. IEEE Trans Med Imaging. 2012;31:1754–60.
3. Karsten J et al. Crit Care 2016;20:3.

**Figure (abstract P140):**
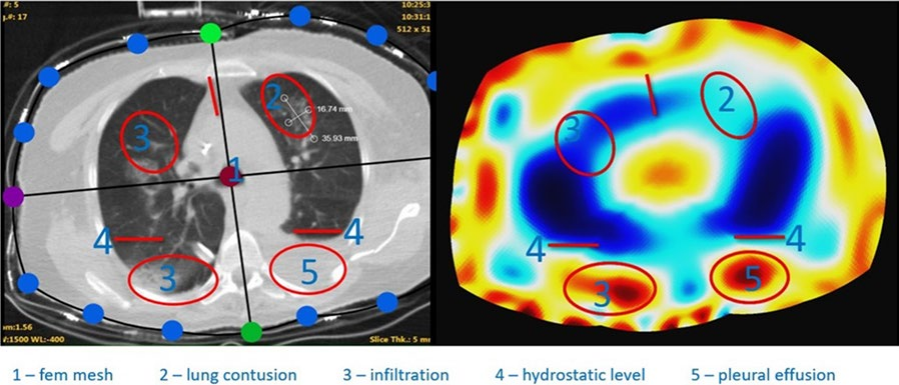
Comparison between CT scan and the resultant EIT reconstructed image.

## P141 Differential effects of mechanical power on mortality among male and female critically ill patients

### D von Wedel^1^, S Redaelli^1^, M Fosset^1^, D Shay^2^, D Talmor^1^, ELV Costa^3^, MBP Amato^3^, B Jung^1^, EN Baedorf-Kassis^4^, MS Schaefer.^1^

#### ^1^Beth Israel Deaconess Medical Center, Harvard Medical School, Department of Anesthesia, Critical Care and Pain Medicine, Boston, USA, ^2^Harvard T.H. Chan School, Department of Epidemiology, Boston, USA, ^3^University of Sao Paolo, Cardiopulmonary Department, Sao Paolo, Brazil, ^4^Beth Israel Deaconess Medical Center, Harvard Medical School, Department of Pulmonary and Critical Care Medicine, Boston, USA

*Critical Care* 2024, **28(Suppl 1):** P141

**Introduction:** Injurious effects of energy delivered to the lung during mechanical ventilation, estimated by mechanical power (MP), may depend on lung size, with potential differential effects between male and female patients. We hypothesized that 1) effects of non-normalized MP on 28-day mortality are modified by patient sex, affecting females more markedly due to smaller lung sizes [1], and 2) differences are mitigated by normalization of MP to respiratory system compliance (C_rs_) as proxy for functional lung size [2].

**Methods:** 19,806 (37.9% female) mechanically ventilated patients from the ICUs of Beth Israel Deaconess Medical Center in Boston, USA were included. Median respiratory settings were calculated for the first 24 h of mechanical ventilation and analyses adjusted for a comprehensive confounder model.

**Results:** Median (IQR) non-normalized MP was higher in males than females (11.8 [9.4–15.9] versus 10.4 J/min [8.1–13.7], *p* < 0.001). Patient sex modified the association between non-normalized MP and mortality (p-for-interaction = 0.018), resulting in a 52.7% larger effect size in females compared to males (absolute + 5.5 [95%CI 4.7–6.2] versus + 3.6% [3.2–4.1] mortality risk increase per 5 J/min). At a threshold of 17 J/min [3], mortality risk was an absolute 8.4% higher for females (Figure). Normalization of MP to C_rs_ (MP/C_rs_) mitigated differential effects (p-for-interaction = 0.23; Figure). In females, normalized MP was 11.1% higher than males and partially (23.8%) mediated increased mortality risk.

**Conclusions:** Changes in non-normalized MP result in a larger effect size in females than males and risks at non-normalized thresholds show relevant differences between both sexes. Normalization of MP to C_rs_ mitigates differences. Higher normalized MP delivered to females mediated increased mortality risk, supporting the use of ventilation strategies individualized to patients’ C_rs_ as a proxy of functional lung size.

**References**

1. Brower RG et al. N Engl J Med. 2000;342:1301–8
2. Gattinoni L et al. Intensive Care Med. 2016;42:1567–75
3. Serpa Neto A et al. Intensive Care Med. 2018;44:1914–22

**Figure (abstract P141):**
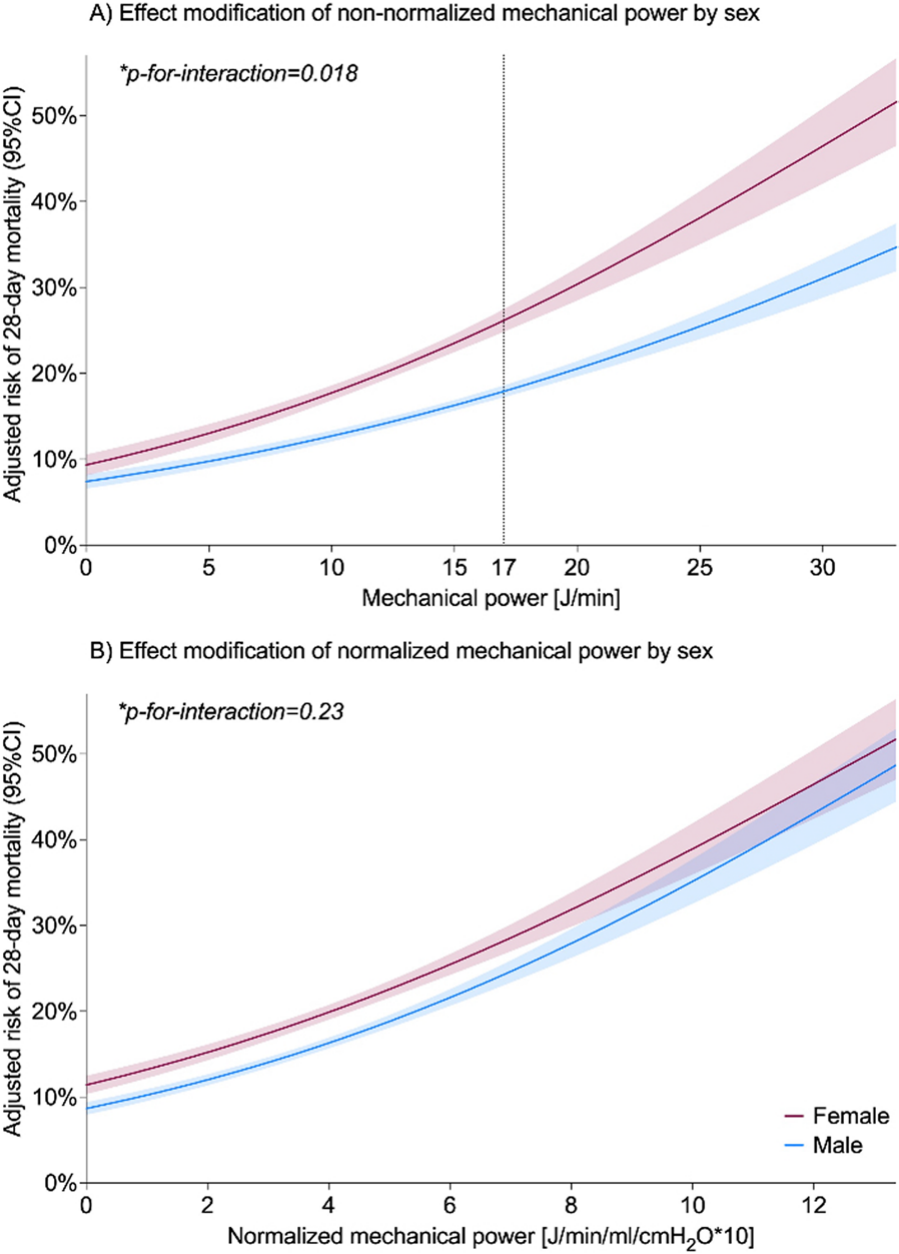
Association of mechanical power with 28-day mortality in male and female patients. The association of non-normalized mechanical power (MP) with mortality was modified by patient sex, resulting in a 54.7% larger effect size per unit change and an absolute 8.4% higher mortality risk at a threshold of 17 J/min [3] among females compared to males (Panel A). Normalization of mechanical power to respiratory system compliance mitigated these differential effects (Panel B).

## P142 Exploring non-invasive estimation of respiratory compliance during pressure support using respiratory oscillometry: a feasibility study

### A Keymolen^1^, MC Van Malderen^2^, A Marchal^1^, B van den Elshout^3^, G Vandersteen^1^, J Lataire^1^, J Jonckheer.^2^

#### ^1^Vrije Universiteit Brussel, Department of Fundamental Electricity and Instrumentation, Elsene, Belgium, ^2^Universitair Ziekenhuis Brussel, Department Intensive Care, Jette, Belgium, ^3^Demcon, Macawi Respiratory Systems, Best, Netherlands

*Critical Care* 2024, **28(Suppl 1):** P142

**Introduction:** Respiratory dynamics are used daily to assess and follow disease evolution in intubated ventilated ICU patients. Dynamic respiratory compliance (*C*_DYN_) estimation is typically performed only during controlled ventilation. During assisted ventilation, invasive methods are necessary to have adequate estimates. However, respiratory oscillometry (RO) superimposes a specific designed, low-amplitude waveform on top of the ventilation and measures the resulting flow, which is used to derive the compliance [1]. This study explores the feasibility of using RO for non-invasive respiratory compliance estimation during assisted ventilation. The results are an interim analysis of the first study patient of the McInvent trial.

**Methods:** Intubated, stable patients are included for this explorative feasibility test. Low-frequency RO is applied three times in a row, using a novel setup [1], and compared with the results of the standard of care *C*_DYN_ estimator [2], using a two-arm crossover design. The measurements are performed twice a day when the patient is on volume control ventilation (VC) and can be continued when ventilation is switched to pressure support (PS).

**Results:** The two measurement sessions were performed on PS (n = 6) and on VC (n = 6). The mean compliance estimated by RO (*C*_RO_) during VC was 42.6 mL.hPa^−1^ (95% CI 38.7–46.4) and statistically equivalent to *C*_DYN_. During PS, the mean *C*_RO_ and *C*_DYN_ were 32.6 mL.hPa^−1^ (95% CI 28.6–36.6) and 810.7 mL.hPa^−1^ (95% CI -1751.2–3372.5), respectively (Figure).

**Conclusions:** Low-frequency RO was feasible and well-tolerated by the patient during PS. The *C*_RO_ during PS was closer to the compliance measured during VC than the comparator, *C*_DYN_ during PS, which produced unreliable results as expected. However, a difference in *C*_RO_ between the two ventilation modes can be observed, disease evolution is a plausible reason. To validate and generalize the results, more patient data is required.

**References**

1. Keymolen A et al. MeMeA IEEE 2023;1–6
2. Bates JH. Cambridge University Press, 2009

**Figure (abstract P142):**
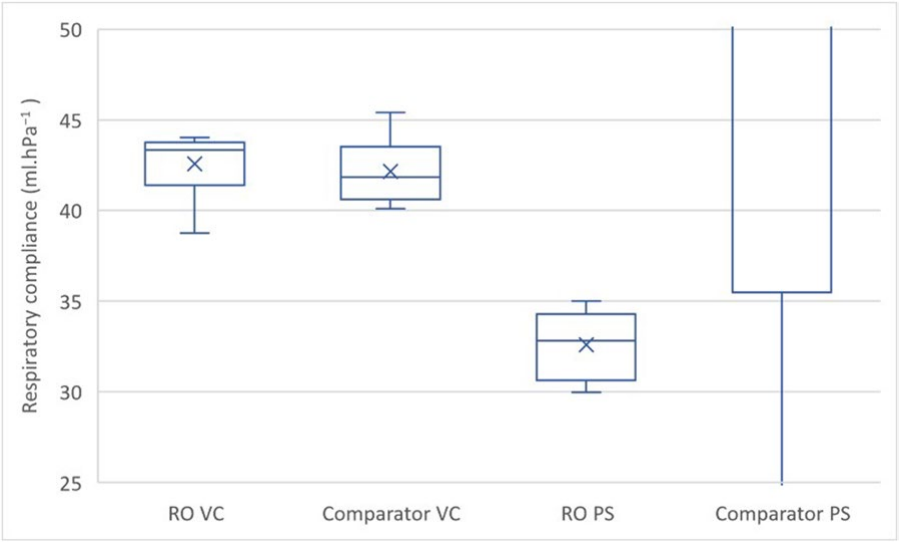
Comparison of respiratory compliance estimation methods: standard (comparator) vs. oscillometry (RO) during volume-controlled (VC) and pressure-support (PS) ventilation. During VC, both estimators agree, exhibiting minimal variability between replicates. In PS, only the RO estimation aligns closely with the VC estimations, maintaining low variability between replicates. However, a notable decrease in the mean value is observed for RO when transitioning from VC to PS.

## P143 Improving the estimation of respiratory mechanics parameters by using an extended equation of motion

### A van Diepen^1^, THGF Bakkes^1^, AJR De Bie Dekker^2^, RA Bouwman^2^, PH Woerlee^3^, S Turco^3^, M Mischi.^3^

#### ^1^Technische Universiteit Eindhoven, Electrical Engineering, Eindhoven, Netherlands, ^2^Catharina Ziekenhuis, Eindhoven, Netherlands, ^3^Technische Universiteit Eindhoven, Eindhoven, Netherlands

*Critical Care* 2024, **28(Suppl 1):** P143

**Introduction:** Respiratory mechanics estimation during mandatory ventilation is based on a first-order linear equation of motion. This equation is oversimplified, does not incorporate turbulent airflow, lung heterogeneity, or viscoelasticity, and omits hysteresis. Hysteresis was recently associated with lung injury risk [1]. We study an extended equation of motion that incorporates flow-dependent resistances and viscoelasticity of the respiratory system, which is related to hysteresis.

**Methods:** The parameters of the extended equation are estimated automatically using sequential quadratic programming. The technique is evaluated in an in-silico one-lung and two-lung model, and data from 10 post-operative patients undergoing volume-controlled ventilation is retrospectively analyzed.

**Results:** The original parameters of the one-lung model could very accurately be reproduced (close to 0% error). The error in the two-lung model was larger; 1–3%. The error of the estimates of the viscoelasticity increased when the heterogeneity in the two-lung model increased due to Pendelluft. The estimates of the post-operative patients are in a feasible range and viscoelastic parameters corresponded to parameters found with manual inspiratory occlusion maneuvers [2]. The estimated nonlinear (turbulent) term of the resistance was relatively high (14–40 cmH_2_Os^2^/L^2^) because the resistances of the endotracheal tube, capnography sensor, and bacterial filter were included.

**Conclusions:** The extended equation of motion technique allows for automatic assessment of the viscoelasticity for arbitrary flow profiles and estimation of a nonlinear resistance term. The estimates of the viscoelasticity can subsequently be used to assess and reduce hysteresis in a mandatory ventilated patient, potentially improving patient outcomes by optimizing the therapeutic intervention strategy.

**References**

1. Marini JJ et al. Crit Care Med 2020;48:1542–43
2. Antonaglia V et al. Eur Respir J. 2000;16:302–8

## P144 Greater brain activity and connectivity induced by phrenic nerve stimulation in moderate ARDS patients

### T Bassi^1^, E Rohrs^2^, M Parfait^3^, S Reynolds^2^, J Mayaux^3^, M Decavele^3^, T Similowski^3^, A Demoule^3^, M Dres^3^

#### ^1^Lungpacer Medical Inc., Critical Care, Burnaby, Canada, ^2^Simon Fraser University, Burnaby, Canada, ^3^Sorbonne Université, INSERM, UMRS1158 Neurophysiologie Respiratoire Expérimentale et Clinique, Paris, France

*Critical Care* 2024, **28(Suppl 1):** P144

**Introduction:** Sedation is used to improve ventilator-patient synchrony, therefore abolishing diaphragm activity. In healthy subjects, diaphragmatic breathing exercises increase gamma frequency power and brain connectivity. This study investigated whether phrenic nerve stimulation in synchrony with mechanical ventilation (MV) would result in changes in brain activity and connectivity within the gamma frequency band in critically ill deeply sedated patients undergoing MV.

**Methods:** A central-line catheter embedded with electrodes was inserted via the left subclavian vein in five patients to stimulate the phrenic nerves bilaterally, in synchrony with MV. The study protocol was comprised of 60-min unpaced sessions interspersed with two 60-min paced sessions. All ventilator settings and administered medications remained unchanged during the study. An electroencephalogram (EEG) was used to collect brain activity. The power spectrum density (PSD) for the gamma frequency was calculated as mV^2^/Hz. EEG Brain connectivity analysis was calculated for the gamma frequency band by constructing a Pearson correlation matrix using the EEG MATLAB toolbox. For the PSD, the Wilcoxon paired test was used for linear analysis between the sessions. *P*-values of the difference in brain connectivity between the paced and unpaced sessions were reported in a heatmap. *P* values < 0.05 were considered statistically significant.

**Results:** A significantly greater gamma PSD was observed bilaterally in the medium prefrontal cortex, insula, posterior cingulate gyrus, and angular gyrus during paced sessions compared to unpaced sessions (red symbol: a patient who had an out-of-the-hospital cardiac arrest). Compared to unpaced sessions, greater brain connectivity was observed during paced sessions (Figure).

**Conclusions:** Phrenic nerve stimulation was associated with greater brain activity and connectivity in gamma frequencies compared to unpaced sessions in deeply sedated MV moderate ARDS patients.

**Figure (abstract P144):**
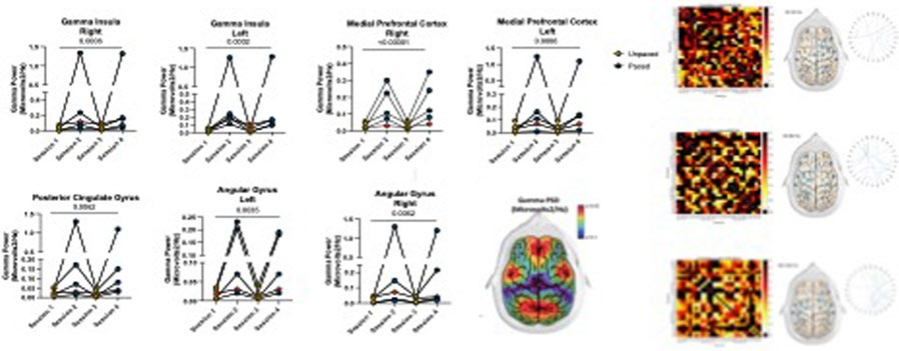
On the left hand-side is observed gamma frequency per patients per session. On the right hand side is observed the heat maps for brain connectivity.

## P145 Association between driving pressure and mortality in very old patients with acute respiratory distress syndrome

### E Papoutsi, K Gkirgkiris, S Gkoufas, I Dimopoulou, A Kotanidou, II Siempos

#### National and Kapodistrian University of Athens Medical School Athens, First Department of Critical Care Medicine and Pulmonary Services, Athens, Greece

*Critical Care* 2024, **28(Suppl 1):** P145

**Introduction:** We evaluated the association between driving pressure and mortality across different age groups of patients with acute respiratory distress syndrome (ARDS) with a special focus on very old patients (≥ 80 years old).

**Methods:** We performed a secondary analysis of individual patient-level data from seven ARDS Network and PETAL Network randomized controlled trials [1–7]. We considered three age groups; namely, patients < 65 years old, 65–79 years old and ≥ 80 years old.

**Results:** Of 4567 patients with ARDS, 201 were ≥ 80 years old, 917 were 65–79 years old and 3449 were < 65 years old. In unadjusted logistic regression analyses, the odds ratio (OR) for the association between driving pressure and mortality was 1.081 [95% confidence interval (CI) 1.027–1.138)] for ≥ 80 years old, 1.054 (95% CI 1.031–1.077) for 65–79 years old and 1.016 (95% CI 1.005–1.028) for < 65 years old. Consistently, in adjusted analyses, the corresponding OR was 1.112 for ≥ 80 years old, 1.055 for 65–79 years old and 1.024 for < 65 years old. The effect of driving pressure on mortality was higher for patients ≥ 80 years old compared to patients 65–79 years old or patients < 65 years old (*p* = 0.009 for the interaction between age and driving pressure; Figure).

**Conclusions:** Very old patients with ARDS may be more vulnerable to high driving pressure than their younger counterparts. These results may advocate for a personalized age-dependent mechanical ventilation approach.

**References**

- Brower RG et al. N Engl J Med. 2000;342:1301–8.
- Brower RG et al. N Engl J Med. 2004;351:327–36.
- Wiedemann HP et al. N Engl J Med. 2006;354:2564–75.
- Matthay MA et al. Am J Respir Crit Care Med. 2011;184:561–8.
- Rice TW et al. JAMA. 2012;307:795–803.
- Truwit JD et al. N Engl J Med.2014;370:2191–200.
- National Heart, Lung, and Blood Institute PETAL Clinical Trials Network. N Engl J Med. 2019;380:1997–2008.

**Figure (abstract P145):**
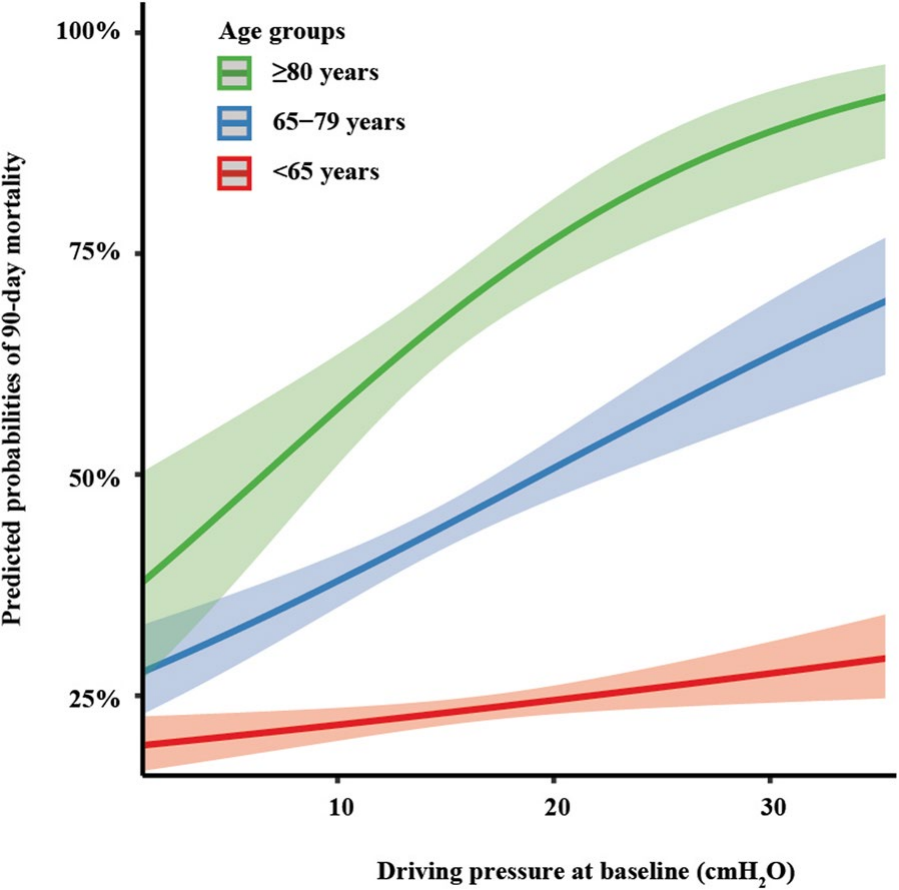
Interaction plot showing that the effect of driving pressure on mortality was influenced by age.

## P146 Identification of ARDS subphenotypes through artificial intelligence: approaching personalized medicine

### G Meza-Fuentes, MA Retamal, M Barbé, I Sánchez-Barraza, I Delgado, R López

#### Universidad del Desarrollo, Instituto de Ciencias e Innovación en Medicina, Santiago, Chile

*Critical Care* 2024, **28(Suppl 1):** P146

**Introduction:** Acute respiratory distress syndrome (ARDS), which accounts for 10.4% of global ICU admissions with a mortality rate of 45%, is a complex disease characterized by noncardiogenic pulmonary edema [1]. Its complexity, derived from various risk factors, poses challenges in personalized diagnosis and treatment, due to its clinical heterogeneity. The current re-evaluation of ARDS criteria [2] intensifies the heterogeneity of the disease, emphasizing the need to identify subphenotypes for personalized treatment and correct patient selection.

**Methods:** We conducted a cross-sectional study at Clínica Alemana de Santiago ICU, approved by the Institutional Ethics Committee. Machine learning categorized ARDS subphenotypes. We used Gaussian Mixture Model (GMM) for initial unsupervised clustering, followed by supervised models for root cause analysis, patient classification, and performance measurement.

**Results:** Our models identified two ARDS subphenotypes with significant clinical differences and outcomes. Subphenotype 1 was characterized by lower mortality, less clinical severity, and a less restrictive pattern with improved gas exchange compared to Subphenotype 2, which exhibited the opposite (Table). The models demonstrated high performance with an area under the ROC curve of 0.94, sensitivity of 94.23% and specificity of 87.5%. The most influential variables in subphenotype discrimination were distension pressure, respiratory rate, EtCO_2_, and normalized tidal volume.

**Conclusions:** This study presents a promising approach to enhance ARDS subphenotyping. Utilizing machine learning models for clustering and prediction involving clinical, ventilatory mechanics, and gas exchange variables allowed for more precise patient stratification. These findings have the potential to optimize individualized treatment selection and improve clinical outcomes in ARDS patients.

**References**

1. Bellani G et al. JAMA. 2016;315:788
2. Matthay MA et al. Am J Respir Crit Care Med. 2024;209:37–47

**Table (abstract P146):** Characteristics of ARDS subphenotypes on the first day of ventilation for included patients

Variable	Subphenotype 1 (n = 172)	Subphenotype 2 (n = 52)	*p* value
Mechanical power (J/min)	17.21 (14.11–19.84)	21.61 (17.58–29.09)	< 0.001*
Tidal volume (mL/kg/PBW)	4.37 (4.02–4.82)	3.84 (2.96–4.59)	< 0.001*
Driving pressure (cmH_2_O)	10 (8–11)	11 (10–12)	< 0.001*
Total respiratory rate (rpm)	24 (20–26)	30 (26–36)	< 0.001*
VD/VT	0.5 (0.43–0.57)	0.62 (0.54–0.73)	< 0.001*
EtCO_2_ (mmHg)	30.5 (33–35.5)	36 (34–41)	< 0.001*
28-Day Mortality	16 (9.3%)	11 (21.15%)	0.021*

Statistical significance was assessed using the Mann-Whitney test, with a significance level set at *p* <0.05. Values are expressed as median (interquartile range). Qualitative variables are presented as absolute frequency (relative frequency). VD/VT (dead space volume/tidal volume), EtCO_2_ (end-tidal CO_2_)

## P147 Exploration of COVID-19 associated bradycardia using heart rate variability analysis in a case–control study of ARDS patients

### H Dumargne^1^, H Patural^2^, F Charbonnieras^3^, D Charier^4^, C Biscarrat^5^, M Chivot^5^, L Argaud^5^, M Cour^5^, A Dargent^1^

#### ^1^Hospices Civils de Lyon, Hôpital Lyon Sud, Service d’Anesthésie-Réanimation Médecine Intensive-Réanimation, Pierre Bénite, France, ^2^Centre Hospitalier Universitaire de Saint-Etienne, Service de Réanimation Pédiatrique, Saint-Etienne, France, ^3^Hospices Civils de Lyon, Hôpital de la Croix Rousse, Unité de Soins Intensifs Cardiologiques, Lyon, France, ^4^Centre Hospitalier Universitaire de Saint-Etienne, Service d’Anesthésie-Réanimation, Saint-Etienne, France, ^5^Hospices Civils de Lyon, Hôpital Edouard Herriot, Service de Médecine Intensive-Réanimation, Lyon, France

*Critical Care* 2024, **28(Suppl 1):** P147

**Introduction:** The bradycardia and dysautonomia observed during SARS-Cov2 infection have raised questions about the involvement of the autonomic nervous system (ANS). Data on ANS dysregulation and its association with outcomes are limited. We aimed to investigate sympathovagal balance, assessed by heart rate variability (HRV), and its clinical prognostic value in patients with acute respiratory distress syndrome (ARDS) related to COVID-19 (C-ARDS) or to other aetiologies (NC-ARDS).

**Methods:** A single-centre prospective case–control study was conducted. Consecutive patients fulfilling ARDS criteria between 2020 and 2022 were included. HRV was assessed offline from 1-h electrographic tracings using time and frequency measures and compared between C-ARDS and NC-ARDS.

**Results:** During the study period, 24 C-ARDS and 19 NC-ARDS were included. Median heart rate was significantly lower in C-ARDS than in NC-ARDS (60 [53–72] versus 101 [91–112] bpm, *p* < 0.001). HRV was significantly impaired in both C-ARDS and NC-ARDS patients (Table). Almost all time and frequency parameters of HRV were significantly less impaired in C-ARDS patients than in NC-ARDS patients. HRV correlated with heart rate only in C-ARDS patients. No significant differences in HRV parameters were found when comparing survivors and non-survivors. A positive correlation was found between low to high frequency ratio (LF/HF) and ICU length of stay (r = 0.576, *p* < 0.001). Other HRV variables were not correlated with clinical outcomes.

**Conclusions:** This prospective study confirms that C-ARDS is associated with marked bradycardia and severe ANS impairment. HRV was less severely impaired and correlated with heart rate only in COVID-19 patients, suggesting a sympathovagal imbalance with vagal overtone. Our study did not find a robust predictive value of HRV analysis, but poor outcomes seemed more related to sympathetic than parasympathetic hyperactivation.

**Table (abstract P147):** Heart rate variablility parameters

Heart Rate Variability	All ARDS (n = 43)	C-ARDS (n = 24)	NC-ARDS (n = 19)	*p* value
Heart rate (bpm)	77 [57–100]	60 [53–72]	101 [91–112]	< 0.001
RR interval (ms)	778 [600–1046]	1004 [836–1142]	595 [538–661]	< 0.001
SDRR (ms)	15 [9–36]	25 [14–46]	10 [7–18]	0.002
VLF (ms)	60 [17–164]	108 [67–257]	19 [3.9–42]	< 0.001
LF (ms)	10 [3.0–29]	15 [10–69]	3.0 [0.71–9.0]	< 0.001
HF (ms)	22 [7.7–64]	31 [18–128]	9.1 [4.0–27]	< 0.001
LF/HF ratio	0.36 [0.13-0.71]	0.45 [0.32-0.87]	0.18 [0.09-0.44]	0.018

Data are presented as medians and interquartile range. ARDS: acute respiratory distress syndrome; C-ARDS: Covid-19 related ARDS; NC-ARDS: non-Covid-19 related ARDS; SDRR: standard deviation of RR intervals; VLF: very low-frequency; LF: low-frequency; HF: high-frequency; bpm: beats per minute.

## P148 Biomarkers of alveolocapillary membrane injury in COVID-19 related ARDS: a pilot study

### A Andrijevic, U Batranovic, V Carapic, S Gavrilovic, J Matijasevic, S Milic, D Nedeljkov

#### Institute for Pulmonary Diseases of Vojvodina, Intensive Care Unit, Sremska Kamenica, Serbia

*Critical Care* 2024, **28(Suppl 1):** P148

**Introduction:** We aimed to investigate serum biomarkers of alveolocapillary membrane injury (sRAGE, endocan and angiopoetin-2) in COVID-19 related ARDS as prognostic markers of 28 day mortality.

**Methods:** An observational, retrospective pilot study was conducted on mechanically ventilated patients, admitted to Institute for pulmonary diseases of Vojvodina from December 2020 until June 2021. All patients fulfilled Berlin criteria for ARDS while SARS CoV-2 infection was proven positive by RT-PCR of nasopharyngeal swab. Within 48 h from starting mechanical ventilation the blood samples were taken for measurement of serum biomarkers (sRAGE, endocan, angiopoetin-2). We collected baseline demographic data, Charlson comorbidity index, APACHE II, SOFA and SAPS2 score, procalcitonin and CRP values, neutrophil/lymphocyte ratio, PaO_2_/FiO_2_ ratio, PEEP and driving pressure values, presence of septic shock at admission and need for VV-ECMO support. The primary endpoint was 28-day mortality.

**Results:** A total of 36 patients were included in the study (Table). The average age was 65 (IQR 16), the observed group consisted of 11 (30.6%) females. Arterial hypertension and obesity were the most common comorbidities. Median Charlson score was 2.5 ± 2, median APACHE II score 20 (IQR 9), median SOFA score 5 (IQR 3) and median SAPS2 score 3 (IQR 16). Overall mortality was 63.9%. Septic shock was present in 25 (69.4%) patients and 3 patients needed VV-ECMO support. There were no differences observed in the level of measured biomarkers with respect to 28 days mortality for endocan and angiopoetin-2 (1.65 ng/mL, IQR 2.7 ng/mL vs .2.53, IQR 3.47 ng/mL, *p* = 0.21 and 1.15 ng/mL, IQR 3.39 ng/mL vs. 2.15, IQR 3.17 g/mL, *p* = 0.24) respectively. Level of sRAGE was lower in COVID survivors but difference did not reach statistical significance (5263 pg/mL, IQR 18,601 pg/mL vs. 18,750, IQR 23,744 pg/mL, *p* = 0.1).

**Conclusions:** Serum value of sRAGE may serve as promising biomarker for COVID-19 ARDS prognostication and clinical trial enrichment, but further studies are needed.

**Table (abstract P148):** Laboratory findings and parameters of mechanical ventilation

	COVID-19 survivors at day 28, n = 13	COVID-19 non-survivors at day 28, n = 23
C-reactive protein (mg/mL) median, IQR	157 (141.1)	113.5 (108.5)
Procalcitonin (ng/mL) median, IQR	0.22 (0.7)	0.35 (1.18)
Neutrophil/lymphocyte ratio median, IQR	13 (18.6)	15.8 (17.1)
PaO_2_/FiO_2_ median, IQR	77 (104)	83 (38)
PEEP median, IQR	10 (4)	12 (2)
Driving pressure median, IQR	13 (6)	12 (5)

## P149 Compliance of the respiratory system in patients with COVID-19 and non-COVID-19 ARDS

### F Righetti^1^, E Colombaroli^2^

#### ^1^Intensive Care Unit, Emergency Department, San Bonifacio, Verona, Italy, ^2^Intensive Care Unit, San Bonifacio, Verona, Italy

*Critical Care* 2024, **28(Suppl 1):** P149

**Introduction:** The comparison of respiratory system compliance (Crs) between COVID19 (Coronavirus disease 2019) non-COVID19 ARDS (acute respiratory distress syndrome) patients has been the object of debate, but few studies have evaluated it when considering applied positive end expiratory pressure (PEEP), which is one of the known determinants of Crs itself. The aim of this study was to compare Crs taking into account the applied PEEP [1].

**Methods:** Two cohorts of patients were created: those with COVID-ARDS and those with non-COVID ARDS. In the whole sample the association between Crs and type of ARDS at different PEEP levels was adjusted for anthropometric and clinical variables. As secondary analyses, patients were matched for predicted functional residual capacity and the same association was assessed. Moreover, the association between Crs and type of ARDS was reassessed at predefined PEEP level of 0, 5, 8, 12, and 15 cmH_2_O with a propensity score-weighted linear model.

**Results:** 263 patients were included in the study, 159 patients with COVID-ARDS and 104 with non-COVID ARDS. The association between Crs and type of ARDS was not significant in both the complete cohorts (*p* = 0.11) and in the matched cohorts (*p* = 0.96). This was true also for the propensity score weighted association at PEEP 5, 8, 12 and 15 cmH_2_O, while it was statistically significant at PEEP 0 (with a median difference of 2 mL/cmH_2_O, which is not clinically significant).

**Conclusions:** The compliance of the respiratory system is similar between COVID ARDS and non-COVID ARDS when calculated at the same PEEP level and while taking into account patients’ anthropometric characteristics.

**Reference**

1. Grasselli G et al. Lancet Respir Med. 2020;8:1201–8

## P150 Analysis of ventilation-perfusion mismatch by electrical impedance tomography and the severity of experimental lung injury

### I Marongiu^1^, E Spinelli^1^, A Damia^2^, F Damarco^3^, L Rosso^2^, M Battistin^4^, S Oldoni^4^, G Lopez^5^, V Vaira^2^, T Mauri^2^

#### ^1^Fondazione IRCCS Ca´ Granda Ospedale Maggiore Policlinico, Department of Anesthesia, Critical Care and Emergency, Milan, Italy, ^2^University of Milan, Department of Pathophysiology and Transplantation, Milan, Italy, ^3^Fondazione IRCCS Ca´ Granda Ospedale Maggiore Policlinico, Division of Thoracic Surgery and Lung Transplantation, Milan, Italy, ^4^Fondazione IRCCS Ca´ Granda Ospedale Maggiore Policlinico, Center for Preclinical Research, Milan, Italy, ^5^University of Milan, Division of Pathology, Milan, Italy

*Critical Care* 2024, **28(Suppl 1):** P150

**Introduction:** The exclusion of one lung from ventilation or perfusion alters the distribution of tidal volume and pulmonary blood flow with impairment of V/Q matching and development of pathological mechanisms inducing lung injury. The measure of V/Q mismatch by Electrical Impedance Tomography (EIT) is regional and could be more accurate than classic global measures of intrapulmonary shunt and physiological dead space (DS).

**Methods:** Sedated and paralyzed pigs were mechanically ventilated (Vt 15 mL/Kg, PEEP 1 cmH_2_O, RR 15, FiO_2_ 0.5) for 24 h: 11 underwent ligation (non-perfused lung injury, NPLI) and 10 exclusion (non-ventilated lung injury, NVLI) of the left lung (LL). Physiological and EIT data were collected along the experiment, and histological score (HS) after sacrifice.

**Results:** In the LL, EIT data showed that nearly all the ventilation was distributed to the DS and high V/Q compartments (Figure panels A and B) in the NPLI group, while the LL of the NVLI group was characterized by 100% of ventilation reaching the shunt compartment (Figure panel C). In the right lung (RL) of the NVLI group, most of ventilation reached the DS and high V/Q compartments (Figure panels D and E), and around 80% of perfusion reached high V/Q units (Figure panel F). In the RL of the NPLI group, instead, large fraction of ventilation and perfusion reached the normal V/Q compartment (Figure panels G and H). Physiological DS differed only after 2 h from start and then was similar between the two groups (Figure panel I). The calculated shunt fraction was low (less than 20%) in both groups and only slightly higher in the NVLI group (Figure panel J). Higher degree of V/Q mismatch in both lungs of the NVLI group was associated with more severe HS compared to the NPLI group (LL HS: NVLI 10.3 ± 2.0 vs. NPLI 6.4 ± 1.6, *p* < 0.0001; RL HS: NVLI 5.5 ± 2.0 vs. NPLI 3.0 ± 0.7, *p* < 0.001).

**Conclusions:** Analysis of V/Q mismatch by EIT is more accurate than classic global measures and could be directly correlated with the severity of lung injury.

**Figure (abstract P150):**
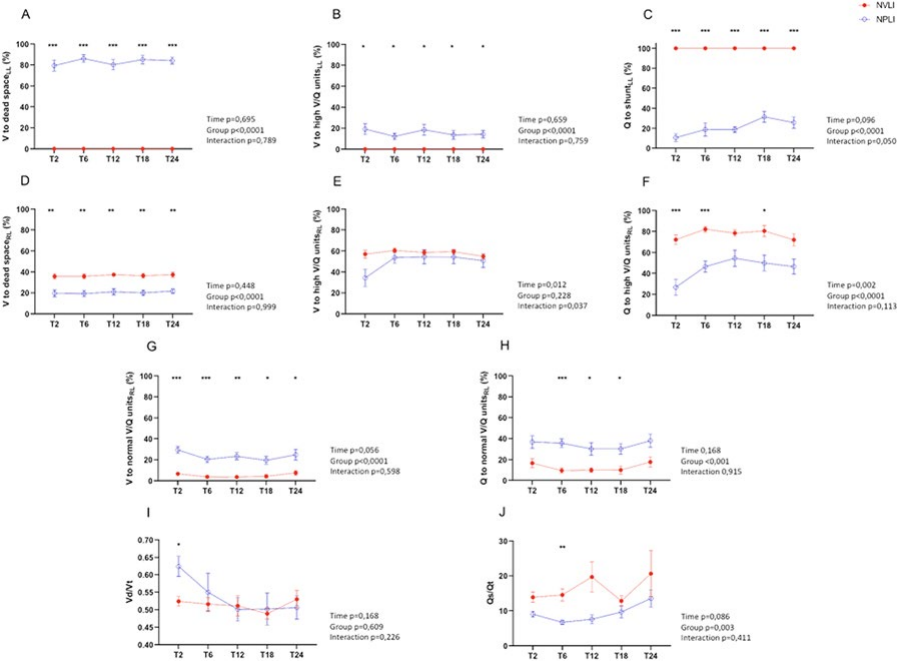
Analysis of regional and global V/Q mismatch. Data are expressed as mean ± SEM. Comparisons are obtained with mixed-effect analysis for repeated measures followed by Tukey’s post-hoc test with groups and time as independent factors and group × time interaction. *P* values are reported in the graphs.

## P151 Volume of recruitment as a predictor of lung stress and mortality in acute respiratory distress syndrome patients: reanalysis of a multicenter international randomized clinical trial

### A Santarisi^1^, A Suleiman^1^, S Redaelli^1^, D Talmor^1^, V Goodspeed^1^, S Harrison^1^, JR Beitler^2^, E Baedorf Kassis^3^

#### ^1^Beth Israel Deaconess Medical Center, Department of Anesthesia, Critical Care and Pain Medicine, Boston, USA, ^2^Columbia University College of Physicians & Surgeons, Center for Acute Respiratory Failure and Division of Pulmonary, Allergy, and Critical Care Medicine, New York, USA, ^3^Beth Israel Deaconess Medical Center, Department of Pulmonary, Critical Care & Sleep Medicine, Boston, USA

*Critical Care* 2024, **28(Suppl 1):** P151

**Introduction:** ARDS decreases aerated lung volume, causing variable lung stress and risk of Ventilator-Induced Lung Injury, despite low tidal volume ventilation. Lung stress assessment is limited to patients with esophageal manometer measurements. Recruitment maneuvers (RM) aim to expand functional lung volume, termed V_RM_. A prior study in 42 patients correlated low V_RM_ with increased lung stress and mortality [1]. Our study aims to validate these findings in a larger cohort.

**Methods:** We performed a post-hoc analysis on EPVent2 randomized controlled trial [2]. We first evaluated the association between V_RM_ and predicted-inspiratory capacity (IC), as predicted using Reference equations [3]. Using linear regression models, we then assessed the association between V_RM_/predicted body weight (PBW) and lung and tidal stress. Lung stress was defined as end-inspiratory transpulmonary pressure and tidal stress as the difference between end-inspiratory and end-expiratory transpulmonary pressure. Logistic regression was used to estimate the association between V_RM_ and 28-day mortality.

**Results:** We included 119 patients. V_RM_ was significantly lower than predicted-IC (mean difference 839.34 ± 368.91 mL; *p* < 0.001) and not correlated with predicted-IC (R^2^ = 0.12; *p* = 0.22). V_RM_/PBW ranged between 4.68 and 31.12 mL/kg. Lower V_RM_/PBW was associated with higher lung stress (ß = 0.33 cmH_2_O per 1 mL/kg of V_RM_/PBW decrease, 95% CI 0.46–0.20; *p* < 0.001) and tidal stress (ß = 0.18 cmH_2_O per 1 mL/kg of V_RM_/PBW, 95% CI 0.29–0.08; *p* = 0.001) (Figure). In analyses adjusted for driving pressure, PaO_2_/FiO_2_, PEEP, plateau pressure, and tidal volume/PBW, V_RM_/PBW remained independently associated with lung but not tidal stress. V_RM_ did not predict 28-day mortality.

**Conclusions:** In ARDS patients, low V_RM_ may predict high lung stress but not mortality.

**References**

1. Beitler JR et al. Crit Care Med. 2016;44:91–9
2. Beitler JR et al. JAMA. 2019;321:846–857
3. Stocks J et al. Eur Respir J. 1995;8:492–506

**Figure (abstract P151):**
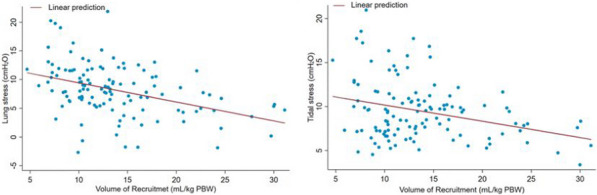
Global lung stress prediction. Left: end-inspiratory lung stress, calculated as transpulmonary pressure during the end-inspiratory pause of a tidal breath. Right: tidal lung stress, calculated as transpulmonary pressure difference between end-inspiratory and end-expiratory pauses during a tidal breath. The Figure highlights unadjusted associations between volume of recruitment/predicted body weight and both lung and tidal stress.

## P152 Association between lung mechanics and the development of acute kidney injury in ARDS patients

### I Andrianopoulos^1^, P Kremmydas^2^, E Papoutsi^2^, I Siempos^2^, S Kokkoris^2^

#### ^1^University Hospital of Ioannina, Intensive Care Unit, Ioannina, Greece, ^2^Evangelismos Hospital, National and Kapodistrian University of Athens Medical School, First Department of Critical Care Medicine and Pulmonary Services, Athens, Greece

*Critical Care* 2024, **28(Suppl 1):** P152

**Introduction:** Mechanical ventilation and acute respiratory distress syndrome (ARDS) are considered as risk factors for acute kidney injury (AKI). Despite the availability of experimental data, few clinical data exist on the effect of lung mechanics and respiratory parameters on the development of AKI. We pursued to investigate the effect of mechanical ventilation on the development of AKI in mechanically ventilated patients with ARDS.

**Methods:** We performed a secondary analysis of individual patient data from seven therapeutic controlled trials conducted by the ARDS and PETAL networks. Chronic kidney disease and early AKI patients (patients developing AKI < 48 h of enrollment) were excluded from the study. Late AKI was defined as the development of AKI more than 48 h and up to seven days after the initiation of mechanical ventilation.

**Results:** Overall, out of 2986 patients with ARDS 726 developed late AKI (late AKI group) and 2260 did not develop AKI (no AKI group). Both driving pressure (DP) and positive end-expiratory pressure (PEEP) at enrollment were independently associated with the development of late AKI [hazard ratio, (HR) 1.039 for increments of 1 cmH_2_O in DP, 95% confidence interval (CI) 1.022–1.056 and HR 1.052 for increments of 1 cmH_2_O in PEEP, 95% CI 1.021, − 1.084, respectively]. In addition, both mean DP and mean PEEP were independently associated with the development of late AKI [HR 1.052 for increments of 1 cm H_2_O in DP, 95% CI 1.035–1.07 and HR 1.203 for increments of 1 cm of H_2_O in PEEP, 95% CI 1.165–1.242]. Age (HR 1.008 for increments of 1 year, 95% CI 1.001–1.0015), diabetes mellitus (HR 1.469, 95% CI 1.110–1.944), cardiovascular (HR 1.525, 95% CI 1.212–1.920) and hepatic dysfunction (HR 1.628, 95% CI 1.192–2.224) were also identified as independent risk factors for late AKI.

**Conclusions:** Injurious mechanical ventilation may cause late AKI.

## P153 Comparison of the effectiveness of awake-prone positioning and high-flow nasal oxygen in patients with COVID-19-related acute respiratory failure between different waves

### N Fuentes^1^, M Olmos^1^, M Busico^2^, A Gallardo^3^, A Vitali^4^, J Quintana^5^, H Kakisu^1^, B Ferreyro^6^, ME González^1^, M Esperatti^1^

#### ^1^Hospital Privado de Comunidad, Critical Care Unit, Mar del Plata, Argentina, ^2^Clínica Olivos SMG, Buenos Aires, Argentina, ^3^Sanatorio Clínica Modelo de Morón, Critical Care Unit, Buenos Aires, Argentina, ^4^Sanatorio de la Trinidad Palermo, Critical Care Unit, Buenos Aires, Argentina, ^5^Clínica Olivos SMG, Critical Care Unit, Buenos Aires, Argentina, ^6^Interdepartmental Division of Critical Care Medicine, University of Toronto, Critical Care Unit, Toronto, Canada

*Critical Care* 2024, **28(Suppl 1):** P153

**Introduction:** Awake positioning (AW-PP) has been shown to reduce the risk of endotracheal intubation (ETI) in patients with COVID-19-related acute respiratory failure (ARF) receiving non-invasive advanced respiratory support in the intensive care unit (ICU) [1, 2]. Many aspects changed during the pandemic (incidence and hospitalization rate), generating variable levels of stress in health systems [3, 4]. Vaccination coverage also changed over time [5]. Despite this, the effective treatments were not re-evaluated. Objective: to compare the effectiveness of AW-PP on relevant clinical outcomes in patients with COVID-19-related ARF between different waves in Argentina.

**Methods:** This multicenter, prospective cohort study included adult patients with COVID-19-related ARF requiring high-flow nasal oxygen. The main exposure was AW-PP (≥ 6 h/day), compared to non-prone positioning. The primary outcome was ETI and the secondary outcome was in-hospital mortality. To adjust for confounding factors (clinical-epidemiological), inverse probability weighting-propensity score and the robust approach were used.

**Results:** 728 patients were included; 360 during the first and 368 during the second wave, of whom 195(54%) and 227(62%) remained on PP for a median (p25–75) of 12(10–16) and 14(8–17) h/day, respectively (AW-PP group). The OR (95% CI) for ETI in the AW-PP group were 0.25(0.13–0.46) and 0.19(0.09–0.31), for first and second waves respectively (*p* = 0.41). The OR for in-hospital mortality were 0.35 (0.17–0.65) and 0.22 (0.12–0.43), respectively (*p* = 0.44) (Figure).

**Conclusions:** AW-PP was associated with a reduction in the risk of ETI and in-hospital mortality in both waves.

**References**

1. Ehrmann S et al. Lancet Respir Med. 2021;9:1387–1395.
2. Li J et al. Lancet Respir Med. 2022;10:573–583.
3. Greco M et al. Intensive Care Med. 2022;48:690–705.
4. Kurtz P et al. Intensive Care Med. 2021;47:538–548.
5. Watson OJ et al. Lancet Infect Dis. 2022;22:1293–1302.

**Figure (abstract P153):**
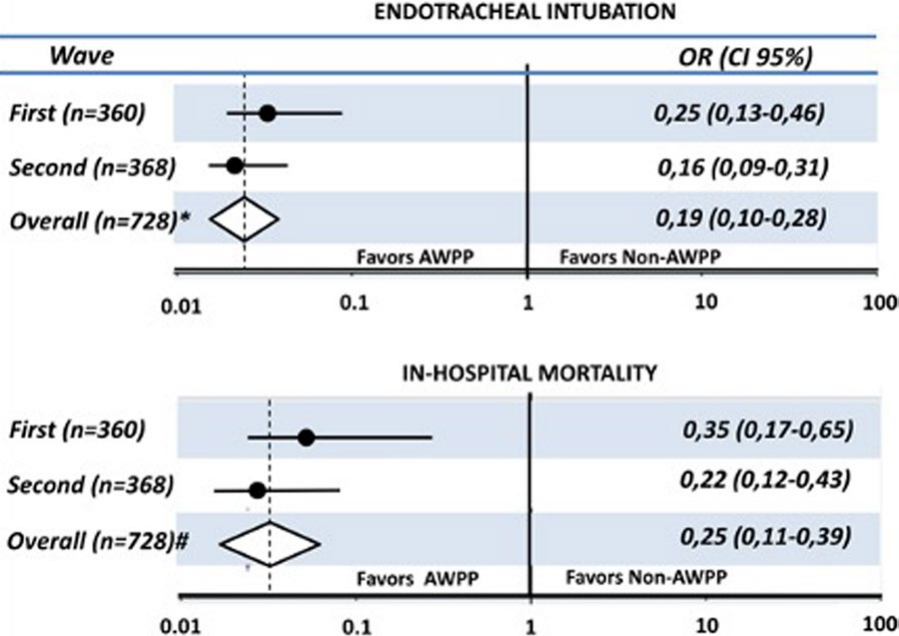
Risk of intubation and in-hospital mortality between groups in awake prone position and non-prone position during 1st and 2nd waves in Argentina.

## P154 Hierarchical clustering to identify phenotypes of prone position responsiveness in ARDS patients

### M Fosset^1^, S Redaelli^1^, D von Wedel^1^, A Suleiman^1^, R Munoz-Acuna^1^, J Josse^2^, EN Baedorf-Kassis^3^, MS Schaefer^1^, B Jung^1^

#### ^1^Beth Israel Deaconess Medical Center, Harvard Medical School, Department of Anesthesia, Critical Care and Pain Medicine, Boston, USA, ^2^Desbrest Institute of Epidemiology and Public Health, PreMeDICaL, Montpellier, France, ^3^Beth Israel Deaconess Medical Center, Harvard Medical School, Department of Pulmonary and Critical Care Medicine, Boston, USA

*Critical Care* 2024, **28(Suppl 1):** P154

**Introduction:** Prone positioning (PP) is recommended during mechanical ventilation (MV) in moderate to severe ARDS [1] to reduce mortality, but it is unclear which subgroups might benefit from it. We aimed to identify patient clusters based on respiratory system mechanics and oxygenation parameters to phenotype PP responders.

**Methods:** We retrospectively included critically ill adult patients receiving invasive MV for ≥ 24 h, and ≥ 1 cycle of PP between 2020 and 2022 at a tertiary academic hospital in Boston, USA. Using hierarchical clustering [2], we identified clusters based on baseline variables and tested their association with changes in PF ratio (PF), mechanical power (MP), driving pressure (DP), and ventilatory ratio (VR) between baseline (supine) and PP. Patients were classified as responders based on a relative change > 0% for PF, and < 0% for the other parameters. We used Pearson's Chi-squared tests with Holm Bonferroni correction for comparisons.

**Results:** 353 patients were included (60[52–69] years, 141[40%] female). Three clusters were identified at baseline. Compared to the overall population's mean, patients in Cluster 1 had significantly higher PF (165[127–220] mmHg) and lower PEEP (8[5–10] cmH20); patients in Cluster 2 had higher PEEP (13[12–15] cmH_2_O) and lower DP (11[10–13] cmH_2_O). Patients in Cluster 3 had higher VR (2.8[2.4–3.3]), lower pH (7.27[7.22–7.31]), and higher PCO_2_ (61[53–66] mmHg; Figure). Across Cluster 1, 2, and 3, there were no significant differences in proportion of responders for PF (74% vs. 82% vs. 75%), MP (39% vs. 42% vs. 42%), DP (41% vs. 40% vs. 44%), and VR (52% vs. 49% vs. 46%). Patients in Cluster 3 died more than in Clusters 1 and 2 (56% vs. 41% and 36%, *p* = 0.037).

**Conclusions:** Unsupervised classification of patients undergoing PP can identify clusters with different prognoses using baseline variables, but it does not identify phenotypes that would benefit from PP.

**References**

1. Grasselli G et al. Intensive Care Med. 2023;49:727–759
2. Husson F et al. Applied Mathematics Dep. 2010

**Figure (abstract P154):**
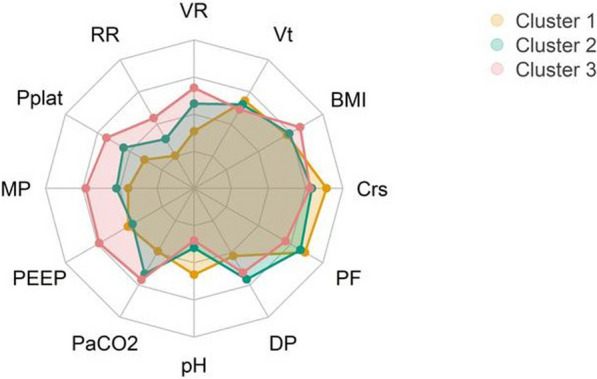
Profiles of the different phenotypes according to the most important variables to determine the clusters. Data have been normalized to have a mean of 0 and a standard deviation of 1 across all phenotypes. When multiple values were present for a given variable, it was summarized by its median. VR ventilatory ratio; Vt tidal volume; BMI body mass index; Crs respiratory system compliance, PF PaO_2_/FiO_2_ ratio; DP driving pressure; pH arterial pH; PaCO_2_ arterial pressure in CO_2_; PEEP positive end-expiratory pressure; MP mechanical power; Pplat plateau pressure; RR respiratory rate.

## P155 Duration of extracorporeal membrane oxygenation management and prognosis of patients with coronavirus disease 2019 complicated by air leak syndrome

### Y Izutani, K Muranishi, S Morimoto, J Maruyama, S Ninomiya, R Kato, R Yuge, T Kitamura, H Ishikura

#### Fukuoka University Hospital, Emergency and Critical Care Center, Fukuoka-shi, Japan

*Critical Care* 2024, **28(Suppl 1):** P155

**Introduction:** Coronavirus disease 2019 (COVID-19)-ARDS is complicated by air leak syndrome (ALS) and requires a prolong lung protection strategy including extracorporeal membrane oxygenation (ECMO). We investigated the effect of ALS on the duration of ECMO management and prognosis of disease.

**Methods:** In this single-centre, retrospective, observational study, patients in ICU who were affected by COVID-19-ARDS that was managed with ECMO, were screened for eligibility from April 2020 to December 2021. Selected patients were categorized into ALS and non-ALS groups. The ALS group was further divided into the post-ALS ECMO group (A–E group) and pre-ALS ECMO group (E–A group). The ICU survival rate, duration of ECMO management, and functional prognosis after 6 months were evaluated.

**Results:** Forty-one patients were included during the study period (ALS group, 19 patients; non-ALS group, 22 patients). The duration of ECMO management (days) was significantly longer in the ALS group [44 (29–64) vs. 9 (7–14), *p* < 0.05] than that in the non-ALS group. The ICU survival rate was 47% in the ALS group and 86% in the non-ALS group (*p* < 0.05). With respect to the functional prognosis of ICU survivors, 78% of the survivors in the ALS group and 88% of the survivors in the non-ALS group were ambulatory (*p* = 0.60). Furthermore, there were five patients in the A–E group and fourteen patients in the E–A group in the ALS group. The duration of ECMO management was similar [22 (9–43) vs. 22 (8–42), *p* = 0.89], but ICU survival rate tended to be higher in the A–E group [80% vs. 36%, *p* = 0.14]. In terms of the functional prognosis of ICU survivors, 100% of the survivors in the A–E group and 60% of the survivors in the E-A group were ambulatory (*p* = 0.44).

**Conclusions:** Patients with severe COVID-19-ARDS complicated with ALS have a lower survival rate than those without ALS. However, their long-term functional prognosis is not poor. Long-term ECMO management should be considered.

## P156 Data-driven clinical decision support system applied to intensive care medicine: study in patients with SARS-CoV-2 pneumonia treated with ECMO

### F Ribeiro^1^, JM Sanches^1^, JR Ribeiro^2^

#### ^1^Institute for Systems and Robotics, Instituto Superior Tecnico, Lisbon, Portugal, ^2^CHLN, ECMO Centre, Intensive Care Department, University Hospital Santa Maria, Lisbon, Portugal

*Critical Care* 2024, **28(Suppl 1):** P156

**Introduction:** The integration of machine learning (ML) into healthcare, particularly in intensive care, holds promise for enhancing quality and safety. This study aims to develop and validate an interpretable ML-based support system for managing patients with SARS-CoV-2 pneumonia treated with ECMO.

**Methods:** In an ECMO referral center, following approval from the ethical committee, protocol-driven, prospective collected data was represented as multivariate time series (MTSs). Each time instant of the patient-specific MTSs was labeled by a blinded ECMO expert intensivist with a ternary value: -1, 0 or + 1, representing, respectively, clinical deterioration, stabilization or improvement. The resulting datasets subsequently underwent additional processing and transformations for modeling. Different ML models were employed, including support vector machine (SVM) with varying kernels, and random forest (RF). Training and validation feature sets were generated through random assignment of patient-specific sequences, each one comprised of 3 consecutive samples, using a split ratio of 4:1. Each sequence was assigned the label corresponding to the third sample, having been exclusively considered, during training, sequences with a label of − 1 and + 1.

**Results:** 82 patients with SARS-CoV-2 pneumonia treated with ECMO generated thousands of labeled sequences. After model training and validation, performance was rigorously assessed (Table), resulting in the selection of a high-performing, well-calibrated and interpretable RF model. A clinical prediction score based on the RF model demonstrated a high ability to anticipate major clinical inflections, suggesting potential for clinical use (AUROC: 0.9226 for 4 h; 0.8940 for 8 h; 0.8535 for 12 h anticipation).

**Conclusions:** Our study illustrates the potential impact of ML systems when applied to complex and sensitive medical contexts, such as intensive care medicine, promising transformative advancements in intensive care practices.

**Table (abstract P156):** ML models

Metrics\Model	SVM (Linear)	SVM (Polynomial)	SVM (Radial basis function)	RF
Precision	0.9567	0.9918	0.9767	0.9959
Recall	0.9644	0.9526	0.9526	0.9565
F!-score	0.9606	0.9718	0.9659	0.9758
AUROC	0.9807	0.9906	0.9903	0.9938
AUPRC	0.9922	0.9962	0.9961	0.9973

## P157 Impact of obesity on outcomes in patients receiving extracorporeal membrane oxygenation: a systematic review and meta-analysis

### WWS Ng^1^, KC Leung^2^, RWH Hui^3^, P Yeung Ng^4^, CW Ngai^1^, SWC Sin^4^

#### ^1^Queen Mary Hospital, Adult Intensive Care Unit, Hong Kong, Hong Kong, SAR China, ^2^West London Renal and Transplantation Center, Hammersmith Hospital, Imperial College Healthcare NHS Trust, London, UK, ^3^Department of Medicine, School of Clinical Medicine, The University of Hong Kong, Hong Kong, Hong Kong, SAR China, ^4^Critical Care Medicine Unit, The University of Hong Kong, Hong Kong, Hong Kong, SAR China

*Critical Care* 2024, **28(Suppl 1):** P157

**Introduction:** Given the growing obesity pandemic, the impact of obesity on ECMO outcomes would be increasingly relevant to our daily practise. We hence performed a meta-analysis on this topic, integrating the latest evidence [1, 2].

**Methods:** Systematic literature search was conducted from inception until September 2023 on MEDLINE, Embase and the Cochrane Library using the terms “ECMO”, “obesity”, and their related terms. Our primary outcome was to assess the impact of obesity on in-hospital or 30-day mortality. The secondary outcomes were to evaluate the impact of obesity on major vascular complications (bleeding or ischemia), ECMO duration and hospital length-of-stay. A random-effects model (DerSimonian and Laird) was adopted for meta-analysis. Study quality was assessed using the Newcastle–Ottawa Scale.

**Results:** This meta-analysis included 24 studies from 2013 to 2023, including a total of 49,102 ECMO patients (mean age 51.14 ± 11.57 years). Obese patients, when compared with non-obese patients, had a significantly lower risk of in-hospital or 30-day mortality (risk difference − 4%, 95% CI − 7 to 0%, I^2^ = 68%, *p* = 0.03) (Figure). Regarding secondary outcomes, obesity had no significant association with major vascular complications (risk difference 1%, 95% CI − 2 to 4%, I^2^ = 29%, *p* = 0.38). Obesity was associated with significantly shorter hospital length-of-stay (mean difference − 2.39 days, 95% CI − 4.62 to − 0.15, I^2^ = 77%, *p* = 0.04), but had no impact on ECMO duration (mean difference 0.34 days, 95%CI -0.06 – 0.74, I2 = 44%, *p* = 0.10).

**Conclusions:** In summary, our meta-analysis showed that obesity is not a negative prognostic factor in ECMO. ECMO, as a life-saving treatment option in critically ill patients, should not be withheld from obese patients who are otherwise suitable candidates.

**References**

1. Rudym D et al. Am J Respir Crit Care Med. 2023;208:685–94
2. Peetermans M et al. Intensive Care Med. 2023;49:37–49

**Figure (abstract P157):**
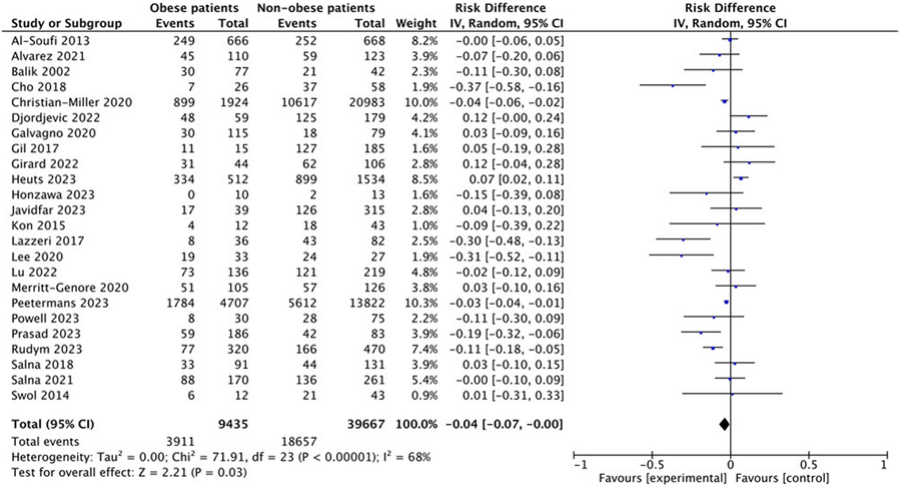
Forest plot of meta-analysis in mortality of obese and non-obese patients on ECMO support.

## P158 Motility of neutrophil granulocytes in patients with ECMO

### JM Methfessel^1^, NS Staschik^1^, FH Herbstreit^1^, TB Brenner^1^, AK Kraus^2^, ZC Cibir^2^, MG Gunzer^2^, S Dubler^3^

#### ^1^Department of Anaesthesiology and Intensive Care, Essen, Germany, ^2^Institute for Experimental Immunology and Imaging, Essen, Germany, ^3^Department of Anaesthesiology and Intensive Care, University Hospital Essen, Essen, Germany

*Critical Care* 2024, **28(Suppl 1):** P158

**Introduction:** Neutrophil granulocytes (NG) are the “first line of defence” against numerous infections (e.g., fungi and bacteria). Patients with severe acute respiratory distress syndrome (ARDS) due to respiratory infections are often treated with extracorporeal membrane oxygenation (ECMO). The impact of the ECMO circulation on the motility of immune cells has just been explored in a few studies and needs further investigation.

**Methods:** This was a prospective, single-centre study. Only patients who were successfully weaned from ECMO were included in the study. Blood samples were taken daily up to 30 days. A new prototype of microscope (ComplexEye) was used to image motility (percentage of moving cells and average speed of moving cells) of NG in a 96-well plate. Different NG-stimuli were used during the study (CXCL1, CXCL8 and fMLP). Subsequent analysis was done by artificial intelligence software.

**Results:** 28 patients were included. 9 patients could successfully weaned from ECMO and included in the final analysis. Results *during* ECMO therapy: The average percentage of moving cells was 70.73% in unstimulated NG (PBS), 87.15% (fMLP), 77.86% (CXCL1) and 84.05% (CXCL8) in stimulated NG (Figure upper panel). The average speed of moving cells in unstimulated NG was 5.61 µm/min and 12.15 µm/min (fMLP), 6.6 µm/min (CXCL1), 9.08 µm/min (CXCL8) in stimulated NG (Figure lower panel). Results *after* ECMO therapy: The average percentage of moving cells was 66.11% in unstimulated NG (PBS), 87.22% (fMLP), 69.29% (CXCL1), 84.94% (CXCL8) in stimulated NG (Figure upper panel). The average speed of moving cells in unstimulated NG was 5.3 µm/min and 11.7 µm/min (fMLP), 5.98 µm/min (CXCL1), 8.91 µm/min (CXCL8) in stimulated NG (Figure lower panel).

**Conclusions:** There was no significant difference in the percentage of moving cells or average speed of neutrophil granulocytes during ECMO therapy compared to no-ECMO therapy in 9 critically ill patients. This data shows that ECMO therapy does not alter chemotaxis in neutrophil granulocytes in critically ill patients with ARDS.

**Figure (abstract P158):**
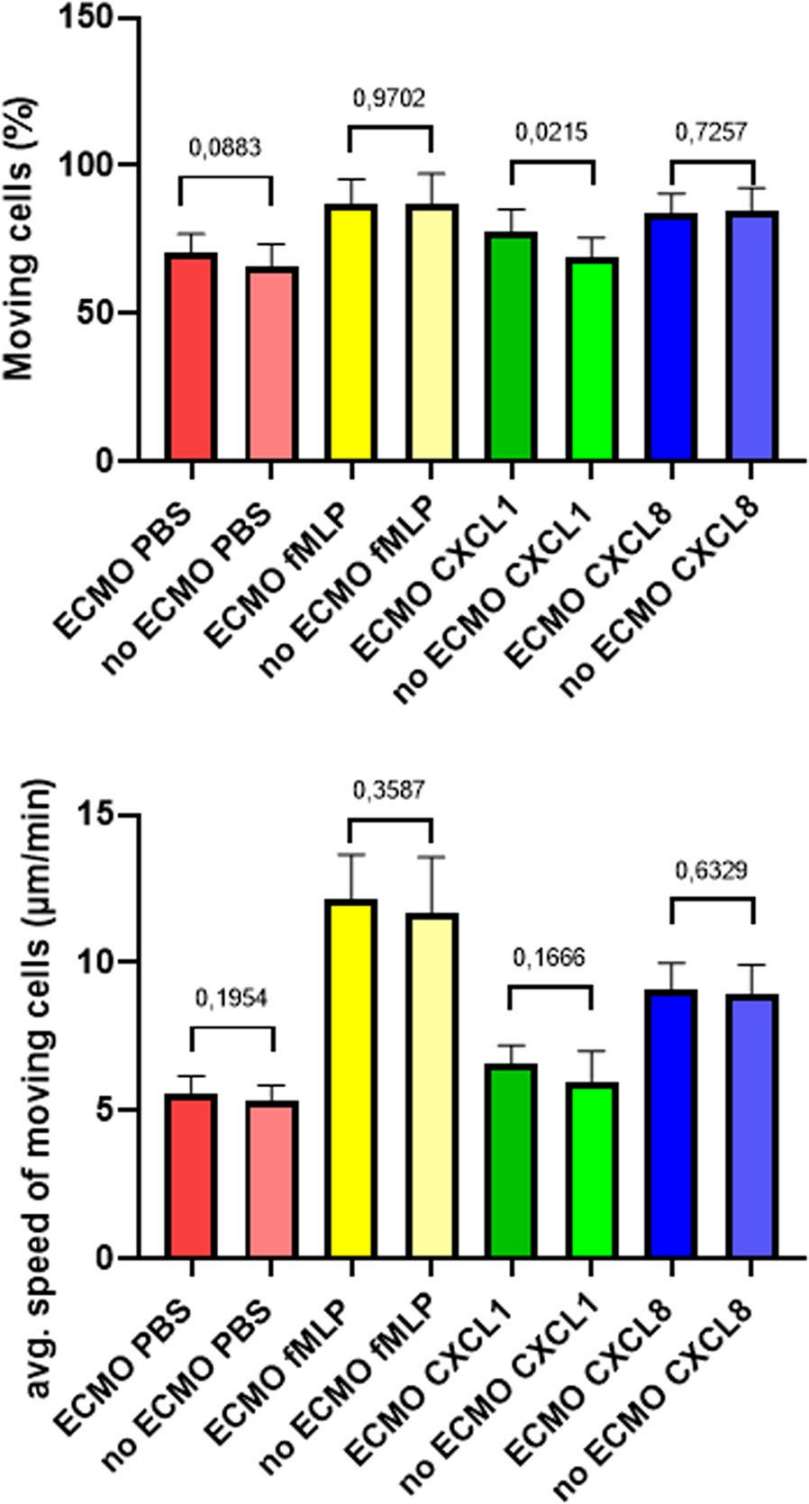
Upper panel. shows percentage of moving cells with different stimuli during and after ECMO therapy of 9 patients. Lower panel shows average speed of moving cells with different stimuli during and after ECMO therapy of 9 patients. Results are presented as mean and standard deviation. *P* values for paired t-tests.

## P159 Extended pre-ECMO mechanical ventilation: good outcome, huge burden

### J Graf^1^, R Pérez-Araos^2^, P Vargas^3^, R López^1^, R Agliati^1^

#### ^1^Clínica Alemana de Santiago, Departamento de Paciente Critico, Santiago, Chile, ^2^Clínica Alemana de Santiago, Facultad de Medicina Clínica Alemana - Universidad del Desarrollo, Santiago, Chile

*Critical Care* 2024, **28(Suppl 1):** P159

**Introduction:** Mechanical ventilation (MV) for > 7 days is considered a contraindication for VV ECMO. In a cohort of VV ECMO patients, we compared those with ≤ 7 days (early connection, EC) to those with > 7 days of pre-ECMO MV (delayed connection, DC). The primary objective is to determine if the duration of pre-ECMO MV independently affects long-term mortality (LTM). Additionally, to compare characteristics and course of patients with EC and DC to ECMO.

**Methods:** Single center retrospective cohort study. We included all patients on VV ECMO at our institution between 02/01/2017 and 08/31/2023. Demographic, physiological variables and severity scores were assessed pre ECMO. In-hospital course was recorded. Survival was followed till 09/30/2023. Variables are presented as median [IQR]. Univariate analysis comparing patients with EC and DC and a multivariate Cox regression analysis for LTM were performed. Significance was set at *p* < 0.05.

**Results:** 52 patients were included, 33 had COVID-19, 10 died in-hospital; 33 had EC and 19 DC. Hospital mortality was 24.2% and 10.5%, respectively (*p* = 0.293). Those who died had higher APACHE II (17.5 [10–23.3] vs. 10 [1–14] points, *p* < 0.01) and lower RESP score (0.5 [− 3 to 2.3] vs. 2 [0–4] points, *p* = 0.03). Follow up was 820 [248–1126] days; 2 more patients died after hospital discharge. In a model including pre-ECMO MV duration, APACHE II and RESP score, the hazard ratio for LTM of pre-ECMO MV duration was 0.944 [95% CI 0.846–1.05], (*p* = 0.307) for each additional day. The Table compares patients with EC and DC. Patients with DC had longer ECMO runs (21 [16–55] vs. 10 [5.5–24] days, *p* < 0.01) and ICU stay (56 [47–118] vs. 31 [13–57] days, *p* < 0.01) than those with EC. Patients with DC also developed more coagulopathy (68% vs. 36%, *p* = 0.026) and required more tracheostomies (84% vs. 39%, *p* < 0.01).

**Conclusions:** Pre-ECMO MV duration had no effect on LTM in VV ECMO patients. Patients with DC had a protracted course compared to those with EC. It is uncertain if these observations pertain only to COVID-19.

**Table (abstract P159):** Patient characteristics according to pre-ECMO mechanical ventilation duration

Characteristic	Early connection (≤ 7 days) n = 33	Delayed connection (> 7 days) n = 19	*p* value
Pre-ECMO MV, days	2 [1–4]	12 [9–15]	0.001
COVID-19	52%	84%	0.035
APACHE II, points	11 [7.5–17.5]	12 [8–14]	0.492
RESP score, points	2 [0–4]	1 [-1–4]	0.344
Lung injury score, points	3.25 [2.75–3.5]	3 [2.5–3.5]	0.430
PEEP, cmH2O	11 [7–14]	5.5 [4.8–10.8]	0.026
Respiratory rate, bpm	30 [24.8–37]	38 [31.5–41]	0.021

## P160 The advanced organ support (ADVOS) hemodialysis system balances blood pH within 24 h in patients with multiple organ failure and hypercapnic acidosis

### T Lahmer^1^, J Honigschnabel^2^, A Perez Ruiz de Garibay^3^, J Erber^1^, M Dibos^1^, S Rasch^1^

#### ^1^Klinik und Poliklinik für Innere Medizin II, Klinikum rechts der Isar der Technischen Universität München, ^2^Technische Universität München, Munich, Germany, ^3^ADVITOS GmbH, Medical Research, Munich, Germany

*Critical Care* 2024, **28(Suppl 1):** P160

**Introduction:** The dialysate fluid with customizable pH and bicarbonate content available within the ADVOS hemodialysis system supports a fast balancing of blood pH in patients with acidosis [1]. Considering that the rapidity of acidemia recovery is an independent risk factor for mortality [2], the aim of this work is to analyze the exact timing for pH correction and the main factors leading to it in patients with multiple organ failure (MOF) and hypercapnic acidosis treated with ADVOS.

**Methods:** Patients treated with ADVOS for MOF and hypercapnic acidosis in the tertiary care ICU of the University Hospital of Technical University of Munich (Germany) from 01/2021 to 02/2022 that survived at least 24 h were included in this retrospective analysis. The primary outcome was the time to reach a blood pH ≥ 7.35. During the ADVOS treatments pre- and post-dialyzer blood samples were taken to analyze the course of blood gases and the CO_2_ removal rates. The course of hemodynamic, hepatic, renal and ventilation parameters were documented.

**Results:** Each of the 24 patients (75% male, 61 years, SOFA 15) received a median of 5 sessions (total 134) with median blood flows of 300 mL/min, concentrate flows of 320 mL/min and dialysate pH of 8.5 under citrate anticoagulation alone (4%) or combined with UFH (96%). The median time to reach pH ≥ 7.35 was 4 h showing a significant pH increase at 24 h (7.21–7.39, *p* < 0.01). A total of 461 pairs of pre and post-dialyzer samples showed a median CO_2_ removal of 53 mL/min which reduced PCO_2_ (64–55, *p* < 0.241). The main determinants for CO_2_ removal were a dialysate with a lower bicarbonate content and a higher pH in combination with a higher blood flow (Figure).

**Conclusions:** A single session of ADVOS corrected blood pH within 4 h and supported the reduction of PCO_2_ through a median CO_2_ removal of 53 mL/min in patients with multiple organ failure and hypercapnic acidosis.

**References**

1. Allescher A et al. Artif Organs. 2021;45:1522–1532.
2. Jung B et al. Crit Care. 2011;15:R238.

**Figure (abstract P160):**
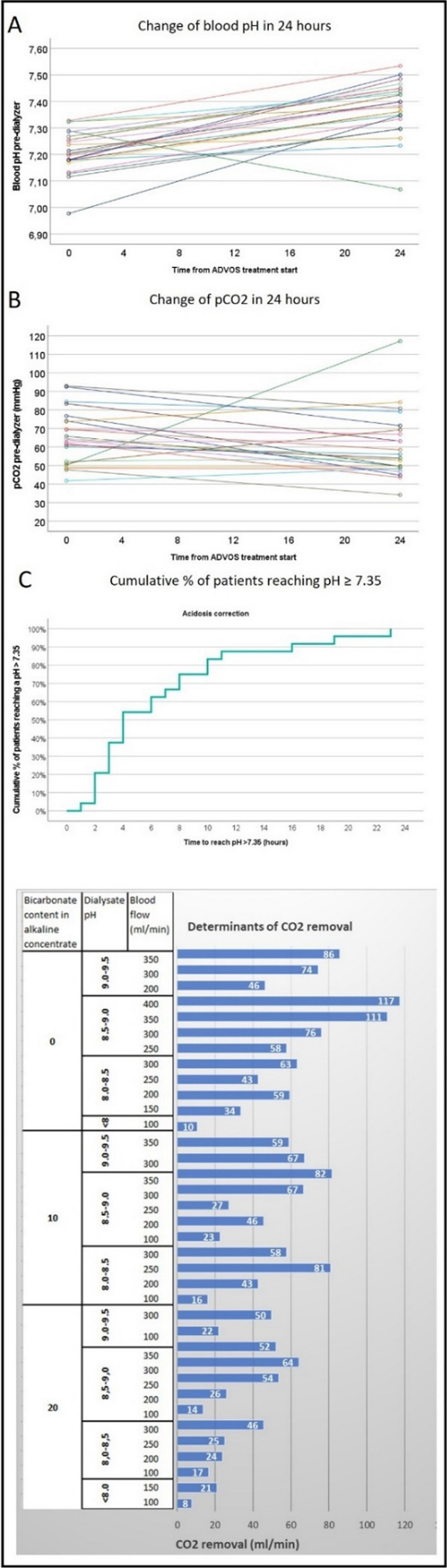
Effect of a 24 h-ADVOS treatment on acidosis and CO_2_ removal. **A** change of blood pH from baseline; **B** change of PCO_2_ from baseline; **C** time to pH correction; **D** main determinants of CO_2_ removal.

## P161 Analysis of the clinical effects of different nebulized inhalation devices on nurses´ inhalation exposure

### Z Qin, HJ Lin

#### The First hospital of Jilin University, Respiratory Department, Changchun, China

*Critical Care* 2024, **28(Suppl 1):** P161

**Introduction:** Secondary inhalation of medical aerosols is an important occupational hazard in clinical and domestic settings. Prolonged exposure to aerosols increases the risk and toxic effects of inhaling unnecessary medications in health care.

**Methods:** This experiment measured the aerosol inhalation rate and bacterial filtration efficiency of nurses when using different nebulizer inhalation devices in conjunction with nebulizer inhalation therapy, in line with the principle of ethical harmlessness and the limitations of experimental techniques, this part of the experiment were conducted using a laboratory simulation study to observe and record the use of patients in nebulizer inhalation therapy with three different nebulizers, A, B and C, respectively, 4L/min and 8L/min. The nebulization was carried out at an oxygen flow rate of 4L/min and 8L/min respectively, the nebulization solution was 10 mL of saline, the nebulization time was 15 min, and the number of inhaled aerosol particles of different particle sizes was measured using a dust particle counter, the number of bacteria before and after filtration was cultured using a bacterial culture dish, and the bacterial filtration efficiency was calculated.

**Results:** (1) The use of nebulizers B and C during nebulized inhalation was further effective in reducing the aerosol inhalation rate of nurses compared to A normal nebulizer and C was superior to B, i.e. the anti-aerosol dispersion nebulizer was effective. (2) The use of nebulizers B and C for nebulized inhalation is also effective in filtering bacterial microorganisms from the aerosol with > 95% filtration compared to A normal nebulizer.

**Conclusions:** These results will help to target improvements to nebulizers and to analyze and evaluate the use of different nebulizers for patients with respiratory diseases, providing evidence to support the selection of appropriate nebulizers for clinical patients and reducing occupational exposure of nurses.

## P162 Diagnosis and management of tracheoesophageal fistula in ICU

### S Tsoukala, N Rapti, M Zervos, T Gkaniatsou, T Melissopoulou, D Xanthis, P Vernikos

#### GNA Laiko, Athens, Greece

*Critical Care* 2024, **28(Suppl 1):** P162

**Introduction:** Tracheoesophageal fistula (TOF) in ICU is a rare but life-threatening condition even if diagnosed early or well-managed. The aim of our study is to assess diagnostic approaches, therapeutic strategies and outcomes of benign TOF in our ICU.

**Methods:** This is a single-center retrospective study of critically ill patients admitted in our ICU who developed TOF after intubation over a 5-year period (2019–2023). Causes and duration of hospitalization, presence/absence and duration of tracheostomy, presence/absence of nasogastric tube, need of ventilatory support and level of consciousness were recorded (Table). Diagnostic approaches included: esophagogastroscopy or flexible bronchoscopy (through nose or tracheostomy). Therapeutic approach included: tracheostomy tube of variable length, gastrostomy, TOF closure with stenting, surgical restoration and weaning from mechanical ventilation.

**Results:** 8 out of 1667 (0.47%) patients, 3 females/5 males with a mean age of 66 years developed a post intubation TOF. 5 patients underwent gastrostomy. TOF was treated surgically in 1 patient, whereas another one was stented as a bridging therapy until surgery. No patient was able to be weaned from mechanical respiratory support. All patients died except the one treated with stent, who is still hospitalized awaiting surgery approach.

**Conclusions:** TOF in ICU can be easily and early diagnosed by endoscopy of the upper digestive and/or respiratory tract. Weaning the patient from the ventilator is a prerequisite for a successful outcome, regardless of the treatment regimen.

**Table (abstract P162):** Patient characteristics

	Causes (number)	Mean duration (days)	Yes/No (number)	GCS≤8/ > 8
Hospitalization	Acute respiratory failure: 7, Stroke: 1	116.5	–	–
Tracheostomy	–	98.1	8/0	–
Nasogastric tube	–	–	8/0	–
Ventilatory Support	–	–	8/0	–
Level of consciousness	–	–	–	2/6

## P163 From the intensive care unit to ward services: a follow-up study by a tracheostomy team

### J Nogueira, F Sequeira, E Germano, R Alves, R Freitas, P Martins

#### Centro Hospitalar e Universitário de Coimbra, Intensive Care Medicine, Coimbra, Portugal

*Critical Care* 2024, **28(Suppl 1):** P163

**Introduction:** Tracheostomies are frequent in ICU in cases of prolonged weaning from mechanical ventilation. A tracheostomy team (TT) was created at our ICU to provide expertise in tracheostomy care. We aim to evaluate the efficiency of TT care and patient’s outcome.

**Methods:** Single-center retrospective cohort of patients with tracheostomy discharged from ICU to general wards. Those followed by TT (from June to November 2023) were compared to patients without follow-up (from June to November 2022). We applied parametric and non-parametric tests; statistical significance set at *p* < 0.05. Data expressed in median and interquartile range or percentage.

**Results:** Of 42 included patients, 17 were followed by TT. Patient’s characteristics (Table) were similar between groups, except for sex and neurological disorders. No statistical difference was found between TT follow-up and no follow-up groups regarding hospital length of stay (47 [36–66] vs. 52 [32–78] days, *p* = 0.86); decannulation rate (52.9% vs. 48%, *p* = 0.75); intrahospital tracheostomy time (23 [17–29] vs. 22 [10–31] days, *p* = 0.43); time to decannulation after ICU (17 [8–24] vs. 14 [5–20] days, *p* = 0.14); intrahospital mortality (29.4% vs. 24%, p 0.73). With no statistical difference, the documentation of tracheostomy complications was higher in TT follow-up group (41.2% vs. 24%, *p* = 0.24) as were the changes of the tracheal tube (47% vs. 24%, *p* = 0.12).

**Conclusions:** Unlike published literature [1], the main endpoints had no statistical difference between groups, possibly due to higher prevalence of neurocritical disorders in TT follow-up group. However, those with TT approach had more adverse events identified, allowing sooner referencing and treatment. More tracheal tubes were changed as part of the tracheostomy care bundle and weaning. Although there are few guidelines, we advocate expertise care by a TT. More studies are needed to establish comprehensively benchmark of TT effectiveness.

**Reference**

1. Speed L et al. J Crit Care 2013;28:261e1–e10

**Table (abstract P163):** Comparison of patient characteristics between groups

	No follow-up group (N = 25)	TT follow-up group (N = 17)	Total (N = 42)	*p*
Age (years)	64 [48–69]	62 [52–73]	62 [49–69]	0.847
Male sex, n (%)	22 (88%)	10 (58.8%)	32 (76.2%)	0.062
Charlson score	3 [1–4]	2 [2–3]	2 [1–3]	0.675
Neurological disorders	19 (76%)	14 (82.4%)	33 (78.6%)	0.716
Mechanical ventilation duration (days)	23 [13–30]	20 [15–24]	20 [13–25]	0.390
Orotracheal intubation duration (days)	16 [13–19]	16 [13–19]	16 [13–19]	0.690
ICU length of stay (days)	26 [20–35]	24 [17–28]	25 [18–31]	0.149

## P164 Innate immune characteristics of COVID pneumonia: impact of the period of the pandemia

### SP Chachali, K Leventogiannis, K Katrini, E Giamarellos-Bourboulis, N Antonakos

#### National and Kapodistrian University of Athens, 4th Department of Internal Medicine, Medical School, Athens, Greece

*Critical Care* 2024, **28(Suppl 1):** P164

**Introduction:** It is unknown if innate immune responses in severe COVID-19 vary over time periods of the pandemic.

**Methods:** In a prospective cohort study, blood samples were collected in two different time periods (March–December 2022 and February–May 2023) from 64 patients with severe COVID-19 and with bacterial pneumonia. Peripheral blood mononuclear cells (PBMCs) were stimulated by heat-killed *Candica albicans* and concentrations of TNF-alpha, interleukin (IL)-6, IL-10, IFN-gamma and interferon gamma induced protein (IP)-10 were measured in supernatants by an enzyme immunoassay.

**Results:** Concentrations of TNF-alpha were greater in the supernatants of PBMCs of patients with severe COVIND-19 than bacterial pneumonia (Figure). The potential of PBMCs presented variability for cytokine production depending on the time of the infection. More precisely, the production of IFN-gamma and IP-10 differed between the two time periods; IL-6, IL-10 and TNF-alpha did not have such trend (Figure).

**Conclusions:** This study shows that severe COVID-19 of the years 2022–2023 is characterized by hypersecretion of TNF-alpha by PBMCs being pro-inflammatory over bacterial CAP. The production of IFN-gamma and IP-10 differs largely between the time periods of the pandemic.

**Acknowledgement:** Published work was ssupported by the project 101046084—EPIC-CROWN-2 funded by the European Union. Views and opinions expressed are however those of the author(s) only and do not necessarily reflect those of the European Union or the European Health and digital Executive Agency.

**Figure (abstract P164):**
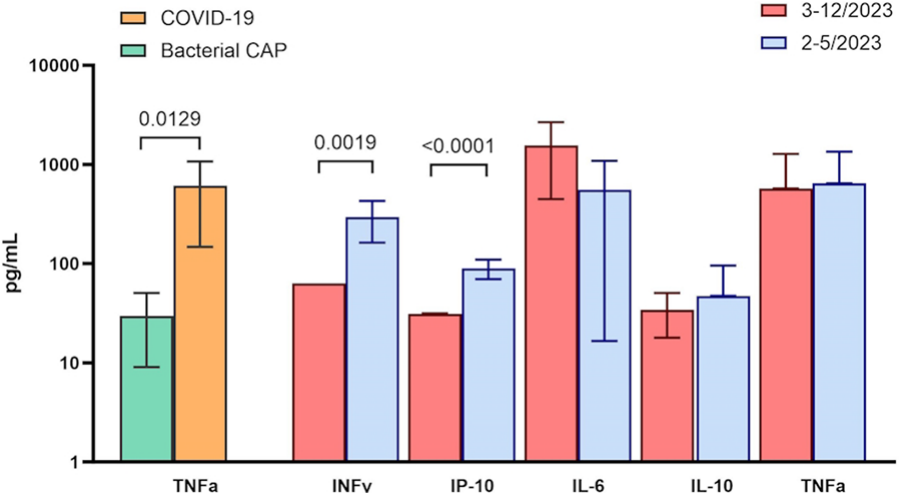
Cytokine concentrations in supernatants of *C. albicans*-stimulated PBMCs.

## P165 Postmortem analyses of myocardial microRNA expression in sepsis

### P Lehto^1^, T Saukko^2^, H Säkkinen^2^, H Syrjälä^2^, R Kerkelä^3^, S Skarp^3^, J Karhu^2^, T Ala-Kokko^1^

#### ^1^Oulu University Hospital, Critical Care Center, Oys, Finland, ^2^Oulu University Hospital, Oys, Finland, ^3^Oulu University, Oulu, Finland

*Critical Care* 2024, **28(Suppl 1):** P165

**Introduction:** Sepsis can lead to myocardial depression playing a significant role in sepsis pathophysiology, clinical care, and outcome [1]. MicroRNAs regulate mRNA expression to affect various physiological processes [2]. We investigated the expression of miRNA in myocardial autopsy specimens in critically ill deceased with sepsis and non-septic controls.

**Methods:** Myocardial autopsy tissue samples from adult patients deceased with sepsis (n = 15) and non-septic control tissue specimens collected from forensic autopsies (n = 15) were obtained. Predicted miRNA targets were retrieved from miRDB and pathway enrichment and classification was performed using PantherDB. The threshold set for up- and downregulated miRNA counts was a fold change of 2 or more and a *p* value of less than 0.05.

**Results:** A total of 32 miRNAs were found in the myocardial specimens. The largest difference in mean counts was with hsa-miR-12136 (mean difference 2933) and the highest fold change with hsa-miR-146b-5p (3,sevenfold increase compared to controls). The threshold for downregulated genes in sepsis compared to controls were achieved with hsa-miR-144-5p and hsa-miR-451a the largest difference in mean counts (mean difference − 44,281) and fold decrease (2, sixfold decrease compared to controls) with the latter. The threshold for upregulated genes in less severe cardiovascular failure (SOFA cardiovascular 0–2) compared to more severe cardiovascular failure (SOFA cardiovascular 3–4) were achieved in hsa-miR-144-5p, hsa-miR-451a and hsa-miR-4787-5p.

**Conclusions:** Several regulatory miRNAs were up- or downregulated in myocardial tissue of septic patients compared to non-septic subjects. The majority of miRNAs identified are associated with biological functions related to sepsis, including cardioprotection, cell adhesion and inflammatory response.

**References**

1. Boissier F et al. J Intensive Med 2021;2:8–16.
2. Antonakos N et al. Front Immunol. 2022;13:951798

## P166 Impact of dehydration on the mortality in patients with sepsis: a nationwide prospective cohort study

### SM Yoon^1^, KE Lee^1^, YH Ahn^1^, J Lee^2^, SM Lee^2^, HY Lee^1^

#### ^1^Seoul National University Hospital, Critical Care Medicine, Seoul, South Korea, ^2^Seoul National University Hospital, Pulmonary and Critical Care Medicine, Seoul, South Korea

*Critical Care* 2024, **28(Suppl 1):** P166

**Introduction:** Dehydration is the most common fluid disorder that increases mortality, morbidity, and hospital length of stay in hospital admitted patients [1]. Dehydration indicates a decrease in total body water, which can be assessed through plasma osmolality [2]. However, there have been few studies analyzing the impact of dehydration at the time of diagnosis on the prognosis of critically ill patients, especially those with sepsis. The objectives of this study is to investigate the effect of dehydration at the time of sepsis diagnosis on patient mortality using serum osmolality.

**Methods:** This nationwide prospective cohort study analyzed patients with sepsis admitted to the 19 tertiary hospitals between September 2019 and December 2021. Patients were classified as dehydration (≥ 300 mOsm/kg) and non-dehydration (< 300 mOsm/kg) group. The primary outcome of 30-day mortality and the secondary outcomes of ICU admission rate and discharged to another hospital were compared using logistic regression in both the unmatched and 1:1 propensity-score-matched (PSM) cohorts.

**Results:** A total of 9783 patients were included in the study, with 3720 in the dehydration group and 6063 in the non-dehydration group. The PSM cohort were comprised of 3158 individuals each, with all covariate imbalances alleviated. The median age of dehydrated patients and non-dehydrated patients were both 76; median SOFA score were 7, 6; respectively in matched cohort. Dehydrated patients exhibited an elevated risk of ICU admission (aOR, 1.50; 95% CI 1.34–1.67), a significant increase in 30-day mortality (aOR, 1.19; 95% CI 1.06–1.33), and a higher likelihood of being transferred to other hospital upon discharge (aOR, 1.25; 95% CI 1.09–1.43) (Table).

**Conclusions:** Among patients with sepsis, those who were dehydrated at the time of diagnosis had significantly higher 28-day mortality, were more likely to be transferred to another hospital upon discharge, and were more likely to be admitted to the ICU.

**References**

1. Munk T et al. Clin Nutr ESPEN 2021;43:415–419
2. Hooper L et al. BMJ Open 2015;5:e008846

**Table (abstract P166):** Results

	Non-dehydration	Dehydration	Adjusted OR (95% CI)
Unmatched	n = 6063	n = 3720	
ICU admission	2638 (43.5%)	2064 (55.5%)	1.47 (1.33–1.62)
30-day mortality	1419 (23.4%)	1259 (33.8%)	1.24 (1.12–1.38)
Matched	n = 3158	n = 3158	
ICU admission	1469 (46.5%)	1696 (53.7%)	1.50 (1.34–1.67)
30-day mortality	889 (28.2%)	992 (31.4%)	1.19 (1.06–1.33)

## P167 Septic shock is associated with a substantial change in the platelet lipidome

### M Dechamps^1^, E de Cartier^2^, J De Poortere^2^, V Robaux^2^, C Des Rosiers^3^, A Forest^3^, J Ambroise^4^, L Bertrand^2^, S Horman^2^, C Beauloye^2^

#### ^1^CARD, IREC, UCLouvain, Brussels, Belgium, ^2^CARD, IREC, Brussels, Belgium, ^3^Montreal Heart Institute Research Center, Metabolomic Platform, Montreal, Canada, ^4^IREC, UCLouvain, Centre de Technologies Moléculaires Appliquées, Brussels, Belgium

*Critical Care* 2024, **28(Suppl 1):** P167

**Introduction:** Sepsis is defined as life-threatening organ dysfunction caused by an impaired host immune response to infection. Sepsis is characterized by major endothelial dysfunction, microvascular alterations, and coagulopathy. In addition to their involvement in pathological hemostatic processes, platelets are key players in sepsis as they promote immunothrombosis. The composition of the platelet lipidome is critical to their function. However, the lipidomic profile of platelets during sepsis has never been studied.

**Methods:** Platelets were isolated from 48 septic and 48 control patients. Lipidomic analysis was carried out by untargeted liquid chromatography–mass spectrometry (QTOF).

**Results:** The lipidomic analysis identified 224 species and showed significant changes in the lipid composition of platelets during sepsis (Figure). Platelets from patients with septic shock showed increased levels of diacyl and triacylglycerols, as well as ceramides and deoxyceramides. A concordant decrease in sphingomyelin species was also observed. Excessive ceramide formation has been associated with multiple disorders, including atherosclerosis and cardiovascular disease. Regarding phospholipids, patients showed a reduction in lysophospholipids as well as alterations in the composition of fatty acid chains. An increase in short and (un)saturated fatty acid chains was observed and associated with a substantial reduction in phosphatidylcholines and phosphatidylethanolamines containing long polyunsaturated fatty acid chains (ω3 and ω6). The latter are key phospholipids for the generation of pro- and anti-inflammatory lipid mediators.

**Conclusions:** Critical changes in the platelet lipidome occur during sepsis. Upregulated lipids are mainly glycerolipids and ceramides, while lysophospholipids are drastically reduced. These changes as well as alterations in the composition of the fatty acyl chains of phospholipids might play a role in the pathophysiology of the disease.

**Figure (abstract P167):**
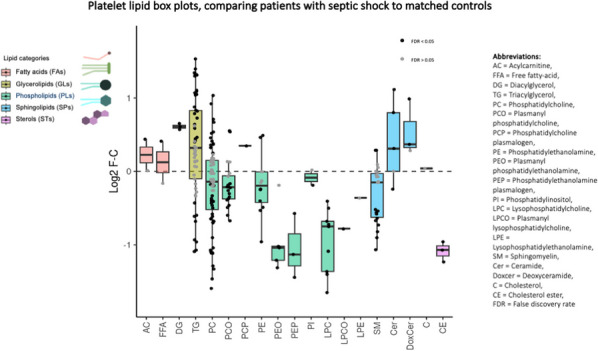
Box-plots of identified lipid classes/subclasses, comparing septic shock patients and controls: the Y-axis displays the Log2FC of the signal intensity values of each lipid species in septic vs control groups. The X-axis shows the lipid classes/subclasses, classified and color-coded based on their respective categories. Within those classes/subclasses, each dot represents a lipid species. The dot color distinguishes lipids exhibiting a significant difference (black) or not (grey) between the groups.

## P168 Use of NEWS2 and SeptiCyte® RAPID in acutely ill patients suspected of sepsis

### SC Cermelli^1^, R Balk^2^, K Navalkar^1^, R Brandon^1^, T Yager^1^, R Davis^1^

#### ^1^Immunexpress, Seattle, USA, ^2^Rush Medical College and Rush University Medical Center, Chicago, USA

*Critical Care* 2024, **28(Suppl 1):** P168

**Introduction:** The UK National Institute for Health & Care Excellence has recently drafted guidelines for use of a National Early Warning Score System (NEWS2) to identify sepsis patients in hospitals and estimate poor outcomes including ICU admission or 30-day mortality. In this study we calculated NEWS2 scores in a patient population that had been retrospectively diagnosed by a panel of 3 physicians into those with or without sepsis and for which SeptiCyte RAPID scores were obtained. SeptiCyte RAPID is a 1-h molecular host immune response assay that provides a likelihood of sepsis. The purpose was to (1) demonstrate the range of NEWS2 scores in an acutely ill patient population suspected of sepsis, (2) demonstrate the accuracy of SeptiCyte RAPID for identifying sepsis.

**Methods:** Data from clinical trials conducted at 10 hospitals in the USA and Europe with critically ill adult patients (n = 419) suspected of sepsis and with 2 or more signs of Systemic Inflammatory Response Syndrome (SIRS) was used [1]. Patients were stratified into groups based on NEWS2 score bins (0–4, 5–6, 7–19). An increasing SeptiScore® (0–15) represents increased likelihood of sepsis, and is determined for each patient by running SeptiCyte RAPID on the BiocartisIdylla™ platform. A partial NEWS2 score was calculated using 6/7 components in 285 patients [2]. Glasgow Coma Score was utilized instead of the ACVPU scale for the consciousness component as described by Zaidi et al. [3].

**Results:** Panel A on the Figure shows that SeptiScore identified sepsis from SIRS in 30% (n = 124) patients having low NEWS2 scores (0–4). Panel B on the Figure shows that 72% (n = 302) of all patients had at least 1 NEWS2 component score of 3, of which 56% (n = 171) had SIRS. In contrast, SeptiScore differentiated patients with sepsis from SIRS irrespective of an elevated NEWS2 single component score of 3.

**Conclusions:** SeptiScore correctly classified sepsis from SIRS regardless of the NEWS2 score stratification.

**References**

1. Miller RR et al. Am J Respir Crit Care Med. 2018;198:903–913
2. Kim DK et al. J Clin Med. 2021;10:1915
3. Zaidi H et al. BMC Public Health. 2019;19:1231

**Figure (abstract P168):**
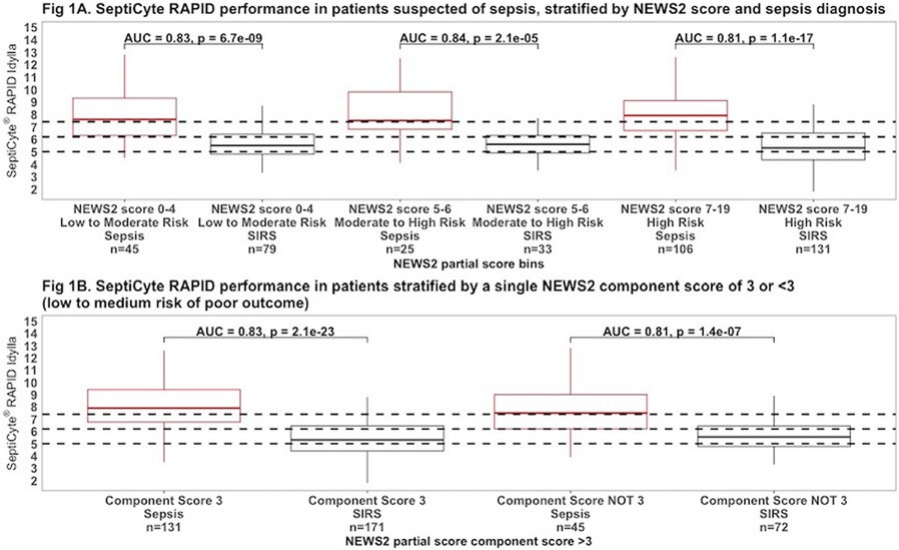
Figure **1A** SeptiCyte RAPID performance in patients suspected of sepsis, stratified by NEWS2 score and sepsis diagnosis. Figure **1B** SeptiCyte RAPID performance in patients stratified by a single NEWS2 component score of 3 or < 3.

## P169 Association of serum iron levels with mortality in sepsis patients: a retrospective study using the MIMIC-IV database

### J Jin^1^, Y Liu^2^, L Yu^1^, Q Zhou^1^

#### ^1^The university of Hong Kong-Shenzhen Hospital, Intensive Care Unit, Shenzhen, China, ^2^Shenzhen Hospital, Southern Medical University Shenzhen, Intensive Care Unit, Shenzhen, China

*Critical Care* 2024, **28(Suppl 1):** P169

**Introduction:** Iron is essential for oxygen transport and tissue repair [1]. Recent evidence links iron to sepsis pathogenesis and prognosis [2], but its association with sepsis mortality is unclear [3, 4]. This study aimed to examine the relationship between serum iron levels and 28-day all-cause mortality in sepsis patients.

**Methods:** This retrospective cohort study used the MIMIC-IV database. The study included 9645 adult sepsis patients with admission serum iron levels. Patients were divided into high and low iron groups based on a 100 μg/dL cut-off. Kaplan–Meier analysis compared 28-day all-cause mortality between groups. Cox proportional hazards modeling examined the association between iron levels and 28-day all-cause mortality, adjusting for confounders (Figure).

**Results:** A total of 1712 patients (17.8%) died within 28 days, with 311 deaths (23.7%) occurring in the high serum iron group. Kaplan–Meier analysis showed a statistically significant increase in 28-day all-cause mortality in the high-serum iron group. Multivariate Cox proportional hazards analysis suggested that serum iron levels associated with 28-day all-cause mortality in sepsis patients (HR 1.60), with a J-shaped curve relationship on RCS. Subgroup analysis showed that iron levels, age, sex, and vasopressor use significantly influenced mortality.

**Conclusions:** Serum iron levels show a significant association with 28-day all-cause mortality in individuals with sepsis and specifically are more predictive in patients under 60 years of age, males, and patients using vasodilators. Further prospective studies are warranted to confirm these findings.

**References**

1. Weiss G et al. Blood. 2019;133:40–50
2. Mohus RM et al. Intensive Care Med. 2018;44:1276–83
3. Piagnerelli M et al. Crit Care. 2004;8:306–7.
4. Piagnerelli M et al. Acta Clin Belg. 2013;68:22–7.

**Figure (abstract P169):**
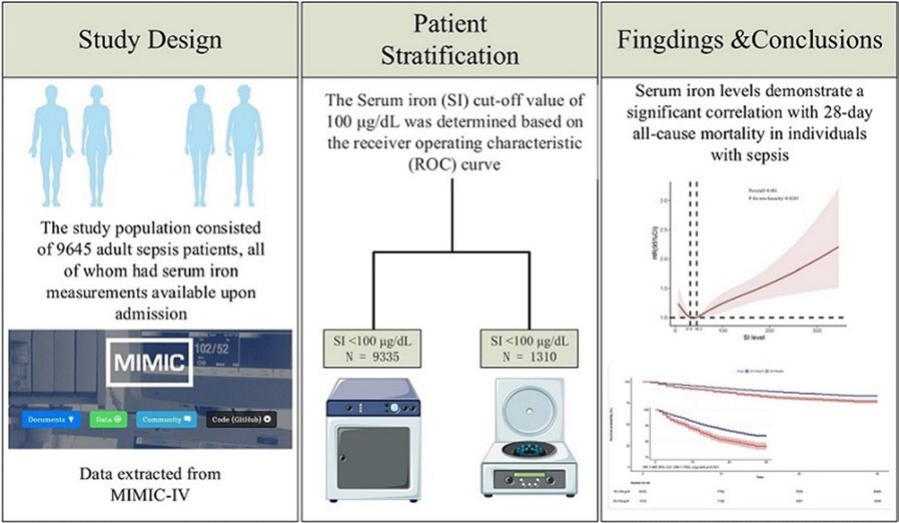
Graphic abstract.

## P170 A proteomic definition of sepsis

### L Mellhammar, E Malmström, A Scott, A Goch Gustaffson, T Mohanty, A Bakochi, L Thelaus, F Kahn, J Malmström, A Linder

#### Faculty of Medicine, Department of Clinical Sciences, Lund, Sweden

*Critical Care* 2024, **28(Suppl 1):** P170

**Introduction:** Sepsis is a clinical syndrome; it has been re-defined as life-threatening organ dysfunction caused by a dysregulated host response to infection (sepsis-3). If the sepsis definition and criteria capture the wrong patients there is a risk of dilutional effect on research and a risk to miss diagnosing patients or delay treatment. By utilizing high-throughput proteomics and explainable machine learning, we look to further interrogate the unique molecular definition of sepsis to improve diagnosis and personalize patient care.

**Methods:** Patients were eligible for inclusion if ≥ 18 years and admitted to the emergency department at Skåne University Hospital, Lund, Sweden with suspected sepsis. Plasma samples were withdrawn at admission and analyzed using data-independent acquisition trapped ion-mobility mass spectrometry to generate comprehensive proteome maps. Through statistical analysis, explainable machine learning, and novel feature selection methods we investigated the unique proteome signatures associated with different clinical parameters to provide a molecular definition of sepsis.

**Results:** In 1380 patients, we identified panels of proteins that were predictive for septic shock. We combined each protein panel into a consensus molecular signature to train a machine learning model that predicts septic shock with high accuracy. Finally, we used this classifier to stratify patients into different probability of belonging to the same entity as septic shock and highlight how increasing risk of being proteomic septic shock like is associated with higher mortality (Figure).

**Conclusions:** Through a combination of high-throughput mass spectrometry and explainable machine learning we identified unique biological signatures associated with the clinical manifestations of sepsis. These findings could be directly applied in the clinic as a proteomic definition of sepsis to provide more accurate sepsis diagnosis and personalized care based on the predicted risk category.

**Figure (abstract P170):**
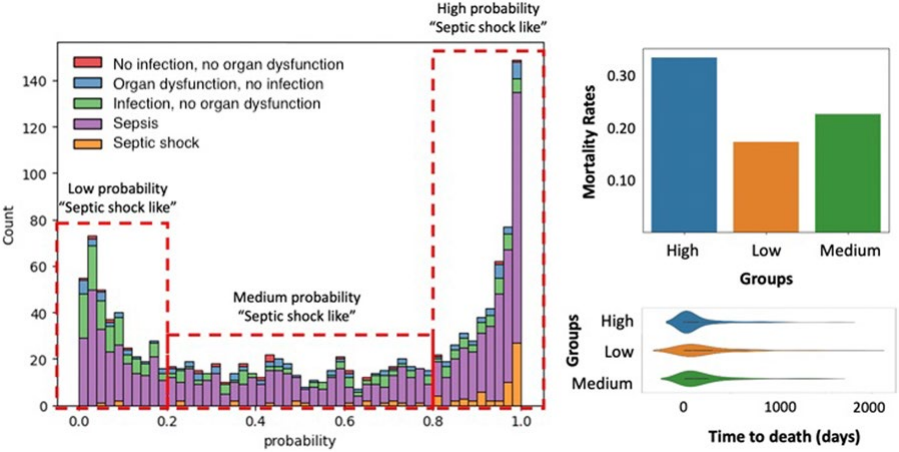
Probability of patients being septic shock like and its mortality.

## P171 Prevalence, early predictors, and outcomes of sepsis in neurocritical illnesses: a prospective cohort study

### F Yuan, LW Wang, ZM Ma, ZJ Jiang, HL Li, AL Lu, SW Wu, HL Lu, WW Wen

#### The Second Affiliated Hospital of Guangzhou University of Chinese Medicine, Department of Neurocritical Care, Guangzhou, China

*Critical Care* 2024, **28(Suppl 1):** P171

**Introduction:** Patients with neurocritical illness are an under-recognized population at high risk of sepsis. We aimed to investigate the prevalence, early predictors, and outcomes of sepsis in neuro-ICU.

**Methods:** Daily and accumulative incidences of sepsis in neuro-ICU were explored. Demographics, medical history, baseline disease severity scores, and baseline biomarkers regarding inflammation, immunology, organ function, and nutritional status were collected and analyzed as potential predictors of sepsis. Logistic regression analyses were used to determine the independent predictors, and a nomogram was used to estimate the individual probability of sepsis in neuro-ICU (Figure).

**Results:** 153 patients were included in this study. Fifty-nine (38.6%) patients developed sepsis, and 21 (14%) patients developed septic shock. More than 86% of septic cases occurred within the first week. SOFA score (RR 1.334, *p* = 0.026), history of diabetes (RR 2.346, *p* = 0.049), and transferrin (RR 0.128, *p* = 0.042) on admission are independent predictors of sepsis. Septic patients had significantly higher mortality (*p* = 0.011), higher medical cost (*p* = 0.028), and a lower rate of functional independence (*p* = 0.010), compared to patients without sepsis.

**Conclusions:** Sepsis afflicted more than one-third of neurocritically-ill patients and occurred mostly in the first week of admission. History of diabetes, serum transferrin, and SOFA score on admission were early predictors. Sepsis led to significantly worse outcomes and higher medical costs.

**Figure (abstract P171):**
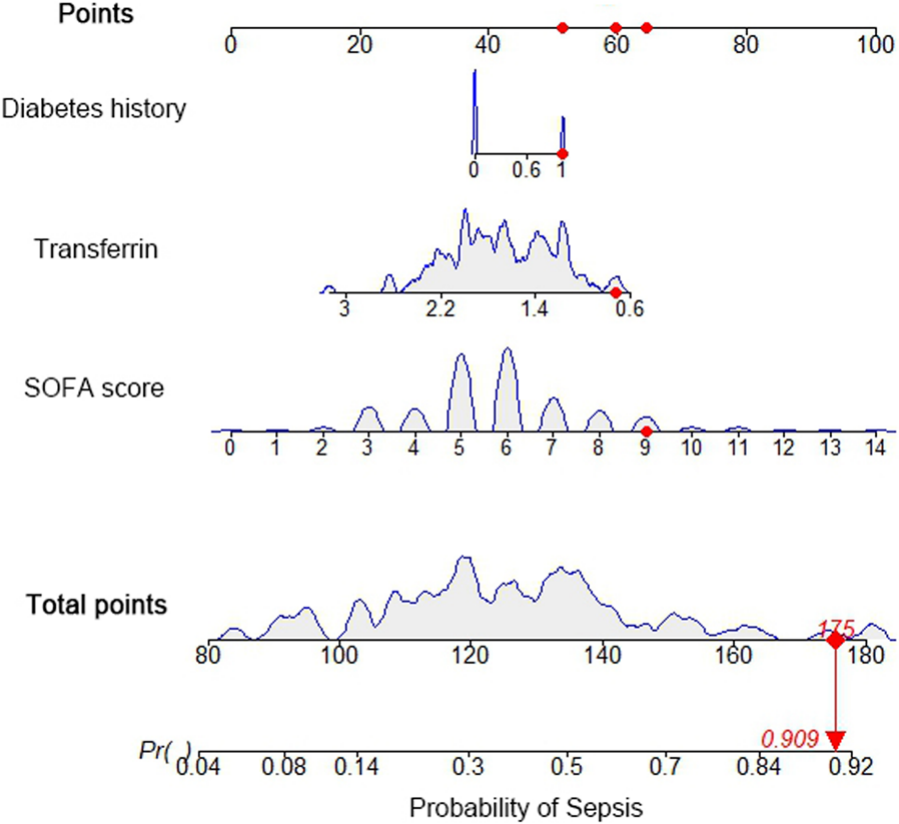
Nomogram to estimate the individual probability of developing sepsis. The areas under the blue curves depict the distribution of independent risk factors. The red dots show an example of calculating the probability of sepsis in a patient with a history of diabetes, a baseline transferrin level of 0.7 g/L, and a baseline SOFA score of 9 (total score is 175, the probability is 0.909).

## P172 Positive predictive value of procalcitonin to identify viral infection in community-acquired respiratory infections

### A Silva, J Martins, A Branco, I Zão, G Cabral-Campello, T Cardoso

#### Centro Hospitalar Tâmega e Sousa, Intensive Care, Penafiel, Portugal

*Critical Care* 2024, **28(Suppl 1):** P172

**Introduction:** Opting for antibiotic treatment in an individual patient is complex and should take into account factors such as the likelihood of bacterial infection before testing, the severity of the presentation, and the concentration of serum biomarkers. Among various markers of inflammation, procalcitonin (PCT) is under scrutiny to assess its accuracy in diagnosing bacterial infections [1]. This study aims to explore the accuracy of PCT value on ICU admission to discriminate viral from bacterial in community-acquired respiratory infections.

**Methods:** Retrospective cohort study, between May 1st 2021 and June 30th 2022, including all adult patients with microbiologic documented community-acquired respiratory infection admitted to the ICU, of a tertiary care hospital. A cut-off value of PCT < 0.5 ng/mL was used. For the primary outcome patients were divided in two groups: only viral infection or bacterial infection/co-infection.

**Results:** A total of 95 patients were included: 61.1% (n = 58) males, with median [IQR] age of 63 [48–74] years. The median [IQR] SAPS II was 30 [24–44] and SOFA score 4 [3–7] points. Among the patients, 66% (n = 63) had a viral pneumonia and 34% (n = 32) bacterial infection (6 were co-infections). The median [IQR] PCT value was 0.28 [0.11–1.28] ng/mL in viral infections and 4 [0.4–20] ng/mL in bacterial infections. For the primary outcome the sensitivity (95% CI) was 64% (50–75%), the specificity (95% CI) 75% (56–89%), positive predictive value for viral infection was 83% (73–90%). This value of PCT had a moderate ability to exclude bacterial infection with a AUROC curve (95% CI) of 0.69 (0.58–0.80).

**Conclusions:** Our study reinforces the utility of PCT to aid in the decision to start or stop antibiotic in community-acquired respiratory tract infection with virus isolation. This research contributes to the ongoing exploration of biomarkers effectiveness in enhancing the clinical approach to respiratory infections.

**Reference**

1. Simon L et al. Clin Infec Dis. 2004;39:206–17

## P173 Can heparin binding protein be of additive value to lactate for the identification of sepsis alert patients in need of vasopressor treatment?

### A Goch Gustafsson, L Mellhammar, A Linder

#### Lund University, Infection Medicine, Lund, Sweden

*Critical Care* 2024, **28(Suppl 1):** P173

**Introduction:** Sepsis and septic shock are conditions with high mortality. For fluid unresponsiveness, lactate is the gold standard biomarker for vasopressor treatment despite its inadequate predictive value. Heparin binding protein (HBP) is a protein mainly stored in granules of neutrophils with increased plasma levels in sepsis and septic shock. Due to its mediation of increased capillary permeability, it causes outflow of plasma. This raises the question whether HBP can play a role in the development of fluid unresponsiveness in sepsis and be of additive value to lactate in predicting vasopressor treatment in sepsis alert patients. With this study, we aimed to investigate admission HBP and lactate plasma levels combined predictive value of vasopressor treatment in sepsis alert patients.

**Methods:** Sepsis alert patients arriving to the emergency department at Lund’s university Hospital were included between 2017 and 2022. For each patient, a blood sample were collected prospectively and later analyzed for HBP using fluorescence dry quantitative immunoassay. Clinical data was extracted retrospectively.

**Results:** 1357 patients were included in this study. Using binary logistic regression together with ROC analysis, subgroup analysis on non-COVID patients with either no treat limit or no-CPR treat limit before admission and no change of treat limit within 72 h or within 72 h but after vasopressor treatment (n = 406) yielded an AUC of 0.810 (95% CI 0.758–0.862; *p* < 0.05) to predict vasopressor treatment within 72 h when combining admission plasma levels of HBP and lactate (Figure).

**Conclusions:** In sepsis alert patients, a model that will be implemented nationally in Sweden, our study shows that the combined use of admission plasma levels of HBP and lactate display a promising correlation to need of vasopressor treatment.

**Figure (abstract P173):**
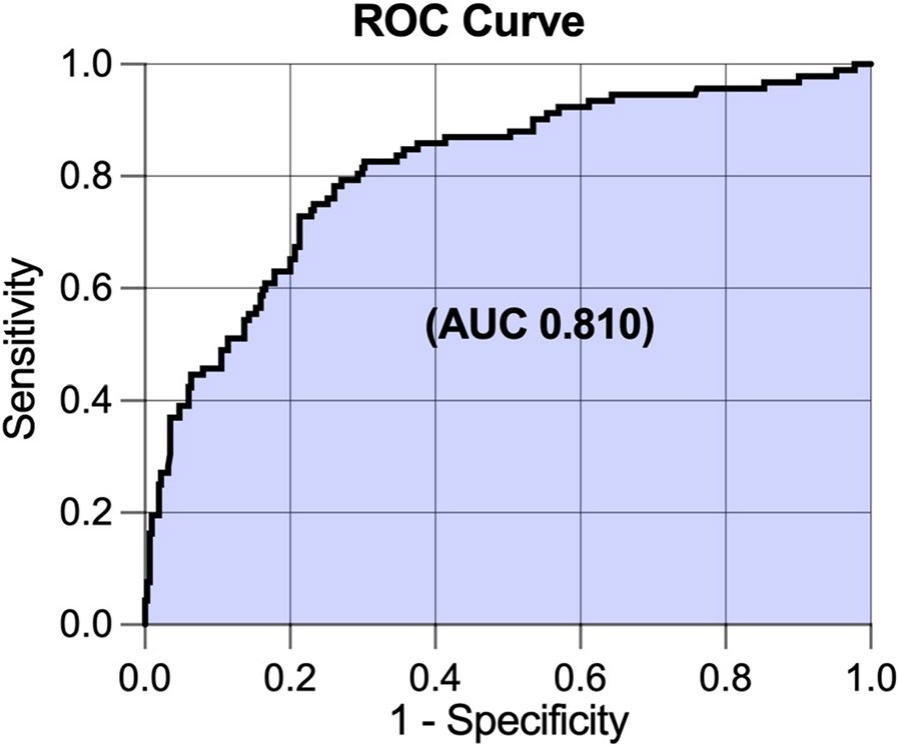
ROC curve displaying admission HBP and lactate plasma levels combined ability of predicting vasopressor treatment within 72 h from admission in sepsis alert patients. 95% CI 0.758–0.862, *p* < 0.05.

## P174 Pancreatic stone protein plasma level of COVID-19 patients with sepsis

### G Melegari^1^, F Carrieri^1^, S Pollonara^2^, C Soriani^3^, T Trenti^4^, P Baffoni^1^, A Pignatti^1^, F Gazzotti^1^, E Bertellini^1^, A Barbieri^2^

#### ^1^Azienda Ospedaliero Universitaria di Modena, Department of Anaesthesia and Intensive Care, Modena, Italy, ^2^University of Modena and Reggio Emilia, School of Anaesthesia And Intensive care, Modena, Italy, ^3^University of Modena and Reggio Emilia, Anaesthesia and Intensive Care, Modena, Italy, ^4^Azienda Ospedaliero Universitaria di Modena, Department of Laboratory Medicine and Pathology, AUSL/AOU, Modena, Italy

*Critical Care* 2024, **28(Suppl 1):** P174

**Introduction:** The use of pancreatic stone protein (PSP) as a potential biomarker for septic shock and early detection of septic conditions in critically ill patients has gained attention recently. COVID-19 patients are at a high risk of developing septic infections. The main objective of this study was to investigate the correlation of plasma levels of PSP in a COVID-19 intensive care unit population to evaluate its role as a biomarker for septic conditions [1].

**Methods:** Ethical approval was granted by our health system's Ethics Committee (Azienda Ospedaliero Universitaria di Modena, Reference number 784/2021). We collected clinical information and blood samples from COVID-19 patients in the ICU at four different time points: upon admission (T0), 72 h later (T1), five days later (T2), and finally, seven days later. We utilized a point-of-care system to measure the PSP plasma level. We considered septic patients using sepsis guideline criteria. Using the probit model, we measured the correlation between PSP and septic infection diagnosis. Then, we compared PSP, C-reactive protein (CRP) procalcitonin (PCT) with the Receiving Curve Operator (ROC) for septic conditions.

**Results:** We collected 80 blood samples from 21 COVID-19 patients; we found a correlation between PSP levels and septic conditions. (*p* < 0.041). PSP showed an ROC of 0.59, higher than CRP and PCT, with a *p* value of 0.000 (Figure).

**Conclusions:** These first results suggest the possible advantages of monitoring PSP plasma levels to detect infections in COVID-19 patients early. Additional data are needed to confirm these results.

**Reference**

1. Pugin J et al. Crit Care. 2021;25:151.

**Figure (abstract P174):**
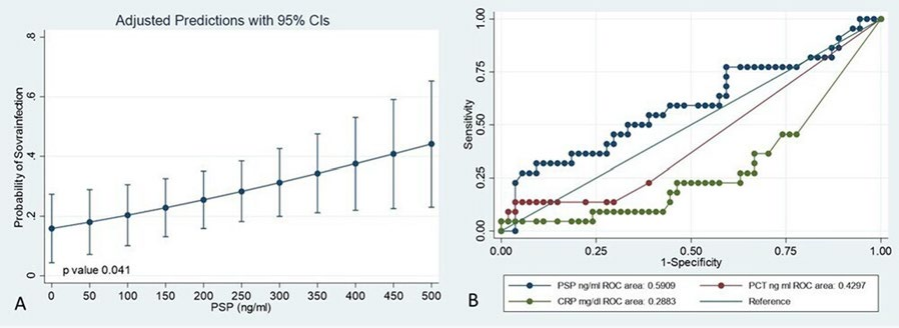
Panel **A** shows the probability of having infections on the Y-axis and the X-axis, the level of PSP. Panel **B** shows the ROC Curve comparison among PSP, CRP and PCT.

## P175 Pancreatic stone protein accuracy in infection diagnosis and prognosis in liver failure patients: a case series

### D Lopes^1^, JP Bandovas^2^, B Chumbinho^2^, C Espírito Santo^1^, B Ferreira^1^, L Val-Flores^1^, R Pereira^1^, F Cardoso^3^, L Bento^4^, P Póvoa^5^

#### ^1^Curry Cabral Hospital, Central Lisbon University Hospital, Department of Intensive Care Medicine (Unidade de Cuidados Intensivos Polivalente), Lisboa, Portugal, ^2^Curry Cabral Hospital, Central Lisbon University Hospital, Department of General Surgery, Lisboa, Portugal, ^3^Curry Cabral Hospital, Central Lisbon University Hospital, Transplant Unit, Lisboa, Portugal, ^4^São José Hospital, Central Lisbon University Hospital, Department of Intensive Care Medicine (Unidade de Urgência Médica), Lisboa, Portugal, ^5^São Francisco Xavier Hospital, CHLO, Intensive Care Unit 4, Department of Intensive Care, Lisboa, Portugal

*Critical Care* 2024, **28(Suppl 1):** P175

**Introduction:** Pancreatic stone protein (PSP) is a protein secreted by the gastro-intestinal tract, that seems to have a higher accuracy in sepsis detection comparing to other biomarkers [1]. It has never been studied in patients with liver failure (LF), that present an inflammatory imbalance predisposing to infection and organ failures [2]. Our purpose was to assess accuracy of PSP on diagnosis of infection and prognosis in LF.

**Methods:** We conducted a prospective observational study on adult patients with LF consecutively admitted to the ICU of a university hospital in 2022–2023. Ongoing overt infection was an exclusion criteria. Daily measurements of biomarkers were performed until discharge, death or for 21 days. Analysis was performed adjusting baseline for first infection episode (median on D3), which was the Reference for those non infected.

**Results:** 16 patients were included, 7 with acute and 9 with acute-on-chronic LF: median age 54(42–64) years, half female, SAPS II 57(49–67) and SOFA 12(10–12) on D1. Median duration of vasopressors and renal replacement therapy was 5 days and 4 for mechanical ventilation (IQR3-8; 2–9; 2–8, respectively). Half of patients were transplanted, 6 were submitted to plasma exchange or hemoadsorption (Cytosorb®), 8 had an infection and 7 died. 216 PSP measurements were performed, with a marked intraindividual variability between days. Infected patients showed higher values of PSP without statistical significance and PCT (*p* < 0.05 on D + 1 and D + 2) vs. non-infected ones (Figure). PSP was higher in non-survivors vs. survivors (*p* < 0.05 from D-1 to D + 4). A positive strong correlation was observed between PSP and SOFA from D + 1 to D + 4 (*p* < 0.05, 0.7 < r < 0.8; 0.5 < R^2^ < 0.6) and a trend on D + 5.

**Conclusions:** In this pilot study we showed PSP rises in LF, with higher levels in infected patients. It seems to be a potential useful biomarker assessing prognosis in patients with LF. Further studies are needed.

**References**

1. Prazak J et al. Crit Care. 2021;25:182
2. Rovegno M et al. Ann Hepatol. 2019;18:543–552

**Figure (abstract P175):**
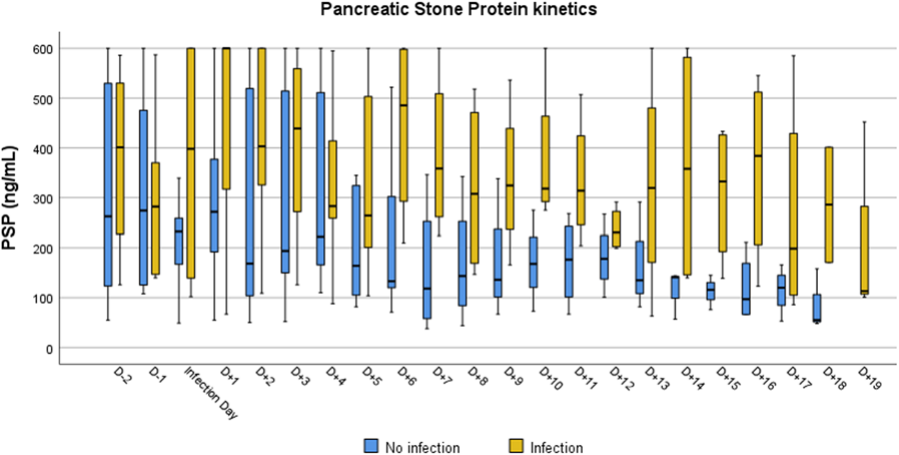
Pancreatic stone protein kinetics between patients with or without an infection.

## P176 Circulating H3.1-nucleosomes as an index of sepsis-induced immunoparalysis: preliminary data from the ImmunoSep trial

### C Psarrakis^1^, E Mouloudi^2^, A Ioakeimidou^3^, M Ntaganou^4^, M Patrani^5^, E Kondili^6^, E Gkeka^7^, M Netea^8^, E Giamarellos-Bourboulis^1^

#### ^1^National and Kapodistrian University of Athens, 4th Department of Internal Medicine, Medical School, Athens, Greece, ^2^Ippokrateion General Hospital, Intensive Care Unit, Thessaloniki, Greece, ^3^Asklipeion General Hospital, Intensive Care Unit, Voula, Greece, ^4^Sotiria Hospital of Chest Diseases, Intensive Care Unit, Athens, Greece, ^5^Korgialeneion Benakeion General Hospital, Intensive Care Unit, Athens, Greece, ^6^University of Crete, Intensive Care Unit, Herakleion, Greece, ^7^Aristotle University, Intensive Care Unit, Thessaloniki, Greece, ^8^Radboud University Medical Center, Department of Internal Medicine, Nijmegen, Netherlands

*Critical Care* 2024, **28(Suppl 1):** P176

**Introduction:** ImmunoSep (Clinicaltrials.gov NCT04990232) is a randomised, double-blind, double-dummy controlled trial classifying patients’ immune endotype measuring serum ferritin and the expression of HLA-DR/CD14^+^-cell by flow cytometry. As flow cytometry is expensive and not routinely available, we investigated the measurement of nucleosomes to provide equivalent classification.

**Methods:** Enrolled patients meet the Sepsis-3 criteria and are classified into macrophage activation-like syndrome (MALS) when serum ferritin is > 4420 ng/mL and into sepsis-induced immunoparalysis (SII) when ferritin is < 4420 ng/mL and the absolute number of HLA-DR receptors on CD14-monocytes is < 5000/cell. Among patients screened plasma H3.1-nucleosomes (Nu.Q® NETs CE-IVD, Belgian Volition), ferritin and HLA-DR/CD14^+^-cell were measured in parallel. The performance of Nu.Q® NETs to select HLA-DR less than 5,000/cell was analysed.

**Results:** A total of 104 patients were analysed, all measurements were completed in parallel. Nucleosome levels were higher in patients with MALS and SII than in patients unclassified to a specific immune endotype (Figure). There was positive correlation between nucleosomes and ferritin (r_s_: + 0.234; *p*: 0.017) and a negative correlation between nucleosome levels and the absolute number of HLA-DR/CD14-cell (r_S_: − 0.303; *p*: 0.002). The area under the ROC curve of nucleosomes to select for patients with less than 5000 HLA-DR receptors/CD14-cell was 0.66 (0.55–0.76; *p*: 0.006). The Youden index set at > 1000 ng/mL selected with odds ratio 3.13 (95% confidence intervals 1.36–7.39; *p*: 0.007) for the presence of SII. At this cut-off the sensitivity and negative predictive value of nucleosomes for the classification of SII is 62.9% and 77.6% respectively.

**Conclusions:** In hospital settings where flow cytometry is not available, measurements of nucleosomes by Nu.Q® NETs can be used as a classification tool for SII.

**Acknowledgement:** Work was funded by Project No 847422/IMMUNOSEP by the European Union’s Horizon 2020 Programme.

**Figure (abstract P176):**
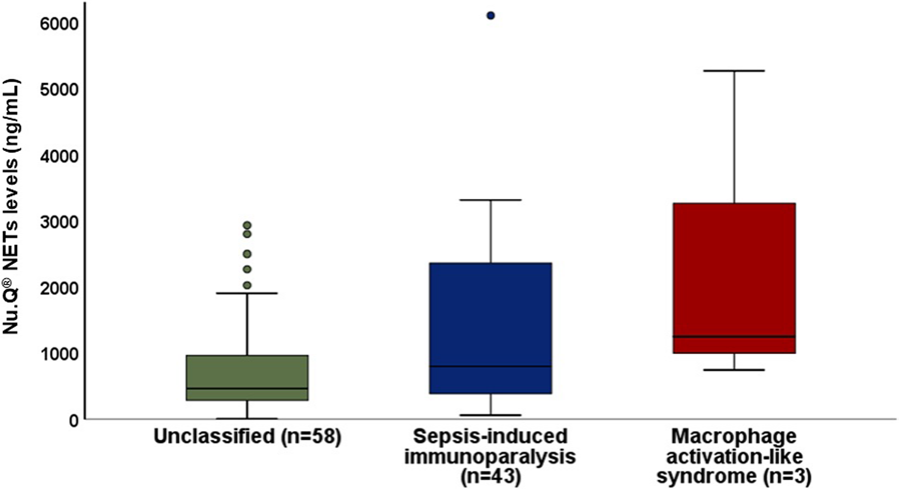
Box plot of circulating H3.1-nucleosomes (Nu.Q® NETs) measurements in patients screened for enrolment in the ImmunoSep trial according to their final classification using ferritin and HLA-DR expression on monocytes.

## P177 Molecular sepsis subphenotypes

### L Mellhammar, E Malmström, A Scott, A Goch Gustafsson, T Mohanty, A Bakochi, L Thelaus, F Kahn, J Malmström, A Linder

#### Faculty of Medicine, Department of Clinical Sciences, Lund, Sweden

*Critical Care* 2024, **28(Suppl 1):** P177

**Introduction:** A subphenotype is a subset of a patient population defined by observable characteristics, distinguished from the population as a whole by natural history, disease manifestation and/or response to treatment. In sepsis, one has to establish subphenotypes without an exact definition. Subphenotypes may be useful in sepsis to improve diagnosis and personalize patient care.

**Methods:** Patients were eligible for inclusion if ≥ 18 years and admitted to the emergency department at Skåne University Hospital, Lund, Sweden with suspected sepsis. Plasma samples were withdrawn at admission and analyzed using data-independent acquisition trapped ion-mobility mass spectrometry to generate comprehensive proteome maps. Through statistical analysis, explainable machine learning, and novel feature selection methods we investigated the unique proteome signatures associated with different clinical parameters such as different organ dysfunctions and infections. Clusters were created out of probabilities to belong to these organ dysfunctions and infections according to proteomic data.

**Results:** In 1380 patients, we identified panels of proteins that were predictive for organ dysfunction and infections. Five clusters or subphenotypes were created out of the probabilities of having organ dysfunctions and infections. These subphenotypes are projected in a diagram, the subphenotypes differed in mortality (Figure).

**Conclusions:** Through a combination of high-throughput mass spectrometry and explainable machine learning we identified subphenotypes associated with the biological signatures of clinical manifestations of sepsis. The subphenotypes could be used for personalized care in sepsis.

**Figure (abstract P177):**
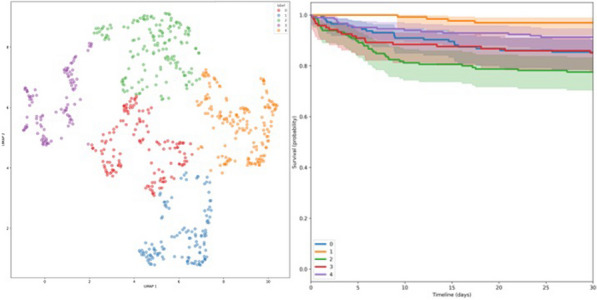
Subphenotypes and their mortalities.

## P178 Sepsis clinical phenotypes complement but do not overlap with immunological phenotypes: secondary analysis of a randomized controlled trial

### E Karakike^1^, N Antonakos^2^, A Kotsaki^2^, M Kyprianou^2^, EJ Giamarellos-Bourboulis^2^

#### ^1^2nd Department of Critical Care, National and Kapodistrian University of Athens, Athens, Greece, ^2^4th Department of Internal Medicine, National and Kapodistrian University of Athens, Athens, Greece

*Critical Care* 2024, **28(Suppl 1):** P178

**Introduction:** Patients with sepsis may be classified in 4 clinical (α, β, γ, and δ) and 3 immunological phenotypes (macrophage activation-like syndrome-MALS, immunoparalysis, and intermediate), with prognostic implications [1,2]. We aimed to assess the presence of clinical phenotypes and their concordance with immunological phenotypes in patients screened for participation in the PROVIDE study (NCT03332225).

**Methods:** PROVIDE was a personalized immunotherapy trial; 240 patients were screened for immunological phenotypes; those with MALS were further randomized to placebo-controlled anakinra [2]. Clinical phenotypes were determined using a 6-variable algorithm (based on creatinine, lactate, aspartate transaminase, bilirubin, C-reactive protein, International Normalized Ratio) [3]. Differential impact of clinical phenotypes on outcome was assessed in patients non-receiving immunotherapy. The primary outcome was 28-day mortality. Concordance was defined by Cramer’s V ≥ 0.3 (indicating at least moderate association).

**Results:** Clinical phenotype determination was possible in 238 patients. Distribution and overlap with immunological phenotypes is shown in the Figure panel A (α: 34.4%, β: 20.6%, γ: 22.3%, δ: 22.7%). The association between clinical and immunological phenotypes was weak (Cramer’s V = 0.190). Among patients non-receiving immunotherapy (n = 223), clinical phenotyping was predictive of mortality only in the intermediate immunophenotype (n = 91; Log rank 16.91; *p* = 0.001); phenotyping could not be predictive of outcome in MALS (n = 33; Log rank 0.57; *p* = 0.902) and immunoparalysis (n = 99; Log rank 1.260; *p* = 0.739) (Figure panel B).

**Conclusions:** Clinical phenotypes allow better characterisation of intermediate immunophenotype, but offer no additional information to MALS and immunoparalysis.

**References**

1. Seymour CW et al. JAMA 2019;321:2003–17
2. Leventogiannis K, et al. Cell Rep Med 2022;3:100817
3. Karakike E et al. Crit Care 2020;24 (Suppl 2):P589

**Figure (abstract P178):**
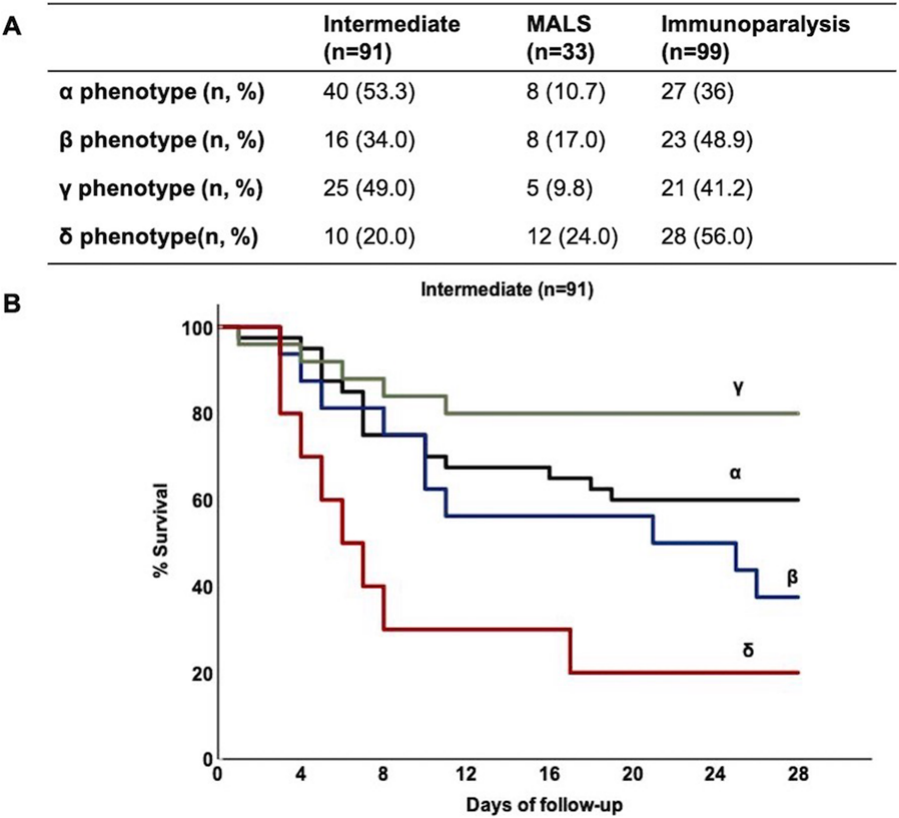
**A** Distribution of clinical phenotypes, according to immunological phenotypes. **B** 28-day survival by clinical phenotype (α, β, γ and δ) among patients with intermediate immune phenotype. MALS: macrophage activation-like syndrome.

## P179 Withdrawn

## P180 Predictors of bloodstream infections in patients undergoing hematopoietic stem-cells transplantation: a prospective cohort study

### I Gur, E Borohovich, A Stern, I Henig, A Miller

#### Rambam Healthcare Campus, Haifa, Israel

*Critical Care* 2024, **28(Suppl 1):** P180

**Introduction:** Early detection of bloodstream-infections (BSI) is particularly challenging in patients undergoing hematopoietic stem-cell transplantation (HSCT) [1]. We strived to develop and comparatively validate a BSI prediction tool in this frail population.

**Methods:** We collected full clinical information on all adult patients hospitalized in our tertiary transplant center following an HSCT from 2010 to 2022. The plethora of data was incorporated into a multivariate prediction model following a three tiered selection process: (1) significantly predicting of BSI on multivariate analysis; (2) hazard proportionality maintained (using scaled Schoenfeld residuals); and (3) no collinearity (r < 0.7) with any other predictors.

**Results:** BSI occurred in 489 (46%) of 1067 patients included. Reduction in total magnesium and inorganic phosphor as well as increase in total bilirubin were independently predictive of BSI (Figure). Combined with total white blood-cells, platelets and eosinophil count, low-density lipoprotein cholesterol and heart-rate increase, we achieved an pseudo-R^2^ = 0.784. Our receiver-operating characteristic area-under-curve was 0.840 (95%CI [0.753–0.927]) versus the previously published artificial intelligence derived BSI prediction models (none of which were validated to HSCT patients specifically) of 0.752 (95%CI [0.700–0.790]) [2] and 0.810 (95%CI [0.800–0.845]) [3, 4].

**Conclusions:** The unique physiology and profound immunosuppression of patients undergoing HSCT may benefit from specially designed BSI predictor models.

**References**

1. Misch EA et al. Infect Dis Clin North Am. 2019;33:399–445
2. Nestor D et al. BMC Infect Dis. 2021;21:316
3. Pai KC et al. J Clin Med. 2021;10:2901
4. Zoabi Y et al. Sci Rep. 2021;11:20101

**Figure (abstract P180):**
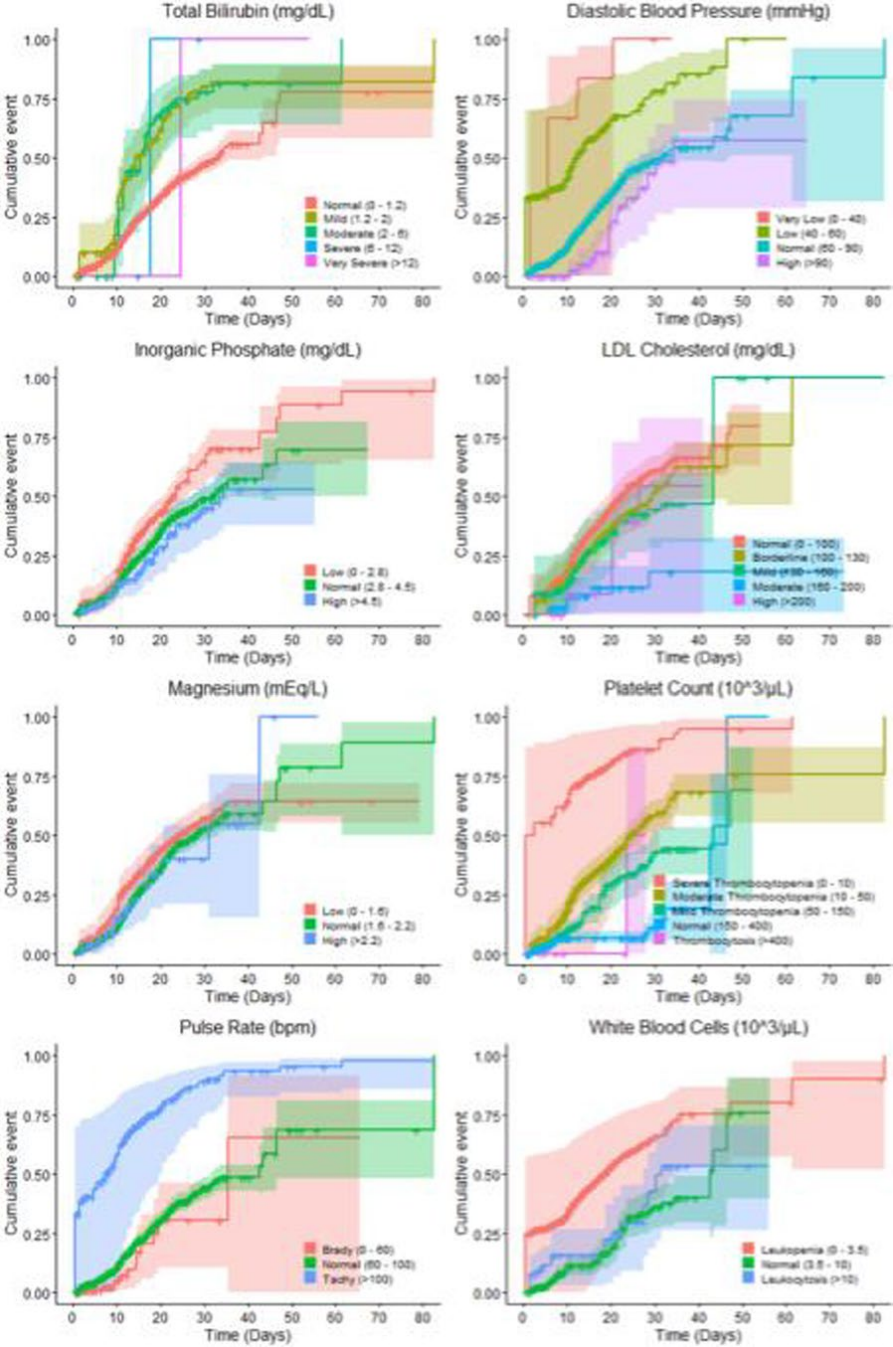
Notable predictors of BSI. The cumulative incidence plots for the variables found significant in the BSI survival analysis are presented.

## P181 Protocolized application of a novel host-response assay in standard sepsis treatment workflows at two different emergency departments

### R Scoggins^1^, H Smithline^2^, TP Aufderheide^3^, M Chinn^3^, T Carver^3^, N Lebedoer^3^, M Sorrells^1^, R Sheybani^1^, H O´Neal^4^

#### ^1^Cytovale Inc., San Francisco, USA, ^2^Baystate Medical Center, Boston, USA, ^3^Medical College of Wisconsin, Miluakee, USA, ^4^LSU Health Science Center/Our Lady of the Lake Regional Medical Center, Baton Rouge, USA

*Critical Care* 2024, **28(Suppl 1):** P181

**Introduction:** Diagnostics to aid the emergency department (ED) in rapid sepsis risk assessment of potentially infected patients are needed [1]. Effective integration into existing workflows will be key for any such test. We evaluated the performance of a host-response test for early sepsis diagnosis implemented in two ED environments as part of the existing protocolized response to suspected infection.

**Methods:** This study enrolled adults (≥ 18) at 2 sites, (Site-1 (S1): Springfield, MA and Site-2 (S2): Milwaukee, WI; Feb.–Jul. 2023). At both sites, ED triage sepsis best practice alerts fired in response to suspicion of infection and ≥ 2 modified SIRS criteria. However, at S1 the test was run on a remnant once the alert fired, whereas at S2, the test was included if ED providers used the sepsis order-set. All blood samples were collected and tested per standard of care within 5 h. The test generates an Index, stratified into 3 interpretation bands (Bands 1–3) of increasing sepsis likelihood [2]. Sepsis status was determined through blinded retrospective physician adjudication.

**Results:** At S1, 189 patients (sepsis prevalence: 12.2%), were stratified as 131 (69.3%) in Band 1, 44 (23.3%) in Band 2, and 14 (7.4%) in Band 3. At S2, 120 patients (sepsis prevalence: 35.0%), were stratified as 39 (32.5%) in Band 1, 42 (35.0%) in Band 2, and 39 (32.5%) in Band 3 (Figure panel A). Differing site sepsis prevalence and operations yielded differences across the Bands. At both sites, the test achieved comparable negative predictive values for Band 1(97.7% and 97.4%) and positive predictive values for Band 3 (71.4% and 59.5%). While the percentage of patients within each Band that received SEP-1 care elements increased across Bands, a similar number of patients received the care metric independent of the Band (Fig panel B).

**Conclusions:** Our findings suggest that this host response test may improve risk stratification and resource utilization despite different ED sepsis protocols.

**References**

1. Paoli CJ et al. Crit Care Med: 2018;46:1889–97
2. US FDA(2022)0.510(k):K220991

**Figure (abstract P181):**
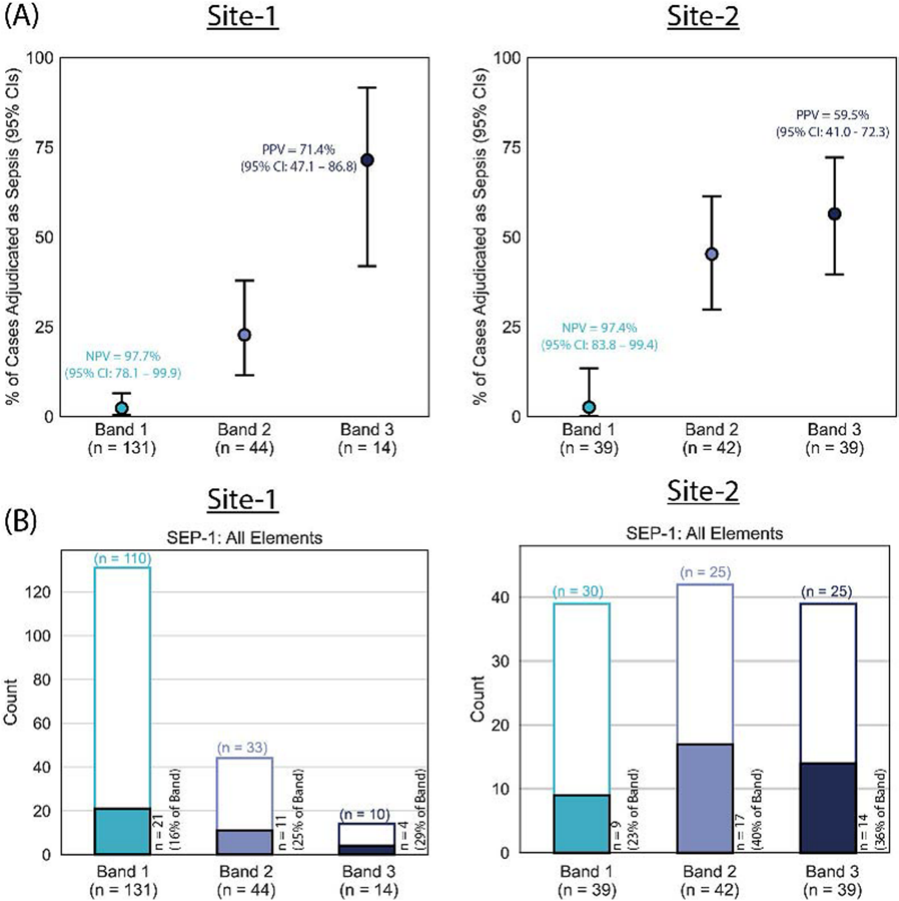
Panel **A** incidence of adjudicated sepsis (per Sepsis-3 definition). Panel **B** administration of all SEP-1 elements (order for blood cultures, order for lactate, administration of antibiotics, all within 3-h), across interpretation bands for the two study sites. Solid bars denote the number of patients in each Band that received the care metric, outlined bars denote the remainder of the patients in each Band that did not receive the care metric.

## P182 Machine learning-based prognostic modelling using a large number of plasma inflammatory mediators in COVID-19 patients

### H Ritter Dal Pizzol^1^, LF Mendes^1^, F Dal-Pizzol^2^, JC Moreira^1^, D Gelain^1^

#### ^1^Universidade Federal do Rio Grade do Sul, Biochemistry, Porto Alegre, Brazil, ^2^Universidade do Extremo Sul Catarinense, Ciências da Saúde, Criciúma, Brazil

*Critical Care* 2024, **28(Suppl 1):** P182

**Introduction:** Since its emergence, COVID-19 has caused major impacts worldwide. A hallmark of the disease is a flawed immune response, characterized by an excessive pro-inflammatory reaction known as "cytokine storm". Gaining a better understanding of the roles that cytokines and others inflammatory mediators play in COVID-19's pathophysiology is essential for identifying valuable prognostic and therapeutic biomarkers, thus we sought to forecast patients' in-hospital mortality and identify the most effective inflammatory protein combinations to make this prediction.

**Methods:** Between June and September 2020, 65 plasma inflammatory mediators were measured in 211 COVID-19 patients within 12 h of admission to the intensive care unit. Patients were divided in development and validation cohorts, and the proteins were grouped in sets of up to three and subjected a machine learning algorithm. Initially, a specific combination of proteins was analysed within the development group using elastic net logistic regression. Subsequently, the results were tested against the validation cohort to evaluate the predictive power of these protein combinations.

**Results:** Bioinformatics models revealed a robust correlation between classic pro-inflammatory cytokines and other proteins associated with immune cell proliferation and activation. Combinations of two or three proteins demonstrated the highest predictive capability for in-hospital mortality, while single proteins displayed considerably lower predictive power (Figure).

**Conclusions:** It is suggested that these proteins serve as prognostic tools for guiding clinical decision-making and treatment strategies for critically ill COVID-19 patients. Furthermore, these findings underscore the idea that an excessively strong pro-inflammatory response is detrimental for a patient`s outcome.

**Figure (abstract P182):**
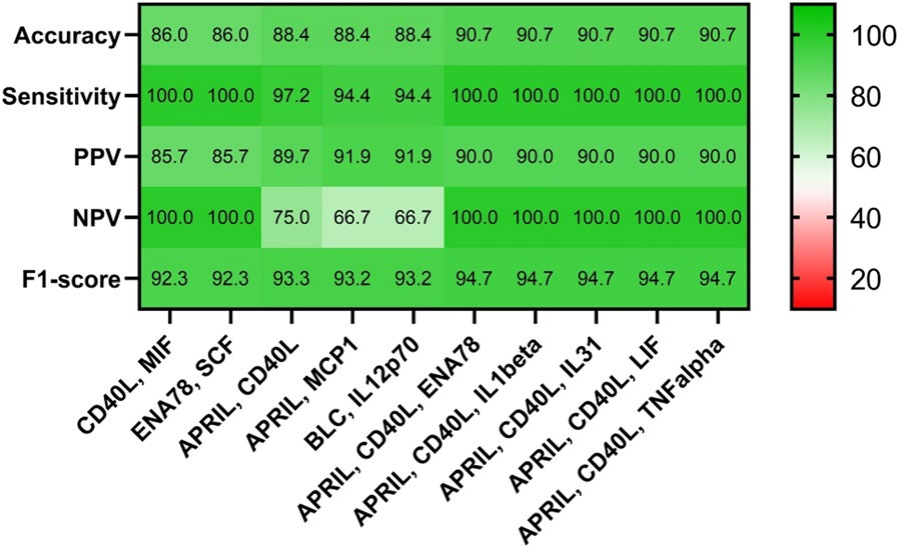
Combination of two and three proteins generate models with high predictive capabilities. Prediction parameters of top 5 models trained with two proteins and top 5 models trained with three proteins, ranked using the arithmetic mean of all presented parameters. Accuracy, sensitivity, positive predictive value, negative predictive value and F1-score are presented in percentage.

## P183 Neutrophil to lymphocyte ratio: prognostic value in the ICU

### F Violini^1^, R Antolini^1^, G Perini^1^, A Raponi^2^, C Pacini^1^, F Santoni^1^, E Vitali^1^, A Salvucci Salice^3^, A Donati^4^, A Carsetti^4^

#### ^1^UNIVPM, Department of Biomedical Sciences and Public Health, Università Politecnica delle Marche, Ancona, Italy, Ancona, Italy, ^2^UNIVPM, Emergency Medicine Residency Program, Università Politecnica delle Marche, Ancona, Italy, Ancona, Italy, ^3^UNIVPM, Medicina e Chirurgia, Ancona, Italy, ^4^UNIVPM, Department of Biomedical Sciences and Public Health, Università Politecnica delle Marche, Ancona, Italy/Clinica di Anestesia e Rianimazione Generale, Respiratoria e del Trauma Maggiore, AOU delle Marche, Ancona, Italy

*Critical Care* 2024, **28(Suppl 1):** P183

**Introduction:** NLR (neutrophil-to-lymphocyte ratio) is defined as the ratio between peripheral blood measured neutrophils and lymphocytes and it defines the relationship between the innate immune response and adaptive immunity. NLR has gained increasing popularity as a marker of systemic inflammation and diseases, as well as a diagnostic [1] and prognostic [2] tool in various medical fields, such as oncology and intensive care. The aim of this study is to evaluate the potential role of the NLR as a prognostic factor in the ICU.

**Methods:** 208 patients admitted to our university hospital ICU between January 2023 and June 2023 were retrospectively enrolled for the purpose of this study, regardless of the admitting diagnosis. We collected data on demographic characteristics, length of stay, laboratory results, infectious agents and ICU mortality. SAPSII and APACHEII scores were calculated at the time of ICU admission. For each patient receiving antibiotic therapy, we collected data regarding blood count, duration of therapy and infectious agent involved (multi drug resistant (MDR) vs. non-MDR), then we calculated the NLR at the time of the first antibiotic prescription. Lastly, we compared NLR values between different subgroups of patients: MDR infected and non-MDR infected (t-test), septic shock and other diagnoses (Mann–Whitney-U test), survivors and non-survivors (Mann–Whitney-U test).

**Results:** The study population was composed by 140 males and 68 females, the median age was 64 years[49; 77]. The overall mean APACHE II score was 14.43 (± 7.63), SAPS II score 44.60 (± 16.17) and median length of stay was 7 days [2; 16.5]. Significant difference was found between survivors and non-survivors NLR (*p* = 0.01), whereas no significant differences were found evaluating other subgroups (*p* > 0.05) [Table].

**Conclusions:** In ICU admitted patients, NLR represent a valid prognostic factor for increased mortality risk, whereas it is not useful to detect MDR infected or septic patients.

**References**

1. Zhou YQ et al. Ther Clin Risk Manag. 2018;14:1863–1869
2. Song M et al. Sci Rep. 2021;11:464

**Table (abstract P183):** Statistical analysis of NLR difference between various patients subgroups.

Subgroups	Significance value
NLR in MDR infected patients 20 (± 16.36) NLR in other	Comparison t-test: *p* > 0.05
NLR in septic shock 34.69 (± 29.58) NLR in other	Comparison t-test: *p* > 0.05
NLR in survivors 11.11 [6.33; 17.35] NLR in non-survivors	Comparison Mann–Whitney-U: * p* = 0.01

## P184 Circulating levels of IL-6 receptor and response to tocilizumab treatment in critical COVID-19

### G Marinakis^1^, A Siampanos^2^, E Karakike^2^, M Patrani^1^, E Giamarellos-Bourboulis^2^

#### ^1^Korgialeneion Benakeion General Hospital, Intensive Care Unit, Athens, Greece, ^2^National and Kapodistrian University of Athens, 4th Department of Internal Medicine, Medical School, Athens, Greece

*Critical Care* 2024, **28(Suppl 1):** P184

**Introduction:** Tocilizumab (TCZ) is an antagonist of the interleukin (IL)-6 receptor included in the treatment algorithm of critical COVID-19. We investigated if circulating concentrations of soluble IL-6 receptor, namely CD126, may be prognostic of the 28-day outcome.

**Methods:** Serum samples and clinical data were prospectively collected from 134 patients under mechanical ventilation for SARS-CoV-2 infection. All patients were treated with TCZ and blood sampling was done before start of treatment. Concentrations of IL-6, CD126 and glycoprotein (gp)130, one molecule of intracellular IL-6 signaling, were measured by an enzyme immunosorbent assay. The study endpoint was the association between circulating levels and the persistence of severe COVID-19 (defined as WHO clinical progression scale-CPS 6 or more) by day 28.

**Results:** Concentrations of CD126, but not of IL-6 and gp130, were higher among patients with persisting severe COVID-19 by day 28 (Figure). Fifty-nine and 68 patients had CD126 more than 100 ng/mL or less than 100 ng/mL at baseline; 69.5% and 50% were at WHO-CPS 6 or more by day 28 (odds ratio 2.27; 1.09–.73; *p* = 0.031).

**Conclusions:** The likelihood to response to TCZ may be guided by baseline levels of CD126. This may frame the start of precision immunotherapy in sepsis with TCZ.

**Figure (abstract P184):**
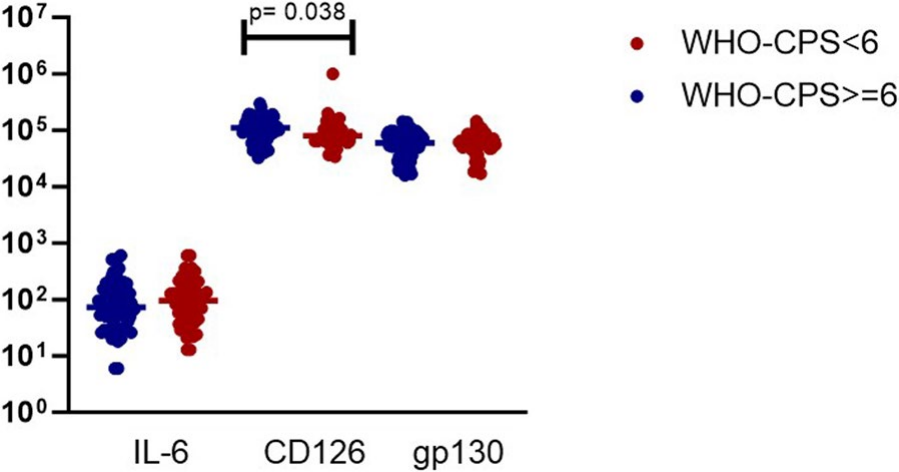
Baseline circulating levels of mediators involved in the IL-6 cascade and 28-day outcome.

## P185 Postoperative IL-6 improves early prediction of infection after pulmonary cancer surgery, a double-center prospective study

### T Reniers^1^, PG Noordzij^1^, E Veen^2^, L Vernooij^1^, FN Hofman^3^, TCD Rettig.^4^

#### ^1^Sint Antonius Hospital, Anesthesiology and Intensive Care, Nieuwegein, Netherlands, ^2^Amphia Hospital, Surgery, Breda, Netherlands, ^3^Sint Antonius Hospital, Cardiothoracic surgery, Nieuwegein, Netherlands, ^4^Amphia Hospital, Anesthesiology and Intensive Care, Breda, Netherlands

*Critical Care* 2024, **28(Suppl 1):** P185

**Introduction:** Approximately 20% of patients undergoing pulmonary surgery suffer from a postoperative infection. Early prediction could improve prevention and management of postoperative infections. Postoperative hyperinflammation increases infection risk. We hypothesized that the inflammatory biomarker IL-6 is an early predictor for postoperative infection.

**Methods:** A double-center prospective cohort study in patients undergoing pulmonary cancer surgery. IL-6 and C-reactive protein (CRP) concentrations were determined after induction of anesthesia, 6, 9, 12, 24, 48 and 72 h postoperatively. The primary outcome was any postoperative infection within 30 days of surgery. Multivariable logistic regression was used to create a basic prediction model including age, sex, surgery duration and Charlson Comorbidity Index. Peak IL-6 concentrations within 24 h of surgery were added to this basic model and compared to a model with peak CRP. C-statistics were calculated to assess the predictive accuracy of IL-6 and CRP.

**Results:** 170 patients were included, of whom 38 (22%) developed a postoperative infection. IL-6 concentrations peaked 6 h after surgery in 84% of patients (Figure panel A). CRP concentrations peaked 24 h after surgery in 88% of patients (Figure panel B). Peak IL-6 was associated with postoperative infection (adjusted odds ratio (aOR) 1.04 per 10 pg/mL increase, 95% confidence interval (CI) 1.00–1.09, *p* < 0.05) as was peak CRP (aOR 1.01, 95% CI 1.00–1.03, *p* = 0.03). Added to the basic prediction model, c-statistics including peak IL-6- or peak CRP concentrations were 0.67 (95% CI 0.56–0.77) and 0.68 (95% CI 0.57–0.77), respectively.

**Conclusions:** Peak IL-6 is an earlier predictor of postoperative infection compared to CRP, creating a window of opportunity to prevent infection.

**Figure (abstract P185):**
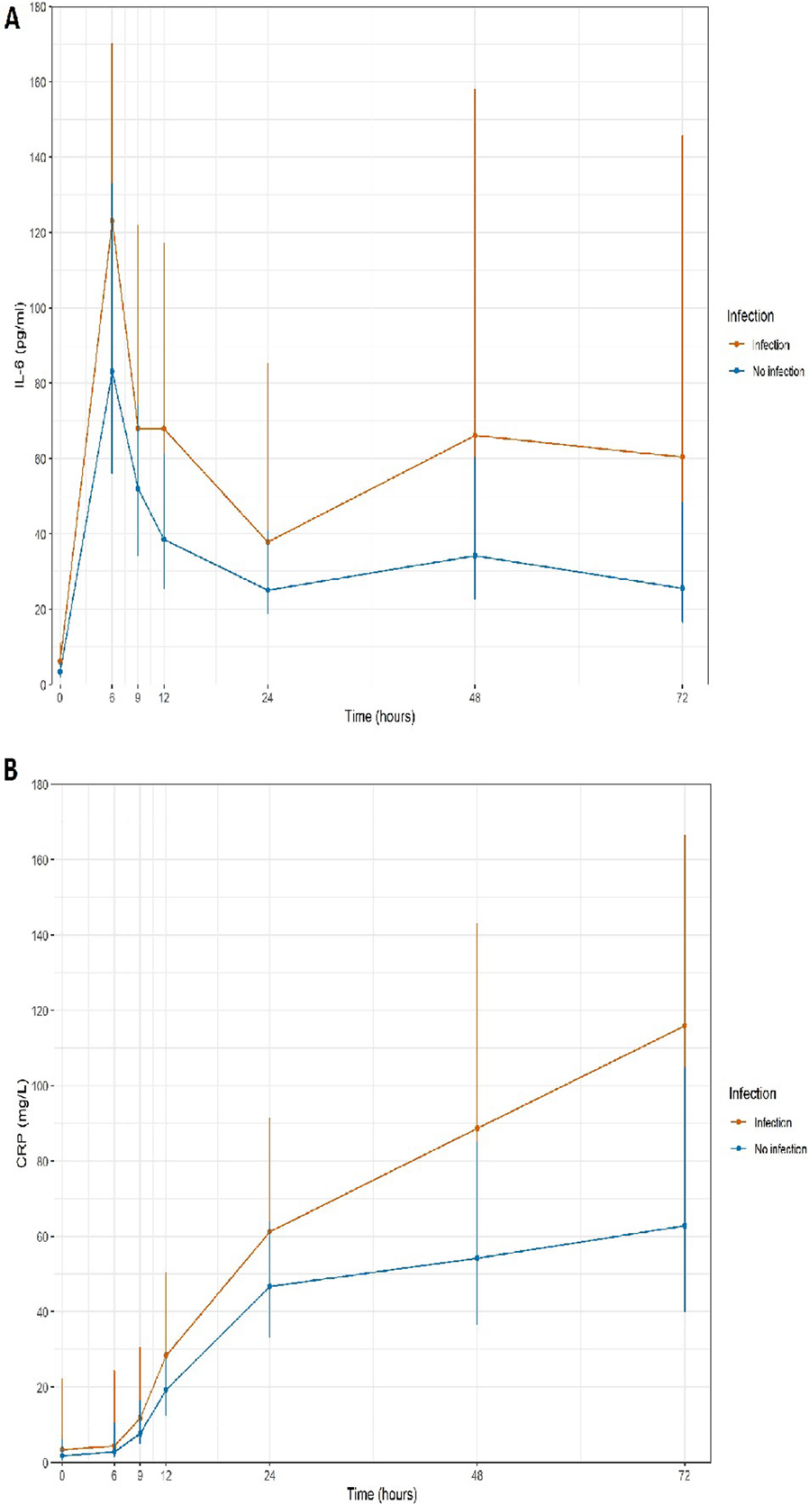
Perioperative concentrations of interleukin 6 (IL-6) (panel **A**) and C-reactive protein (CRP) (panel **B**) in patients with and without infection (median and inter quartile range).

## P186 Impact of COVID-19 omicron variant on lung transplantation patients: a single tertiary medical center experience

### KW Chang, SW Leu, HC Hu, KC Kao

#### Chang Gung Memorial Hospital, Taoyuan, Taiwan, Department of Thoracic Medicine, Taoyuan, Taiwan, Republic of China

*Critical Care* 2024, **28(Suppl 1):** P186

**Introduction:** Lung transplantation patients with coronavirus disease 2019 (COVID-19) have high mortality. However, the impact of the Omicron variant in lung transplantation patients is unclear. This study focuses on lung transplant patients diagnosed with COVID-19 during the Omicron wave, and investigate the clinical presentations, outcomes and pulmonary function.

**Methods:** In this single-center retrospective study, we enrolled lung transplantation patients diagnosed with COVID-19 from January 2022 to December 2022. Demographic, laboratory and pulmonary function data were recorded. Pre-COVID-19 pulmonary function data were obtained from the closest routine test, and post-COVID-19 tests were arranged after release from quarantine.

**Results:** Of the 22 enrolled patients, four were admitted to general wards for treatment and observation, and two needed oxygen support. All of the patients recovered from COVID-19. There were no significant decreases in forced vital capacity (FVC) (2.3 ± 0.6 vs. 2.2 ± 0.8 L, *p* = 0.363) and forced expiratory volume in the first second (FEV1) (2.0 ± 0.6 vs. 1.9 ± 0.8 L, *p* = 0.269) after COVID-19 infection. Daily prednisolone dose and serum tacrolimus levels were significantly correlated to the duration of positive COVID-19 test and change in FEV1. Patients who were not fully vaccinated or did not receive a booster dose had a significantly longer positive test duration (9.1 ± 4.1 vs. 26.6 ± 24.4 days, *p* = 0.05), and greater decrease in FEV1 (23.6 ± 137.3 vs. − 331.8 ± 537.8 L, *p* = 0.040).

**Conclusions: **In this study, lung transplant patients infected with COVID-19 during the Omicron wave did not have severe disease and the mortality rate was low. Immunosuppressive medications and COVID-19 vaccination were correlated with viral clearance and pulmonary function after COVID-19 infection.

## P187 Retrospective evaluation of the relationship between infections and mortality during the ICU hospitalisation of polytrauma patients

### O Ayvaz, S Hosaf, T Kayim, E Karakoc, B Yelken

#### Eskişehir Osmangazi University, Anaesthesiology and Reanimation, Eskisehir, Turkey

*Critical Care* 2024, **28(Suppl 1):** P187

**Introduction:** Polytrauma accounts for 9% of the causes of death worldwide [1]. Understanding the causes of mortality in trauma patients guides patient management [2]. The aim of this study is to determine the relationship of infections and mortality among for polytrauma patients that had been treated in intensive care unit (ICU).

**Methods:** The study was conducted by examining the records of 78 polytrauma patients between the ages of 18 and 85 who had been treated in ICU at a 3rd level university hospital between June 2020 and June 2023 retrospectively. Demographical parameters, mortality, mechanical ventilation, GKS, SOFA, APACHE-II, CRP and procalcitonin levels were analyzed using Mann Whitney U-Test was used for comparisons between groups, and Chi-Square test was used for comparisons of categorical variables. Spearman's Correlation Analysis was used to examine the relationship between continuous variables.

**Results:** Mortality ratio among polytrauma patients was 17.8% (n:14). There was statistically significant difference in terms of GCS, SOFA, APACHE-II and the level of procalcitonin at 6.th day between survivors and non-survivors. The first 7-day levels of CRP were similar between survivors and nonsurvivors. Respiratory tract culture and blood culture positivity had a correlation with mortality. The SOFA scores of the nonsurvivors recorded at the day of the diagnosis of the infection were significantly higher.

**Conclusions:** As a result of this study, it was concluded while CRP levels are high due to trauma and do not predict mortality, procalcitonin levels can be used to predict the presence of infections and relationship between infections and mortality. In order to develop appropriate prescriptions regarding empirical antibiotic selections, the study result that blood culture and respiratory tract culture positivity is associated with mortality should be taken into consideration.

**References**

1. Van Breugel JM et al. World J Emerg Surg. 2020;15:1–13
2. Iyengar KP et al. Orthop Res Rev. 2023;15:27–38

## P188 Using positive reinforcement to improve prescribing behaviours at a large teaching hospital in the UK

### M Saba^1^, E Thomas^1^, R Gill^1^, M Arshad^1^, E Plunkett^2^, JM Patel^1^

#### ^1^Queen Elizabeth Hospital, Intensive Care Unit, Birmingham, UK, ^2^University Hospitals Birmingham, Anaesthetics, Birmingham, UK

*Critical Care* 2024, **28(Suppl 1):** P188

**Introduction:** Antimicrobials are administered to 70% of patients on Intensive Care Units (ICU), despite less than half having a confirmed infection [1]. Robust antimicrobial stewardship is crucial to tackle resistance. Guidelines recommend antimicrobial prescriptions include the duration and indication [2]. We aimed to use positive re-enforcement to improve adherence to antimicrobial prescribing within our ICU.

**Methods:** Baseline data on antimicrobial prescriptions was collected in December 2022 at a 100-bed ICU in the UK. Between February and April 2023, 8 random weeks were selected to review prescriptions and certificates were sent to those prescribers who had met all the required standards. This was repeated in August 2023 to coincide with rotation of the medical work force. Data collected included the number of patients on antibiotics and the number of prescriptions that met the required standards.

**Results:** A total of 335 patients were on antimicrobials in the first phase of this study. Baseline data demonstrated that only 29% (n = 10) of the prescriptions met the required standard. Over the following 8-weeks there was a significant improvement in prescribing to 70% by week 7 (n = 18). In total 127 certificates were issued during this time. In the second, phase 322 patients were on antimicrobials. Baseline data was similar at 29% (n = 12). Again, as the study progressed and positive re-enforcement was implemented, an increase in compliance was observed with a peak of 78% (n = 18) in week 6, (Figure).

**Conclusions:** This study has demonstrated that the use of positive re-enforcement can significantly improve prescribing habits and that a sustained change can be achieved. We propose that positive feedback should be used more widely in critical care to implement behavioural changes and postulate that these may improve patient care.

**References**

1. Pandolfo et al. BMJ. 2022;31:199–210
2. NICE. Antimicrobial Stewardship. (QS121); P10. 2016.

**Figure (abstract P188):**
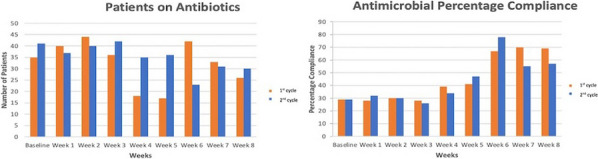
Bar chart comparison of antimicrobial prescribing compliance in audit cycle 1 and 2.

## P189 Clinical infections and microbiological characteristics in patients admitted to ICUs after the earthquake in the southeastern part of Türkiye in 2023

### EK Kaya^1^, B Halacli^1^, G Guven^1^, M Yildirim^2^, E Gemcioglu^3^, B Erdemir Sullu^4^, RC Yuksel^5^, A Esmaoglu Coruh^6^, K Gundogan^5^, A Topeli^1^

#### ^1^Hacettepe University Faculty of Medicine, Department of Internal Medicine/Division of Intensive Care Medicine, Ankara, Turkey, ^2^Ministry of Health, Etlik City Hospital, Department of Internal Medicine/Intensive Care Unit, Ankara, Turkey, ^3^Ministry of Health, Etlik City Hospital, Department of Internal Medicine, Ankara, Turkey, ^4^Hacettepe University Faculty of Medicine, Division of Intensive Care Medicine, Ankara, Turkey, ^5^Erciyes University Faculty of Medicine, Department of Internal Medicine/Division of Intensive Care Medicine, Kayseri, Turkey, ^6^Erciyes University Faculty of Medicine, Department of Anesthesiology, Kayseri, Turkey

*Critical Care* 2024, **28(Suppl 1):** P189

**Introduction:** The aims were to determine clinical infections, and microbiological characteristics in patients admitted to the ICUs after the earthquake in the southeastern part of Türkiye in February 2023, to determine antibiotic susceptibility patterns, appropriateness of antibiotic use, patient outcomes, and to identify factors influencing development of microbiologically proven clinical infections.

**Methods:** This is a retrospective, multi-center, observational study conducted on adult critically-ill earthquake victims in 6 ICUs of 3 tertiary referral hospitals. Patients were stratified into groups based on culture positivity timing on a 72-h breakpoint and factors influencing infections were analyzed.

**Results:** 107 adult earthquake victims (58 females, median age 37) were included. Infection was present in 50.5% of the patients, predominantly with multidrug-resistant pathogens, including *Acinetobacter baumannii* and *Klebsiella pneumonia.* The ICU mortality was observed in 9.3% of patients. Regarding the first isolated microorganism, appropriate antibiotic therapy was initiated in only 11.7% of patients within the first 72 h of ICU admission. The ROC curve for the development of infection demonstrated that time stuck under the rubble ≥ 11.5 h had an AUC 0.64 [CI 0.53–0.75] (*p* = 0.019). Multivariate logistic regression revealed that amputation (OR 5.30 [1.03–27.36], *p* = 0.046) and intermittent hemodialysis before ICU admission (OR 2.98 [1.04–8.52], *p* = 0.043) were found to be independent factors predicting infection.

**Conclusions:** Half of the patients admitted to the ICUs following the earthquake had microbiologically proven clinical infections. MDR pathogens were isolated even in the early stages of ICU admission with a high rate of inappropriate antibiotic treatment. There is a high likelihood of infection among patients who have been stuck under rubble for more than 12 h, and who have undergone invasive procedures like amputation, and hemodialysis.

## P190 Continuation of concomitant antibiotics is associated with worse outcomes in patients with *Clostridioides difficile* infectious disease

### D Adukauskienė^1^, R Mickus^1^, A Adukauskaitė^2^

#### ^1^Medical Academy, Lithuanian University of Health Sciences, Medical Academy, Lithuanian University of Health Sciences, Kaunas, Lithuania, ^2^Department of Cardiology and Angiology, University Hospital of Innsbruck, Department of Cardiology and Angiology, University Hospital of Innsbruck, Innsbruck, Austria

*Critical Care* 2024, **28(Suppl 1):** P190

**Introduction:** During the episode of *Clostridioides difficile* (*C. difficile*) infectious disease (CDID) antibiotics may need to be used concomitantly for treatment of infection other than CDID [1]. The aim of this study was to evaluate the impact of the continuation of concomitant antibiotics (cAB) on outcomes in CDID patients (pt) when the CDID treatment was concordant with Guidelines.

**Methods:** A single-center, retrospective 10-yr cohort study was conducted in the largest university-affiliated hospital in Lithuania. Pt with primary episodes of CDID (diarrhea and positive stool test for *C. difficile* toxin A/B) in the period of 2011–2020 were included. cAB was defined as AB used concomitantly for treatment of other infection than CDID ≥ 3 days after CDID diagnosis. The impact of continuation of cAB on 30-day all-cause mortality and 30-day survival has been estimated. *IBM SPSS 23.0,* binary logistic regression analysis*, *Kaplan–Meier model, log-rank, Breslow, Tarone-Ware, and Pearson’s χ^2^ tests were used for statistics, level of significance—*p* < 0.05.

**Results:** The treatment was concordant with Guidelines in 224 cases out of study total of 370 pt. In CDID pt the treatment with cAB was estimated as a predictor for 30-day all-cause mortality (OR 0.3; 95% CI 0.1–0.9; *p* = 0.047). The 30-day follow-up included 53/224 pt. The 30-day all-cause mortality was n = 13/25 (52.0%) in CDID pt with cAB, as compared with n = 7/28 (25.0%) in cases with their discontinuation, *p* = 0.053. The 30-day survival decreased (*p* < 0.05) in CDID pt treated with cAB (Figure).

**Conclusions:** The use of concomitant antibiotics in CDID patients is associated with worse outcomes even in cases of CDID treatment concordant with Guidelines; therefore, this antibiotic therapy always should be justified, de-escalated, or ceased.

**Reference**

1. McDonald LC et al. Clin Infect Dis. 2018;66:1–48

**Figure (abstract P190):**
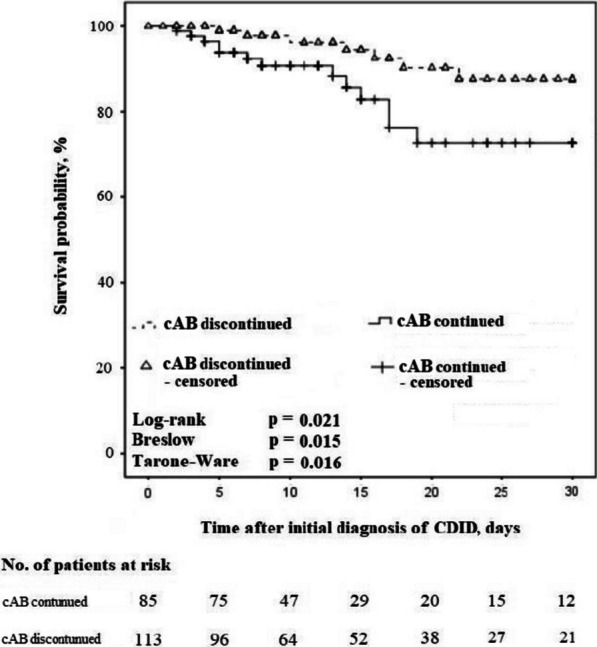
The 30-day survival in CDID pt treated with cAB.

## P191 Antibiotic resistance cluster analysis in critical patients

### AO Oliveira^1^, AM Mestre^1^, AG Gonçalves^1^, MVC Vera-Cruz^1^, LP Pássaro^2^, JGP Gonçalves-Pereira^1^

#### ^1^Hospital de Vila Franca de Xira, ICU, Vila Franca de Xira, Portugal, ^2^Hospital de Vila Franca de Xira, Infectious Disease Department, Vila Franca de Xira, Portugal

*Critical Care* 2024, **28(Suppl 1):** P191

**Introduction:** Infection is one of the major concerns in the intensive care unit (ICU) and the main reason for several admissions. Sepsis and septic shock are associated with significant morbidity and mortality and pose great pressure on the ICU and the entire health system. Studies have shown a close relationship between infection, hospital length of stay, and mortality.

**Methods:** Retrospective cohort study including all patients admitted to a multipurpose ICU between 2015 and 2019. Demographic and clinical data were collected as microbiological isolates, along with their antibiotic resistance profile. A two-step cluster analysis was performed to identify patterns.

**Results:** We evaluate 2085 admissions of which 820 have a microbiological isolate. As much as 55 different bacteria were found. Male gender was predominant (61.9%). The mean age and mean SAPSII Score were 67.4 ± 13.6 years and 50.4 ± 18.9 respectively. The median ICU length of stay was 4.2 days (IQR 5.83). ICU and In-hospital mortality were 20.8% and 34.3%, respectively. It was possible to segregate the sample into 3 different clusters. The major predictors for each cluster were resistance to common antibiotics: Cluster 1 (*Amoxicillin* resistant); Cluster 2 (multi-sensible) and Cluster 3 (Quinolone and Penicillin resistance). Both ICU LOS and time of mechanical ventilation were statistically significative higher in Cluster 3. Clusters 1 and 2 were similar in our model. This classification was not helpful to identify different patterns of risk for ICU and Hospital mortality (Table).

**Conclusions:** Cluster analysis techniques are a new and powerful method to analyze and discriminate different groups within large samples. In this exploratory analysis, we developed a model with good internal coherence. This was able to point to differences in patients' profiles according to isolated bacteria antibiotic resistance patterns. Accordingly, we were able to identify a sub-group with higher ICU LOS and time on mechanical ventilation, although without different mortality risks.

**Table (abstract P191):** Sample characteristics

Variable | Sample	Cluster 1	Cluster2	Cluster3	*p* value
N | (746)	248 (33.2%)	441 (59.1%)	57 (7.6%)	
ICU-LOS (mean) | 6.58 ± 6.8	6.23 ± 6.4	6.35 ± 6.3	9.85 ± 9.9	0.001†
SAPS II Score (mean) | 50.38 ± 18.9	49.4 ± 18.6	50.8 ± 19.3	51.3 ± 17.2	0.588†
Ventilation days. (mean) | 6.33 ± 6.6	5.92 ± 6.5	6.16 ± 6.2	9.47 ± 8.9	0.001†
Age (mean) | 67.45 ± 13.6	67.13 ± 13	67.78 ± 13.7	66.2 ± 15.1	0.643†
ICU mortality (%) | 20.8	20.2	21.3	19.3	0.9◊
In-Hosp. mortality (%) | 34.3	36.7	32.7	36.8	0.516◊

LOS - length of stay. SAPS II - Simplified Acute Physiology Score II. Statistical comparison between cluster variances (†- ANOVA test; ◊ Chi square test)

## P192 Identification and comparison of the lower respiratory tract microbiome composition by conventional culture and 16 s rRNA gene sequencing in bronchoalveolar lavage from intubated intensive care patients with and without ventilation associated pneumoniae

### IG Bustos^1^, LM Mendez^1^, JC Olivella^1^, AE Viñan Garces^1^, G Guerrón-Gómez^1^, ED Ibáñez-Prada^1^, CC Serrano-Mayorga^1^, I Martin-Loeches^2^, A Rodriguez^3^, LF Reyes^1^

#### ^1^Facultad de Medicina, Universidad de La Sabana, Bogota, Colombia, ^2^Multidisciplinary Intensive Care Research Organization (MICRO), Department of Intensive Care Medicine, St. James´s Hospital, Dublin, Ireland, ^3^Critical Care Department, URV/IISPV/CIBERES, Hospital Universitari Joan XXIII, Tarragona, Spain

*Critical Care* 2024, **28(Suppl 1):** P192

**Introduction:** Ventilator-associated pneumonia (VAP) is usually caused by bacterial pathogens with high resistance rates; consequently, we propose two methods for describing and analyzing lower respiratory tract Microbiome composition dynamics. Identifying the etiological microorganisms (MOs) during VAP and their relationship with the lung microbiome is crucial for optimizing antibiotic treatment to improve antimicrobial usage.

**Methods:** This prospective cohort study was conducted at Clinica Universidad de La Sabana in Colombia from January 2020 to July 2022. In the intensive care unit (ICU), ventilated patients were enrolled within 12 h of hospital admission. Bronchoalveolar lavage samples were collected using a standardized protocol. Specimens were obtained at ICU admission and 72 h after admission. MOs were classified by species through Conventional Culture (CC) and by Operational Taxonomic Units (OTUs) using 16S ribosomal RNA (rRNA) gene sequencing. Statistical analysis was performed using R studio.

**Results:** A total of 80 patients were included; 51% (41/80) developed VAP. Culture/sequencing results and characterization are shown in the Figure. It displays the MOs and OTUs identified and their distribution. Initial results showed that 88% of MO in conventional culture were Gram-positive, 7.5% were Gram-negative. In contrast, in 16 s rRNA sequencing, 35.5% were Gram-positive OTUs, and 64.5% were Gram-negative. Comparing MOs abundance in patients with and without VAP, significant differences were found for *S. aureus* (23.5% vs. 42.3% *p* < 0.01). Overall OTUs concentrations showed no significant difference between patients without VAP vs. VAP. Gram-negative OTUs were predominately present in both patient groups.

**Conclusions:** Conventional culture often yields false negatives for anaerobes in respiratory samples, but genomic sequencing identifies that Gram-negative OTUs were frequently present. Genomic sequencing offers valuable insights into pneumonia-causing agents and could be helpful in clinical practice.

**Figure (abstract P192):**
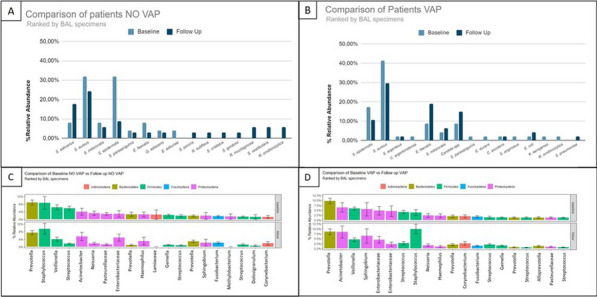
**A** Percentage of relative abundance by conventional culture during baseline and follow-up for NO VAP patients; **B** Percentage of relative abundance by conventional culture during baseline and follow-up for VAP patients; **C** Percentage of relative abundance by 16S rRNA sequencing during baseline and follow-up for NO VAP patients; **D** Percentage of relative abundance by 16S rRNA sequencing during baseline and follow-up for VAP patients.

## P193 Characteristics and risk factors in intensive care unit patients with bacteremia and pneumonia caused by carbapenem-resistant *Klebsiella pneumoniae*

### BC Wu, MY Teo, KW Chang, LC Chiu, SW Leu, KC Kao, HC Hu

#### Chang Gung Memorial Hospital, Thoracic Medicine, Taoyuan City, Taiwan, Republic of China

*Critical Care* 2024, **28(Suppl 1):** P193

**Introduction:** Infections caused by carbapenem-resistant *Klebsiella pneumoniae* (CRKP) have emerged as a serious threat to the lives of patients in intensive care units (ICUs) [1]. We aimed to identify predictive factors for ICU patients with CRKP-induced bacteremia and pneumonia to enhance treatment and prognosis.

**Methods:** We conducted a retrospective study involving data from ICU-treated patients with CRKP-induced bacteremia and pneumonia at Chang Gung Memorial Hospital, Linkou branch from January 2017 to December 2021. Clinical characteristics, laboratory data, as well as treatment and outcome information were collected. Predictive factors were analyzed using statistical methods to determine their association with outcomes.

**Results:** A total of 161 patients were included in the study. Thirty-day mortality was reported for 105 patients (65%). Most CRKP clinical isolates were carbapenemase producers (132/161; 81.9%), of which *K. pneumoniae* carbapenemase (KPC)-producing isolates were most prevalent (112/132; 84.8%). Cox regression analysis revealed that a ceftazidime-avibactam-containing antibiotic regimen (hazard ratio (HR) 0.22, confidence interval (CI) 95% 0.10–0.50, *p* < 0.001) (Figure) and the use of active antibiotics within 48 h (HR 0.47, CI 95% 0.26–0.85, *p* = 0.013) were associated with a favorable outcome, while a high sequential organ failure assessment (SOFA) score (HR 1.24, CI 95% 1.15–1.35, *p* < 0.001) was associated with death.

**Conclusions:** The use of a definitive antibiotic within 48 h and a ceftazidime-avibactam-containing antibiotic regimen had a better 30-day mortality outcome. Early detection of the pathogen and specific isolates is important in clinical practice and can improve mortality.

**Reference**

- Zhu XY et al. Chin Med J (Engl). 2021;134:1735–1737.

**Figure (abstract P193):**
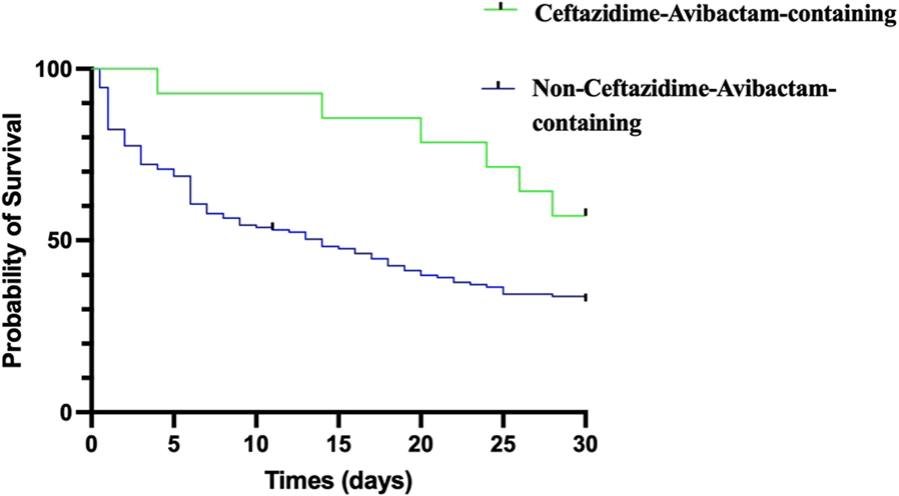
Kaplan–Meier curves for survival of patients with ceftazidime-avibactam-containing and non ceftazidime-avibactam-containing antibiotic.

## P194 Predictors of mortality in intensive care unit respiratory failure patients with blood stream infection caused by *Klebsiella pneumoniae* carbapenemase-producing *Klebsiella pneumoniae*

### MY Teo, BC Wu, KW Chang, LC Chiu, SW Leu, KC Kao, HC Hu

#### Chang Gung Memorial Hospital, Department of Thoracic Medicine, Taoyuan City, Taiwan, Republic of China

*Critical Care* 2024, **28(Suppl 1):** P194

**Introduction:** The *Klebsiella pneumoniae* carbapenemase-producing Kp (KPC-Kp) strain infection is gradually emerging as a significant problem contributing to the mortality of intensive care unit (ICU) patients, especially blood stream infection (BSI). The aim of this study was to identify risk factors associated with mortality in ICU patients with BSI caused by KPC-Kp.

**Methods:** All data from patients who suffered from BSI caused by KPC-Kp in ICU from January 2017 to December 2021 were retrospective analyzed in Chang Gung Memorial Hospital, Taoyuan, Taiwan. All patients simultaneously suffered with respiratory failure and were all received mechanical ventilation. We analyzed outcome of BSI with KPC-Kp in ICU patients.

**Results:** A total of 168 patients were identified during the study period. The 30 days mortality rate was 61.9%. Higher Pitt bacteremia score, SOFA score and Charlson morbidity score were found in our patients. The most frequent infection source of BSI was pneumonia (112, 66.7%). The result of multivariate analysis showed ceftazidime-avibactam (CAZ-AVI) based regimen (hazard ratio (HR) 0.312, 95% confidence interval (CI) 0.151–0.648; *p* < 0.002) and appropriate antibiotic treatment within 48 h after KPC-Kp BSI onset (HR 0.623; 95% CI 0.408–0.950; *p* < 0.028) were independently associated with favorable outcomes (Figure).

**Conclusions:** We found that appropriate antibiotic treated within 48 h after KPC-Kp blood stream infection onset and CAZ-AVI treated were crucial factor in favorable outcome among patients with respiratory failure in ICU. We recommend the utilize of novel rapid molecular test in high risk of hospital acquired KPC-Kp infections patients [1]. Subsequently, it is advisable to treat with effective antibiotics such as CAZ-AVI as soon as possible.

**Reference**

1. Satlin MJ et al. Clin Infect Dis. 2022;75:2066–2075.

**Figure (abstract P194):**
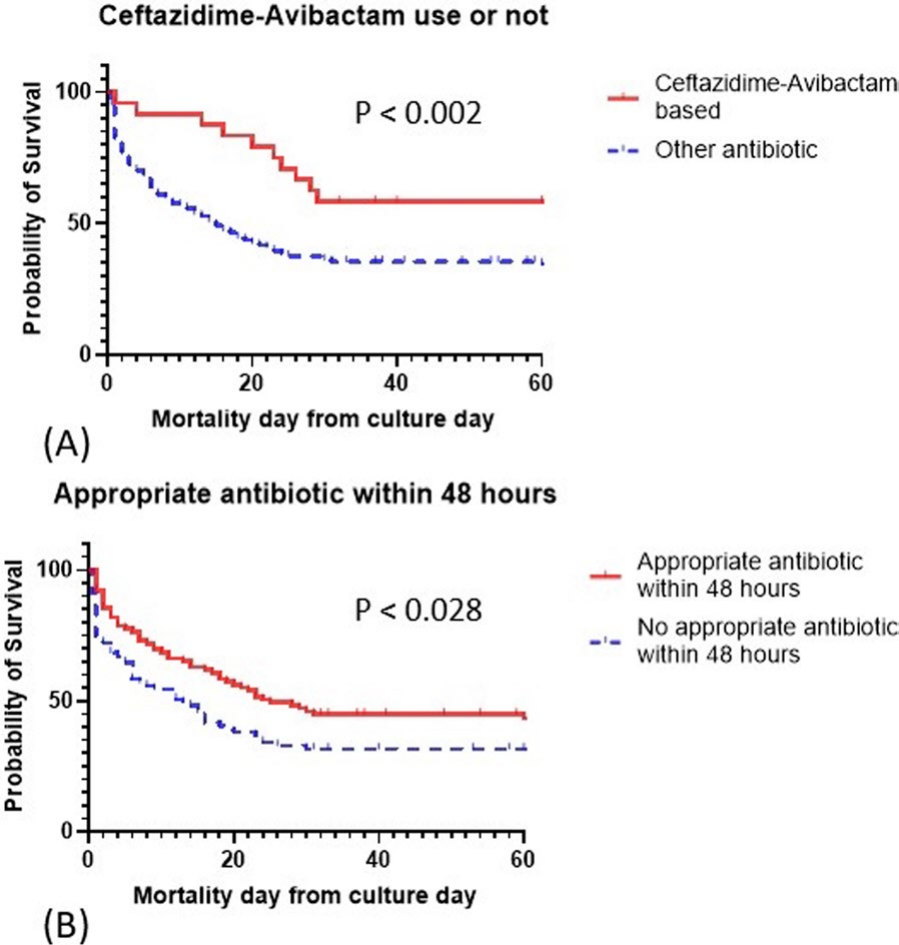
Kaplan–Meier curves for survival of patient with ceftazidime-avibactam based regimen treated or not (**A**); Appropriate antibiotic treated within 48 h or not (**B**).

## P195 Carbapenemase producing *Acinetobacter junii*: outbreak in a bronchoscopy operation theatre in General University Hospital in Prague (GUH)

### G Kroneislová, J Závora, V Adámková

#### General University Hospital in Prague, Clinical Microbiology and ATB Center, Prague 2, Czech Republic

*Critical Care* 2024, **28(Suppl 1):** P195

**Introduction:** *Acinetobacter* spp. is one of the pathogens causing hospital-acquired infections. Nowadays, the number of carbapenem-resistant strains is increasing. Colonization and infections caused by these strains can become a problem of our time.

**Methods:** If *Acinetobacter* spp. is detected in these materials in GUH, susceptibility to selected antibiotics (co-trimoxazole, ciprofloxacin, gentamicin, amikacin, imipenem and meropenem) is routinely tested by disc diffusion method. If the strain is resistant to carbapenems, carbapenemase production is determined using rapid immunochromatographic test (CARBA-5) and MIC of colistin is determined.

**Results:** Between 12/2022 and 5/2023, (VIM-type carbapenemase-producing) *Acinetobacter* spp. was detected in 34 unique BAL or aspirate samples. The strains were identified by MALDI-TOF (mass spectrometry) as *Acinetobacter junii.* All these strains were susceptible only to gentamicin and colistin. CARBA-5 test detected VIM-type carbapenemase in these strains. These results were confirmed in the National Reference laboratory (NRL) for antibiotics. The NRL also proved the isolates to be identical by genotyping. Epidemiological investigation found out that bronchoscopy of all patients colonized with this strain was performed in the same operation theatre. 144 samples were taken from the environment of the bronchoscopy operating theatre and staff. The source of the carbapenemase-producing *Acinetobacter junii* was found in the heating bath for saline solution used in bronchoscopy procedure. Corrective actions were implemented and *Acinetobacter* spp. strain with this resistance phenotype has not been detected since.

**Conclusions:** Environmental sampling is essential during outbreaks, but it is also recommended to be done periodically as a prevention of outbreaks. It is crucial to search for the source of the contaminant in adequate areas.

## P196 Carbapenemase-producing enterobacteriaceae colonization at ICU admission: impact of immunosuppression on subsequent infection and outcome

### D Lopes^1^, ME Batista^1^, M Barbosa^1^, R Junior^2^, G Cristóvão^3^, P Nejo^4^, S Machado^1^, J Casimiro^1^, R Pereira^1^, F Cardoso^5^

#### ^1^Curry Cabral Hospital, Central Lisbon University Hospital, Department of Intensive Care Medicine (Unidade de Cuidados Intensivos Polivalente), Lisboa, Portugal, ^2^São José Hospital, Central Lisbon University Hospital, Department of Intensive Care Medicine (Unidade de Urgência Médica), Lisboa, Portugal, ^3^Curry Cabral Hospital, Central Lisbon University Hospital, Department of Infectious Diseases, Lisboa, Portugal, ^4^Santarém District Hospital, Department of Intensive Care Medicine, Santarém, Portugal, ^5^Curry Cabral Hospital, Central Lisbon University Hospital, Transplant Unit, Lisboa, Portugal

*Critical Care* 2024, **28(Suppl 1):** P196

**Introduction:** Previous studies showed carbapenemase-producing enterobacteriaceae (CPE) carriers have a high probability of developing a subsequent infection, with worse outcomes [1]. Association between immunosuppression and MDR colonization or infection has shown conflicting results in ICU. Our purpose was to assess outcomes in critically ill patients CPE colonized according to immune status.

**Methods:** We conducted a retrospective study on adult patients admitted to an ICU from 2019 to 2022 in a university hospital with a screening of CPE on admission ± 48 h. CPE infection on admission and ICU stay < 48 h were exclusion criteria. CPE colonization was the primary exposure. Hospital mortality was the primary endpoint. Associations were studied with logistic regression.

**Results:** Among 965 patients included, 631 (65%) were males and median age was 61 (49–69) years. Overall, 504 (52%) were admitted for non-surgical reasons, with a median SAPSII of 42 (28–55). Immunosuppression (IS) was present in 512 (53%) patients, the main cause being solid organ transplantation (386 (40%) patients). On ICU admission, 58 (6%) patients were CPE colonized and 35 (4%) developed a CPE infection. Overall, 261 (27%) patients died during hospital stay. CPE colonized patients were more likely admitted to ICU for urgent surgery (22% vs. 10%; *p* = 0.004), less likely immunosuppressed for any given reason (34% vs. 54%; *p* = 0.005), had higher SAPSII (51 vs. 41; *p* = 0.004), were more likely CPE infected (17% vs. 3%; *p* < 0.001), and more likely to die during hospital stay (48% vs. 26%; *p* < 0.001) than non-colonized ones (Table). Following adjustment for ICU admission type, IS status and SAPSII, CPE colonization was associated with higher odds of hospital mortality (aOR (95% CI) 2.00 (1.07–3.73); *p* = 0.031), contrary to CPE infection (aOR (95% CI) 1.62 (0.41–6.75); *p* = 0.49).

**Conclusions:** In a large cohort of critically ill patients, CPE colonization was associated with worse short-term mortality, irrespective of IS status.

**Reference**

1. Gomides MDA et al. PLoS ONE. 2022;17:e0262554

**Table (abstract P196):** Clinical characteristics, infection and outcomes of patients according to Carbapenemase-producing enterobacteriaceae colonization at ICU admission.

	All patients screened at ICU admission N = 965 (IQR or %)	No CPE colonization n = 907 (IQR or %)	CPE colonization n = 58 (IQR or %)	*p*
SAPS II score	42 (28–55)	41 (28–55)	51 (35–61)	0.004
Immunosuppression (n)	512 (53)	492 (54)	20 (34)	0.005
CPE infection (n)	35 (4)	25 (3)	10 (17)	< 0.001
Hospital length of stay (days)	25 (15–45)	24 (15–44)	31 (18–65)	0.033
ICU mortality	168 (17)	147 (16)	21 (36)	< 0.001
Hospital mortality	261 (27)	233 (26)	28 (48)	< 0.001

CPE carbapenemase-producing enterobacteriaceae, IQR interquartile range, SAPS II Simplified Acute Physiology Score II.

## P197 Is daptomycin a suitable alternative option for initial treatment of infections caused by vancomycin and/or linezolid resistant Gram-positives?

### J Závora, G Kroneislová, V Adámková

#### General University Hospital, Clinical Microbiology and ATB Centre, Prague, Czech Republic

*Critical Care* 2024, **28(Suppl 1):** P197

**Introduction:** The increasing antimicrobial resistance is usually associated with Gram-negative bacteria, but attention should also be paid to Gram-positive resistant bacteria. Linezolid-resistant strains of staphylococci and vancomycin and/or linezolid resistant enterococci are more frequently detected. Enterococci resistant to all commonly available antibiotics are no exception. These highly resistant strains mainly threaten immunocompromised, haemato-oncology patients, but can also cause hospital-acquired infections in patients without known immunosuppression.

**Methods:** We have selected 20 unique isolates of coagulase-negative staphylococci (CoNS) resistant to linezolid and 25 unique isolates of *E. faecium* resistant to vancomycin and/or linezolid obtained from clinical samples of patients hospitalized in General University Hospital in Prague in 2022–2023. Minimum inhibitory concentration values of vancomycin, teicoplanin, linezolid and daptomycin were in this study determined by gradient strip method.

**Results:** Interpretation criteria (breakpoints by EUCAST 2023) are provided for all tested antibiotics except for daptomycin in *E. faecium.* In CoNS group, no resistance to vancomycin or daptomycin was detected, resistance to teicoplanin was 20%. In *E. faecium* group, 56% of strains were resistant to linezolid and the same percentage resistant to vancomycin, 40% resistant to teicoplanin. All isolates of *E. faecium* were susceptible to daptomycin (using ECOFF according to EUCAST—8 mg/L).

**Conclusions:** Daptomycin could be an appropriate treatment option for infections caused by vancomycin and/or linezolid-resistant strains of Gram-positive cocci. It would be appropriate for official breakpoint to be defined. Because of the different results of susceptibility to vancomycin and teicoplanin, it is important to always test both antibiotics.

## P198 Factors associated with the usage of cefepime versus piperacillin/tazobactam in patients with community-acquired pneumonia admitted to the intensive care unit

### CC Serrano-Mayorga^1^, S Duque^2^, ED Ibáñez-Prada^1^, E Garcia-Gallo^2^, MP Rojas Arrieta^1^, IG Bustos Moya^1^, MJ Contreras^1^, AE Viñan Garces^1^, I Martin-Loeches^3^, LF Reyes^1^

#### ^1^Facultad de Medicina, Universidad de La Sabana, Bogota, Colombia, ^2^Pandemic Sciences Institute, University of Oxford, Oxford, UK, ^3^Multidisciplinary Intensive Care Research Organization (MICRO), Department of Intensive Care Medicine, St. James´s Hospital, Dublin, UK

*Critical Care* 2024, **28(Suppl 1):** P198

**Introduction:** Cefepime and piperacillin/tazobactam are antimicrobials recommended by the IDSA/ATS for empirical management of critical community-acquired pneumonia (CAP) patients at higher risk of CAP due to *Pseudomonas aeruginosa*. Concerns have been raised about which should be used in clinical practice. Thus, we aim to compare the effect of cefepime and piperacillin/tazobactam in critical CAP patients by simulating a randomized controlled trial.

**Methods:** Adult patients admitted to the intensive care unit (ICU) with CAP registered in the MIMIC-IV database treated with cefepime or piperacillin/tazobactam at admission were included in the analysis. Multivariate analyses, targeted maximum likelihood estimation (TMLE), and survival analyses were conducted to assess the impact of cefepime and piperacillin/tazobactam treatment on 28-day, hospital, and ICU mortality.

**Results:** A total of 2026 patients were included, 58.9% (947/2026) being male, with a mean (SD) age of 68.0 (15.4) years. Among them, 46.7% (947/2026) presented with respiratory failure, and 26.8% (543/2026) developed septic shock. A total of 68% (1384/2026) received Cefepime and 31.6% (642/2026) piperacillin/tazobactam-based treatment. Age, PTT, serum potassium and temperature were associate with preferring cefepime over piperacillin/tazobactam (OR 1.14 IC 95% [1.01–1.27], *p* = 0.03), (OR 1.14 IC 95% [1.03–1.26], *p* = 0.009), (OR 1.1 IC 95% [1.01–1.22], *p* = 0.039) and (OR 1.13 IC 95% [1.03–1.24], *p* = 0.014)] (Figure). Mortality among the study groups were similar. 28-day mortality (29.2% [404/1384] vs 30.7% [197/642], *p* = 0.53), hospital mortality (24.6% [340/1384] vs. 24% [154/642], *p* = 0.82) and the ICU mortality (15.2% [210/ 1384] vs. 14.2% [91/642], *p* = 0.6) (Figure).

**Conclusions:** Cefepime and piperacillin/tazobactam in ICU-admitted CAP patients could be equivalent. Clinicians may determine pReferences based on availability and safety profiles. Further prospective studies are needed to support these findings.

**Figure (abstract P198):**
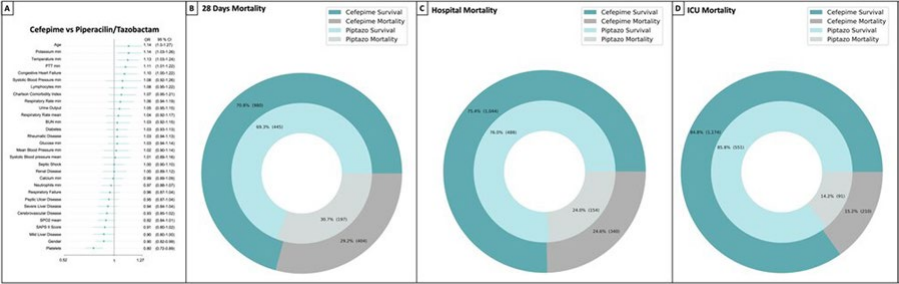
Panel **A** Multivariate logistic regression model for the use of cefepime or piperacillin/tazobactam; Panel **B** Comparison between mortality among cefepime and piperacillin/tazobactam at 28 days; Panel **C** Comparison between hospital mortality; Panel **D** Comparison between ICU mortality.

## P199 Continuous versus intermittent beta-lactam dosing in critically ill patients with sepsis: a randomized controlled trial

### AB Khan^1^, S Omar^2^

#### ^1^University of Witwatersrand, Critical Care, Johannesburg, South Africa, ^2^University of Witwatersrand, Intensive Care/Critical Care, Johannesburg, South Africa

*Critical Care* 2024, **28(Suppl 1):** P199

**Introduction:** To determine the effect of continuous/extended (CI) beta-lactam dosing in critically ill patients with sepsis compared to intermittent dosing (IB).

**Methods:** A prospective, open-labelled randomized controlled trial comparing IB vs CI at a multi-disciplinary adult, university-affiliated intensive care unit (ICU). We assessed four beta-lactam antibiotics: amoxicillin-clavulanate, piperacillin-tazobactam, imipenem and meropenem among patients meeting the sepsis-3 definition.

**Results:** Baseline characteristics were well matched (Table). The primary outcome, Day14 (D14) clinical cure was not significantly higher in the CI (52/64, 81.3%) versus IB (43/58, 74.1%), *p* = 0.19. ICU length of stay (days) was not significantly different CI (9, 5–16) versus IB (9.5, 5–13), *p* = 0.58. D90 mortality trended lower in the CI group (12/52, 23.1%) versus IB (20/49, 40.8%), *p* = 0.056. D90 mortality among the culture-negative sepsis group demonstrated significantly lower mortality in the CI group (4/24, 16.7%) compared to IB (9/20, 45%), *p* = 0.040. D90 mortality among patients with a source of sepsis in non-pulmonary sites demonstrated a trend to lower mortality in the CI group (6/28, 21.4%) versus IB (11/24, 45.2%), *p* = 0.062. The trend to lower D90 mortality in the CI versus IB groups was found for amoxicillin-clavulanate (16.7% vs. 42.1%), piperacillin-tazobactam (22.2 vs. 31.6%) and Meropenem/Imipenem (31.3% vs. 54.5%).

**Conclusions:** Despite a non-significant difference in Day14 clinical cure rates for septic patients receiving continuous beta-lactam antibiotics, we found significantly lower D90 mortality among the culture-negative sepsis group receiving continuous infusions and a trend to lower D90 mortality for the culture-positive group.

**Table (abstract P199):** Baseline characteristics

Variable	IB median (IQR)	CI median (IQR)	*p*
Age years	36 (25–50)	31 (26–39)	0.09
Male n (%)	34/58 (58.6)	41/64 (64)	0.54
APACHE II score	9.5 (6–13)	8 (5–13)	0.34
Septic shock	23/58 (39.7)	28/64 (43.8)	0.65
Diagnosis: medical	23/58 (39.7%)	20/64 (31.3%)	0.33
Diagnosis: surgical	12/58 (20.6%)	13/64 (20.3%)	0.96
Diagnosis: trauma	23/58 (39.7%)	31/64 (48.4%)	0.33

## P200 Vaccine effectiveness of the national vaccination campaign against SARS-CoV-2 in patients with acute respiratory failure (ARF) with criteria for ventilatory support: a multicentric cohort study

### N Fuentes^1^, L Stein^1^, A Lagazio^2^, M Laíz^3^, A Gallardo^4^, L De Wouters^5^, I Santomil^1^, M Busico^3^, M Mogadouro^2^, M Esperatti^1^

#### ^1^Hospital Privado de Comunidad, Critical Care Unit, Mar del Plata, Argentina, ^2^Sanatorio de la Trinidad Palermo, Critical Care Unit, Buenos Aires, Argentina, ^3^Clínica Olivos SMG, Critical Care Unit, Buenos Aires, Argentina, ^4^Sanatorio Clínica Modelo de Morón, Critical Care Unit, Buenos Aires, Argentina, ^5^Hospital Privado de Comunidad, Infectious Disease Department, Mar del Plata, Argentina

*Critical Care* 2024, **28(Suppl 1):** P200

**Introduction:** Vaccine effectiveness (VE) against SARS-CoV-2 infection, hospitalization, and death from COVID-19 has been demonstrated [1–3]. In Argentina, it was also confirmed in adults ≥ 60 years VE to prevent infection and death [4]. However, it has not been possible to evaluate VE in hospitalized and critically ill patients due lack of availability in surveillance data. Objective: to estimate the VE for death and other clinically relevant outcomes in critically ill patients due to ARF-COVID-19.

**Methods:** A multicenter retrospective cohort study in 5 ICUs in Argentina. All patients admitted who have ARF-COVID-19 confirmed by real-time PCR were included. Patients were classified as Vaccinated when they had received at least one dose at least 14 days before admission to the ICU; the rest of the patients were classified as Unvaccinated. The outcomes were mortality, endotracheal intubation (ETI), support ventilatory-free days (SVFD), ICU stay, and hospital stay. An inverse probability weighting (IPW) approach was used to control for potential treatment-assigned bias. A robust approach analysis was used to adjust other potential confounders.

**Results:** 651 patients were included, 91 were vaccinated [89.9% (n = 80) with 1 dose]. No differences were found between the groups in any outcome evaluated (Table). Differences in ICU and hospital mortality were not confirmed after adjustment for confounding factors [OR 0.66 (95% CI 0.36–1.22) and 0.69 (95% CI 0.37–1.27), respectively].

**Conclusions:** No differences were found between vaccinated and unvaccinated in the results, this could be due to the small number of vaccinated, incomplete regimens (88% only 1 dose), or finally immunized patients who present with ARF-COVID-19 could express some clinical-immunological response of the host that could not be explored.

**References**

1. López Bernal J et al. BMJ. 2021;373:n10882.
2. Haas EJ et al. Lancet. 2021;397:1819–1829.
3. Jara A et al. N Engl J Med. 2021;386:875–884.
4. Rearte A et al. Lancet. 2022;399:1254–1264.

**Table (abstract P200):** Clinical outcomes (unadjusted)

Outcome	Vaccinated (n = 91)	Unvaccinated (n = 560)	*p*
In-hospital mortality, n (%)	37 (40)	155 (28)	0.01
ETI, n (%)	41 (45)	248 (44.3)	0.89
ICU stay (days), median (percentile 25–75)	8 (4–15)	9 (4–15)	0.85
Hospital stay (days), median (percentile 25–75)	13 (10–23)	13 (9–28)	0.93
SVFD (days), median (percentile 25–75)	19 (0–24)	18.5 (0–25.5)	0.38
Time to death (days), median. (percentile 25–75)	15 (9–25)	8 (5–11)	< 0.001
Time to ETI (days), median (percentile 25–75)	2 (1–4.5)	1 (0–2)	< 0.001

## P201 SARS-CoV-2 pneumonia in intensive care unit: predictors of major cardiovascular events

### D Hamdi^1^, I Belloumi^2^, W Demni^1^, I Sahnoun^1^, A Houatmia^2^, K Ouerhani^1^, M Bahri^1^, D Abdelhadi^1^, NE Nouira^1^

#### ^1^Mongi Slim Academic Hospital, Emergency Department, Tunis, Tunisia, ^2^Tabarka Hospital, Intensive Care Unit, Jendouba, Tunisia

*Critical Care* 2024, **28(Suppl 1):** P201

**Introduction:** SARS-CoV-2 pneumonia is the most severe manifestation of COVID-19, and most frequent cause of morbi-mortality. Cardiovascular events can increase death in patient with SARS-CoV-2 pneumonia. The aim of our study was to determine the predictors factors of major cardiovascular events in patients admitted to the intensive care unit for SARS-CoV-2 pneumonia.

**Methods:** Retrospective, descriptive, and analytical study conducted over a period of 12 months (October 2020–September 2021). Patients aged 18 years and older, admitted to the intensive care unit for the management of SARS-CoV-2 pneumonia, Predictors of major cardiovascular events were identified through univariate and multivariate analysis.

**Results:** We included 120 patients. The average age was 63 ± 17 years, with a predominance of males. The main cardiovascular risk factor was hypertension (22.5%), the most frequent comorbidities were arrythmia (7.8%) and coronary disease (5.2%). Major symptoms included dyspnea (80.8%) and coughing (76.6%). Half of patients had a systolic blood pressure < 90 mmHg and 53% had SPO_2_ < 91%·PaO_2_/FiO_2_ ratio was less than 100 in 37.5% of cases. The incidence of major cardiovascular events was 74.2%. Arrythmia was the most common cardiovascular events (25.2%)% followed by hemodynamic shock (15.8%). The predictors of major cardiovascular events identified through univariate analysis were: age > 65 years, male gender, hypertension, chest pain (*p* = 0.001, OR 16), hyperleucocytosis (*p* < 0.001, OR 5.88), creatinine (*p* < 0.001, OR 59), lactate dehydrogenase (*p* < 0.001, OR 4.65), mechanical ventilation (*p* = 0.001, OR 5.76). In multivariate analysis, the independent predictors of major cardiovascular event were: age > 65 years, male gender, chest pain and mechanical ventilation.

**Conclusions:** Determining the predictors of cardiovascular events could help clinicians to identify patients with high risk of short-term complications, to optimize their management and then reduce morbi-mortality.

## P202 The trend of severe neck infections patients and the waves of COVID-19

### R Echigoya^1^, T Ohtake^2^

#### ^1^Kurashiki Central Hospital, Emergency and Critical Care Center, Okayama, Japan, ^2^Kurashiki Central Hospital, Okayama, Japan

*Critical Care* 2024, **28(Suppl 1):** P202

**Introduction:** There are diseases whose prevalence has changed around the time of the COVID-19 pandemic. In particular, it is said that the prevalence of depression and anxiety disorders has sharply increased. Following the spread of COVID-19 in Japan, there has been an increase in neck infections requiring intensive care unit management at our hospital, prompting an investigation into the actual situation.

**Methods:** From the medical records of our hospital, we retrospectively extracted patients with severe neck infections admitted to the emergency Intensive Care Unit (EICU) from January 2013 to October 2023, before the onset of the COVID-19 pandemic. We examined age, gender ratio, underlying diseases, presence of endotracheal intubation and mechanical ventilation, number of surgical drainages, and the number of tracheostomies.

**Results:** During the period, 85 individuals received treatment in the EICU. The fluctuation in the annual number of admitted patients and the prevalence of COVID-19 in Japan is depicted in the Figure. The average age was 57 years (range: 22–90), with 52 males and 33 females. The diagnosed diseases (with some overlap) included acute epiglottitis in 28, peritonsillar abscess in 12, acute pharyngolaryngitis in 12, retropharyngeal abscess in 5, and mediastinitis in 2. Cases with abscess formation in any part of the neck (neck abscess) amounted to 25 individuals. Almost all cases received antibiotic therapy, with 39 individuals undergoing surgical drainage, 80 requiring endotracheal intubation and mechanical ventilation, and 11 necessitating tracheostomy. Three patients died.

**Conclusions:** After the onset of the COVID-19 pandemic, there was a noticeable increase in the number of patients with neck infections requiring intensive care management. However, the proportion of patients requiring tracheostomy was remarkably low, and it was believed that with proper airway management and infection control, the prognosis could be favorable.

**Figure (abstract P202):**
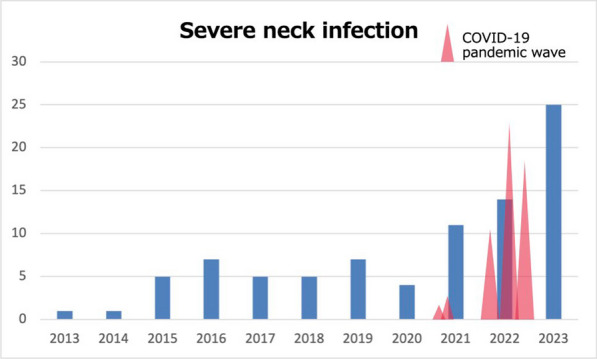
The number of severe neck infections patients and the waves of COVID-19.

## P203 Comparison of the results of the use of dexamethasone compared with methylprednisolone pulses in severe pneumonia due to COVID-19. Cohort study

### N Fuentes^1^, M Olmos^1^, ME González^1^, L De Wouters^2^, I Santomil^1^, A Miranda Tirado^1^, J Suárez Díaz^3^, L Stein^3^, M Flamini Marczuk^1^, M Esperatti^1^

#### ^1^Hospital Privado de Comunidad, Critical Care Unit, Mar del Plata, Argentina, ^2^Hospital Privado de Comunidad, Infectious Disease Department, Mar del Plata, Argentina, ^3^Hospital Privado de Comunidad, Mar del Plata, Argentina

*Critical Care* 2024, **28(Suppl 1):** P203

**Introduction:** SARS-CoV-2 infection can cause acute respiratory failure (ARF) associated with COVID-19 with a high mortality rate. The use of systemic steroids showed improvement in mortality, especially in patients requiring invasive mechanical ventilation (IMV) [1]. There are some reports on the use of pulse methylprednisolone (MPS) in COVID-19 with controversial results [2–4]. This study aims to evaluate the effectiveness and safety of patients who received MPS pulses compared with dexamethasone.

**Methods:** A retrospective cohort study was performed comparing COVID-19 patients ≥ 18 years with ARF who received MPS compared with dexamethasone from July 2020 to September 2021. The effectiveness outcomes [endotracheal intubation (ETI), ICU- mortality (ICU), in-hospital mortality] and safety outcomes (insulin requirements and infections) were evaluated. An inverse probability weighting-propensity score (IPW-PS) approach was used to control for potential treatment-assigned bias. A robust approach analysis was used to adjust other potential confounders.

**Results:** 328 patients were included, 100 in the dexamethasone group and 228 in the MPS group. The characteristics were balanced after the IPW-PS (standardized difference of the mean < 0.15) (Figure panel A). Patients in the MPS group compared with the patients in the dexamethasone group had higher ETI rates (30% vs. 10%, *p* = 0.001), higher ICU mortality (32% vs. 14%, *p* = 0.001), higher in-hospital mortality (35% vs. 15%, *p* < 0.001), higher insulin requirement (*p* = 0.01); and higher number of infections (*p* < 0.001) (Figure panel B).

**Conclusions:** The use of MPS is associated with increased ETI rates, mortality, higher insulin requirement, and higher incidence of infections.

**References**

1. The RECOVERY Collaborative Group et al. N Engl J Med. 2021;384:693–704
2. Jerónimo CMP et al. Clin Infect Dis. 2021;72:e373-e381.
3. Ranjbar K et al. BMC Infect Dis. 2021;21:337.
4. Cusacovich I et al. Mediators Inflamm. 2021;2021:6,637,227

**Figure (abstract P203):**
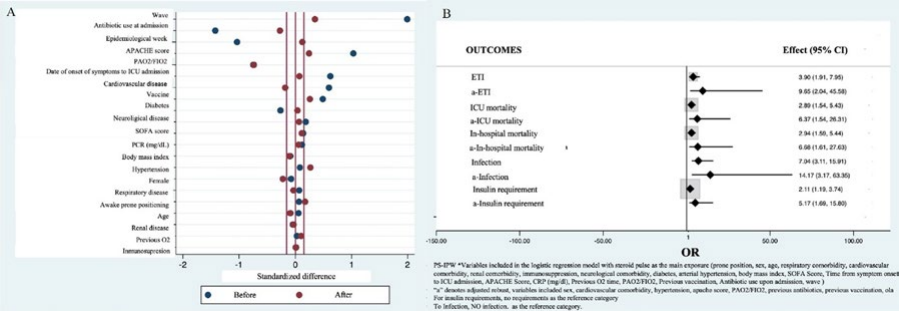
Panel **A** standardized differences of the covariates before and after applying the inverse probability weighting propensity score (IPW-PS). Panel **B** odd ratios post-IPW-PS and adjusted for the most relevant clinical outcomes.

## P204 Impact of COVID-19 pandemic in rate of ventilator-associated pneumonias caused by MDR pathogens: a 5-year retrospective study

### JP Cidade^1^, H Moreira^1^, R Carnevale^1^, L Morais^1^, T Miranda^1^, G Guerreiro^1^, P Fidalgo^1^, P Moniz^1^, E Gonçalves^2^, P Póvoa^1^

#### ^1^Hospital São Francisco Xavier, Intensive Care Department, Lisbon, Portugal, ^2^Hospital São Francisco Xavier, Microbiology Department, Lisbon, Portugal

*Critical Care* 2024, **28(Suppl 1):** P204

**Introduction:** Multidrug-resistant (MDR) bacteria remains a health threat to intensive care unit (ICU) patients worldwide. These rates might have been further aggravated by the impact of COVID-19, with secondary nosocomial infections being reported in higher rates, with higher antibiotic consumption, potentially leading to increased risk of MDR. We aimed to describe the prevalence of ventilator-associated pneumonias (VAP), caused by MDR bacteria, and to describe their clinical characteristics and outcomes.

**Methods:** Single center retrospective cohort study performed at two ICU of a university hospital. All adult mechanically ventilated (MV) patients, admitted between 2018 and 2022, were included if they presented a positive lower respiratory tract sample, due to clinical suspicion of VAP. Patients were divided according to the occurrence of VAP by MDR pathogen, and according to their occurrence before or after the beginning of COVID-19 pandemic (2020).

**Results:** 126 patients were included, and 37 of the observed VAPs were caused by MDR. No differences were found between MDR and non-MDR VAP patients in demographic and clinical characteristics or in ventilatory, vasopressor or renal supports. Furthermore, these patients did not differ in ICU length-of-stay or 28-day mortality rates. On the other hand, patients with VAP after 2020 (n = 72; 36 had COVID-19 pneumonia at admission) had significantly lower rates of isolation of MDR bacteria, and higher rates of invasive multiorgan support. Moreover, at sampling dates, VAP patients after COVID-19 pandemic presented lower PaO_2_/FiO_2_ and higher C-reactive protein serum levels. These patients also had higher ICU length-of-stay but lower mortality rate (*p* = 0.004 and log-rank test *p* = 0.03, Figure).

**Conclusions:** Critically ill patients undergoing invasive MV during and after COVID19 pandemic appear to present lower rates of MDR pathogens causing VAP and lower mortality rates despite higher need of organ support requirements and ICU length-of-stay.

**Figure (abstract P204):**
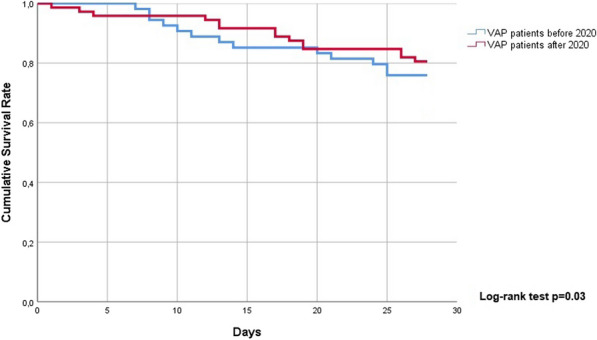
Kaplan–Meier survival analysis of mechanically ventilated patients with VAP diagnosis before and after COVID-19 pandemic.

## P205 How often do we need to adjust the dose of linezolid in critically ill patients to get the target?

### I Jordão^1^, N Andrade^2^, CM Silva^2^, M Peixinho^3^, M Rocha^4^, F Parente^1^, JP Baptista^2^

#### ^1^Centro Hospitalar Universitário de Coimbra, EPE, Farmacologia Clínica, Coimbra, Portugal, ^2^Centro Hospitalar Universitário de Coimbra, EPE, Serviço de Medicina Intensiva, Coimbra, Portugal, ^3^Centro Hospitalar Universitário de Coimbra, EPE, Serviço de Patologia Clínica, Coimbra, Portugal, ^4^Centro Hospitalar Universitário de Coimbra, EPE, Farmácia Hospital, Coimbra, Portugal

*Critical Care* 2024, **28(Suppl 1):** P205

**Introduction:** Critically ill patients present unpredictable changes in the pharmacokinetics of different drugs [1] including linezolid (LZD) [2], increasing the risk of toxicity due to accumulation and the risk of underdosing with therapeutic failure. This study aimed to assess the importance of TDM of LZD in patients admitted to an Intensive Care Unit (ICU) of a tertiary hospital.

**Methods:** Retrospective study of patients admitted to a multipurpose ICU between 2022/11 and 2023/07, who received treatment with LZD and performed TDM. To determine LZD exposure, minimum concentrations (C_min_) and maximum concentrations (C_max_) were measured after a minimum of 3 treatment administrations. The primary endpoint were the achievement of a Cmin of LZD between 2 and 8 mg/L and AUC > 100 µg/mL/h. Secondary endpoints included analysis of TDM-guided dose adjustments.

**Results:** A total of 56 patients were included (Table). Most patients upon admission had medical pathologies (53.4%). The median duration of LZD treatment in the ICU was 8 days [4.0–10.5]. The therapeutic range was achieved at the first measurement in 16/56 patients (28.6%) with a median C_min_ 2.6 [1.5–6.9] mg/L, median AUC 137.5 [88.0–250.8] µg/mL/h. After TDM-guided dose adjustments, a total of 26 patients (46.4%) were within therapeutic range after a median of 2.8 [1.5–3.8] days. After 2 TDM adjustments, 31 patients (55.4%) were within therapeutic range after a median of 1.6 [1.0–2.9] days. In 21 patients (37.5%), therapeutic levels were not reached, 25% had subtherapeutic levels and 12.5% had toxic levels. In 19 patients (33.9%) after reaching therapeutic levels, maintaining dose and administration interval resulted in toxic or subtherapeutic levels.

**Conclusions:** Use of TDM in critically ill patients under LNZ treatment increases the probability of reaching the therapeutic target, reducing the risk of underdosing.

**References**

1. Abdul-Aziz MH et al. Intensive Care Med. 2020;46:1127–1153
2. Dong H et al. Int J Antimicrob Agents. 2011;38:296–300

**Table (abstract P205):** Demographic and clinical characteristics of LZD-TDM patients

Characteristic	Result
Age, years, median [IQR]	64.5 [53.5–74.0]
Male, n (%)	42 (75.0%)
Body mass index, Kg/m^2^, median [IQR]	30.9 [27.9–34.9]
SOFA score, median [IQR]	11.0 [9.0–13.5]
SAPS 3, median [IQR]	73.0 [60.3–82.8]
Creatinine clearance mL/min, median [IQR]	32.6 [7.0–82.9]
Renal replacement therapy, n (%)	24 (42.9%)
Septic shock n (%)	51 (91.1%)

IQR – interquartile range

## P206 Gas-producing liver abscess caused by *C. perfringens:* a case series review

### M Iwahara, R Echigoya

#### Kurashiki Central Hospital, Intensive Care Department, Okayama, Japan

*Critical Care* 2024, **28(Suppl 1):** P206

**Introduction:** *Clostridium perfringens* (hereinafter *C. perfringens*) is known as the causative agent of gas gangrene resulting from trauma, but it can also be a causative agent of food poisoning and sepsis. The development of gas-producing liver abscess is often accompanied by intravascular hemolysis, leading to a rapid and frequently fatal course within a short period.

**Methods:** From April 2010 to April 2023, patients admitted to our hospital were studied retrospectively for cases of gas-producing liver abscess with *C. perfringens* detected in blood cultures. Data including age, underlying conditions, presence of intravascular hemolysis, treatment, mortality rate, and time from admission to death were extracted from our medical chart.

**Results:** The results are presented in the Table. There were a total of 9 cases of gas-producing liver abscess caused by *C. perfringens*, with a median age of 77 years (range: 74–82). Malignant tumors were observed in 66.7% (6/9 cases), and diabetes was noted in 44.4% (4/9 cases). During examination at the emergency department, intravascular hemolysis was observed in 88.9% (8/9 cases), and 66.7% (6/9 cases) underwent drainage in addition to antibiotic treatment. A total of 55.6% (5/9 cases) resulted in mortality, with a median time from admission to death of 24 h (range: 19–94). Several cases without underlying conditions such as malignant tumors or diabetes have been observed to have long-term survival.

**Conclusions:** Gas-producing liver abscess caused by *C. perfringens* tends to occur in immunocompromised patients such as those with malignant tumors or diabetes. This condition is characterized by the rapid development of intravascular hemolysis, leading to a swift progression to death. In cases of gas-producing liver abscess with concurrent intravascular hemolysis, early initiation of drainage and antibiotic treatment with consideration for *C. perfringens* is desirable. However, even with prompt treatment, survival is often challenging in many cases.

**Table (abstract P206):** Results

Variable	All n = 9
Age median (IQR)	77 (74–82)
Malignancy n%	66.7% (6/9)
Diabetes n%	44.4% (4/9)
Intravascular hemolysis n%	88.9% (8/9)
Drainage n%	66.7% (6/9)
Death n%	55.6% (5/9)
Hospitalization-death time median (IQR)	24 (19–94)

## P207 High dose nebulized colistin improves mortality in bacteremic ventilator-associated pneumonia from *Acinetobacter baumannii*

### N Kazakos^1^, N Lagos^1^, T Maniatopoulou^1^, E Toli^1^, A Papathanasiou^1^, G Papathanakos^1^, C Kittas^2^, D Koulenti^3^, V Koulouras^4^, I Andrianopoulos^1^

#### ^1^University Hospital of Ioannina, Intensive Care Unit, Ioannina, Greece, ^2^University Hospital of Ioannina, Microbiology Department, Ioannina, Greece, ^3^The University of Queensland, UQ Centre for Clinical Research / Faculty of Medicine, Brisbane, Australia, ^4^University Hospital of Ioannina, Intensive Care Medicine, Ioannina, Greece

*Critical Care* 2024, **28(Suppl 1):** P207

**Introduction:** *Acinetobacter baumannii* (AB) is a difficult- to-treat (DTR) pathogen that causes ventilator-associated pneumonia (VAP) associated with high mortality. In order to improve the outcome of such infections nebulized colistin (NC) was introduced with promising but conflicting results on mortality in earlier studies. Currently, NC is used at a much higher daily dose from that used previously. Nevertheless, there is little evidence on the effect of high dose NC in the outcome of AB VAPs, especially in the current era where the percentage of colistin resistant AB strains is rising.

**Methods:** We conducted a retrospective study comparing bacteremic AB VAP that were treated with NC with those that were not.

**Results:** Overall, 59 patients (21 treated and 38 not treated with NC) were included. The 28-day mortality and 7-day mortality were significantly lower in the patient group treated with NC (52.4% vs. 78,9%, *p* = 0.034) and (9.5% vs. 47.4%, *p* = 0.003) respectively. Patients treated with NC had a higher percentage of sepsis resolution by day 7 (38.1% vs. 13.5%, *p* = 0.023) and were more likely to be off vasopressors by day 7 (28.6% vs. 8.1%, *p* = 0.039). There was no statistical difference among the two groups in their baseline characteristics or complications.

**Conclusions:** The addition of NC in the treatment regime of AB VAP improves patients’ mortality.

## P208 Evaluation of the safety and efficacy of inhaled liposomal amphotericin B in mechanically ventilated patients

### M López Olivencia^1^, N Paredes de Dios^1^, A De Abreu Ramírez^1^, J Sáez de la Fuente^2^, S García Plaza^1^, S Sáez Noguero^1^, J Fortún Abete^3^, A Blandino Ortiz^1^, R de Pablo Sánchez^1^, MC Soriano Cuesta^1^

#### ^1^Ramón y Cajal University Hospital, Intensive Care Medicine, Madrid, Spain, ^2^Ramón y Cajal University Hospital, Pharmacy, Madrid, Spain, ^3^Ramón y Cajal University Hospital, Infectious Diseases Department, Madrid, Spain

*Critical Care* 2024, **28(Suppl 1):** P208

**Introduction:** Primary objective: to assess the safety of inhaled liposomal amphotericin B (ABL-inh) in mechanically ventilated (MV) patients through a patient safety-focused protocol. Secondary objective: to analyze the efficacy of ABL-inh prophylaxis in patients at risk of invasive pulmonary aspergillosis (IPA) [1].

**Methods:** Retrospective study in the ICU (June 2021–2023). Included MV patients receiving ABL-inh for > 7 days. Administration protocol: ABL-inh nebulization (25 mg/48 h), Aerogen® nebulizer, reconstitution with water, active humidification system, and post-nebulization filter replacement. Recorded complications: bronchospasm and expiratory valve obstruction with/without clinical repercussions. Efficacy of ABL-inh prophylaxis defined as absence of IPA during ICU stay. Demographic data, severity scores, ABL-inh indication, and clinical outcomes collected. Results expressed as mean ± standard deviation.

**Results:** 35 patients received ABL-inh. Epidemiological data and outcomes in the Table. 85.7% (30/35) received ABL-inh prophylaxis; 40% (12/30) hematologic/oncologic/immunosuppressed patients, 60% (18/30) other risk factors along with environmental conidia contamination (> 15 CFU/m^3^). 5 patients received ABL-inh as adjunctive therapy. Complications associated with ABL-inh observed in 17.1% (6/35) of patients: 3 with resolved bronchospasm upon ABL-inh withdrawal, 2 with expiratory valve obstruction without clinical impact, and 1 with a limited episode of hypoxemia due to expiratory valve obstruction. ABL-inh prophylaxis was effective in 80% (24/30). 4/6 who developed IPA under prophylactic treatment were high-risk immunosuppressed patients.

**Conclusions:** Administration of ABL-inh in MV patients was safe and well-tolerated. Implementation of an ABL-inh protocol could be effective in preventing complications, favorably tipping the risk–benefit balance for MV patients at risk of IPA.

**Reference**

1. Intensive Care Med. 2022;48:360–361

**Table (abstract P208):** Epidemiological data

Characteristic	(n = 35 patients)
SOFA score (mean ± SD)	8.10 ± 3.43 points
APACHE score (mean ± SD)	17.93 ± 4.78 points
ICU length of stay (mean ± SD)	48.89 ± 28.82 days
Days of mechanical ventilation (mean ± SD)	41.20 ± 24.63 days
Days of ABL-inh (mean ± SD)	17.06 ± 11.84 days
Complications (%)	6/35 (17.1) patients
Development IPA under prophylactic treatment (%)	6/30 (20%) patients

## P209 Analysis of *Candida* spp. infections in adult intensive care units in Santander Colombia

### JA Cárdenas Londoño^1^, J Leon Gomez^2^

#### ^1^Fundación Cardioinfantil, Cardiovascular Critical Care Unit, Bogotá DC, Colombia, ^2^Centro de Tratamiento e Invetigacion sobre Cancer Luis Carlos Sarmiento Angulo - CTIC, Hospitalization Unit. Fundacion CTIC, Bogotá DC, Colombia

*Critical Care* 2024, **28(Suppl 1):** P209

**Introduction:** Invasive mycoses are linked to higher mortality and morbidity. Most of these infections are hospital acquired, are more frequent in intensive care units and account approximately for 15% of healthcare related infections [1].

**Methods:** Ecologic observational study which collected data from The National Administrative Department of Statistics.

**Results:** Nine hospitals in Santander, Colombia, reported 768 hospital acquired infections in adults. Approximately 9.8% of those infections were caused by *Candida* spp. Most of the cases occurred in male patients (54.6%), with an average age of 59 years. Most of the cases are caused by *Candida albicans* and *Candida tropicalis*, 46% and 32% respectively. The most common infection sites were the urinary tract and the bloodstream. The average mortality rate was 22.6% between 2016 and 2022. The average age of patients who died was higher than those who survived (57 years and 62 years, respectively) but there was no statistically significant association between average age and mortality (*p* = 0.33).

**Conclusions:** Most cases are diagnosed in male patients. The main etiological agents are *Candida albicans* and *Candida tropicalis*, and non-albicans species are becoming relevant pathogens that cause many invasive mycoses in this series as well as other reported ones. In this case series *Candida* spp. isolates were isolated mainly in urine and blood culture with little representation of other sample types. Age has been identified as a relevant risk factor, with most cases occurring in patients older than 60 years old. The average mortality rate reported in this case series was 22.6%, with previously reported mortality rates as higher as 40–50%. Change in the number of confirmed *Candida* spp. infections between 2020 and 2021 probably attributed to an increase in the number of ICU patients due to the COVID-19 pandemic.

**Reference**

1. Cortés JA et al. Biomedica. 2020;40:195–207.

## P210 Socio-demographic factors associated with mortality from tuberculous meningitis in Colombia

### JA Cárdenas Londoño^1^, A Obando^2^

#### ^1^Fundación Cardioinfantil, Cardiovascular Critical Care Unit, Bogotá DC, Colombia, ^2^Fundación Santa Fe de Bogotá, Anesthesia Department, Bogotá DC, Colombia

*Critical Care* 2024, **28(Suppl 1):** P210

**Introduction:** Tuberculous meningitis is a manifestation of extrapulmonary tuberculosis, developing in 1–5% of the approximately 10 million cases of TB worldwide. Is the most lethal form of *Mycobacterium tuberculosis* infection [1].

**Methods:** Collected data from the National Administrative Department of Statistics between 2007 and 2022 [2]. Univariate analysis included central tendency measures depending on data nature and distribution, bivariate analysis included X2 test for independence and logistic regression modeling to obtain OR’s with their corresponding 95% Confidence Intervals. The statistical significance of the tests was defined as *p* < 0.05.

**Results:** A total of 5657 patients were obtained. The age range was 1 to 85 years. There were 127 cases of migrant population, 91% of the patients were hospitalized at the time of the report. The male to female presentation ratio is 2:1. Mortality by age group is higher in Senior 29.8 and was explored as a risk factor for mortality with a statistically significant P. Mortality is higher in the group of hospitalized patients 24.6% compared to non-hospitalized patients 7.2%. Mortality in the migrant population and the general population is the same. The population with a subsidized type of social security has a mortality of 26.4% compared to 19.3% for the contributory type.

**Conclusions:** This is the largest series reported by a country. We found statistically significant risk factors associated with mortality to be age over 65 years, affiliation to subsidized regime, hospitalized at the time of diagnosis, there is no association between geographical location and mortality, we also identified that hospitals with lower number of cases have higher mortality.

**References**

1. Navarro-Flores A et al. J Neurol 2022;269:3482–94
2. https://www.datos.gov.co/

## P211 In-hospital mortality in adult immunocompromised patients with community-acquired pneumonia admitted to the ICU

### AE Viñan Garces^1^, JC Olivella^1^, ED Ibáñez-Prada^1^, S Duque^2^, E Garcia-Gallo^2^, JM Restrepo^1^, EG Couto-Luvie^1^, LF Reyes^1^

#### ^1^Facultad de Medicina, Universidad de La Sabana, Bogota, Colombia, ^2^Pandemic Sciences Institute, University of Oxford, Oxford, UK

*Critical Care* 2024, **28(Suppl 1):** P211

**Introduction:** Community-acquired pneumonia (CAP) in immunocompromised patients represents a heterogeneous group with outcomes and specific pathogens related to the immunosuppression state. This study aims to determine which cause of immunosuppression is associated with a higher risk of in-hospital mortality in immunosuppressed adults diagnosed with CAP admitted to the intensive care unit (ICU).

**Methods:** An observational prospective cohort study was performed based on the MIMIC-IV database. Adult patients with CAP and immunosuppression admitted to the ICU during the first 24 h between 2008 and 2019 were included. The total cohort was subcategorized according to immunosuppression cause (i.e., solid tumor, hematological tumor, autoimmunity status, transplant status, asplenia, human immunodeficiency virus [HIV], sickle cell disease, and inflammatory bowel disease). A multivariate logistic regression model was developed to evaluate the relationship between immunocompromise and in-hospital mortality.

**Results:** A total of 1776 patients were included; 58.89% were male with a median age (IQR) of 68 (56–79). Solid tumor was the most frequent cause of immunosuppression (55.68% [989/1776]), followed by hematologic tumor (17,3% [307/1779]). A quarter of the cohort (25.11% [446/1776]) had an etiological microbial isolation. Typical bacterial pathogens related to CAP were the most frequently isolated (57.39% [256/446]). A total of 71,6% (1274/1779) of patients died during the hospital admission. Older age and solid tumors were associated with a two-fold increase in mortality risk among CAP patients with immunosuppression (Table).

**Conclusions:** This study exposes that among immunosuppressed patients with CAP, solid tumor is strongly associated with in-hospital mortality, and each immunosuppression cause represents a different mortality risk for this population. Immunosuppressed patients with CAP might have different clinical outcomes according to the etiology of their immunosuppression and should be addressed individually.

**Table (abstract P211):** Univariate and multivariate analysis of immunosuppression cause and in-hospital mortality in immunosuppressed patients with CAP admitted to ICU

Variables	Univariate analysis OR (95% CI)	*p* value	Multivariate analysis OR (95% CI)	*p* value
Age	1.03 (1.99–3.03)	< 0.01	1.02 (1.01–1.03)	< 0.01
Solid tumor	2.46 (1.99–3.03)	< 0.01	1.88 (1.48–2.38)	< 0.01
Asplenia	0.23 (0.12–0.59)	0.01	0.38 (0.16–0.88)	0.03
HIV/AIDS	0.53 (0.35–0.82)	0.01	0.88 (0.56–1.39)	0.59
Inflammatory bowel disease	0.47 (0.30–0.74)	0.01	0.60 (0.38–0.95)	0.03
Autoimmune status	0.51 (0.36–0.73)	< 0.01	0.67 (0.46–0.97)	0.03
Hematological tumor	1.12 (0.85–1.48)	0.45		

## P212 SCORE2 as a prediction severity score for patients with community acquired pneumonia: an analysis of the ACCESS study

### S Doulou^1^, K Dakou^2^, K Leventogiannis^1^, E Giamarellos-Bourboulis^1^

#### ^1^National and Kapodistrian University of Athens, 4th Department of Internal Medicine, Medical School, Athens, Greece, ^2^Hellenic Institute for the Study of Sepsis, Athens, Greece

*Critical Care* 2024, **28(Suppl 1):** P212

**Introduction:** SCORE2 is a combination of multiple commonly measured patient characteristics that is suggested by the Food and Drug Administration of the USA as a tool for early recognition of patients with COVID-19 at risk for progression into severe respiratory failure. It is investigated if this score can also identify patients with community acquired pneumonia (CAP) at risk for unfavorable outcome.

**Methods:** ACCESS is a prospective, double-blind randomized controlled trial (EudraCT 2020-004452-15; ClinicalTrials.gov NCT04724044) investigating early clinical anti-inflammatory responses of clarithromycin treatment in CAP. The prognostic performance of SCORE2 was analyzed in the 133 patients allocated to the comparator arm receiving treatment with standard of care antibiotics and placebo. SCORE2 was calculated using three binary variables from the medical history (chronic renal disease, stroke, smoking) and the cutoffs from 5 continuous variables (urea, SOFA score, neutrophil to lymphocyte ratio (NLR), age, hemoglobin). Survival analysis for 28-day mortality was done.

**Results:** Ninety-eight patients had SCORE2 < 3 and 35 patients SCORE2 3 or more. 3.8% and 20.6% respectively progressed into acute respiratory distress syndrome (ARDS) and need for mechanical ventilation the first 8 days. 28-day mortality was 7.7% and 30.8% respectively (Figure).

**Conclusions:** SCORE2 is an easy applicable score for the early detection of patients with CAP at risk for progression into ARDS and death.

**Figure (abstract P212):**
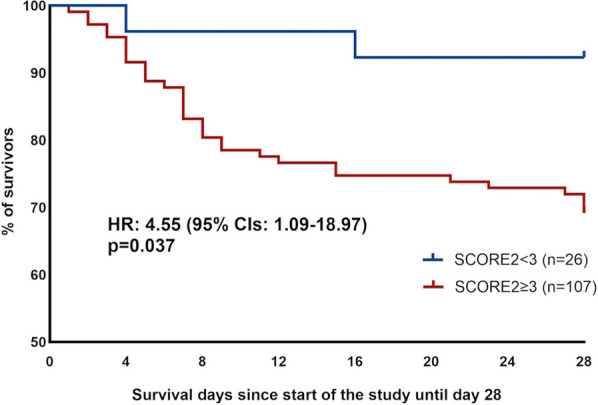
28-day mortality by the degree of SCORE2.

## P213 Validation of a new data-driven SLOSR ICU efficiency measure in community-acquired pneumonia patients: a multicenter study of 45,866 admissions in 85 Brazilian units

### A Quintairos^1^, G Fonte^2^, GF Ferrari^2^, L Santos Lourenço Bastos^2^, S Hamacher^2^, F Augusto Bozza^2^, I Tona Peres^2^, JI Figueira Salluh^3^

#### ^1^D’Or Institute for Research and Education (IDOR), Rio de Janeiro, Brazil; Department of Critical Care and Postgraduate Program in Translational Medicine, Rio de Janeiro, Brazil; Department of Critical and Intensive Care Medicine, Academic Hospital Fundación Santa Fe de Bogota, Bogota, Colombia, ^2^Department of Industrial Engineering, Pontificial Catholic University of Rio de Janeiro, Rio de Janeiro, Brazil, ^3^D’Or Institute for Research and Education (IDOR), Rio de Janeiro, Brazil, Rio de Janeiro, Brazil

*Critical Care* 2024, **28(Suppl 1):** P213

**Introduction:** Improving patient outcomes with optimal resource use is a major challenge.The Standardized Resource Use (SRU) is an indicator widely applied to analyze ICU efficiency [1]. However, it is well-known that when evaluating some specific conditions, traditional scoring systems tend to do worse [2]. Community-acquired pneumonia (CAP) remains a leading cause of morbidity and mortality, with many aspects of treatment to be better understood [3]. Recently, a multicenter study has developed and validated a data-driven methodology to evaluate ICU efficiency using machine learning (ML) techniques with better calibration and more accuracy [2,4]. The SLOSR (Standardized Length of Stay Ratio) provides a more reliable and potentially actionable indicator to assess ICU performance [5]. This work aims to validate the SLOSR in a subgroup of CAP patients.

**Methods:** We included 45,866 CAP admissions, from January 2019 to December 2023, in 85 general ICUs of a Brazilian hospital network. We proposed the SLOSR to evaluate ICU efficiency in this subgroup of patients, which uses the prediction model proposed by Peres et al. [4]. To demonstrate SLOSR models, we used R2, calibration plots, and funnel plots.

**Results:** The calibration showed a high concordance between observed and expected ICU LoS estimates per ICU for SLOSR (R^2^ = 0.89) (Figure). In the funnel plot, the median SLOSR was 1.00 (0.8–1.23) (Figure). Seven ICUs were outside the higher 99% control limit. SLOSR presented a high concordance to obtain a standardized metric for ICU efficiency in a specific subgroup population of CAP.

**Conclusions:** The SLOSR is a new metric that can be used to evaluate ICU efficiency in subgroup of CAP patients with high calibration and accuracy.

**References**

1. Rothen HU et al. Intensive Care Med 2007;33:1329–36.
2. Quintairos A et al. Intensive Care Med 49(2):223–225
3. Niederman MS et al. Eur Respir Rev. 2022 Dec 14;31(166):220123.
4. Peres IT et al. Anaesth Crit Care Pain Med 2022;41:101142.
5. Peres IT et al. Intensive Care Med 2023.

**Figure (abstract P213):**
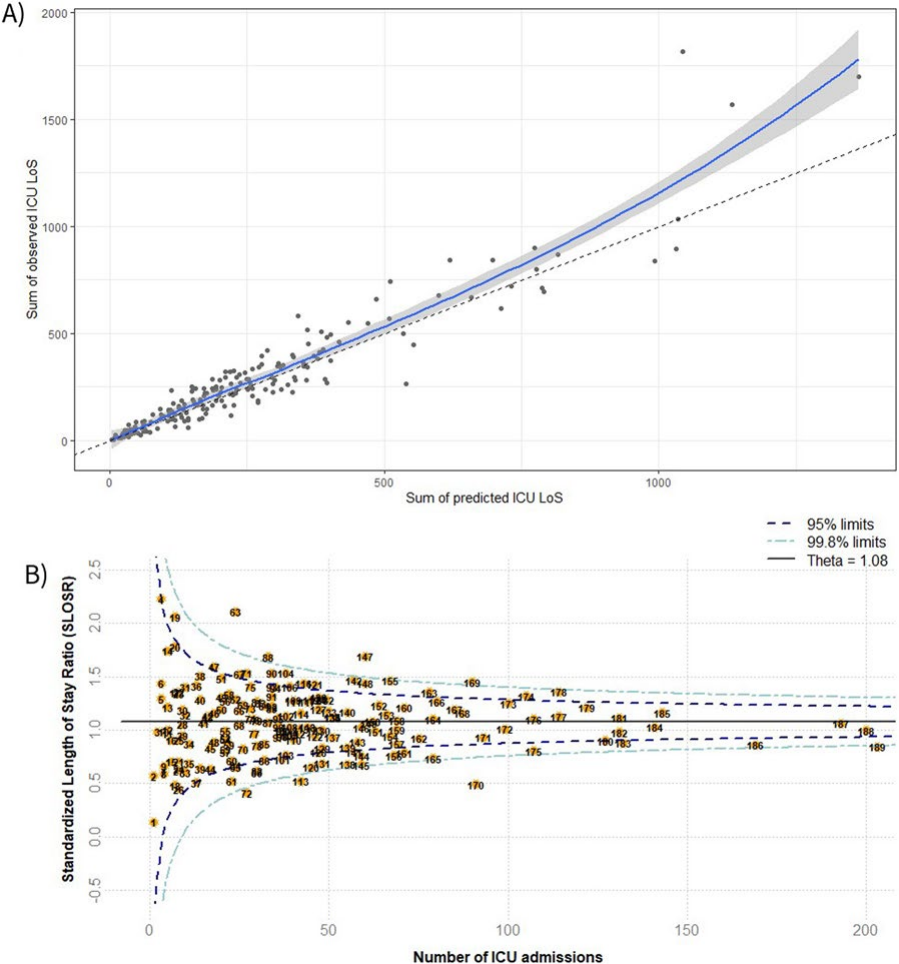
**A** Calibration of SLOSR model in terms of observed and predicted LoS in ICU level for ICU CAP patients. **B** Funnel plot of SLOSR for ICU CAP patients.

## P214 A breath-based in vitro diagnostic assay for the detection of lower respiratory tract infections

### DC Chen^1^, M Mirski^2^, SC Chen^3^, EAC Canton^1^, KMK Kiser^1^, CRH Haddaway^1^, MSC Cetta^1^, YP Pan^4^, WAB Bryden^1^, MM McLoughlin^1^

#### ^1^Zeteo Tech Inc, Biomedical Engineering, Sykesville, USA, ^2^Johns Hopkins Medicine, Anesthesiology & Critical Care, Baltimore, USA, ^3^University of Maryland, Biostatistics, College Park, USA, ^4^Maryland Psychiatric Research Center, Psychiatry, Baltimore, USA

*Critical Care* 2024, **28(Suppl 1):** P214

**Introduction:** An accurate diagnosis is critical to reducing mortality in people with lower respiratory tract infections (LRTIs) which have been recognized as the fifth-leading cause of mortality globally. Current microbiological culture is time-consuming, and nucleic acid amplification-based molecular technologies cannot distinguish between colonization and infection. Previously, we described the development of a sampling system for effectively capturing biomolecules from human breath, and identified a new class of proteoform markers of protease activation, termed proteolytic products of infection (PPI), for potentially detecting LRTIs [1].

**Methods:** Here, we further present an in vitro assay by designing a specific substrate-sensor to human neutrophil elastase (HNE), which is highly up-regulated in LRTIs. The sensor is an HNE substrate that is cleaved by the enzyme with higher sensitivity and specificity, yielding higher resolution. We then applied this in vitro assay to breath samples collected from both intubated patients and healthy volunteers (Figure panels A–B).

**Results:** The findings revealed that the LRTI group demonstrated a significant mean differential, showing a 9.8-fold elevation in measured HNE as compared to the non-LRTI group, and a 9.2-fold compared to healthy volunteers (Figure panels C–D). The in vitro assay's diagnostic potential for LRTIs was assessed by constructing an ROC curve, resulting in an AUC value of 0.987 (Figure panel E). Using an optimal threshold for HNE at 0.2 pM, the sensitivity was determined to be 1.0 and the specificity to be 0.867. Further correlation analysis revealed a strong positive relationship between the measured HNE-activity and the protein concentration in the breath samples.

**Conclusions:** Our results demonstrate that this breath-based, in-vitro assay provides high diagnostic performance for LRTIs, suggesting that the technology may be useful in the near term for the accurate diagnosis of LRTIs in critical care.

**Reference**

1. Chen D et al. Breath Res. 2000;15:16001.

**Figure (abstract P214):**
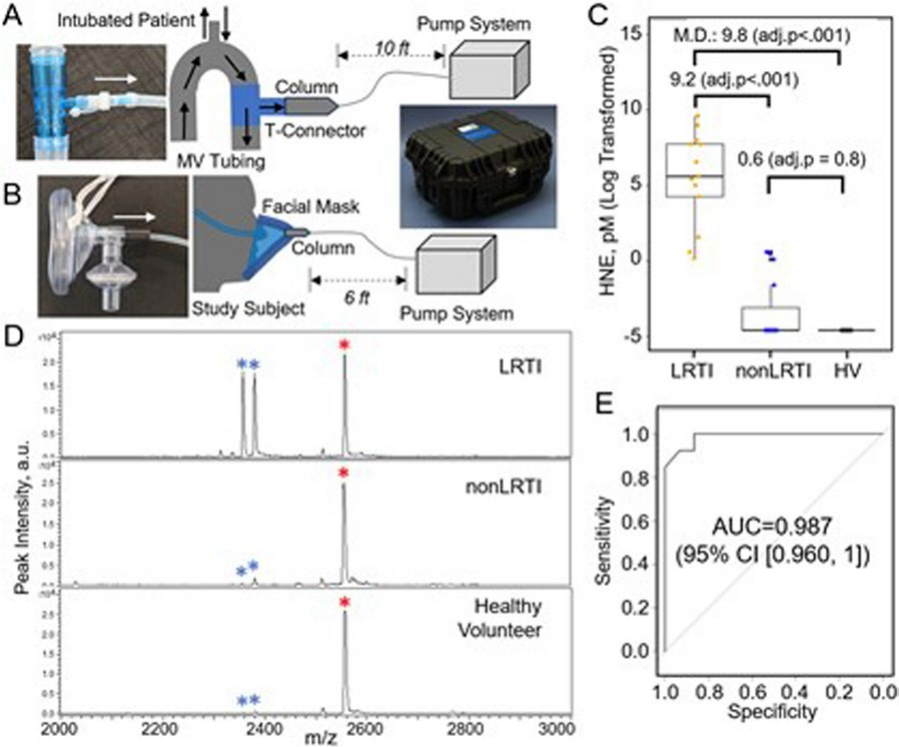
(**A**, **B**) Schematic of breath collection from intubated patients on mechanical ventilation in ICU and from healthy volunteers using a facial mask. **C** Boxplot display of the distribution of natural-log-transformed HNE measurements. **D** Representative MALDI-TOF spectra of breath samples collected from different groups. **E** Receiver operating characteristic (ROC) curve analysis for the in vitro assay performance.

## P215 Sepsis golden hour algorithm: impact on outcomes

### M Vivekanandan, B Rowland-Jones, D Cottam, M Judd, J Sheehan, P Bhogal, M Zuleika

#### Royal Surrey NHS Foundation Trust, Intensive Care Unit, Guildford, UK

*Critical Care* 2024, **28(Suppl 1):** P215

**Introduction:** Sepsis is associated with significant morbidity and mortality. The aim of this audit was to assess the impact of the introduction of ‘the Sepsis Golden Hour Algorithm’ on outcomes of patients admitted to the ICU with sepsis and compare with previous prospective audits done between 19/03/19 and 19/11/19 (2019).

**Methods:** Prospective re-audit of patients admitted to the ICU at the Royal Surrey NHS Foundation Trust from 01/08/21 to 31/03/22 (2021) with a diagnosis of sepsis as per Sepsis 3 definition [1]. The following parameters were compared with previous audit data: administration of antibiotics within 1 h of sepsis diagnosis, associated organ dysfunction and ICU and hospital outcomes. Appropriate statistical analysis was applied.

**Results:** Comparative analysis of both audits in the Table. Further comparison of results between 2021 and 2019 audits respectively were as follows: The number of male patients were 41 (64%) versus 71 (60%). Blood cultures were done in 47 (74%) versus 40 (44%) patients and sepsis 6 was delivered in 26 (41%) versus 61(51%) patients within an hour. Senior review was done within 2 h in 35 (55%) versus 63 (53%) patients. Vasopressors were administered in 48 (75%) versus 96 (81%) patients. Noninvasive ventilation was required in 21 (33%) versus 43 (36%) and Invasive Ventilation in 14 (22%) versus 38 (32%) patients. Renal replacement therapy was delivered to 7 (11%) versus 18 (15%) patients.

**Conclusions:** The number of patients who had received antibiotics (94%) substantially increased when compared to previous audit (74%). This could have had an impact on the decrease in length of ICU and hospital (*p* = 0.002) stay and also the reduction in hospital mortality from 28.6% to 18.75% (*p* = 0.003). Compliance to the administration of timely antibiotics was enabled by the Sepsis Golden Hour Algorithm but there was still room for improvement with the other components of the Sepsis 6.

**Reference**

1. Singer M et al. JAMA. 2016;315:801–810

**Table (abstract P215):** Comparative analysis of audits

Variable	Aug 21–March 22 (2021)	March 19–Nov 19 (2019)	*p* value
Number of patients	64	119	
Mean age (years)	65.5 (SD 14.243), range: 19–90	65.7 (SD 14.470) Range: 18–96	
Antibiotics within 1 h	62 (94%)	88 (74%)	
Mean length of stay in ICU (days)	5.1 (SD 6.286), range: 0–38	7.689 (SD 9.847) Range: 0–47	*p* = 0.06
Mean length of stay in hospital (days)	16.66 (SD 16.123), range: 0–84	26.317 (SD26.455) Range: 1–149	*p* = 0.002
ICU mortality	12 (18.75%)	19 (16%)	*p* = 0.486
Hospital mortality	12 (18.75%)	34 (28.6%)	*p* = 0.003

## P216 Precision medicine in septic shock with enibarcimab: biomarker guided definition of target population

### P Pickkers^1^, D Van Liers^2^, C Knothe^3^, J Struck^3^, S Witte^3^, PF Laterre^4^, A Mebazaa^5^

#### ^1^Radboud University Medical Center, Intensive Care, Nijmegen, Netherlands, ^2^Radboud University Medical Center, Department of Intensive Care Medicine and Radboud Center for Infectious Diseases, Nijmegen, Netherlands, ^3^Adrenomed AG, Adrenomed AG, Hennigsdorf, Germany, ^4^Centre Hospitalier Régional Mons-Hainaut, Department of Intensive Care Medicine, Mons, Belgium, ^5^University Hospitals Saint-Louis–Lariboisière, Department of Anaesthesiology, Burn and Critical Care, Paris, France

*Critical Care* 2024, **28(Suppl 1):** P216

**Introduction:** Enibarcimab is a non-neutralizing monoclonal anti-adrenomedullin (ADM) antibody, aimed to improve endothelial barrier function in septic shock. AdrenOSS-2 was a double-blind, randomized, placebo-controlled, biomarker-guided, phase II trial of enibarcimab in septic shock patients. Elevated bio-ADM plasma concentration at baseline (> 70 pg/mL) was required for inclusion. Circulating dipeptidyl-peptidase 3 (cDPP3) measured in a central lab was used to exclude patients unlikely to respond to enibarcimab treatment. Omission of patients with cDPP3 > 70 ng/mL at baseline was prespecified in the statistical analysis plan for the assessment of 28-day mortality.

**Methods:** All-cause mortality for 28-day follow-up was evaluated and Kaplan–Meier plots were generated comparing enbarcimab (combined doses) vs. placebo as specified in the trial protocol and statistical analysis plan.

**Results:** 301 patients were enrolled in the full analysis population, 35 (23.5%) and 42 (27.6%) patients died in the enibarcimab and placebo group, respectively (HR 0.84, *p* = 0.439). In the prespecified subgroup of cDPP3 ≤ 70 ng/mL (n = 269 patients), 28-day mortality tended to decrease to 18.0% (n = 23) in the enibarcimab group, whereas mortality rate in the placebo group remained 26.2% (n = 37) resulting in a HR of 0.65 (*p* = 0.106) (Figure). Treatment groups remained balanced across all demographic and baseline characteristics in the prespecified subgroup. The safety profile also remained unchanged regarding number and frequency of adverse events (AE). The positive treatment effect of enibarcimab improved further when cDPP3 thresholds of ≤ 50 ng/mL (n = 249) and ≤ 30 ng/mL (n = 195) were applied, resulting in a significant reduced 28-day mortality with enibarcimab in the subgroup of DPP3 ≤ 30 ng/mL (HR 0.31, *p* = 0.004).

**Conclusions:** These previously unpublished data from the AdrenOSS-2 trial show that a precision medicine approach using bio-ADM and cDPP3 for patient selection can overcome patient heterogeneity in septic shock leading to improved mortality.

**Figure (abstract P216):**
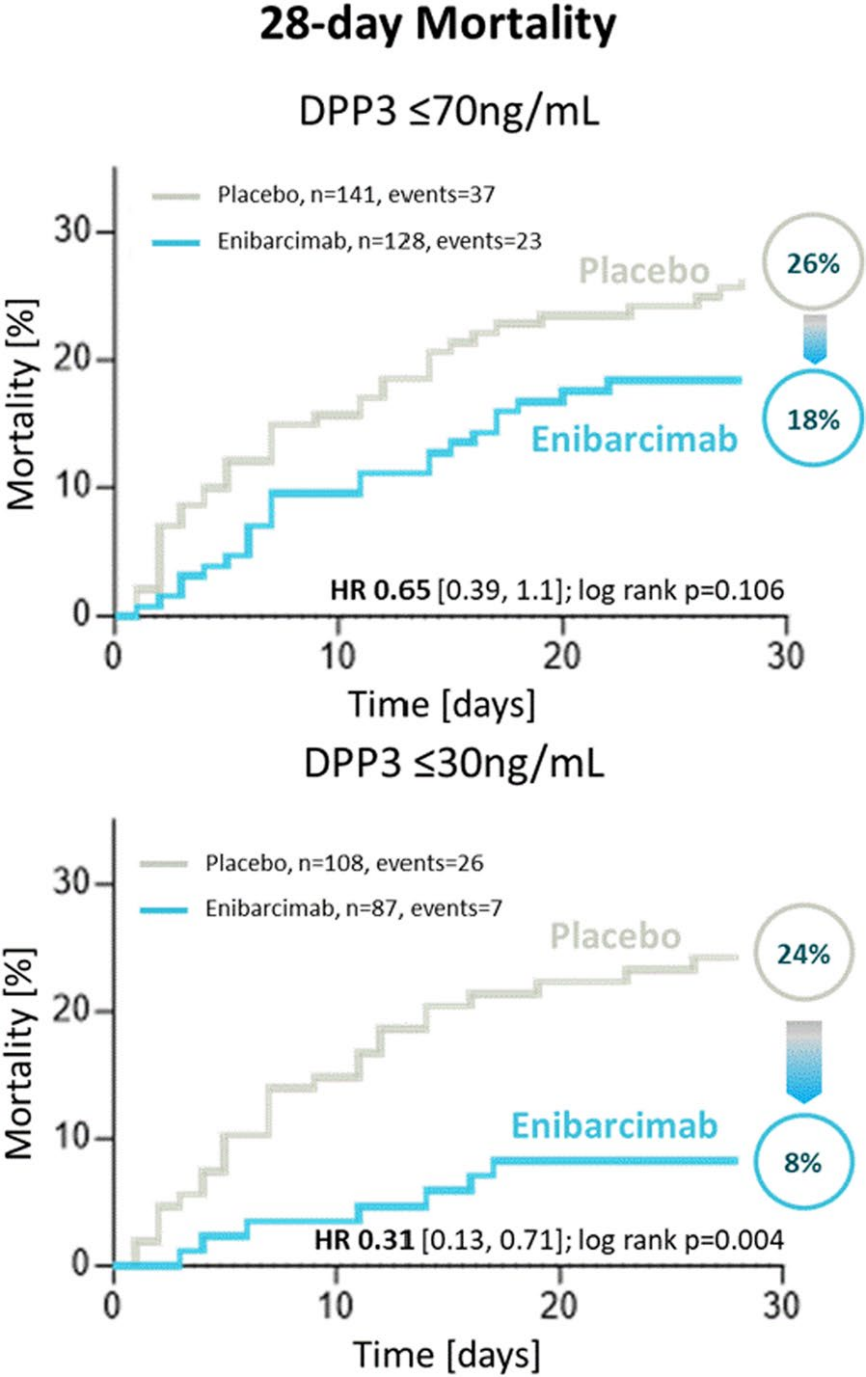
Kaplan–Meier of 28-day mortality after enibarcimab/placebo infusion, based on circulating dipeptidyl peptidase 3 (cDPP3) stratification. Data are displayed for the prespecified subgroup of cDPP3 ≤ 70 ng/mL (top) and the subgroup of cDPP3 ≤ 30 ng/mL (bottom). Both enibarcimab dosing groups were combined.

## P217 Modulation of NET release by plasma derived immunoglobulins: trimodulin (polyvalent IgM, IgA, IgG solution) vs. standard IVIg

### R Zapf^1^, U Steffen^1^, F Bohländer^2^

#### ^1^Friedrich-Alexander University (FAU) Erlangen-Nürnberg and Universitätsklinikum Erlangen, Department of Internal Medicine 3 - Rheumatology and Immunology, Erlangen, Germany, ^2^Biotest AG, Translational Research, Dreieich, Germany

*Critical Care* 2024, **28(Suppl 1):** P217

**Introduction:** The release of neutrophil extracellular traps (NETs) represents an important mechanism of pathogen defense by neutrophils. However, in case of dysregulated immune responses (as in severe lung infectious diseases), overwhelming NET formation can be detrimental [1]. Trimodulin is a polyvalent immunoglobulin preparation (~ 23% IgM, ~ 21% IgA and ~ 56% IgG) in development for the treatment of hospitalized patients with severe respiratory tract infections (e.g. sCAP). The immunomodulatory activity of trimodulin on neutrophils was shown in vitro and by analysis of clinical trial data [2]. The aim of this study is to investigate if modulation of NET release could be another relevant immunomodulatory mode of action for trimodulin.

**Methods:** Neutrophils were isolated from healthy donors and NET release was induced by PMA or immune complexes (heat aggregated IgA or IgG). Trimodulin, IVIg or buffer were added and NET release was measured by cytox green fluorescence intensity. Furthermore end-point measurements of NET markers were performed.

**Results:** The addition of trimodulin to PMA- or immune complex stimulated neutrophils leads to a strong dose-dependent reduction of NET release (up to 57% reduction in amount of external DNA compared to untreated control), while standard IVIg has only little effect. Mechanistically trimodulin and standard IVIg seem to condense the NETs, but there are no distinct effects on the measured NET markers.

**Conclusions:** The results of this in vitro study demonstrate that modulation of NET release may be a viable mode of action for trimodulin, which may be relevant in the treatment of severe lung infectious diseases. The superior immunomodulatory effects of trimodulin on neutrophils could be mediated by the additional IgM and IgA antibodies within trimodulin. Further studies are needed to unravel the mechanism and clinical relevance of these findings.

**References**

1. Porto BN et al. Front Immunol. 2016;7:311
2. Singer M et al. Crit Care. 2023;27:436

## P218 Probiotics as modifiers of the innate immune response in COVID-19 and ARDS: a case–control study

### I Mitrou, C Psarrakis, N Antonakos, K Leventogiannis, E Giamarellos-Bourboulis

#### National and Kapodistrian University of Athens, 4th Department of Internal Medicine, Medical School, Athens, Greece

*Critical Care* 2024, **28(Suppl 1):** P218

**Introduction:** There is controversy if probiotic intake may be beneficiary or not for patients with COVID-19. We hypothesized that innate immune responses in COVID-19 may differ by severity and modulated by probiotics.

**Methods:** Participants with confirmed COVID-19 infection were enrolled in the study between March 2020 and December 2021 and classified into patients with and without ARDS (acute respiratory distress syndrome). Peripheral blood mononuclear cells (PBMCs) are isolated and stimulated with probiotics. The studied probiotics were a commercially available preparation of *Saccharomyces boulardii*, *Bifidobacterium lactis* BB-12, *Lactobacillus acidophilus* LA-5 and *Lactobacillus plantarum* (Lactolevure, UniPharma, Athens, Greece). The concentrations of tumour necrosis factor (TNF)alpha, interleukin (IL)-1beta, and IL-6 were determined in cell supernatants by enzyme-linked immunosorbent assay. The same experimental procedure was performed for healthy volunteers.

**Results:** Experiments were done with 20 healthy volunteers, 19 patients without ARDS and 39 patients with ARDS. Results showed that probiotics do not change the production of IL-6 but decrease the production of TNFalpha and IL-1beta (Figure).

**Conclusions:** Results suggest that probiotics may modulate the production of some of the pro-inflammatory cytokines in severe COVID-19.

**Figure (abstract P218):**
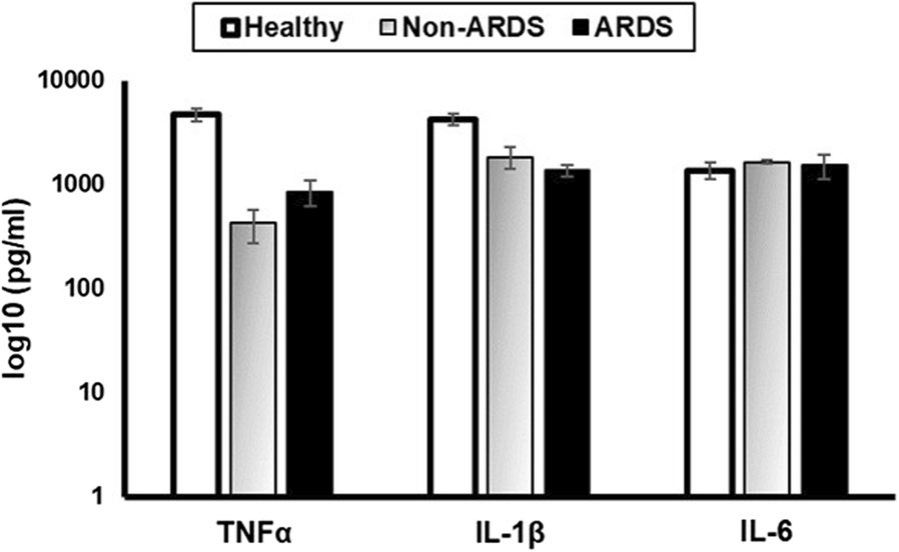
Cytokine production capacity by PBMCs in the presence of probiotics.

## P219 Tocilizumab timing and COVID-19 mortality: a cohort study of early vs late administration

### F Macgregor^1^, A O´Prey^2^, C Caulfield^2^, P MacTavish^3^, R Lowrie^4^, P Henderson^5^

#### ^1^Royal Alexandra Hospital, Pharmacy Department, Paisley, UK, ^2^Queen Elizabeth University Hospital, Pharmacy Department, Glasgow, UK, ^3^Glasgow Royal Infirmary, Pharmacy Department, Glasgow, UK, ^4^Pharmacy and Prescribing Support Unit, Glasgow, UK, ^5^Royal Alexandra Hospital, Intensive Care Unit, Paisley, UK

*Critical Care* 2024, **28(Suppl 1):** P219

**Introduction:** The optimal timing of tocilizumab treatment during the disease course of COVID-19 has yet to be adequately defined in the context of randomised controlled trials, and the effect of tocilizumab on real-world populations remains unclear. We examined the effect of different timing of tocilizumab, on mortality, in a cohort of adults with COVID-19.

**Methods:** All adults (≥ 18 years old) with confirmed COVID-19 admitted to four hospitals in the West of Scotland between 8th Jan 2021 and 31st March 2021 and who received tocilizumab were included in a retrospective cohort study. Patients were assigned to either an early (day 0 or day 1 of admission) or late (days 2–7 of admission) cohort based on tocilizumab initiation. The primary outcome was 90 day all-cause mortality, in early versus late cohorts. Secondary outcomes were 28 and 180 day all-cause mortality.

**Results:** 203 patients were included (138 in the early cohort and 65 in the late cohort). Mortality was significantly higher in the late cohort compared to the early cohort (adjusted OR 3.33; CI 1.29 to 8.54; *p* = 0.012). The secondary outcomes demonstrated the same effect with higher rates of death at 28 days (late cohort adjusted OR 3.28; CI 1.23 to 8.75; *p* = 0.018) and 180 days (late cohort adjusted OR 3.70; CI 1.45–9.45; *p* = 0.006). This effect was seen whether the outcome was adjusted or unadjusted. Mortality at 90 days in the early cohort was 22% (n = 30) compared to 45% (n = 29) in the late cohort (*p* < 0.001) (Figure).

**Conclusions:** Early administration of tocilizumab within the first 2 days of hospitalisation was associated with significant survival benefit compared to late exposure. Late administration was associated with particularly high mortality.

**Figure (abstract P219):**
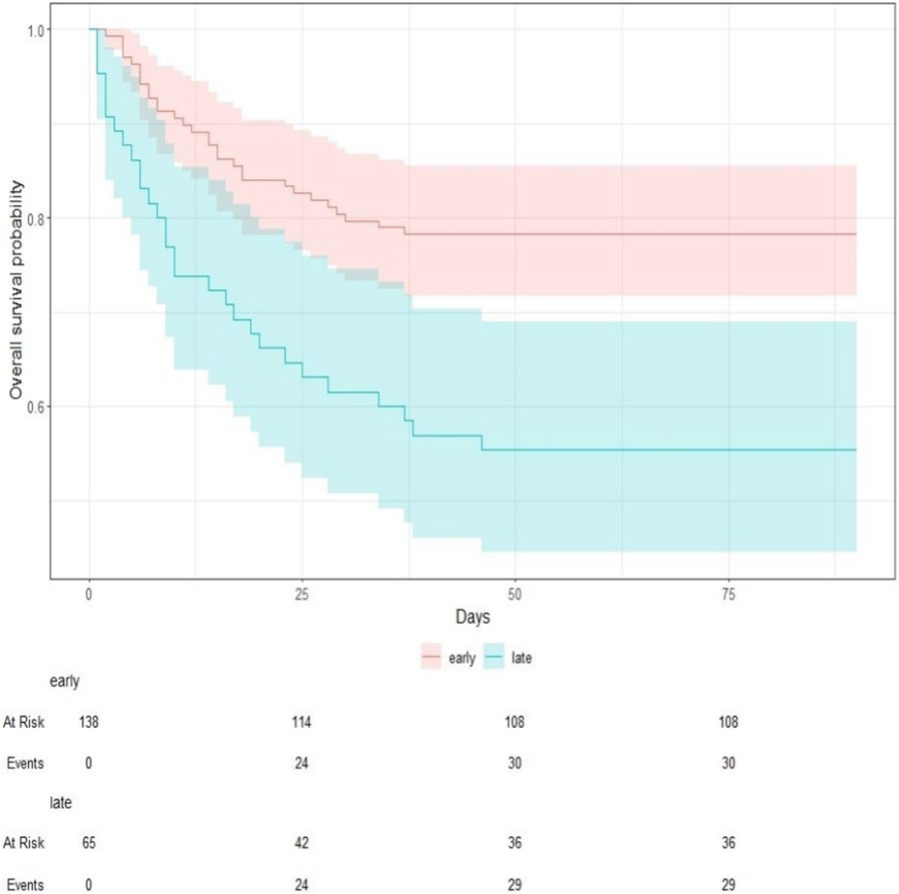
Kaplan–Meier curve for survival through 90 days.

## P220 Hemoperfusion with Efferon LPS NEO device reverts multiple organ failure, decreases IL-6 and TNF levels and systemic immune inflammation in children with sepsis

### SM Stepanenko^1^, II Afukov^2^, YS Aleksandrovich^3^, EV Zilbert^4^, AB Chatchukhina^2^, IV Turischev^5^, MA Rusak^6^, AL Shavkin^6^, AY Popov^7^, V Pisarev^8^

#### ^1^N.I. Pirogov Russian National Research Medical University, Moscow, Russian Federation, ^2^G.N. Speransky Children’s City Clinical Hospital No. 9, Moscow, Russian Federation, ^3^Saint-Petersburg State Pediatric Medical University, Saint Petersburg, Russian Federation, ^4^N.F. Filatov Children’s City Hospital, Moscow, Russian Federation, ^5^Saint Vladimir Children’s City Clinical Hospital, Moscow, Russian Federation, ^6^St. Petersburg State Health Care Institution "Children’s Municipal Multi-Specialty Clinical Center of High Medical Technology", Saint Petersburg, Russian Federation, ^7^A.N. Nesmeyanov Institute of Organoelement Compounds of Russian Academy of Sciences, Moscow, Russian Federation, ^8^Federal Research and Clinical Center of Intensive Care Medicine and Rehabilitology, V.A.Negovsky Institute of General Reanimatology, Moscow, Russian Federation

*Critical Care* 2024, **28(Suppl 1):** P220

**Introduction:** Pediatric sepsis is a major life threat due to multiple organ failure (MOF) progression and resistance to conventional therapies. Extracorporeal hemoperfusion (EH) using Efferon LPS adsorber abrogates MOF and halts septic shock (SS) in adult patients [1]. Here, we report case series of children with sepsis to assess the feasibility and safety of EH with a new pediatric Efferon LPS NEO device (PE).

**Methods:** PE contains porous polymeric scaffold with the surface-immobilized LPS-selective ligand. Seventeen children (9 girls) with sepsis (10 with SS), average age and range: 26 (1–159 months), were included in the study following Ethical Committee approval. Children received two treatments, lasted ≥ 4 h, 24 h apart, with PE. The blood cell count, levels of C-reactive protein (CRP), procalcitonin (PCT), creatinine, urea, ALT and AST were monitored on days 0, 1, 2, 3, 5, 7 and 14. Plasma IL-1b, IL-6 and TNF were determined on days 0 and 3 after the inclusion into the study. STATA 16.0 (StataCorp, USA) were used to assess significance.

**Results:** In SS patients, EH resulted in: (a) early abrogation of shock as confirmed by a decrease in vasopressors dose (Figure panel D), (b) MOF reversal as determined by pSOFA (*p* = 0.003, Figure panel A), (c) lower creatinine (*p* = 0.006, Figure panel C) and C-reactive protein levels starting from days 2–3, (d) increases in PaO_2_/FiO_2_ index on days 7 and 14 (*p* = 0.004, Figure panel B). Children exhibited significantly decreased cytokines levels concentration in 72 h post-EH (Figure panels F and G). In non-SS children, the cell ratio-based biomarkers exhibited early vs late prognosis for SII and NLR (Figure panels I and H, respectively). Decreased thrombocyte levels on days 1–3 post-EH were normalized on day 7. Only 2 of 17 most severely ill children with refractory SS have deceased by the 60 day.

**Conclusions:** These results argue for feasibility and safety of EH with PE in pediatric sepsis.

**Reference**

1. Rey et al. Shock. 2023;59:846–854

**Figure (abstract P220):**
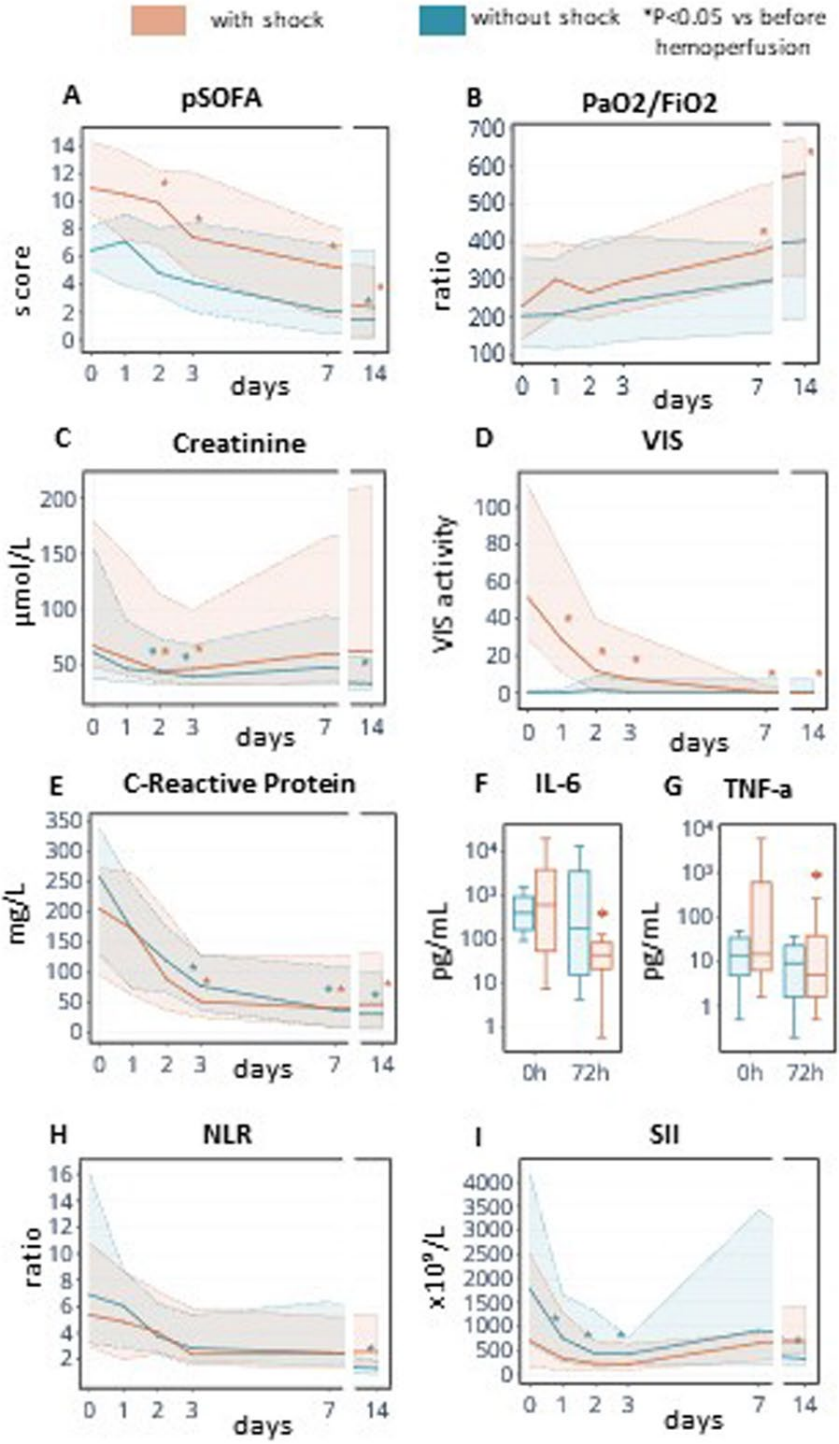
Clinically relevant effects of hemoperfusion with Efferon LPS NEO in children with sepsis. Notes: *p < 0.05 versus day 0. VIS—vasoactive-inotropic score (summation of all administered inotropes); NLR—neutrophil /lymphocyte counts ratio; SII—systemic immune inflammation index: platelet × neutrophil/lymphocyte counts.

## P221 The international, prospective COSMOS registry on the use of CytoSorb® in critically ill patients: preliminary results after first 100 patients

### A Kribben^1^, M Thielmann^2^, FS Taccone^3^, JL Teboul^4^, FM Brunkhorst^5^, G Bottari^6^, J Hidalgo^7^, T Klaus^8^, EN Deliargyris^9^, R Ferrer^10^

#### ^1^Clinic for Nephrology, University Duisburg-Essen, University Hospital Essen, Essen, Germany, ^2^Department of Thoracic- and Cardiovascular Surgery, Westgerman Heart & Vascular Center, Essen, Germany, ^3^Department of Intensive Care, Hôpital Universitaire de Bruxelles (HUB), Université Libre de Bruxelles (ULB), Brussels, Belgium, ^4^Therapeutics and Critical Care Medicine, Medical ICU, Bicetre Hospital, Paris-Saclay University, Paris, France, ^5^Integriertes Forschungs- und Behandlungszentrum (IFB) Sepsis und Sepsisfolgen, University Jena, Jena, Germany, ^6^Pediatric Intensive Care Unit, Children Hospital Bambino Gesù, Rome, Italy, ^7^General Intensive Care Unit and COVID-19 Unit, Belize Healthcare Partners, Belize City, Belize, ^8^CytoSorbents Europe GmbH, Berlin, Germany, ^9^CytoSorbents Corporation and CytoSorbents Medical Inc., Princeton, New Jersey, USA, ^10^ Intensive Care Department, Vall d´Hebron University Hospital, Shock, Organ Dysfunction and Resuscitation Research Group (SODIR), Barcelona, Spain

*Critical Care* 2024, **28(Suppl 1):** P221

**Introduction:** The international COSMOS (**C**yt**OS**orb® Treat**M**ent **O**f Critically Ill Patient**S**) registry tracks utilization patterns and clinical outcomes of the CytoSorb® hemoadsorption device in real-world critical care settings.

**Methods:** Data is collected at various points: before and during CytoSorb®, 24 h after treatment, at Intensive Care Unit (ICU) and hospital discharge, and day 90 (+ 20). T-tests or Wilcoxon rank sum tests are used for continuous variables and data are presented as mean ± standard deviation or median [interquartile ranges].

**Results:** A total of 100 patients (30% female, age 58.6 ± 15.89) from 12 sites were included in this analysis. CytoSorb® was applied for various critical care indications (Figure), mean number of used adsorbers was 2.5 ± 2.14. Mean APACHE II was 23.5 [15.0, 30.0] and SOFA score 12.0 [9.0, 14.0] over the whole cohort with an actual ICU-mortality rate of 40.4% and a median ICU stay of 17 [10.0, 27.0] days. In the sepsis sub-cohort median SOFA score before CytoSorb® Therapy was 13.0 [11.0, 15.0] whereas actual ICU-mortality was 41.5%, lower than expected according to SOFA score. The platform used for integration of CytoSorb® was continuous renal replacement therapy (CRRT) (70.8%), standalone hemoperfusion (13.5%), extracorporeal membrane oxygenation (ECMO) (12.4%), and the remaining slow low efficiency dialysis (SLED) or intermittent hemodialysis. Compared to baseline significantly lower plasma levels for lactate (*p* < 0.0001) and creatinine (*p* < 0.0001) were observed after CytoSorb® treatment whereas albumin did not change (*p* = 0.315). Mean norepinephrine (NE) dosage went down significantly from 0.909 to 0.386 µg/kg/min (*p* = 0.030) and NE/MAP ratio from 0.012 to 0.005 (*p* = 0.033).

**Conclusions:** The International COSMOS Registry delivers real world data and depicts a broad variety of indications and platforms for integration of the device. Lactate, creatinine, and need for norepinephrine decreased significantly during CytoSorb® treatment.

**Figure (abstract P221):**
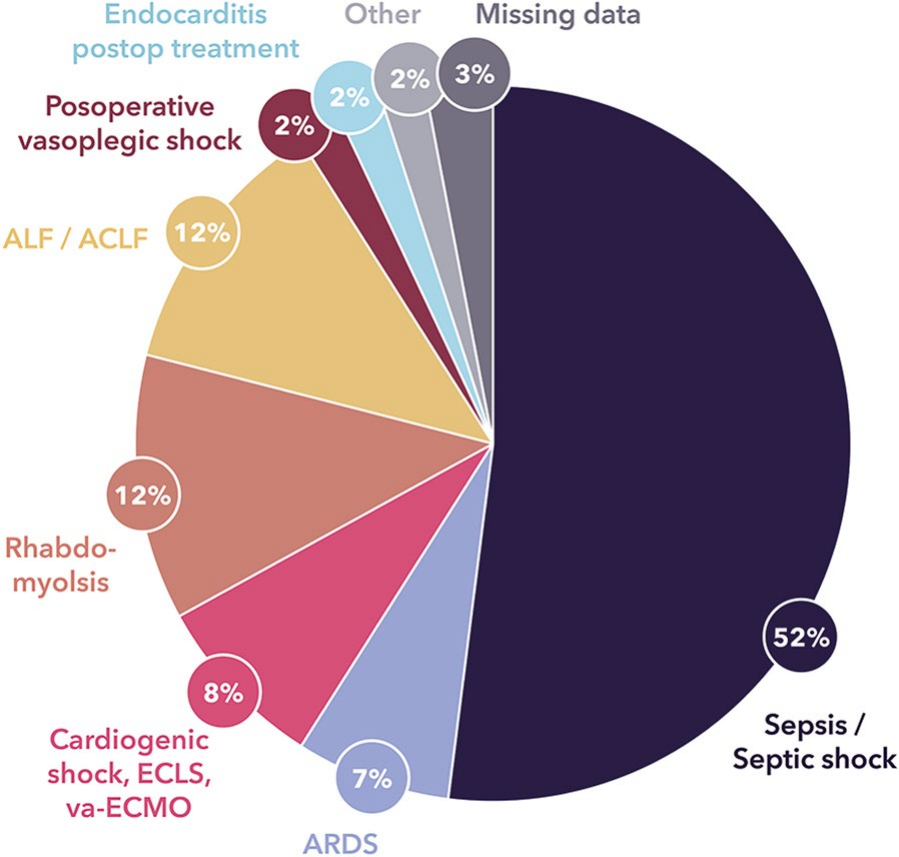
Number* and type of indications for CytoSorb® Therapy (*multiple indications may apply for certain patients).

## P222 Mortality comparison between positive single-center randomized trials and subsequent multicenter randomized trials in critically ill patients: a systematic review

### Y Kotani^1^, S Turi^2^, A Ortalda^2^, M Baiardo Redaelli^2^, C Marchetti^2^, R Losiggio^2^, G Lombardi^2^, N Maimeri^2^, G Landoni^2^, R Bellomo^3^

#### ^1^Kameda Medical Center, Department of Intensive Care Medicine, Kamogawa, Japan, ^2^IRCCS San Raffaele Scientific Institute, Department of Anesthesia and Intensive Care, Milan, Italy, ^3^The University of Melbourne, Department of Critical Care, Melbourne, Australia

*Critical Care* 2024, **28(Suppl 1):** P222

**Introduction:** It is unclear how often significant mortality reduction shown in single-center randomized controlled trials (sRCTs) involving critically ill patients are confirmed by subsequent multicenter randomized controlled trials (mRCTs). The aim of this systematic review was to evaluate if statistically significant mortality reduction of sRCTs were confirmed by subsequent mRCTs.

**Methods:** We searched PubMed for sRCTs published in the New England Journal of Medicine, JAMA, or Lancet, from inception until December 31, 2016. Studies reporting a statistically significant mortality reduction using any treatment (drug, technique, or strategy) among adult critically ill patients were included in this review. We then searched for subsequent mRCTs addressing the same research question of the sRCT, and compared the consistency between sRCTs and mRCTs in terms of mortality findings when any mRCT was available.

**Results:** We identified 19 sRCTs showing a significant mortality reduction in critically ill adults, with 16 sRCTs followed by at least one subsequent mRCT (24 trials in total). Among these 16 sRCTs, one (6%) was followed by a mRCT confirming a significant mortality reduction; 14 (88%) were followed by mRCTs with no significant mortality difference (Figure). The positive finding of one sRCT (6%) on intensive glycemic control was contradicted by a subsequent mRCT showing a significant mortality increase.

**Conclusions:** Significant mortality reduction shown by sRCTs is rarely replicated by subsequent mRCTs. The findings of sRCTs should be considered hypothesis-generating. Clinicians should wait for multicentric validation before changing practice.

**Acknowledgements:** This study was registered in the PROSPERO International Prospective Register of Systematic Reviews (CRD42023455362). This study was published in November 2023 (Crit Care 27:465).

**Figure (abstract P222):**
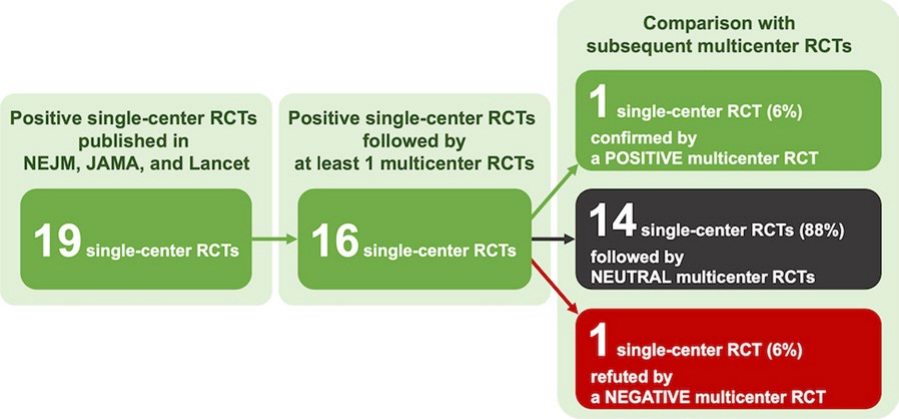
Mortality findings in multicenter randomized trials following positive single-center trials. RCT randomized controlled trials, NEJM New England Journal of Medicine.

## P223 Cardiac dysfunction is common prior to sepsis hospitalization

### S Iyer^1^, JN Kennedy^1^, JC Jentzer^2^, MH Senussi^3^, CW Seymour^1^

#### ^1^University of Pittsburgh, Critical Care Medicine, Pittsburgh, USA, ^2^Mayo Clinic College of Medicine and Science, Cardiovascular Medicine, Rochester, USA, ^3^Baylor College of Medicine, Cardiology & Critical Care Medicine, Houston, USA

*Critical Care* 2024, **28(Suppl 1):** P223

**Introduction:** We sought to characterize the heterogeneity in cardiac function prior to sepsis. While guidelines recommend urgent resuscitation in sepsis, optimization may require an understanding of pre-existing and sepsis-related cardiac dysfunction. Little is known about the epidemiology and impact of pre-existing dysfunction on sepsis resuscitation.

**Methods:** We studied patients from 2013 to 2014 at 12 UPMC hospitals. We included patients meeting Sepsis-3 criteria within 6 h of presentation and linked transthoracic echocardiogram (TTE) findings and chronic β-blocker therapy up to 12 months prior. Outcomes included ICU admission and length of stay, and mortality at 28-days up to 3 years. Continuous data were presented as means with standard deviations; categorical variables as frequencies and percentages (Stata 18.0).

**Results:** Among 513,281 patients, 31,052 met Sepsis-3 criteria within 6 h of arrival, of whom 4599 had TTE data (mean age 65 (SD: 15 years), 49.5% male, 84% White). One in five had pre-existing cardiac dysfunction (974 of 4599): 715 (73%) with left ventricular (LV), 136 (14%) with right ventricular (RV), and 123 (13%) patients with biventricular (BV) dysfunction (Table). Overall, 45% received chronic β-blocker therapy (2056 of 4599), and 26% had pre-existing cardiac dysfunction (526 of 2056). Despite similar clinical presentation of sepsis, ICU admission was greater in patients with ventricular dysfunction (83% v. 67%), and vasopressor administration more common (BV: 51%, LV: 36%, RV: 33%, none: 23%). Mortality was higher among those with cardiac dysfunction; for instance, 25% for BV versus 18% for no dysfunction at 28 days and 52% versus 43% at 12 months.

**Conclusions:** Pre-existing cardiac dysfunction was present in one in five sepsis patients with TTE data and over half received chronic β-blocker therapy. These patients experienced greater organ support, ICU admissions, and worse outcomes, despite similar sepsis presentations.

**Table (abstract P223):** Characteristics before and after sepsis by TTE abnormality in the 12 months prior to sepsis

Characteristic	None (N = 3625)	RV dysfunction (N = 136)	LV dysfunction (N = 715)	BV dysfunction (N = 123)
Age, years, mean (SD)	68 (15)	71 (14)	70 (15)	71 (13)
Sex, male, no. (%)	1889 (52.1%)	75 (55.1%)	262 (36.6%)	51 (41.5%)
White race, no. (%)	3066 (84.6%)	120 (88.2%)	582 (81.4%)	103 (83.7%)
Black race, no. (%)	462 (12.7%)	12 (8.8%)	106 (14.8%)	15 (12.2%)
Chronic β-blocker therapy in past 1 year, no. (%)	1530 (42.2%)	56 (41.2%)	402 (56.2%)	68 (55.3%)
Vasopressors during hospital stay, no. (%)	832 (23.0%)	45 (33.1%)	258 (36.1%)	63 (51.2%)
ICU admission during hospital stay, no. (%)	2432 (67.1%)	107 (78.7%)	538 (75.2%)	102 (82.9%)

## P224 The effects of vasopressin in combination with norepinephrine in cardiac function

### FA Gonzalez, J Bacariza, R Varudo, J Leote, I Ribeiro, R Meireles, C Martins, V Pereira, R Gomes, A Fernandes

#### Hospital Garcia de Orta, Intensive Care Medicine Department, Almada, Portugal

*Critical Care* 2024, **28(Suppl 1):** P224

**Introduction:** Low doses of vasopressin (V) may be added to NE to maintain arterial blood pressure in refractory septic shock and to decrease exposure to NE, according to the Surviving Sepsis Campaign (SSC) guidelines [1]. The aim of the work was to compare the effect of NE alone and NE/V combination on hemodynamics, tissue perfusion and cardiac function in septic shock patients.

**Methods:** Before-and-after non-experimental observational study, in septic shock patients: before, all patients received only NE; and after, all patients received a combination of NE and V, when NE dose was equal to or greater than 0.5mcg/kg/min, according to the SSC. The hemodynamic evaluation included echocardiography and a bioreactance device.

**Results:** 45 patients were included, 25 patients in the NE group and 20 patients in the NE/V group. 64.7% males, mean age of 68.2 years, with similar comorbidities and APACHE II scores. There was a decrease in the NE dose of 31.74% (*p* = 0.048) after 8–12 h of starting V in the NE/V group. There was a trend in the NE/V group towards higher mean arterial pressure, lower heart rate, and metabolic normalization (pH, base excess, and lactate), but it was not significant. The NE/V group had higher cardiac output (4.6 vs. 3.9 mL/min, *p* = 0.06) and higher left ventricular outflow tract velocity–time integral (LVOT-VTI) (19.6 vs. 17.9 cm, *p* = 0.04) than the NE group, despite having similarly left ventricular ejection fraction. There was no difference between the groups regarding mortality or other organ dysfunctions.

**Conclusions:** In patients with refractory septic shock, there was a statistically significant decrease in the dose of NA after starting vasopressin and a higher cardiac output and LVOT-VTI in the NE/V group, suggesting that lower NE doses with the combination of vasopressin can improve cardiac performance and hemodynamic profile.

**Reference**

1. Evans L et al. Intensive Care Med. 2021;47:1181–1247.

##  P225 A pilot randomised trial to compare live video versus document upload for clinical trial monitoring for the BALANCE (Bacteraemia Antibiotic Length Actually Needed for Clinical Effectiveness) trial

### D Dwivedi^1^, S Davies^2^, A Rishu^3^, N Daneman^4^, R Fowler^5^, B Rogers^2^

#### ^1^Monash Health, Intensive Care Unit/Infectious Diseases, Melbourne, Australia, ^2^Monash Health, Infectious Diseases, Melbourne, Australia, ^3^Sunnybrook Research Institute, Intensive Care Unit/Infectious Diseases, Toronto, Canada, ^4^Sunnybrook Research Institute, Infectious diseases, Toronto, Canada, ^5^Sunnybrook Research Institute, Intensive Care Unit, Toronto, Canada

*Critical Care* 2024, **28(Suppl 1):** P225

**Introduction:** The BALANCE trial is a multi-national, randomized trial of shorter duration (7 days) versus longer duration (14 days) antibiotic treatment for patients with bloodstream infections and has enrolled 3630 patients. Based on national guidelines, the trial monitored participating Australian sites using central monitoring and limited source data verification (SDV). Due to the COVID-19 pandemic source data verification was completed remotely. Aim—To compare two approaches of remote SDV for clinical-trial monitoring: (1) UPLOADing of copies of source-data documents to a cloud-based platform list with off-line SDV (2) LIVE SDV via a video link (e.g., zoom) between the site and trial monitor. Objective—To evaluate the time, acceptability, user pReference and assess the quality of the monitoring using the two approaches.

**Methods:** All Australian sites in the BALANCE trial were invited to participate in this study. Willing sites were randomised (1:1) between the two methods of SDV. Site staff (and the monitor) completed a worksheet recording key parameters of the SDV such as the time taken to locate and handle the documents. An anonymous survey of the staff members experience was also collected.

**Results:** Out of 18 sites in Australia, 59% of the sites participated in SDV via LIVE monitoring while 41% via UPLOADing of documents. Amongst the sites randomised to LIVE method, an equal number (44%) of participants prefer face to face monitoring or LIVE monitoring over UPLOAD method. In comparison, sites that were randomised to UPLOAD method, only 33% of participants prefer UPLOAD method than face to face. However, none of the participants prefer UPLOAD method than LIVE method. Results of only anonymous survey are demonstrated. Analysis of the quantitative measures of time spent collecting documents, number of source documents required etc. is ongoing and will be presented.

**Conclusions:** Remote monitoring via LIVE method is preferred than UPLOADing of documents by research staff participating in BALANCE trial.

## P226 The challenge of delayed intensive care unit admissions

### M Sheik, M Abbas, J Dixon

#### Epsom & St Helier University Hospital NHS Trust, Critical Care Department, Surrey, UK

*Critical Care* 2024, **28(Suppl 1):** P226

**Introduction:** We aimed to study the effects of delayed intensive care unit (ICU) admissions and the impact of a policy to ensure emergency bed capacity. There is a lack of consensus on whether delayed ICU admissions translate to mortality effects. Increasing ICU admission capacity to mitigate delays potentially risks lowering the threshold for admission to patients who are either too well or, conversely, too unwell to benefit from ICU care.

**Methods:** We conducted an observational study of two cohorts of patients which had delayed (DA) and non-delayed (Control) emergency admissions to ICU across 2 hospital sites. Delayed admissions were defined as admissions > 4 h. Prospective data were collected over five years before, and two years after, the policy change. Student t-test compared APACHE-2 scores and standardised mortality rate (SMR) pre and post policy.

**Results:** Prior to policy change, the results show a DA rate of 8.8%; similar APACHE-2 scores in controls (15.46 ± 6.89; n = 4320) versus DA (15.51 ± 7.02, n = 410), *p* = 0.89; increased mortality in DA vs .controls (26.5% vs. 13.1%); SMR was 0.86 ± 0.07 in controls versus 1.26 ± 0.41 in DA, *p* < 0.0001. This corresponds to an estimated 11.2 ± 6.8 potentially avoidable deaths per year in the DA group across both hospitals. Following the policy change, DA were eventually eliminated. Pooled data showed SMR reduced from0.90 ± 0.60 (n = 4730) prior to policy change, to 0.81 ± 0.11 (n = 1750) after change (*p* < 0.0001).

**Conclusions:** Delayed admissions have a statistically significant increased rate of mortality compared to non-delayed admissions even when mean APACHE-II scores are similar. Following the policy change a significant reduction in delayed admissions and decrease in mortality for all emergency admissions was observed. We conclude that spare emergency admission capacity in ICU has a mortality benefit and recommend the adoption of similar policies in other ICUs.

## P227 Effects of rotating shift work schedule on sleep parameters in healthy intensive care unit nurses

### A Ioannidis^1^, A Tsaloglidou^1^, C Papadopoulos^2^, N Pantelas^3^, D Komninou^3^, A Petsa^3^, K Founta^3^, C Sidera^3^, E Markidou^3^, T Kafkia^1^

#### ^1^International Hellenic University, School of Health Sciences, Department of Nursing, Sindos-Thessaloniki, Greece, ^2^Aristotle University, School of Medicine, Third Cardiology Department, Thessaloniki, Greece, ^3^General Anticancer Hospital "Metaxa", Intensive Care Unit, Piraeus, Greece

*Critical Care* 2024, **28(Suppl 1):** P227

**Introduction:** Rotating shift work schedules can disrupt the circadian rhythm and negatively affect sleep. This study aimed to investigate the effects of rotating shift work schedules on sleep parameters in a cohort of healthy nurses working in an intensive care unit (ICU).

**Methods:** A cross sectional observational study was conducted among a group of healthy nurses working in an ICU. Self-reported sleep quality was assessed using the Pittsburgh Sleep Quality Index (PSQI). Sleep parameters were collected with a sleep diary and a multi sensor smartwatch that has been shown to adequately estimate sleep parameters (Fitbit Charge 5, Google, USA). Measurements were collected for 24 h after a morning, an afternoon, or a night shift.

**Results:** The sample of the study consisted of 22 nurses (19 female and 3 male) with mean age 42 ± 8.2 years. The mean PSQI score was 7.6 ± 0.32, while only 8 (36.4%) nurses had a PSQI score ≤ 5 which corresponds to good sleep quality. In total, there were 46 Fitbit recordings included in the analysis (Table). Reported sleeping time on the sleep diary and the time asleep measured by the smartwatch showed strong correlation (Pearson r = 0.87, *p* < 0.05). Moreover, the sleep parameters calculated by the smartwatch showed significant differences among the shift types. Sleep duration was longer after a night shift which could be attributed to the sleep deficit of the night shift. As hypothesized, the recordings after a night shift revealed an even poorer quality of sleep.

**Conclusions:** The findings of this study suggest that rotating shift work schedules can negatively affect sleep parameters in healthy ICU nurses. Future research should focus on developing interventions to improve sleep quality and reduce the negative effects of shift work on health and well-being.

**Table (abstract P227):** Sleep parameters measured by Fitbit Charge 5

Parameter	Morning shift (n = 14)	Afternoon shift (n = 17)	Night shift (n = 15)	*p*
Time asleep (hours)	6.4	6.1	7.7	< 0.05
REM sleep cycles (n)	4.4	3.7	3.4	< 0.05
REM sleep duration (%)	22.8	18.5	14.6	< 0.05
Sleep score (%)	78	75	67	< 0.05

## P228 Emergency bag bleep test: How much weight is too much?

### AC Casey, J Wright, R Coe, R Jones

#### North Bristol NHS Trust, Intensive Care Medicine, Bristol, UK

*Critical Care* 2024, **28(Suppl 1):** P228

**Introduction:** The appropriate contents of emergency bags are widely debated but the impact of excessive weight is not considered [1]. We looked at the effects a lighter bag has on time to location and response times.

**Methods:** 15 volunteers were timed travelling 200 m, up 4 flights of stairs and back using our current bag (13.2 kg) and a stripped back bag (3.1 kg). We measured their response time, resting heart rates, heart rates on arrival and their processing time to ready equipment for intubation.

**Results:** The increase in time with the heavier bag compared to lighter ranged from + 35 s to − 32 s, with a 12 s average increase. Participants were on average 9 bpm more tachycardic carrying the larger bag (range − 11 bpm to + 47 bpm). Participants felt the larger bag was "horrible," "hard work," their legs were "burning," "shaking," and were "more likely to fall”. Processing time improved on average 2.2 s (range − 15 s to + 21 s) faster after the third time opening the bag, despite this being with the heavier bag.

**Conclusions:** A lighter bag improves response time, practitioner satisfaction and results in a lower heart rate at arrival. Processing time did not reduce but improved on repetition, which shows that familiarity and regular exposure is important for speed of response. Overall, a lighter and more familiar bag means a faster response.

**Reference**

1. Nowadly CD et al. Mil Med. 2023;188:e1678–84.

## P229 Does simulation training in an intensive care setting improve final-year medical students’ feelings of preparedness and level of competence?

### M Guinat^1^, JD Chiche^1^, Y Takeuchi^2^

#### ^1^Lausanne University Hospital (CHUV), Department of Intensive Care Medicine, Lausanne, Switzerland, ^2^Faculty of Biology and Medicine, Medical Education Unit of the School of Medicine of Lausanne, Lausanne, Switzerland

*Critical Care* 2024, **28(Suppl 1):** P229

**Introduction:** Undergraduate curricula should offer hands-on experiences related to acute care. Instructional models offer guidelines for developing effective learning strategies in an intensive care context. This study aimed to (1) determine the association between student’s level of preparedness to manage acutely ill patients and their level of competence before and after a simulation course, (2) explore how the design of the course influenced students’ feelings of preparedness.

**Methods:** Ten final-year medical students, while in an internship in the intensive care unit, participated in a one-month course designed with the 4C/ID model to learn acute care management [1]. Students’ feelings of preparedness and level of competence were measured before (pretest) and after (posttest) the course, as well as the correlation between these concepts. Then, two focus groups were conducted to provide a deeper understanding of the design factors of the course that affect feelings of preparedness.

**Results:** Students’ feelings of preparedness scores on the post-test (M = 37, SD = 2.81) were significantly higher than on the pretest (M = 32, SD = 4.53) (*p* < 0.05). The level of competence on the post-test (M = 3, SD = 0.632) was significantly higher than on the pretest (M = 1, SD = 0.516) (*p* < 0.05). We did not find a correlation between these two concepts at the pretest (τb = − 0.064, *p* = 0.829). We found a strong correlation between the level of competence and students’ feelings of preparedness at the post-test (τb = 0.751, *p* < 0.05). Design factors that influence students’ feelings of preparedness include modeling examples, exposition to authentic learning tasks of adequate complexity, feedback, and teaching a structured strategy.

**Conclusions:** A course designed with 4C/ID model improved students’ self-confidence related to acute care management with a strong alignment with the level of competence. These results could inform educators of the design course to teach critical care skills.

**Reference**

1. Vandewaetere M et al. Med Teach 2015;37:4–20

## P230 A health equity monitoring framework based on process mining

### JN Adams^1^, J Ziegler^2^, M McDermott^3^, MJ Douglas^4^, R Eber^5^, JW Gichoya^6^, D Goode^3^, S Sankaranarayanan^7^, WMP van der Aalst^1^, LA Celi^8^

#### ^1^RWTH Aachen University, Chair of Process and Data Science, Aachen, Germany, ^2^University of Manitoba, Department of Internal Medicine, Winnipeg, Canada, ^3^Harvard Medical School, Department of Biomedical Informatics, Boston, USA, ^4^University of Arizona, Department of Surgery, Tucson, USA, ^5^Montpellier University, Montpellier Research in Management, Montpellier, France, ^6^Emory University, Department of Radiology & Imaging Sciences, Atlanta, USA, ^7^Massachusetts Institute of Technology, Computer Science and Artificial Intelligence Laboratory, Cambridge, USA, ^8^Massachusetts Institute of Technology, Laboratory for Computational Physiology, Cambridge, MA, USA

*Critical Care* 2024, **28(Suppl 1):** P230

**Introduction:** This study introduces a process mining framework to comprehensively evaluate health equity in healthcare delivery. This framework aims to provide a more nuanced understanding of disparities in healthcare processes and help authorities evaluate equity in care delivery by addressing the gap in tools for assessing health equity across various health conditions and treatments.

**Methods:** The study utilized process mining techniques to analyze event logs, capturing all actions during patient care. This approach allowed for the examination of disparities in both single and multiple treatment steps as well as in the overall strategy of treatment delivery. The framework was applied to sepsis patients in the (ICU), with a specific focus on differences in treatment based on sex and English language proficiency. The techniques are generalized to a process mining framework for monitoring health equity.

**Results:** The application of this framework revealed no significant disparities in the treatment of male and female patients with sepsis. However, a notable delay was observed in the initiation of treatment for patients with limited English proficiency, despite similar severity in their condition and subsequent treatment steps as their English-speaking counterparts (Figure).

**Conclusions:** The process mining framework developed in this study offers a comprehensive method for assessing health equity, subsuming existing techniques that typically focus on isolated aspects of patient care. It provides valuable insights into healthcare disparities, thereby serving as a crucial tool for research and policy-making to promote more equitable healthcare delivery.

**Figure (abstract P230):**
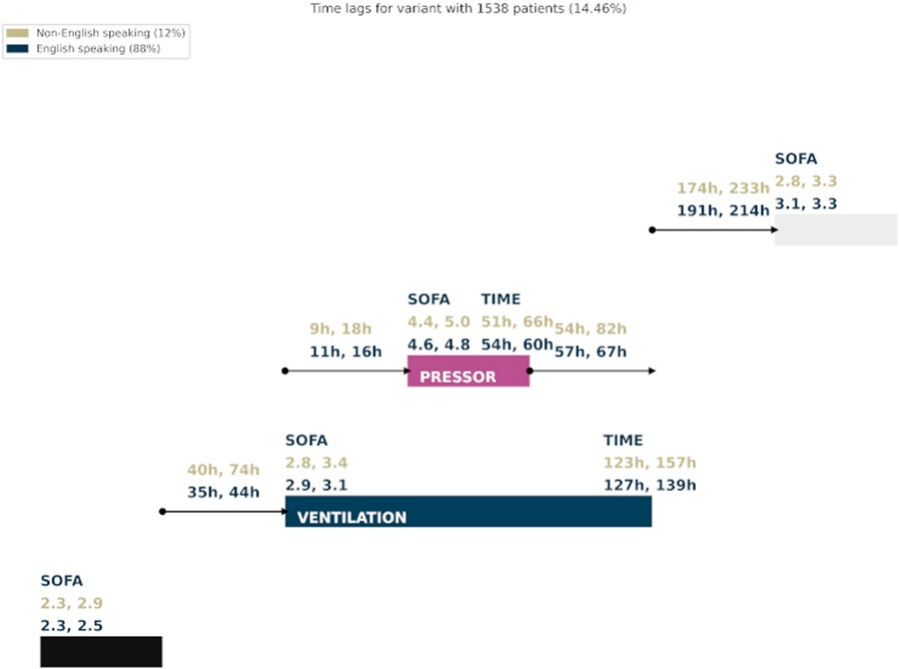
Differences in treatment times for English speakers and non-English speakers. The results are shown for a specific ordering of treatment steps. 90%-confidence intervals are shown for each metric.

## P231 Increase in prevalence of burnout among intensive care professionals during the COVID-19 pandemic: a national comparison study between the prepandemic and the pandemic period

### V Pakou^1^, I Andrianopoulos^2^, G Papathanakos^2^, A Ntantana^3^, M Gouva^4^, V Koulouras^2^

#### ^1^University of Ioannina, Medical School, Ioannina, Greece, ^2^University Hospital of Ioannina, Intensive Care Unit, Ioannina, Greece, ^3^Papageorgiou General Hospital of Thessaloniki, Intensive Care Unit, Papageorgiou General Hospital of Thessaloniki, Thessaloniki, Greece, ^4^University of Ioannina, Department of Nursing, Ioannina, Greece

*Critical Care* 2024, **28(Suppl 1):** P231

**Introduction:** Early studies report a high prevalence of burnout during the COVID-19 pandemic. Nevertheless, few studies compare burnout among ICU healthcare workers (HCW) before and during this period. We went on to investigate the extent of burnout among ICU HCW in Greece during the COVID-19 pandemic, its relationship to spiritual attitudes and mental health and compare it with the situation before the COVID-19 pandemic.

**Methods:** A survey questionnaire, addressed to all ICU HCW, was sent out to all Greek ICUs from June 2015 to December 2015 (pre-pandemic period) and from December 2021 to June 2022 (pandemic period). The questionnaire was a compilation of three questionnaires assessing responders’ level of burnout (Maslach Burnout Inventory), spirituality (Spiritual and Religious Attitudes Questionnaire (SpREUK) sf-10) and level of psychopathology [Symptom Checklist 90-R (SCL-90)]. A comparison of the two cohorts’ results ensued.

**Results:** Overall, 1013 HCW participated (440 from the pre-pandemic and 573 pandemic period), mean age was 40.82 ± 7.19 and 41.60 ± 8.33 (*p* = 0.122) for the two groups respectively. Overall, respondents from the pandemic period compared to the pre-pandemic period had worse scores across all three burnout categories: emotional exhaustion (25.80 ± 10.93 vs. 22.27 ± 10.96, *p* < 0.001), depersonalization (10.40 ± 6.51 vs. 8.68 ± 6.25, *p* < 0.001) and personal accomplishment (31.36 ± 8.63 vs. 35.06 ± 7.50, *p* < 0.001). In addition, respondents from the pandemic era scored lower in the spirituality scale compared to the pre-pandemic period (total score 36.20 ± 12.54, vs. 39.11 ± 12.34, *p* < 0.001). Finally, respondents from the pandemic period had a worse Global Severity Index (0.56 ± 0.44 vs. 0.913 ± 0.73, *p* < 0.001) consistent with an increase in psychopathology during the pandemic period among ICU HCW.

**Conclusions:** During the COVID-19 pandemic era ICU HCW experienced significantly more psychological distress symptoms, had a higher degree of burnout and scored lower in the spirituality scale.

## P232 The portfolio effect: a new opportunity for improving handoffs quality in ICUs

### C Monard^1^, J Carrere^1^, P Abraham^1^, V Cerro^1^, S Polazzi^2^, C Payet^2^, T Rimmelé^1^, A Duclos^2^

#### ^1^Hospices Civils de Lyon, Hopital Edouard Herriot, Service d´anesthésie-réanimation, Lyon, France, ^2^Université Claude Bernard Lyon 1, Research on Healthcare Performance RESHAPE, INSERM U1290, Lyon, France

*Critical Care* 2024, **28(Suppl 1):** P232

**Introduction:** Handoffs are a major determinant of safety but their implementation is heterogeneous and non-standardized. Organizational factors, including the order in which individual cases are handled within the handoff, may play a role in their quality. We aimed to confirm the existence of the portfolio effect (e.g., a decrease in duration of individual cases handoffs as the global handoff progresses) in ICU’s handoffs.

**Methods:** We conducted an observational study in two ICUs (ICU-1, a 20-bed trauma and surgical ICU and ICU-2 a 10-bed medico surgical ICU) within a university hospital, over a 6-month period. The duration of each case (i.e., the handoff of a single patient) during morning handoffs was measured. Patients’ socio-demographic and clinical data were extracted from electronic medical records if they did not refuse to participate. The effect of the case position on its duration was determined using a linear regression model. The case position within the handoff was categorized as either before or after the median position. Covariates associated with case duration were included in the model (age, sex, Charlson comorbidities index, SAPS II score, number of organs supports the night before, center (ICU-1 or ICU-2), admission reason).

**Results:** 2485 individual cases nested in 169 morning handoffs and related to 494 patients stays were included. The mean (± SD) duration of the morning handoff was 60 (± 12.5) minutes in ICU-1 and 35.2 (± 10.6) minutes in ICU-2. The mean (± SD) duration of a case was 175 (± 108) seconds. Trauma stays, patients’ severity and comorbidities, and the number of organs supports were associated with longer case presentations (Figure). Adjusting for these covariates, cases in the second half were shorter compared to cases in the first half [RR 0.65, 95%CI (0.51–0.80)].

**Conclusions:** Interventions aiming to improve handoffs should focus on content *and* setting. The ordering of cases may be of importance and we suggest avoiding to present systematically the same patient at the end.

**Figure (abstract P232):**
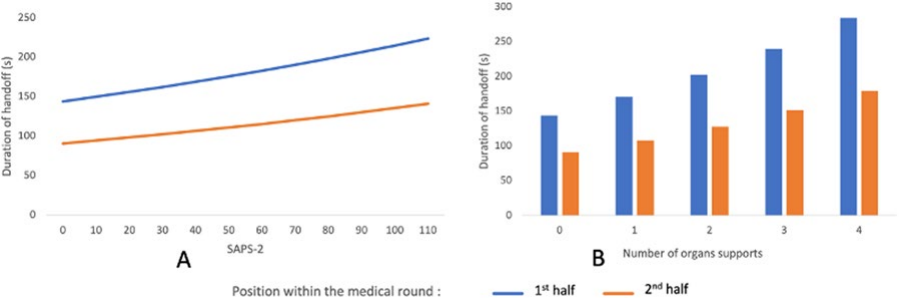
Panel **A** Increase in individual handoff duration according to SAPS-II score (Simplified Acute Physiology Score) in the first and second half of the medical handoff. Panel **B** Increase in individual handoff duration according to the number of organ supports provided the previous night in the first and second halves of the medical handoff (organ supports include renal replacement therapy, mechanical ventilation, vasopressors and others).

## P233 Machine learning improves ICU admission based on lung ultrasound score

### D Oliveira-Saraiva^1^, J Leote^2^, N Garcia^3^, F Gonzalez^2^, HA Ferreira^4^

#### ^1^Institute of Biophysics and Biomedical Engineering, Physics, Lisboa, Portugal, ^2^Critical Care Department, Hospital Garcia de Orta E.P.E, Almada, Portugal, ^3^LASIGE, Lisboa, Portugal, ^4^Institute of Biophysics and Biomedical Engineering, Lisboa, Portugal

*Critical Care* 2024, **28(Suppl 1):** P233

**Introduction:** Can we improve how we look at lung ultrasound score (LUS)? LUS has been considered an effective metric for identifying COVID-19 patients who are likely to require ICU admission. However, different methods are proposed to compute LUS, and no standard currently exists. Therefore, in this study, machine learning (ML) models based on lung ultrasound (LU) were explored to improve decision on ICU admission.

**Methods:** LU data from fifty-one COVID-19 patients were collected, along with ICU admission status [1]. LUSs were computed using three different methods [1,2,3], whereas individual LU findings (LUFs): B-lines; irregular pleura; subpleural, and lobar consolidations, were also considered. Then, support vector machine (SVM) models were built using the LUSs and LUFs as input features, and tenfold cross-validation and Bayesian optimization were applied for robustness.

**Results:** The Table shows the performances of SVM models based on LU in the prediction of ICU admission. Among the previously proposed LUSs, LUS-C showed the highest Acc (82.4%), PPV (86.7%), and NPV (76.2%) values in the prediction of ICU admission. On the contrary, the LUS-A showed the highest AUC (79.0%). Moreover, when using individual LUFs as input for the SVM models, better predictions of ICU admission were obtained. In particular, when using LUF-1 (B-lines alone), higher performance values were obtained for all metrics in comparison with LUS-A and LUS-B. Finally, when using all individual LUFs together (LUF-4), instead of the original LUS-A, as input for the model, increases in Acc, NPV, and AUC values of 5.9%, 28.6%, and 7.7%, were respectively observed.

**Conclusions:** The approach of using total values of LUSs seem to result in the loss of valuable information. In fact, by leveraging ML with individual LUFs, the prediction of ICU admission seems to be improved; nonetheless, warranting validation from clinical practice.

**References**

1. Leote J et al. Ultrasound J. 2022:14:28
2. Ji L et al. Crit Care. 2020;24:700
3. Dargent A et al. PloS One. 2020;15:e0236312

**Table (abstract P233):** Accuracy (Acc), positive predictive value (PPV), negative predictive value (NPV), and area under the curve (AUC) values of the machine learning models built using different inputs

Inputs for the model	Acc (%)	PPV (%)	NPV (%)	AUC (%)
LUS-A	78.4	83.3	71.4	79.0
LUS-B	76.5	78.8	72.2	76.9
LUS-C	82.4	86.7	76.2	73.8
LUF-1 (B-lines)	80.4	86.2	72.7	81.0
LUF-2 (B-lines + Pleura)	82.4	80.6	86.7	89.0
LUF-3 (B-lines + Pleura + SubPCons)	84.3	82.9	87.5	87.9
LUF-4 (B-lines + Pleura + SubPCons + LobCons)	84.3	79.5	100.0	86.7

Lung ultrasound scores (LUSs): LUS-A from [1]; LUS-B from [2]; and LUS-C from [3]; and individual lung ultrasound findings (LUFs): LUF-1 (B-lines); LUF-2 (B-lines+Pleura); LUF-3 (B-lines+pleura+subpleural consolidations (SubPCons)); and LUF-4 (B-lines+Pleura+SubPCons+lobar consolidations (LobCons)), were considered

## P234 Obstetric admissions to intensive care unit: an 11-year retrospective study

### C Rodrigues^1^, I Pinto^2^, D Cabral^2^, D Gomes^2^, G Nobre de Jesus^2^

#### ^1^Faculdade de Medicina da Universidade de Lisboa, Clinica Universitária de Medicina Intensiva, Lisboa, Portugal, ^2^Centro Hospitalar e Universitário Lisboa Norte, Serviço de Medicina Intensiva, Lisboa, Portugal

*Critical Care* 2024, **28(Suppl 1):** P234

**Introduction:** Maternal mortality has declined significantly over the past few decades, particularly in developed countries. Pregnant and postpartum women rarely require intensive care unit (ICU) admission. There are few studies regarding the morbimortality in obstetric critical illness.

**Methods:** This is an observational retrospective cohort study of all pregnant and postpartum women admitted to an ICU of a tertiary university hospital between January 2012 and January 2023. The aim of this study was to assess the most common obstetric-related conditions that require ICU admission. Data was collected from electronic files and SPSS® software was used for statistical analysis.

**Results:** 102 ICU admissions relating to 100 patients were included. 13 (12.7%) were admitted during pregnancy and 89 (87.3%) were postpartum women. Mean age was 33 ± 6.6 years. Mean length-of-stay was 2.5 ± 3.1 days. 38 women (37.3%) had two or more causes of ICU admission and the most common were hypertensive disorders of pregnancy (HDP), postpartum hemorrhage (45.1%) and infection (18.6%). The most common complications were acute kidney injury (26.5%) and respiratory failure (23.5%). About 55% of the admissions required organ support such as blood products (40.2%), invasive mechanical ventilation (30.4%), inotropes and/or vasopressors (15.7%), dialysis (4.9%) or non-invasive ventilation (4.9%). Coagulopathy (B = 11.83, t = 5.22, *p* = 0.006), need for Invasive Mechanical Ventilation (IMV) (B = 10.09, t = 3.92, *p* = 0.017) and gamma-glutamyl transferase (GGT) values at admission (B = 0.035, t = 3.23, *p* = 0.032) were independent predictors of length of stay in HDP patients. We observed no maternal deaths and 20 fetal or neonatal deaths (20.0%).

**Conclusions:** HDP and postpartum hemorrhage were the leading causes of ICU admission. We observed a high rate of organ dysfunction, mainly invasive mechanical ventilation requirement, which is consistent with other studies.

## P234A Peripartum hypertensive disorders: a sub-cohort outcome analysis

### I Pinto^1^, C Rodrigues^2^, D Cabral^1^

#### ^1^Centro Hospitalar e Universitário Lisboa Norte, Serviço de Medicina Intensiva, Lisboa, Portugal, ^2^Faculdade de Medicina da Universidade de Lisboa, Clinica Universitária de Medicina Intensiva, Lisboa, Portugal

*Critical Care* 2024, **28(Suppl 1):** P234A

**Introduction:** The hypertensive disorders of pregnancy (HDP), including preeclampsia/eclampsia and hemolysis elevated liver enzymes and low platelets (HELLP) syndrome are the most frequent nonhemorrhagic diagnoses requiring intensive care unit (ICU) admission in peripartum women. Data regarding factors predicting poor outcome in these patients is scarce.

**Methods:** We analyzed a cohort of pregnant and postpartum women admitted to a tertiary ICU between January 2012 and January 2023. A sub-cohort of patients diagnosed with HDP was retrospectively selected. Main objective was to identify ICU length-of-stay (LoS) predictors. We used t-student test or Pearson correlation coefficient to identify significant associations between independent variables and LoS. A multiple linear regression model with selected variables was used to predict LoS.

**Results:** A total of 49 patients were included, mean age was 32.51 ± 7.0 years. Mean LoS was 2.5 ± 2.5 days. Mean SOFA and SAPS II at admission were 3.0 ± 2.4 and 17.0 ± 9.4, respectively. We observed 36 (73.5%) cesarean deliveries and 37 (75.5%) preterm births. 16 (32.7%) patients had two or more HDP diagnosis. Preeclampsia was observed in 35 patients (71.4%), HELLP syndrome in 24 (49.0%) and eclampsia in 6 (12.2%). In a multiple linear regression, coagulopathy (B = 11.83, t = 5.22, *p* = 0.006), need for invasive mechanical ventilation (IMV) (B = 10.09, t = 3.92, *p* = 0.017) and gamma-glutamyl transferase (GGT) values at admission (B = 0.035, t = 3.23, *p* = 0.032) were independent predictors of LoS.

**Conclusions:** To the best of our knowledge, this is the first study to evaluate predictors of outcome in HDP patients. Our analysis suggests that specific complications, namely acute respiratory failure requiring IMV and coagulopathy may have an impact on these patients´ outcome. Although these results need further confirmation with prospective studies, our data can help to predict morbidity in critically ill peripartum women with HDP.

## P235 Experience “near miss” to postpartum hemorrhage

### LF Godinez Monroy, JA Villalobos Silva, C Zarazua Sosa, JA Garza Carrion

#### Hospital General de Ciudad Victoria, Critical Care, Ciudad Victoria, Mexico

*Critical Care* 2024, **28(Suppl 1):** P235

**Introduction:** Despite all preventive efforts, postpartum hemorrhage is the main cause of maternal mortality in developing countries in Latin America. Its most frequent causes are uterine atony, coagulation deficiencies, and obstetric trauma. 80% of cases are preventable [1].

**Methods:** We searched data for 48 months to date (2021–2023). Historical retrospective, descriptive and comparative study, carried out in a second level hospital in northeastern Mexico. We evaluated categorical and numerical variables to analyze general characteristics of the population with major obstetric bleeding admitted to the intensive care unit. Groups of survivors and non-survivors were held.

**Results:** 78 records of immediate postpartum patients > 18 years old who were admitted to the ICU were included. Age 27.2 ± 7.3 (15–49) years, ICU hospital stay 3.36 ± 2.4 (2–27) days, admission lactate 2.90 ± 1.30 (1.0–6.4) mmol/L, bleeding 2250 ± 1001 (900–4500) mL, atony 21%, rebleeding 24 h 18%, crystalloid resuscitation 24 h 3590 ± 1966 (750–9750) mL, APACHE 8.1 ± 4.8 (4–23), SAPS II. 18.2 ± 7.8 (12–45), SOFA 5.6 ± 2.8 (4–14). Spearman r = 0.940 *p* < 0.001 (bleeding/rebleeding). Spearman r = 0.78 *p* < 0.003 (bleeding/hypoalbuminemia). SOFA score prediction of mortality AUC = 0.94 (95% CI 0.67–0.97) (Figure). Mortality 9%.

**Conclusions:** Major obstetric bleeding was directly correlated with severe hypoalbuminemia and rebleeding in the following 24 h. SOFA score showed high precision and was the best predictor of mortality in our study.

**Reference**

1. Kroh S et al. Anesthesiology Clin. 2021;39:597–611.

**Figure (abstract P235):**
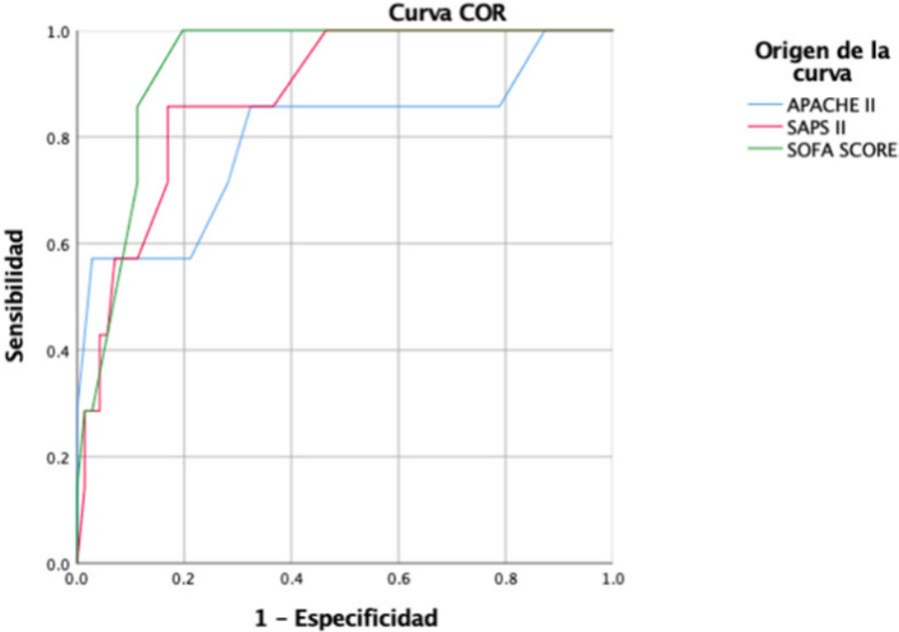
Sensitivity and specificity.

## P236 Obstetric hemorrhage as a prognostic factor in the intensive care unit

### LF Godínez Monroy, C Zarazúa Sosa, JA Garza Carrión, JA Villalobos Silva, A Zárate Gracia

#### Hospital General Dr. Norberto Treviño Zapata, Medicina Crítica, Ciudad Victoria, Tamaulipas, Mexico

*Critical Care* 2024, **28(Suppl 1):** P236

**Introduction:** Obstetric hemorrhage remains the leading cause of maternal mortality worldwide and a common cause of admission to the intensive care unit (ICU). Defined as a cumulative blood loss greater than 1000 mL or blood loss with signs or symptoms of hypovolemia within 24 h after the delivery process; severe hemorrhage is defined as blood loss greater than 2000 mL [1].

**Methods:** We conducted a retrospective cohort study of critically ill obstetric patients admitted to ICU for obstetric hemorrhage from March 2022 to June 31, 2023. 72 medical records were reviewed after obstetric hemorrhage and admission to the ICU. Demographic data, APACHE II score, SOFA, complications were included. The data were used for descriptive analysis of the variables, percentages, means, standard deviation consequently p value (< 0.05), categorical variables with X^2^, Odds Ratio with 95% CI multivariate predictors of risk factors in the ICU.

**Results:** 72 medical records were analyzed, average age 27 ± 7.4 (15–49), admitted to the ICU after cesarean section, hemorrhage > 1000 mL: hypotension 62%, shock 37%, hypovolemia-grade III 19.4%, hypovolemia-grade IV 15.3%, rebleeding 18%. SOFA 6 ± 2 (4–10), APACHE II 12 ± 3 (8–18). ICU stay 5 ± 2 (1–25) days. Bleeding volume 3261 ± 1050 (1000–4500) mL, lactate intake 2.9 ± 1.3 (0.9–6.4) mmol, albumin intake 2.9 ± 0.7 (1–5.5) gr/dL. Water balance upon admission 1490 ± 635 (300–5410) mL, 50% (n-36) had a prolonged stay > 72 h in the ICU; shock was associated with rebleeding (odds ratio 12.9) *p* < 0.05. Association of hypoalbuminemia with rebleeding (odds ratio 1.9) p NS, grade III-IV hypovolemic shock with rebleeding (odds ratio 24.4) *p* < 0.05, acute renal failure with rebleeding (odd ratio 2.5) p NS. Mortality 5.5%.

**Conclusions:** Most had massive hemorrhage, associated with elevated lactate levels and hypoalbuminemia, resulting in prolonged hospital stay and increased risk of morbidity and mortality.

**Reference**

1. Rath WH. Acta Obstet Gynecol Scand. 2011;90:421–8

## P237 Associations of COVID infection and prolonged syndromes with nutritional disorders one year after onset: a multicenter prospective study

### K Kawabata, S Suganuma, N Yokoyama, S Kashiwagi, M Idei, M Yokose, S Takaki, N Kanda, K Nakamura

#### Yokohama City University Hospital, Intensive Care, Yokohama, Japan

*Critical Care* 2024, **28(Suppl 1):** P237

**Introduction:** COVID-19 patients have a variety of clinical symptoms and some of the patients suffer from malnutrition. Hovever, risk factors for long-term nutritional disorders are unclear and need to be revealed to prevent nutritional disorders by early nutritional intervention.

**Methods:** This study was a post-hoc analysis of COVID-19 Recovery Study II (CORES II). Adult patients admitted with the diagnosis of COVID-19 and discharged alive were included for this study, The information such as symptoms and changes in life over the first year after onset were collected using a self-administered questionnaire. We examined the associations of baseline characteristics, disease severity, or symptoms that prolonged one month after onset with malnutrition disorders one year after the onset, defined by the Malnutrition Universal Screening Tool (MUST) score ≥ 1, using logistic regression analysis.

**Results:** A total of 1082 patients (mean age was 56.0 years old, 66% were male, and 38% admitted to the intensive care unit) were analyzed. Of 1082, 267 (24.7%) had malnutrition one year after onset. In multivariate analysis using variables that were statistically significant in univariate analysis, the following factors were found to be independently associated with the malnutrition: female (OR [95% confidence interval], 1.45 [1.02–2.07]), body mass index (BMI) < 18.5 kg/m^2^ (49.5 [14.3–171]), 18.5 < BMI < 20 (10.5 [5.89–18.9]), BMI > 30 (2.63 [1.84–3.76]), maintenance dialysis (3.50 [1.30–9.45]), hospital stay (1.01[1.00–1.02]), and deconcentrating at 1 month after onset (1.68 [1.04–2.72]).

**Conclusions:** Female, underweight, obese, long-term stay at hospital, dialysis, deconcentration at 1 month after onset were associated with malnutritional disorders 1 year after the infection. Patients with these factors are at high risk for long-term malnutritional disorders.

**Acknowledgement:** CORES II was supported by MHLW Research on Emerging and Reemerging Infectious Diseases and Immunization (Program Grant Number JPMH21HA2011).

## P238 Difference in mortality among children in pediatric and adult intensive care units

### J Choi, J Cho

#### Samsung Medical Center, Department of Critical Care, Seoul, South Korea

*Critical Care* 2024, **28(Suppl 1):** P238

**Introduction:** Few real world evidences exist to provide evidences supporting the benefits of pediatric intensive care units (PICUs) on national scale. Therefore, this study aims to evaluate the mortality difference among children admitted to PICUs and adult intensive care units (AICUs) on a nationwide database.

**Methods:** This population-based retrospective cohort study analyzed admissions of children in intensive care units from 1-month to 17-year-old requiring respiratory support from 2016 to 2018 using Health Insurance Review and Assessment database. We used univariate and multivariate logistic regression analyses to estimate the risk factors of mortality. Mortality difference between PICUs and AICUs in subgroups were described using forest plot.

**Results:** A total of 22,383 pediatric ICU admissions were identified from 2016 to 2018. Admission in AICU were associated with significantly increased odds of mortality in multivariate logistic regression analysis (OR 1.620, *p* < 0.001). Emergency admissions (OR 1.623, *p* < 0.001) and ICU stay > 1 day (OR 1.369, *p* = 0.001) were associated with higher mortality in regression analysis. Surgical admissions were associated with lower odds of mortality (OR 0.258, *p* < 0.001). The odds reducing effect in mortality were more prominent in surgical, circulatory, and ICU stay 1 day compared to counterparts in forest plot (Figure).

**Conclusions:** Mortality of children admitted to PICUs were lower than AICUs. The reduction in mortality were more prominent among surgical patients, circulatory disease patients, and short ICU stay of 1 day.

**Figure (abstract P238):**
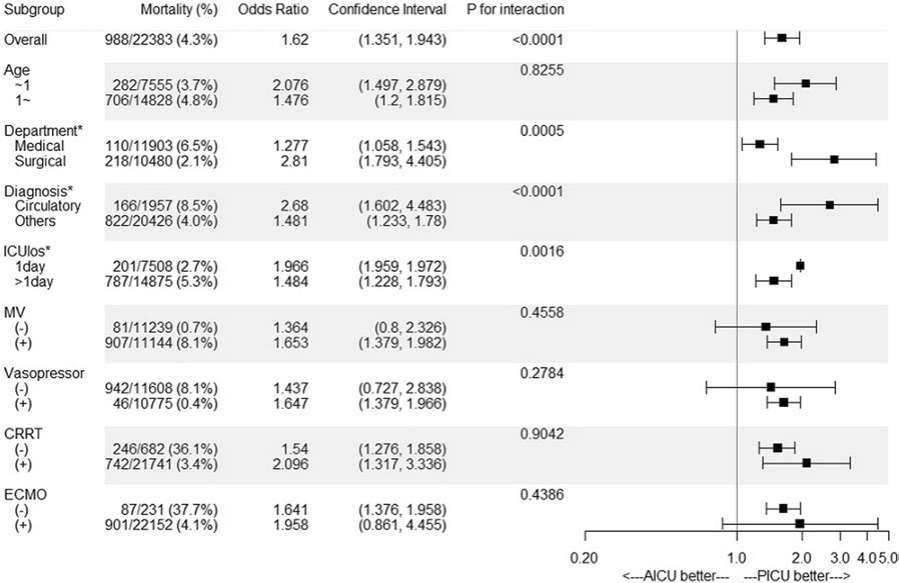
Adjusted for sex, region, admission path (outpatient vs emergency center), hospital type, and admission year.

## P239 Validation of pediatric risk of mortality (PRISM IV) and pediatric index of mortality (PIM 3) in critically ill pediatric oncology patients

### AP Kulkarni^1^, KRK Kalvit^2^, SJB Bhosale^3^

#### ^1^Tata Memorial Hospital, Department of Anaesthesia, Critical Care & Pain, Mumbai, Maharashtra State, India, ^2^Tata Memorial Hospital, Homi Bhabha National Institute, Ex-Asst Professor, Division of Critical Care, Dept. of Anaesthesiology, Critical Care & Pain, Mumbai, Maharashtra State, India, ^3^Tata Memorial Hospital, Homi Bhabha National Institute, Professor, Division of Critical Care, Dept of Anaesthesiology, Critical Care & Pain, Mumbai, Maharashtra State, India

*Critical Care* 2024, **28(Suppl 1):** P239

**Introduction:** No specific scoring systems are validated for pediatric critically ill cancer patients; except those for children with HSCT (O-PRISM) [1]. PRISM IV and PIM 3 have not been validated in this population. We aimed to evaluate the performance of these scores in our ICU.

**Methods:** This is a single-center prospective observational study in pediatric patients admitted for ≥ 24 h. Demographic, physiological and laboratory parameters were collected: lab data (2 h before—till 4 h after) and physiological data (within 4 h of admission). The worst values were considered for the score calculations, which were done using online calculators [3,4].

**Results:** We enrolled 415 pediatric critically ill cancer patients and the ICU. The hospital mortality was 32.7% and 36.1%. For the predicting of hospital mortality, the AUROC for PRISM IV was 0.71, PRISM IV estimated mortality was 0.74 and PIM 3 estimated mortality was 0.78 (Figure). The logistic model for PRISM IV score and the PRISM IV estimated mortality (%) indicated a good model fit. The goodness-of-fit test for the PIM 3 did not fit the data well.

**Conclusions:** PRISM-IV and PIM-3 scoring systems show moderate discriminative ability in this population. However, only the PRISM-IV scoring system showed good calibration in the prediction of survival.

**References**

1. Schneider DT et al. Bone Marrow Transplant. 2000;25:1079–86.
2. Leal PB et al. J Pediatr Hematol Oncol. 2020;42:e563–e568.
3. https://www.cpccrn.org/calculators/prismivcalculator/
4. https://www.espnic.eu/calcolatori/Paediatric%20Index%20of%20Mortality%203%20_%20Professional%20Resources%20_%20Education%20-%20European%20Society%20of%20Paediatric%20Neonatal%20Intensive%20Care%20-%20ESPNIC.html

**Figure (abstract P239):**
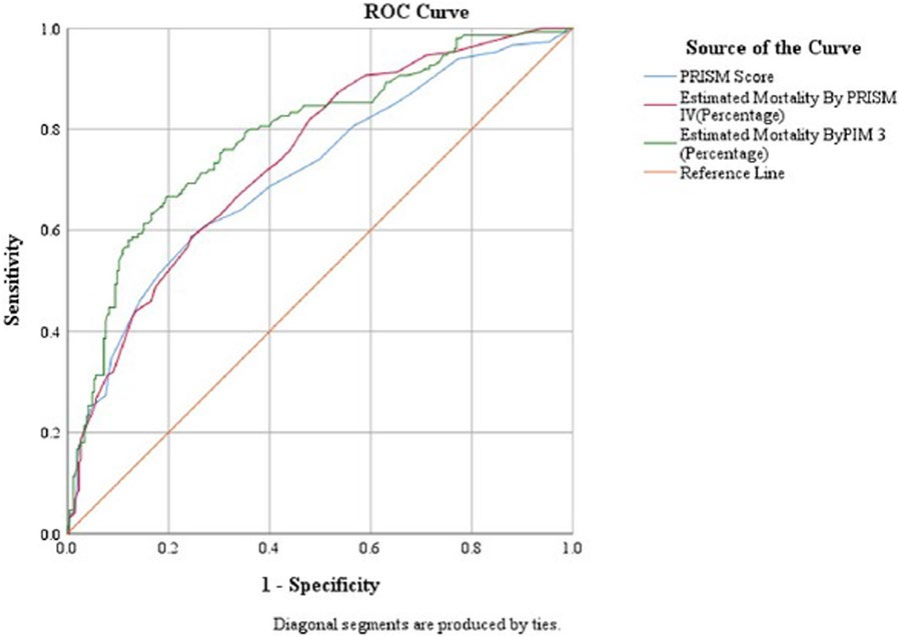
ROC curves for PRISM IV and PIM 3 scoring systems.

## P240 One-year functional recovery from severe COVID-19 is severely affected in the Swedish intensive care and hospital admitted working age cohort

### B Ahlström^1^, R Fritioff^1^, M Hultström^2^, IM Larsson^1^, G Strandberg^1^, M Lipcsey^1^

#### ^1^Uppsala University, Faculty of Medicine, Department of Surgical Sciences, Uppsala, Sweden, ^2^Uppsala University, Faculty of Medicine, Department of Medical Cell Biology, Uppsala, Sweden

*Critical Care* 2024, **28(Suppl 1):** P240

**Introduction:** We hypothesized that the functional recovery, as reflected by sick leave, differ between ICU-admitted and hospitalized COVID-19 patients and the background disability in the population. Long-term symptoms are common in individuals surviving the acute phase of COVID-19, especially those treated in intensive care units (ICUs). Being unable to return to work from sick leave after severe illness is a marker of impeded recovery.

**Methods:** In this cohort-study, we identified all working-age individuals with a COVID-19 discharge diagnosis from an ICU or hospital until 31st July 2020 in national registries. The ICU patients had age, sex, and county-matched population controls randomly assigned. The ICU patients were compared to the other groups on the number of sick leave-free days alive during the first year in multivariable ordinal logistic regression and the proportion of alive individuals on sick leave after one year using multi variable binary logistic regression to adjust for baseline characteristics.

**Results:** We included 1405 COVID-19 ICU patients, 6895 Covid-19 hospital patients, and 5575 population controls. The ICU patients had a substantially higher burden of sick leave than the hospital patients and the population controls (Figure). ICU patients, in comparison to hospital patients, had an odds ratio (OR) of 0.18 (0.16–0.20, 95% confidence interval, CI) for at least one more sick leave-free days alive and ICU patients compared to population controls had an OR of 0.034 (0.029–0.040, 95% CI both *p* < 0.001). Being on sick leave one year after inclusion had similar but inverse ORs.

**Conclusions:** Long-term recovery after ICU admission for COVID-19 is low compared to recovery after non-ICU hospitalization with COVID-19, and the baseline disability in the population.

**Figure (abstract P240):**
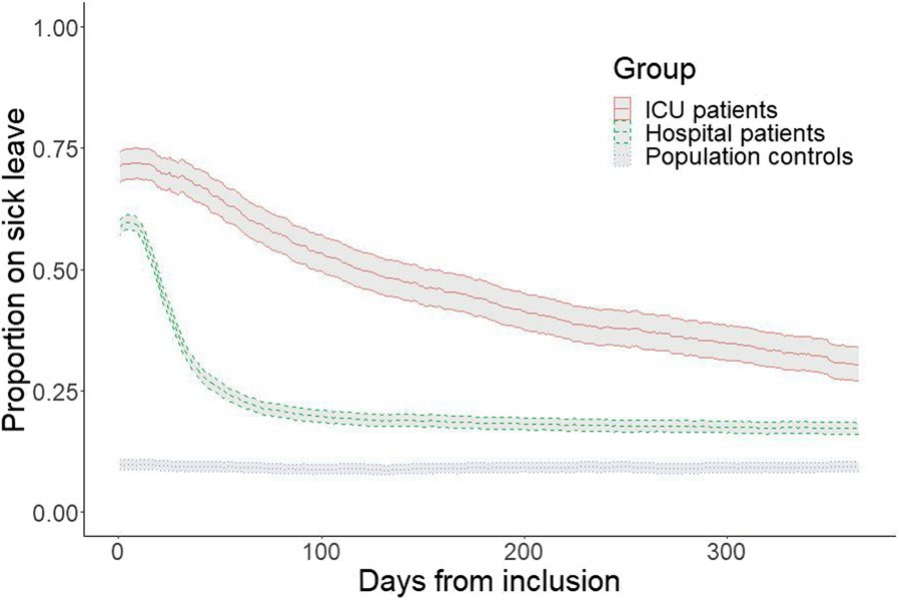
Proportion of individuals on sick leave per day from inclusion to day 365 stratified by group. The grey area represents the 99% pointwise confidence interval. COVID-19: coronavirus disease 2019; ICU: intensive care unit.

## P241 Clavien–Dindo 3–5 complications at 90-days after liver transplantation (LT)

### A Sabate^1^, M Caballero^1^, L Perez^1^, R Gutierrez^2^, R Pujol^3^, J Vidal^3^, A Blasi^3^

#### ^1^Hosital Universitari de Bellvitge, Anesthesiology, Hospitalet del Llobregat, Spain, ^2^Hospital de Cruces, Anesthesiology, Bilbao, Spain, ^3^Hospital Clinic, Anesthesiology, Barcelona, Spain

*Critical Care* 2024, **28(Suppl 1):** P241

**Introduction:** We used a log-binomial regression model to evaluate associations to complications defined by a Clavien-Dindo classification of 3–5 (CDC 3–5) at 90 days after LT.

**Methods:** Organ recovery from controlled cardiac-death donors were managed by normothermic perfusion. Risk was adjusted for age, sex, and MELD score. ClinicalTrials.gov (NCT01539057). CERCA Programme Generalitat de Catalunya.

**Results:** 176 patients were included. Vena cava preservation was achieved in 96% of patients. A portocaval shunt was used in 43.75%. CDC 3–5 status was present in 27.8% (95% CI 21.2–34.5%). 22 patients required reintervention (11 for postoperative bleeding, 3 for biliary complications, 6 for infection, 1 for vascular thrombosis, 1 required a redo LT). Thrombotic complications developed in 8 patients, infective complications occurred in 21 patients (8 had pneumonia, 6 had abdominal infections, and 7 had catheter infections), 2 pneumothoraxes occurred related to central venous access, 4 patients required cardioversion, 15.3% of patient has required mechanical ventilation > 24 h. There were no differences in age, sex, cardiac and respiratory disease, liver diagnose, prior abdominal surgery, diabetes, portal thrombosis, preoperative kidney dysfunction, donor age in the two CDC grade. Surgical outcome are shown in the Table. A baseline hemoglobin 89.0 (84–104) versus 96.0 (84–110) was significant. Hb > 95 g/L conferred protection to CDC 3–5 with a sensitivity of 0.65 (95% CI 0.5–0.78), specificity of 0.54 (95% CI 0.44–0.62), negative predictive value of 0.8 (95% CI 0.7–0.88), positive predictive value of 0.35 (95% CI 0.25–0.46). Intraoperative RBC transfusion of > 2.5 units (aRR 2.02, 95% CI 1.09–3.73) and a surgical time > 390 min was also associated with CDC 3–5.

**Conclusions:** Anemia and major and RBC transfusion of > 2.5 units indicates risk for complications after LT. Given these results, it seems worthwhile to consider the correction of preoperative anemia.

**Table (abstract P241):** Surgical data

Variable	CDC grade, 3–5 (n = 49, 27.8%)	CDC grade, 0–2 (n = 127, 72.2%)
Warm ischemia time (min)	35 (26–52)	40 (27–52)
Cold ischemia time (min)	357 (272–445)	380 (287–444)
Length of surgery (min)*	435 (330–1420)	380 (295–1448)
Reperfusion syndrome	53%	44%
Tranexamic acid administration	44.9%	35.5%
Total RBC (units)* intra + 24 h.post	4 (2–7)	2 (0–4)
Total fluid therapy including albumin (mL)	5511 (4125–8400)	5184 (4153–6766)

* significant

## P242 The pictorial fit-frail scale independently predicts 90-day mortality in critically ill older patients

### L Statlender^1^, O Theou^2^, R Merchshiev^1^, T Shochat^3^, L Cooper^4^, I Kagan^1^

#### ^1^Rabin Medical Center, Beilinson Hospital, Intensive Care Unit, Petah Tikva, Israel, ^2^Dalhousie University and Nova Scotia Health, Division of Geriatric Medicine, Halifax, Canada, ^3^Rabin Medical Center, Beilinson Hospital, Statistical Consulting Unit, Petah Tikva, Israel, ^4^Rabin Medical Center, Beilinson Hospital, Department of Geriatric Medicine, Petah Tikva, Israel

*Critical Care* 2024, **28(Suppl 1):** P242

**Introduction:** Frailty is a state of high vulnerability to adverse health outcomes. It is recognized as an important risk factor in older critically ill patients. The Pictorial Fit-Frail scale (PFFS) is a quick, easy-to-use tool to assess frailty. It is validated in several clinical settings, but not in the ICU. Since 9/2022 we started to routinely ask patients and/or family members of patients aged ≥ 70 who are hospitalized for at least 24 h in the ICU, to complete the PFFS based on the patient's health status prior to hospitalization.

**Methods:** A retrospective study. We collected from the electronic record demographic data, admission prognostic scores (APACHE2, SOFA), PFFS, and mortality status at 90 days from admission. We calculated the correlation between PFFS and prognostic scores. Patients were grouped based on 90-day mortality status. We compared baseline characteristics using t-test or chi-square, as appropriate. Univariate and multivariate analyses were performed to examine the association of clinical factors with mortality.

**Results:** A total of 155 patients aged ≥ 70 were admitted to the ICU for at least 24 h during the study period (9/2022–5/2023). Of those, 125 (81%) filled the PFFS questionnaire. Pearson correlation coefficient between PFFS/APACHE2 and PFFS/SOFA were very low (0.02, and 0.08, respectively). Mortality at 90d was 42% for the entire cohort. No significant differences were found between the alive/dead groups regarding age, gender, BMI, and Diabetes. APACHE2, SOFA score, and PFFS were significantly higher in the mortality group (25.55, 8.75, and 0.35, respectively) compared to those who survived (21.35, 6.20. and 0.24, respectively; *p* < 0.001). In multivariate analysis (Figure), only the SOFA and PFFS were significantly associated with 90-day mortality.

**Conclusions:** The PFFS has a low correlation with ICU well-accepted prognostic scores which shows that they are distinct constructs. The PFFS is associated with 90-day mortality of older critically ill patients, independent of other prognostic scores.

**Figure (abstract P242):**
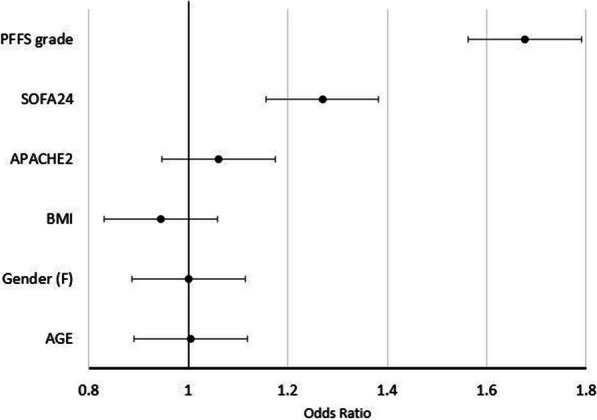
The association of clinical factors with 90-day mortality (odds ratio with 95% CI). *PFFS grade reflects frailty severity. It is categorized according the PFFS score. PFFS > 0.2 was considered frail.

## P243 The added value of high sensitive troponin T and creatine kinase-myocardial band on the predictive performance of EuroSCORE II for in-hospital mortality after elective coronary artery surgery

### MSY Thio^1^, TCD Rettig^2^, LM Vernooij^3^, KF Beukema^3^, PG Noordzij^3^

#### ^1^Amphia Hospital, Anaesthesiology, Intensive Care and Pain Medicine, Breda, Netherlands, ^2^Amphia Hospital, Breda, Netherlands, ^3^St. Antonius Hospital, Nieuwegein, Netherlands

*Critical Care* 2024, **28(Suppl 1):** P243

**Introduction:** Myocardial injury after cardiac surgery is a risk factor for 30-day mortality. Cardiac biomarkers are routinely measured to quantify the severity of postoperative myocardial injury. Cardiac troponin (cTn) and creatine kinase-myocardial band (CK-MB) are established cardiac biomarkers. However, the prognostic value of both biomarkers for mortality after cardiac surgery is unclear.

**Methods:** We conducted a retrospective single-center cohort study between 2018 and 2022 in patients undergoing elective coronary artery bypass graft surgery (CABG). Logistic regression models were used to analyze the European System for Cardiac Operative Risk Evaluation II (EuroSCORE II) with peak concentrations of cTnT and CK-MB. Predictive performance of these models for in-hospital mortality was evaluated with c-statistics and calibration plots. To demonstrate clinical application, the net reclassification index (NRI) was calculated.

**Results:** In 2032 CABG patients the in-hospital mortality rate was 1.2% (N = 24). Postoperative cardiac biomarkers were higher in patients who died compared to survivors (1.04 ug/L [0.54–2.45] vs. 0.51 ug/L [0.34–0.80] *p* < 0.001 for cTnT and 84.00 U/L [37.50–126.25] vs. 33.00 U/L [26.00–43.00] *p* < 0.001 for CK-MB). Prediction models with cardiac biomarkers enhanced calibration in high-risk patients, compared to EuroSCORE II alone, and correctly reclassified 38% (cTnT) to 46% (CK-MB) of patients with in-hospital mortality as high risk. CK-MB showed better calibration and better discrimination for in-hospital mortality compared to cTnT (c-statistic 0.88 vs. 0.86).

**Conclusions:** Postoperative cardiac biomarkers add value to EuroSCORE II for the prediction of in- hospital mortality after elective CABG. Risk stratification of high-risk patients slightly favors a model with CK-MB over cTnT.

## P244 Association between ethnicity and COVID-19 severity in a district general hospital in East London

### P Odedra^1^, S Rizvi^1^, A Boedo^1^, C Megoran^2^, P Antoine^1^

#### ^1^Homerton Healthcare, Adult Critical Care Unit, London, UK, ^2^Homerton Healthcare, Internal Medicine, London, UK

*Critical Care* 2024, **28(Suppl 1):** P244

**Introduction:** During the SARS-CoV-2 pandemic, in the United Kingdom, ethnic minorities have experienced poorer outcomes. This study conducted in Hackney (London, UK), a diverse and deprived urban community, aims at characterising the association between ethnicity and COVID-19 severity.

**Methods: **A retrospective cohort study was conducted at Homerton University Hospital. Patients with PCR-confirmed SARS-CoV-2 infection admitted between 1 March 2020 and 1 June 2020 were included. Indices of Multiple Deprivation (IMD) and self-declared ethnicities were collected. Co-morbidities, organ support requirements and markers of severity were also collected and compared according to ethnic background.

**Results: **Out of 362 included patients, 47.79% were White, 40.60% Black and 11.60% Asian. Despite Asian and Black patients being significantly younger than White patients, mortality rates were comparable across all ethnic groups. IMD scores were comparable across ethnic groups. Compared to White, prevalence of diabetes mellitus (DM) was higher in Asian patients while prevalence of hypertension and DM was higher in Black patients. Compared to White, organ support requirements were significantly increased in Black and Asian patients. Interestingly, only Asian patients required more respiratory support (OR (CPAP or IMV) 3.241, CI95 1.567–6.476) while both Asian and Black patients required more renal replacement therapy compared to White patients (OR 3.761, CI95 1.210–13.76; OR 2.930, CI95 1.067–7.601, respectively). In line with this, markers of severity also indicated higher inflammation in Asian and Black patients compared to White.

**Conclusions:** Morbidity is influenced by ethnic background in COVID-19. Little is known about the association between ethnicity and morbi-mortality in other conditions associated with inflammation. Understanding how ethnic background impacts severity and outcome in other diseases is crucial for targeted public health interventions and equitable resource allocation.

## P245 Predictive cellular markers of outcome in pleural empyema: post-COVID-19 vs no COVID-19 patients

### V Pisarev^1^, AG Chumachenko^2^, DL Fetlam^3^, AA Tarlycheva^2^, AN Kuzovlev^2^, AV Grechko^2^

#### ^1^Federal Research and Clinical Center of Intensive Care Medicine and Rehabilitology, V.A.Negovsky Institute of General Reanimatology, Moscow, Russian Federation, ^2^Federal Research and Clinical Center of Intensive Care Medicine and Rehabilitology, Moscow, Russian Federation, ^3^I.V. Davidovsky City Clinical Hospital, Moscow, Russian Federation

*Critical Care* 2024, **28(Suppl 1):** P245

**Introduction:** Previously we have shown that immune cells ratio predict outcome in pleural emphysema (PE) patients. Since COVID-19 causes lasting harm to the lungs and immune system, we sought to find whether the previous COVID-19 may affect the predictive value of immune cells-based biomarker panel in PE patients.

**Methods:** Study cohort included 216 patients (30% women) with PE, median age 54 (IQR, 41–66), median SOFA score 2 (IQR, 2–2) on admission. Eighty-three patients were considered post-COVID-19 patients as confirmed by previous PCR data. Immune cell count, Neutrophil/lymphocyte ratio (NLR), systemic immune-inflammation index, SII (NLR × platelets), Systemic inflammation response index, SIRI (NLR × monocytes) were used as biomarkers. Logrank test, Chi-square and Fisher test were employed to determine significance at *p* < 0.05.

**Results:** Both post-COVID-19 and No-COVID-19 patients displayed similar hospital lethality (8.3% and 10.5%, respectively, *p* = 0.614). Analysis of neutrophils (N), lymphocytes (L) and monocytes (M) numbers in the whole cohort of patients upon admission revealed significant associations of lethality and increased N (HR = 3.2, 95% CI 1.3–7.6; *p* = 0.026), decreased L (HR = 4.1 95% CI 1.6–10.7; *p* = 0.0005) and increased M (HR = 3.5 95% CI 1.1–11.6; *p* = 0.002) counts (Figure panels A, B, C). After dividing the whole cohort into two groups (post-COVID-19 and no-COVID-19), significance of association of cell biomarker values and outcome were remained only for lymphocyte concentration (HR = 5.9 and HR = 2.9, respectively, *p* < 0.02 in both groups). In a whole cohort, significantly increased values of ratio-based biomarkers SII, SIRI and NLR predicted lethality (Figure panels A, B, C). After dividing the cohort in two groups, most increased (> 9) and statistically significant values for lethality prediction were revealed only in post-COVID-19 patients (Figure panels D, E, F), but not in a No Covid-19 group (Figure panels G, H, I).

**Conclusions:** In PE patients, increased SII, SIRI and NLR values significantly predict unfavorable outcome only in post-COVID-19 group.

**Figure (abstract P245):**
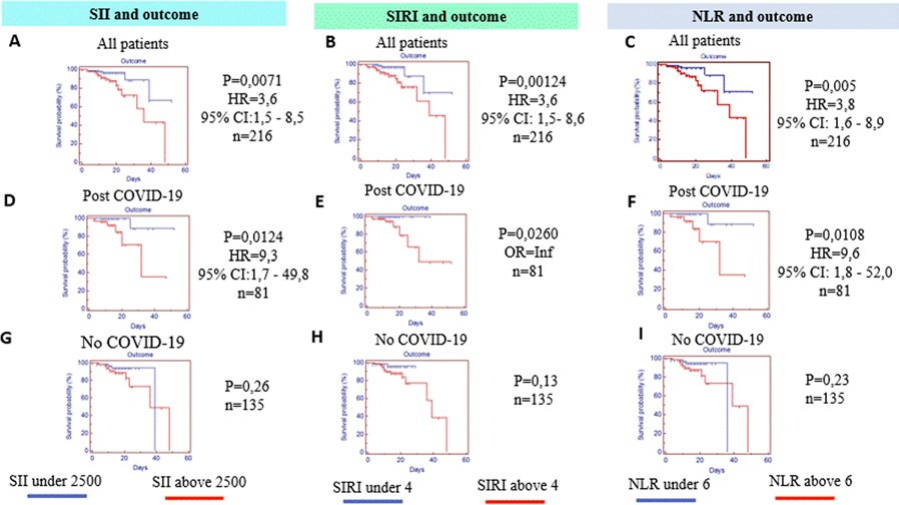
Prediction of pleural empyema outcome using SII, SIRI and NLR indices in post-COVID-19 and No-COVID-19 patients.

## P246 Risk factors for deterioration of the quality of life after discharge from the ICU in patients with COVID-19

### L Simões, C Monteiro, M Sequeira, P Martins

#### Coimbra´s University Hospital, Intensive Care Department, Coimbra, Portugal

*Critical Care* 2024, **28(Suppl 1):** P246

**Introduction:** Post-intensive care syndrome (PICS) affects patients' quality of life. The aim of this study is to assess the risk factors for the reduction of subjective perception of general health status associated with ICU stay in COVID-19 patients.

**Methods:** Retrospective observational study of 186 COVID-19 patients discharged from the ICU, evaluated in a follow-up consultation between July 2021 and July 2023, at a tertiary center in Portugal, The patient’s subjective general health status is classified as a percentage. Data analysis included frequencies, Spearman correlation, Mann–Whitney and χ^2^ test.

**Results:** The statistically significant factors that we identified were hospitalization length of stay (*p* = 0.001, 95% CI − 0.35 to − 0.009, ρ − 0.236), days under sedation (*p* = 0.008, 95% CI − 0.34 to − 0.046, ρ − 0.194), days of invasive mechanical ventilation (*p* = 0.023, 95% CI − 0.31 to − 0.020, ρ − 0.17), days under corticosteroid therapy (*p* < 0.001, 95% CI − 0.34 to − 0.11, ρ − 0.254), and days under neuromuscular blockade (*p* = 0.005, 95% CI − 0.34 to − 0.058, ρ − 0.204). Age, sex, SAPS II and SOFA score on admission did not show statistical significance. The negative value of the Spearman correlation coefficient indicates an inverse correlation, meaning that as the hospitalization/sedation/ventilation days increase, the self-rated general health tends to decrease. However, it suggests a weak to moderate correlation between the variables. In this sample, 45.2% of patients reported feeling less than 80% of their pre-hospitalization well being.

**Conclusions:** Critically ill COVID-19 patients can have a compromised quality of life after ICU discharge. These data reinforce the need for close follow-up for these patients.

## P247 Follow-up of COVID-19 patients after intensive care unit discharge

### L Simões, C Monteiro, M Sequeira, P Martins

#### Coimbra´s University Hospital, Intensive Care Department, Coimbra, Portugal

*Critical Care* 2024, **28(Suppl 1):** P247

**Introduction:** COVID-19 led to several intensive care admissions in recent years and with extended lengths of stay, resulting in changes in the quality of life of surviving patients. The aim of this study was to analyze the data of follow-up consultations in COVID-19 patients admitted to the ICU.

**Methods:** Retrospective descriptive analysis of 186 selected patients discharged from the ICU after hospitalization for COVID-19, evaluated in a follow-up consultation between July 2021 and July 2023, at a tertiary center in Portugal, 6 to 9 months after discharge.

**Results:** A total of 186 patients were included, with a median age of 58 ± 12.9 years. 65.6% male and 34.4% female. 31.7% of patients were previously healthy, and 64% had a non-disabling chronic disease. The mean SAPSII was 33 ± 10.6 and the SOFA was 5 ± 2.7. Hospital stay lasted an average of 17.0 ± 29.5 days and 14.2 ± 17.8 days in intensive care. The mean time of invasive mechanical ventilation was 14.2 ± 13.3 days. 12.9% were placed on ECMO. 58.6% of patients received neuromuscular blockade with a mean duration of 6.9 ± 4.5 days. Corticosteroid therapy was widely used with 88.2% receiving corticosteroid therapy for a mean of 6.5 ± 8.9 days. 37.1% of patients were diagnosed with delirium in the ICU. During the follow-up consultation, 34.9% of patients described their stay in the ICU as good, 15.6% described it as anxiety-inducing, 13.4% associated it with fear, 9.7% as distressing and 26.3% had no memory of their ICU stay. 37.1% of patients reported mobility problems, 34.3% reported experiencing daily pain, 39.3% reported anxiety, 45.2% of patients reported feeling less than 80% of their pre-hospitalization well being. The mean EuroQol 5 (EQ-5D) score was 0.78.

**Conclusions:** The follow-up after discharge from the ICU is important to evaluate and screen for post-intensive care syndrome. EuroQol is an useful instrument to evaluate the health-related quality of life that should be used during ICU follow-up.

## P248 Risk factors for delirium in critically ill COVID-19 patients and the impact on quality of life after discharge

### L Simões, C Monteiro, M Sequeira, P Martins

#### Coimbra´s University Hospital, Intensive Care Department, Coimbra, Portugal

*Critical Care* 2024, **28(Suppl 1):** P248

**Introduction:** COVID-19 led to numerous ICU hospitalizations with prolonged lengths of stay. Follow-ups after ICU discharge are important for screening for post-intensive care syndrome (PICS). Delirium is common in the ICU and can impact patients’ quality of life after discharge. The aim of this study is to evaluate the risk factors for delirium and its impact after ICU discharge.

**Methods:** Retrospective observational study of 186 critically ill COVID-19 patients, evaluated in a follow-up consultation between July 2021 and July 2023, at a tertiary center in Portugal. Data analysis included frequencies, logistic regression to assess risk factors associated with delirium development, and Mann–Whitney to evaluate the relationship between the presence of delirium during hospitalization and physical, psychological and cognitive impairments 6–9 months after discharge.

**Results:** In the evaluation of risk factors for delirium, statistically significant factors were the length of stay (*p* < 0.01, OR 1.04, 95% CI 1.01–1.06), days on mechanical ventilation (*p* < 0.01, OR 1.05, 95% CI 1.02–1.07), days on neuromuscular blockade (NMB) (*p* = 0.04, OR 1.05, 95% CI 1.01–1.09), days on corticosteroids (*p* < 0.01, OR 1.14, 95% CI 1.06–1.22), and days under sedation (*p* < 0.01, OR 1.06, 95% CI 1.03–1.09). SAPS II at admission (*p* = 0.2), gender (*p* = 0.3), and age (*p* = 0.8) had no statistical significance. After ICU discharge, delirium during hospitalization was associated with mild to moderate pain (*p* = 0.04), difficulty with self-care (*p* = 0.02), anxiety (*p* = 0.01), and subjective perception of worse health compared to pre-hospitalization (*p* = 0.04).

**Conclusions:** Prolonged length of stay and longer duration of mechanical ventilation, with the need for NMB and more days under sedation are risk factors for the development of delirium in ICU patients, with an impact on patients’ quality of life after discharge. These findings reinforce the need for follow-up of critically ill survivors of COVID-19.

## P249 Prospective ultrasonographic evaluation of femoral and vastus intermedius muscles as predictors of ICU-acquired weakness in critically ill patients

### M Chaves^1^, S Torres^2^, P Lisnedia^3^, J Alvarado^2^, MV Stozitzky^2^, CA Santacruz^2^

#### ^1^Fundación Santa Fe de Bogotá, Bogota, Colombia, ^2^Fundación Santa Fe de Bogotá, ICU, Bogota, Colombia, ^3^Fundación Santa Fe de Bogotá, Instituto de Medicina del Ejercicio y Rehabilitación (IMER), Bogota, Colombia

*Critical Care* 2024, **28(Suppl 1):** P249

**Introduction:** Prediction of intensive care unit-acquired weakness (ICU-AW), a condition with worsening functional outcomes and increasing healthcare costs affecting ICU survivors, using muscular ultrasound (MUS) has been seldomly tested. This research assessed the diagnostic performance of changes in femoral cross-sectional area (Fcsa) and femoral + vastus intermedius thickness (F + VIth) for predicting the onset of ICU-AW.

**Methods:** 43 patients, surgical (n = 25) and medical (n = 18), underwent serial MUS measurements of Fcsa and F + VIth on days 1–5 after ICU admission. Patients with ICU-AW (MRC scale < 48 at ICU discharge) were compared to those without ICU-AW on the outcomes of mechanical ventilation (MV) days, ICU length of stay (ICU-LOS), extubation failure, need for tracheostomy, and ICU mortality. The predictive capacity of day 1 to day 5 measurements, as well as the proportional (Δ%) and absolute (ΔAbs) changes in the Fcsa and F + VIth indices for the development of ICU-AW, were assessed using the receiver operating capacity area under the curve (ROC-AUC curve).

**Results:** Patients who developed ICU-AW (n = 12; 28%) exhibited a significant reduction in Fcsa (*p* < 0.001) from Day 1 to Day 5. Both Day 1 Fcsa (ROC-AUC 0.72, sensibility 83%, specificity 67%) and F + VIth (ROC-AUC 0.82, specificity 90%) demonstrated moderate predictive capabilities for ICU-AW. Day 1–Day 5 progressive changes in Fcsa (Δ%, ROC-AUC 0.75, specificity 93% and ΔAbs, ROC-AUC 0.7, sensibility 55%, specificity 88%) exhibited similar predictive potential (Figure). While ICU-AW patients had longer ICU-LOS, increased ventilator days, and higher extubation failure rates, no significant differences were observed in tracheostomy need or mortality.

**Conclusions:** Early and progressive muscle loss of Fcsa and F + VIth detected by MUS was able to predict ICU-AW within 5 days after ICU admission, potentially enabling early identification of patients at risk for this debilitating condition and informing timely interventions to improve patient outcomes.

**Figure (abstract P249):**
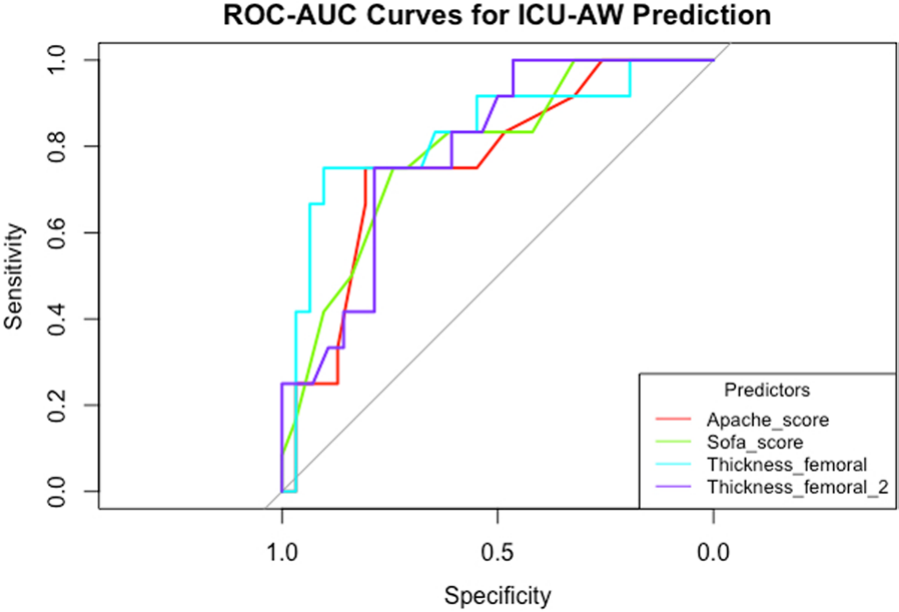
ROC-AUC for ICU-AW prediction.

## P250 Development and internal validation of a novel cardiac-extended SOFA score for improved 30-day mortality prediction in sepsis

### S Lörstad^1^, Y Wang^2^, S Tehrani^1^, S Shekarestan^3^, P Åstrand^4^, P Gille-Johnson^4^, T Jernberg^3^, J Persson^3^

#### ^1^Division of Internal Medicine and Infectious Diseases, Department of Clinical Sciences, Karolinska Institutet, Danderyd University Hospital, Karolinska Institutet, Stockholm, Sweden, ^2^Department of Clinical Sciences, Karolinska Institutet, Danderyd University Hospital, Stockholm, Sweden, ^3^Division of Cardiovascular Medicine, Department of Clinical Sciences, Karolinska Institutet, Danderyd University Hospital, Stockholm, Sweden, ^4^Internal Medicine and Infectious Diseases Clinic, Danderyd University Hospital, Stockholm, Sweden

*Critical Care* 2024, **28(Suppl 1):** P250

**Introduction:** The cardiovascular component of the Sequential Organ Failure Assessment (SOFA) score needs updating. Our aim was to develop a cardiac extended (CE)-SOFA score which reflects myocardial injury and dysfunction and improves 30-day mortality prediction.

**Methods:** A retrospective cohort of critically ill sepsis patients (n = 503) from a previously published report was randomly divided into an exploratory (n = 250) and validation cohort (n = 253). The SOFA score calculated at the point of admission to the ICU was used as the Reference score. High-sensitivity cardiac troponin T (hs-cTnT), N-terminal pro B-type natriuretic peptide (NT-proBNP), heart rate (HR) values and atrial fibrillation (AF; yes/no) were each assigned a point value from 0 to 4 using natural cubic spline. Each variable’s points were added to the SOFA score in different combinations. Improved 30-day mortality discrimination was evaluated using logistic regression and area under receiver operating characteristic curves (AUC).

**Results:** The hs-cTnT (OR 1.9, 95% CI 1.4–2.5), NT-proBNP (OR 1.7, 95% CI 1.3–2.2), and AF (OR 2.0, 95% CI 1.5–2.8), point groups were associated with 30-day mortality after adjustment for the other five SOFA score components whereas the current cardiovascular component (OR 0.85, 95% CI 0.6–1.2) and HR (OR 0.9, 95% CI 0.6–1.3) point group were not. All 23 different CE score combinations enhanced prognostic accuracy (AUC 0.67–0.75, *p* < 0.001) compared to the SOFA score (AUC 0.62, *p* = 0.002). The best performing score (CE-SOFA), which included hs-cTnT, NT-proBNP and AF, showed improved discriminative ability in the validation cohort (Figure).

**Conclusions:** The CE-SOFA score improved 30-day mortality discrimination compared to the SOFA score. There is a need for prospective external validation of the CE-SOFA score ahead of SOFA 2.0.

**Figure (abstract P250):**
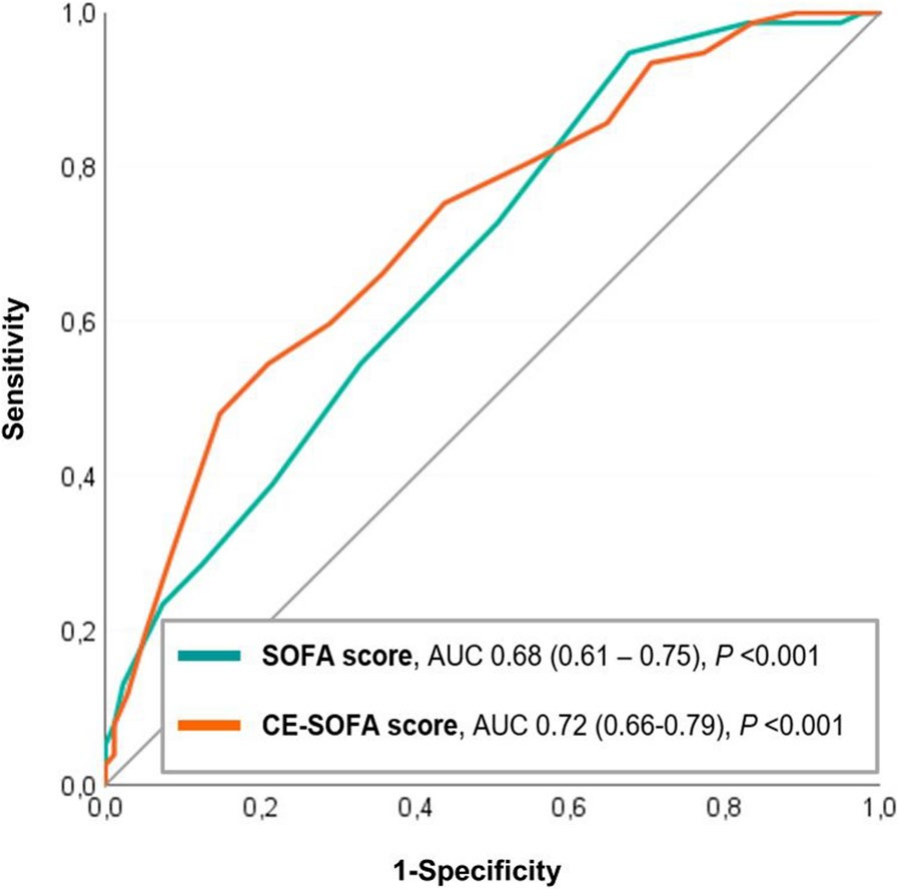
Validation of the best-performing cardiac-extended (CE)-SOFA score in the validation cohort. Receiver operating characteristic curves showing 30-day mortality discrimination of the SOFA score compared to the CE-SOFA score. The CE-SOFA score (0–36) is the sum of the SOFA score (0–24), hs-cTnT points (0–4), NT-proBNP points (0–4) and AF points (0 or 4).

## P251 Post-intensive care COVID survivorship clinic: 12-month follow-up results

### J Collins^1^, M Gilmartin^1^, S Mason^1^, A Horgan^1^, M Ryberg^2^, M Baily-Scanlan^3^, M Donnelly^1^, V O´Doherty^2^, YP Kelly^4^

#### ^1^Tallaght University Hospital, Department of Critical Care, Dublin 24, Ireland, ^2^Tallaght University Hospital, Department of Psychology, Dublin 24, Ireland, ^3^Tallaght University Hospital, Department of Physiotherapy, Dublin 24, Ireland, ^4^Tallaght University Hospital, Intensive Care, Dublin 24, Ireland

*Critical Care* 2024, **28(Suppl 1):** P251

**Introduction:** The aim of this study was to assess the difference in cognitive, psychological and physical consequences of COVID-19 in patients who had been admitted to the ICU and discharged alive, between their 6-month and 12-month follow-up at our post-ICU (PICS) clinic.

**Methods:** We performed a prospective cohort study in our multidisciplinary post-ICU (PICS) follow-up clinic at Tallaght University Hospital, which includes critical care physicians, a psychologist, a physiotherapist and a research nurse. Our study population were patients who had been admitted to the ICU in our tertiary referral centre with COVID-19 pneumonitis 12 months earlier.

**Results:** A total of 20 patients attended the 12-month PICS follow-up clinic following admission to ICU with COVID-19 pneumonitis. Mean grip strength had improved significantly from 25 (SD 1) to 31.3 pounds (8.2; *p* < 0.001) between the 6-month and 12-month PICS clinic follow-up. The 6-min walk test time had also significantly improved from 360.9 m (SD 130.6) to 417.4 m (119.3; *p* = 0.014). The lowest Borg scale score had significantly improved from 0.4 (SD 0.9) to 0.2 (0.5; *p* = 0.046). There was no significant improvement in cognitive and psychological outcomes between the 6-month and 12-month clinic follow-up, with the mean Montreal Cognitive Assessment (MoCA) Score remaining low at 23.4 (SD 9) and the mean Patient Health Questionnaire-9 (PHQ-9) score high at 11.6 (SD 13.1), in keeping with moderate depression.

**Conclusions:** In this single centre prospective cohort study, physical outcomes significantly improved for patients followed up in a multidisciplinary post-intensive clinic between 6-months and 12-months post ICU discharge. There was however no significant improvement in cognitive and psychological outcomes between the 6-month and 12-month follow-up, indicating that patients continue to require multidisciplinary follow-up for an extended period post ICU discharge to ameliorate the burden of comorbidity associated with post-ICU survivorship.

## P252 Association of sepsis with long-term mortality and causes of death in the Swedish intensive care cohort

### B Ahlström, IM Larsson, G Strandberg, M Lipcsey

#### Uppsala University, Faculty of Medicine, Department of Surgical Sciences, Uppsala, Sweden

*Critical Care* 2024, **28(Suppl 1):** P252

**Introduction:** We aimed to determine whether sepsis in the intensive care unit (ICU) is independently associated with long-term mortality (primary outcome) and distribution of causes of death (secondary outcome) in order to inform follow up interventions. Follow up clinics for ICU patients are increasingly being used in order to decrease long-term mortality and morbidity. However, the long-term mortality and causes of death are not thoroughly described.

**Methods:** We report a cohort study on Swedish intensive care registry (SIR) patients admitted to an ICU between 2005 and 2016 with outcomes collected until June 2020. Patients diagnosed with sepsis were allocated to the sepsis group and all other patients to the non-sepsis group. The primary outcome was the hazard ratio (HR) for death in patients with sepsis compared to non-sepsis patients in a multivariable Cox model for the entire follow-up and predefined periods. The secondary outcome was the distribution of marginal risk ratios (mRRs) in multivariable multinomial model for causes of death between sepsis and non-sepsis patients.

**Results:** We included 33,994 patients with sepsis and 280,635 without, with an overall median age of 65 years (interquartile range [IQR], 50–76) and 41.6% women. Median follow-up in survivors was 3071 (IQR, 2137–4136) days. The HR of death for sepsis versus non-sepsis was 1.02 (95% confidence interval 1.00–1.04, *p* = 0.032). Sepsis was associated with survival 0–30 days from admission but mortality during later study periods until 5 years from admission (Figure). Sepsis was associated with an increased mRR for infectious, other tumors, and urogenital causes of death. Non-sepsis was associated with an increased mRR for circulatory injuries, intoxications, and psychiatric causes of death.

**Conclusions:** In our cohort, ICU patients with sepsis have a higher adjusted risk of death than other ICU patients. The risk of death from an infection remained increased also in the last study period 8–15.5 years after admission in sepsis patients.

**Figure (abstract P252):**
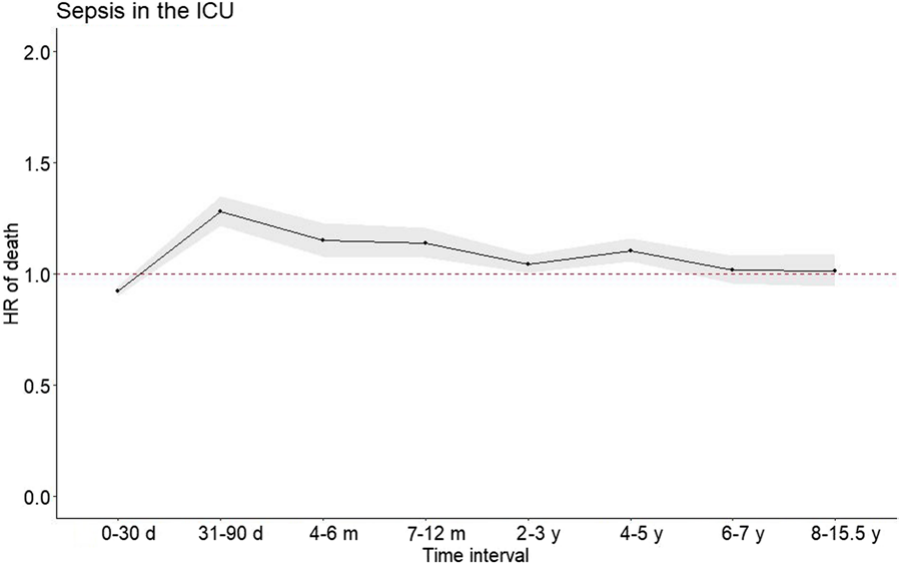
Line plots of hazard ratios for variables in a series of Cox models on mortality after intensive care admission. Each Cox model is fitted for a specific time interval: 0–30 days, 31–90 days, 4–6 months, 7–12 months, 2–3 years, 4–5 years, 6–7 years and 8–15.5 years. The shaded area is the 95% confidence interval, unadjusted for repeated measures.

## P253 The risk of subsequent cardiovascular events among sepsis survivors in Sweden: a population-based matched cohort study

### H Wetterberg^1^, A Nilsson^2^, A Linder^1^, J Sundén-Cullberg^3^, M Inghammar^1^

#### ^1^Lund University, Infection Medicine, Department of Clinical Sciences Lund, Lund, Sweden, ^2^Lund University, Department of Laboratory Medicine/Division of Occupational and Environmental Medicine, Lund, Sweden, ^3^Karolinska Institutet, Department of Medicine Huddinge, Division of Infectious Diseases, Stockholm, Sweden

*Critical Care* 2024, **28(Suppl 1):** P253

**Introduction:** Severe sepsis can trigger acute cardiovascular events, but for how long this increased risk remains in sepsis survivors is unclear. The aim of this study was to assess the long-term risk of cardiovascular hospitalizations and deaths in patients with community-acquired severe sepsis admitted to an ICU compared to the general population.

**Methods:** This retrospective cohort study included 20,313 individuals with incident hospital-treated sepsis or infection identified through Swedish health care registries. Patients admitted between 2008 and 2019 and aged ≥ 18 were included. Each case was matched to 20 controls, and to remove confounding we applied entropy balancing. Cardiovascular event was defined as hospitalisation or death due to cerebral infarction, heart failure or myocardial infarction. To explore the impact of prior heart disease, we conducted a subanalysis on 5,477 patients with no history of heart disease.

**Results:** Among the survivors, 14.5% had a cardiovascular event during the entire follow-up period, compared to 9.6% among the controls. Overall, the risk of cardiovascular events was higher in sepsis patients during the first four months after discharge (see Figure). The increased risk of cerebral infarction was limited to the first two months, myocardial infarction to the first three months, and heart failure to the first four months. Among the 5477 patients with no history of heart disease, the increased risk of incident cardiovascular event persisted for at least six months in comparison to their matched controls.

**Conclusions:** In the first four months following discharge, sepsis survivors had a heightened risk of cardiovascular events compared to matched population controls. Subsequently, this elevated risk diminished. However, as patients with no history of heart disease had increased risk for at least 6 months after discharge, sepsis might be a risk factor for cardiovascular events and preventive measures should be considered.

**Figure (abstract P253):**
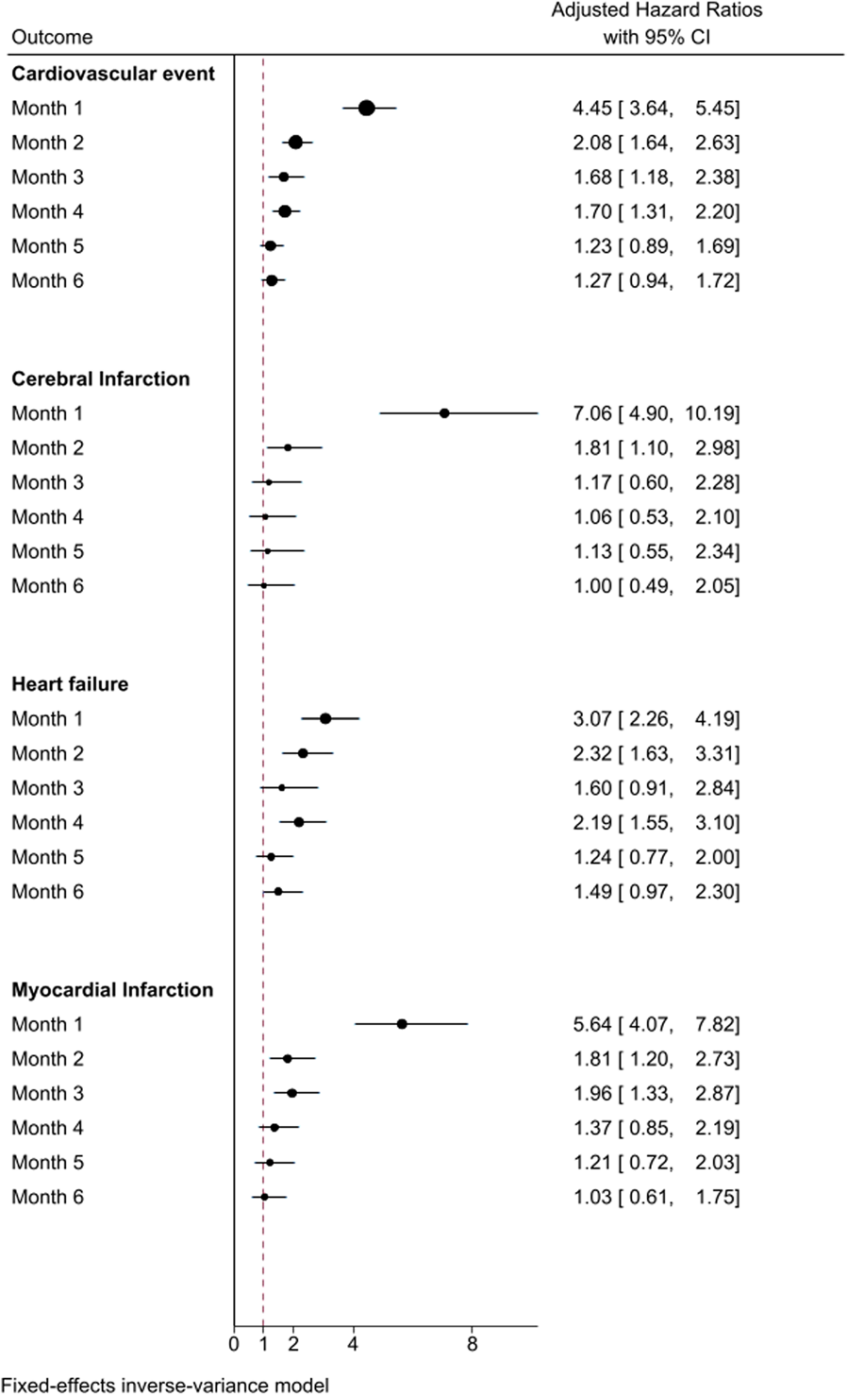
Adjusted hazard ratios of major cardiovascular events after severe sepsis.

## P254 Prevalence of post-intensive care syndrome in survivors after critical care illness in Japan: a prospective nationwide registry study by JPICS database

### J Hatakeyama^1^, S Inoue^2^, D Kawakami^3^, Y Ogata^4^, S Aso^5^

#### ^1^Osaka Medical and Pharmaceutical University, Emergency and Critical Care Medicine, Takatsuki, Japan, ^2^Wakayama Medical University, Emergency and Critical Care Medicine, Wakayama, Japan, ^3^Aso Iizuka Hospital, Intensive Care Medicine, Iizuka, Japan, ^4^Yao Tokushukai General Hospital, Critical Care Medicine, Osaka, Japan, ^5^Graduate School of Medicine, University of Tokyo, Biostatistics and Bioinformatics, Tokyo, Japan

*Critical Care* 2024, **28(Suppl 1):** P254

**Introduction:** The Japanese Society of Intensive Care Medicine (JSICM) has started a database project (Japanese Post-Intensive Care Syndrome: JPICS Database) to investigate the long-term prognosis of patients who have left the ICU from October 2022. In the present study, this database was used to investigate the prevalence of PICS 3 months after ICU discharge.

**Methods:** The JPICS database is a system that registers patients' mobile phone numbers during hospitalization and automatically conducts a questionnaire survey on their living conditions, physical functions, cognitive functions, and mental health using a mobile phone short message system 3 months, 6 months and 1 year after ICU discharge. The prevalence of PICS 3 months after ICU discharge was investigated in patients admitted to the ICU between October 2022 and June 2023. PICS was defined as any one of the following functional impairments: a physical impairment was defined as a score of ≤ 90 points on the Barthel Index, cognitive impairment as a score of < 40 points on the Short-Memory Questionnaire, and mental disorder as a score of ≥ 8 points on the Hospital Anxiety and Depression Scale-anxiety or -depression.

**Results:** 84 patients who responded to the questionnaire were included in the analysis. Age was 70.5 years, male 63.1%, ventilated 60.7%, SOFA score 6 points at ICU admission, PICS prevalence was 66.7%, physical impairment 39.3%, cognitive impairment 46.4%, mental disorder 41.7% and multiple functional impairments were found in about 40% of the patients. In addition, 24% of patients complained of pain that occurred after ICU discharge and analgesic was required in approximately 30% of these patients. The employment rate before admission was 45.2%, and 44.7% were able to return to work after ICU discharge.

**Conclusions:** The prevalence of PICS was approximately 60% and multiple functional impairments were present in approximately 40% of patients 3 months after ICU discharge. The promotion of PICS measures in critically ill patients is a future challenge.

## P255 Subphenotypes at intensive care unit discharge are associated with one-year mortality

### MA Slim^1^, RBE van Amstel^1^, LDJ Bos^1^, OL Cremer^2^, WJ Wiersinga^3^, T van der Poll^3^, LA van Vught^1^

#### ^1^Amsterdam UMC, Department of Intensive Care, Amsterdam, Netherlands, ^2^University Medical Center Utrecht, Department of Intensive Care, Utrecht, Netherlands, ^3^Amsterdam UMC, Department of Medicine, Amsterdam, Netherlands

*Critical Care* 2024, **28(Suppl 1):** P255

**Introduction:** Intensive care unit (ICU)-survivors show persistent cognitive, physical and functional impairment, leading to an increased risk of mortality. On ICU admission subphenotypes [1] have been identified in patients admitted with acute respiratory distress syndrome (ARDS) that are prognostic of outcome and predictive of treatment response. We hypothesized that if these ARDS subphenotypes are assigned at ICU discharge, they are associated with one-year outcome.

**Methods:** A secondary analysis of a prospective observational cohort study conducted in two Dutch ICUs between 2011 and 2014 was executed. Patients discharged alive from the ICU and of whom biomarkers were measured on the day of ICU discharge (− 2 or − 1 days) were included. Subphenotypes were adjudicated by using a previously published parsimonious model making use of plasma levels of bicarbonate, interleukin-8 and protein C to adjudicate patients into two groups, a hyperinflammatory and a hypoinflammatory subphenotype. Subphenotype distribution at ICU discharge, clinical characteristics, plasma protein biomarkers at ICU discharge and outcomes were analyzed.

**Results:** Of the 1483 patients in our cohort, 6% (n = 86) were assigned to the hyperinflammatory and 94% (1397) to the hypoinflammatory subphenotype at ICU discharge. Patients adjudicated to the hyperinflammatory subphenotype were discharged with signs of more severe disease (higher SOFA scores (7 [IQR 5–9] vs. 4 [IQR 2–6], *p* < 0.001). Patients discharged with the hyperinflammatory subphenotype showed significantly more derailed biomarkers at ICU discharge in all domains (Figure). One-year mortality was higher in patients adjudicated to the hyperinflammatory subphenotype (48% vs. 28%, *p* < 0.001).

**Conclusions:** Patients adjudicated to the hyperinflammatory subphenotype at ICU discharge showed significantly stronger anomalies in pathways implicated in the pathogenesis of critical disease and increased mortality at one-year follow up.

**Reference**

1. Calfee CS et al. Lancet Respir Med. 2014;2:611–620

**Figure (abstract P255):**
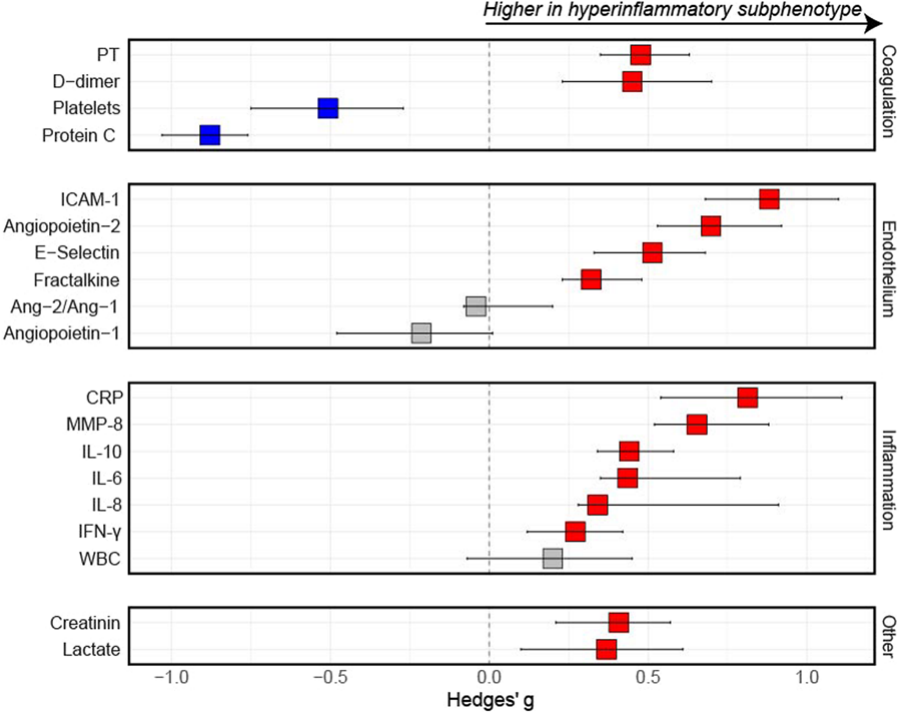
Biomarker differences at ICU discharge according to subphenotypes. The magnitude in biomarker differences is expressed with Hedges’ g with their 95% confidence interval.

## P256 Health-related quality of life of critical patients 1 year after discharge from intensive care, prospective cohort study

### N Fuentes, M Esperatti, M Olmos, E Corso, I Santomil, M Flamini Marczuk, A Miranda Tirado, L Stein, J Suárez Díaz, M González

#### Hospital Privado de Comunidad, Critical Care Unit, Mar del Plata, Argentina

*Critical Care* 2024, **28(Suppl 1):** P256

**Introduction:** Health-related quality of life (HRQoL) is the relevant outcome of intensive care on which everyone involved agrees [1–3]. The objective of this study was to estimate HRQOL in adults who suffered a critical illness at one year. Additionally, possible predictors of HRQoL were evaluated.

**Methods:** A prospective cohort study was conducted all patients requiring life support admitted at critical care unit (ICU) between 2017 and 2019 were included. HRQoL was assessed using EuroQoL-5D-5L (EQ-index valeu) at admission and at follow-up (6 and 12 months). Longitudinal data analysis (LDA) was performed to evaluate the effect of time on HRQoL. Predictors of “better versus worse” HRQoL at year were identified using logistic regression.

**Results:** 195 patients were included (Table), EQ-index was baseline 0.94(0.85–1), at 6 months 0.94(0.83–1) and at 12 months 0.90(0.71–1). In LDA, HRQoL at 12 months was worse (*p* < 0.001), even adjusted for confounders (being very old, severity at admission, requiring mechanical ventilation, female sex, number of comorbidities and frailty) (*p* < 0.001). Being ≥ 80 (*p* = 0.026), female sex (*p* = 0.013), and having three or more comorbidities (*p* = 0.047) were significantly associated with the worst perception of HRQoL at 12 months follow-up. There was a trend in the association between frailty and HRQoL (*p* = 0.064). The severity of the disease (APACHE ≥ 20) (*p* = 0.431) and the requirement for mechanical ventilation (*p* = 0.975) had no association with the perception of HRQoL.

**Conclusions:** Patients significantly worsened their HRQoL at one year of post-intensive care follow-up. Being over 80 years old, being female, and having three or more comorbidities at admission were significantly associated with worse HRQoL at follow-up.

**References**

1. Ridley SA et al. Anaesthesia. 1990;45:808–813.
2. Graf J et al. Crit Care Med 2003;31:2163–2169.
3. Desai SV et al. Crit Care Med. 2011;39:371–379.

**Table (abstract P256):** Basal characteristics of the population

Characteristic	Result
Age years (median, pp 25–75%)	65.9 (52.9–74.5)
Female; n (%)	68 (36)
APACHE Score (median, pp 25–75%)	19 (14–23)
SOFA Score (median, pp 25–75%)	9 (6–10)
Mechanical ventilation(n = 181); n (%)	145 (80.11)
Renal replacement therapy (n = 181); n(%)	17 (9.4)
Vasopressor requirement (n = 181); n (%)	149 (82.3)
Comorbidities number (median; pp 25–75%)	2 (2–4)

## P257 Characteristics and outcomes of patients discharged direct to home from intensive care

### H Amada^1^, J Jerred^2^, J Thomas^2^, PA Turton^2^

#### ^1^Warrington and Halton Hospitals NHS Trust, Anaesthesia, Warrington, UK , ^2^Warrington and Halton Hospitals NHS Trust, Intensive Care Unit, Warrington, UK

*Critical Care* 2024, **28(Suppl 1):** P257

**Introduction:** The incidence of discharging patients directly to home (DDH) is variable across units, but there is evidence that the practice is increasing. There is conflicting data on the safety of DDH, compared to discharging to a ward (DW). The aim of this service evaluation is to identify differences in admission characteristics between DDH and DW patients, and to compare physical function at ITU discharge, and the incidence of re-admission and death after discharge.

**Methods:** Survivors of critical illness who had a period of mechanical ventilation were identified from routine data sources. Patients were placed in two groups based on discharge status (DDH or DW). Their baseline admission characteristics, length of stay and mechanical ventilation, and their physical function at discharge from ITU were compared, as were re-admission rates and mortality within 90 days of discharge.

**Results:** There were 53 patients in the DDH group and 108 in the DW group, and there were no differences in sex between groups. The DDH group were significantly younger (Median age 44 vs. 56.5 years, *p* < 0.001), had significantly lower APACHE II scores (12 vs. 16, *p* < 0.001), and were mechanically ventilated for significantly fewer days (2 vs. 4, *p* < 0.001). The number of days from extubation to a formal decision to discharge was significantly lower in the DDH group (2 vs. 5 days, *p* < 0.001). Fifty percent of DW patients were admitted to ITU having first been an inpatient, compared to 24.52% in the DDH group (*p* = 0.002). Physical function scores at discharge from ITU were significantly higher in the DDH group (median CPAx score 48 vs. 38, *p* < 0.001). There were no differences in re-admission or mortality.

**Conclusions:** Discharge direct to home does not lead to an increased incidence of re-admission or death compared to patients discharged to a ward, and they are discharged with higher physical function scores. Age, illness severity, number of days ventilated, and inpatient status prior to ITU admission are favourable in DDH patients.

## P258 Green ICU: a proposed protocol to greenify intensive care: a single center experience

### D Correia^1^, AR Silva^2^, B Queiroz^2^, S Travassos^2^, G Nobre de Jesus^2^, JM Ribeiro^2^

#### ^1^Centro Hospitalar Universitário Lisboa Norte, Intensive Care Medicine department, Lisboa, Portugal, ^2^Centro Hospitalar Universitário Lisboa Norte, Serviço de Medicina Intensiva, Lisboa, Portugal

*Critical Care* 2024, **28(Suppl 1):** P258

**Introduction:** If healthcare in the global scale were a country, it would be the fifth biggest carbon emitter on the planet. This awareness has globally increased concern. Within the hospital, the intensive care unit (ICU) is one of the considerable waste generators. Although challenging, awareness regarding this issue is important, and measures can be undertaken to reduce this impact.

**Methods:** A literature review identified the problem’s magnitude and potential targets for improvement regarding the environmental waste and pollution associated with the ICU. The project is running at an urban, tertiary care, university hospital. Accordingly, we identified and proposed an integrated pool of measurements designed to have impact on waste production and treatment at the ICU level. In a Delphi-like manner, the measures to implement were selected.

**Results:** Of the proposed measurements, subscription rate > 80% evolved the following: recycling all the confidential paper waste—with a witnessed recycling preserving the confidentiality; recycling all the non-contaminated plastic and cardboard waste; developing regular scientific dedicated internal meetings; abolishing pre-made central venous catheters kits—with unnecessary material—and replace it with individually selectable material; reserve the single-use bronchoscope to emergency situations and installation of light sensors on the halls to prevent lighting when not needed. For each measurement we selected a work group dedicated to its implementation. The first three listed are already implemented and the remaining ones are in assessment, logistics scouting and implementation process. Assessment of fulfillment by professionals revealed a high level of adhesion.

**Conclusions:** There is an unavoidable environmental impact of health care in general. Nevertheless, it is possible to reduce such impact by modulating simple practices. Green-ICU teams and green-ICU bundles produce awareness and can be motors to foster specific practice changes personalized to each ICU.

## P259 Comparative environmental impact assessment of reutilized and disposable bronchoscopes

### B Queiroz, S Travassos, D Correia, G Nobre de Jesus, J Ribeiro

#### Centro Hospitalar Universitário Lisboa Norte, Intensive Care Medicine, Lisbon, Portugal

*Critical Care* 2024, **28(Suppl 1):** P259

**Introduction:** In an eco-conscious era, the idea of a green intensive care unit (ICU) is evolving healthcare. With medical care contributing about 4.4% of CO_2_ emissions through energy use and waste production, choices like reusing bronchoscopes versus single-use ones are being weighed due to their impact on waste and energy.

**Methods:** Literature review assessed hospitals' practices on reusable and disposable bronchoscopes. A prospective single-center observational study was conducted to analyse key environmental indicators, including carbon footprint, water consumption and waste generation, number of items used, their weight and recyclability.

**Results:** Reusable bronchoscopes are greener in manufacturing but use more water and energy in their lifecycle due to reprocessing. Despite comparable clinical effectiveness, single-use bronchoscopes do not minimize costs when considering expenses related to cross-infection. With this evidence, we hypothesized that in our ICU, the reusable bronchoscopes would be more environmentally friendly due to the energy source used in our hospital. In a national ranking analysis, our hospital was placed first as the most energetically efficient hospital—partially due to the use of solar panels as energy sources—and, in second place regarding hydric efficiency. We're changing our urban university hospital protocol to favour reusable bronchoscopes over single-use ones, except for emergencies such as difficult tracheal intubation and for restrictive periods. Over the next year, a before-after study is being conducted, focusing on processing costs, sterilization chemicals, water and energy use, and waste produced.

**Conclusions:** The environmental impact of bronchoscopes is influenced by various factors and the decision-making regarding which one is more favourable should include a personalized analysis of each ICU. Future advancements should focus on developing more sustainable reprocessing practices and enhance the eco-efficiency of disposable options.

## P260 EMR smart phrases to improve and audit quality metrics compliance in the cardiac surgery ICU

### R Said, A Ramirez, D Loftus, J Giordano, A Stuart

#### Hackensack University Medical Center, Cardiac Surgery, Hackensack, USA

*Critical Care* 2024, **28(Suppl 1):** P260

**Introduction:** Confronting challenges within a high-volume, high-acuity ICU characterized by a notable staff turnover poses inherent difficulties in communication and performance tracking. The adoption of a strategic approach, emphasizing key metrics, and the utilization of electronic medical record (EMR) smart phrases emerge as pragmatic measures to streamline documentation processes and systematically monitor pertinent data [1].

**Methods:** We prioritized two key improvement metrics—mobility goals and pressure ulcer prevention. The ICU leadership designed and implemented the following steps:

- Novel user-friendly EMR smart phrases for comprehensive data capture and best practices were created.
- Clear communication on the rationale behind these metrics to improve staff understanding.
- Implementation of training sessions and regular audits to ensure consistent and correct usage.
- The nurse documented daily patient status; if the prescribed interventions for mobility and pressure ulcer prevention were not met, then barriers were reported.
- Maintaining data accuracy with a feedback loops of email reports and meetings.
- Celebrated achievements and addressed improvement areas.
- Encouraging continuous improvement with staff feedback on smart phrase usability.
- Optimization of rounding strategies for effective communication and goal attainment.
- Regular huddles keeping everyone informed, fostering a culture of transparency and collective engagement.

**Results:** After 6 months, nursing staff compliance with smart phrase use rose from 60 to 96%. Mobility goal attainment increased from a 6-month average of 50.6–77.4% (Figure), and pressure ulcer incidence dropped from 1.6/month to zero.

**Conclusions:** By focusing on specific metrics and utilizing smart phrases in the EMR, you can streamline data collection and make it easier for staff to track and report on performance. Regular communication and a commitment to continuous improvement will be crucial in navigating the challenges of a high-volume, high-acuity ICU with a dynamic workforce.

**Reference**

1. Kieffer et al. J Perinat Med. 2023;51:956–61

**Figure (abstract P260):**
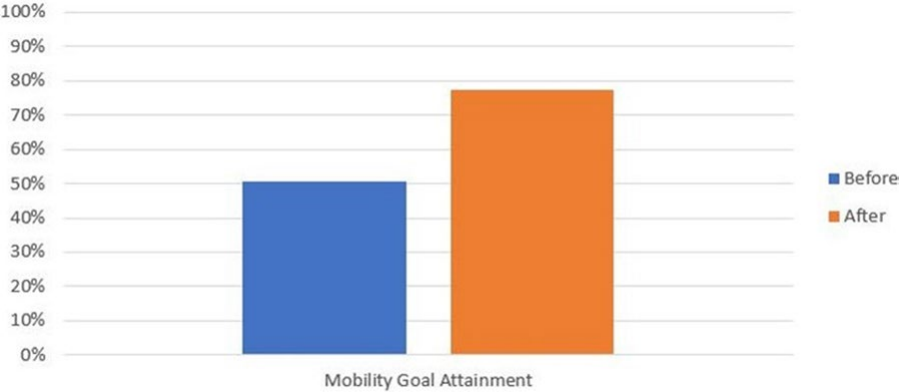
Improvements in 6 month mobility goals with smart phrase implementation.

## P261 Palliative care screening tool: a necessary and essential instrument to intensivists

### A Fonseca Barbosa^1^, D Dias^2^, J Patrício^1^, C Araújo Costa^1^, Â Simas^1^, C Simões Pereira^1^

#### ^1^Hospital Beatriz Ângelo, Serviço de Medicina Intensiva, Loures, Portugal, ^2^Hospital do Espírito Santo de Évora, Serviço de Medicina Interna, Évora, Portugal

*Critical Care* 2024, **28(Suppl 1):** P261

**Introduction:** In recent years, ICU admissions during the last month of life have risen by up to 30% [1]. Palliative Care (PC) in the ICU improves quality of life and, particularly end-of-life (EOL) care, by anticipating and addressing suffering when curative treatments are no longer indicated or effective. The benefits of PC in the ICU are clearly documented: improves symptom management and quality of life, shortens ICU and Hospital length of stay (LOS), and limits non-beneficial life-sustaining interventions [2]. Nevertheless, research highlights clinicians’ inaccuracy identifying PC needs and recognizing EOL patients. Recent evidence supports using tools like the Palliative Care Screening Tool (PCST), allowing early identification of patients in need of palliative care.

**Methods:** Retrospective observational study at a Portuguese ICU over a period of 2 months. The aim was to quantify the prevalence of palliative care requirements, analyze and compare the 90-day and hospital mortality rates, length of stay, level of care, re-admissions and do-not-resuscitate (DNR) prescription. The PCST was employed to assess palliative care needs prevalence.

**Results:** Among 135 ICU patients, 40.7% needed palliative care. 38.2% were in level 3 care. There was no difference in hospital or 90-day mortality between the two groups. PC group had longer hospital LOS, while ICU LOS showed no difference. Remarkably, 90% of readmissions were from the PC group (*p* < 0.001). Only 30.9% had electronically prescribed DNR directives (*p* < 0.001).

**Conclusions:** The high rate of ICU admissions (40.7%) requiring PC highlights the need for better patient recognition and management. Tools like PCST aid clinical decision-making and assist clinicians in identifying appropriate levels of intervention. Future large-scale studies assessing morbidity and 6 months and 1-year mortality can provide deeper insights on palliative patients' needs and outcomes.

**References**

1. Mercadante S et al. BMC Anesthesiol. 2018;18:106
2. Pan H et al. Intensive Care Res. 2023;3:77–82

## P262 Perception of barriers to implementation of patient- and family-centred care: a survey in low- and middle-income countries

### DJ Doyle^1^, K DeMontille^2^, FA Torres^3^, T Al-Musawi^4^, S Sulaiman^5^, YC Yeh^6^, VA Rojas^7^, S Chetty^8^

#### ^1^Case Western Reserve University, Anesthesiology, Cleveland Clinic Lerner College of Medicine, Cleveland, USA, ^2^Pfizer Medical, Hospital and Sterile Injectables, Dubai, United Arab Emirates, ^3^Finis Terrae University, Las Condes Clinic Santiago, Santiago, Chile, ^4^Al Salam Hospital, Intensive Care Unit, Al-Khobar, Saudi Arabia, ^5^National Heart Institute, Institut Jantung Negara, Kuala Lumpur, Malaysia, ^6^National Taiwan University Hospital, Department of Anaesthesiology, Taipei, Taiwan, Republic of China, ^7^Hospital Clínico Universidad de Chile, Critical Care Unit, Department of Medicine, Santiago, Chile, ^8^Stellenbosch University, Anaesthesiology and Critical Care, Faculty of Medicine and Health Sciences, Cape Town, South Africa

*Critical Care* 2024, **28(Suppl 1):** P262

**Introduction:** This study assessed and evaluated barriers to implementing patient- and family-centered care (PFCC) in intensive care units (ICUs) in Asia, Africa, Latin America, and the Middle East. PFCC is a compassionate approach to ICU care, where patients and families are essential members of the healthcare team. The benefits of PFCC are well known, but most publications and research derive from the global North and little is known about PFCC practices and barriers in low- and middle-income countries (LMICs). In ICU, the complexity and vulnerability of the patient’s condition, family involvement, and work requires a high level of culturally sensitive practices.

**Methods:** A 21-question survey based on international guidelines for FCC in neonatal, pediatric, and adult ICUs and a report from the task force of the World Federation of Societies of Intensive and Critical Care Medicine, was conducted in English and Spanish in a convenience sample of ICU HCPs in LMICs. Data were stored on a secure server with encryption and captured using Research Electronic Data Capture. This research was approved by the Human Research Ethics Committee (Ref N22/10/117) of Stellenbosch University.

**Results:** A total of 324 HCPs (Spanish = 66; English = 258) completed the survey from 24 countries across Asia = 47%, Latin America = 20%, Africa = 19% and the Middle East = 13%. The majority were ICU nurses and medical specialists (75%) and 47% worked in ICU for ≥ 10 years. While most indicated familiarity with PFCC (94%), only 56% reported it as part of their daily clinical practice. The commonest barriers to implementation of PFCC were lack of time (58%), shortage of manpower (51%) and concern about infection control (46%).

**Conclusions:** Implementation of PFCC in LMICs can be improved, with further studies needed to overcome barriers and provide a roadmap to enable all stakeholders to benefit from PFCC. PFCC can promote cultural competence and diversity by involving patients and families in care planning and decision making.

## P263 Unlocking Maastricht III: anticipated gains

### D Martins Fernandes^1^, F Dias^2^, A Cardoso-Fernandes^3^, A Silva^4^, C Basílio^2^, N Gatta^2^, R Roncon-Albuquerque Jr^2^, JA Paiva^2^

#### ^1^Intensive Care Department, Areosa, Portugal, ^2^Centro Hospitalar Universitário São João, Intensive Care Department, Paranhos, Portugal, ^3^Unidade Local de Saúde Alto Minho, Internal Medicine Department, Viana do Castelo, Portugal, ^4^Centro Hospitalar Tâmega e Sousa, Intensive Care Department, Penafiel, Portugal

*Critical Care* 2024, **28(Suppl 1):** P263

**Introduction:** To reduce the discrepancy between demand and availability of organs for transplantation, controlled donation after circulatory death (cDCD) has gained momentum and became an accepted practice in many European countries. Despite the prevailing practice of curtailing medical interventions in futile cases, Portugal has yet to implement cDCD [1]. This study aims to predict the expected rise in organ donation from implementing such a program.

**Methods:** Retrospective observational study, conducted in a Portuguese tertiary’s hospital Intensive Care Unit (ICU), during 2019, including all deceased patients under 75 years of age, provided they had no neoplasia, infectious risk or significant renal or hepatic dysfunction. Timing of controlled suspension of treatment was determined upon the documented transition to comfort care. To identify potential donors a maximum agonic phase of 30 min for the liver and pancreas, 60 min for the lung, and 120 min for the kidney were used. Results were compared with donors and collected organs from the same hospital and timeframe.

**Results:** Among 311 deaths, 152 occurred after suspension of organ support. Most of patients were male (67%), with a mean age of 68 years and a mean SAPS II of 57. Mean ICU length of stay was 4 days and mean time from admission to withdrawal of organ support was 1 day. The most frequent form of organ support was invasive mechanical ventilation (74%). In the subgroup analysis 10 individuals (6.6%) died within less than 120 min, potentially providing 3 livers, 3 pancreases, 8 lungs, and 20 kidneys for transplantation. This would result in a 21% annual growth in transplantation activity in this hospital.

**Conclusions:** This study anticipates a significant increase in organ donation from implementing cDCD, with important public health and social impact, aligned with ethical end-of-life care in ICU.

**Reference**

1. Council of Europe European Committee on Organ Transplantation & European Directorate for the Quality of Medicines & HealthCare, 2021.

## P264 Outcomes of critically ill super-elderly patients admitted to intensive care units in a middle-income country: a multicentric cohort study

### G Moralez^1^, G Martins^2^, I Faria^2^, M Pitrowsky^2^, M Balbi^2^, L Bastos^3^, J Salluh^2^

#### ^1^D´Or Institute for Research and Education, Intensive Care, Rio de Janeiro, Brazil, ^2^D´Or Institute for Research and Education, Critical Care, Rio de Janeiro, Brazil, ^3^Pontificia Universidade Catolica - PUC, Industrial engineer, Rio de Janeiro, Brazil

*Critical Care* 2024, **28(Suppl 1):** P264

**Introduction:** CAP admissions are a major cause of ICU for elderly patients. In the present study, we described clinical characteristics, resource use, and outcomes of elderly and super-elderly patients with CAP in 65 ICUs in Brazil.

**Methods:** Anonymized data of patients older than 65y was prospectively collected (2019 and 2022 and 2023, January to July). IRB-approval (CAAE: 17079119.7.0000.5249). We compared elderly and super-elderly according to clinical characteristics, resource use, and hospital outcomes. We analyzed mechanical ventilation use and its outcomes. Descriptive statistics were used for all variables. Parametric tests were used to determine group differences.

**Results:** A total of 18,017 elderly patients were included. Sixty percent were 80 years old or more. Overall hospital mortality was 15%. The super-elderly were sicker at ICU admission, more frequently frail and had higher hospital LOS and mortality (Table). Of the 1920 mechanically ventilated patients, 1014 were women and 906 were men. Elderly people represented 789 (41.1%) and super-elderly 1131 (58.9%). Hospital mortality rates in MV patients were 1209 (63%), 429 (54%) were elderly and 780 (69%) super-elderly. ICU mortality was 1015 (53%), 359 (45.5%) deaths at the elderly group and 656 (58.8%) at the super-elderly group (*p* < 0.05).

**Conclusions:** Overall characteristics were similar among elderly and super-elderly groups. However, super-elderly were more frail and had worse outcomes. We found significant differences in the ICU and Hospital LOS and ICU and hospital Mortality for both the total population and those under mechanical ventilation. Short-term outcomes of the elderly and super-elderly ICU population in a Middle-income country are reasonable. Further studies on quality of life and long-term outcomes are still needed for this population.

**Table (abstract P264):** Clinical characteristics and outcomes

	Elderly (65–79 y) N = 7254	Super-elderly (> 79 years) N = 10,763	*p* value
SAPS 3 points (media/ Sd)	53.67/ ± 9.46)	59.14 ± 8.7	< 0.05
Charlson Comorbidities Index (media/ Sd)	2.11/ ± 2.33	1.97 / ± 1.9	0.573
Frail patients frequency	1209 (16.7%)	2605 (24.2)	< 0.05
Hospital mortality	10.3%	17.9%	< 0.05
Hospital length of stay	13.65 (18.35)	15.38 (19.5)	< 0.05
ICU mortality MV patients	359/789 (45.5%)	656/1131 (58%)	< 0.05
Hospital mortality MV patients	429/789 (54%)	780/ 1131 (69%)	< 0.05

## P265 The old, the bad and the frail

### M Batista, M Barbosa, C Pires, M Amaral, J Henriques, S Costa, R Costa, A Monteiro, J Casimiro, N Germano

#### ^1^Centro Hospitalar Universitário de Lisboa Central, Unidade de Cuidados Intensivos Polivalente - Hospital Curry Cabral, Lisboa, Portugal

*Critical Care* 2024, **28(Suppl 1):** P265

**Introduction:** Intensive care unit (ICU) admission is a weighted decision, specially in patients with chronic disease and older age. These variables may translate into reduced physical, physiological and cognitive reserve, which defines frailty [1]. Its assessment alongside organ dysfunctions can change risk of death stratification and decision to admit.

**Methods:** Retrospective cohort of 578 patients admitted to an ICU between January and December 2022. Patients were divided in two groups concerning frailty status—Clinical Frailty Scale ≥ 4 defined as frail. Baseline characteristics and acute outcomes were analyzed to find predictors of mortality.

**Results:** Frailty prevalence at admission was 54%. Median age was higher in frail group (65 vs. 54 years, *p* < 0.001) and no differences were found in type of admission. Frail patients had higher SAPS II (52 vs. 43, *p* < 0.001) and longer ICU and hospital length of stay (9 vs. 8 days, *p* = 0.40; 22 vs. 18 days, *p* = 0.16). There was a higher vasoactive drug use in frail group (77% vs. 64%; *p* = 0.001), but no differences on mechanical ventilation (MV) (28% vs. 22%; *p* = 0.163) or renal replacement therapy (RRT) (63% vs. 61%; *p* = 0.642). Multivariate analysis revealed that hospital mortality and one-year mortality was significantly higher for frail patients (RR 2.9, 95% CI 1.7, 4.9, *p* < 0.001; RR 2.0, 95% CI 1.3, 3.1, *p* < 0.001, respectively), but no relation with gender, age or type of admission. In the frail group, MV or RRT, but not vasopressors, had also a higher risk of one-year mortality (RR 2.2, 95% CI 1.3, 3.5, *p* < 0.001; RR 4.0, 95% CI 2.4, 6.6, *p* < 0.001; respectively). 5.3% of the frailty group needed nursing homes as of 4.1% in the non-frail group. One year after ICU discharge 49% of the survivors were classified as frail.

**Conclusions:** Frailty is common in patients admitted to ICU and it negatively influences outcomes, mainly after medical interventions, regardless of age. The awareness of this vulnerable population empowers the physician to choose the best approach.

**Reference**

1. Clegg A et al. Lancet 2013;381:752–62

## P266 Integrating palliative care into an intensive care unit: impact on caregivers

### F Lemaitre^1^, C Bielmair^2^, A Denorme^2^, M Dumort^2^, A Pire^2^

#### ^1^CHRSM site Meuse, ICU, Namur, Belgium, ^2^CHRSM site Meuse, ICU nursing, Namur, Belgium

*Critical Care* 2024, **28(Suppl 1):** P266

**Introduction:** Adopting a palliative approach in intensive care allows for prioritizing interdisciplinary collaboration, where the patient is placed at the center of care, taking into their physical, relational, psychological, moral, social, and spiritual needs. It also involves giving meaning to care for healthcare providers, avoiding moral distress and the risk of burnout associated with the administration of deemed inappropriate care.

**Methods:** We organize a weekly meeting with the ICU team and the palliative team. We evaluated all the patients to define a therapeutic project adapted to each one. We studied the impact of this program on the caregivers (5 MDs, 53 nurses, 2 physiotherapists, 1 social assistant) and its impact on the ethical climate in the ICU.

**Results:** 100% of the caregivers participated in the study and responded with yes/no/less (Y/N/L) to questions about end-of-life (see Table).

**Conclusions:** This approach allows for the development of a therapeutic project tailored to each patient, avoiding the initiation or continuation of unreasonable or futile treatments. This has a positive impact on the well-being of healthcare teams through improved communication and a reduction in the loss of meaning. The investigation continues to assess if the results are confirmed at 1-year, in which case, it would be relevant to extend this project to other intensive care services.

**Table (abstract P266):** Results

Have you experienced:	Before % (Y)	After 6 months % (Y/N/L)
Discomfort	90	8.3/21.7/70
Difficulty in communication	78.7	13.6/35/55.9
Lack of consideration for future QOL	86.4	3.3/35/ 61.7
Loss of meaning in your work*	78.7	11.7/38.3/50
Improvement in the sense of work		68.3/18.3/13.3
Impact on decision-making		95.1/4.9
A better-developed project		100

*The results are confirmed among all caregivers. It is noteworthy that the loss of meaning appears early among nurses (66.6% of nurses working for less than 2 years before the project, 20% after)

## P267 End of life in intensive care: Portuguese first data

### DDD Dias^1^, J Patrício^2^, AB Barbosa^2^, ÂS Simas^2^, CP Pereira^2^

#### ^1^Hospital do Espírito Santo de Évora, Medicina, Évora, Portugal, ^2^Hospital Beatriz Ângelo, Serviço Medicina Intensiva, Loures, Portugal

*Critical Care* 2024, **28(Suppl 1):** P267

**Introduction:** A recent study assessed frailty in Portuguese intensive care units (ICU), but the prevalence of patients at end-of-life (EOL) remains unknown [1]. Definition of EOL is used when a patient is likely to die within the next 12 months. Palliative care is not spread among Portuguese hospitals and these patients might not be identified. The aims of this study were to assess the prevalence of EOL patients in our unit and to compare their 90-day and in-hospital mortality rates, days of hospitalization and 30-day re-hospitalization with patients who were not at EOL.

**Methods:** Retrospective and observational study, including patients discharged from April to May/2023. We applied the criteria of Gold Standards Framework—Proactive Identification Guidance (GSF-PIG) organ failure specific indicators to identify EOL patients. We compare EOL group with non-EOL group. Statistical analysis was performed using SPSS®.

**Results:** 135 patients were included. We identified 39 (28.9%) EOL patients and 96 (71.1%) non-EOL patients. No difference was found in sex distribution between both groups. 64.1% male sex in EOL group and 67.7% in non-EOL group, *p* = 0.687. EOL group was older, median age 71 [60;79] years compared to 65 [46;76] years in the non-EOL group. EOL group 90-day mortality was higher 46.22% (n = 18) versus 18.8% (n = 18), *p* = 0.001. In-hospital mortality was also higher in EOL group, 41.0% (n = 16) versus 16.7% (n = 16), *p* = 0.003. There was no significant difference in re-hospitalization, EOL group rate was 12.8% (n = 5) versus 5.2% (n = 5) in non-EOL group. It was not found significant difference in days of hospitalization neither in ICU nor in the ward (Table).

**Conclusions:** The prevalence of EOL patients in our unit was significant and their mortality rates were higher. Surprisingly, there was no difference in re-hospitalization rate or hospitalization days between groups. These are the first data concerning EOL patients in Portuguese ICU.

**Reference**

1. Correia I et al. J Palliat Care. 2021;37:552–561

**Table (abstract P267):** Main outcomes compared

Variable	EOL group 28.9% (n = 39)	Non-EOL group 71.1% (n = 96)	100% (n = 35)
90 days mortality	46.2% (n = 18)	18.8% (n = 18)	*p* value: 0.001
In-hospital mortality	41.0% (n = 16)	16.7% (n = 16)	*p* value: 0.003
30-days re-hospitalization	12.8% (n = 5)	5.2% (n = 5)	*p* value: 0.126
Hospitalization days in ICU	median 4 [2;9]	median 5 [3;9]	*p* value: 0.233
Hospitalization days (total)	median 16 [7;23]	median 14 [8;29]	*p* value 0.723

Significant differences were found in mortality, but not in hospitalization days neither re-hospitalization rate

## P268 Fourteen years of organ donation program at a small district hospital: how and why it changed

### D Lopes, L Pessoa, R Assis, N Catorze

#### Centro Hospitalar do Médio Tejo, Serviço de Medicina Intensiva, Abrantes, Portugal

**Critical Care** 2024, **28(Suppl 1):** P268

**Introduction:** Organ transplants spared thousands of lives and greatly improved the quality of life of many more, regrettably not enough patients will benefit from this procedure. The severe shortage of donors across all organs remains the major constraint.

**Methods:** To evaluate how and why organ harvesting has improved since the beginning of donor program (2009). Observational retrospective study of all patients admitted to the ICU as potential donors. Patients were divided in 2 groups: the effective donors and those who did not fulfil criteria for transplant. Age, gender, admission diagnosis, organs harvested and reasons for refusal were recorded.

**Results:** We enrolled 94 donors, 44 men, mean age of 65 years. Were harvested 217 organs: 3 hearts, 10 lungs, 3 pancreas, 81 livers, 122 kidneys, along with corneas, heart valves, and bones. Haemorrhagic stroke was the major cause of admission (47.3%), followed by ischemic stroke (20.4%), hypoxic ischemic encephalopathy (18.3%) and intracranial hypertension (14.0%). The elderly patients’ prevalence and the Neurosurgery absence, forcing some patients’ transfer to other hospital, were the reasons why neurovascular causes surpass neurotrauma. Brain death criteria not achieved, irreversible organ disfunction, neoplastic disease not excluded and personal opting-out also prevented harvesting on some occasions.

**Conclusions:** Small hospitals with motivated and informed clinicians may support organ donation. Our hospital has invested in qualification regarding detection and good management, achieving 8 clinicians and 3 nurses with Transplant Procurement Management formation. The proximity between ICU and the emergency service, the creation of internal emergency teams and the rapid stroke diagnosis pathway, an alert system based in the NEWS where all patients with neurological depression are referred to the ICU with an emergent text message and the more extended criteria, especially age, are crucial for increasing of donors. Nevertheless, more efforts are needed.

## P269 Organ donation in immigrant population: a retrospective analysis from the most populated region of Italy

### S Marelli^1^, F Pozzi^2^, G Piccolo^3^, M Sacchi^3^, A Chieregato^2^

#### ^1^University Milano Bicocca, Medicine and Surgery, Milano, Italy, ^2^Niguarda Hospital, Neurointensive Care Unit, Milan, Italy, ^3^Coordinamento Regionale Trapianti Lombardia, Lombardia Organ Procurement Organization, Milano, Italy

*Critical Care* 2024, **28(Suppl 1):** P269

**Introduction:** The attitude toward post-mortem organ donation is complex and multifactorial and may be influenced by numerous factors. Given the ongoing and emergent phenomenon of immigration, underlying attitude of individuals migrated from other countries is becoming an important part of the donation process.

**Methods:** Retrospective observational study (2019–2023) including all Lombardy donors. Data were obtained from the Lombardy Donor Record System “Donor Manager”. General demographic and descriptive variables were collected. Quantitative variables are expressed as mean ± SD for normal distribution and median (IQR 25–75) for non-normal. Qualitative variables are expressed as percentage.

**Results:** Two hundred forty-nine foreign-born potential donors were considered. Mean age was 45 ± 15 years. Males accounted 64% (n = 160) of the sample. Two hundred thirty-eight (96%) were eligible for donation, refusal rate was 47% (n = 116), with no age or sex difference compared to non-refusal group (*p* = 0.22; *p* = 0.61 respectively). Refusal rate was almost two times higher, compared to Italian-born population (26%). Among eligible foreign-born donors, 65% (n = 154) were extra-EU born and had a higher rate (54% vs. 39% in EU born) of donation refusal (*p* = 0.04). Moreover, by dividing eligible foreign-born donors into different continents we assessed refusal rate, showing a significant difference (*p* < 0.01). Africa was the continent with the highest refusal rate (66%), followed by Asia (53%), Europe (39%) and America (29%) (Figure).

**Conclusions:** In this Italian subset of foreign-donor population the refusal rate was almost doubled compared to the Italian rate, interestingly it is even higher in non EU born immigrants. Our results underline the importance of different social approaches to different cultures, the importance of talking with local foreign communities and building trust in the health institution, from access to care to the possibility of organ donation.

**Figure (abstract P269):**
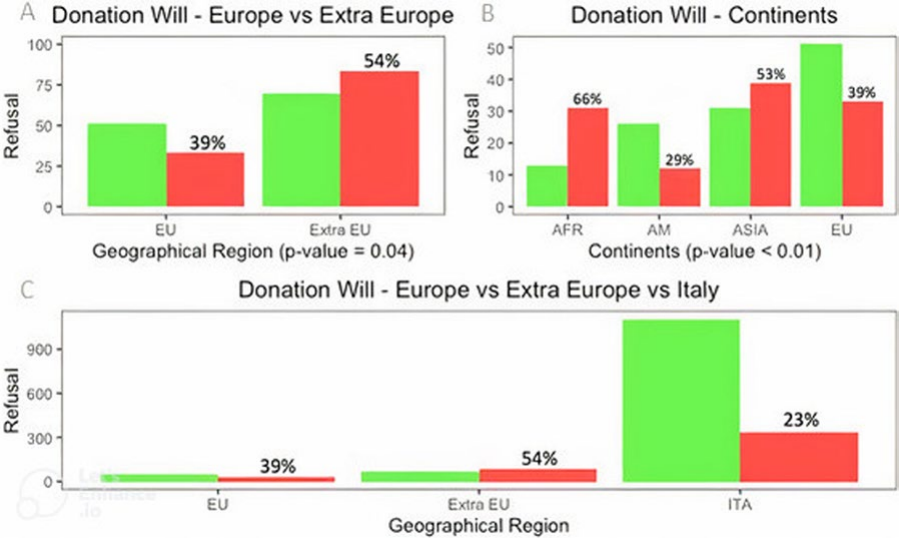
All the plots illustrate refusal (red) and non-refusal (green) rate of eligible donors. EU = Europe; AFR = Africa; AM = America. In plot **A** and plot **B** are displayed foreign-born donors divided by Europe and Extra-Europe born and by continent of birth. In plot **C** we wanted to show the comparison of refusal rate between foreign-born donors and Italian donors.

